# Projected Impacts of Climate and Land-Use Change on the Global Diversity of Birds

**DOI:** 10.1371/journal.pbio.0050157

**Published:** 2007-06-05

**Authors:** Walter Jetz, David S Wilcove, Andrew P Dobson

**Affiliations:** 1 Division of Biological Sciences, University of California San Diego, La Jolla, California, United States of America; 2 Ecology and Evolutionary Biology Department, Princeton University, Princeton, New Jersey, United States of America; 3 Woodrow Wilson School, Princeton University, Princeton, New Jersey, United States of America; Imperial College, United Kingdom

## Abstract

Over the past few decades, land-use and climate change have led to substantial range contractions and species extinctions. Even more dramatic changes to global land cover are projected for this century. We used the Millennium Ecosystem Assessment scenarios to evaluate the exposure of all 8,750 land bird species to projected land-cover changes due to climate and land-use change. For this first baseline assessment, we assumed stationary geographic ranges that may overestimate actual losses in geographic range. Even under environmentally benign scenarios, at least 400 species are projected to suffer >50% range reductions by the year 2050 (over 900 by the year 2100). Although expected climate change effects at high latitudes are significant, species most at risk are predominantly narrow-ranged and endemic to the tropics, where projected range contractions are driven by anthropogenic land conversions. Most of these species are currently not recognized as imperiled. The causes, magnitude and geographic patterns of potential range loss vary across socioeconomic scenarios, but all scenarios (even the most environmentally benign ones) result in large declines of many species. Whereas climate change will severely affect biodiversity, in the near future, land-use change in tropical countries may lead to yet greater species loss. A vastly expanded reserve network in the tropics, coupled with more ambitious goals to reduce climate change, will be needed to minimize global extinctions.

## Author Summary

Land conversion and climate change have already had significant impacts on biodiversity and associated ecosystem services. Using future land-cover projections from the recently completed Millennium Ecosystem Assessment, we found that 950–1,800 of the world's 8,750 species of land birds could be imperiled by climate change and land conversion by the year 2100. These projections are based on the assumption that birds will not dramatically shift their ranges in response to a changing climate, a process that would lessen the range contractions we predict. While climate change will be the principal driver of range contractions at higher latitudes, our projections reveal that land conversion (e.g., deforestation, conversion of grasslands to croplands, etc.) will have a much larger effect on species that inhabit the tropics. This is because birds in the tropics are especially diverse and tend to have small ranges, making them particularly vulnerable to extinction; in contrast, birds at higher latitudes are less diverse and tend to have large ranges. A vastly expanded reserve network in the tropics, coupled with more ambitious goals to reduce greenhouse gas emissions and monitor biodiversity impacts, will be needed to minimize global extinctions.

## Abstract 3

Evaluating changes in range size of land bird species using Millenium Ecosystem Assessment scenarios reveals that land conversion, as well as climate change, will lead to the decline of many species, particularly those in the tropics.

## Introduction

Accelerated climate change and the destruction of natural habitats through direct human activities are two of the greatest threats to terrestrial biodiversity. In recent decades, they have led to substantial range contractions and species extinctions [1–5]. Even more dramatic environmental change is projected for this century [6–8]. Substantial evidence emphasizes the importance of human land-use changes as a cause of species declines and extinctions [5,6,9–12]. Recent studies have highlighted existing and future impacts of human-induced climate change on species persistence [2–4,13,14] and have stressed climate change as a primary concern for the setting of conservation priorities [15,16]. Most of these studies have been based on data collected in the temperate zone, where climate change is predicted to be more pronounced. To date, there have been no global forecasts of the relative and synergistic effects of future climate change and habitat loss on vertebrate distributions. Moreover, our conceptual understanding of what makes some regions and species vulnerable to one threat or the other is still limited. We integrated the exposure of species to climate and land-use change through the combined effects of these drivers on global land cover and explored the resulting reductions in range size and possible extinctions within the world's 8,750 terrestrial bird species. For this first global assessment, we used the simplifying yet transparent assumption of stationary geographic ranges, which allows us to quantify risk in terms of the projected vegetation changes across a species' current range. Although this assumption yields worst-case projections and a number of factors could modify the local details and timeline of our projections, we think the general picture that emerges is robust: a clear and striking geographic disjunction between the relative impacts of future habitat loss and climate change on global avian diversity.

We used the Millennium Ecosystem Assessment (MA) global scenarios to provide examples of possible environmental futures [8,17,18]. The four MA scenarios use plausible ranges of future greenhouse gas emissions and human population and economic growth to estimate how much of a region will be affected by anthropogenic climate change and agricultural expansion. They are characterized by their different approaches to development and ecosystem management. With respect to development, two scenarios (Global Orchestration and TechnoGarden) assume the world becomes increasingly globalized; the other two (Order from Strength and Adapting Mosaic) assume it becomes increasingly regionalized. With respect to ecosystem management, two scenarios (Global Orchestration and Order from Strength) are reactive; they assume that environmental problems causing the breakdown of ecosystem processes are addressed only after they occur. The other two (TechnoGarden and Adapting Mosaic) assume such problems are managed more proactively.

The modeling framework that is part of the MA integrates the interacting effects of future climate and land-use changes and forecasts expected changes to the geographic occurrence of 18 natural and human-made land-cover types [17,19,20]. These expected changes are separated into those due to climate change (change from one natural to another natural land-cover category) and those due to direct human land-use change (change from natural to human-caused land-cover type). We overlaid the geographic occurrence of these land-cover changes with bird distribution data to estimate the areas transformed to a different habitat and thus presumably lost across each species' global range. In the absence of extensive worldwide surveys, we used refined species extent-of-occurrence maps that minimize range overestimation. We recognize that range maps are a scale-dependent abstraction of species' actual occurrence [21] that limit interpretation at fine geographic scales. However, assuming there are no dramatic geographic or ecological trends in range overestimation, this approach yields reliable and urgently needed insights into the impact and interplay of the two major threats to biodiversity at the global scale.

## Results

**Figure 1:**
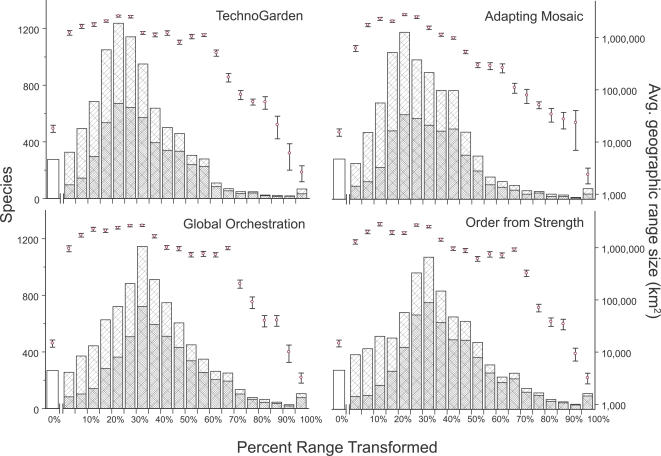
Projected Range Transformations Frequency distribution of projected range transformations for 8,750 bird species by 2100 across the four MA socioeconomic scenarios (histograms, left axis). Within each bar, the height of the shading refers to proportion of projected transformation due to habitat conversion (dark gray) or climate change (light gray). The count of species with exactly zero range transformations is represented as open bars and separated by a gap from the bars representing >0 to ≤100% range transformation. Points with error bars give the average (± SE) current geographic range size (in km^2^) for species in each range transformation category. Although none of the scenarios is likely to predict the actual pattern of land-cover change, they provide “broad confidence limits” that are likely to span the range of possible futures. The four scenarios are briefly described in Materials and Methods.

Range loss varies dramatically across species in all four MA scenarios (Figures 1 and S1). The mean expected range contraction across all scenarios by the year 2050 is 21–26% (depending on scenario) and rises to 29–35% by 2100 (arithmetic means). In the less environmentally conscious scenarios, ~400–900 bird species are projected to have over 50% of their current range transformed to a different habitat by 2050 (Tables 1 and S1); this number roughly doubles by 2100. The species that show minimal loss of range are wide-ranging species, confirming that large ranges provide a buffer against environmental change (Figures 1, S1, and S2). In contrast, the largest potential loss of range size occurs among species that have restricted ranges (Figure S2); this fact highlights the double jeopardy for species that already have small population sizes, specialized habitat requirements, and that are exposed to a high risk of extinction from stochastic demographic processes [22].

**Table 1:**
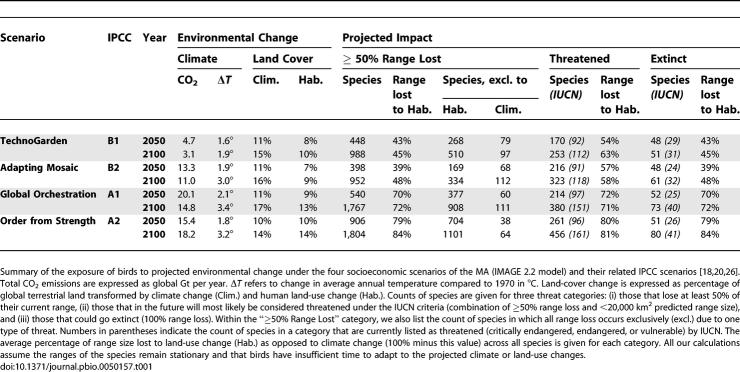
The Exposure of Birds to Projected Environmental Change

Small population or range size and rapid loss of habitat are among the characteristics that formally characterize Red List species under grave threat of extinction [23,24]. Under all four MA scenarios, roughly 170–260 species are projected to experience substantial (i.e., a greater than 50%) range declines that lead to range sizes of less than 20,000 km^2^ by 2050 (an additional 83–195 species are projected to experience this by 2100). Should this occur, then under the “restricted distribution” criterion of The World Conservation Union (IUCN) (Criterion B), they would likely be classified at least as “vulnerable” in the future (and as “near threatened” now), due to their small range sizes combined with continued decline and range-wide threat (see [25] for further discussion). Fewer than half of the species identified in this way are currently listed by IUCN. Under these criteria the total number of threatened species in the analysis would increase by 19–30% by 2050 and 29–52% by 2100. Moreover, of the 886 species in this analysis that are already listed as threatened, 418–475 are expected to have further range losses of at least 20% by 2050 under all scenarios. The risk of extinction to these species is thus likely to grow significantly.

**Figure 2:**
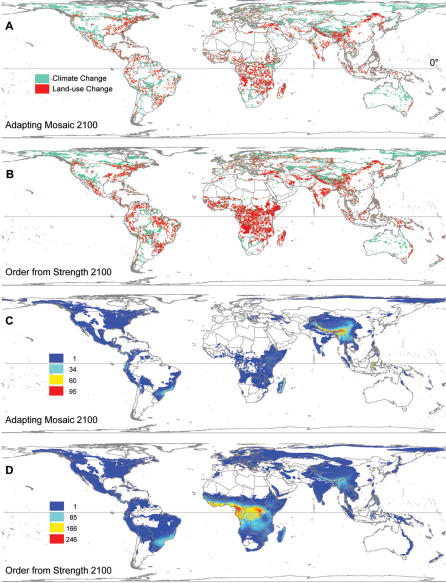
Geographic Patterns and Projected Impact of Environmental Change (A, B) Patterns of change in land cover due to land-use and climate change by 2100. (C, D) The resulting potential impact for birds: the pattern of richness of species with projected range declines ≥50%. This represents the summed, current-day occurrence of qualifying species across a 0.5° grid. Patterns are given for the environmentally proactive “Adapting Mosaic” scenario (A, C), and the environmentally reactive “Order from Strength” scenario (B, D). Maps are in equal-area cylindrical projection. Colors in C and D vary from dark blue to dark red, and the legends provide labels for select colors along this continuous scale (minimum, ~1/3, ~2/3, maximum of data).

**Figure 3:**
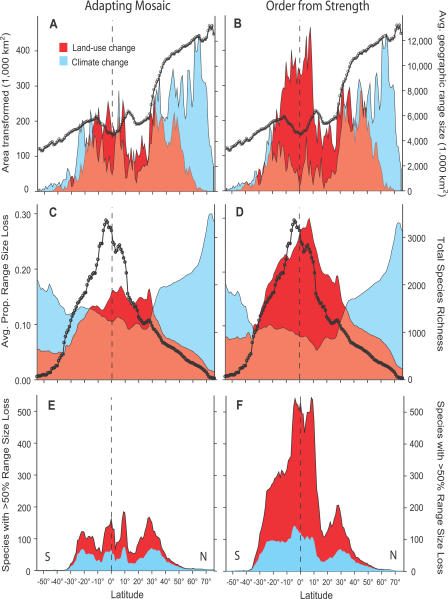
Environmental Change, Avian Biogeography, and Loss in Range Size Projected latitudinal pattern in type of global environmental change, geographic range size, species richness, and the resulting loss in geographic range size (8,750 bird species, 1° bands of latitude). Climate (cyan, on top and semitransparent) and land-use (red) changes between now and 2100 are evaluated for two scenarios: on the left, “Adapting Mosaic” (A, C, E), and on the right, “Order from Strength” (B, D, F). Top (A, B): Total area transformed (area plot, lighter color indicates overlap) and average (± SE) current geographic range size of species per latitudinal band (point and line plot); Middle (C, D): Average proportional loss of range size (area plot, lighter color indicating overlap) and total number of bird species whose range currently overlaps at each latitudinal band (point and line plot). Bottom (E, F): Count of species with ≥50% of range transformed jointly by climate change or land-use change (stacked area plot, coloration indicates the proportion of range area that is transformed by each land change type). Whereas climate change leads to a significant net change of habitat in the polar and temperate regions, the small numbers of bird species that live there on average have very large geographic ranges. Thus, proportional contractions in range size there are much smaller than for the vast majority of bird species that live in the tropics and experience significant reductions in their smaller range sizes due to land-use change. The outcome are many species with significant range reduction in the tropics and subtropics, because of the coincidence of habitat conversion with areas of high species richness. This is particularly the case in the environmentally reactive “Order from Strength” scenario, where large areas of land are converted to agriculture.

We initially use the “Adapting Mosaic” scenario to illustrate the geography of environmental change [18]. This relatively optimistic scenario represents a world that deals proactively with environmental issues; nonetheless, between now and 2100 it projects that approximately 25% of areas currently classified as natural will be transformed—16% due to climate change and 9% due to land-use conversions. Although changes to the land cover are projected to occur globally, there are pronounced regional and latitudinal patterns (Figures 2A and 3A). Changes driven by climate change are strongest in the high latitudes (>30°) of Siberia and North America, a reflection of the greater temperature increases projected for these regions [26]. In contrast, human land-use change dominates at lower latitudes, specifically in Central and South America, central Africa, and portions of India and China; this reflects the importance of forecasted high levels of economic and population growth in these regions. The alternate “Order from Strength” scenario represents a world with only reactive management of environmental issues and projects a transformation of 28% of land, half of which is due to human land-use change; much larger parts of regions such as central Africa are converted to agriculture (Figure 2B). This scenario would result in direct habitat loss in the tropics and subtropics that is approximately double that of the “Adapting Mosaic” scenario (compare Figures 3A and 3B). In both scenarios, range reductions in species that suffer small-to-intermediate proportional range losses are driven more or less equally by climate and land-use change (Figure 1 and Table 1). However, the situation is very different for species that are projected to suffer extensive range losses; these are largely caused by direct human land-use change.

Each projected outcome reflects the covariance between the spatial distribution of the different impacts and the biogeography of bird distributions. Climate change–induced land-cover changes are consistently projected to have the greatest potential impact on species that live far from the equator, in particular in the large northern landmasses, where individual species tend to exhibit very broad distributions and communities as a whole are low in richness (Figures 3 and S3). Bird species that live between 0° and 20° latitude have less than half the geographic range size of birds occurring between 40° and 60° latitude (5.4 × 10^6^ km^2^ versus 11.1 × 10^6^ km^2^, arithmetic means). Average range sizes across bands of absolute latitude increase steadily toward the poles (Spearman rank correlation: *r*_s_ = 0.96, *p* < 0.001, *n* = 75; all 8,750 species), a pattern that is predominantly driven by species in the northern hemisphere (Figure 3A and 3B). The higher latitudes are much poorer in species: only 1,186 species occur above 40° North or below 40° South, but 7,485 (~86% of total) are located between 20° North and South; species counts per 1° band consistently decrease with increasing absolute latitude (*r*_s_ = 0.98, *p* < 0.001, *n* = 75). It follows that the high proportional range loss caused by climate-driven land-cover changes in the high latitudes affects a smaller number of species (Figure 3C and 3D). Conversely, even under the environmentally more benign “Adapting Mosaic” scenario, range loss due to land-use change in tropical and subtropical regions will have potentially devastating consequences for the many, more narrowly distributed species found there (Figure 3A and 3C). Land-use change is responsible for more than half of the range contractions in this scenario, and by itself causes twice as many species to lose over half their range (Figure 3E and Table 1). In the case of the “Order from Strength” scenario, land-use changes below 20° latitude are almost double the magnitude observed in the “Adapting Mosaic” scenario and coincide with a dip in range size and peak in species richness; this concomitance of tropical land-use change and many species with small ranges predicts the dramatic numbers of species potentially experiencing ≥50% range loss (Figure 3B, 3D, and 3F).

The projected impact of environmental change differs markedly across the four socioeconomic scenarios (Table 1, Figures 1 and S1). These differences are mostly driven by the variation in the magnitude of land-use change between scenarios, which is greater than the variation in projected climate change (Table 1). Projected exposure roughly corresponds to the economic and ethical values attributed to biodiversity and ecosystem services in each scenario. Scenarios that represent reactive environmental management lead to greater range losses, largely due to direct human land transformation. Conversely, scenarios that focus on environmental protection or technological solutions to environmental problems result in fewer species suffering major range contractions; of these, between one quarter and a half are primarily affected by climate change driven land-cover changes, depending on whether average area loss or an exclusive 50% range loss threshold is used as an indicator (Table 1). Consistently across all scenarios, the regions with the highest number of species suffering dramatic range contractions are Central America, southeastern Brazil, eastern Madagascar, and the Himalayan highlands (Figure 2C and 2D). All of these have been identified as key biodiversity hotspots in analyses using a diversity of taxa and contemporary rates of habitat loss [27]. Our projections suggest that the Andes and central Africa will also deserve increased attention from conservationists due to high projected levels of habitat loss.

## Discussion

The evaluation we have made of species' exposure to climate change is based on changes in land cover and relies on the well established dependence of land cover on climatic conditions. Our evaluation is transparent and avoids many of the potential conceptual and methodological pitfalls inherent in more complex approaches. But the approach presented here makes some important assumptions: we assume that birds exhibit persistent habitat associations and are limited in their dispersal. In some cases, habitat specialists may be affected by habitat changes finer than those registered by the available land-cover categorization, which would cause us to underestimate climate change impacts for these species. Conversely, range shifts may alleviate the projected impact of climate change [2,28,29] (and thus increase the relative importance of other threats). This would cause us to overestimate the impact of climate change except for high-altitude species, which face limited area available for dispersal [30]. Similar responses might also mitigate the impact of human land-use change (but given the geographic separation of impacts, this effect is likely to be small). Unfortunately, identifying more highly resolved climatic niche boundaries and estimating range shifts from spatial data are inherently difficult problems. Similarly, more detailed modeling of extinction risk would require us to make critical assumptions about ecological interactions between species, crucial niche components, and changes in potential habitat barriers. Even though notable progress has been made, there is still a lack of general consensus on which of the available modeling approaches provides the best insights given the data limitations for most tropical species [31,32]. Furthermore, more detailed models of interactions between climate and land-use change should ideally consider other threats such as infectious diseases, species invasions, and increased persecution which are likely to additionally impact the loss of populations. On a more optimistic note, species currently recognized as specialists may adapt to new habitats including those created under some forms of human encroachment [33,34]. Habitats such as regrowth forests, which our analysis counted as lost to primary forest species, may in fact be able to support at least some of the original species pool [35]. Similarly, while refined range maps were used in this analysis, not all parts of the current and projected range will be fully occupied; this will inevitably result in an underestimation of the impact of environmental change for a significant proportion of species, particularly those with specialized niches and heterogeneous distributions across their current geographic range. We acknowledge that further understanding and modeling of these issues is crucially important for accurate predictions at a fine scale. There clearly is need for further broad-scale studies that develop individual species models while exploring the sensitivity of results to assumptions and methods. Complementary progress will come from detailed studies limited to focal regions and few taxa that carefully estimate as many factors as possible potentially driving range shifts, contractions, and adaptations. However, given the detailed information required for such analyses, these studies are unlikely to provide timely advice to decision-makers who must grapple with the issues of climate change and anthropogenic habitat loss now. Further broad-scale work is needed to explicitly model loss of habitat along elevational gradients, assess threats to long-distance migrants [36], and to additionally take into account the global reserve network that may successfully protect against land-use but not climate change impacts.

This study is the first one to our knowledge to investigate exposure to climate change for a full, species-rich clade across the whole world and the first one to concomitantly evaluate the effects of direct land-use change. Our results show notable differences to previous studies [e.g. 15]; this may be due to their assessment of only climate change, methodological differences, and the restriction of the majority of these studies to mostly temperate species (which are projected to experience highest temperature changes). Our results suggest that the impact of climate-change induced land-cover changes on range sizes in birds will likely be considerable. However, habitat loss in economically emerging tropical countries will continue to pose an even more direct and immediate threat to a greater number of bird species. Although the geography, magnitude, and type of impact will depend critically on the socioeconomic pathways different nations choose to follow, even the most optimistic scenarios lead to substantial range contractions of species, especially of those already vulnerable to extinction because of their current restricted ranges. Only by rapidly expanding the network of protected areas in the tropics can we hope to prevent hundreds of species from becoming imperiled or even extinct. The scenarios that proactively acknowledge that the natural environment provides crucial services to the human economy seem likely to conserve both a higher quality of life for the human population and a higher diversity of species.

## Materials and Methods

### Species and distribution sources.

We evaluated the effect of projected land-cover changes on the breeding distributions of 8,750 species of land birds (out of 9,713 total), excluding water birds and endemics of small oceanic islands that were to small to be included in the MA projections (see Tables S2 and S3 for lists of included and excluded species, respectively). The classification of species follows Sibley & Ahlquist [37] for nonpasserines and Barker et al. [38] for passerines and was updated for newly described species and recent splits and lumps. Distributions were compiled from the most accurate sources giving expert opinion range (extent of occurrence) maps for a given broad geographic region or taxonomic group (see Figure S4 and Table S2 for details). Essentially the same sources were used by [39]. Originally in polygon format, the maps were re-sampled to 0.01° resolution in geographic projection for further analysis.

### Refined extent of occurrence maps.

Extent of occurrence maps are the only type of distributional information available at global scales. By definition, they overestimate the area of occupancy [40] potentially resulting in a dramatic underestimation of proportional losses in geographic range. To address this issue, the extent of occurrence maps for this analysis were refined by clipping from the rasterized species' range maps those habitat types that are definitely unsuitable for the species. We subjected range maps (0.01° resolution, geographic projection) to two clipping steps with finer resolution environmental datasets (in geographic projection). We set the analysis resolution to that of the range maps: whenever the environmental data layers indicated the majority of a 0.01° range map grid cell as unsuitable, it was deleted from a species' range. In the first step, we clipped off elevations outside the maximum or minimum observed for the species (data available for 4,726 species [41]) using the GTOPO30 digital elevation model at 0.0083° resolution [42]. For the second step we first compiled potential habitats listed for species from the literature ([41]; 3,472 different habitat descriptions available across all 8,750 species). We then linked the recorded habitats to one or more of the most representative 22 habitat categories used in the Global Land Cover 2000 database land-cover classification [43]. This resulted in a list of potential land-cover categories occupied by each species. Finally, each species' range map was overlaid with the GLC2000 land-cover map (in geographic projection at 0.0089° resolution, i.e., ~1 km^2^ at the equator), and all land-cover categories not listed for a species were clipped from the range. Together, these steps caused a mean reduction of 21.5% (standard error 3.5%) of range area compared to the original extent of occurrence maps, while incurring only minimal false absences (generally below 1%, [Jetz, unpublished data]). Qualitatively similar results were gained with unrefined extent of occurrence maps, but they reveal smaller proportional range losses and thus tend to underestimate counts of potentially threatened species (Table S1).

To calculate a species' geographic range size, we first projected a map of the world in geographic projection and 0.01° resolution to equal area projection and calculated the true area (in km^2^) of each 0.01° grid cell. Geographic range size was then given by the summed area of all 0.01° grid cells occupied by a species. The MA land-cover projections are only available at a coarser resolution (0.5°, geographic projection). Therefore, we separately recorded the summed area (in km^2^) of all 0.01° grid cells occupied by a species in each MA 0.5° grid cell. This data then formed the basis for the calculation of proportional range transformations outlined below. All overlays and map calculations were performed using the ESRI Arc and Grid software (V. 9.0; ESRI 2004, http://www.esri.com).

### The MA scenarios.

The MA developed four scenarios that could be used to examine and compare changes in land use and global climate under a variety of deliberately diverse and different social and political futures. They were developed to compare four possible extreme conditions in the year 2050 and also provide extrapolations to 2100 [8,18, see 44 for discussion of the importance of region and scale]. The scenarios are not predictions; their principal utility is to delineate the range of possible futures. The four scenarios can be briefly described as follows [8,18]: (i) Adapting Mosaic. In this scenario, regional political responses and economic activity are focused within each major watershed. Local institutions are strengthened and local ecosystems managed proactively. Economic growth is initially low but increases with time. Human population levels approach those estimated for the scenario with the highest rate of human population growth. (ii) Order from Strength. This scenario represents a regionalized and fragmented world that is concerned with security and protection; it pays little attention to public goods and takes a reactive approach to environmental problems. It has the lowest economic growth rates of the four scenarios (they even decrease with time), but these are combined with the highest human population growth rates. (iii) TechnoGarden. This scenario depicts a globally connected world that relies strongly on environmentally sound technology. Ecosystems are increasingly dependent upon technological fixes. Economic growth is relatively high and accelerates, while human population settles into the midrange of projections. (iv) Global Orchestration. Under this scenario, a globally connected society focuses on global trade and economic liberalization but takes a reactive approach to ecosystem problems. It also takes some strong steps to reduce poverty and inequality by investment in infrastructure and education. This scenario has the highest rate of global economic growth and the lowest human population size by 2050.

### Land-cover projections and proportional range transformations.

The MA scenario evaluations are based on the IMAGE 2.2 model [19], a dynamic Earth-system model that estimates future changes to Earth's land-cover in terms of chains of driving forces, pressures, state, and response variables, covering both the natural environment and the socioeconomic system. The model explicitly integrates the forecasts of direct human encroachment with projections of climate change effects on vegetation physiognomy, based on the BIOME model [45], and considers resulting interactions (for detailed assumptions on vegetation shifts and adaptation speed see [19]). We note that these sort of integrated models will likely experience substantial improvement over the coming decade, and projected hotspots of change may well shift geographically as the field (and knowledge about regional drivers) progresses.

The model provides information on current and future distributions of 18 different land-cover types at 0.5° resolution (66,661 terrestrial grid cells), three of which indicate direct human impact from agriculture or urbanization, *cropland, permanent pasture, regrowth forest* [18,20]. We evaluated changes in land cover between 1985 (the approximate median of time period over which range maps were compiled in the sources used) and 2050 and 2100, respectively. Changes from one of the 15 natural to one of the three human-caused land-cover types were considered as transformations due to land-use change for all but the 822 bird species tolerant of at least minor human encroachment (following the habitat preference analysis presented above). Changes from one natural to another natural land-cover type without direct human impact were considered as transformation due to climate change. In some cases (<10% of total grid cell transformations), the new habitat was among those registered suitable for a species. Given the significant perturbation that most habitat transformations create, we counted this grid cell as lost for the species. Although this approach technically overestimates the potential impact of climate change, qualitatively it does not affect the results, because almost all of these habitat generalist species are widespread and therefore have small proportional range loss. During the 21st century, some areas are forecast to be exposed to climate change before land-use change and vice versa. Therefore, if a grid cell already experienced land-cover change by 2050, the respective type of change was the one also registered for 2100. The projected land-cover changes were overlaid with species breeding distributions, and for every transformed 0.5° grid cell, the occupied range area registered for this cell and species was subtracted from the original range size. The remaining untransformed range area was calculated to 1-km^2^ resolution and compared to the original range size.

### Assumptions.

Our approach allows a global and integrated perspective of the effects of land-cover changes driven by climate change and the effects of land-use change on biodiversity. It has the advantage of being transparent and useful for a large number of species. Additionally, it is not fraught by the data limitations and possible methodological pitfalls that are associated with the attempt to quantify species' exact environmental niche and potential for adaptation. Some recent predictions of climate-related extinctions have relied on developing climatic correlates of current species distributions and evaluating potential shifts in these “bioclimatic envelopes” to estimate range loss [15,16]. Our method assumes core habitat associations and evaluates the proportion of a species' range that will be transformed to unsuitable habitat by climate change. It uses well substantiated characterizations of species' persistent habitat requirements. Species range shifts in response to changing climate will likely lower the estimated proportional range loss attributed to it. The alleviating effect regarding the impact of land-use change is more equivocal and likely smaller, given that the direction and magnitude of shifts are uncertain and land-use change is mostly subtropical and tropical (where on the whole, effects of climate change are weaker). Environmental change occurring below the spatial resolution of this analysis (majority area of a 0.5° grid cell, i.e., ~1,540 km^2^ near the equator, ~990 km^2^ at 50° latitude) may lead to further habitat and range loss and thus to threats not evaluated here. Conversely, not all parts of a grid cell may experience the land-cover change projected for its majority, potentially overestimating losses. Nonuniform distribution of abundances within species ranges mean that range contraction does not straightforwardly translate into population loss [46]. Finally, more subtle changes in habitat type too fine for the categorization offered in the MA scenarios may lead to additional range losses, while some versatile species may be unaffected by changes of land-cover types. We acknowledge that these issues require further detailed analyses at regional and single species levels. However, there is no reason to assume that they would introduce a systematic bias into our analyses.

## Supporting Information

Figure S1 Predicted Range Transformations by 2050 Based on Unrefined Extent of Occurrence Ranges (71 KB PDF)

Figure S2 Range Size and Projected Range Loss (97 KB PDF)

Figure S3 Environmental Change, Avian Biogeography, and Loss in Range Size by 2050 and 2100 (793 KB PDF)

Figure S4 Bird Distribution Data Sources (196 KB PDF)

Table S1 Projected Environmental Change and Its Impact on Birds—Based on Unrefined Maps (113 KB DOC)

Table S2 Individual Proportional Range Loss Results for Species Analyzed (8,750 Species). (1.0 MB PDF)

Table S3 Species Not Analyzed (1,125 Species). (793 KB PDF)

## Abbreviations

IUCN: The World Conservation Union
MA: Millennium Ecosystem Assessment
