# Supplementary material for: Projected Impacts of Climate and Land-Use Change on the Global Diversity of Birds
Source: PLoS Biol. 2007 Jun 5;5(6):e157. doi: 10.1371/journal.pbio.0050157 (PMC1885834; doi:10.1371/journal.pbio.0050157)
Supplement: Supplementary file 1 [file pbio.0050157.sg001.pdf]

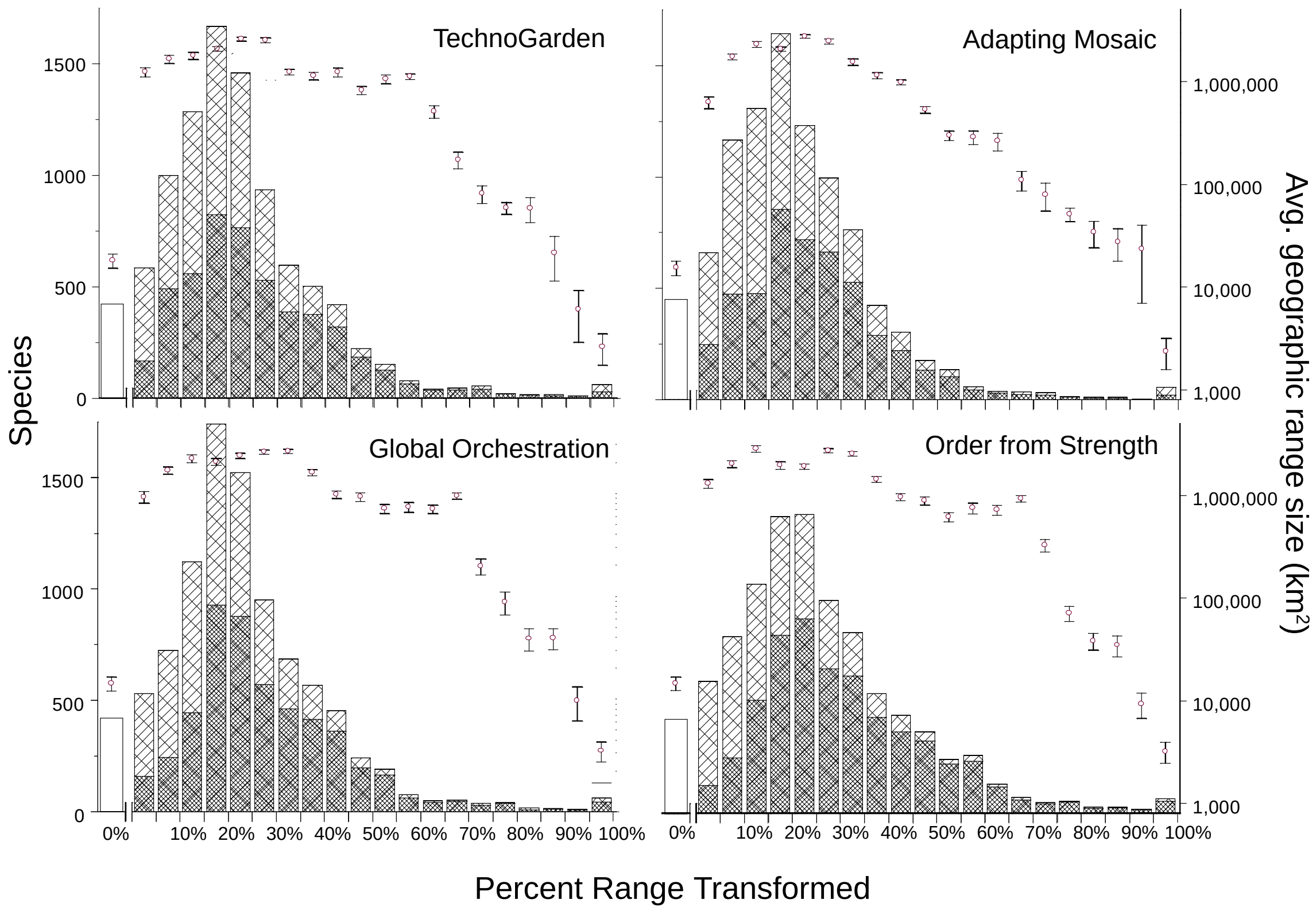

Figure S1: Predicted range transformations by 2050. Frequency distribution of predicted range transformations for 8,750 bird species by 2050 across socioeconomic scenarios (histograms, left axis). In each figure the height of bars refer to proportion of predicted transformation due to land-use change (dark grey) or climate change (light grey). Error bars give the average (± s.e.) current geographic range size (in km^2) for species in each range transformation category.
