# Supplementary material for: Projected Impacts of Climate and Land-Use Change on the Global Diversity of Birds
Source: PLoS Biol. 2007 Jun 5;5(6):e157. doi: 10.1371/journal.pbio.0050157 (PMC1885834; doi:10.1371/journal.pbio.0050157)
Supplement: Supplementary file 2 [file pbio.0050157.sg002.pdf]

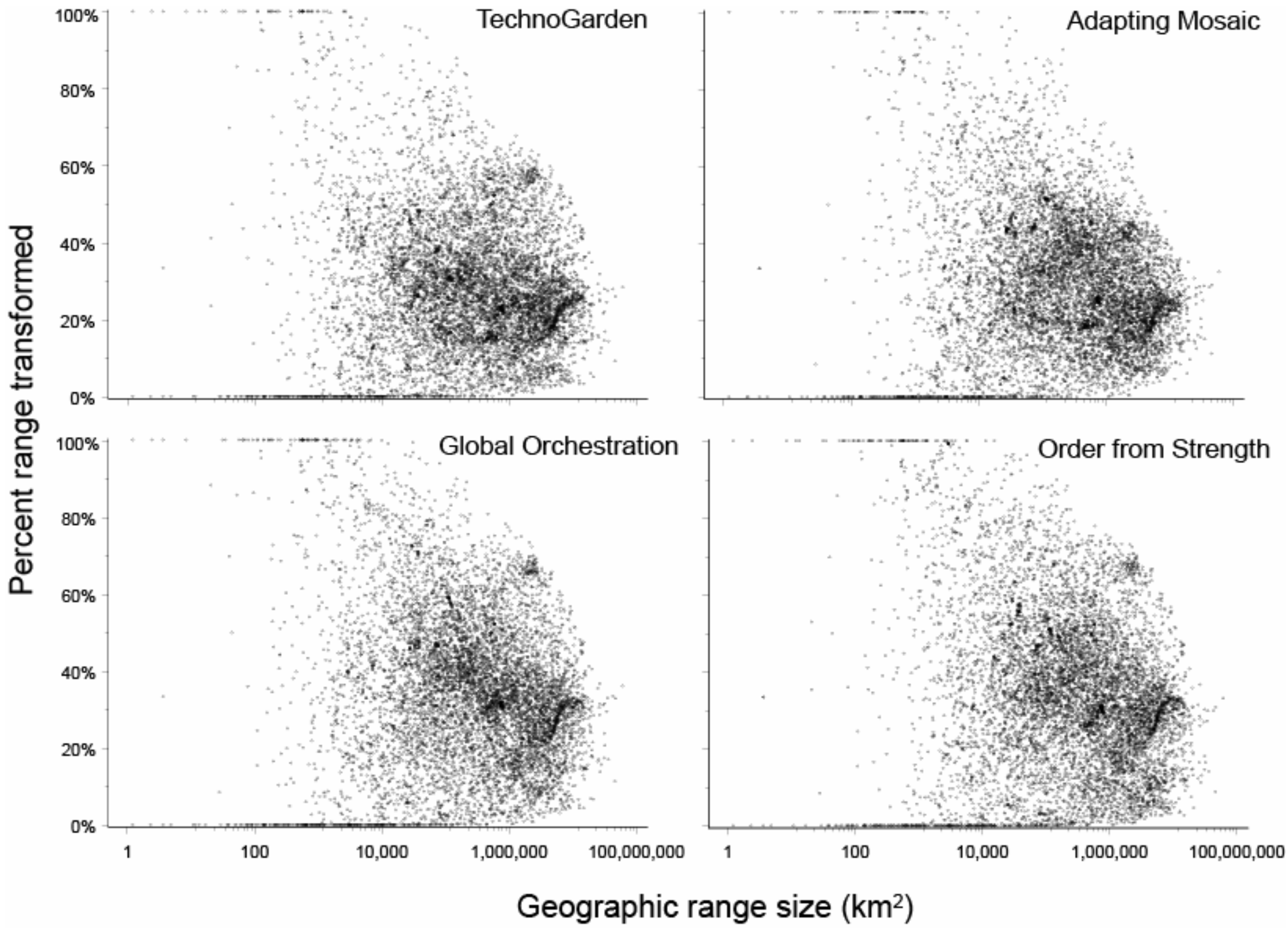

Figure S2: Ranges size and projected range loss. The relationship between current geographic range size (based on refined extent of occurrence maps) and projected range transformation (combined climate change and land-use change) by 2100 across socioeconomic scenarios. Each data point refers to one species.
