# Supplementary material for: Projected Impacts of Climate and Land-Use Change on the Global Diversity of Birds
Source: PLoS Biol. 2007 Jun 5;5(6):e157. doi: 10.1371/journal.pbio.0050157 (PMC1885834; doi:10.1371/journal.pbio.0050157)
Supplement: Supplementary file 3 [file pbio.0050157.sg003.pdf]

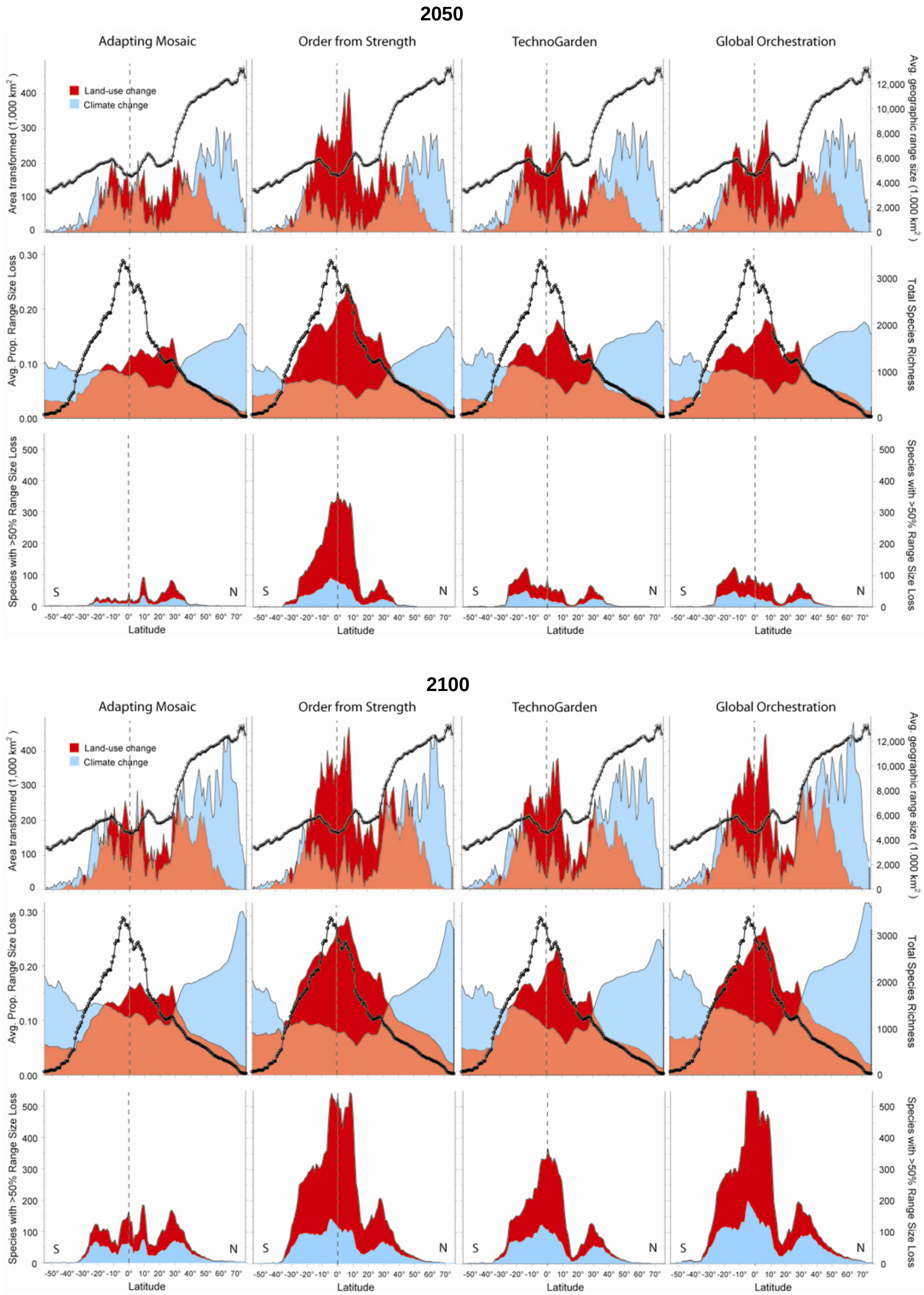

Figure S3: Environmental change, avian biogeography, and loss in range size, by 2050 and 2100. Projected latitudinal pattern in type of global environmental change, geographic range size, species richness and the resulting loss in geographic range size (8,750 bird species, 1° bands of latitude). Climate (cyan) and land-use (red) changes are

evaluated for all four scenarios, between now and 2050 and 2100, respectively. First row: Total area transformed and average (± s.e.) current geographic range size of species per latitudinal band; second row: Average proportional loss of range size and total number of bird species whose range currently overlaps at each latitudinal band. Third row: Count of species with ≥ 50% of range transformed jointly by climate change or land-use change (coloration indicates the proportion of range area that is transformed by each land change type). Note that the six left panels for 2100 are identical with Figure 3 in the printed article.
