# Supplementary material for: Projected Impacts of Climate and Land-Use Change on the Global Diversity of Birds
Source: PLoS Biol. 2007 Jun 5;5(6):e157. doi: 10.1371/journal.pbio.0050157 (PMC1885834; doi:10.1371/journal.pbio.0050157)
Supplement: Supplementary file 5 [file pbio.0050157.st002.pdf]

Table S2: Individual proportional range loss results for species analyzed (8,750 species).

IUCN: 'T' indicates considered 'threatened' by IUCN (Red List, 2004).

Ag: 'H' indicates that species can occur in at least one habitat that has experience direct human impact (agriculture, urbanization). For these, conservatively complete resistance against human land-use change was assumed and range loss only due to climate change was assessed.

R: 'x' indicates currently less than 20,000km^2 refined extent of occurrence range size.

Range Source: Sources on which extent of occurrence map is based (see below).

Percentage loss in geographic range: Predicted percentage loss in refined extent of occurrence due to land-use change (*H*) and climate change (*C*) between present and 2050, 2100 respectively. Four scenarios were evaluated (see Fig. 1): *TG*: TechnoGarden; *AM*: Adaptive Mosaic; *GO:* Global Orchestration; *OS*: Order from Strength. For each species and scenario prediction it is indicated whether the resulting range size was under 20,000km^2 (*x*).

<u>*Range Sources:*</u>

Non-Passerines:

(1)

Hoyo, J. d., A. Elliott, and J. Sargatal 1992. Handbook of the birds of the world. Volume 1: Ostrich to Ducks. Lynx Edicions, Barcelona.

Elliott, A., J. Sargatal, and J. d. Hoyo 1994. Handbook of the birds of the world. Volume 2: New World Vultures to Guineafowl. Lynx Edicions, Barcelona.

Hoyo, J. d., A. Elliott, and J. Sargatal 1996. Handbook of the birds of the world. Volume 3: Hoatzin to Auks. Lynx Edicions, Barcelona.

Hoyo, J. d., A. Elliott, and J. Sargatal 1997. Handbook of the birds of the world. Volume 4: Sandgrouse to Cuckoos. Lynx Edicions, Barcelona.

Hoyo, J. d., A. Elliott, and J. Sargatal 1999. Handbook of the birds of the world. Volume 5: Barn Owls to Hummingbirds. Lynx Edicions, Barcelona.

Hoyo, J. d., A. Elliott, and J. Sargatal 2001. Handbook of the birds of the world. Volume 6: Mousebirds to Hornbills. Lynx Edicions, Barcelona.

Hoyo, J. d., A. Elliott, and J. Sargatal 2002. Handbook of the birds of the world. Volume 7: Jacamars to Woodpeckers. Lynx Edicions, Barcelona.

Passerines:

(2)

Ridgely, R. S., Allnutt, T. F., Brooks, T., McNicol, D. K., Mehlman, D. W., Young, B. E. & Zook, J. R. 2003. Digital Distribution Maps of the Birds of the Western Hemisphere, version 1.0. Arlington, Virginia, USA: NatureServe.

(3)

Snow, D., Perrins, C. M., Gillmor, R., Vincent, D. F. & Wilson, M. G. 1998. The birds of the Western Palearctic. Oxford: Oxford University Press. 2 Volumes.

Cramp, S. 1988. Handbook of the birds of Europe, the Middle East and North Africa : the birds of the Western Palearctic. Volume V, Tyrant flycatchers to thrushes. Oxford University Press, Oxford.

Cramp, S., and D. J. Brooks 1992. Handbook of the birds of Europe, the Middle East and North Africa : the birds of the Western Palearctic. Volume VI, Warblers. Oxford University Press, Oxford.

Cramp, S., C. M. Perrins, D. J. Brooks, and E. Dunn 1993. Handbook of the birds of Europe, the Middle East and North Africa : the birds of the Western Palearctic. Volume VII, Flycatchers to Shrikes Oxford University Press, Oxford.

Cramp, S., C. M. Perrins, D. J. Brooks, and E. Dunn 1994. Handbook of the birds of Europe, the Middle East and North Africa : the birds of the Western Palearctic. Volume VIII, Crows to finches Oxford University Press, Oxford.

Cramp, S., C. M. Perrins, D. J. Brooks, and E. Dunn 1994. Handbook of the birds of Europe, the Middle East and North Africa : the birds of the Western Palearctic. Volume IX, Buntings and new world warblers. Oxford University Press, Oxford.

(4)

Hèue, F. 1970. Les oiseaux du Proche et du Moyen Orient, de la Mâediterranâee aux contreforts de l'Himalaya. Paris: Editions. N. Boubee & Cie. 948 p.

Porter, R. F., Christensen, S. & Schiermacker-Hansen, P. 1996. Field guide to the birds of the Middle East. London. San Diego: Poyser; Academic Press. 460 p.

(5)

Keith, S., E. K. Urban, and C. H. Fry 1992. The Birds of Africa. Vol. 4: Broadbills to Chats. Academic Press, London & New York.

Urban, E. K., C. H. Fry, and S. Keith 1997. The Birds of Africa. Vol. 5: Thrushes to Puffback Flycatcher. Academic Press, London & New York.6. Langrand, O. 1990 Guide to the birds of Madagascar. New Haven, CT: Yale University Press. 364 p.

Fry, C. H., S. Keith, and E. K. Urban 2000. The Birds of Africa. Vol. 6: Picathartes to Oxpeckers. Academic Press, London & New York.

Fry, C. H., and S. Keith 2004. The Birds of Africa. Vol. 7: Sparrows to Buntings. Academic Press, London & New York.

(6)

Langrand, O. 1990. Guide to the birds of Madagascar. New Haven, CT: Yale University Press. 364 p.

(7)

Ding, T.-S. 2005. Distribution of Breeding Birds in East Asia (http://asianbird.zo.ntu.edu.tw/) (National University of Taiwan, Taiwan.

Ding, T.-S., Yuan, H.-W., Geng, S., Koh, C.-N. & Lee, P.-F. 2006. Macro-scale bird species richness patterns of the East Asian mainland and islands: energy, area and isolation. Journal of Biogeography 33, 683-693.

Flint, V. E., N. Bourso-Leland, and A. A. Kuznetsov 1984. A Field Guide to the Birds of Russia and Adjacent Territories. Princeton University Press, Princeton NJ.

Sinclair, I., and O. Langrand 1998. Birds of the Indian Ocean Islands. Struik Publishers, Cape Town.

(8)

Beehler, B. M., Pratt, T. K. & Zimmerman, D. A. 1986. Birds of New Guinea. Princeton, N.J.: Princeton University Press. 293 p.

Coates, B. J. 1985. The birds of Papua New Guinea : including the Bismarck Archipelago and Bougainville. Volume I. Alderley, Qld., Australia: Dove Publications. 464 p.

Coates, B. J. 1990. The birds of Papua New Guinea : including the Bismarck Archipelago and Bougainville. Volume II. Alderley, Qld., Australia: Dove Publications. 576 p.

(9)

Doughty, C. 1999. Birds of the Solomons, Vanuatu & New Caledonia. London: Christopher Helm, A &C Black. 206 p.

(10)

Simpson, K. & Day, N. 2004. Birds of Australia: Princeton University Press. 382 p.

(11)

Pratt, D., Bruner, P. L. & Berrett, D. G. 1987. A field guide to the birds of Hawaii and the tropical Pacific. Princeton: Princeton University Press. 409 p.

(12)

Bull PC, Gaze PD, Robertson CJR, Zealand OSoN 1985. The atlas of bird distribution in New Zealand. Wellington North, N.Z.: Ornithological Society of New Zealand. 296 p.

Heather, B., Robertson, H. & Onley, D. 2005. Field Guide to the Birds of New Zealand: Penguin Books Ltd. 432p.

(13, 14)

Misc. primary sources.

Supplementary Table 2

Family	Species	IUCN	Ag	R	Range Source	Percentage loss in geographic range 2050 TG		AM		GO		OS		2100 TG		AM		GO		OS	
						H	C	H	C	H	C	H	C	H	C	H	C	H	C	H	C
Struthionidae	Struthio camelus				1	15	3	7	8	16	3	18	2	17	4	8	11	18	6	18	5
Rheidae	Rhea americana				1	7	10	5	7	7	8	11	5	10	14	9	10	13	12	18	8
Rheidae	Rhea pennata				1	1	6	1	7	1	7	2	6	2	9	3	13	3	16	5	14
Casuariidae	Casuarius casuarius	T			1	5	5	6	4	11	4	12	3	9	7	14	7	18	6	21	5
Casuariidae	Casuarius bennetti				1	5	3	5	2	18	1	16	1	11	5	13	6	30	4	28	2
Casuariidae	Casuarius unappendiculatu	T			1	2	2	8	1	11	1	11	1	10	2	20	2	19	1	23	1
Casuariidae	Dromaius novaehollandiae		H		1	0	13	0	10	0	12	0	8	0	15	0	14	0	16	0	10
Apterygidae	Apteryx australis	T	H		1	0	13	0	11	0	12	0	10	0	14	0	13	0	18	0	11
Apterygidae	Apteryx owenii	T		x	1	0	0x	0	0x	0	0x	0	0x	0	0x	0	24x	0	24x	0	0x
Apterygidae	Apteryx haastii	T		x	1	0	19x	0	19x	5	19x	0	19x	0	37x	0	57x	5	67x	14	57x
Tinamidae	Tinamus tao				1	9	13	6	15	7	15	13	13	14	16	13	18	23	18	25	16
Tinamidae	Tinamus solitarius				1	19	11	16	9	24	8	27	7	29	17	29	15	42	12	44	13
Tinamidae	Tinamus osgoodi	T			1	2	0	6	0	3	0	8	0	2	0	9	7	9	7	25	0
Tinamidae	Tinamus major				1	6	11	5	11	5	12	9	11	9	12	8	14	15	14	17	13
Tinamidae	Tinamus guttatus				1	4	8	2	7	3	7	6	7	7	8	5	8	12	9	13	8
Tinamidae	Nothocercus bonapartei				1	15	3	14	4	15	4	26	4	21	4	24	8	26	8	37	5
Tinamidae	Nothocercus julius				1	16	2	17	3	18	2	30	2	27	3	35	5	37	2	45	4
Tinamidae	Nothocercus nigrocapillus				1	15	6	14	5	14	5	22	5	22	6	26	7	24	6	36	6
Tinamidae	Crypturellus berlepschi				1	15	4	16	5	18	2	17	3	22	5	20	5	20	5	25	3
Tinamidae	Crypturellus cinereus				1	4	9	3	9	3	9	6	9	7	10	6	11	11	11	12	11
Tinamidae	Crypturellus soui		H		1	0	12	0	11	0	11	0	9	0	14	0	13	0	15	0	12
Tinamidae	Crypturellus ptaritepui				1	3	33	0	37	3	33	0	37	3	33	0	37	3	33	0	37
Tinamidae	Crypturellus obsoletus				1	13	9	10	9	13	8	18	8	21	11	21	11	25	10	31	10
Tinamidae	Crypturellus cinnamomeus				1	12	4	23	2	18	4	30	2	15	4	26	12	23	8	34	4
Tinamidae	Crypturellus undulatus				1	6	13	4	11	4	12	9	10	10	16	8	14	13	16	17	13
Tinamidae	Crypturellus transfasciatus				1	6	8	11	7	13	10	17	8	12	8	19	11	27	11	33	11
Tinamidae	Crypturellus strigulosus				1	5	11	3	10	3	11	8	9	9	12	6	12	15	13	17	10
Tinamidae	Crypturellus boucardi				1	14	4	24	4	17	5	30	3	17	5	31	6	25	7	37	5
Tinamidae	Crypturellus kerriae	T			1	16	0	24	6	14	1	16	5	28	5	25	6	21	10	32	10
Tinamidae	Crypturellus erythropus				1	7	11	5	11	8	10	13	9	12	12	9	14	21	15	21	12
Tinamidae	Crypturellus duidae				1	5	16	0	16	4	16	8	16	5	24	5	39	10	39	10	39
Tinamidae	Crypturellus noctivagus				1	18	11	14	9	22	8	24	7	24	18	24	14	37	12	39	12
Tinamidae	Crypturellus atrocapillus				1	14	23	3	33	5	32	13	28	15	29	9	39	18	39	23	36
Tinamidae	Crypturellus variegatus				1	4	8	3	7	3	7	6	7	7	8	5	9	12	10	13	9
Tinamidae	Crypturellus brevirostris				1	0	9	1	9	0	9	1	9	2	10	2	10	2	12	4	10
Tinamidae	Crypturellus bartletti				1	1	8	2	8	1	8	3	8	5	10	5	10	5	10	10	9
Tinamidae	Crypturellus parvirostris				1	7	17	5	14	6	16	12	12	11	21	9	18	17	20	20	16
Tinamidae	Crypturellus casiquiare				1	0	0	0	0	0	0	0	0	0	0	0	0	0	0	0	0
Tinamidae	Crypturellus tataupa				1	7	15	6	12	8	13	11	10	10	20	10	17	15	18	18	14
Tinamidae	Rhynchotus rufescens				1	8	14	6	10	8	11	12	7	11	18	10	13	15	15	19	10
Tinamidae	Nothoprocta taczanowskii	T		x	1	3	19x	8	0x	9	3x	4	9x	3	19x	10	12x	29	3x	13	9x
Tinamidae	Nothoprocta kalinowskii	T		x	1	3	33x	5	33x	3	33x	36	1x	5	33x	5	33x	3	35x	38	1x

Family	Species	IUCN	Ag	R	Range Source	Percentage loss in geographic range 2050 TG		AM		GO		OS		2100 TG		AM		GO		OS	
						H	C	H	C	H	C	H	C	H	C	H	C	H	C	H	C
Tinamidae	*Nothoprocta ornata*				1	7	13	7	14	5	14	9	12	9	16	10	18	11	20	15	15
Tinamidae	*Nothoprocta pentlandii*				1	5	16	5	18	4	17	8	14	6	22	7	27	9	27	13	21
Tinamidae	*Nothoprocta cinerascens*		H		1	0	9	0	8	0	8	0	7	0	12	0	15	0	18	0	16
Tinamidae	*Nothoprocta perdicaria*		H		1	0	7	0	5	0	6	0	6	0	9	0	10	0	13	0	12
Tinamidae	*Nothoprocta curvirostris*				1	22	4	28	4	13	4	49	0	29	5	47	10	67	8	54	6
Tinamidae	*Nothura darwinii*				1	4	12	4	13	3	13	5	11	6	16	5	20	6	22	9	17
Tinamidae	*Nothura chacoensis*				1	3	9	1	7	4	6	7	7	6	13	4	19	6	28	11	22
Tinamidae	*Nothura maculosa*		H		1	0	9	0	4	0	6	0	3	0	13	0	7	0	10	0	7
Tinamidae	*Nothura minor*	T			1	9	11	7	6	9	9	12	5	13	15	11	6	14	9	17	5
Tinamidae	*Nothura boraquira*				1	5	19	3	9	3	14	8	6	7	26	7	11	8	18	14	8
Tinamidae	*Taoniscus nanus*	T			1	8	11	13	6	12	6	15	6	12	23	15	13	22	11	25	10
Tinamidae	*Eudromia elegans*				1	1	6	0	6	1	7	1	6	1	8	0	12	1	14	1	12
Tinamidae	*Eudromia formosa*				1	0	5	0	5	1	5	1	4	0	8	0	12	3	12	2	11
Tinamidae	*Tinamotis pentlandii*				1	4	11	5	11	3	11	7	9	4	14	7	17	9	17	13	12
Tinamidae	*Tinamotis ingoufi*				1	0	3	0	3	0	4	0	3	0	8	0	9	0	9	1	9
Cracidae	*Ortalis vetula*				1	7	8	14	4	9	6	18	2	8	10	17	21	12	10	21	4
Cracidae	*Ortalis cinereiceps*				1	22	6	36	8	25	11	41	8	30	7	42	10	40	13	53	9
Cracidae	*Ortalis garrula*				1	22	2	16	7	17	2	15	0	26	7	21	11	24	15	26	4
Cracidae	*Ortalis ruficauda*				1	23	5	18	7	21	5	28	3	28	9	27	11	35	14	41	7
Cracidae	*Ortalis erythroptera*	T			1	18	5	6	6	10	9	18	2	21	5	19	10	30	13	23	7
Cracidae	*Ortalis wagleri*				1	14	16	20	7	9	12	41	4	17	18	20	22	12	16	44	5
Cracidae	*Ortalis poliocephala*				1	6	3	21	0	10	2	23	0	7	3	21	12	15	2	24	0
Cracidae	*Ortalis canicollis*				1	6	11	3	12	4	11	9	9	8	14	7	19	12	20	16	16
Cracidae	*Ortalis leucogastra*				1	8	0	17	0	19	0	28	0	11	0	18	2	22	6	30	0
Cracidae	*Ortalis guttata*				1	6	10	5	11	6	11	9	10	10	12	10	14	13	13	17	12
Cracidae	*Ortalis motmot*				1	6	11	4	11	5	11	10	11	11	12	8	14	20	15	19	14
Cracidae	*Ortalis superciliaris*				1	5	13	2	13	7	12	13	9	10	15	5	19	22	21	31	16
Cracidae	*Penelope argyrotis*				1	17	4	16	5	16	4	22	3	23	7	24	10	34	15	33	6
Cracidae	*Penelope barbata*	T			1	2	4	10	3	12	5	16	5	12	4	30	5	25	7	43	5
Cracidae	*Penelope ortoni*	T			1	12	4	18	8	17	4	26	6	20	7	29	11	33	8	29	7
Cracidae	*Penelope montagnii*				1	16	6	12	5	12	4	24	4	22	6	25	8	31	7	39	7
Cracidae	*Penelope marail*				1	6	14	4	13	4	14	7	13	10	15	7	16	15	18	15	16
Cracidae	*Penelope superciliaris*				1	7	17	5	18	6	18	12	16	12	20	9	20	21	21	23	19
Cracidae	*Penelope dabbenei*				1	9	21	16	13	7	16	20	14	9	21	21	13	25	16	29	14
Cracidae	*Penelope purpurascens*				1	14	5	19	5	15	6	25	4	18	6	25	11	25	11	31	6
Cracidae	*Penelope perspicax*	T		x	1	14	11x	14	11x	25	0x	25	11x	14	11x	14	11x	25	0x	25	11x
Cracidae	*Penelope albipennis*	T			1	0	0	0	0	0	0	0	0	0	0	0	0	0	0	0	0
Cracidae	*Penelope jacquacu*				1	4	11	3	12	3	12	6	12	7	13	6	15	9	15	12	14
Cracidae	*Penelope obscura*				1	8	8	8	6	10	5	10	6	10	12	12	9	17	7	17	8
Cracidae	*Penelope pileata*				1	18	29	10	30	9	30	25	24	24	29	20	30	23	31	34	26
Cracidae	*Penelope ochrogaster*	T			1	4	26	0	13	1	21	8	9	7	33	4	17	8	25	16	11
Cracidae	*Penelope jacucaca*	T			1	6	17	5	11	6	14	13	7	7	21	10	12	8	16	20	7
Cracidae	*Pipile pipile*	T		x	1	62	0x	52	0x	62	0x	62	0x	62	0x	52	0x	62	0x	62	0x
Cracidae	*Pipile cumanensis*				1	5	8	3	8	4	8	7	7	9	9	8	12	12	12	15	11

Family	Species	IUCN	Ag	R	Range Source	Percentage loss in geographic range															
						2050								2100							
						TG		AM		GO		OS		TG		AM		GO		OS	
						H	C	H	C	H	C	H	C	H	C	H	C	H	C	H	C
Cracidae	*Pipile cujubi*				1	5	20	3	21	3	22	8	20	9	22	7	22	16	23	15	22
Cracidae	*Pipile jacutinga*	T			1	18	9	18	8	23	6	26	7	27	15	24	17	36	11	38	13
Cracidae	*Aburria aburri*				1	12	4	15	4	14	4	24	4	20	5	24	8	28	6	37	5
Cracidae	*Chamaepetes unicolor*				1	30	7	37	13	27	14	47	12	38	7	39	22	37	18	59	12
Cracidae	*Chamaepetes goudotii*				1	14	6	15	5	14	5	28	4	22	6	28	7	31	7	41	5
Cracidae	*Penelopina nigra*				1	9	2	21	1	21	2	24	1	11	2	21	8	23	3	26	3
Cracidae	*Oreophasis derbianus*	T			1	17	2	27	2	20	2	29	2	19	2	30	2	26	3	31	2
Cracidae	*Nothocrax urumutum*				1	3	4	1	4	1	4	4	4	5	4	5	4	6	4	11	4
Cracidae	*Mitu tomentosa*				1	4	11	2	11	5	10	6	11	7	13	5	16	11	16	10	16
Cracidae	*Mitu salvini*				1	6	1	3	0	4	1	9	0	11	1	11	1	14	1	25	1
Cracidae	*Mitu tuberosa*				1	6	14	4	15	4	15	9	14	10	16	8	17	16	17	17	16
Cracidae	*Mitu mitu*			x	1	1	71x	16	64x	21	50x	32	29x	1	93x	16	73x	27	53x	32	38x
Cracidae	*Pauxi pauxi*	T			1	28	6	20	6	31	3	39	3	34	14	37	15	46	22	54	5
Cracidae	*Pauxi unicornis*	T			1	16	9	8	8	8	8	13	8	24	9	19	15	24	16	26	15
Cracidae	*Crax rubra*				1	16	4	22	5	17	5	30	4	21	5	28	9	28	9	37	6
Cracidae	*Crax alberti*	T			1	16	1	22	5	11	5	25	2	24	2	27	9	35	12	34	5
Cracidae	*Crax daubentoni*				1	25	6	13	10	20	5	25	5	29	10	22	15	40	13	43	8
Cracidae	*Crax alector*				1	3	13	2	13	3	12	5	12	7	14	4	17	10	17	10	16
Cracidae	*Crax globulosa*	T			1	2	12	1	12	2	12	4	12	5	14	5	14	6	14	9	14
Cracidae	*Crax fasciolata*				1	8	18	5	20	6	20	13	19	15	22	10	25	26	26	26	24
Cracidae	*Crax blumenbachii*	T		x	1	19	4x	11	4x	24	0x	23	4x	22	5x	15	12x	35	6x	26	12x
Megapodiidae	*Alectura lathami*				1	3	31	4	16	6	25	6	10	4	40	5	33	8	38	11	13
Megapodiidae	*Aepypodius arfakianus*				1	7	2	5	1	21	1	18	1	13	8	14	9	35	8	36	3
Megapodiidae	*Aepypodius bruijnii*	T		x	1	11	0x	0	0x	0	0x	0	0x	11	0x	11	0x	0	0x	0	0x
Megapodiidae	*Talegalla cuvieri*				1	1	0	0	0	0	0	1	0	2	0	11	0	4	0	6	0
Megapodiidae	*Talegalla fuscirostris*				1	3	4	4	3	14	2	16	1	6	7	11	7	25	6	22	2
Megapodiidae	*Talegalla jobiensis*				1	2	3	3	1	29	1	15	1	12	5	11	7	43	5	33	4
Megapodiidae	*Macrocephalon maleo*	T			1	15	22	28	14	14	20	16	18	19	22	30	28	24	35	29	39
Megapodiidae	*Megapodius nicobariensis*	T		x	1	28	0x	91	0x	79	0x	37	0x	28	0x	100	0x	100	0x	100	0x
Megapodiidae	*Megapodius cumingii*				1	12	12	22	9	15	11	18	11	15	14	27	16	28	23	27	19
Megapodiidae	*Megapodius bernsteinii*			x	1	26	7x	27	5x	13	18x	1	18x	26	13x	30	11x	14	24x	1	24x
Megapodiidae	*Megapodius reinwardt*				1	3	16	2	8	7	14	8	4	4	18	5	14	11	19	11	5
Megapodiidae	*Megapodius freycinet*				1	16	0	20	0	19	0	16	0	28	0	22	0	32	0	29	0
Megapodiidae	*Megapodius affinis*				1	2	4	6	2	27	2	13	2	12	6	13	6	40	3	31	3
Megapodiidae	*Megapodius eremita*				1	14	4	11	1	30	1	33	1	34	7	19	1	55	2	48	1
Megapodiidae	*Megapodius layardi*	T		x	1	4	0x	12	0x	31	0x	10	0x	12	0x	30	0x	47	0x	42	0x
Megapodiidae	*Megapodius wallacei*	T			1	14	0	18	0	21	0	20	0	29	0	19	0	31	0	34	0
Megapodiidae	*Leipoa ocellata*	T			1	0	6	0	5	0	6	0	6	0	8	0	7	0	9	0	7
Phasianidae	*Lerwa lerwa*				1	20	4	22	3	22	3	23	2	22	6	24	10	23	12	24	7
Phasianidae	*Ammoperdix griseogularis*				1	7	3	7	2	8	2	10	2	12	4	10	3	13	3	15	3
Phasianidae	*Ammoperdix heyi*				1	0	1	0	1	0	1	0	1	0	1	0	1	0	1	0	1
Phasianidae	*Tetraogallus caucasicus*				1	8	43	4	46	11	47	4	50	11	51	16	54	21	54	21	51
Phasianidae	*Tetraogallus caspius*				1	8	10	5	10	7	10	10	11	14	12	7	13	15	14	18	13
Phasianidae	*Tetraogallus tibetanus*				1	16	14	17	14	17	13	18	13	17	23	20	30	24	31	25	26

Family	Species	IUCN	Ag	R	Range Source	Percentage loss in geographic range 2050 TG H	C	AM H	C	GO H	C	OS H	C	2100 TG H	C	AM H	C	GO H	C	OS H	C
Phasianidae	*Tetraogallus altaicus*				1	2	7	1	8	2	7	1	8	2	9	2	10	3	11	2	11
Phasianidae	*Tetraogallus himalayensis*				1	21	16	23	15	23	15	23	14	23	27	27	35	31	36	31	30
Phasianidae	*Tetraogallus obscurus*				1	26	1	26	4	27	4	28	2	27	6	28	12	33	7	33	6
Phasianidae	*Tetraophasis szechenyii*				1	45	1	46	1	48	0	46	1	48	3	48	7	51	4	50	5
Phasianidae	*Alectoris barbara*				1	11	2	11	2	10	3	11	2	11	2	11	2	10	3	11	2
Phasianidae	*Alectoris melanocephala*				1	0	0	0	0	0	0	0	0	0	0	0	0	0	1	0	1
Phasianidae	*Alectoris chukar*				1	9	9	9	9	10	9	12	9	12	13	12	18	15	19	17	16
Phasianidae	*Alectoris philbyi*				1	0	0	0	0	0	0	0	0	0	0	0	0	0	1	0	1
Phasianidae	*Alectoris magna*				1	29	8	31	11	32	11	36	8	31	23	34	30	47	18	49	13
Phasianidae	*Alectoris graeca*				1	5	25	5	19	5	24	7	15	10	31	8	25	13	32	8	19
Phasianidae	*Alectoris rufa*		H		1	0	19	0	9	0	15	0	7	0	30	0	22	0	28	0	11
Phasianidae	*Francolinus francolinus*				1	15	4	14	3	15	4	19	3	19	5	18	6	19	7	23	4
Phasianidae	*Francolinus pictus*				1	12	2	13	2	14	1	18	1	14	3	15	3	16	3	24	1
Phasianidae	*Francolinus pintadeanus*				1	8	5	12	5	10	5	11	5	9	9	13	8	12	11	14	8
Phasianidae	*Francolinus pondicerianus*		H		1	0	3	0	3	0	3	0	2	0	5	0	6	0	7	0	3
Phasianidae	*Francolinus gularis*	T			1	8	0	7	0	8	1	7	0	9	0	8	2	8	2	10	0
Phasianidae	*Peliperdix coqui*				1	24	3	16	6	25	2	26	2	25	4	19	10	27	5	27	4
Phasianidae	*Peliperdix albogularis*				1	18	3	12	3	23	2	31	2	27	3	16	6	33	4	33	3
Phasianidae	*Peliperdix schlegelii*				1	19	2	8	4	22	4	33	1	30	3	12	9	34	8	36	4
Phasianidae	*Peliperdix lathami*				1	28	6	15	8	29	8	47	5	42	8	23	14	48	11	52	7
Phasianidae	*Peliperdix sephaena*				1	36	3	18	15	40	1	40	1	38	4	21	20	41	3	41	3
Phasianidae	*Scleroptila streptophorus*				1	29	3	17	6	32	2	33	1	31	3	21	10	36	2	37	1
Phasianidae	*Scleroptila finschi*				1	70	0	52	1	70	0	70	0	70	0	54	4	70	0	70	0
Phasianidae	*Scleroptila africanus*				1	4	5	2	6	4	7	3	6	4	7	2	9	4	10	4	10
Phasianidae	*Scleroptila levaillantii*				1	17	3	11	6	17	3	18	2	17	4	12	8	18	3	18	4
Phasianidae	*Scleroptila psilolaemus*				1	59	1	18	28	65	0	65	1	64	1	26	33	65	2	65	2
Phasianidae	*Scleroptila shelleyi*				1	39	1	33	3	39	1	39	1	39	2	33	8	39	1	39	1
Phasianidae	*Scleroptila levaillantoides*				1	10	3	5	6	10	3	10	3	10	5	6	10	10	7	10	6
Phasianidae	*Pternistis nahani*	T			1	44	6	30	10	45	6	59	7	63	6	34	10	59	6	67	7
Phasianidae	*Pternistis hartlaubi*				1	20	3	13	7	20	3	19	5	20	7	14	13	20	3	19	5
Phasianidae	*Pternistis bicalcaratus*				1	12	3	7	3	14	3	20	2	18	3	9	6	21	6	21	4
Phasianidae	*Pternistis clappertoni*				1	10	1	5	2	12	1	13	0	12	1	7	4	13	2	13	2
Phasianidae	*Pternistis icterorhynchus*				1	11	2	5	3	12	1	15	1	14	2	7	3	15	2	15	1
Phasianidae	*Pternistis harwoodi*	T		x	1	86	0x	86	0x	86	0x	86	0x	86	0x	86	0x	86	0x	86	0x
Phasianidae	*Pternistis adspersus*				1	9	3	5	4	9	3	9	3	9	5	5	9	9	4	9	4
Phasianidae	*Pternistis capensis*				1	3	8	1	10	3	11	2	10	3	11	1	14	3	17	3	16
Phasianidae	*Pternistis hildebrandti*				1	39	1	32	3	39	1	41	1	41	2	35	5	42	1	42	1
Phasianidae	*Pternistis natalensis*				1	18	2	16	3	18	3	18	3	18	5	17	9	18	5	18	5
Phasianidae	*Pternistis ahantensis*		H		1	0	6	0	8	0	10	0	4	0	7	0	12	0	11	0	6
Phasianidae	*Pternistis squamatus*		H		1	0	6	0	11	0	6	0	4	0	7	0	14	0	7	0	5
Phasianidae	*Pternistis griseostriatus*	T			1	71	0	52	0	71	0	71	0	71	0	52	0	71	0	71	0
Phasianidae	*Pternistis leucoscepus*		H		1	0	4	0	22	0	1	0	1	0	4	0	26	0	2	0	2
Phasianidae	*Pternistis rufopictus*				1	43	10	41	20	55	8	53	10	48	10	52	20	74	8	67	15
Phasianidae	*Pternistis afer*				1	31	2	24	3	31	2	33	2	32	3	26	8	33	3	33	3

Family	Species	IUCN	Ag	R	Range Source	Percentage loss in geographic range 2050 TG H	TG C	AM H	AM C	GO H	GO C	OS H	OS C	2100 TG H	TG C	AM H	AM C	GO H	GO C	OS H	OS C
Phasianidae	*Pternistis swainsonii*				1	12	1	10	2	12	1	12	1	12	3	10	8	13	3	12	2
Phasianidae	*Pternistis erckelii*				1	17	4	9	2	17	1	17	1	17	4	11	2	17	2	17	2
Phasianidae	*Pternistis ochropectus*	T			1	0	0	0	0	0	0	0	0	0	0	0	15	0	22	0	15
Phasianidae	*Pternistis castaneicollis*				1	55	3	21	17	61	0	61	1	58	3	29	23	61	1	61	1
Phasianidae	*Pternistis nobilis*				1	28	3	31	5	35	5	63	5	56	3	36	5	61	6	66	5
Phasianidae	*Pternistis jacksoni*			x	1	32	0x	28	2x	32	0x	32	0x	32	0x	30	2x	32	0x	32	0x
Phasianidae	*Pternistis camerunensis*	T			1	53	0	29	0	43	0	71	0	64	0	29	0	58	0	74	0
Phasianidae	*Pternistis swierstrai*	T			1	61	0	53	0	61	0	61	0	61	0	55	0	61	0	61	0
Phasianidae	*Perdix perdix*		H		1	0	21	0	19	0	23	0	17	0	25	0	21	0	26	0	19
Phasianidae	*Perdix dauurica*		H		1	0	6	0	7	0	7	0	7	0	8	0	11	0	13	0	12
Phasianidae	*Perdix hodgsoniae*		H		1	0	18	0	17	0	18	0	17	0	32	0	42	0	41	0	37
Phasianidae	*Rhizothera longirostris*				1	15	3	18	3	16	3	21	3	22	4	25	4	30	4	31	3
Phasianidae	*Margaroperdix madagaren*		H		1	0	0	0	4	0	0	0	0	0	0	0	4	0	0	0	0
Phasianidae	*Melanoperdix nigra*	T			1	17	3	20	3	19	3	23	3	25	4	27	4	33	4	34	3
Phasianidae	*Coturnix coturnix*		H		1	0	11	0	10	0	12	0	9	0	13	0	13	0	15	0	11
Phasianidae	*Coturnix japonica*		H		1	0	4	0	4	0	4	0	5	0	5	0	6	0	6	0	6
Phasianidae	*Coturnix pectoralis*		H		1	0	12	0	8	0	10	0	6	0	15	0	10	0	14	0	7
Phasianidae	*Coturnix coromandelica*		H		1	0	6	0	5	0	5	0	4	0	9	0	12	0	14	0	8
Phasianidae	*Coturnix delegorguei*				1	27	2	15	8	29	1	29	1	29	3	18	11	30	3	30	2
Phasianidae	*Coturnix ypsilophora*		H		1	0	15	0	10	0	13	0	6	0	19	0	14	0	18	0	8
Phasianidae	*Coturnix adansonii*				1	34	3	21	7	35	4	42	2	40	4	25	10	42	5	44	3
Phasianidae	*Coturnix chinensis*		H		1	0	8	0	5	0	7	0	4	0	11	0	10	0	12	0	6
Phasianidae	*Anurophasis monorthonyx*			x	1	0	1x	27	0x	1	0x	15	1x	1	1x	29	1x	1	0x	15	1x
Phasianidae	*Perdicula asiatica*				1	15	7	13	7	14	6	18	3	15	9	15	11	16	11	24	6
Phasianidae	*Perdicula argoondah*				1	13	7	10	9	10	8	15	6	13	10	11	16	11	18	19	9
Phasianidae	*Perdicula erythrorhyncha*				1	19	3	20	3	20	2	27	1	21	4	22	5	22	5	41	1
Phasianidae	*Perdicula manipurensis*	T			1	38	8	40	8	40	8	42	8	42	11	41	12	42	12	48	10
Phasianidae	*Xenoperdix udzungwensis*	T			1	29	0	20	3	29	0	29	0	29	0	26	3	29	0	29	0
Phasianidae	*Arborophila torqueola*				1	42	8	45	7	38	12	43	8	46	11	47	11	43	14	46	11
Phasianidae	*Arborophila rufogularis*				1	27	16	31	14	25	18	29	18	31	19	34	18	31	22	37	20
Phasianidae	*Arborophila atrogularis*				1	30	11	31	11	28	14	27	13	35	14	34	14	32	16	34	15
Phasianidae	*Arborophila crudigularis*				1	0	0	0	0	0	0	0	0	0	0	0	0	0	0	0	0
Phasianidae	*Arborophila mandellii*	T			1	65	7	70	3	64	7	67	5	66	9	73	5	66	8	71	7
Phasianidae	*Arborophila brunneopectus*				1	13	14	18	12	16	13	18	11	15	14	18	14	21	16	25	15
Phasianidae	*Arborophila rufipectus*	T			1	0	0	0	0	0	0	0	0	0	0	0	0	0	0	0	0
Phasianidae	*Arborophila orientalis*	T			1	22	4	25	4	27	2	27	4	26	4	31	4	44	2	36	4
Phasianidae	*Arborophila javanica*			x	1	0	0x	0	0x	0	0x	0	0x	1	0x	0	0x	0	0x	0	0x
Phasianidae	*Arborophila hyperythra*				1	19	0	21	2	16	5	16	2	29	0	24	2	37	5	36	2
Phasianidae	*Arborophila gingica*	T			1	5	0	5	0	5	0	5	0	5	0	5	0	5	0	5	0
Phasianidae	*Arborophila davidi*	T		x	1	0	0x	0	0x	0	0x	0	0x	0	0x	0	0x	0	0x	0	0x
Phasianidae	*Arborophila cambodiana*	T		x	1	14	0x	14	0x	14	0x	22	0x	14	0x	14	10x	22	5x	23	3x
Phasianidae	*Arborophila rubrirostris*				1	22	7	21	7	26	5	26	6	26	7	26	8	42	5	35	6
Phasianidae	*Arborophila ardens*	T			1	0	0	0	0	0	0	0	0	0	0	0	0	0	0	0	0
Phasianidae	*Arborophila chloropus*				1	10	12	13	11	12	12	17	11	12	16	14	21	16	25	24	20

Family	Species	IUCN	Ag	R	Range Source	Percentage loss in geographic range 2050 TG H	2050 TG C	2050 AM H	2050 AM C	2050 GO H	2050 GO C	2050 OS H	2050 OS C	2100 TG H	2100 TG C	2100 AM H	2100 AM C	2100 GO H	2100 GO C	2100 OS H	2100 OS C
Phasianidae	*Arborophila merlini*			x	1	0	12x	0	12x	9	12x	2	10x	0	12x	0	12x	24	12x	2	10x
Phasianidae	*Arborophila charltonii*		H		1	0	8	0	7	0	7	0	8	0	9	0	8	0	7	0	8
Phasianidae	*Caloperdix oculea*				1	17	6	22	7	19	6	25	6	23	7	29	8	35	8	34	8
Phasianidae	*Haematortyx sanguiniceps*				1	15	0	18	2	10	4	12	2	23	0	22	2	25	4	28	2
Phasianidae	*Rollulus rouloul*				1	13	2	16	3	15	3	19	2	20	3	22	3	28	3	28	2
Phasianidae	*Ptilopachus petrosus*				1	15	2	7	5	17	2	20	1	19	2	10	8	21	4	21	3
Phasianidae	*Bambusicola fytchii*				1	21	10	23	10	19	12	20	12	23	12	26	13	22	15	27	13
Phasianidae	*Bambusicola thoracica*				1	4	0	4	0	4	0	4	0	4	0	4	0	4	0	4	0
Phasianidae	*Galloperdix spadicea*				1	13	2	15	2	14	2	19	1	14	3	16	5	15	6	26	1
Phasianidae	*Galloperdix lunulata*				1	13	2	13	2	15	1	19	0	15	3	15	2	18	2	27	0
Phasianidae	*Galloperdix bicalcarata*				1	48	0x	53	0x	39	3x	51	0x	48	0x	56	0x	48	3x	58	0x
Phasianidae	*Ithaginis cruentus*				1	22	1	23	1	23	1	23	1	24	2	25	2	25	2	26	2
Phasianidae	*Tragopan melanocephalus*	T		x	1	0	0x	0	0x	0	0x	0	0x	0	0x	0	0x	0	0x	0	0x
Phasianidae	*Tragopan satyra*			x	1	67	1x	69	0x	68	0x	75	0x	67	1x	75	0x	68	0x	75	0x
Phasianidae	*Tragopan blythii*	T			1	30	5	30	6	28	9	27	8	38	7	35	8	30	9	30	8
Phasianidae	*Tragopan temminckii*				1	8	0	8	0	8	0	8	0	8	0	9	0	9	0	9	0
Phasianidae	*Tragopan caboti*	T			1	9	0	9	0	9	0	9	0	9	0	9	0	9	0	9	0
Phasianidae	*Pucrasia macrolopha*				1	13	1	13	0	13	0	13	0	13	1	14	3	13	3	13	1
Phasianidae	*Lophophorus impejanus*				1	67	5	68	2	68	2	72	1	69	7	74	5	74	4	76	3
Phasianidae	*Lophophorus sclateri*	T			1	15	2	16	2	18	2	17	0	21	4	18	4	23	3	21	2
Phasianidae	*Lophophorus lhuysii*	T			1	38	1	39	3	37	4	40	2	38	8	42	9	42	5	43	3
Phasianidae	*Gallus gallus*				1	21	8	24	8	21	9	26	8	25	11	28	11	29	13	35	11
Phasianidae	*Gallus sonneratii*				1	14	4	17	4	17	2	22	1	15	6	19	6	19	6	32	1
Phasianidae	*Gallus lafayetii*				1	52	0	55	0	40	3	54	0	53	0	60	0	52	3	63	0
Phasianidae	*Gallus varius*				1	9	2	4	4	1	4	3	2	10	9	7	10	13	5	11	3
Phasianidae	*Lophura leucomelanos*		H		1	0	6	0	6	0	7	0	6	0	7	0	9	0	10	0	8
Phasianidae	*Lophura nycthemera*				1	7	6	11	4	8	6	12	5	7	11	12	8	12	14	16	9
Phasianidae	*Lophura imperialis*			x	1	0	56x	0	56x	21	56x	50	16x	0	56x	0	56x	40	56x	50	16x
Phasianidae	*Lophura edwardsi*	T		x	1	0	25x	0	25x	29	25x	20	7x	0	25x	0	25x	56	25x	20	7x
Phasianidae	*Lophura swinhoii*			x	1	0	0x	0	0x	0	0x	0	0x	0	0x	0	0x	0	0x	0	0x
Phasianidae	*Lophura inornata*	T		x	1	48	0x	19	11x	31	0x	32	0x	50	0x	20	11x	56	0x	53	0x
Phasianidae	*Lophura erythrophthalma*	T			1	20	4	27	4	23	4	27	5	28	6	36	5	42	5	39	5
Phasianidae	*Lophura ignita*				1	13	2	16	3	15	3	18	2	20	3	23	3	28	3	28	2
Phasianidae	*Lophura diardi*				1	11	14	17	13	13	16	15	16	13	20	17	23	18	30	23	24
Phasianidae	*Lophura bulweri*	T			1	9	0	12	1	7	2	10	1	15	0	14	1	17	2	20	1
Phasianidae	*Crossoptilon crossoptilon*				1	23	1	24	2	24	1	24	1	24	2	26	4	26	2	25	3
Phasianidae	*Crossoptilon mantchuricum*	T			1	0	0	0	0	0	0	0	0	0	0	0	0	0	0	0	0
Phasianidae	*Crossoptilon auritum*				1	11	2	11	3	11	3	11	3	11	2	11	4	12	4	12	3
Phasianidae	*Catreus wallichii*	T			1	39	4	38	4	39	1	45	0	41	8	42	11	44	17	46	4
Phasianidae	*Syrmaticus ellioti*	T			1	17	0	17	0	17	0	17	0	17	0	17	0	17	0	17	0
Phasianidae	*Syrmaticus humiae*	T			1	29	10	33	8	27	12	25	13	30	12	35	9	30	14	29	14
Phasianidae	*Syrmaticus mikado*			x	1	0	0x	0	0x	0	0x	0	0x	0	0x	0	0x	0	0x	0	0x
Phasianidae	*Syrmaticus soemmerringii*				1	7	18	8	15	9	17	7	17	8	28	10	34	13	32	10	32
Phasianidae	*Syrmaticus reevesii*	T			1	2	0	2	0	2	0	2	0	2	0	2	0	2	0	2	0

Family	Species	IUCN	Ag	R	Range Source	Percentage loss in geographic range 2050 TG H	TG C	AM H	AM C	GO H	GO C	OS H	OS C	2100 TG H	TG C	AM H	AM C	GO H	GO C	OS H	OS C
Phasianidae	*Phasianus colchicus*		H		1	0	12	0	10	0	11	0	8	0	15	0	13	0	15	0	11
Phasianidae	*Phasianus versicolor*				14	8	20	9	15	9	19	8	17	10	28	12	32	14	32	11	30
Phasianidae	*Chrysolophus pictus*				1	2	0	2	0	2	0	2	0	2	0	2	0	2	0	2	0
Phasianidae	*Chrysolophus amherstiae*				1	16	1	16	0	16	0	16	1	16	1	18	1	17	1	16	1
Phasianidae	*Polyplectron chalcurum*				1	24	5	23	6	27	4	28	5	27	5	30	6	47	4	36	5
Phasianidae	*Polyplectron inopinatum*	T			1	5	10x	23	10x	16	10x	45	10x	13	11x	24	10x	43	10x	52	10x
Phasianidae	*Polyplectron germaini*	T			1	7	14	11	8	3	14	5	14	7	15	11	18	11	24	14	23
Phasianidae	*Polyplectron bicalcaratum*				1	28	8	32	8	28	10	31	9	33	11	34	11	33	13	37	12
Phasianidae	*Polyplectron malacense*	T			1	12	7	15	6	14	7	24	7	19	7	20	7	31	7	30	7
Phasianidae	*Polyplectron schleiermache*	T			1	3	0	6	0	10	0	7	0	8	0	10	0	15	0	12	0
Phasianidae	*Polyplectron emphanum*	T		x	1	21	18x	19	22x	24	9x	17	20x	21	18x	19	44x	47	31x	29	50x
Phasianidae	*Rheinardia ocellata*	T			1	7	17	15	17	25	18	34	13	19	20	17	18	41	20x	34	14
Phasianidae	*Argusianus argus*				1	13	3	16	3	15	3	19	3	20	3	22	4	28	4	28	3
Phasianidae	*Afropavo congensis*	T			1	36	3	27	4	37	1	61	1	54	3	41	4	76	2	75	1
Phasianidae	*Pavo cristatus*				1	29	3	34	3	32	3	39	2	32	4	38	4	37	5	57	2
Phasianidae	*Pavo muticus*	T			1	17	7	19	7	15	9	18	8	20	9	22	11	19	13	24	10
Phasianidae	*Falcipennis falcipennis*				1	1	9	0	10	0	10	0	10	2	12	1	13	2	13	5	12
Phasianidae	*Falcipennis canadensis*				1	4	19	6	17	5	18	6	17	5	24	8	25	9	26	10	24
Phasianidae	*Dendragapus obscurus*				1	5	23	9	19	7	21	11	17	6	30	13	31	15	35	20	26
Phasianidae	*Lagopus lagopus*				1	1	20	1	20	1	21	1	19	2	27	2	31	2	34	3	29
Phasianidae	*Lagopus mutus*				1	0	16	0	15	0	15	0	15	0	24	0	31	1	33	1	29
Phasianidae	*Lagopus leucurus*				1	0	36	1	35	1	36	1	35	1	48	2	58	2	61	2	57
Phasianidae	*Tetrao tetrix*				1	6	14	5	13	7	15	6	12	7	16	7	15	10	18	10	14
Phasianidae	*Tetrao mlokosiewiczi*			x	1	12	36x	9	39x	15	34x	13	40x	18	43x	19	44x	26	42x	24	40x
Phasianidae	*Tetrao urogallus*				1	3	16	3	15	4	16	4	14	4	19	5	19	7	20	7	18
Phasianidae	*Tetrao parvirostris*				1	4	11	4	11	4	11	4	11	4	13	4	18	4	19	5	18
Phasianidae	*Bonasa bonasia*				1	5	13	5	12	5	13	5	12	6	15	6	15	8	16	9	15
Phasianidae	*Bonasa sewerzowi*				1	16	0	16	0	16	1	15	2	16	1	18	3	17	2	16	3
Phasianidae	*Bonasa umbellus*				1	9	23	14	20	13	21	15	19	13	28	18	27	22	28	21	25
Phasianidae	*Centrocercus urophasianus*				1	0	14	0	11	0	13	0	10	0	15	0	13	1	15	2	11
Phasianidae	*Tympanuchus phasianellus*				1	2	18	3	15	3	17	3	13	2	22	4	21	5	24	5	19
Phasianidae	*Tympanuchus cupido*	T			1	7	10	8	6	8	7	12	2	10	12	12	9	15	10	16	3
Phasianidae	*Tympanuchus pallidicinctus*	T			1	0	8	1	5	1	8	1	5	0	11	1	14	1	19	1	15
Phasianidae	*Meleagris gallopavo*				1	6	19	11	12	9	15	12	9	10	23	15	18	19	22	18	12
Phasianidae	*Meleagris ocellata*				1	5	4	15	0	16	4	26	4	7	4	21	13	19	17	32	7
Numididae	*Agelastes meleagrides*	T			1	26	5	17	2	20	6	38	1	30	7	18	8	32	8	38	1
Numididae	*Agelastes niger*				1	30	7	16	10	24	8	55	3	46	7	28	11	53	9	64	4
Numididae	*Numida meleagris*				1	28	3	18	5	29	3	34	2	33	4	21	9	35	5	35	3
Numididae	*Guttera plumifera*				1	32	5	19	8	25	7	51	4	48	5	30	9	53	8	62	5
Numididae	*Guttera pucherani*				1	36	5	24	10	37	5	47	3	44	6	28	13	48	6	50	3
Numididae	*Acryllium vulturinum*				1	44	5	9	32	52	1	52	1	48	5	13	36	52	2	52	1
Odontophoridae	*Dendrortyx barbatus*	T			1	10	0	8	0	7	0	17	0	10	0	18	0	7	0	17	0
Odontophoridae	*Dendrortyx macroura*				1	5	6	23	1	10	6	27	0	8	6	24	5	16	6	28	0
Odontophoridae	*Dendrortyx leucophrys*				1	21	2	28	2	25	3	40	2	23	2	33	6	32	5	41	2

Family	Species	IUCN	Ag	R	Range Source	2050 TG H	2050 TG C	2050 AM H	2050 AM C	2050 GO H	2050 GO C	2050 OS H	2050 OS C	2100 TG H	2100 TG C	2100 AM H	2100 AM C	2100 GO H	2100 GO C	2100 OS H	2100 OS C
Odontophoridae	*Oreortyx pictus*				1	5	35	8	26	8	31	11	20	6	39	11	34	14	40	16	26
Odontophoridae	*Callipepla squamata*				1	1	21	3	17	2	19	4	16	1	24	3	33	2	35	4	29
Odontophoridae	*Callipepla douglasii*				1	16	15	27	9	15	13	40	7	23	18	27	21	18	23	43	14
Odontophoridae	*Callipepla californica*				1	3	23	5	17	5	21	6	14	3	26	7	24	8	30	10	18
Odontophoridae	*Callipepla gambelii*				1	0	19	1	21	0	23	2	22	1	23	1	36	0	44	2	39
Odontophoridae	*Philortyx fasciatus*		H		1	0	4	0	2	0	4	0	0	0	4	0	13	0	4	0	0
Odontophoridae	*Colinus virginianus*		H		1	0	11	0	5	0	9	0	4	0	14	0	12	0	14	0	5
Odontophoridae	*Colinus nigrogularis*		H		1	0	6	0	4	0	6	0	3	0	10	0	15	0	11	0	6
Odontophoridae	*Colinus cristatus*		H		1	0	7	0	7	0	6	0	5	0	9	0	10	0	11	0	8
Odontophoridae	*Colinus leucopogon*				14	20	0	30	2	31	5	43	0	24	0	32	5	33	7	48	0
Odontophoridae	*Odontophorus gujanensis*				1	7	12	5	12	5	12	10	12	12	13	9	15	19	17	20	15
Odontophoridae	*Odontophorus capueira*				1	13	11	11	8	19	8	21	6	18	16	19	12	31	11	35	10
Odontophoridae	*Odontophorus melanotis*				1	21	7	35	9	26	12	43	9	28	7	41	11	39	13	53	9
Odontophoridae	*Odontophorus erythrops*				1	15	4	17	6	18	3	19	4	22	7	22	6	25	6	27	5
Odontophoridae	*Odontophorus atrifrons*	T			1	22	1	17	5	18	5	20	1	29	1	23	6	32	11	28	1
Odontophoridae	*Odontophorus hyperythrus*				1	15	5	4	5	9	2	25	5	22	5	7	5	25	2	30	5
Odontophoridae	*Odontophorus melanonotu*	T		x	1	20	0x	18	0x	28	0x	51	0x	24	10x	59	0x	80	0x	55	0x
Odontophoridae	*Odontophorus speciosus*				1	5	8	3	8	3	8	6	8	9	9	9	11	9	11	15	10
Odontophoridae	*Odontophorus dialeucos*	T		x	1	17	0x	19	0x	14	0x	17	0x	31	9x	21	0x	31	9x	35	9x
Odontophoridae	*Odontophorus strophium*	T		x	1	16	0x	39	0x	12	0x	39	0x	33	0x	45	0x	50	12x	44	11x
Odontophoridae	*Odontophorus columbianus*				1	38	0	18	0	41	0	44	0	45	6x	41	12x	53	17x	52	5x
Odontophoridae	*Odontophorus leucolaemus*				1	35	6	44	14	23	16	42	13	40	6	46	16	35	16	58	13
Odontophoridae	*Odontophorus balliviani*				1	9	21	12	15	7	20	16	21	14	25	18	20	22	24	25	23
Odontophoridae	*Odontophorus stellatus*				1	1	3	1	3	1	3	2	3	2	5	2	5	3	6	5	5
Odontophoridae	*Odontophorus guttatus*				1	12	4	21	4	15	6	28	5	15	5	25	11	23	11	34	6
Odontophoridae	*Dactylortyx thoracicus*				1	10	3	21	1	16	3	28	2	12	3	26	9	21	7	34	3
Odontophoridae	*Cyrtonyx montezumae*				1	7	16	18	9	8	13	21	8	8	18	18	26	11	27	25	18
Odontophoridae	*Cyrtonyx ocellatus*				1	12	2	21	2	21	3	28	2	15	2	23	2	25	6	29	2
Odontophoridae	*Rhynchortyx cinctus*				1	18	6	28	8	21	9	31	7	26	7	32	9	32	12	41	8
Turnicidae	*Turnix sylvatica*				1	18	3	13	5	19	3	22	3	21	5	15	9	22	7	24	5
Turnicidae	*Turnix maculosa*		H		1	0	21	0	13	0	18	0	9	0	26	0	23	0	28	0	12
Turnicidae	*Turnix hottentotta*				1	32	2	25	4	32	2	34	2	33	3	27	8	33	3	35	2
Turnicidae	*Turnix tanki*				1	10	3	11	3	10	3	12	3	11	5	12	5	12	7	16	4
Turnicidae	*Turnix ocellata*				1	2	12	13	5	3	12	8	10	2	21	13	16	8	20	12	16
Turnicidae	*Turnix suscitator*		H		1	0	4	0	4	0	4	0	4	0	7	0	7	0	9	0	5
Turnicidae	*Turnix nigricollis*				1	49	0	27	5	49	0	49	0	49	0	37	5	49	0	49	0
Turnicidae	*Turnix melanogaster*	T			1	5	10	5	15	22	8	24	9	9	26	5	25	29	22	38	13
Turnicidae	*Turnix castanota*				1	0	18	0	12	0	17	0	6	0	19	0	16	0	22	0	10
Turnicidae	*Turnix olivii*	T		x	1	0	11x	0	9x	0	19x	0	17x	0	29x	0	19x	0	19x	0	17x
Turnicidae	*Turnix varia*				1	2	20	3	11	4	17	5	7	4	26	4	19	7	25	8	10
Turnicidae	*Turnix worcesteri*				1	5	5	9	5	7	5	11	3	5	16	13	18x	15	15x	15	10x
Turnicidae	*Turnix everetti*	T		x	1	0	0x	0	0x	0	0x	0	0x	0	13x	0	14x	0	13x	0	0x
Turnicidae	*Turnix pyrrhothorax*		H		1	0	18	0	13	0	16	0	10	0	20	0	18	0	20	0	12
Turnicidae	*Turnix velox*		H		1	0	12	0	9	0	10	0	7	0	13	0	12	0	14	0	9

Family	Species	IUCN	Ag	R	Range Source	Percentage loss in geographic range 2050 TG H	TG C	AM H	AM C	GO H	GO C	OS H	OS C	2100 TG H	TG C	AM H	AM C	GO H	GO C	OS H	OS C
Turnicidae	*Ortyxelos meiffrenii*				1	15	4	5	11	16	4	20	3	19	5	6	14	20	6	21	4
Indicatoridae	*Indicator maculatus*				1	35	6	20	9	32	7	53	4	50	7	29	11	55	8	60	6
Indicatoridae	*Indicator variegatus*				1	41	1	29	4	42	1	43	1	42	2	32	7	43	1	43	1
Indicatoridae	*Indicator indicator*				1	29	3	19	5	30	3	35	2	34	3	22	8	35	4	36	3
Indicatoridae	*Indicator archipelagicus*				1	19	8	20	7	22	5	28	7	24	10	27	8	46	7	38	7
Indicatoridae	*Indicator minor*				1	29	2	18	5	31	2	33	1	32	3	20	8	34	2	34	2
Indicatoridae	*Indicator conirostris*				1	32	7	20	9	30	9	51	5	47	8	28	13	52	11	58	6
Indicatoridae	*Indicator willcocksi*				1	27	6	16	8	26	9	44	5	39	8	23	14	45	13	49	7
Indicatoridae	*Indicator exilis*				1	37	5	22	8	35	7	55	4	51	6	30	12	55	9	60	5
Indicatoridae	*Indicator pumilio*				1	26	7	26	11	34	8	51	1	45	7	27	13	45	10	52	1
Indicatoridae	*Indicator meliphilus*				1	36	4	25	11	40	2	40	2	38	4	28	13	43	2	42	2
Indicatoridae	*Indicator xanthonotus*				1	58	3	61	2	66	2	67	1	68	5	67	4	71	3	71	2
Indicatoridae	*Melichneutes robustus*				1	34	6	22	8	33	7	55	5	51	7	33	10	59	8	63	5
Indicatoridae	*Melignomon eisentrauti*				1	11	8	6	5	25	3	30	3	21	8	9	7	31	3	31	3
Indicatoridae	*Melignomon zenkeri*				1	38	7	23	10	36	9	59	6	55	7	33	11	62	10	68	6
Indicatoridae	*Prodotiscus insignis*				1	41	8	24	12	38	10	58	5	53	10	32	16	56	11	64	7
Indicatoridae	*Prodotiscus zambesiae*				1	44	0	33	1	44	0	45	0	45	1	35	5	45	0	45	0
Indicatoridae	*Prodotiscus regulus*				1	32	2	25	3	32	2	36	1	35	3	28	7	36	3	37	2
Picidae	*Jynx torquilla*		H		1	0	14	0	13	0	15	0	12	0	17	0	16	0	18	0	15
Picidae	*Jynx ruficollis*		H		1	0	3	0	7	0	4	0	3	0	4	0	10	0	6	0	4
Picidae	*Picumnus innominatus*				1	15	4	17	4	16	4	17	4	17	5	19	5	20	6	22	6
Picidae	*Picumnus aurifrons*				1	5	7	3	7	3	7	9	7	10	8	7	8	18	9	18	8
Picidae	*Picumnus pumilus*				1	0	2	0	2	1	1	2	1	0	4	0	4	2	3	3	3
Picidae	*Picumnus lafresnayi*				1	7	1	4	1	5	1	11	0	13	1	15	1	17	1	26	0
Picidae	*Picumnus exilis*				1	4	14	4	14	5	14	7	13	7	16	6	19	14	19	13	18
Picidae	*Picumnus nigropunctatus*				14	15	5	13	5	9	5	25	5	20	5	19	6	20	6	35	5
Picidae	*Picumnus fuscus*				1	0	91x	0	91x	0	91x	0	91x	0	91x	0	91x	0	91x	0	91x
Picidae	*Picumnus sclateri*				1	0	7	3	3	0	7	1	6	0	7	3	8	1	12	1	11
Picidae	*Picumnus squamulatus*				1	24	5	20	5	22	3	34	2	30	11	32	13	45	17	50	6
Picidae	*Picumnus spilogaster*				1	6	21	1	21	6	21	8	19	9	22	6	24	13	26	15	22
Picidae	*Picumnus minutissimus*				1	1	24	2	22	2	22	3	22	3	24	6	24	7	26	5	24
Picidae	*Picumnus pygmaeus*				1	9	20	6	17	8	18	12	10	11	23	8	20	12	22	18	14
Picidae	*Picumnus steindachneri*	T			1	4	2	18	2	6	2	15	0	17	2	32	2	12	2	49	0
Picidae	*Picumnus varzeae*				1	24	12	14	9	12	10	11	9	36	12	29	9	32	13	36	12
Picidae	*Picumnus dorbygnianus*				1	15	14	18	9	16	9	24	11	22	14	25	13	31	13	33	12
Picidae	*Picumnus temminckii*				1	20	6	17	7	28	4	29	5	28	14	32	15	46	9	49	11
Picidae	*Picumnus albosquamatus*				1	11	25	4	26	6	28	13	24	15	32	10	34	17	34	22	30
Picidae	*Picumnus cirratus*				1	9	9	7	8	9	7	12	7	14	12	12	13	23	12	22	12
Picidae	*Picumnus rufiventris*				1	5	5	4	5	4	5	8	5	10	6	10	7	12	7	18	7
Picidae	*Picumnus fulvescens*				1	15	30	19	26	18	26	33	11	18	39	23	29	25	32	38	18
Picidae	*Picumnus limae*	T			1	0	4	0	4	1	4	1	4	1	4	0	4	15	4	28	4x
Picidae	*Picumnus nebulosus*				1	23	2	19	4	31	2	31	3	35	7	39	7	51	4	57	6
Picidae	*Picumnus castelnau*		H		1	0	1	0	0	0	0	0	0	0	1	0	0	0	0	0	0
Picidae	*Picumnus subtilis*				1	18	2	22	1	22	2	35	2	30	2	40	2	39	2	51	2

Family	Species	IUCN	Ag	R	Range Source	Percentage loss in geographic range 2050 TG H	TG C	AM H	AM C	GO H	GO C	OS H	OS C	2100 TG H	TG C	AM H	AM C	GO H	GO C	OS H	OS C
Picidae	*Picumnus olivaceus*				1	19	6	23	8	20	9	31	6	25	7	29	10	32	11	38	7
Picidae	*Picumnus granadensis*				1	6	5	6	5	6	5	7	5	6	5	6	5	10	5	7	5
Picidae	*Picumnus cinnamomeus*				1	16	1	11	6	13	1	10	0	18	8	14	13	16	22	16	8
Picidae	*Sasia africana*				1	35	6	23	8	33	7	56	4	52	6	34	9	61	8	65	4
Picidae	*Sasia abnormis*				1	13	3	16	3	16	3	18	3	20	4	21	4	27	4	26	3
Picidae	*Sasia ochracea*				1	18	8	21	7	18	8	19	8	20	9	23	9	22	10	24	9
Picidae	*Nesoctites micromegas*			x	1	10	8x	16	3x	11	8x	21	0x	10	8x	16	5x	11	8x	21	0x
Picidae	*Melanerpes candidus*		H		1	0	14	0	11	0	13	0	10	0	18	0	16	0	18	0	14
Picidae	*Melanerpes lewis*		H		1	0	18	0	14	0	16	0	12	0	23	0	25	0	28	0	19
Picidae	*Melanerpes herminieri*			x	1	0	0x	100	0x	0	0x	100	0x	0	0x	100	0x	100	0x	100	0x
Picidae	*Melanerpes portoricensis*			x	1	23	0x	23	0x	23	0x	23	0x	23	0x	23	0x	23	0x	23	0x
Picidae	*Melanerpes erythrocephalu*		H		1	0	20	0	9	0	15	0	6	0	23	0	12	0	18	0	6
Picidae	*Melanerpes formicivorus*				1	7	14	13	11	8	13	17	9	9	17	14	21	11	24	20	16
Picidae	*Melanerpes pucherani*				1	15	5	21	7	17	7	27	4	20	6	26	9	25	9	34	6
Picidae	*Melanerpes chrysauchen*				1	21	4	20	10	12	12	25	7	25	4	24	13	19	14	31	8
Picidae	*Melanerpes cruentatus*				1	5	10	4	10	4	10	8	9	9	11	8	12	15	13	17	11
Picidae	*Melanerpes flavifrons*				1	17	9	14	6	22	5	23	5	22	17	25	14	39	10	40	12
Picidae	*Melanerpes cactorum*				1	7	14	4	15	5	14	7	14	9	16	6	23	12	23	12	23
Picidae	*Melanerpes striatus*		H		1	0	13	0	11	0	15	0	0	0	16	0	12	0	15	0	0
Picidae	*Melanerpes radiolatus*			x	1	16	0x	16	7x	11	7x	43	7x	43	0x	16	7x	11	7x	43	7x
Picidae	*Melanerpes chrysogenys*				1	13	7	36	3	19	7	37	3	15	10	36	15	24	12	40	3
Picidae	*Melanerpes hypopolius*				1	7	2	23	0	9	2	19	0	7	2	23	5	9	2	19	0
Picidae	*Melanerpes pygmaeus*				1	4	4	21	0	14	4	30	2	7	5	26	13	17	11	35	6
Picidae	*Melanerpes rubricapillus*				1	19	6	14	8	15	7	21	5	23	8	20	10	28	12	31	8
Picidae	*Melanerpes uropygialis*				1	4	8	9	5	3	8	14	6	5	8	9	13	5	16	15	11
Picidae	*Melanerpes carolinus*				1	10	19	15	8	14	14	13	5	16	22	21	11	28	17	20	6
Picidae	*Melanerpes superciliaris*				1	11	14x	29	15x	17	10x	41	7x	21	14x	32	17x	21	11x	43	7x
Picidae	*Melanerpes aurifrons*				1	5	12	10	9	7	10	12	8	6	15	11	22	9	19	13	13
Picidae	*Melanerpes hoffmannii*		H		1	0	0	0	0	0	0	0	0	0	0	0	9	0	8	0	0
Picidae	*Sphyrapicus varius*				1	21	31	31	18	31	25	29	14	28	33	43	22	46	27	44	16
Picidae	*Sphyrapicus nuchalis*				1	2	15	5	12	3	14	5	12	3	20	6	21	7	24	10	17
Picidae	*Sphyrapicus ruber*				1	7	29	12	21	11	25	15	17	9	35	16	34	17	39	22	26
Picidae	*Sphyrapicus thyroideus*				1	4	21	12	13	9	18	12	12	6	28	15	27	17	32	23	19
Picidae	*Xiphidiopicus percussus*		H		1	0	6	0	5	0	5	0	3	0	6	0	7	0	5	0	3
Picidae	*Campethera punctuligera*				1	10	3	6	3	11	3	16	1	15	3	8	6	17	6	17	4
Picidae	*Campethera scriptoricauda*				14	43	1	37	1	44	0	44	0	43	1	43	2	45	0	45	0
Picidae	*Campethera nubica*				1	31	3	13	15	35	1	36	1	33	3	16	17	36	2	36	2
Picidae	*Campethera bennettii*				1	32	2	22	3	31	2	33	2	33	4	23	10	33	5	34	4
Picidae	*Campethera abingoni*				1	41	1	29	3	41	1	44	1	43	2	32	7	44	3	45	2
Picidae	*Campethera mombassica*				1	3	0	3	0	3	0	3	0	3	0	3	0	3	0	3	0
Picidae	*Campethera notata*				1	0	4	0	4	0	4	0	4	0	5	0	5	0	5	0	5
Picidae	*Campethera maculosa*				1	25	3	14	4	22	4	38	2	31	4	20	7	36	5	38	2
Picidae	*Campethera cailliautii*				1	40	4	29	6	40	5	53	3	50	5	35	9	54	6	57	3
Picidae	*Campethera tullbergi*				1	32	7	25	7	34	6	39	4	36	9	31	10	40	7	41	5

Family	Species	IUCN	Ag	R	Range Source	Percentage loss in geographic range 2050 TG		AM		GO		OS		2100 TG		AM		GO		OS	
						H	C	H	C	H	C	H	C	H	C	H	C	H	C	H	C
Picidae	*Campethera nivosa*				1	36	6	24	8	34	7	53	4	49	7	32	10	55	8	59	4
Picidae	*Campethera caroli*				1	37	6	23	9	35	7	56	4	52	7	32	11	58	8	62	5
Picidae	*Geocolaptes olivaceus*				1	4	4	4	4	3	6	4	4	4	4	4	5	3	7	4	5
Picidae	*Dendropicos elachus*				1	9	5	3	5	8	4	15	3	14	5	5	10	16	10	16	7
Picidae	*Dendropicos poecilolaemu*		H		1	0	6	0	13	0	6	0	2	0	6	0	15	0	7	0	3
Picidae	*Dendropicos abyssinicus*				1	39	0	22	6	41	0	41	0	41	0	27	8	41	0	41	0
Picidae	*Dendropicos fuscescens*				1	32	3	20	7	34	3	38	3	36	4	24	11	38	5	39	4
Picidae	*Dendropicos gabonensis*				1	32	6	23	7	33	7	55	4	49	8	34	9	59	9	62	5
Picidae	*Dendropicos lugubris*				14	27	8	11	12	24	10	39	5	32	9	18	16	34	11	41	7
Picidae	*Dendropicos stierlingi*				1	50	0	43	0	50	0	50	0	50	2	46	3	50	0	50	0
Picidae	*Dendropicos namaquus*		H		1	0	2	0	6	0	2	0	2	0	4	0	10	0	4	0	4
Picidae	*Dendropicos pyrrhogaster*				1	26	9	14	11	29	10	42	8	36	11	24	19	43	13	46	13
Picidae	*Dendropicos xantholophus*				1	39	6	26	8	37	6	58	4	54	6	36	10	62	7	66	4
Picidae	*Dendropicos elliotii*				1	35	6	22	6	32	6	51	4	49	7	31	10	53	8	57	5
Picidae	*Dendropicos goertae*				1	20	4	10	6	22	4	30	3	27	5	14	9	31	7	32	5
Picidae	*Dendropicos spodocephalu*				1	35	4	28	8	39	0	36	3	36	4	29	9	39	0	36	3
Picidae	*Dendropicos griseocephalu*				1	45	1	33	2	46	1	49	0	48	2	34	6	48	1	49	1
Picidae	*Dendropicos obsoletus*				1	16	2	9	4	19	2	22	1	21	2	12	7	24	4	23	3
Picidae	*Dendrocopos temminckii*				1	14	14	25	10	18	13	17	12	16	15	28	21	27	27	24	25
Picidae	*Dendrocopos maculatus*				1	15	11	24	11	13	12	22	12	21	18	30	14	25	21	34	13
Picidae	*Dendrocopos moluccensis*				1	14	2	15	2	16	2	19	1	17	3	19	4	21	4	29	1
Picidae	*Dendrocopos canicapillus*		H		1	0	4	0	5	0	5	0	5	0	7	0	7	0	8	0	7
Picidae	*Dendrocopos kizuki*				1	14	11	12	12	13	13	12	13	15	16	14	20	16	19	18	18
Picidae	*Dendrocopos minor*				1	6	14	5	13	7	15	6	12	8	17	7	16	11	19	11	15
Picidae	*Dendrocopos auriceps*				1	31	4	36	1	36	0	36	0	34	4	38	2	38	3	42	1
Picidae	*Dendrocopos macei*				1	21	5	23	5	20	7	22	6	24	8	26	10	24	12	28	10
Picidae	*Dendrocopos atratus*				1	20	10	27	8	20	11	20	11	22	13	30	11	26	14	27	13
Picidae	*Dendrocopos mahrattensis*				1	14	3	16	2	14	2	19	1	15	4	17	7	16	9	28	3
Picidae	*Dendrocopos dorae*	T			1	0	0	0	0	0	0	0	0	0	0	0	0	0	6	0	6
Picidae	*Dendrocopos hyperythrus*				1	39	11	42	10	39	12	40	11	43	15	45	16	44	17	45	16
Picidae	*Dendrocopos cathpharius*				1	29	10	31	9	29	11	29	10	32	11	32	11	32	12	34	11
Picidae	*Dendrocopos darjellensis*				1	39	3	41	3	40	3	43	0	44	6	43	6	45	6	49	3
Picidae	*Dendrocopos medius*				1	11	21	10	18	10	22	12	16	15	25	15	23	22	26	20	19
Picidae	*Dendrocopos leucotos*				1	6	19	4	19	7	22	5	18	9	22	8	21	12	24	15	21
Picidae	*Dendrocopos major*		H		1	0	13	0	12	0	14	0	11	0	16	0	16	0	18	0	15
Picidae	*Dendrocopos syriacus*		H		1	0	8	0	7	0	8	0	6	0	10	0	8	0	10	0	7
Picidae	*Dendrocopos leucopterus*		H		1	0	6	0	6	0	7	0	7	0	7	0	12	0	16	0	14
Picidae	*Dendrocopos assimilis*				1	6	3	7	4	6	1	7	1	6	4	7	4	6	2	12	2
Picidae	*Dendrocopos himalayensis*				1	63	3	63	3	62	1	68	2	66	5	68	4	71	2	70	2
Picidae	*Picoides lignarius*				1	13	17	12	14	9	16	13	13	15	22	16	24	20	25	24	21
Picidae	*Picoides mixtus*				1	4	12	3	7	4	10	7	7	6	17	6	11	8	13	11	10
Picidae	*Picoides nuttallii*				1	1	34	5	25	1	35	5	20	1	39	5	34	2	44	9	27
Picidae	*Picoides scalaris*				1	4	15	9	13	5	14	11	12	5	18	9	28	7	27	12	21
Picidae	*Picoides pubescens*				1	7	19	12	13	11	16	11	11	11	23	16	18	20	22	18	14

Family	Species	IUCN	Ag	R	Range Source	Percentage loss in geographic range															
						2050								2100							
						TG		AM		GO		OS		TG		AM		GO		OS	
						H	C	H	C	H	C	H	C	H	C	H	C	H	C	H	C
Picidae	*Picoides borealis*	T			1	13	28	26	14	20	19	23	8	24	34	34	19	44	25	35	9
Picidae	*Picoides stricklandi*				1	15	12	31	8	15	11	44	7	21	14	32	16	20	22	50	14
Picidae	*Picoides villosus*				1	7	19	11	14	10	17	11	12	10	23	14	20	17	23	16	17
Picidae	*Picoides albolarvatus*				1	4	25	14	17	9	20	16	14	7	31	17	27	16	33	22	21
Picidae	*Picoides tridactylus*				1	5	13	4	13	5	14	5	13	5	16	5	18	7	18	7	17
Picidae	*Picoides dorsalis*				1	1	16	3	15	2	16	3	15	2	22	4	24	5	25	6	23
Picidae	*Picoides arcticus*				1	2	17	4	15	4	17	5	15	3	22	6	22	6	24	8	21
Picidae	*Veniliornis callonotus*				1	4	12	1	12	4	14	5	12	8	14	6	17	8	19	6	17
Picidae	*Veniliornis dignus*				1	15	8	13	7	14	7	25	9	21	8	20	12	33	9	36	10
Picidae	*Veniliornis nigriceps*				1	18	8	13	7	14	7	25	7	23	9	23	11	32	9	34	8
Picidae	*Veniliornis fumigatus*				1	13	5	16	5	14	5	25	4	19	6	24	9	24	8	36	5
Picidae	*Veniliornis passerinus*				1	6	13	4	11	5	13	10	10	10	16	8	14	14	16	18	13
Picidae	*Veniliornis frontalis*				1	16	5	19	2	18	1	21	1	18	5	20	2	20	2	21	1
Picidae	*Veniliornis spilogaster*				1	17	7	15	6	24	4	25	4	24	14	27	11	40	7	42	9
Picidae	*Veniliornis sanguineus*				1	1	23	3	20	3	20	6	20	4	23	10	22	10	22	8	22
Picidae	*Veniliornis kirkii*				1	16	5	14	7	13	7	18	4	18	7	21	11	23	14	26	7
Picidae	*Veniliornis chocoensis*				1	12	2	15	4	13	0	9	4	24	2	15	4	17	4	22	4
Picidae	*Veniliornis cassini*				1	4	16	3	16	4	15	7	15	8	17	6	20	14	21	13	19
Picidae	*Veniliornis affinis*				1	4	10	2	11	3	11	7	10	8	12	6	13	13	13	15	12
Picidae	*Veniliornis maculifrons*				1	11	6	11	6	22	4	14	6	12	10	17	22x	45	13x	30	18x
Picidae	*Piculus simplex*				1	19	5	32	7	28	8	40	6	25	5	40	8	41	9	48	6
Picidae	*Piculus callopterus*				1	15	18	29	25x	12	25	38	18x	15	21	31	25x	36	28x	56	21x
Picidae	*Piculus litae*				1	15	1	11	2	13	0	13	2	23	1	15	2	16	2	21	2
Picidae	*Piculus leucolaemus*				1	3	3	2	3	3	3	6	3	6	4	8	5	7	5	13	4
Picidae	*Piculus flavigula*				1	4	10	3	11	3	11	7	10	8	12	6	13	13	13	14	12
Picidae	*Piculus chrysochloros*				1	5	12	3	11	4	12	8	10	8	14	6	15	13	16	16	13
Picidae	*Piculus aurulentus*				1	16	7	15	6	21	5	24	6	24	14	24	13	39	8	41	11
Picidae	*Piculus auricularis*				1	13	9	29	5	15	9	43	3	17	11	29	17	20	14	45	5
Picidae	*Piculus rubiginosus*				1	9	9	11	9	10	9	16	8	13	11	16	16	18	14	22	11
Picidae	*Piculus rivolii*				1	15	5	14	4	13	5	23	5	20	7	22	10	29	10	29	7
Picidae	*Colaptes atricollis*				1	3	29	8	32	9	32	12	23	5	41	10	47	17	44	21	36
Picidae	*Colaptes punctigula*				1	6	8	4	8	5	8	10	7	11	9	9	9	17	10	19	8
Picidae	*Colaptes melanochloros*				1	8	13	5	11	8	12	12	9	11	17	10	16	16	18	20	14
Picidae	*Colaptes auratus*				1	6	18	10	13	9	16	10	11	9	22	13	19	15	22	15	16
Picidae	*Colaptes chrysoides*				1	0	19	2	22	0	25	4	23	1	24	2	37	1	43	4	40
Picidae	*Colaptes fernandinae*	T		x	1	4	48x	5	48x	50	0x	78	0x	4	48x	5	48x	52	0x	78	0x
Picidae	*Colaptes pitius*				1	7	14	7	12	7	14	7	13	10	22	11	23	16	24	14	24
Picidae	*Colaptes rupicola*		H		1	0	20	0	22	0	21	0	18	0	27	0	31	0	29	0	24
Picidae	*Colaptes campestris*		H		1	0	13	0	9	0	10	0	6	0	18	0	12	0	14	0	9
Picidae	*Celeus brachyurus*		H		1	0	5	0	4	0	5	0	5	0	7	0	7	0	8	0	6
Picidae	*Celeus loricatus*				1	21	6	31	11	25	11	36	9	29	8	36	11	37	14	47	10
Picidae	*Celeus grammicus*				1	4	8	3	8	3	8	7	8	8	9	8	10	11	10	14	9
Picidae	*Celeus undatus*				1	5	14	3	12	5	13	8	12	9	14	6	15	17	16	16	13
Picidae	*Celeus castaneus*				1	13	5	26	4	19	6	33	5	17	6	32	8	27	10	39	6

Family	Species	IUCN	Ag	R	Range Source	Percentage loss in geographic range 2050 TG		AM		GO		OS		2100 TG		AM		GO		OS	
						H	C	H	C	H	C	H	C	H	C	H	C	H	C	H	C
Picidae	Celeus elegans				1	4	11	3	12	3	12	7	11	8	13	6	14	13	15	14	13
Picidae	Celeus lugubris				1	8	11	6	10	5	10	10	10	13	13	11	18	17	21	19	19
Picidae	Celeus flavescens				1	11	14	7	10	12	11	16	7	14	18	13	15	24	16	29	12
Picidae	Celeus flavus				1	6	10	3	10	4	10	9	9	10	11	7	12	16	13	19	11
Picidae	Celeus spectabilis				1	11	2	7	2	8	1	16	1	16	3	20	4	17	2	31	3
Picidae	Celeus torquatus				1	4	9	3	9	3	9	7	9	8	10	6	11	13	12	14	11
Picidae	Dryocopus galeatus	T			1	24	7	20	7	32	6	35	7	37	12	37	13	51	9	55	11
Picidae	Dryocopus pileatus				1	10	23	16	15	15	19	15	12	15	27	21	20	27	25	23	16
Picidae	Dryocopus lineatus				1	7	11	7	10	7	11	13	9	12	13	11	14	17	14	21	12
Picidae	Dryocopus schulzi				1	1	2	0	2	1	2	1	1	1	3	0	4	1	4	1	2
Picidae	Dryocopus javensis				1	16	6	19	6	17	7	18	6	20	8	23	9	25	10	27	8
Picidae	Dryocopus hodgei			x	1	10	0x	10	0x	10	0x	10	0x	10	0x	20	0x	20	0x	20	0x
Picidae	Dryocopus martius				1	6	13	6	13	7	14	6	12	8	16	7	17	10	18	11	16
Picidae	Campephilus pollens				1	11	7	18	6	16	7	26	5	20	8	26	9	32	10	33	7
Picidae	Campephilus haematogast				1	13	4	20	5	20	4	28	3	22	6	32	7	37	6	42	5
Picidae	Campephilus rubricollis				1	4	9	3	9	3	9	7	8	8	10	6	10	13	11	15	10
Picidae	Campephilus robustus				1	14	11	12	8	18	7	20	7	21	16	20	14	30	11	31	13
Picidae	Campephilus guatemalensi				1	11	6	21	5	14	6	30	4	16	8	24	16	20	12	35	7
Picidae	Campephilus melanoleuco				1	7	12	5	10	6	11	11	9	11	15	9	13	16	15	19	12
Picidae	Campephilus gayaquilensis				1	13	8	9	5	14	8	15	1	14	8	12	6	18	9	19	2
Picidae	Campephilus leucopogon				1	7	10	6	7	5	8	8	7	10	11	9	13	11	15	13	13
Picidae	Campephilus magellanicus				1	12	15	10	14	10	15	9	14	14	24	15	26	18	27	18	25
Picidae	Campephilus imperialis	T			1	19	12	46	5	25	9	59	6	28	12	48	11	37	19	69	12
Picidae	Campephilus principalis	T			1	12	27	15	13	16	20	15	11	26	33	22	19	36	28	21	12
Picidae	Picus mineaceus				1	10	1	14	2	14	2	16	2	17	2	19	2	25	3	25	2
Picidae	Picus chlorolophus				1	20	8	24	7	20	8	24	7	23	10	27	11	26	12	33	10
Picidae	Picus puniceus				1	12	2	16	2	16	3	18	2	20	3	21	3	26	3	27	2
Picidae	Picus flavinucha		H		1	0	7	0	6	0	8	0	7	0	9	0	9	0	11	0	10
Picidae	Picus mentalis				1	10	2	14	2	14	2	17	3	16	3	19	2	24	2	24	3
Picidae	Picus viridanus				1	8	6	14	6	10	6	10	6	10	6	15	7	15	7	15	8
Picidae	Picus vittatus				1	10	10	14	10	12	11	15	11	13	14	16	17	18	20	22	17
Picidae	Picus xanthopygaeus				1	15	4	16	4	15	4	19	3	17	5	18	7	17	7	27	5
Picidae	Picus squamatus		H		1	0	3	0	3	0	1	0	0	0	4	0	4	0	3	0	2
Picidae	Picus awokera				1	7	15	10	13	8	15	8	14	8	24	13	30	13	31	12	29
Picidae	Picus viridis				1	8	24	8	19	11	24	11	17	13	28	12	24	19	28	17	20
Picidae	Picus rabieri				1	3	7	3	8	6	8	16	8	11	21	5	22	9	22	20	16
Picidae	Picus erythropygius				1	15	5	20	5	12	5	13	7	17	9	21	15	15	18	19	17
Picidae	Picus canus				1	12	11	12	10	13	12	13	10	14	14	14	13	17	15	18	12
Picidae	Dinopium rafflesii				1	13	3	16	3	16	3	18	3	20	4	23	4	27	4	28	4
Picidae	Dinopium shorii				1	25	12	27	12	27	16	26	15	30	17	30	18	35	19	41	18
Picidae	Dinopium javanense				1	13	7	17	7	15	7	18	7	17	9	21	10	23	12	26	10
Picidae	Dinopium benghalense		H		1	0	2	0	2	0	1	0	1	0	3	0	4	0	5	0	1
Picidae	Chrysocolaptes lucidus				1	19	10	22	10	19	11	22	11	23	13	26	14	27	16	32	14
Picidae	Chrysocolaptes festivus				1	20	3	23	3	21	2	29	1	23	4	26	4	24	4	46	1

Family	Species	IUCN	Ag	R	Range Source	Percentage loss in geographic range 2050 TG		AM		GO		OS		2100 TG		AM		GO		OS	
						H	C	H	C	H	C	H	C	H	C	H	C	H	C	H	C
Picidae	*Gecinulus grantia*				1	25	9	27	8	24	10	29	8	28	11	30	11	30	14	35	10
Picidae	*Gecinulus viridis*				1	11	9	18	9	11	10	13	11	12	11	19	12	18	12	19	12
Picidae	*Sapheopipo noguchii*	T		x	1	69	0x	0	0x	0	0x	0	0x	84	0x	0	0x	0	0x	0	0x
Picidae	*Blythipicus rubiginosus*				1	9	2	13	2	11	3	15	2	15	2	17	2	21	3	22	2
Picidae	*Blythipicus pyrrhotis*				1	18	5	19	5	18	6	19	5	20	7	21	7	22	8	23	7
Picidae	*Reinwardtipicus validus*				1	13	3	16	3	16	3	19	3	20	4	23	3	30	3	28	3
Picidae	*Meiglyptes tristis*				1	13	4	17	4	18	4	21	4	20	5	24	5	30	5	30	5
Picidae	*Meiglyptes jugularis*				1	10	11	14	9	10	11	12	11	11	12	15	14	15	17	18	16
Picidae	*Meiglyptes tukki*				1	12	2	17	3	15	3	18	3	18	3	23	3	27	3	28	3
Picidae	*Hemicircus concretus*				1	13	2	16	2	16	2	18	2	20	3	22	3	29	3	27	2
Picidae	*Hemicircus canente*				1	14	6	19	5	16	6	19	6	17	9	22	11	21	14	30	12
Picidae	*Mulleripicus fulvus*				1	12	14	27	10	18	14	13	13	15	15	30	22	27	28	23	30
Picidae	*Mulleripicus funebris*				1	14	11	25	10	14	12	22	11	20	18	31	14	27	21	34	12
Picidae	*Mulleripicus pulverulentus*				1	13	6	17	6	15	8	18	7	19	9	20	10	22	11	26	10
Megalaimidae	*Psilopogon pyrolophus*				1	15	9	21	9	24	8	30	8	20	9	29	9	50	8	37	8
Megalaimidae	*Megalaima virens*				1	19	3	20	2	19	3	20	3	21	4	21	4	21	4	22	4
Megalaimidae	*Megalaima lagrandieri*				1	5	19	8	17	12	19	16	11	7	19	9	17	20	19	24	11
Megalaimidae	*Megalaima zeylanica*				1	23	3	25	3	25	2	31	1	26	3	29	3	30	3	47	1
Megalaimidae	*Megalaima lineata*		H		1	0	25	0	23	0	25	0	21	0	25	0	23	0	25	0	22
Megalaimidae	*Megalaima viridis*				1	21	4	36	4	34	3	38	2	25	4	43	4	38	6	62	2
Megalaimidae	*Megalaima faiostricta*				1	10	14	12	12	12	15	19	13	12	17	13	20	18	23	25	19
Megalaimidae	*Megalaima corvina*			x	1	0	0x	0	0x	0	0x	0	0x	0	0x	0	0x	0	0x	0	0x
Megalaimidae	*Megalaima chrysopogon*				1	10	3	14	3	13	3	18	3	16	4	21	3	25	4	27	3
Megalaimidae	*Megalaima rafflesii*				1	8	2	13	2	12	2	17	2	15	3	18	2	23	2	26	2
Megalaimidae	*Megalaima mystacophanos*				1	10	2	13	2	13	2	18	3	16	3	19	3	25	3	27	3
Megalaimidae	*Megalaima javensis*			x	1	11	0x	0	0x	0	0x	0	0x	12	0x	8	0x	18	0x	8	1x
Megalaimidae	*Megalaima flavifrons*			x	1	35	0x	33	0x	35	0x	35	0x	35	0x	33	0x	35	0x	35	0x
Megalaimidae	*Megalaima franklinii*				1	30	9	33	8	30	10	34	9	35	12	36	11	36	13	40	11
Megalaimidae	*Megalaima oorti*				1	12	11	13	11	17	10	20	9	16	12	16	14	28	13	26	11
Megalaimidae	*Megalaima asiatica*				1	31	6	33	6	30	7	32	7	35	9	36	8	35	9	37	9
Megalaimidae	*Megalaima monticola*				1	19	0	25	3	18	3	22	0	32	0	31	3	31	3	40	0
Megalaimidae	*Megalaima incognita*				1	9	14	12	12	11	14	16	13	11	19	12	21	16	26	22	21
Megalaimidae	*Megalaima henricii*				1	12	3	14	4	14	3	22	3	18	4	20	4	25	4	31	3
Megalaimidae	*Megalaima armillaris*			x	1	3	0x	0	0x	0	0x	0	0x	3	0x	3	0x	17	0x	3	0x
Megalaimidae	*Megalaima pulcherrima*			x	1	3	0x	29	0x	4	0x	61	0x	3	0x	33	0x	30	0x	61	0x
Megalaimidae	*Megalaima australis*				1	19	8	22	7	19	9	22	8	24	10	26	11	27	13	30	11
Megalaimidae	*Megalaima eximia*				1	24	0	22	4	10	5	22	1	31	0	27	4	22	5	36	1
Megalaimidae	*Megalaima rubricapilla*				1	33	0	50	1	38	2	51	1	35	1	59	1	48	2	76	1
Megalaimidae	*Megalaima haemacephala*		H		1	0	5	0	5	0	5	0	5	0	8	0	9	0	11	0	7
Megalaimidae	*Calorhamphus fuliginosus*				1	11	3	17	3	16	3	23	3	18	4	25	3	30	4	32	3
Lybiidae	*Gymnobucco calvus*				1	29	5	15	6	23	4	42	4	40	6	25	8	45	5	48	4
Lybiidae	*Gymnobucco peli*				1	27	5	15	6	23	4	45	4	42	6	25	7	47	5	53	5
Lybiidae	*Gymnobucco sladeni*				1	38	5	26	9	41	6	63	4	57	5	38	9	71	7	72	4
Lybiidae	*Gymnobucco bonapartei*				1	36	7	23	10	33	9	57	5	52	9	33	13	59	11	65	6

Family	Species	IUCN	Ag	R	Range Source	Percentage loss in geographic range 2050 TG H	TG C	AM H	AM C	GO H	GO C	OS H	OS C	2100 TG H	TG C	AM H	AM C	GO H	GO C	OS H	OS C
Lybiidae	*Stactolaema leucotis*				1	33	1	29	1	33	0	33	0	33	2	31	4	33	0	33	0
Lybiidae	*Stactolaema anchietae*				1	41	0	23	1	42	0	44	0	43	0	25	3	42	1	44	0
Lybiidae	*Stactolaema whytii*				1	47	0	40	0	47	0	49	0	48	1	42	4	49	0	49	0
Lybiidae	*Stactolaema olivacea*				1	22	0	17	2	22	0	22	0	22	1	17	4	23	0	23	0
Lybiidae	*Pogoniulus scolopaceus*		H		1	0	7	0	9	0	8	0	4	0	8	0	11	0	9	0	5
Lybiidae	*Pogoniulus coryphaeus*				1	31	4	24	6	32	6	37	3	39	5	30	8	39	7	38	4
Lybiidae	*Pogoniulus leucomystax*				1	35	0	31	1	35	0	35	0	35	2	34	3	35	0	35	0
Lybiidae	*Pogoniulus simplex*				1	33	0	28	2	33	0	33	0	33	0	29	4	33	0	33	0
Lybiidae	*Pogoniulus atroflavus*				1	34	6	22	8	32	7	55	4	50	6	32	10	58	8	63	5
Lybiidae	*Pogoniulus subsulphureus*				1	34	5	22	7	32	6	54	4	49	6	33	8	58	7	62	4
Lybiidae	*Pogoniulus bilineatus*				1	37	5	26	7	37	6	51	3	47	6	33	10	51	7	55	4
Lybiidae	*Pogoniulus chrysoconus*				1	28	2	18	4	29	3	34	2	32	3	21	8	34	4	35	3
Lybiidae	*Pogoniulus pusillus*				1	40	3	19	15	44	1	44	1	42	3	23	18	45	1	45	1
Lybiidae	*Buccanodon duchaillui*				1	33	6	22	8	32	7	52	4	47	6	31	10	54	8	58	4
Lybiidae	*Tricholaema hirsuta*				1	35	6	23	8	33	8	55	4	51	7	33	11	57	9	62	5
Lybiidae	*Tricholaema diademata*				1	40	2	26	8	43	1	43	1	42	2	32	10	45	2	45	2
Lybiidae	*Tricholaema frontata*				1	51	0	36	1	52	0	53	0	53	1	37	5	53	0	53	0
Lybiidae	*Tricholaema leucomelas*				1	8	4	4	6	8	5	8	5	8	7	5	11	8	9	8	8
Lybiidae	*Tricholaema lacrymosa*				1	39	2	29	5	40	1	40	1	39	2	34	6	40	1	40	1
Lybiidae	*Tricholaema melanocephal*				1	42	4	14	25	49	1	50	1	46	5	18	29	50	3	50	2
Lybiidae	*Lybius undatus*				1	62	2	22	20	69	0	69	1	67	2	33	25	69	2	69	2
Lybiidae	*Lybius vieilloti*				1	11	3	6	3	13	3	18	2	16	3	8	6	19	5	19	4
Lybiidae	*Lybius leucocephalus*		H		1	0	3	0	6	0	2	0	1	0	3	0	8	0	4	0	2
Lybiidae	*Lybius chaplini*				1	44	0	34	0	44	0	44	0	44	0	34	11	44	0	44	0
Lybiidae	*Lybius rubrifacies*				1	31	0	17	0	31	0	31	0	31	0	22	5	31	0	31	0
Lybiidae	*Lybius guifsobalito*				1	36	2	21	7	40	1	40	1	39	2	26	9	41	2	41	1
Lybiidae	*Lybius torquatus*				1	36	1	27	2	35	1	38	1	37	2	29	6	37	2	38	1
Lybiidae	*Lybius melanopterus*				1	24	2	20	2	25	0	25	0	24	2	22	2	26	0	26	0
Lybiidae	*Lybius minor*				1	50	4	35	5	48	5	60	2	57	5	40	8	57	5	62	3
Lybiidae	*Lybius bidentatus*				1	36	5	21	9	37	7	51	4	46	6	28	13	51	9	54	6
Lybiidae	*Lybius dubius*				1	15	3	8	3	17	3	23	2	21	3	11	6	25	4	24	3
Lybiidae	*Lybius rolleti*		H		1	0	1	0	3	0	1	0	1	0	1	0	4	0	1	0	1
Lybiidae	*Trachyphonus purpuratus*				1	36	6	23	9	34	7	55	4	51	7	32	12	57	9	61	5
Lybiidae	*Trachyphonus vaillantii*				1	32	1	25	2	32	2	34	1	34	3	27	7	34	3	34	2
Lybiidae	*Trachyphonus margaritatus*				1	7	3	3	4	8	3	10	2	9	3	3	7	10	7	10	5
Lybiidae	*Trachyphonus erythroceph*				1	43	5	16	22	50	1	51	1	47	5	21	27	51	3	51	3
Lybiidae	*Trachyphonus darnaudii*				1	47	4	19	24	54	1	54	1	51	5	24	28	56	1	55	2
Ramphastidae	*Capito aurovirens*				1	3	2	2	1	2	2	5	1	6	2	7	2	8	2	12	1
Ramphastidae	*Capito wallacei*	T		x	14	4	0x	4	0x	4	0x	4	0x	27	0x	9	0x	27	0x	27	0x
Ramphastidae	*Capito maculicoronatus*				1	12	5	21	10	11	7	16	8	21	8	22	10	22	14	31	12
Ramphastidae	*Capito squamatus*				1	18	3x	16	3x	28	3x	20	0x	19	3x	17	3x	28	3x	31	0x
Ramphastidae	*Capito hypoleucus*	T			1	20	2	14	4	15	6	23	0x	22	2x	21	6x	20	6x	23	2x
Ramphastidae	*Capito dayi*				1	5	24	3	27	2	28	6	26	9	28	4	28	13	28	11	28
Ramphastidae	*Capito quinticolor*				1	15	2	16	5	15	0	8	5	24	2	16	5	15	5	20	5

Family	Species	IUCN	Ag	R	Range Source	2050 TG H	2050 TG C	2050 AM H	2050 AM C	2050 GO H	2050 GO C	2050 OS H	2050 OS C	2100 TG H	2100 TG C	2100 AM H	2100 AM C	2100 GO H	2100 GO C	2100 OS H	2100 OS C
Ramphastidae	*Capito niger*				1	3	7	2	7	3	7	5	7	6	8	6	9	9	10	11	9
Ramphastidae	*Capito brunneipectus*				1	26	10	10	10	16	7	16	7	38	10	23	10	41	7	40	7
Ramphastidae	*Eubucco richardsoni*				1	4	4	2	4	3	4	6	4	7	5	7	5	9	5	12	5
Ramphastidae	*Eubucco bourcierii*				1	19	8	23	9	17	9	29	9	25	12	28	15	32	16	39	13
Ramphastidae	*Eubucco tucinkae*				1	7	5	1	5	7	4	9	3	17	6	13	6	17	5	19	5
Ramphastidae	*Eubucco versicolor*				1	23	10	23	8	24	8	35	7	31	10	34	10	40	8	45	8
Ramphastidae	*Semnornis frantzii*				1	26	10	39	19	20	19	43	19	34	10	39	26x	35	26	59	19x
Ramphastidae	*Semnornis ramphastinus*				1	13	9	8	10	18	10	16	3	14	10	11	10	22	10x	24	3
Ramphastidae	*Aulacorhynchus prasinus*				1	14	6	16	5	13	6	24	5	20	7	23	10	26	9	31	6
Ramphastidae	*Aulacorhynchus sulcatus*				1	17	2	19	1	25	2	17	2	20	14	29	8	28	17	26	13
Ramphastidae	*Aulacorhynchus derbianus*				1	17	9	15	9	16	9	24	9	22	10	23	15	29	14	31	14
Ramphastidae	*Aulacorhynchus haematop*				1	14	4	18	4	13	6	22	2	22	5	28	5	28	9	27	4
Ramphastidae	*Aulacorhynchus huallagae*	T		x	1	0	46x	0	46x	0	46x	46	0x	0	46x	0	46x	0	78x	46	0x
Ramphastidae	*Aulacorhynchus coeruleicin*				1	28	7	20	5	19	6	39	6	37	7	35	9	46	7	47	8
Ramphastidae	*Pteroglossus inscriptus*				1	6	12	4	12	4	12	10	11	11	13	9	14	16	15	19	13
Ramphastidae	*Pteroglossus viridis*				1	3	14	3	14	3	14	6	14	7	15	5	17	13	18	12	17
Ramphastidae	*Pteroglossus bitorquatus*				1	7	20	4	21	4	22	11	20	13	22	8	23	22	23	22	21
Ramphastidae	*Pteroglossus azara*				1	4	5	3	5	4	5	7	5	7	7	8	7	10	7	14	7
Ramphastidae	*Pteroglossus castanotis*				1	4	13	3	12	3	13	7	12	8	15	8	15	11	16	15	14
Ramphastidae	*Pteroglossus aracari*				1	8	14	5	14	7	14	13	13	13	17	10	18	23	18	24	16
Ramphastidae	*Pteroglossus torquatus*				1	13	4	19	4	16	5	26	4	17	5	23	8	22	9	32	5
Ramphastidae	*Pteroglossus frantzii*				1	27	9x	31	22x	15	22x	35	22x	40	9x	31	38x	31	35x	57	22x
Ramphastidae	*Pteroglossus pluricinctus*				1	5	3	3	3	3	3	7	3	8	4	9	5	11	5	16	5
Ramphastidae	*Pteroglossus beauharnaesi*				1	3	10	2	10	2	10	5	10	6	11	5	13	8	13	10	13
Ramphastidae	*Baillonius bailloni*				1	21	12	17	10	27	8	30	9	29	21	29	17	45	12	43	15
Ramphastidae	*Andigena laminirostris*			x	1	11	10x	22	12x	40	12x	51	0x	13	21x	43	12x	48	12x	54	0x
Ramphastidae	*Andigena hypoglauca*				1	15	8	19	7	20	9	29	7	26	9	29	12	41	11	37	8
Ramphastidae	*Andigena cucullata*				1	26	3	13	1	15	1	27	3	31	3	26	6	29	6	27	8
Ramphastidae	*Andigena nigrirostris*				1	16	2	20	4	14	3	29	4	24	4	27	8	35	8	33	8
Ramphastidae	*Selenidera spectabilis*				1	19	4	24	8	22	7	31	8	24	5	27	10	31	9	41	8
Ramphastidae	*Selenidera reinwardtii*				1	3	2	2	2	3	2	5	2	6	3	7	4	8	4	13	3
Ramphastidae	*Selenidera nattereri*				1	0	6	0	6	0	6	0	6	0	7	0	8	0	8	1	8
Ramphastidae	*Selenidera culik*				1	4	13	3	14	4	13	8	13	8	14	6	16	15	16	14	15
Ramphastidae	*Selenidera maculirostris*				1	20	9	18	8	26	6	29	7	31	16	29	15	44	11	45	14
Ramphastidae	*Selenidera gouldii*				1	7	20	3	22	5	23	11	21	11	23	8	24	23	25	22	23
Ramphastidae	*Ramphastos sulfuratus*				1	14	5	24	5	17	7	31	5	18	6	29	11	26	11	39	7
Ramphastidae	*Ramphastos brevis*				1	13	4	16	6	15	3	15	3	23	4	17	6	19	6	26	3
Ramphastidae	*Ramphastos vitellinus*				1	5	11	4	11	4	12	8	11	9	13	8	14	14	14	16	13
Ramphastidae	*Ramphastos dicolorus*				1	17	9	15	8	23	7	25	7	25	14	26	13	36	10	38	12
Ramphastidae	*Ramphastos ambiguus*				1	17	5	21	6	19	6	29	6	25	6	31	9	33	10	38	7
Ramphastidae	*Ramphastos tucanus*				1	5	11	4	11	4	12	8	11	9	12	8	13	15	14	16	13
Ramphastidae	*Ramphastos toco*				1	9	15	6	14	9	15	14	12	14	19	13	20	24	21	25	18
Galbulidae	*Galbalcyrhynchus leucotis*				1	7	1	4	0	4	0	9	0	12	1	13	1	15	0	22	0
Galbulidae	*Galbalcyrhynchus purusian*				1	3	6	3	6	4	6	6	6	7	8	9	10	8	11	12	8

Family	Species	IUCN	Ag	R	Range Source	2050 TG H	2050 TG C	2050 AM H	2050 AM C	2050 GO H	2050 GO C	2050 OS H	2050 OS C	2100 TG H	2100 TG C	2100 AM H	2100 AM C	2100 GO H	2100 GO C	2100 OS H	2100 OS C
						Percentage loss in geographic range															
Galbulidae	*Brachygalba salmoni*				1	11	7	24	12	6	12	24	7	13	15	25	12	18	23	35	15
Galbulidae	*Brachygalba goeringi*				1	24	6	24	7	25	3	33	3	34	7	36	11	53	10	52	8
Galbulidae	*Brachygalba lugubris*				1	5	16	2	17	3	17	7	16	8	18	6	20	13	21	13	20
Galbulidae	*Brachygalba albogularis*				1	0	4	0	4	1	4	2	4	3	6	4	8	3	8	4	6
Galbulidae	*Jacamaralcyon tridactyla*	T			1	21	12	16	11	30	8	27	11	25	21	24	18	47	11	42	17
Galbulidae	*Galbula albirostris*				1	4	9	2	9	3	9	5	9	7	10	5	11	10	11	11	11
Galbulidae	*Galbula cyanicollis*				1	5	14	3	14	3	14	7	14	9	15	6	15	15	16	16	15
Galbulidae	*Galbula ruficauda*		H		1	0	15	0	13	0	15	0	11	0	19	0	17	0	19	0	14
Galbulidae	*Galbula galbula*				1	3	14	2	14	3	14	5	14	6	16	4	18	11	19	10	18
Galbulidae	*Galbula pastazae*	T			1	25	6	20	7	20	7	39	7	33	10	45	17	64	9	57	14
Galbulidae	*Galbula tombacea*				1	5	1	3	0	3	2	7	0	9	1	6	0	11	2	12	0
Galbulidae	*Galbula cyanescens*				1	3	5	3	4	3	4	6	4	6	6	9	6	7	7	14	5
Galbulidae	*Galbula chalcothorax*				1	4	1	3	0	4	1	7	0	10	1	10	1	12	1	18	0
Galbulidae	*Galbula leucogastra*				1	3	14	2	13	2	13	5	13	6	15	5	15	11	16	11	14
Galbulidae	*Galbula dea*				1	4	9	3	9	3	9	7	8	8	10	7	10	13	11	15	10
Galbulidae	*Jacamerops aureus*				1	5	8	4	8	4	8	8	7	9	9	8	10	15	10	17	9
Bucconidae	*Notharchus macrorhynchos*				1	7	10	6	10	7	11	11	10	11	12	11	14	16	14	19	12
Bucconidae	*Notharchus swainsoni*				1	16	12	15	11	24	6	25	9	22	24	25	24	45	14	42	18
Bucconidae	*Notharchus pectoralis*				1	11	5	16	8	13	6	16	5	18	7	17	8	22	10	26	8
Bucconidae	*Notharchus ordii*				1	2	5	0	5	1	5	4	5	4	6	1	6	8	6	8	6
Bucconidae	*Notharchus tectus*				1	6	13	4	14	5	15	8	14	10	15	9	17	15	18	16	17
Bucconidae	*Bucco macrodactylus*				1	3	9	2	9	2	9	4	9	6	11	5	11	6	12	9	11
Bucconidae	*Bucco tamatia*				1	3	11	2	11	3	12	5	11	7	13	5	14	10	15	11	14
Bucconidae	*Bucco noanamae*				1	9	0	18	7	10	0	4	7	22	4	19	7	17	11	21	11
Bucconidae	*Bucco capensis*				1	3	9	3	9	2	10	5	10	6	11	6	11	10	12	11	12
Bucconidae	*Nystalus radiatus*				1	14	5	17	7	12	5	17	5	21	7	19	7	20	9	28	7
Bucconidae	*Nystalus chacuru*				1	8	15	6	11	8	12	12	9	13	19	10	15	18	17	22	13
Bucconidae	*Nystalus striolatus*				1	5	19	3	20	5	21	9	19	9	22	10	25	15	24	19	23
Bucconidae	*Nystalus maculatus*				1	7	15	5	14	6	15	10	11	9	18	9	19	14	21	18	16
Bucconidae	*Hypnelus ruficollis*				1	23	2	17	6	17	7	21	4	27	8	20	15	29	22	27	11
Bucconidae	*Hypnelus bicinctus*				14	16	13	5	16	13	14	15	14	19	14	13	18	26	16	26	15
Bucconidae	*Malacoptila fusca*				1	3	6	1	6	2	6	5	6	5	6	4	7	9	7	12	7
Bucconidae	*Malacoptila semicincta*				1	1	7	1	7	1	7	3	7	2	10	3	12	4	13	4	10
Bucconidae	*Malacoptila striata*				1	13	13	10	16	15	13	18	13	17	22	16	26	31	22	27	22
Bucconidae	*Malacoptila fulvogularis*				1	17	8	18	5	20	6	33	6	26	8	28	10	38	9	46	7
Bucconidae	*Malacoptila rufa*				1	4	13	3	13	3	13	7	13	8	15	6	15	15	16	16	15
Bucconidae	*Malacoptila panamensis*				1	18	6	27	8	20	8	33	6	24	7	32	10	30	11	42	7
Bucconidae	*Malacoptila mystacalis*				1	16	3	18	5	14	5	22	3	23	6	25	9	28	11	31	4
Bucconidae	*Micromonacha lanceolata*				1	5	1	5	1	5	1	9	1	10	1	12	2	11	1	22	1
Bucconidae	*Nonnula rubecula*				1	3	8	2	7	3	8	5	7	6	9	5	9	9	10	10	9
Bucconidae	*Nonnula sclateri*				1	1	7	1	7	1	7	4	7	6	9	5	10	5	11	9	9
Bucconidae	*Nonnula brunnea*				1	9	3	4	1	4	0	14	1	12	3	9	1	17	0	21	1
Bucconidae	*Nonnula frontalis*				1	14	5	18	9	13	9	26	5	19	9	22	10	28	15	35	9
Bucconidae	*Nonnula ruficapilla*				1	4	18	3	19	4	19	7	18	9	21	7	21	15	22	16	20

Family	Species	IUCN	Ag	R	Range Source	2050 TG H	2050 TG C	2050 AM H	2050 AM C	2050 GO H	2050 GO C	2050 OS H	2050 OS C	2100 TG H	2100 TG C	2100 AM H	2100 AM C	2100 GO H	2100 GO C	2100 OS H	2100 OS C
Bucconidae	*Nonnula amaurocephala*				1	0	0	0	0	0	0	0	0	0	0	0	0	0	0	0	0
Bucconidae	*Hapaloptila castanea*				1	12	6	17	2	17	6	23	6	16	10	22	7	25	6	25	6
Bucconidae	*Monasa atra*				1	2	14	2	14	3	13	5	13	5	15	4	18	10	18	9	18
Bucconidae	*Monasa nigrifrons*				1	6	13	4	12	4	13	10	11	10	14	9	15	16	16	20	13
Bucconidae	*Monasa morphoeus*				1	6	10	4	10	5	10	10	9	10	11	9	12	16	13	19	11
Bucconidae	*Monasa flavirostris*				1	2	3	2	3	2	3	4	3	5	4	6	5	5	5	11	4
Bucconidae	*Chelidoptera tenebrosa*				1	6	13	4	12	5	13	10	10	9	16	8	15	14	16	18	13
Bucerotidae	*Tropicranus albocristatus*				1	34	7	21	9	31	8	54	4	50	7	31	12	57	9	62	5
Bucerotidae	*Tockus hartlaubi*				1	33	5	22	8	32	6	53	3	50	6	32	9	58	7	62	4
Bucerotidae	*Tockus camurus*				1	34	6	21	9	33	7	55	4	51	8	31	12	58	9	63	6
Bucerotidae	*Tockus monteiri*				1	15	8	7	11	14	9	13	9	15	12	7	21	14	15	13	15
Bucerotidae	*Tockus erythrorhynchus*				1	19	3	9	8	21	2	23	2	22	4	11	11	23	5	23	4
Bucerotidae	*Tockus flavirostris*				1	35	4	12	19	40	1	40	1	38	4	16	22	40	3	41	2
Bucerotidae	*Tockus leucomelas*				1	12	5	7	8	12	6	12	6	12	9	7	16	12	11	12	10
Bucerotidae	*Tockus deckeni*				1	46	5	18	25	53	1	53	1	49	5	22	29	53	3	53	2
Bucerotidae	*Tockus alboterminatus*				1	39	2	30	3	40	2	44	2	43	3	33	7	43	2	44	2
Bucerotidae	*Tockus bradfieldi*				1	13	1	8	3	13	0	13	0	13	2	8	9	13	3	13	1
Bucerotidae	*Tockus fasciatus*				1	34	7	22	9	34	8	52	5	47	8	30	13	54	10	58	6
Bucerotidae	*Tockus hemprichii*				1	48	1	32	1	50	0	50	0	49	1	39	3	50	0	50	0
Bucerotidae	*Tockus nasutus*				1	24	3	15	5	26	3	29	2	28	4	18	9	30	4	30	4
Bucerotidae	*Tockus pallidirostris*				1	55	2	41	3	55	2	59	1	59	3	44	6	58	2	60	1
Bucerotidae	*Ocyceros griseus*				1	24	2	44	2	37	2	47	1	26	2	53	2	43	3	80	1
Bucerotidae	*Ocyceros gingalensis*			x	1	45	0x	42	0x	39	2x	44	0x	46	0x	48	0x	47	2x	49	0x
Bucerotidae	*Ocyceros birostris*				1	21	4	21	4	21	2	27	1	23	5	24	5	23	5	40	1
Bucerotidae	*Anthracoceros coronatus*				1	30	2	40	2	34	1	43	1	34	3	46	2	42	3	75	1
Bucerotidae	*Anthracoceros albirostris*				1	19	6	22	6	20	7	23	7	24	8	26	9	29	10	32	9
Bucerotidae	*Anthracoceros malayanus*				1	13	2	16	3	15	3	19	2	20	3	22	3	29	3	29	2
Bucerotidae	*Anthracoceros marchei*	T		x	1	21	26x	21	29x	21	21x	24	20x	21	26x	21	48x	42	39x	35	48x
Bucerotidae	*Anthracoceros montani*	T		x	1	61	0x	23	40x	28	38x	21	38x	61	0x	49	40x	28	40x	21	40x
Bucerotidae	*Buceros rhinoceros*				1	13	2	16	3	15	3	18	2	20	3	22	3	28	3	28	2
Bucerotidae	*Buceros bicornis*				1	27	8	31	7	28	8	32	8	31	11	35	11	36	13	41	11
Bucerotidae	*Buceros hydrocorax*				1	14	12	24	11	13	12	22	12	20	18	29	15	26	21	34	13
Bucerotidae	*Buceros vigil*				1	13	2	16	3	15	3	19	2	20	3	22	3	28	3	28	3
Bucerotidae	*Anorrhinus tickelli*				1	4	16	6	16	0	16	0	16	6	16	6	27	0	27	1	27
Bucerotidae	*Anorrhinus austeni*				1	25	13	28	11	26	14	30	11	31	17	32	15	35	18	37	15
Bucerotidae	*Anorrhinus galeritus*				1	13	2	16	3	15	3	18	2	20	3	22	3	28	3	28	3
Bucerotidae	*Penelopides affinis*				1	19	14	26	16	13	14	27	16	31	17	26	16	28	21	40	17
Bucerotidae	*Penelopides manillae*				1	8	9	22	5	15	10	16	6	8	19	34	13	25	22	27	8
Bucerotidae	*Penelopides mindorensis*	T		x	1	0	8x	4	3x	1	8x	42	5x	0	27x	22	5x	7	27x	42	11x
Bucerotidae	*Penelopides panini*	T		x	1	31	21x	30	19x	6	26x	7	21x	31	21x	30	19x	10	26x	28	21x
Bucerotidae	*Penelopides exarhatus*				1	14	14	27	9	19	13	17	12	16	15	30	21	28	29	24	26
Bucerotidae	*Aceros comatus*				1	13	3	16	4	15	4	18	3	19	4	21	5	27	5	27	4
Bucerotidae	*Aceros nipalensis*	T			1	34	8	35	8	32	11	32	10	38	11	39	11	37	13	39	12
Bucerotidae	*Aceros corrugatus*				1	14	2	17	3	16	2	18	3	21	3	23	3	29	3	28	3

Family	Species	IUCN	Ag	R	Range Source	Percentage loss in geographic range 2050 TG		AM		GO		OS		2100 TG		AM		GO		OS	
						H	C	H	C	H	C	H	C	H	C	H	C	H	C	H	C
Bucerotidae	*Aceros waldeni*	T		x	1	31	24x	33	21x	0	29x	6	24x	31	24x	33	21x	0	29x	31	24x
Bucerotidae	*Aceros leucocephalus*				1	17	16	26	18	8	18	19	18	26	19	26	18	24	23	34	18
Bucerotidae	*Aceros cassidix*				1	14	13	25	10	18	13	17	12	16	15	28	21	27	27	24	25
Bucerotidae	*Aceros undulatus*				1	18	6	21	6	19	7	22	7	23	8	25	9	28	10	30	9
Bucerotidae	*Aceros everetti*	T		x	1	0	0x	0	0x	0	0x	0	0x	0	11x	0	11x	0	11x	0	0x
Bucerotidae	*Aceros subruficollis*	T			1	9	9	16	9	9	9	16	9	12	9	18	15	11	15	22	15
Bucerotidae	*Aceros plicatus*				1	6	3	7	2	19	1	17	1	13	5	14	5	31	4	29	2
Bucerotidae	*Ceratogymna bucinator*				1	42	2	32	3	41	2	45	1	44	3	34	7	44	3	46	2
Bucerotidae	*Ceratogymna fistulator*				1	33	6	22	9	33	7	53	5	48	7	31	13	56	10	59	6
Bucerotidae	*Ceratogymna brevis*				1	45	0	36	1	45	0	45	0	45	1	40	3	45	0	45	0
Bucerotidae	*Ceratogymna subcylindricu*				1	40	6	20	10	41	7	52	4	50	8	26	15	53	9	55	6
Bucerotidae	*Ceratogymna cylindricus*				1	34	8	22	10	32	9	55	5	49	9	31	13	56	11	62	6
Bucerotidae	*Ceratogymna atrata*				1	35	6	22	8	32	8	54	4	49	7	31	11	55	9	60	5
Bucerotidae	*Ceratogymna elata*				1	22	6	13	7	23	7	32	4	29	7	18	12	33	9	35	6
Bucorvidae	*Bucorvus abyssinicus*				1	22	2	11	6	25	2	30	2	28	3	15	9	31	4	31	3
Bucorvidae	*Bucorvus leadbeateri*				1	38	2	29	2	39	1	41	1	40	3	31	6	40	2	41	2
Upupidae	*Upupa epops*		H		1	0	7	0	7	0	7	0	6	0	9	0	11	0	11	0	9
Phoeniculidae	*Phoeniculus purpureus*				1	22	3	14	5	24	3	26	2	25	4	17	9	27	4	27	3
Phoeniculidae	*Phoeniculus damarensis*				1	28	8	10	21	33	4	33	4	30	10	11	28	33	5	33	6
Phoeniculidae	*Phoeniculus somaliensis*				1	46	4	14	24	53	0	53	0	50	4	19	28	53	1	53	1
Phoeniculidae	*Phoeniculus bollei*				1	31	6	20	10	32	9	46	4	43	7	28	12	47	9	53	5
Phoeniculidae	*Phoeniculus castaneiceps*				1	29	4	19	6	26	6	43	5	44	4	30	7	44	8	54	6
Rhinopomastida	*Rhinopomastus aterrimus*				1	26	3	14	5	28	3	34	2	32	4	18	8	35	5	35	4
Rhinopomastida	*Rhinopomastus cyanomela*				1	27	2	22	4	28	2	28	2	28	4	23	8	28	4	28	4
Rhinopomastida	*Rhinopomastus minor*				1	41	4	15	23	48	1	47	1	44	4	19	26	49	2	48	2
Trogonidae	*Apaloderma narina*				1	39	4	28	5	38	4	50	2	47	5	33	9	50	5	53	3
Trogonidae	*Apaloderma aequatoriale*				1	31	6	21	8	25	8	54	5	49	7	30	8	55	8	63	5
Trogonidae	*Apaloderma vittatum*				1	36	4	28	4	38	3	41	3	39	5	33	7	42	4	42	3
Trogonidae	*Pharomachrus mocinno*				1	17	2	27	5	19	6	29	5	19	2	28	10	27	8	34	5
Trogonidae	*Pharomachrus antisianus*				1	19	7	19	6	16	7	30	6	25	8	27	10	33	10	37	8
Trogonidae	*Pharomachrus fulgidus*				1	23	0	13	0	23	0	18	0	30	0	22	1	29	0	29	1
Trogonidae	*Pharomachrus auriceps*				1	18	4	17	3	15	4	26	4	23	6	25	8	31	10	33	7
Trogonidae	*Pharomachrus pavoninus*				1	3	7	3	7	2	7	5	7	7	8	6	9	8	10	12	8
Trogonidae	*Euptilotis neoxenus*				1	18	10	38	5	20	9	52	6	25	12	39	11	28	21	60	12
Trogonidae	*Priotelus temnurus*				1	9	17x	29	17x	15	11x	40	7x	18	17x	32	19x	20	12x	42	7x
Trogonidae	*Priotelus roseigaster*			x	1	6	4x	6	3x	6	3x	9	0x	6	4x	6	3x	6	3x	9	0x
Trogonidae	*Trogon massena*				1	15	6	23	6	17	7	29	6	21	7	27	10	25	10	38	7
Trogonidae	*Trogon melanurus*				1	4	9	3	9	3	9	6	9	8	10	7	11	12	11	13	10
Trogonidae	*Trogon clathratus*				1	32	7	40	19	22	19	46	14	39	7	42	22	37	22	58	14
Trogonidae	*Trogon comptus*				1	19	2	13	7	14	2	17	5	24	2	14	7	14	7	22	5
Trogonidae	*Trogon bairdii*				1	25	8	27	17	23	17	36	17	34	8	28	32	40	26	52	17x
Trogonidae	*Trogon viridis*				1	5	11	4	12	4	12	8	11	9	13	7	14	14	15	16	13
Trogonidae	*Trogon citreolus*				1	7	5	17	2	9	5	28	2	8	8	17	20	15	8	30	2
Trogonidae	*Trogon melanocephalus*				1	12	4	22	3	18	5	31	3	15	5	27	10	23	9	35	5

Family	Species	IUCN	Ag	R	Range Source	Percentage loss in geographic range 2050 TG H	C	AM H	C	GO H	C	OS H	C	2100 TG H	C	AM H	C	GO H	C	OS H	C
Trogonidae	*Trogon mexicanus*				1	15	7	32	3	19	6	41	2	19	9	33	10	25	12	44	5
Trogonidae	*Trogon elegans*				1	11	9	25	4	13	8	35	4	14	11	26	16	18	13	38	6
Trogonidae	*Trogon collaris*				1	5	8	5	8	5	8	9	7	9	9	9	10	14	11	16	9
Trogonidae	*Trogon personatus*				1	14	6	18	6	15	7	24	6	22	10	27	11	28	14	31	12
Trogonidae	*Trogon rufus*				1	7	8	6	8	7	8	10	7	11	9	10	10	18	10	19	9
Trogonidae	*Trogon surrucura*				1	19	8	15	7	24	5	27	5	28	14	27	12	39	9	41	10
Trogonidae	*Trogon curucui*				1	5	13	3	13	4	13	8	12	9	15	7	16	15	17	18	15
Trogonidae	*Trogon violaceus*				1	6	9	5	9	5	9	10	8	9	10	9	12	15	12	17	10
Trogonidae	*Harpactes reinwardtii*	T		x	1	0	0x	0	0x	0	0x	0	0x	0	0x	0	0x	0	0x	0	0x
Trogonidae	*Apalharpactes mackloti*				14	16	10	18	9	21	6	22	9	21	10	25	9	41	6	31	9
Trogonidae	*Harpactes fasciatus*				1	30	1	43	1	46	1	46	0	36	1	51	1	54	2	81	0
Trogonidae	*Harpactes kasumba*				1	12	1	15	2	15	2	18	1	19	2	20	3	26	3	27	2
Trogonidae	*Harpactes diardii*				1	12	1	15	2	14	2	17	1	19	2	20	2	25	3	26	1
Trogonidae	*Harpactes ardens*				1	15	11	26	10	14	11	23	12	22	19	34	14	27	22	39	13
Trogonidae	*Harpactes whiteheadi*				1	11	0	30	2	15	2	33	0	16	0	41	2	32	2	42	0
Trogonidae	*Harpactes orrhophaeus*				1	7	2	15	2	12	3	20	3	12	2	21	3	24	3	31	3
Trogonidae	*Harpactes duvaucelii*				1	12	2	14	2	14	3	17	2	18	3	20	3	25	3	25	2
Trogonidae	*Harpactes oreskios*				1	15	8	17	8	15	9	19	9	18	11	19	12	26	14	27	12
Trogonidae	*Harpactes erythrocephalus*				1	18	5	20	4	19	5	21	4	20	6	21	6	22	7	24	6
Trogonidae	*Harpactes wardi*				1	45	5	48	4	47	7	51	3	51	9	52	6	53	7	58	3
Coraciidae	*Coracias garrulus*		H		1	0	14	0	12	0	15	0	11	0	17	0	15	0	18	0	13
Coraciidae	*Coracias abyssinica*		H		1	0	4	0	5	0	4	0	3	0	4	0	9	0	6	0	5
Coraciidae	*Coracias caudata*		H		1	0	3	0	6	0	3	0	2	0	4	0	11	0	4	0	3
Coraciidae	*Coracias spatulata*				1	41	0	33	1	42	0	42	0	42	1	34	6	42	1	43	1
Coraciidae	*Coracias naevia*		H		1	0	3	0	7	0	3	0	2	0	4	0	11	0	5	0	4
Coraciidae	*Coracias benghalensis*		H		1	0	5	0	5	0	5	0	4	0	8	0	9	0	11	0	7
Coraciidae	*Coracias temminckii*		H		1	0	16	0	11	0	15	0	14	0	17	0	21	0	24	0	24
Coraciidae	*Coracias cyanogaster*				1	26	4	13	6	32	6	44	4	38	6	19	11	48	7	47	7
Coraciidae	*Eurystomus glaucurus*				1	33	3	22	5	34	3	41	2	39	4	27	8	42	4	44	3
Coraciidae	*Eurystomus gularis*				1	33	6	22	8	32	7	53	4	49	7	32	11	57	10	62	5
Coraciidae	*Eurystomus orientalis*				1	12	8	14	6	14	8	15	6	15	11	17	10	20	12	21	8
Coraciidae	*Eurystomus azureus*	T			1	13	0x	23	0x	24	0x	23	0x	33	0x	23	0x	41	0x	37	0x
Brachypteraciida	*Brachypteracias leptosomu*	T			1	56	0	40	2	57	0	57	0	57	0	53	2	57	0	57	0
Brachypteraciida	*Brachypteracias squamiger*	T			1	65	0	42	3	67	0	67	0	67	0	62	3	67	0	67	0
Brachypteraciida	*Atelornis pittoides*				1	61	0	44	4	62	0	62	0	62	0	57	4	62	0	62	0
Brachypteraciida	*Atelornis crossleyi*				1	66	0	42	3	68	0	68	0	68	0	63	3	68	0	68	0
Brachypteraciida	*Uratelornis chimaera*	T		x	1	70	0x	8	0x	70	0x	70	0x	70	0x	55	0x	70	0x	70	0x
Leptosomidae	*Leptosomus discolor*		H		1	0	0	0	7	0	0	0	0	0	0	0	7	0	0	0	0
Momotidae	*Hylomanes momotula*				1	11	5	22	4	15	6	28	5	16	7	27	8	24	9	35	7
Momotidae	*Aspatha gularis*				1	9	0	13	0	13	3	18	0	9	0	13	8	14	3	19	0
Momotidae	*Electron platyrhynchum*				1	5	8	5	8	6	8	11	7	10	9	10	9	17	9	21	8
Momotidae	*Electron carinatum*	T			1	16	5	27	4	21	6	36	4	20	6	35	9	31	8	41	5
Momotidae	*Eumomota superciliosa*				1	6	3	20	1	19	3	26	2	10	5	22	12	21	7	31	4
Momotidae	*Baryphthengus martii*				1	5	7	5	7	5	7	9	6	9	8	10	8	12	8	17	7

Family	Species	IUCN	Ag	R	Range Source	Percentage loss in geographic range 2050								2100							
						TG		AM		GO		OS		TG		AM		GO		OS	
						H	C	H	C	H	C	H	C	H	C	H	C	H	C	H	C
Momotidae	*Baryphthengus ruficapillus*				1	16	11	15	7	21	7	24	6	24	18	25	13	36	12	37	11
Momotidae	*Momotus mexicanus*				1	10	9	24	6	11	9	34	3	13	10	24	18	18	12	36	5
Momotidae	*Momotus momota*				1	8	12	6	11	6	12	11	10	11	14	10	15	16	16	19	13
Momotidae	*Momotus aequatorialis*				1	14	1	19	1	17	1	28	1	26	2	36	3	31	2	46	2
Todidae	*Todus multicolor*				1	7	15	29	13	14	9	39	6	16	15	32	16	19	10	41	6
Todidae	*Todus angustirostris*				1	11	8	15	6	11	10	22	0	11	10	15	7	11	10	22	0
Todidae	*Todus mexicanus*			x	1	18	0x	18	0x	18	0x	18	0x	18	0x	18	0x	18	0x	18	0x
Todidae	*Todus todus*			x	1	17	0x	17	7x	11	7x	46	7x	46	0x	17	7x	11	7x	46	7x
Todidae	*Todus subulatus*				1	8	8	17	3	8	9	21	0	8	9	17	3	8	9	21	0
Alcedinidae	*Ceyx lepidus*				1	6	5	8	4	18	4	17	3	14	6	14	7	30	6	27	4
Alcedinidae	*Ceyx erithacus*				1	18	5	22	5	19	6	22	5	23	6	26	8	28	9	32	7
Alcedinidae	*Ceyx melanurus*	T			1	14	11	24	11	14	12	21	11	21	17	30	14	27	20	33	12
Alcedinidae	*Ceyx fallax*				1	14	13	26	9	19	13	15	12	16	14	29	22	28	30	23	27
Alcedinidae	*Ispidina madagascariensis*				1	54	0	25	10	55	0	55	0	55	0	34	10	55	0	55	0
Alcedinidae	*Ispidina picta*				1	31	3	21	5	32	3	40	2	38	4	26	8	41	4	42	3
Alcedinidae	*Ispidina lecontei*				1	35	5	24	6	37	5	56	3	53	6	35	8	64	6	65	4
Halcyonidae	*Lacedo pulchella*				1	12	5	15	5	14	5	17	5	17	7	20	8	24	9	25	7
Halcyonidae	*Dacelo novaeguineae*		H		1	0	20	0	12	0	18	0	8	0	25	0	18	0	24	0	10
Halcyonidae	*Dacelo leachii*				1	0	21	0	14	2	18	2	10	1	24	1	22	3	25	4	13
Halcyonidae	*Dacelo tyro*			x	1	2	31x	0	32x	6	32x	18	32x	18	31x	0	41x	27	41x	19	41x
Halcyonidae	*Dacelo gaudichaud*				1	4	3	5	3	16	2	15	1	9	5	11	6	28	4	24	3
Halcyonidae	*Clytoceyx rex*				1	6	1	7	0	20	0	14	1	11	4	14	4	33	3	28	2
Halcyonidae	*Cittura cyanotis*				1	15	27	40	14	17	26	23	16	23	27	44	17	21	36	39	23
Halcyonidae	*Pelargopsis amauropterus*				1	10	3	14	3	17	4	18	4	11	5	17	5	22	6	24	5
Halcyonidae	*Halcyon badia*				1	35	5	23	7	35	6	55	3	52	6	33	8	62	6	63	4
Halcyonidae	*Halcyon smyrnensis*		H		1	0	4	0	4	0	4	0	4	0	7	0	7	0	9	0	5
Halcyonidae	*Halcyon cyanoventris*				1	2	0	0	0	0	0	0	0	5	0	1	1	2	0	1	0
Halcyonidae	*Halcyon leucocephala*				1	24	2	15	5	25	2	29	1	27	3	18	8	29	3	30	2
Halcyonidae	*Halcyon senegalensis*				1	31	3	20	5	31	4	39	2	37	4	25	8	40	5	42	3
Halcyonidae	*Halcyon malimbica*				1	32	5	19	7	31	6	48	4	44	6	26	10	49	8	52	5
Halcyonidae	*Halcyon albiventris*				1	33	1	27	2	33	1	35	1	35	3	29	6	35	2	35	2
Halcyonidae	*Halcyon chelicuti*		H		1	0	3	0	5	0	3	0	2	0	4	0	9	0	5	0	4
Halcyonidae	*Todiramphus winchelli*	T			1	21	13	25	16	12	16	24	17	32	16	26	17	26	21	38	18
Halcyonidae	*Todiramphus diops*		H		1	0	0	0	0	0	0	0	0	0	0	0	0	0	0	0	0
Halcyonidae	*Todiramphus lazuli*			x	1	15	0x	17	0x	21	0x	20	0x	33	0x	17	0x	26	0x	43	0x
Halcyonidae	*Todiramphus macleayii*				1	2	22	3	12	7	19	6	7	3	29	4	24	9	28	11	10
Halcyonidae	*Todiramphus albonotatus*				1	7	0	7	0	51	0	46	0	24	0	27	0	72	0x	59	0
Halcyonidae	*Todiramphus leucopygius*				1	10	2x	11	0x	16	0x	26	0x	24	8x	25	0x	54	0x	45	0x
Halcyonidae	*Todiramphus farquhari*	T		x	1	14	0x	36	0x	39	0x	12	0x	17	0x	60	0x	52	0x	64	0x
Halcyonidae	*Todiramphus pyrrhopygia*				1	0	12	1	9	1	11	1	8	1	14	1	13	1	15	2	9
Halcyonidae	*Todiramphus funebris*	T		x	1	14	0x	18	0x	28	0x	24	0x	31	0x	18	0x	44	0x	33	0x
Halcyonidae	*Todiramphus chloris*				1	9	9	11	7	11	8	13	5	12	12	14	12	18	13	20	8
Halcyonidae	*Todiramphus enigma*			x	1	0	0x	0	0x	0	0x	0	0x	0	0x	0	0x	0	0x	0	0x
Halcyonidae	*Todiramphus saurophaga*				1	10	2	8	2	23	2	20	1	21	3	16	2	36	2	33	1

Family	Species	IUCN	Ag	R	Range Source	Percentage loss in geographic range 2050 TG H	TG C	AM H	AM C	GO H	GO C	OS H	OS C	2100 TG H	TG C	AM H	AM C	GO H	GO C	OS H	OS C
Halcyonidae	*Todiramphus australasia*				1	5	19	4	19	13	11	14	18	5	22	4	23	14	13	14	18
Halcyonidae	*Todiramphus sanctus*				1	1	16	1	10	2	14	2	8	2	19	2	15	3	19	4	9
Halcyonidae	*Caridonax fulgidus*				1	14	6	10	9	3	9	8	6	14	21	10	25	13	14	23	6
Halcyonidae	*Melidora macrorrhina*				1	5	3	5	2	17	1	14	1	10	5	12	6	28	4	26	2
Halcyonidae	*Actenoides bougainvillei*	T		x	1	4	0x	4	0x	11	0x	14	0x	24	0x	4	0x	44	0x	46	0x
Halcyonidae	*Actenoides concretus*				1	13	3	15	4	15	4	18	3	19	4	21	4	27	4	27	4
Halcyonidae	*Actenoides lindsayi*				1	9	10	22	6	14	11	15	7	9	20	34	14	24	22	26	9
Halcyonidae	*Actenoides hombroni*	T			1	19	14	26	18	8	18	18	19	29	17	26	18	23	22	35	19
Halcyonidae	*Actenoides monachus*				1	14	14	26	9	19	13	16	12	16	15	30	22	29	30	24	27
Halcyonidae	*Actenoides princeps*				1	15	15	27	9	17	15	19	10	18	17	31	20	24	35	26	24
Halcyonidae	*Syma torotoro*				1	4	4	5	3	15	2	14	1	9	5	12	6	25	4	22	3
Halcyonidae	*Syma megarhyncha*				1	4	1	6	0	26	0	23	1	10	8	14	10	40	8	40	4
Halcyonidae	*Tanysiptera hydrocharis*			x	1	6	31x	8	28x	42	11x	26	11x	14	31x	9	32x	57	16x	44	16x
Halcyonidae	*Tanysiptera galatea*				1	5	3	7	2	18	1	15	1	11	5	13	6	29	4	26	2
Halcyonidae	*Tanysiptera ellioti*			x	1	0	0x	0	0x	0	0x	0	0x	0	0x	0	0x	0	0x	0	0x
Halcyonidae	*Tanysiptera riedelii*			x	1	0	5x	0	5x	0	5x	0	5x	0	5x	0	5x	0	5x	0	5x
Halcyonidae	*Tanysiptera carolinae*			x	1	0	0x	0	0x	0	0x	0	0x	0	0x	0	0x	0	0x	0	0x
Halcyonidae	*Tanysiptera nympha*				1	10	3	5	4	9	4	3	2	11	3	12	4	14	4	18	2
Halcyonidae	*Tanysiptera danae*				1	0	0	0	0	30	0	33	0	9	15	12	28	51	24x	56	15
Halcyonidae	*Tanysiptera sylvia*				1	2	23	2	15	15	24	18	11	8	29	7	30	23	33	23	15
Nyctyornithidae	*Nyctyornis amictus*				1	12	3	15	4	15	4	18	3	19	4	21	4	27	5	27	4
Nyctyornithidae	*Nyctyornis athertoni*				1	23	10	28	9	24	12	28	11	27	14	31	15	30	18	36	15
Meropidae	*Meropogon forsteni*				1	16	16	34	8	15	16	18	11	19	16	36	18	20	37	30	29
Meropidae	*Merops gularis*		H		1	0	5	0	7	0	6	0	3	0	6	0	9	0	7	0	4
Meropidae	*Merops muelleri*		H		1	0	6	0	8	0	6	0	4	0	6	0	9	0	7	0	4
Meropidae	*Merops bulocki*				1	26	2	14	4	31	2	36	2	33	3	19	7	38	3	38	3
Meropidae	*Merops bullockoides*				1	38	3	27	4	37	3	42	2	40	4	30	9	41	3	42	3
Meropidae	*Merops pusillus*				1	32	3	20	7	34	3	40	2	37	4	24	10	40	4	41	3
Meropidae	*Merops variegatus*				1	39	4	26	6	38	4	53	3	50	4	34	8	55	5	58	3
Meropidae	*Merops oreobates*		H		1	0	5	0	11	0	4	0	2	0	5	0	13	0	5	0	2
Meropidae	*Merops hirundineus*		H		1	0	3	0	4	0	3	0	2	0	4	0	9	0	5	0	4
Meropidae	*Merops breweri*				1	33	7	22	10	31	8	51	4	48	7	32	11	55	9	59	5
Meropidae	*Merops revoilii*				1	30	3	8	22	35	1	35	1	33	4	10	25	35	3	35	2
Meropidae	*Merops albicollis*				1	14	3	6	6	15	2	19	1	17	3	8	9	19	6	19	4
Meropidae	*Merops orientalis*				1	11	3	9	4	11	3	14	3	13	5	11	7	14	7	18	4
Meropidae	*Merops boehmi*				1	55	0	46	0	55	0	56	0	56	1	48	2	56	0	56	0
Meropidae	*Merops viridis*				1	10	5	13	5	11	5	13	5	14	8	16	7	19	9	19	7
Meropidae	*Merops persicus*		H		1	0	6	0	6	0	7	0	6	0	7	0	10	0	12	0	9
Meropidae	*Merops superciliosus*		H		1	0	1	0	3	0	1	0	1	0	1	0	7	0	4	0	4
Meropidae	*Merops philippinus*				1	14	7	17	6	17	7	18	6	17	10	20	10	22	13	24	9
Meropidae	*Merops ornatus*				1	1	16	1	11	3	14	3	7	2	19	2	15	4	20	5	9
Meropidae	*Merops apiaster*		H		1	0	10	0	9	0	11	0	8	0	13	0	12	0	14	0	10
Meropidae	*Merops leschenaulti*				1	19	6	23	6	21	7	23	7	22	9	26	10	26	12	30	9
Meropidae	*Merops malimbicus*				1	31	6	21	7	35	6	46	3	44	7	32	11	54	8	51	5

Family	Species	IUCN	Ag	R	Range Source	Percentage loss in geographic range 2050 TG H	C	AM H	C	GO H	C	OS H	C	2100 TG H	C	AM H	C	GO H	C	OS H	C
Meropidae	*Merops nubicus*				1	22	2	12	6	25	2	28	1	27	3	15	9	29	3	29	2
Coliidae	*Colius striatus*				1	34	4	23	5	33	4	45	3	42	5	29	9	44	5	48	3
Coliidae	*Colius leucocephalus*				1	22	5	8	16	26	1	26	1	23	5	10	17	27	2	27	1
Coliidae	*Colius castanotus*				1	49	1	41	1	50	0	50	1	49	1	44	1	50	0	50	1
Coliidae	*Colius colius*				1	4	7	2	9	4	9	4	8	4	10	2	14	4	13	4	13
Coliidae	*Urocolius macrourus*				1	27	4	12	12	30	2	32	2	30	4	15	16	33	5	33	3
Coliidae	*Urocolius indicus*				1	22	3	17	4	22	3	22	3	23	5	18	9	22	6	23	5
Cuculidae	*Clamator jacobinus*		H		1	0	2	0	5	0	2	0	1	0	3	0	8	0	4	0	3
Cuculidae	*Clamator levaillantii*				1	46	2	32	3	47	2	52	1	51	3	36	6	52	3	54	2
Cuculidae	*Clamator coromandus*				1	14	5	16	5	14	6	16	6	16	7	17	7	17	9	19	8
Cuculidae	*Clamator glandarius*				1	23	4	15	6	25	3	28	2	27	5	18	10	29	6	29	4
Cuculidae	*Pachycoccyx audeberti*				1	42	2	34	3	41	2	46	1	45	3	38	6	46	3	47	2
Cuculidae	*Cuculus crassirostris*				1	18	17	22	11	13	18	23	13	20	19	26	26	23	36	29	25
Cuculidae	*Cuculus sparverioides*				1	16	4	19	4	17	5	19	5	19	6	21	6	22	7	24	6
Cuculidae	*Cuculus varius*		H		1	0	4	0	4	0	4	0	3	0	5	0	5	0	6	0	3
Cuculidae	*Cuculus vagans*				1	13	3	16	4	15	4	18	4	19	4	21	4	28	5	28	4
Cuculidae	*Cuculus fugax*				1	13	3	14	3	13	4	14	4	15	5	16	5	17	6	18	5
Cuculidae	*Cuculus pectoralis*				1	10	13	18	13	10	14	12	14	13	19	22	17	22	21	20	18
Cuculidae	*Cuculus solitarius*				1	37	4	25	5	37	4	49	3	46	5	31	9	50	6	52	4
Cuculidae	*Cuculus clamosus*				1	33	3	22	5	34	4	44	2	41	4	28	8	46	5	47	3
Cuculidae	*Cuculus micropterus*				1	19	3	21	3	20	3	22	3	21	4	23	4	24	5	28	4
Cuculidae	*Cuculus canorus*				1	6	13	6	12	7	14	7	11	8	16	8	17	10	19	11	16
Cuculidae	*Cuculus gularis*				1	31	3	20	5	32	3	38	2	36	4	24	9	39	4	40	3
Cuculidae	*Cuculus saturatus*				1	15	3	17	3	15	4	17	4	19	5	20	4	22	4	22	4
Cuculidae	*Cuculus horsfieldi*				14	4	8	4	8	4	9	4	9	5	10	5	13	5	13	6	13
Cuculidae	*Cuculus poliocephalus*				1	21	7	21	7	21	7	22	7	22	11	23	14	25	13	25	12
Cuculidae	*Cuculus rochii*				1	52	0	31	5	52	0	52	0	52	0	41	5	52	0	52	0
Cuculidae	*Cuculus pallidus*				1	0	12	1	9	1	11	1	8	1	14	1	12	2	15	2	9
Cuculidae	*Cercococcyx mechowi*				1	36	6	25	10	37	8	55	5	52	7	33	12	57	10	63	6
Cuculidae	*Cercococcyx olivinus*				1	44	8	24	13	41	9	62	5	58	8	32	14	59	10	67	6
Cuculidae	*Cercococcyx montanus*				1	42	1	37	2	43	2	47	1	46	2	40	5	45	3	47	1
Cuculidae	*Cacomantis sonneratii*		H		1	0	4	0	4	0	5	0	4	0	7	0	7	0	8	0	6
Cuculidae	*Cacomantis merulinus*				1	15	5	18	5	16	5	19	5	18	7	20	8	22	9	26	6
Cuculidae	*Cacomantis variolosus*				1	7	11	9	7	11	9	12	5	12	14	13	12	19	14	18	7
Cuculidae	*Cacomantis castaneiventris*				1	4	6	5	4	15	5	13	2	9	9	11	9	25	8	23	3
Cuculidae	*Cacomantis heinrichi*			x	1	12	0x	22	0x	24	0x	23	0x	31	0x	22	0x	42	0x	38	0x
Cuculidae	*Cacomantis flabelliformis*		H		1	0	16	0	8	0	13	0	5	0	21	0	15	0	20	0	7
Cuculidae	*Rhamphomantis megarhyn*				1	12	1	3	3	22	3	16	3	15	5	6	12	32	12	34	8
Cuculidae	*Chrysococcyx minutillus*				1	6	13	8	8	12	11	10	6	9	17	10	16	20	17	17	8
Cuculidae	*Chrysococcyx lucidus*		H		1	0	19	0	11	0	16	0	8	0	23	0	17	0	23	0	10
Cuculidae	*Chrysococcyx basalis*		H		1	0	12	0	9	0	11	0	7	0	14	0	12	0	15	0	9
Cuculidae	*Chrysococcyx ruficollis*				1	6	1	8	0	22	0	14	1	16	8	13	10	33	8	32	4
Cuculidae	*Chrysococcyx meyeri*				1	5	1	6	1	18	1	14	1	11	4	14	5	30	4	27	2
Cuculidae	*Chrysococcyx maculatus*				1	24	6	26	5	24	6	25	5	26	8	28	8	28	9	29	8

Family	Species	IUCN	Ag	R	Range Source	2050 TG H	2050 TG C	2050 AM H	2050 AM C	2050 GO H	2050 GO C	2050 OS H	2050 OS C	2100 TG H	2100 TG C	2100 AM H	2100 AM C	2100 GO H	2100 GO C	2100 OS H	2100 OS C
Cuculidae	*Chrysococcyx xanthorhync*		H		1	0	5	0	5	0	5	0	5	0	8	0	7	0	9	0	7
Cuculidae	*Chrysococcyx osculans*				1	1	12	1	9	1	11	1	7	1	14	1	12	2	14	2	8
Cuculidae	*Chrysococcyx flavigularis*				1	37	7	23	10	37	9	59	5	54	8	33	15	62	12	66	7
Cuculidae	*Chrysococcyx klaas*				1	31	3	20	5	31	3	39	2	37	4	25	8	40	5	41	3
Cuculidae	*Chrysococcyx cupreus*				1	35	4	23	6	36	4	45	2	43	5	28	10	46	5	48	3
Cuculidae	*Chrysococcyx caprius*				1	28	3	17	6	29	3	35	2	33	4	21	10	36	5	37	4
Cuculidae	*Caliechthrus leucolophus*				1	5	2	5	1	19	1	13	1	12	5	14	6	29	5	27	3
Cuculidae	*Surniculus lugubris*				1	16	4	19	4	17	5	19	4	19	6	21	7	22	8	24	6
Cuculidae	*Surniculus velutinus*				14	12	12	18	11	10	13	15	13	15	18	22	15	20	20	22	15
Cuculidae	*Microdynamis parva*				1	4	3	6	2	17	1	15	1	10	5	12	6	28	5	26	3
Cuculidae	*Eudynamys scolopacea*		H		1	0	6	0	5	0	6	0	4	0	9	0	9	0	10	0	6
Cuculidae	*Eudynamys taitensis*				1	5	20	6	16	16	16	14	13	8	25	10	23	17	24	20	18
Cuculidae	*Scythrops novaehollandiae*				1	2	20	4	13	4	17	4	9	3	23	4	20	5	24	6	12
Cuculidae	*Ceuthmochares aereus*				1	37	5	26	7	36	6	50	3	47	6	33	10	51	7	54	4
Cuculidae	*Phaenicophaeus diardi*				1	13	2	16	3	16	3	19	2	20	3	22	3	28	3	28	3
Cuculidae	*Phaenicophaeus sumatran*				1	12	2	16	3	15	3	17	2	19	3	21	3	28	3	27	2
Cuculidae	*Phaenicophaeus tristis*				1	20	8	22	7	20	9	24	8	23	10	25	10	26	13	30	11
Cuculidae	*Phaenicophaeus viridirostri*		H		1	0	3	0	3	0	2	0	1	0	4	0	5	0	6	0	1
Cuculidae	*Phaenicophaeus leschena*				1	11	5	12	5	9	6	14	4	12	8	13	10	10	12	18	6
Cuculidae	*Phaenicophaeus chloropha*				1	12	3	15	3	14	3	17	3	19	4	21	4	27	4	26	3
Cuculidae	*Phaenicophaeus javanicus*				1	13	2	15	3	15	3	18	3	19	3	21	3	27	3	27	3
Cuculidae	*Phaenicophaeus calyorhyn*				1	14	13	27	9	19	13	16	12	16	14	30	21	29	29	24	26
Cuculidae	*Phaenicophaeus curvirostri*				1	13	2	15	3	15	3	18	2	20	3	21	3	28	3	27	3
Cuculidae	*Phaenicophaeus pyrrhocep*	T			1	51	0	55	0	40	3	54	0	52	0	60	0	52	3	63	0
Cuculidae	*Phaenicophaeus supercilio*				1	7	8	20	5	12	9	15	6	7	19	33	12	24	21	26	7
Cuculidae	*Phaenicophaeus cumingi*				1	8	9	22	5	14	9	16	6	8	19	34	13	26	21	27	8
Cuculidae	*Carpococcyx radiatus*				1	8	0	12	1	11	2	13	1	15	1	17	1	20	2	22	1
Cuculidae	*Carpococcyx viridis*	T			1	17	0	18	11	17	10	29	1	18	1	28	11	54	10x	40	1
Cuculidae	*Carpococcyx renauldi*				1	10	13	15	12	13	14	14	13	13	18	15	22	18	27	22	21
Cuculidae	*Coua gigas*				1	39	1	3	12	39	1	39	1	39	1	11	12	39	1	39	1
Cuculidae	*Coua coquereli*				1	46	1	9	13	46	1	46	1	46	1	16	13	46	1	46	1
Cuculidae	*Coua serriana*				1	84	0	56	4	87	0	87	0	87	0	82	4	87	0	87	0
Cuculidae	*Coua reynaudii*				1	70	0	45	5	72	0	72	0	72	0	65	5	72	0	72	0
Cuculidae	*Coua cursor*				1	25	0	2	3	25	0	25	0	25	0	11	3	25	0	25	0
Cuculidae	*Coua ruficeps*				1	38	0	4	11	38	0	38	0	38	0	11	11	38	0	38	0
Cuculidae	*Coua cristata*				1	52	0	30	7	53	0	53	0	53	0	40	7	53	0	53	0
Cuculidae	*Coua verreauxi*			x	1	18	0x	2	0x	18	0x	18	0x	18	0x	2	0x	18	0x	18	0x
Cuculidae	*Coua caerulea*				1	68	0	43	5	70	0	70	0	70	0	63	5	70	0	70	0
Centropodidae	*Centropus milo*			x	1	17	2x	16	0x	19	0x	42	0x	29	2x	20	0x	48	0x	64	0x
Centropodidae	*Centropus goliath*				1	20	0	29	0	24	0	22	0	35	0x	30	0	41	0x	40	0x
Centropodidae	*Centropus violaceus*				1	5	0	7	0	50	0	43	0	26	0	23	0	70	0	56	0
Centropodidae	*Centropus menbeki*				1	5	3	5	2	17	1	14	1	10	5	12	6	28	5	26	2
Centropodidae	*Centropus ateralbus*				1	6	0	7	0	51	0	43	0	26	0	23	0	72	0	57	0
Centropodidae	*Centropus phasianinus*				1	1	22	1	13	4	19	3	9	2	27	2	23	6	27	6	11

Family	Species	IUCN	Ag	R	Range Source	2050 TG H	2050 TG C	2050 AM H	2050 AM C	2050 GO H	2050 GO C	2050 OS H	2050 OS C	2100 TG H	2100 TG C	2100 AM H	2100 AM C	2100 GO H	2100 GO C	2100 OS H	2100 OS C
						Percentage loss in geographic range															
Centropodidae	*Centropus spilopterus*		H	x	1	0	0x	0	0x	0	0x	0	0x	0	0x	0	0x	0	0x	0	0x
Centropodidae	*Centropus bernsteini*		H		1	0	5	0	3	0	2	0	2	0	6	0	4	0	3	0	2
Centropodidae	*Centropus chalybeus*			x	1	0	6x	0	6x	0	6x	0	6x	0	6x	0	6x	0	6x	0	6x
Centropodidae	*Centropus rectunguis*	T			1	13	2	16	3	16	3	19	2	20	3	22	3	28	3	29	2
Centropodidae	*Centropus steerii*	T		x	1	0	5x	3	1x	0	5x	41	4x	0	23x	21	2x	3	23x	41	7x
Centropodidae	*Centropus sinensis*				1	13	4	15	3	14	4	16	3	15	5	17	6	18	7	22	5
Centropodidae	*Centropus andamanensis*			x	1	10	0x	10	0x	10	0x	10	0x	10	0x	20	0x	20	0x	20	0x
Centropodidae	*Centropus nigrorufus*	T			1	6	0	0	0	0	0	0	0	8	0	5	0	6	0	5	1
Centropodidae	*Centropus viridis*				1	7	12	14	10	8	14	9	14	9	18	17	15	15	19	13	17
Centropodidae	*Centropus toulou*				1	52	0	31	5	52	0	52	0	52	0	41	5	52	0	52	0
Centropodidae	*Centropus grillii*				1	26	2	18	3	26	2	31	1	30	2	21	6	32	3	32	2
Centropodidae	*Centropus bengalensis*		H		1	0	4	0	4	0	5	0	4	0	6	0	7	0	8	0	6
Centropodidae	*Centropus chlororhynchus*	T			1	46	1	39	0	50	0	47	2	55	1	54	0	56	0	54	2
Centropodidae	*Centropus leucogaster*				1	30	5	18	9	31	6	42	4	39	5	24	10	44	7	47	4
Centropodidae	*Centropus anselli*				1	34	4	20	7	32	6	52	3	50	4	32	8	60	7	62	4
Centropodidae	*Centropus monachus*				1	37	6	22	9	38	6	56	4	52	6	32	12	57	7	62	6
Centropodidae	*Centropus cupreicaudus*				1	29	1	24	2	29	1	30	1	30	1	25	8	30	1	30	1
Centropodidae	*Centropus senegalensis*				1	17	3	10	4	20	3	24	2	23	3	13	8	25	5	25	4
Centropodidae	*Centropus superciliosus*				1	34	3	18	13	37	2	38	2	37	4	21	17	38	2	39	2
Centropodidae	*Centropus melanops*				1	20	14	27	16	12	15	26	17	31	17	27	16	27	21	40	17
Centropodidae	*Centropus celebensis*				1	14	13	27	9	19	13	17	12	16	14	30	21	28	29	24	27
Centropodidae	*Centropus unirufus*				1	7	6	17	5	12	7	13	4	7	16	25	13	22	16	20	8
Coccyzidae	*Coccyzus pumilus*				1	16	6	12	7	13	6	20	5	20	9	18	12	26	13	28	9
Coccyzidae	*Coccyzus cinereus*				1	7	11	5	10	7	11	9	9	10	16	9	16	14	16	16	14
Coccyzidae	*Coccyzus erythropthalmus*				1	11	24	17	17	16	20	18	14	15	28	23	23	27	26	27	18
Coccyzidae	*Coccyzus americanus*				1	7	18	14	11	11	14	14	9	12	21	18	18	20	22	20	13
Coccyzidae	*Coccyzus euleri*				1	7	14	5	14	6	15	11	12	10	17	9	17	18	19	20	16
Coccyzidae	*Coccyzus minor*				1	11	9	16	8	12	9	21	7	14	11	19	15	20	13	27	10
Coccyzidae	*Coccyzus melacoryphus*				1	7	11	5	10	6	11	10	9	10	14	9	14	16	14	18	12
Coccyzidae	*Coccyzus lansbergi*				1	24	3	18	6	25	6	26	4	30	11	29	15	38	21	39	8
Coccyzidae	*Hyetornis pluvialis*			x	1	16	0x	16	5x	10	5x	40	5x	40	0x	16	5x	10	5x	40	5x
Coccyzidae	*Hyetornis rufigularis*	T			1	9	9	15	6	9	10	22	0	9	11	15	7	9	10	22	0
Coccyzidae	*Piaya cayana*				1	8	11	7	10	8	11	13	9	12	14	12	14	18	15	21	12
Coccyzidae	*Piaya melanogaster*				1	3	10	2	10	3	10	5	10	6	11	5	12	8	13	11	12
Coccyzidae	*Piaya minuta*				1	6	10	4	10	5	10	9	9	10	11	8	13	15	13	16	12
Coccyzidae	*Saurothera merlini*				1	9	15x	29	15x	17	10x	42	6x	20	15x	32	17x	23	11x	45	6x
Coccyzidae	*Saurothera vetula*			x	1	18	0x	18	6x	10	6x	44	6x	44	0x	18	6x	10	6x	44	6x
Coccyzidae	*Saurothera longirostris*			x	1	9	7x	14	3x	10	7x	19	0x	9	8x	14	4x	10	7x	19	0x
Coccyzidae	*Saurothera vieilloti*			x	1	18	0x	18	0x	18	0x	18	0x	18	0x	18	0x	18	0x	18	0x
Crotophagidae	*Crotophaga major*				1	7	12	5	11	7	11	11	9	11	15	10	14	17	15	19	12
Crotophagidae	*Crotophaga ani*		H		1	0	12	0	10	0	11	0	9	0	15	0	14	0	15	0	12
Crotophagidae	*Crotophaga sulcirostris*		H		1	0	7	0	8	0	9	0	6	0	10	0	16	0	15	0	10
Crotophagidae	*Guira guira*				1	7	14	5	11	7	12	11	9	10	18	9	15	15	17	19	13
Neomorphidae	*Tapera naevia*		H		1	0	11	0	10	0	11	0	9	0	14	0	14	0	15	0	12

Family	Species	IUCN	Ag	R	Range Source	Percentage loss in geographic range 2050								2100							
						TG		AM		GO		OS		TG		AM		GO		OS	
						H	C	H	C	H	C	H	C	H	C	H	C	H	C	H	C
Neomorphidae	*Morococcyx erythropygus*		H		1	0	1	0	1	0	2	0	0	0	1	0	11	0	4	0	0
Neomorphidae	*Dromococcyx phasianellus*				1	7	12	6	11	6	12	11	10	11	14	10	14	16	15	20	12
Neomorphidae	*Dromococcyx pavoninus*				1	6	12	3	12	4	12	8	11	9	14	7	15	15	16	17	14
Neomorphidae	*Geococcyx californianus*				1	3	17	6	15	3	17	7	14	4	22	6	28	6	30	9	24
Neomorphidae	*Geococcyx velox*				1	12	3	27	2	21	3	34	1	15	4	28	14	26	5	36	2
Neomorphidae	*Neomorphus geoffroyi*				1	8	9	7	9	7	9	15	9	15	10	13	11	24	11	26	10
Neomorphidae	*Neomorphus radiolosus*	T			1	15	5	14	5	22	5	23	2	16	8	24	5	32	5	29	2
Neomorphidae	*Neomorphus rufipennis*				1	5	17	4	18	5	18	7	17	10	20	8	25	12	26	13	25
Neomorphidae	*Neomorphus pucheranii*				1	1	0	0	0	0	0	2	0	2	0	3	0	2	0	6	0
Opisthocomidae	*Opisthocomus hoazin*				1	4	10	3	9	3	10	7	9	8	10	6	11	13	12	15	11
Cacatuidae	*Probosciger aterrimus*				1	4	4	5	4	16	3	13	2	9	7	10	8	26	6	24	3
Cacatuidae	*Calyptorhynchus baudinii*				1	0	42	0	24	0	35	0	7	0	50	0	24	0	42	0	7
Cacatuidae	*Calyptorhynchus latirostris*	T			1	0	13	0	9	0	12	0	4	0	16	0	11	0	16	0	4
Cacatuidae	*Calyptorhynchus funereus*				1	4	25	6	13	10	18	10	10	8	34	8	25	13	30	17	13
Cacatuidae	*Calyptorhynchus banksii*				1	0	18	0	11	1	16	1	8	0	22	0	19	1	22	2	10
Cacatuidae	*Calyptorhynchus lathami*				1	8	14	12	9	16	9	18	6	11	20	14	15	22	17	30	9
Cacatuidae	*Callocephalon fimbriatum*				1	9	11	13	2	17	6	22	2	19	16	18	3	27	7	31	3
Cacatuidae	*Cacatua roseicapilla*				1	1	17	2	11	3	15	3	8	2	21	2	17	4	21	5	9
Cacatuidae	*Cacatua leadbeateri*				1	0	7	0	6	0	7	0	6	0	7	0	7	0	9	0	7
Cacatuidae	*Cacatua sulphurea*	T	H		1	0	13	0	10	0	12	0	12	0	16	0	21	0	24	0	21
Cacatuidae	*Cacatua galerita*		H		1	0	17	0	10	0	14	0	7	0	20	0	16	0	20	0	9
Cacatuidae	*Cacatua ophthalmica*				1	6	0	7	0	52	0	44	0	26	0	24	0	73	0	57	0
Cacatuidae	*Cacatua moluccensis*	T		x	1	15	0x	18	0x	21	0x	19	0x	33	0x	18	0x	25	0x	42	0x
Cacatuidae	*Cacatua alba*	T		x	1	12	0x	22	0x	24	0x	23	0x	32	0x	22	0x	42	0x	38	0x
Cacatuidae	*Cacatua haematuropygia*	T	H		1	0	32	0	34	0	32	0	38	0	37	0	37	0	42	0	45
Cacatuidae	*Cacatua goffini*			x	1	7	22x	7	22x	29	0x	56	29x	7	22x	7	22x	42	0x	56	29x
Cacatuidae	*Cacatua sanguinea*		H		1	0	11	0	9	0	10	0	8	0	11	0	11	0	12	0	10
Cacatuidae	*Cacatua pastinator*		H		1	0	11	0	6	0	8	0	3	0	11	0	6	0	11	0	3
Cacatuidae	*Cacatua tenuirostris*		H		1	0	21	0	7	0	17	0	0	0	26	0	7	0	21	0	0
Cacatuidae	*Cacatua ducorpsii*				1	13	5	13	1	23	1	26	1	28	10	23	1	52	2	41	1
Cacatuidae	*Nymphicus hollandicus*		H		1	0	10	0	9	0	10	0	8	0	11	0	11	0	12	0	9
Psittacidae	*Chalcopsitta atra*				1	16	1	4	4	6	4	1	4	18	1	7	4	11	4	18	4
Psittacidae	*Chalcopsitta duivenbodei*				1	3	4	3	4	20	4	16	0	13	5	14	4	36	4	32	0
Psittacidae	*Chalcopsitta sintillata*				1	0	5	3	5	10	3	14	2	3	9	7	12	18	10	19	4
Psittacidae	*Chalcopsitta cardinalis*				1	17	5	12	1	23	1	28	1	31	9	21	1	49	2	44	1
Psittacidae	*Eos histrio*	T		x	1	0	0x	0	0x	0	0x	0	0x	0	0x	0	0x	0	0x	0	0x
Psittacidae	*Eos squamata*				1	17	0	23	0	19	0	17	0	29	0	26	0	32	0	30	0
Psittacidae	*Eos bornea*				1	13	0	15	0	17	0	20	0	28	0x	15	0	23	0	35	0x
Psittacidae	*Eos reticulata*			x	1	10	10x	7	10x	19	1x	65	17x	10	10x	7	10x	26	1x	65	17x
Psittacidae	*Eos cyanogenia*	T		x	1	0	7x	0	7x	0	7x	0	7x	0	7x	0	7x	0	7x	0	7x
Psittacidae	*Eos semilarvata*			x	1	11	0x	14	0x	22	0x	19	0x	31	0x	14	0x	26	0x	41	0x
Psittacidae	*Pseudeos fuscata*				1	4	3	6	2	19	2	14	1	11	5	12	6	31	5	26	3
Psittacidae	*Trichoglossus ornatus*				1	14	13	25	10	17	13	17	13	16	14	28	20	26	27	23	25
Psittacidae	*Trichoglossus haematodus*				1	3	16	5	9	10	14	9	5	7	21	8	17	16	20	16	7

Family	Species	IUCN	Ag	R	Range Source	Percentage loss in geographic range															
						2050								2100							
						TG		AM		GO		OS		TG		AM		GO		OS	
						H	C	H	C	H	C	H	C	H	C	H	C	H	C	H	C
Psittacidae	*Trichoglossus euteles*			x	1	7	24x	10	25x	14	15x	3	22x	7	24x	10	25x	14	15x	3	22x
Psittacidae	*Trichoglossus flavoviridis*				1	11	3	25	2	23	4	13	4	11	5	28	21	33	30	15	26
Psittacidae	*Trichoglossus johnstoniae*			x	1	32	12x	31	13x	15	13x	31	13x	32	12x	31	13x	20	13x	31	13x
Psittacidae	*Trichoglossus chlorolepidot*				1	4	24	6	14	8	20	8	9	6	32	7	25	11	30	14	12
Psittacidae	*Psitteuteles versicolor*				1	0	14	0	9	0	12	0	5	0	14	0	13	0	15	0	7
Psittacidae	*Psitteuteles iris*				1	2	20	3	20	12	11	2	18	2	20	3	20	12	11	2	18
Psittacidae	*Psitteuteles goldiei*				1	7	0	8	0	17	0	18	0	13	6	17	6	30	6	31	2
Psittacidae	*Lorius garrulus*	T			1	20	0	29	0	24	0	22	0	35	0	30	0	41	0x	38	0x
Psittacidae	*Lorius domicella*	T		x	1	14	0x	17	0x	21	0x	19	0x	33	0x	17	0x	26	0x	43	0x
Psittacidae	*Lorius lory*				1	5	3	5	3	18	3	15	1	10	6	11	9	29	8	28	3
Psittacidae	*Lorius hypoinochrous*				1	3	0	4	0	40	0	41	0	17	7	17	14	62	11	56	7
Psittacidae	*Lorius albidinuchus*			x	1	0	0x	13	0x	62	0x	49	0x	49	0x	18	0x	95	0x	67	0x
Psittacidae	*Lorius chlorocercus*			x	1	12	3x	11	3x	35	0x	35	3x	36	3x	11	3x	55	3x	59	3x
Psittacidae	*Phigys solitarius*			x	1	21	5x	19	5x	27	0x	41	0x	22	5x	41	5x	80	0x	79	0x
Psittacidae	*Vini australis*			x	1	0	0x	0	0x	2	0x	0	0x	52	0x	0	0x	2	0x	2	0x
Psittacidae	*Glossopsitta concinna*				1	8	13	12	5	15	9	16	4	16	16	16	5	22	10	26	4
Psittacidae	*Glossopsitta pusilla*				1	5	18	8	9	12	13	13	6	10	27	10	19	16	24	21	9
Psittacidae	*Glossopsitta porphyroceph*				1	1	13	1	9	2	13	2	7	4	15	2	12	3	17	3	8
Psittacidae	*Charmosyna palmarum*	T		x	1	6	0x	21	0x	31	0x	8	0x	20	0x	38	0x	44	0x	43	0x
Psittacidae	*Charmosyna rubrigularis*				1	6	0	8	0	51	0	43	0	26	0	23	0	71	0	56	0
Psittacidae	*Charmosyna meeki*				1	11	6x	10	2x	20	1x	19	2x	28	6x	12	2x	46	3x	40	2x
Psittacidae	*Charmosyna toxopei*	T		x	1	9	0x	7	0x	9	0x	12	0x	16	0x	7	0x	21	0x	12	0x
Psittacidae	*Charmosyna multistriata*				1	9	0	5	0	18	0	20	0	13	5	23	0	28	0	26	0
Psittacidae	*Charmosyna wilhelminae*				1	7	1	7	0	20	0	14	1	13	6	16	7	31	6	30	3
Psittacidae	*Charmosyna rubronotata*				1	7	3	4	3	8	3	7	2	13	3	13	3	15	3	18	2
Psittacidae	*Charmosyna placentis*				1	5	3	6	3	20	2	16	1	12	5	12	6	31	5	27	3
Psittacidae	*Charmosyna diadema*	T		x	1	13	28x	44	8x	22	18x	16	16x	13	28x	55	10x	24	20x	19	16x
Psittacidae	*Charmosyna amabilis*	T		x	1	21	5x	18	5x	27	0x	41	0x	23	5x	40	5x	78	0x	76	0x
Psittacidae	*Charmosyna margarethae*				1	16	5	14	1	24	1	30	1	30	8	23	1	51	2	47	1
Psittacidae	*Charmosyna pulchella*				1	7	1	3	0	15	0	16	1	11	7	13	8	30	7	30	3
Psittacidae	*Charmosyna josefinae*				1	9	1	4	0	13	0	16	2	13	4	16	2	26	0	27	2
Psittacidae	*Charmosyna papou*				1	9	0	5	0	17	0	18	0	13	6	13	7	32	7	33	2
Psittacidae	*Oreopsittacus arfaki*				1	7	1	8	1	18	1	13	2	12	6	18	11	32	10	27	5
Psittacidae	*Neopsittacus musschenbro*				1	8	1	7	0	20	0	17	1	14	6	15	7	32	6	32	3
Psittacidae	*Neopsittacus pullicauda*				1	6	1	8	0	21	0	18	1	13	6	16	7	34	6	33	3
Psittacidae	*Nestor notabilis*	T			1	0	29	0	21	3	24	0	21	2	38	0	34	3	38	2	32
Psittacidae	*Nestor meridionalis*	T			1	7	23	7	22	17	20	16	15	8	31	11	32	17	35	23	24
Psittacidae	*Micropsitta keiensis*				1	6	2	3	3	6	3	10	2	8	2	9	3	16	3	17	2
Psittacidae	*Micropsitta geelvinkiana*			x	1	0	5x	0	5x	0	5x	0	5x	0	5x	0	5x	0	5x	0	5x
Psittacidae	*Micropsitta pusio*				1	2	2	4	1	29	1	22	0	14	4	13	6	42	5	38	2
Psittacidae	*Micropsitta meeki*			x	1	25	0x	0	0x	55	0x	75	0x	75	0x	0	0x	81	0x	75	0x
Psittacidae	*Micropsitta finschii*				1	13	4	12	1	28	1	30	1	32	7	20	1	53	2	45	1
Psittacidae	*Micropsitta bruijnii*				1	9	2	8	1	21	1	19	1	16	6	16	6	34	6	34	2
Psittacidae	*Cyclopsitta gulielmitertii*				1	4	5	4	4	18	3	18	2	10	7	11	8	33	6	29	4

Family	Species	IUCN	Ag	R	Range Source	Percentage loss in geographic range 2050								2100							
						TG		AM		GO		OS		TG		AM		GO		OS	
						H	C	H	C	H	C	H	C	H	C	H	C	H	C	H	C
Psittacidae	*Cyclopsitta diophthalma*		H		1	0	4	0	3	0	3	0	2	0	6	0	6	0	5	0	4
Psittacidae	*Psittaculirostris desmarestii*				1	6	0	3	1	16	1	13	1	8	2	8	4	30	4	22	1
Psittacidae	*Psittaculirostris edwardsii*				1	3	4	5	2	38	2	15	0	15	5	12	2	53	2	32	0
Psittacidae	*Psittaculirostris salvadorii*	T			1	3	2	6	0	4	0	9	2	14	2	19	2	10	0	22	2
Psittacidae	*Bolbopsittacus lunulatus*		H		1	0	11	0	12	0	12	0	13	0	17	0	16	0	19	0	15
Psittacidae	*Psittinus cyanurus*		H		1	0	3	0	3	0	3	0	3	0	4	0	4	0	4	0	3
Psittacidae	*Psittacella brehmii*				1	9	1	8	0	18	0	16	1	13	6	17	7	31	6	32	3
Psittacidae	*Psittacella picta*				1	7	0	5	0	18	0	17	0	11	8	16	8	33	8	30	3
Psittacidae	*Psittacella modesta*				1	12	1	11	1	16	1	17	2	18	5	22	2	25	1	30	2
Psittacidae	*Psittacella madaraszi*				1	9	0	5	0	21	0	20	0	13	8	11	9	38	9	36	3
Psittacidae	*Geoffroyus geoffroyi*		H		1	0	5	0	5	0	3	0	3	0	8	0	9	0	6	0	4
Psittacidae	*Geoffroyus simplex*				1	5	2	7	1	17	1	11	1	11	5	13	8	27	7	24	3
Psittacidae	*Geoffroyus heteroclitus*				1	10	2	10	1	38	0	37	1	28	4	23	1	61	1	51	1
Psittacidae	*Prioniturus montanus*				1	8	10	23	5x	13	11	19	6	8	20x	38	12x	25	26x	33	8x
Psittacidae	*Prioniturus waterstradti*			x	1	28	7x	25	10x	25	10x	25	10x	28	7x	25	10x	35	10x	25	10x
Psittacidae	*Prioniturus platenae*	T		x	1	22	25x	21	29x	22	19x	27	18x	22	25x	21	49x	45	40x	38	46x
Psittacidae	*Prioniturus luconensis*	T			1	8	9	21	5	13	9	15	6	8	19	33	13	24	22	26	8
Psittacidae	*Prioniturus discurus*				1	15	12	24	11	13	12	22	12	21	18	30	14	25	21	34	13
Psittacidae	*Prioniturus verticalis*	T		x	1	100	0x	25	3x	37	0x	25	0x	100	0x	37	3x	37	3x	25	3x
Psittacidae	*Prioniturus flavicans*				1	21	29	21	18	1	30	24	22	26	30	23	29	9	40	36	33
Psittacidae	*Prioniturus platurus*		H		1	0	13	0	9	0	13	0	13	0	14	0	20	0	26	0	24
Psittacidae	*Prioniturus mada*			x	1	9	0x	7	0x	9	0x	12	0x	16	0x	7	0x	21	0x	12	0x
Psittacidae	*Tanygnathus megalorynch*		H		1	0	2	0	3	0	3	0	4	0	4	0	5	0	4	0	4
Psittacidae	*Tanygnathus lucionensis*		H		1	0	13	0	13	0	14	0	14	0	18	0	17	0	21	0	17
Psittacidae	*Tanygnathus sumatranus*				1	16	13	27	10	17	12	18	12	20	15	30	19	27	27	28	23
Psittacidae	*Tanygnathus gramineus*	T		x	1	9	0x	7	0x	9	0x	12	0x	16	0x	7	0x	21	0x	12	0x
Psittacidae	*Eclectus roratus*				1	4	3	7	2	19	2	15	1	12	5	14	6	30	5	26	3
Psittacidae	*Psittrichas fulgidus*	T			1	5	2	7	2	19	2	17	1	11	7	15	9	29	8	31	3
Psittacidae	*Prosopeia splendens*	T	H	x	1	0	40x	0	40x	0	40x	0	40x	0	40x	0	40x	0	40x	0	40x
Psittacidae	*Prosopeia personata*	T	H	x	1	0	0x	0	0x	0	0x	0	0x	0	0x	0	0x	0	0x	0	0x
Psittacidae	*Prosopeia tabuensis*		H	x	1	0	16x	0	16x	0	0x	0	0x	0	16x	0	16x	0	0x	0	0x
Psittacidae	*Alisterus scapularis*		H		1	0	17	0	8	0	11	0	5	0	27	0	21	0	24	0	9
Psittacidae	*Alisterus amboinensis*				1	12	1	8	2	10	2	7	2	19	1	14	2	15	3	20	3
Psittacidae	*Alisterus chloropterus*				1	2	2	4	1	18	1	14	1	8	4	11	6	30	5	25	2
Psittacidae	*Aprosmictus jonquillaceus*			x	1	4	27x	6	27x	17	16x	3	24x	4	27x	6	27x	17	16x	3	24x
Psittacidae	*Aprosmictus erythropterus*		H		1	0	22	0	15	0	19	0	11	0	26	0	22	0	26	0	13
Psittacidae	*Polytelis swainsonii*	T			1	0	0	0	0	0	0	0	0	1	3	0	0	0	4	0	0
Psittacidae	*Polytelis anthopeplus*		H		1	0	15	0	10	0	15	0	3	0	15	0	11	0	17	0	3
Psittacidae	*Polytelis alexandrae*				1	0	5	0	5	0	5	0	5	0	5	0	6	0	6	0	6
Psittacidae	*Purpureicephalus spurius*		H		1	0	20	0	12	0	17	0	4	0	23	0	12	0	20	0	4
Psittacidae	*Barnardius zonarius*		H		1	0	6	0	6	0	6	0	5	0	6	0	7	0	7	0	6
Psittacidae	*Barnardius barnardi*				14	0	16	0	13	0	15	0	11	0	18	0	15	0	18	0	12
Psittacidae	*Platycercus caledonicus*		H		1	0	43	0	13	0	36	0	13	0	44	0	13	0	36	0	13
Psittacidae	*Platycercus elegans*		H		1	0	13	0	5	0	10	0	4	0	18	0	7	0	12	0	4

Family	Species	IUCN	Ag	R	Range Source	Percentage loss in geographic range 2050								2100							
						TG		AM		GO		OS		TG		AM		GO		OS	
						H	C	H	C	H	C	H	C	H	C	H	C	H	C	H	C
Psittacidae	*Platycercus adelaidae*				14	0	22	0	19	0	22	0	13	0	22	0	19	0	22	4	13
Psittacidae	*Platycercus flaveolus*				14	0	6	0	4	0	6	0	1	0	6	0	6	0	10	0	4
Psittacidae	*Platycercus venustus*		H		1	0	15	0	10	0	14	0	5	0	16	0	13	0	18	0	7
Psittacidae	*Platycercus adscitus*		H		1	0	31	0	19	0	26	0	14	0	37	0	32	0	37	0	17
Psittacidae	*Platycercus eximius*		H		1	0	12	0	5	0	10	0	3	0	16	0	6	0	13	0	4
Psittacidae	*Platycercus icterotis*		H		1	0	11	0	8	0	11	0	4	0	13	0	9	0	14	0	4
Psittacidae	*Northiella haematogaster*		H		1	0	10	0	9	0	10	0	7	0	12	0	10	0	13	0	8
Psittacidae	*Psephotus haematonotus*		H		1	0	11	0	8	0	10	0	6	0	15	0	10	0	13	0	7
Psittacidae	*Psephotus varius*				1	0	3	0	3	0	4	0	4	0	4	0	5	0	6	0	4
Psittacidae	*Psephotus dissimilis*				1	0	8	0	7	0	8	0	0	0	8	0	8	0	8	0	0
Psittacidae	*Psephotus chrysopterygius*	T			1	0	38	0	19	0	37	0	12	0	38	0	30	0	38	0	12
Psittacidae	*Cyanoramphus novaezelan*				1	4	16	6	13	10	14	9	14	6	19	10	18	10	20	11	17
Psittacidae	*Cyanoramphus auriceps*				1	2	28	3	22	13	23	13	16	3	36	6	33	13	37	18	25
Psittacidae	*Cyanoramphus forbesi*	T		x	14	0	0x	0	0x	0	0x	0	0x	0	0x	0	0x	0	0x	0	0x
Psittacidae	*Cyanoramphus malherbi*	T		x	14	0	46x	0	37x	18	37x	0	37x	0	64x	0	55x	18	37x	0	55x
Psittacidae	*Eunymphicus cornutus*	T		x	1	13	32x	39	8x	21	21x	15	19x	13	32x	51	13x	22	25x	20	19x
Psittacidae	*Neopsephotus bourkii*				1	0	2	0	2	0	2	0	3	0	2	0	2	0	3	0	3
Psittacidae	*Neophema chrysostoma*		H		1	0	24	0	12	0	20	0	7	0	26	0	12	0	22	0	7
Psittacidae	*Neophema elegans*				1	0	12	0	8	0	11	0	5	1	13	0	8	1	12	1	5
Psittacidae	*Neophema petrophila*				1	0	25	0	17	0	23	1	10	1	26	0	17	0	23	1	10
Psittacidae	*Neophema chrysogaster*	T			1	0	25	0	25	9	25	0	25	0	25	7	25	9	25	13	25
Psittacidae	*Neophema pulchella*		H		1	0	4	0	3	0	3	0	3	0	10	0	6	0	9	0	4
Psittacidae	*Neophema splendida*				1	0	1	0	1	0	1	0	1	0	1	0	2	0	2	0	2
Psittacidae	*Lathamus discolor*	T			1	0	43	0	13	5	36	0	13	2	44	3	13	8	36	7	13
Psittacidae	*Melopsittacus undulatus*		H		1	0	10	0	9	0	9	0	8	0	12	0	11	0	13	0	9
Psittacidae	*Pezoporus wallicus*				1	2	21	6	5	10	17	8	5	10	24	7	9	15	21	14	7
Psittacidae	*Pezoporus occidentalis*	T			1	0	8	0	8	0	8	0	7	0	9	0	9	0	11	0	10
Psittacidae	*Strigops habroptilus*	T		x	1	0	0x	0	0x	0	0x	0	0x	0	0x	0	0x	0	0x	0	0x
Psittacidae	*Coracopsis vasa*				1	54	0	28	6	54	0	54	0	54	0	39	6	54	0	54	0
Psittacidae	*Coracopsis nigra*				1	51	0	27	6	51	0	51	0	51	0	37	6	51	0	51	0
Psittacidae	*Psittacus erithacus*				1	34	5	22	7	31	6	51	4	47	5	31	8	54	7	57	4
Psittacidae	*Poicephalus robustus*		H		1	0	1	0	2	0	1	0	1	0	2	0	7	0	2	0	2
Psittacidae	*Poicephalus gulielmi*				1	33	6	21	9	32	7	58	3	48	6	29	10	56	7	65	3
Psittacidae	*Poicephalus senegalus*		H		1	0	3	0	4	0	3	0	2	0	3	0	6	0	5	0	4
Psittacidae	*Poicephalus crassus*				1	32	9	10	13	28	15	54	9	50	14	17	29	49	23	59	15
Psittacidae	*Poicephalus meyeri*		H		1	0	2	0	5	0	2	0	2	0	3	0	9	0	3	0	3
Psittacidae	*Poicephalus flavifrons*				1	35	0	22	1	38	0	38	0	38	0	29	4	38	1	38	1
Psittacidae	*Poicephalus rufiventris*				1	39	3	11	26	45	1	46	0	43	3	14	29	45	3	46	2
Psittacidae	*Poicephalus cryptoxanthus*				1	39	1	34	1	39	1	39	1	39	2	36	4	40	1	40	1
Psittacidae	*Poicephalus rueppellii*				1	12	2	5	3	10	2	10	2	12	6	5	10	10	4	10	4
Psittacidae	*Agapornis canus*		H		1	0	0	0	6	0	0	0	0	0	0	0	6	0	0	0	0
Psittacidae	*Agapornis pullarius*		H		1	0	6	0	10	0	7	0	4	0	7	0	15	0	10	0	6
Psittacidae	*Agapornis taranta*			x	1	55	2x	23	3x	56	2x	56	2x	56	2x	29	16x	56	2x	56	2x
Psittacidae	*Agapornis swindernianus*				1	33	4	21	7	35	5	56	2	51	4	34	7	64	6	67	3

Family	Species	IUCN	Ag	R	Range Source	2050 TG H	2050 TG C	2050 AM H	2050 AM C	2050 GO H	2050 GO C	2050 OS H	2050 OS C	2100 TG H	2100 TG C	2100 AM H	2100 AM C	2100 GO H	2100 GO C	2100 OS H	2100 OS C
Psittacidae	*Agapornis roseicollis*				1	8	6	4	6	7	5	6	6	8	8	4	13	7	9	6	10
Psittacidae	*Agapornis fischeri*		H		1	0	7	0	11	0	6	0	7	0	9	0	12	0	6	0	9
Psittacidae	*Agapornis personatus*				1	52	2	40	4	52	2	52	2	52	3	48	4	53	2	52	2
Psittacidae	*Agapornis lilianae*				1	47	0	40	2	47	0	47	0	47	4	42	10	47	2	47	2
Psittacidae	*Agapornis nigrigenis*	T		x	1	26	0x	26	0x	26	0x	26	0x	26	0x	26	0x	26	0x	26	0x
Psittacidae	*Loriculus vernalis*		H		1	0	4	0	5	0	6	0	5	0	7	0	8	0	11	0	8
Psittacidae	*Loriculus beryllinus*				1	52	1	49	0	47	4	56	2	57	1	59	0	57	4	63	2
Psittacidae	*Loriculus philippensis*		H		1	0	12	0	12	0	13	0	13	0	18	0	15	0	20	0	16
Psittacidae	*Loriculus galgulus*		H		1	0	3	0	3	0	3	0	3	0	4	0	4	0	4	0	3
Psittacidae	*Loriculus stigmatus*				1	14	13	24	9	17	12	17	12	15	14	27	21	26	26	23	24
Psittacidae	*Loriculus amabilis*				1	18	0	28	0x	22	0	21	0	34	0x	28	0x	40	0x	39	0x
Psittacidae	*Loriculus sclateri*			x	14	33	11x	33	11x	13	31x	2	31x	33	15x	33	15x	13	36x	2	36x
Psittacidae	*Loriculus catamene*	T		x	1	0	0x	0	0x	0	0x	0	0x	0	0x	0	0x	0	0x	0	0x
Psittacidae	*Loriculus aurantiifrons*				1	4	3	6	3	16	1	14	1	10	5	13	8	27	5	25	3
Psittacidae	*Loriculus tener*				1	6	0	7	0	50	0	43	0	26	0	23	0	71	0	56	0
Psittacidae	*Loriculus exilis*				1	14	14	24	10	18	13	16	13	16	15	27	22	27	28	23	26
Psittacidae	*Loriculus pusillus*				1	6	0	0	0	0	0	0	0	7	0	4	0	10	0	4	0
Psittacidae	*Loriculus flosculus*	T		x	1	17	8x	25	14x	7	14x	0	14x	17	8x	25	14x	7	14x	27	14x
Psittacidae	*Psittacula eupatria*		H		1	0	3	0	3	0	3	0	2	0	6	0	7	0	9	0	4
Psittacidae	*Psittacula krameri*		H		1	0	3	0	3	0	3	0	2	0	4	0	7	0	6	0	3
Psittacidae	*Psittacula echo*	T		x	1	0	0x	0	0x	0	0x	0	0x	0	0x	0	0x	0	0x	0	0x
Psittacidae	*Psittacula himalayana*				1	64	6	66	4	64	3	74	0	68	8	71	7	72	5	77	2
Psittacidae	*Psittacula finschii*		H		1	0	6	0	5	0	6	0	6	0	11	0	9	0	14	0	8
Psittacidae	*Psittacula intermedia*			x	1	0	0x	0	0x	0	0x	0	0x	0	0x	0	0x	0	0x	0	0x
Psittacidae	*Psittacula cyanocephala*		H		1	0	2	0	2	0	1	0	1	0	3	0	4	0	4	0	1
Psittacidae	*Psittacula roseata*		H		1	0	6	0	6	0	7	0	6	0	12	0	13	0	17	0	11
Psittacidae	*Psittacula columboides*				1	31	1	58	0	45	0	53	0	33	2	63	0	54	0	94	0
Psittacidae	*Psittacula calthropae*			x	1	51	0x	41	0x	42	0x	42	0x	52	0x	52	0x	52	0x	52	0x
Psittacidae	*Psittacula derbiana*				1	43	3	45	2	47	2	47	2	48	3	47	2	49	2	49	2
Psittacidae	*Psittacula alexandri*				1	12	8	15	8	13	9	15	9	14	11	17	14	18	16	21	13
Psittacidae	*Psittacula caniceps*			x	1	27	0x	87	0x	77	0x	39	0x	27	0x	100	0x	100	0x	100	0x
Psittacidae	*Psittacula longicauda*				1	13	2	16	3	16	2	18	2	21	3	23	3	29	3	28	2
Psittacidae	*Anodorhynchus hyacinthin*	T			1	7	16	3	10	2	14	12	7	10	21	7	12	10	17	22	9
Psittacidae	*Anodorhynchus leari*	T		x	1	0	0x	0	0x	0	0x	1	0x	0	0x	0	0x	0	0x	1	0x
Psittacidae	*Cyanopsitta spixii*	T		x	1	10	0x	75	0x	8	0x	18	0x	10	67x	75	0x	8	67x	18	67x
Psittacidae	*Ara ararauna*				1	5	14	3	12	3	13	8	11	9	16	6	15	12	17	15	13
Psittacidae	*Ara glaucogularis*	T		x	1	6	11x	6	11x	0	11x	0	11x	6	11x	6	11x	6	11x	0	11x
Psittacidae	*Ara militaris*	T			1	11	9	22	5	14	8	28	6	16	11	25	13	21	14	33	9
Psittacidae	*Ara ambigua*	T	H		1	0	5	0	9	0	9	0	6	0	6	0	10	0	12	0	7
Psittacidae	*Ara macao*		H		1	0	10	0	10	0	11	0	10	0	11	0	13	0	14	0	12
Psittacidae	*Ara chloropterus*				1	5	12	3	12	4	13	8	12	9	14	6	16	14	17	15	15
Psittacidae	*Ara rubrogenys*	T			1	44	0	46	0	44	0	42	0	48	0	55	0x	48	0	51	0
Psittacidae	*Ara severa*				1	5	11	3	11	4	11	9	10	9	13	6	13	14	14	17	12
Psittacidae	*Ara manilata*				1	5	9	3	8	4	8	9	7	9	9	7	10	17	11	19	9

Family	Species	IUCN	Ag	R	Range Source	2050 TG H	2050 TG C	2050 AM H	2050 AM C	2050 GO H	2050 GO C	2050 OS H	2050 OS C	2100 TG H	2100 TG C	2100 AM H	2100 AM C	2100 GO H	2100 GO C	2100 OS H	2100 OS C
Psittacidae	*Ara couloni*				1	0	6	1	6	1	6	3	6	8	7	2	8	6	8	3	7
Psittacidae	*Ara maracana*				1	8	15	4	14	5	15	14	11	12	19	8	17	21	19	27	14
Psittacidae	*Ara auricollis*				1	6	20	3	21	4	24	8	20	10	27	7	32	14	35	15	31
Psittacidae	*Ara nobilis*				1	5	16	3	13	3	15	8	11	8	19	6	16	12	18	16	14
Psittacidae	*Aratinga acuticaudata*		H		1	0	8	0	6	0	7	0	5	0	11	0	9	0	11	0	8
Psittacidae	*Aratinga guarouba*	T			1	18	7	10	7	14	6	17	6	29	8	19	9	52	8	41	8
Psittacidae	*Aratinga holochlora*		H		1	0	6	0	4	0	4	0	2	0	7	0	25	0	8	0	4
Psittacidae	*Aratinga brevipes*	T		x	1	0	0x	0	0x	0	0x	0	0x	0	0x	0	0x	0	0x	0	0x
Psittacidae	*Aratinga rubritorques*			x	1	2	0x	18	0x	42	0x	24	0x	2	0x	18	0x	44	3x	24	0x
Psittacidae	*Aratinga wagleri*		H		1	0	4	0	3	0	4	0	2	0	5	0	6	0	7	0	4
Psittacidae	*Aratinga mitrata*				1	14	14	11	11	10	10	15	10	17	16	16	15	22	15	24	13
Psittacidae	*Aratinga erythrogenys*		H		1	0	8	0	7	0	12	0	9	0	8	0	10	0	15	0	11
Psittacidae	*Aratinga finschi*		H		1	0	5	0	8	0	11	0	8	0	5	0	15	0	14	0	8
Psittacidae	*Aratinga leucophthalmus*		H		1	0	13	0	11	0	12	0	9	0	16	0	14	0	15	0	12
Psittacidae	*Aratinga euops*	T			1	8	6	24	6	11	5	28	3	14	6	26	8	16	6	32	3
Psittacidae	*Aratinga chloroptera*	T		x	1	0	0x	0	0x	0	0x	0	0x	0	0x	0	0x	0	0x	0	0x
Psittacidae	*Aratinga solstitialis*				1	6	13	4	13	5	12	9	11	10	14	8	15	18	15	16	14
Psittacidae	*Aratinga jandaya*				1	6	19	3	20	5	20	12	15	9	22	5	24	15	27	24	20
Psittacidae	*Aratinga auricapilla*				1	16	11	14	7	19	6	23	6	20	20	22	15	39	12	38	13
Psittacidae	*Aratinga weddellii*				1	6	8	2	8	4	8	6	8	10	9	9	12	11	11	15	11
Psittacidae	*Aratinga nana*				1	11	5	23	3	18	5	32	4	15	6	29	12	24	9	38	6
Psittacidae	*Aratinga canicularis*				1	9	3	17	2	13	3	27	1	11	5	17	16	18	7	29	1
Psittacidae	*Aratinga aurea*		H		1	0	19	0	14	0	17	0	11	0	24	0	18	0	23	0	15
Psittacidae	*Aratinga pertinax*		H		1	0	10	0	11	0	10	0	10	0	12	0	14	0	15	0	14
Psittacidae	*Aratinga cactorum*				1	9	19	6	15	6	16	15	7	10	24	11	16	11	19	20	8
Psittacidae	*Nandayus nenday*		H		1	0	8	0	7	0	7	0	7	0	10	0	13	0	15	0	14
Psittacidae	*Leptosittaca branickii*	T			1	15	2	17	2	18	2	32	1	26	2	32	4	34	2	47	2
Psittacidae	*Ognorhynchus icterotis*	T			1	16	4	15	2	20	3	28	2	19	6	28	4	36	3	32	4
Psittacidae	*Rhynchopsitta pachyrhync*	T			1	21	11	46	5	26	10	58	5	28	14	48	11	33	19	65	9
Psittacidae	*Rhynchopsitta terrisi*	T		x	1	0	37x	20	9x	0	9x	0	9x	0	37x	20	39x	0	9x	0	9x
Psittacidae	*Cyanoliseus patagonus*		H		1	0	4	0	4	0	5	0	4	0	5	0	8	0	10	0	9
Psittacidae	*Pyrrhura cruentata*	T			1	17	5	14	2	30	1	22	2	22	6	17	9	45	6	34	9
Psittacidae	*Pyrrhura devillei*				1	6	7	5	7	4	7	16	7	15	7	19	10	25	10	35	10
Psittacidae	*Pyrrhura frontalis*				1	18	9	19	6	24	6	29	6	27	13	30	10	42	8	43	10
Psittacidae	*Pyrrhura lepida*				1	6	10	3	10	9	10	18	9	14	12	6	15	40	15	41	12
Psittacidae	*Pyrrhura perlata*				1	3	20	4	19	2	19	7	18	9	20	6	20	10	21	12	19
Psittacidae	*Pyrrhura molinae*				1	6	23	2	27	3	27	5	26	8	29	5	38	12	39	12	38
Psittacidae	*Pyrrhura picta*				1	4	9	2	8	3	8	6	8	7	9	6	10	12	10	13	9
Psittacidae	*Pyrrhura leucotis*				1	18	9	11	9	22	7	18	9	25	15	20	18	38	11	34	17
Psittacidae	*Pyrrhura viridicata*	T		x	1	2	0x	2	0x	2	0x	2	0x	2	0x	2	0x	2	45x	2	0x
Psittacidae	*Pyrrhura egregia*				1	0	17	0	17	1	17	0	18	0	22	1	27	2	29	0	32
Psittacidae	*Pyrrhura melanura*				1	4	1	2	1	3	0	6	0	7	1	7	1	10	0	15	0
Psittacidae	*Pyrrhura orcesi*	T		x	1	35	26x	0	3x	0	29x	0	3x	35	26x	0	3x	61	29x	61	3x
Psittacidae	*Pyrrhura rupicola*				1	10	5	8	4	12	5	17	4	19	6	18	6	22	6	28	6

Family	Species	IUCN	Ag	R	Range Source	Percentage loss in geographic range 2050								2100							
						TG		AM		GO		OS		TG		AM		GO		OS	
						H	C	H	C	H	C	H	C	H	C	H	C	H	C	H	C
Psittacidae	*Pyrrhura albipectus*	T		x	1	20	37x	3	48x	4	49x	4	48x	20	37x	3	48x	48	51x	42	51x
Psittacidae	*Pyrrhura calliptera*	T			1	22	0	42	0	32	0	50	0	27	0	42	0	76	10x	64	5
Psittacidae	*Pyrrhura hoematotis*				1	39	0	18	0	50	0	30	0	54	3	50	3	53	8	36	8
Psittacidae	*Pyrrhura rhodocephala*			x	1	26	0x	28	0x	34	0x	63	0x	32	25x	40	24x	44	38x	76	0x
Psittacidae	*Pyrrhura hoffmanni*				1	27	10	40	18x	16	18	37	18x	37	10x	42	23x	34	23x	59	18x
Psittacidae	*Enicognathus ferrugineus*				1	2	7	1	7	2	8	1	8	2	13	2	16	3	17	3	17
Psittacidae	*Enicognathus leptorhynchu*		H		1	0	11	0	7	0	9	0	9	0	14	0	11	0	11	0	12
Psittacidae	*Myiopsitta monachus*		H		1	0	6	0	5	0	5	0	4	0	7	0	9	0	11	0	9
Psittacidae	*Myiopsitta luchsi*			x	14	44	0x	71	0x	45	0x	83	0x	57	0x	71	0x	45	0x	83	0x
Psittacidae	*Bolborhynchus aymara*				1	6	9	3	9	5	8	8	7	7	13	4	18	7	18	11	13
Psittacidae	*Bolborhynchus aurifrons*		H		1	0	17	0	19	0	19	0	16	0	21	0	27	0	27	0	22
Psittacidae	*Bolborhynchus lineola*				1	12	3	16	3	14	5	21	3	15	5	20	10	22	9	26	4
Psittacidae	*Bolborhynchus orbygnesiu*				1	8	20	12	20	9	20	14	17	10	28	17	29	20	28	24	22
Psittacidae	*Bolborhynchus ferrugineifro*	T		x	1	0	0x	0	0x	0	0x	0	0x	0	0x	0	0x	0	0x	0	0x
Psittacidae	*Forpus cyanopygius*				1	13	10	17	5	8	7	32	2	15	14	17	24	10	11	35	2
Psittacidae	*Forpus passerinus*		H		1	0	12	0	12	0	12	0	11	0	14	0	16	0	17	0	15
Psittacidae	*Forpus xanthopterygius*		H		1	0	15	0	13	0	14	0	11	0	19	0	16	0	17	0	14
Psittacidae	*Forpus conspicillatus*				1	15	5	15	7	11	6	21	4	18	6	20	8	25	10	27	6
Psittacidae	*Forpus sclateri*				1	4	9	2	9	3	9	5	8	7	10	5	11	10	11	11	11
Psittacidae	*Forpus coelestis*		H		1	0	9	0	9	0	13	0	9	0	9	0	14	0	20	0	15
Psittacidae	*Forpus xanthops*	T		x	1	0	0x	6	0x	6	0x	7	0x	1	0x	6	2x	17	0x	17	0x
Psittacidae	*Brotogeris tirica*				1	14	9	11	7	20	5	19	5	20	15	20	13	33	9	32	10
Psittacidae	*Brotogeris versicolurus*				1	5	7	4	6	3	7	7	5	8	7	7	7	13	8	14	6
Psittacidae	*Brotogeris chiriri*				1	7	17	5	12	6	15	12	10	11	22	9	15	15	18	20	12
Psittacidae	*Brotogeris pyrrhopterus*	T			1	15	2	13	2	8	4	28	2	21	2	19	12	34	10	29	10
Psittacidae	*Brotogeris jugularis*				1	17	4	19	6	15	5	26	3	22	6	23	10	28	12	34	5
Psittacidae	*Brotogeris cyanoptera*				1	3	2	2	2	3	2	6	2	6	3	7	4	8	4	13	3
Psittacidae	*Brotogeris chrysopterus*				1	7	12	4	11	7	11	10	10	11	12	9	14	19	14	19	12
Psittacidae	*Brotogeris sanctithomae*				1	4	11	3	11	3	11	5	11	7	12	5	13	10	13	10	12
Psittacidae	*Nannopsittaca panychlora*				1	0	0	0	0	1	0	0	0	6	1	2	2	2	5	10	2
Psittacidae	*Nannopsittaca dachilleae*				1	4	6	1	6	4	6	12	6	4	10	23	10	7	10	20	10
Psittacidae	*Touit batavica*				1	12	18	7	18	14	16	21	14	18	19	16	21	26	21	27	15
Psittacidae	*Touit huetii*				1	7	10	5	9	7	9	13	8	14	10	11	10	25	11	25	8
Psittacidae	*Touit costaricensis*	T		x	1	24	16x	26	29x	23	29x	27	29x	27	16x	28	33x	53	33x	50	29x
Psittacidae	*Touit dilectissima*				1	10	4	13	6	10	4	13	4	16	9	15	9	15	15	24	6
Psittacidae	*Touit purpurata*				1	4	8	2	7	3	8	6	7	7	8	5	8	13	9	13	8
Psittacidae	*Touit melanonotus*	T			1	20	22x	22	24x	24	17x	16	21x	20	50x	28	44x	57	23x	36	45x
Psittacidae	*Touit surda*	T			1	14	14	12	17	20	12	12	15	22	23	16	30	38	19	31	28
Psittacidae	*Touit stictoptera*	T			1	12	2	7	2	14	1	22	2	18	2	21	2	30	1	44	2
Psittacidae	*Pionites melanocephala*				1	4	9	2	9	3	9	6	9	7	11	5	12	10	12	10	12
Psittacidae	*Pionites leucogaster*				1	4	10	3	10	3	10	7	9	7	11	6	11	15	11	15	10
Psittacidae	*Pionopsitta pileata*		H		1	0	6	0	5	0	3	0	5	0	14	0	13	0	8	0	9
Psittacidae	*Pionopsitta haematotis*				1	16	5	25	6	17	7	32	5	20	6	29	10	26	12	40	7
Psittacidae	*Pionopsitta pulchra*				1	12	4	12	7	11	3	10	4	19	4	12	7	13	7	17	4

Family	Species	IUCN	Ag	R	Range Source	2050 TG H	2050 TG C	2050 AM H	2050 AM C	2050 GO H	2050 GO C	2050 OS H	2050 OS C	2100 TG H	2100 TG C	2100 AM H	2100 AM C	2100 GO H	2100 GO C	2100 OS H	2100 OS C
Psittacidae	*Pionopsitta barrabandi*				1	2	6	2	6	1	7	3	6	4	8	3	8	5	9	7	8
Psittacidae	*Pionopsitta pyrilia*	T			1	17	2	19	5	13	5	23	2	25	9	26	11	25	16	33	6
Psittacidae	*Pionopsitta caica*				1	3	14	2	14	3	14	6	14	5	15	4	18	12	18	11	18
Psittacidae	*Gypopsitta vulturina*				1	18	14	10	13	12	13	21	11	28	14	20	15	43	15	40	13
Psittacidae	*Hapalopsittaca melanotis*				1	18	13	10	14	11	13	19	13	29	14	18	16	26	14	32	16
Psittacidae	*Hapalopsittaca amazonina*	T			1	13	0	32	3	20	3	43	3	24	12	38	14	47	19	56	7
Psittacidae	*Hapalopsittaca fuertesi*	T		x	1	0	25x	0	25x	0	25x	0	25x	0	25x	0	25x	0	25x	0	25x
Psittacidae	*Hapalopsittaca pyrrhops*	T			1	2	18	10	10	17	20x	17	20x	2	18	20	10	17	20x	17	20x
Psittacidae	*Graydidascalus brachyurus*				1	4	4	3	3	2	4	6	3	6	4	6	3	11	4	12	3
Psittacidae	*Pionus menstruus*		H		1	0	12	0	12	0	12	0	11	0	14	0	15	0	16	0	13
Psittacidae	*Pionus sordidus*				1	16	5	15	4	18	3	29	3	25	6	29	7	35	5	44	5
Psittacidae	*Pionus maximiliani*				1	9	15	6	14	8	15	13	12	13	20	11	19	19	20	23	17
Psittacidae	*Pionus tumultuosus*		H		1	0	6	0	5	0	5	0	5	0	9	0	12	0	11	0	6
Psittacidae	*Pionus senilis*		H		1	0	4	0	4	0	5	0	4	0	5	0	12	0	8	0	6
Psittacidae	*Pionus chalcopterus*				1	13	7	11	6	19	7	25	7	18	8	23	9	37	9	36	9
Psittacidae	*Pionus fuscus*				1	6	12	4	12	5	12	10	11	11	13	8	15	22	15	21	13
Psittacidae	*Amazona leucocephala*				1	10	11	29	12	16	8	38	5	18	11	32	14	20	9	41	5
Psittacidae	*Amazona collaria*	T	H	x	1	0	0x	0	3x	0	3x	0	3x	0	0x	0	3x	0	3x	0	3x
Psittacidae	*Amazona ventralis*	T	H		1	0	13	0	11	0	15	0	0	0	17	0	13	0	15	0	0
Psittacidae	*Amazona albifrons*		H		1	0	5	0	3	0	5	0	2	0	6	0	12	0	10	0	4
Psittacidae	*Amazona xantholora*				1	4	3	20	0	14	3	28	2	7	5	24	13	17	11	34	6
Psittacidae	*Amazona agilis*	T		x	1	18	0x	18	6x	10	6x	44	6x	44	0x	18	6x	10	6x	44	6x
Psittacidae	*Amazona vittata*	T		x	1	75	0x	75	0x	75	0x	75	0x	75	0x	75	0x	75	0x	75	0x
Psittacidae	*Amazona tucumana*				1	9	11	7	7	5	7	9	4	11	13	10	7	9	11	12	4
Psittacidae	*Amazona pretrei*	T			1	27	1	18	1	35	0	34	1	41	1	35	2	49	0	50	1
Psittacidae	*Amazona viridigenalis*	T	H		1	0	8	0	3	0	3	0	3	0	11	0	39	0	3	0	3
Psittacidae	*Amazona finschi*		H		1	0	8	0	6	0	8	0	3	0	11	0	23	0	13	0	6
Psittacidae	*Amazona autumnalis*		H		1	0	4	0	5	0	5	0	4	0	5	0	10	0	8	0	5
Psittacidae	*Amazona dufresniana*				1	2	21	7	17	3	19	4	19	4	23	13	20	9	25	8	25
Psittacidae	*Amazona rhodocorytha*	T			1	10	20	15	18	14	16	10	19	11	29	20	32	31	26	18	35
Psittacidae	*Amazona brasiliensis*	T			1	36	12x	5	31x	16	21x	16	29x	36	20x	18	38x	25	27x	24	34x
Psittacidae	*Amazona festiva*				1	4	1	2	1	1	1	5	0	6	2	4	1	7	2	9	0
Psittacidae	*Amazona xanthops*				1	6	21	2	12	2	17	9	9	8	27	5	14	8	20	16	11
Psittacidae	*Amazona barbadensis*	T			1	24	9	23	13	21	17	7	12	33	26	27	23	22	43	11	45
Psittacidae	*Amazona aestiva*		H		1	0	19	0	15	0	18	0	14	0	25	0	20	0	23	0	18
Psittacidae	*Amazona ochrocephala*		H		1	0	6	0	6	0	6	0	6	0	8	0	10	0	9	0	8
Psittacidae	*Amazona amazonica*		H		1	0	13	0	11	0	12	0	10	0	16	0	14	0	16	0	12
Psittacidae	*Amazona mercenaria*				1	14	5	18	4	19	5	30	3	23	7	33	7	37	7	44	4
Psittacidae	*Amazona farinosa*				1	5	9	4	9	4	9	9	8	9	10	8	11	15	11	16	10
Psittacidae	*Amazona kawalli*				1	11	22	3	29	15	22	12	19	16	22	13	29	26	22	29	19
Psittacidae	*Amazona vinacea*	T			1	23	10	19	10	28	7	33	8	28	20	35	17	47	9	50	15
Psittacidae	*Amazona versicolor*	T		x	1	0	44x	41	44x	44	0x	44	0x	0	44x	41	44x	44	0x	85	0x
Psittacidae	*Amazona arausiaca*	T		x	1	50	0x	50	0x	50	0x	50	0x	50	0x	50	0x	50	0x	50	0x
Psittacidae	*Amazona guildingii*	T		x	1	0	0x	100	0x	0	0x	0	0x	0	0x	100	0x	0	0x	100	0x

Family	Species	IUCN	Ag	R	Range Source	Percentage loss in geographic range 2050								2100							
						TG		AM		GO		OS		TG		AM		GO		OS	
						H	C	H	C	H	C	H	C	H	C	H	C	H	C	H	C
Psittacidae	*Amazona imperialis*	T		x	1	50	0x	50	0x	50	0x	50	0x	50	0x	50	0x	50	0x	50	0x
Psittacidae	*Deroptyus accipitrinus*				1	4	10	3	10	3	10	6	9	7	11	6	12	13	13	14	11
Psittacidae	*Triclaria malachitacea*		H		1	0	8	0	12	0	7	0	10	0	18	0	21	0	11	0	18
Apodidae	*Cypseloides niger*				1	11	18	20	14	14	17	26	12	15	23	24	23	22	25	34	16
Apodidae	*Cypseloides lemosi*			x	1	0	12x	0	12x	0	12x	0	12x	0	12x	0	12x	1	12x	0	12x
Apodidae	*Cypseloides rothschildi*				1	0	7	0	7	0	8	0	7	0	9	0	12	0	13	0	12
Apodidae	*Cypseloides fumigatus*				1	17	12	12	13	18	11	18	12	25	17	21	23	35	18	33	20
Apodidae	*Cypseloides cherriei*				1	27	11	17	15	21	15	41	7	34	11	37	21	45	21	49	7
Apodidae	*Cypseloides storeri*			x	1	13	17x	40	0x	20	17x	40	0x	13	17x	40	5x	21	17x	40	0x
Apodidae	*Cypseloides cryptus*				1	20	6	26	7	20	7	40	6	29	9	35	16	37	14	49	8
Apodidae	*Cypseloides senex*				1	8	14	7	11	8	14	14	10	14	20	12	16	23	16	23	13
Apodidae	*Streptoprocne phelpsi*			x	1	6	8x	6	8x	6	8x	6	8x	6	8x	19	14x	14	22x	19	19x
Apodidae	*Streptoprocne rutila*				1	15	4	20	3	17	4	29	2	19	5	25	10	25	8	34	3
Apodidae	*Streptoprocne zonaris*				1	10	12	11	9	11	11	16	8	14	16	15	14	20	15	22	11
Apodidae	*Streptoprocne biscutata*				1	12	11	10	5	15	6	17	4	16	19	17	11	27	10	27	8
Apodidae	*Streptoprocne semicollaris*				1	17	8	33	2	18	6	44	1	21	9	34	15	24	11	48	5
Apodidae	*Hydrochous gigas*				1	11	0	20	4	12	0	37	0	13	0	21	4	34	0	47	0x
Apodidae	*Collocalia esculenta*				1	11	4	14	3	17	4	17	3	17	6	20	6	30	7	29	5
Apodidae	*Collocalia linchi*				1	4	3	2	2	3	1	5	2	7	3	4	4	8	1	8	2
Apodidae	*Collocalia troglodytes*				1	12	13	18	12	11	14	15	14	15	18	22	17	21	21	23	17
Apodidae	*Aerodramus francicus*			x	1	43	0x	43	0x	43	0x	43	0x	43	0x	43	0x	43	0x	43	0x
Apodidae	*Aerodramus unicolor*				1	36	1	45	0	43	2	49	1	41	3	53	0	51	3	68	1
Apodidae	*Aerodramus mearnsi*				1	11	13	18	14	10	15	13	15	13	19	23	18	22	22	21	18
Apodidae	*Aerodramus infuscata*				1	14	5	21	5	20	5	13	7	18	7	22	16	30	18	22	19
Apodidae	*Aerodramus hirundinacea*				1	6	2	6	1	19	1	12	1	14	4	15	4	29	3	26	2
Apodidae	*Aerodramus spodiopygius*				1	12	7	17	4	35	4	35	2	25	7	30	4	57	4	53	2
Apodidae	*Aerodramus terraereginae*				1	0	33	0	22	0	36	2	15	0	43	0	38	0	46	2	23
Apodidae	*Aerodramus brevirostris*				1	23	4	24	4	22	4	23	4	25	5	26	5	24	6	26	6
Apodidae	*Aerodramus whiteheadi*			x	1	0	0x	27	0x	0	0x	0	0x	0	0x	27	0x	27	0x	0	0x
Apodidae	*Aerodramus nuditarsus*				1	7	0	5	0	22	0	17	0	13	3	11	1	38	1	29	0
Apodidae	*Aerodramus orientalis*			x	1	4	0x	6	0x	16	0x	21	0x	15	0x	6	0x	41	0x	69	0x
Apodidae	*Aerodramus salanganus*				1	14	0	19	1	15	2	21	1	21	0	24	1	32	2	33	1
Apodidae	*Aerodramus vanikorensis*				1	7	4	8	4	19	3	17	3	13	6	14	9	31	8	27	7
Apodidae	*Aerodramus maximus*				1	11	3	15	3	14	3	15	2	18	4	21	4	25	3	24	3
Apodidae	*Aerodramus fuciphagus*				1	11	5	13	5	13	5	13	5	17	6	18	6	23	5	22	5
Apodidae	*Aerodramus papuensis*				1	4	4	6	2	30	2	18	1	14	10	12	10	42	8	40	5
Apodidae	*Mearnsia picina*				1	20	14	26	16	12	15	25	17	32	16	26	16	27	20	40	17
Apodidae	*Mearnsia novaeguineae*				1	1	5	5	3	16	2	16	1	8	6	13	6	27	4	25	3
Apodidae	*Zoonavena grandidieri*				1	69	1	35	9	71	1	71	1	71	1	52	9	71	1	71	1
Apodidae	*Zoonavena thomensis*			x	1	0	87x	0	87x	87	0x	87	0x	0	87x	0	87x	87	0x	87	0x
Apodidae	*Zoonavena sylvatica*				1	29	7	42	6	34	7	42	5	33	8	47	7	40	8	67	6
Apodidae	*Telacanthura ussheri*				1	31	7	21	8	32	7	47	4	41	7	27	12	47	10	52	6
Apodidae	*Telacanthura melanopygia*				1	30	9	20	14	28	11	48	10	43	9	24	18	45	17	50	12
Apodidae	*Rhaphidura leucopygialis*				1	11	2	15	2	15	2	17	2	17	3	22	3	27	3	27	3

Family	Species	IUCN	Ag	R	Range Source	2050 TG H	2050 TG C	2050 AM H	2050 AM C	2050 GO H	2050 GO C	2050 OS H	2050 OS C	2100 TG H	2100 TG C	2100 AM H	2100 AM C	2100 GO H	2100 GO C	2100 OS H	2100 OS C
						Percentage loss in geographic range															
Apodidae	*Rhaphidura sabini*				1	32	6	18	9	33	7	52	5	46	7	27	14	48	12	57	7
Apodidae	*Neafrapus cassini*				1	31	6	16	9	30	7	53	4	49	7	27	10	54	9	61	5
Apodidae	*Neafrapus boehmi*				1	45	0	36	0	45	0	47	0	47	1	37	5	46	1	47	0
Apodidae	*Hirundapus caudacutus*				1	15	6	15	6	15	6	15	6	16	8	16	9	17	9	17	8
Apodidae	*Hirundapus cochinchinensi*				1	19	10	18	10	15	15	18	13	24	14	21	16	18	19	23	15
Apodidae	*Hirundapus giganteus*				1	15	4	19	5	17	5	21	5	20	6	23	7	27	8	31	6
Apodidae	*Hirundapus celebensis*				1	12	15	20	12	8	16	14	14	14	20	24	16	18	22	23	16
Apodidae	*Chaetura spinicauda*				1	10	14	8	13	8	13	18	9	16	15	13	14	22	16	30	11
Apodidae	*Chaetura egregia*				1	5	5	2	5	3	4	6	4	7	6	7	8	8	7	13	7
Apodidae	*Chaetura martinica*			x	1	6	18x	40	9x	14	9x	37	0x	6	18x	54	9x	28	9x	63	0x
Apodidae	*Chaetura cinereiventris*				1	10	6	8	6	12	5	14	5	15	8	15	10	22	8	23	9
Apodidae	*Chaetura pelagica*				1	9	20	14	11	13	16	13	8	14	24	19	15	24	20	19	10
Apodidae	*Chaetura vauxi*				1	11	12	19	9	15	11	23	7	14	15	22	19	21	18	27	11
Apodidae	*Chaetura chapmani*				1	7	14	6	15	6	15	10	14	11	17	9	20	16	21	17	18
Apodidae	*Chaetura brachyura*				1	6	9	4	8	5	8	9	8	10	10	8	10	16	11	16	10
Apodidae	*Chaetura andrei*				1	7	11	6	10	9	10	11	8	10	15	12	15	17	16	19	14
Apodidae	*Schoutedenapus myoptilus*				1	32	4	25	7	36	2	38	0	36	4	29	9	38	2	39	0
Apodidae	*Schoutedenapus schouted*	T			1	37	3	12	15	39	3	40	0	40	3	24	17	40	3	40	0
Apodidae	*Aeronautes saxatalis*				1	3	20	6	18	4	20	8	17	3	23	7	28	6	30	10	25
Apodidae	*Aeronautes montivagus*				1	16	5	13	5	14	5	21	3	21	6	21	8	27	9	27	6
Apodidae	*Aeronautes andecolus*				1	5	16	6	17	5	17	9	14	7	21	8	27	12	26	15	22
Apodidae	*Tachornis phoenicobia*				1	12	8	25	7	15	8	32	2	17	9	26	8	18	8	34	2
Apodidae	*Tachornis furcata*				1	20	2	15	6	15	2	23	0	24	14	20	21	23	29	27	10
Apodidae	*Tachornis squamata*				1	10	13	8	11	10	12	17	8	13	16	13	14	20	15	27	11
Apodidae	*Panyptila sanctihieronymi*				1	13	4	31	1	23	4	39	1	17	4	31	11	29	5	41	1
Apodidae	*Panyptila cayennensis*				1	8	16	7	16	7	17	14	14	12	18	11	19	17	20	22	16
Apodidae	*Cypsiurus parvus*				1	33	3	21	6	34	3	42	2	39	4	26	9	42	4	44	3
Apodidae	*Cypsiurus balasiensis*				1	15	5	18	5	16	5	19	5	18	7	21	8	22	9	27	6
Apodidae	*Tachymarptis melba*				1	13	6	10	7	14	7	16	5	15	9	13	11	16	10	18	7
Apodidae	*Tachymarptis aequatorialis*				1	38	4	26	8	41	2	41	2	39	4	30	12	43	3	43	2
Apodidae	*Apus alexandri*			x	1	28	5x	18	5x	41	0x	83	0x	28	5x	18	5x	50	0x	83	0x
Apodidae	*Apus apus*				1	4	13	4	12	5	14	5	11	7	16	6	17	9	21	9	16
Apodidae	*Apus unicolor*			x	1	0	10x	3	10x	0	10x	8	10x	0	10x	3	10x	0	16x	8	12x
Apodidae	*Apus niansae*				1	14	1	9	3	16	0	16	0	15	1	11	4	16	0	16	0
Apodidae	*Apus pallidus*				1	3	4	2	3	3	4	4	3	5	5	4	4	5	5	5	4
Apodidae	*Apus barbatus*				1	31	2	22	5	32	3	32	2	31	3	26	8	33	3	33	3
Apodidae	*Apus berliozi*				1	0	0	0	0	0	0	0	0	0	0	0	0	0	0	0	0
Apodidae	*Apus bradfieldi*				1	3	8	1	9	2	10	2	9	3	13	1	20	2	18	2	18
Apodidae	*Apus pacificus*				1	9	7	9	7	9	8	9	7	9	9	9	11	10	12	10	11
Apodidae	*Apus acuticauda*	T			1	38	0	46	0	36	2	46	0	43	0	46	0	41	2	46	0
Apodidae	*Apus affinis*				1	19	3	12	6	20	3	23	2	22	4	15	9	23	5	25	3
Apodidae	*Apus nipalensis*				1	14	6	17	5	16	6	17	6	17	10	21	9	21	11	22	9
Apodidae	*Apus horus*				1	19	4	16	4	20	4	20	4	20	5	17	8	21	6	20	6
Apodidae	*Apus caffer*				1	22	3	13	7	24	2	25	2	24	4	15	11	26	4	26	4

Family	Species	IUCN	Ag	R	Range Source	Percentage loss in geographic range															
						2050								2100							
						TG		AM		GO		OS		TG		AM		GO		OS	
						H	C	H	C	H	C	H	C	H	C	H	C	H	C	H	C
Apodidae	*Apus batesi*				1	32	2	18	3	31	6	46	4	51	2	31	8	51	13	52	7
Hemiprocnidae	*Hemiprocne coronata*				1	19	5	22	4	20	5	24	4	22	7	25	7	24	10	33	6
Hemiprocnidae	*Hemiprocne longipennis*				1	12	4	15	4	15	4	16	4	18	5	21	7	26	7	24	6
Hemiprocnidae	*Hemiprocne mystacea*				1	6	2	7	2	19	1	17	1	13	5	14	5	31	4	29	2
Hemiprocnidae	*Hemiprocne comata*				1	13	3	17	3	15	4	19	3	20	4	23	4	28	5	28	3
Trochilidae	*Glaucis aenea*				1	19	6	28	7	26	8	35	5	28	6	32	10	33	10	43	5
Trochilidae	*Glaucis hirsuta*				1	6	12	4	12	5	12	10	11	10	14	9	15	15	16	18	14
Trochilidae	*Threnetes niger*				1	5	9	4	8	4	8	8	8	9	10	8	10	13	11	16	10
Trochilidae	*Threnetes ruckeri*				1	17	4	21	7	18	7	27	5	23	6	26	9	27	11	34	6
Trochilidae	*Phaethornis yaruqui*				1	14	3	13	5	14	2	11	4	23	3	13	5	18	5	22	4
Trochilidae	*Phaethornis guy*				1	17	5	19	5	17	5	33	4	26	5	28	9	35	8	47	5
Trochilidae	*Phaethornis syrmatophorus*				1	11	7	13	6	15	7	26	5	16	8	22	9	29	9	36	7
Trochilidae	*Phaethornis superciliosus*				1	5	12	4	12	4	12	9	11	10	13	7	14	18	16	18	14
Trochilidae	*Phaethornis longirostris*				14	15	6	25	5	18	7	30	4	19	7	28	11	25	11	37	5
Trochilidae	*Phaethornis malaris*				1	5	6	3	6	4	6	8	6	8	7	8	8	11	8	14	7
Trochilidae	*Phaethornis eurynome*				1	20	9	18	8	28	6	29	7	29	16	31	15	46	10	46	14
Trochilidae	*Phaethornis hispidus*				1	4	11	3	11	3	11	6	11	7	13	6	14	9	14	12	14
Trochilidae	*Phaethornis anthophilus*				1	15	5	18	8	15	7	27	4	20	9	23	12	30	15	35	6
Trochilidae	*Phaethornis bourcieri*				1	4	8	3	8	2	8	5	8	7	9	7	10	9	11	12	10
Trochilidae	*Phaethornis koepckeae*				1	22	6	25	2	27	4	43	5	35	6	40	6	50	4	55	5
Trochilidae	*Phaethornis philippii*				1	1	10	0	10	1	10	2	10	3	11	4	12	3	13	6	11
Trochilidae	*Phaethornis squalidus*				1	23	14	12	20	28	12	23	17	24	34	25	35	47	19	33	31
Trochilidae	*Phaethornis rupurumii*				1	12	13	7	15	9	14	12	14	19	16	15	18	27	18	25	19
Trochilidae	*Phaethornis augusti*				1	13	11	10	12	13	10	20	8	21	13	18	18	31	16	32	12
Trochilidae	*Phaethornis pretrei*				1	9	14	7	9	8	11	15	6	12	20	12	14	16	17	23	11
Trochilidae	*Phaethornis subochraceus*				1	12	18	7	19	6	18	15	17	20	20	15	31	30	28	35	27
Trochilidae	*Phaethornis nattereri*				1	8	25	1	31	4	33	12	26	9	35	5	39	11	41	21	34
Trochilidae	*Phaethornis gounellei*				1	6	16	5	11	5	13	13	4	7	20	8	11	9	15	18	6
Trochilidae	*Phaethornis ruber*				1	5	10	4	9	4	10	8	9	9	11	8	11	14	12	17	11
Trochilidae	*Phaethornis stuarti*				1	34	8	22	6	21	6	33	8	38	8	35	9	36	9	36	11
Trochilidae	*Phaethornis griseogularis*				1	7	6	7	6	7	6	13	6	14	8	17	8	17	9	26	10
Trochilidae	*Phaethornis longuemareus*				1	5	20	5	19	6	19	11	17	8	20	10	23	12	25	14	19
Trochilidae	*Phaethornis atrimentalis*				14	10	1	7	1	9	1	18	0	17	1	19	1	23	1	34	0
Trochilidae	*Phaethornis striigularis*				1	17	4	20	6	17	6	27	3	22	6	26	10	26	10	35	5
Trochilidae	*Phaethornis idaliae*				1	7	6	7	6	12	3	13	6	12	7	8	8	25	5	15	8
Trochilidae	*Eutoxeres aquila*				1	15	3	14	5	13	4	24	3	24	3	23	6	29	6	42	4
Trochilidae	*Eutoxeres condamini*				1	13	4	10	3	11	3	21	3	20	4	21	5	26	4	36	3
Trochilidae	*Androdon aequatorialis*				1	9	6	11	8	7	6	11	5	16	8	12	8	13	10	21	7
Trochilidae	*Ramphodon naevius*				1	30	12	19	19	30	11	30	16	35	31	35	33	52	17	43	31
Trochilidae	*Ramphodon dohrnii*	T		x	1	7	5x	9	5x	35	1x	5	5x	12	6x	14	22x	49	14x	33	22x
Trochilidae	*Doryfera johannae*				1	8	10	10	10	8	11	11	12	14	13	18	17	16	18	23	21
Trochilidae	*Doryfera ludovicae*				1	22	9	21	9	14	11	34	9	30	11	28	16	37	16	41	13
Trochilidae	*Phaeochroa cuvierii*				1	16	7	30	8	21	9	37	7	21	8	36	11	30	12	43	8
Trochilidae	*Campylopterus curvipennis*				1	6	3	16	2	10	3	23	2	7	4	20	12	13	8	28	4

Family	Species	IUCN	Ag	R	Range Source	Percentage loss in geographic range 2050 TG		2050 AM		2050 GO		2050 OS		2100 TG		2100 AM		2100 GO		2100 OS	
						H	C	H	C	H	C	H	C	H	C	H	C	H	C	H	C
Trochilidae	*Campylopterus largipennis*				1	6	11	4	10	4	11	9	10	9	12	7	12	14	13	17	11
Trochilidae	*Campylopterus rufus*		H		1	0	0	0	0	0	0	0	0	0	0	0	22	0	0	0	0
Trochilidae	*Campylopterus hyperythrus*				1	7	7	0	14	7	10	0	14	7	15	9	25	12	25	5	34
Trochilidae	*Campylopterus duidae*				1	0	13	2	12	0	13	0	13	1	16	2	17	0	19	1	19
Trochilidae	*Campylopterus hemileucur*		H		1	0	2	0	3	0	4	0	3	0	2	0	8	0	6	0	3
Trochilidae	*Campylopterus ensipennis*			x	1	31	0x	2	0x	2	0x	24	0x	31	0x	9	0x	16	0x	36	0x
Trochilidae	*Campylopterus falcatus*				1	21	4	20	5	17	4	33	3	29	7	31	8	46	10	52	4
Trochilidae	*Campylopterus phainopepl*	T		x	1	0	0x	23	0x	23	0x	0	0x	23	0x	23	0x	23	10x	23	0x
Trochilidae	*Campylopterus villaviscens*				1	12	1	8	1	11	0	24	1	21	2	20	4	30	0	42	3
Trochilidae	*Eupetomena macroura*				1	7	17	6	11	7	14	12	8	10	23	10	14	14	17	18	11
Trochilidae	*Florisuga mellivora*				1	6	11	5	11	5	12	10	11	10	13	9	14	15	15	18	13
Trochilidae	*Melanotrochilus fuscus*				1	14	13	15	12	21	11	22	10	20	17	23	18	36	15	35	17
Trochilidae	*Colibri delphinae*				1	14	10	15	10	15	10	23	9	21	12	23	15	28	15	33	13
Trochilidae	*Colibri thalassinus*				1	16	5	18	5	15	6	25	4	22	7	24	9	27	10	32	7
Trochilidae	*Colibri coruscans*				1	15	10	13	10	12	10	20	9	19	14	21	17	28	17	29	15
Trochilidae	*Colibri serrirostris*				1	7	14	6	8	7	11	11	7	10	20	10	12	14	15	17	9
Trochilidae	*Anthracothorax viridigula*				1	5	12	4	12	5	12	9	11	8	13	7	15	19	15	18	13
Trochilidae	*Anthracothorax prevostii*		H		1	0	3	0	3	0	4	0	2	0	5	0	10	0	9	0	6
Trochilidae	*Anthracothorax veraguensi*				1	29	0	47	5	7	5	39	5	37	0	49	5	11	5	50	5x
Trochilidae	*Anthracothorax nigricollis*				1	8	13	6	11	7	12	12	10	12	15	10	15	18	15	21	13
Trochilidae	*Anthracothorax mango*			x	1	17	0x	17	5x	10	5x	40	5x	40	0x	17	5x	10	5x	40	5x
Trochilidae	*Anthracothorax dominicus*				1	15	11	20	10	17	14	32	0	15	15	20	11	17	14	32	0
Trochilidae	*Anthracothorax viridis*			x	1	1	0x	1	0x	1	0x	1	0x	1	0x	1	0x	1	0x	1	0x
Trochilidae	*Avocettula recurvirostris*				1	5	14	4	14	4	14	8	14	8	15	7	18	16	19	15	19
Trochilidae	*Eulampis jugularis*			x	1	4	14x	44	9x	22	5x	39	0x	4	14x	59	9x	35	5x	73	0x
Trochilidae	*Eulampis holosericeus*			x	1	20	10x	49	7x	31	3x	45	0x	20	10x	59	7x	42	3x	68	0x
Trochilidae	*Chrysolampis mosquitus*		H		1	0	16	0	14	0	15	0	11	0	20	0	17	0	19	0	14
Trochilidae	*Orthorhyncus cristatus*			x	1	27	8x	48	5x	37	4x	48	0x	27	8x	55	5x	45	4x	63	0x
Trochilidae	*Klais guimeti*				1	15	4	18	5	17	5	27	5	22	6	28	8	29	10	35	7
Trochilidae	*Abeillia abeillei*				1	10	1	18	1	13	2	19	1	11	1	22	4	16	4	20	1
Trochilidae	*Stephanoxis lalandi*				1	19	3	15	3	22	2	25	3	24	6	31	7	42	4	44	6
Trochilidae	*Lophornis ornatus*				1	2	14	2	14	3	13	5	14	5	15	4	17	11	17	8	17
Trochilidae	*Lophornis gouldii*				1	6	19	2	17	3	20	11	14	10	24	6	20	15	23	22	17
Trochilidae	*Lophornis magnificus*				1	11	12	10	6	15	6	16	4	15	19	17	11	27	10	27	8
Trochilidae	*Lophornis brachylopha*	T		x	1	11	0x	18	0x	11	0x	18	0x	11	0x	18	8x	18	0x	18	0x
Trochilidae	*Lophornis delattrei*				1	12	12	14	14	14	14	22	13	18	16	20	23	27	22	30	22
Trochilidae	*Lophornis stictolophus*				1	14	2	15	3	12	3	20	2	20	5	31	7	29	7	42	3
Trochilidae	*Lophornis chalybeus*				1	5	11	4	11	4	11	7	11	9	14	9	15	11	14	14	15
Trochilidae	*Lophornis pavoninus*				1	4	18	6	19	2	21	1	22	9	25	8	29	4	33	3	36
Trochilidae	*Lophornis helenae*				1	17	5	27	5	21	7	35	4	21	6	32	9	27	7	41	6
Trochilidae	*Lophornis adorabilis*				1	23	7	34	16	10	19	37	16	33	7	34	28	21	28	53	16x
Trochilidae	*Popelairia popelairii*				1	12	3	15	3	19	2	29	1	20	4	28	5	34	3	43	2
Trochilidae	*Popelairia langsdorffi*				1	2	1	1	1	2	1	3	1	5	2	5	2	5	2	8	2
Trochilidae	*Popelairia letitiae*				1	3	22	0	26	1	26	0	26	12	27	0	87x	9	78x	4	87x

Family	Species	IUCN	Ag	R	Range Source	Percentage loss in geographic range 2050 TG		AM		GO		OS		2100 TG		AM		GO		OS	
						H	C	H	C	H	C	H	C	H	C	H	C	H	C	H	C
Trochilidae	*Popelairia conversii*				1	12	6	16	8	10	8	18	6	18	8	19	11	19	13	26	8
Trochilidae	*Discosura longicauda*				1	3	15	4	14	4	15	4	15	5	17	5	20	8	21	7	21
Trochilidae	*Chlorestes notatus*		H		1	0	12	0	11	0	11	0	9	0	13	0	13	0	14	0	12
Trochilidae	*Chlorostilbon olivaresi*			x	1	2	0x	16	0x	2	0x	26	0x	21	0x	16	0x	46	0x	55	0x
Trochilidae	*Chlorostilbon mellisugus*		H		1	0	7	0	7	0	7	0	6	0	8	0	11	0	11	0	8
Trochilidae	*Chlorostilbon aureoventris*				1	6	12	5	8	7	9	10	6	9	15	8	11	12	13	15	9
Trochilidae	*Chlorostilbon ricordii*				1	11	11	33	11	23	8	41	5	21	11	35	13	28	9	47	5
Trochilidae	*Chlorostilbon swainsonii*				1	11	8	16	6	11	9	22	0	11	9	16	6	11	9	22	0
Trochilidae	*Chlorostilbon maugaeus*			x	1	19	0x	19	0x	19	0x	19	0x	19	0x	19	0x	19	0x	19	0x
Trochilidae	*Chlorostilbon russatus*				1	21	1	22	6	15	4	23	3	29	9	27	16	32	18	35	6
Trochilidae	*Chlorostilbon stenura*				1	34	2	26	5	25	6	34	4	41	11	39	16	39	27	45	13
Trochilidae	*Chlorostilbon alice*				1	27	5	22	5	26	10	16	8	33	13	35	11	31	23	19	26
Trochilidae	*Chlorostilbon poortmani*				1	13	3	15	3	9	6	21	3	19	5	21	11	28	15	30	8
Trochilidae	*Cynanthus sordidus*				1	8	3	27	0	16	3	31	0	10	3	27	8	21	3	32	0
Trochilidae	*Cynanthus latirostris*				1	5	21	17	15	5	18	23	16	8	21	17	25	8	29	28	25
Trochilidae	*Cyanophaia bicolor*			x	1	23	9x	31	0x	23	9x	31	0x	23	9x	59	0x	23	9x	59	0x
Trochilidae	*Thalurania ridgwayi*	T			1	14	7	29	4	24	7	34	2	20	7	29	18	26	7	41	2
Trochilidae	*Thalurania colombica*				1	19	6	27	7	20	8	35	5	25	7	33	13	31	15	43	6
Trochilidae	*Thalurania fannyi*				1	10	5	13	8	9	6	16	3	15	6	14	8	17	9	23	5
Trochilidae	*Thalurania furcata*				1	5	12	4	11	4	12	9	10	9	15	8	14	14	15	16	13
Trochilidae	*Thalurania watertonii*				1	8	22	6	22	9	21	15	15	10	24	8	25	17	25	26	20
Trochilidae	*Thalurania glaucopis*				1	14	9	12	6	20	6	21	4	21	14	21	10	32	9	33	8
Trochilidae	*Panterpe insignis*			x	1	23	10x	40	14x	42	14x	49	10x	26	10x	40	18x	60	17x	65	10x
Trochilidae	*Damophila julie*		H		1	0	5	0	6	0	5	0	4	0	6	0	6	0	9	0	5
Trochilidae	*Lepidopyga coeruleogularis*				1	9	3	15	4	9	5	17	3	13	4	16	4	13	8	22	4
Trochilidae	*Lepidopyga lilliae*	T		x	1	2	0x	17	0x	19	0x	2	0x	19	0x	17	0x	19	0x	19	0x
Trochilidae	*Lepidopyga goudoti*				1	14	1	17	4	16	3	23	1	16	5	19	9	25	16	25	4
Trochilidae	*Hylocharis xantusii*				1	0	1	0	1	0	0	6	0	0	1	0	1	0	0	6	0
Trochilidae	*Hylocharis leucotis*				1	13	6	24	2	16	4	31	1	15	6	26	14	21	10	35	4
Trochilidae	*Hylocharis eliciae*				1	17	6	27	8	20	9	33	6	22	8	30	11	27	13	40	8
Trochilidae	*Hylocharis sapphirina*				1	5	9	4	8	4	8	7	8	8	11	8	11	12	12	14	11
Trochilidae	*Hylocharis cyanus*				1	5	12	4	13	5	13	9	12	9	14	8	16	15	17	17	15
Trochilidae	*Hylocharis chrysura*				1	8	8	6	6	8	6	11	5	11	11	9	9	14	9	16	7
Trochilidae	*Hylocharis grayi*				1	5	7	9	5	9	5	7	4	12	7	10	5	16	6	16	4
Trochilidae	*Chrysuronia oenone*				1	11	3	8	2	9	2	15	2	16	4	16	4	21	4	27	3
Trochilidae	*Goldmania violiceps*			x	1	12	22x	32	35x	18	35x	40	22x	12	32x	32	35x	49	45x	43	32x
Trochilidae	*Goethalsia bella*			x	1	24	51x	7	75x	0	75x	46	51x	24	51x	7	75x	7	75x	49	51x
Trochilidae	*Trochilus polytmus*			x	1	13	0x	13	0x	13	0x	45	0x	45	0x	13	0x	13	0x	45	0x
Trochilidae	*Trochilus scitulus*			x	14	23	0x	23	28x	0	28x	23	28x	23	0x	23	28x	0	28x	23	28x
Trochilidae	*Leucochloris albicollis*				1	9	7	8	5	12	5	14	5	14	10	14	9	21	8	22	9
Trochilidae	*Polytmus guainumbi*				1	9	14	6	8	8	10	12	6	12	19	11	11	16	14	19	9
Trochilidae	*Polytmus milleri*				1	9	16	7	21	6	22	0	25	14	26	10	34	9	37	3	44
Trochilidae	*Polytmus theresiae*				1	3	13	3	12	2	12	5	13	5	15	5	16	9	17	9	17
Trochilidae	*Leucippus fallax*				1	31	4	18	14	23	7	22	4	35	8	23	15	27	13	34	12

Family	Species	IUCN	Ag	R	Range Source	2050 TG H	2050 TG C	2050 AM H	2050 AM C	2050 GO H	2050 GO C	2050 OS H	2050 OS C	2100 TG H	2100 TG C	2100 AM H	2100 AM C	2100 GO H	2100 GO C	2100 OS H	2100 OS C
Trochilidae	*Leucippus baeri*				1	0	6x	0	6x	0	6x	0	6x	0	6x	0	18x	0	18x	0	18x
Trochilidae	*Leucippus taczanowskii*			x	1	0	3x	2	3x	2	3x	2	3x	0	3x	2	3x	3	3x	3	3x
Trochilidae	*Leucippus chlorocercus*				1	0	1	0	0	0	0	2	0	0	1	7	0	0	0	13	0
Trochilidae	*Taphrospilus hypostictus*				1	19	7	17	7	17	7	32	6	27	8	28	11	37	9	45	7
Trochilidae	*Amazilia viridicauda*			x	1	3	10x	1	0x	0	10x	3	10x	5	10x	1	28x	25	10x	25	10x
Trochilidae	*Amazilia chionogaster*				1	9	9	6	9	5	9	11	6	12	10	7	14	13	13	19	6
Trochilidae	*Amazilia candida*				1	10	4	20	3	14	4	27	3	12	5	25	10	19	9	32	5
Trochilidae	*Amazilia chionopectus*				1	7	8	7	7	12	7	14	5	12	10	16	13	19	12	22	9
Trochilidae	*Amazilia versicolor*				1	8	14	6	10	8	11	12	8	12	19	11	13	17	15	21	11
Trochilidae	*Agyrtria rondoniae*				14	4	6	18	6	0	6	6	6	36	6	18	6	31	6	39	6
Trochilidae	*Amazilia luciae*	T		x	1	10	0x	13	0x	12	0x	12	0x	11	0x	13	0x	13	0x	26	1x
Trochilidae	*Amazilia fimbriata*				1	7	15	5	13	5	14	11	11	10	18	9	16	16	17	19	14
Trochilidae	*Amazilia lactea*				1	9	13	8	5	10	7	14	4	13	19	13	8	19	10	21	6
Trochilidae	*Amazilia amabilis*				1	16	6	22	8	18	9	28	4	24	7	27	10	28	12	36	6
Trochilidae	*Amazilia rosenbergi*			x	1	8	2x	9	3x	7	0x	8	3x	14	2x	9	3x	7	3x	11	3x
Trochilidae	*Amazilia boucardi*	T		x	1	5	0x	0	0x	5	0x	25	0x	5	0x	0	5x	25	0x	25	0x
Trochilidae	*Amazilia franciae*				1	17	6	17	5	18	7	23	4	20	6	21	6	21	8	27	5
Trochilidae	*Amazilia leucogaster*				1	2	19	2	18	6	17	10	15	6	21	5	23	19	21	24	19
Trochilidae	*Amazilia cyanocephala*				1	10	3	19	3	13	4	23	2	11	4	22	11	17	5	25	3
Trochilidae	*Amazilia cyanifrons*				1	7	8	1	6	1	8	8	6	8	8	7	8	10	8	8	8
Trochilidae	*Amazilia beryllina*				1	11	9	26	4	14	8	31	4	14	10	27	14	19	13	35	9
Trochilidae	*Amazilia cyanura*				1	13	0	15	0	20	4	25	0	13	0	15	4	20	5	25	0
Trochilidae	*Amazilia saucerrottei*				1	13	2	12	3	13	4	20	0	17	3	17	9	21	10	24	1
Trochilidae	*Amazilia tobaci*				1	19	5	12	7	19	5	21	4	25	6	21	9	34	10	32	8
Trochilidae	*Amazilia viridigaster*				1	13	2	9	3	6	3	11	1	13	2	12	3	9	6	14	1
Trochilidae	*Saucerottia cupreicauda*				14	7	7	3	11	5	8	3	11	8	7	8	18	9	18	7	21
Trochilidae	*Amazilia edward*				1	23	12	31	17	14	19	31	17	27	12	32	22	21	23	49	17
Trochilidae	*Amazilia rutila*				1	11	4	20	3	14	5	31	2	13	6	22	14	19	9	35	4
Trochilidae	*Amazilia yucatanensis*				1	3	4	8	2	6	3	10	2	4	6	10	20	7	6	12	3
Trochilidae	*Amazilia tzacatl*		H		1	0	4	0	5	0	5	0	3	0	5	0	9	0	9	0	4
Trochilidae	*Amazilia castaneiventris*	T		x	1	29	0x	10	0x	10	0x	29	0x	29	0x	29	0x	29	0x	29	0x
Trochilidae	*Amazilia amazilia*				1	7	16	7	16	8	20	12	10	8	24	10	27	16	30	18	19
Trochilidae	*Amazilia alticola*			x	14	0	0x	0	0x	0	0x	0	0x	0	0x	0	0x	0	0x	0	0x
Trochilidae	*Amazilia viridifrons*				1	3	6	18	0	4	5	16	0	3	6	18	6	12	5	16	0
Trochilidae	*Amazilia violiceps*				1	14	11	28	8	16	10	35	7	17	13	28	23	21	22	39	15
Trochilidae	*Eupherusa poliocerca*	T			1	4	7	22	0	7	7	32	0	4	7	22	7	18	7	32	0
Trochilidae	*Eupherusa cyanophrys*	T		x	1	11	1x	32	0x	11	0x	11	0x	11	1x	32	1x	32	0x	11	0x
Trochilidae	*Eupherusa eximia*				1	16	5	22	6	21	7	34	5	18	6	28	12	30	12	41	6
Trochilidae	*Eupherusa nigriventris*			x	1	22	14x	33	18x	34	18x	36	18x	24	14x	36	21x	65	21x	53	18x
Trochilidae	*Elvira chionura*				1	30	9	43	17x	11	19	39	17	42	9	44	23x	24	26	59	17x
Trochilidae	*Elvira cupreiceps*			x	1	51	0x	39	15x	61	15x	78	0x	51	0x	43	33x	61	15x	78	0x
Trochilidae	*Microchera albocoronata*				1	29	6	49	9	38	11	59	9	36	6	55	10	51	11	66	9
Trochilidae	*Chalybura buffonii*				1	13	3	15	3	13	4	21	2	21	5	20	5	26	9	30	4
Trochilidae	*Chalybura urochrysia*				1	21	6	26	7	21	8	29	6	29	7	31	8	30	12	37	8

Family	Species	IUCN	Ag	R	Range Source	Percentage loss in geographic range 2050 TG H	TG C	AM H	AM C	GO H	GO C	OS H	OS C	2100 TG H	TG C	AM H	AM C	GO H	GO C	OS H	OS C
Trochilidae	*Aphantochroa cirrochloris*				1	12	13	11	8	16	9	19	5	17	21	18	13	26	13	28	10
Trochilidae	*Lampornis clemenciae*				1	10	11	22	6	12	8	27	5	12	11	22	19	17	15	30	9
Trochilidae	*Lampornis amethystinus*				1	9	3	21	1	14	2	25	0	11	3	22	7	20	3	26	1
Trochilidae	*Lampornis viridipallens*				1	12	3	26	3	15	3	31	3	15	5	32	12	25	6	34	7
Trochilidae	*Lampornis sybillae*				1	15	0	20	0	18	4	26	0	15	0	25	0	24	4	29	1
Trochilidae	*Lampornis hemileucus*			x	1	21	15x	27	23x	28	23x	29	23x	23	15x	30	25x	56	25x	50	23x
Trochilidae	*Lampornis castaneoventris*				1	33	9	28	19	40	19x	52	16x	36	9	35	29x	58	21x	64	16x
Trochilidae	*Lamprolaima rhami*				1	8	3	17	1	11	3	19	1	10	3	19	8	14	3	20	2
Trochilidae	*Adelomyia melanogenys*				1	16	6	16	5	16	6	26	4	23	7	26	8	30	9	36	6
Trochilidae	*Anthocephala floriceps*	T			1	0	7	6	7	7	7	0	7	7	7	6	7	13	7	7	7
Trochilidae	*Phlogophilus hemileucurus*				1	12	3	13	3	23	2	32	3	21	5	31	8	46	2	53	6
Trochilidae	*Phlogophilus harterti*				1	24	11	25	6	22	9	41	8	36	11	33	11	39	9	56	8
Trochilidae	*Clytolaema rubricauda*				1	14	9	12	7	18	5	19	6	17	17	19	15	31	11	28	12
Trochilidae	*Heliodoxa imperatrix*				1	12	2	9	1	12	0	9	1	18	2	9	1	12	1	16	1
Trochilidae	*Heliodoxa xanthogonys*				1	7	18	8	19	5	21	7	22	14	22	11	26	11	29	11	31
Trochilidae	*Heliodoxa gularis*				1	8	1	3	1	4	0	12	1	13	1	10	1	15	0	18	1
Trochilidae	*Heliodoxa branickii*				1	15	10	8	9	8	10	15	10	19	10	13	19	23	13	22	13
Trochilidae	*Heliodoxa schreibersii*				1	6	1	2	0	3	1	7	0	11	1	10	0	12	1	20	0
Trochilidae	*Heliodoxa aurescens*				1	3	6	1	6	1	6	4	6	5	7	4	7	6	8	9	7
Trochilidae	*Heliodoxa rubinoides*		H		1	0	6	0	5	0	6	0	5	0	6	0	7	0	7	0	7
Trochilidae	*Heliodoxa jacula*				1	24	7	26	13	21	13	26	10	25	12	29	13	37	18	36	15
Trochilidae	*Heliodoxa leadbeateri*		H		1	0	6	0	6	0	6	0	3	0	8	0	11	0	12	0	6
Trochilidae	*Eugenes fulgens*		H		1	0	10	0	6	0	8	0	6	0	11	0	16	0	15	0	9
Trochilidae	*Hylonympha macrocerca*	T		x	1	8	0x	0	0x	0	0x	8	0x	8	0x	0	0x	0	0x	8	0x
Trochilidae	*Sternoclyta cyanopectus*				1	32	0x	18	0	27	0	35	0x	33	15x	38	16x	33	27x	41	8x
Trochilidae	*Topaza pella*				1	5	9	3	8	3	9	7	8	9	9	6	10	13	11	13	10
Trochilidae	*Oreotrochilus chimborazo*			x	1	18	2x	37	2x	16	2x	57	0x	32	2x	68	7x	98	2x	57	5x
Trochilidae	*Oreotrochilus estella*				1	13	21	11	24	9	23	16	20	16	29	17	32	20	34	28	23
Trochilidae	*Oreotrochilus stolzmanni*				14	0	0	10	0	6	0	6	0	0	7	10	3	10	3	10	2
Trochilidae	*Oreotrochilus leucopleurus*				1	4	6	5	7	3	7	9	5	8	9	7	11	7	17	15	11
Trochilidae	*Oreotrochilus melanogaste*				1	1	9	6	8	6	1	2	1	1	12	6	12	6	1	2	4
Trochilidae	*Oreotrochilus adela*				1	27	9	14	15	18	15	32	0	34	9	20	20	25	15	47	0
Trochilidae	*Urochroa bougueri*				1	14	6	15	5	18	7	22	7	18	8	25	9	33	8	29	11
Trochilidae	*Patagona gigas*				1	6	13	7	14	5	13	10	11	9	17	12	18	16	18	17	15
Trochilidae	*Aglaeactis cupripennis*				1	11	2	14	2	10	2	20	2	14	6	22	6	35	6	27	5
Trochilidae	*Aglaeactis castelnaudii*				1	16	0	14	1	10	4	14	9	25	1	16	8	21	8	17	11
Trochilidae	*Aglaeactis aliciae*	T		x	1	0	6x	0	6x	0	6x	0	6x	0	6x	0	6x	0	6x	0	6x
Trochilidae	*Aglaeactis pamela*			x	1	12	0x	12	0x	12	0x	12	0x	12	0x	12	0x	12	0x	12	0x
Trochilidae	*Lafresnaya lafresnayi*		H		1	0	3	0	3	0	4	0	3	0	5	0	7	0	8	0	5
Trochilidae	*Pterophanes cyanopterus*				1	18	6	21	6	16	7	28	5	25	7	33	8	34	9	35	7
Trochilidae	*Coeligena coeligena*				1	20	7	18	6	15	7	23	6	26	9	26	10	32	11	31	8
Trochilidae	*Coeligena wilsoni*				1	15	3	10	4	21	4	13	0	16	3	11	4	22	4	23	0
Trochilidae	*Coeligena prunellei*	T		x	1	11	0x	11	0x	11	0x	11	0x	11	0x	11	0x	11	0x	11	0x
Trochilidae	*Coeligena torquata*				1	18	5	18	5	18	6	26	6	27	8	25	11	34	12	34	8

Family	Species	IUCN	Ag	R	Range Source	Percentage loss in geographic range 2050 TG H	C	AM H	C	GO H	C	OS H	C	2100 TG H	C	AM H	C	GO H	C	OS H	C
Trochilidae	*Coeligena inca*				14	14	9	14	6	10	8	19	5	15	10	20	13	24	15	27	11
Trochilidae	*Coeligena phalerata*			x	1	6	0x	7	0x	19	0x	6	0x	19	0x	13	0x	19	0x	13	0x
Trochilidae	*Coeligena bonapartei*			x	1	24	0x	24	0x	26	0x	23	0x	27	0x	27	0x	32	0x	24	0x
Trochilidae	*Coeligena eos*			x	14	21	1x	33	6x	24	6x	56	6x	33	26x	47	31x	46	44x	85	6x
Trochilidae	*Coeligena helianthea*				1	17	1	21	1	13	1	16	1	23	1	27	1	29	7	23	4
Trochilidae	*Coeligena lutetiae*				1	12	9	11	10	19	10x	22	3	15	9	29	10x	43	11x	32	3x
Trochilidae	*Coeligena violifer*				1	9	7	11	4	7	6	12	5	14	8	14	7	15	8	19	6
Trochilidae	*Coeligena iris*				1	3	16	4	11	11	17	18	9	3	16	15	11	12	23x	18	9
Trochilidae	*Ensifera ensifera*				1	16	5	17	5	11	6	20	5	20	6	24	7	24	10	25	7
Trochilidae	*Sephanoides sephaniodes*				1	5	11	5	10	4	11	5	11	7	17	8	20	10	22	10	22
Trochilidae	*Boissonneaua flavescens*				1	16	3	20	5	14	3	25	3	24	5	28	8	28	14	32	5
Trochilidae	*Boissonneaua matthewsii*				1	17	7	20	5	24	6	37	5	27	7	35	8	43	6	52	6
Trochilidae	*Boissonneaua jardini*				1	12	9	9	9	19	9x	16	3	12	9	12	9	23	9x	25	3x
Trochilidae	*Heliangelus mavors*				1	32	3	49	5	15	5	46	5	48	25x	66	27x	49	48x	82	13x
Trochilidae	*Heliangelus amethysticollis*				1	13	7	13	6	16	7	21	7	21	7	22	9	29	9	27	7
Trochilidae	*Heliangelus clarisse*				14	31	1	43	4	16	4	43	3	42	10	54	13	43	28	58	8
Trochilidae	*Heliangelus strophianus*			x	1	26	12x	20	14x	42	14x	35	0x	26	12x	25	14x	46	14x	55	0x
Trochilidae	*Heliangelus exortis*				1	14	4	16	8	14	4	15	8	19	6	22	9	17	9	20	9
Trochilidae	*Heliangelus micraster*			x	14	10	21x	15	14x	0	27x	0	27x	10	21x	15	14x	11	34x	11	29x
Trochilidae	*Heliangelus viola*			x	1	0	8x	0	8x	0	8x	0	8x	0	8x	0	10x	0	10x	0	10x
Trochilidae	*Heliangelus regalis*	T		x	1	0	0x	0	0x	0	0x	0	0x	0	0x	0	0x	0	0x	0	0x
Trochilidae	*Eriocnemis nigrivestis*	T		x	1	8	57x	30	57x	30	57x	87	0x	8	57x	30	57x	38	57x	87	0x
Trochilidae	*Eriocnemis vestitus*				1	26	4	28	4	14	6	33	6	30	9	40	12	43	18	41	10
Trochilidae	*Eriocnemis godini*	T		x	1	53	0x	23	0x	78	0x	23	0x	53	0x	24	0x	78	0x	76	0x
Trochilidae	*Eriocnemis luciani*			x	1	25	13x	19	20x	27	21x	27	11x	26	13x	24	20x	44	25x	49	15x
Trochilidae	*Eriocnemis sapphiropygia*				14	22	6	33	5	28	6	43	4	35	6	46	7	44	6	55	4
Trochilidae	*Eriocnemis cupreoventris*				1	30	0	38	0	15	0	45	0	38	8	45	14	39	26	56	6
Trochilidae	*Eriocnemis mosquera*				1	9	8	9	9	14	9	15	4	10	9	12	9	20	9	21	4
Trochilidae	*Eriocnemis glaucopoides*				1	11	8	7	11	6	11	19	2	13	10	9	15	9	17	25	2
Trochilidae	*Eriocnemis mirabilis*	T		x	1	0	0x	0	0x	0	0x	0	0x	0	0x	0	0x	0	0x	0	0x
Trochilidae	*Eriocnemis alinae*				1	10	4	12	4	19	4	34	0	18	5	26	6	36	6	45	1
Trochilidae	*Eriocnemis derbyi*			x	1	15	7x	20	7x	30	6x	37	1x	15	14x	28	7x	30	6x	38	1x
Trochilidae	*Haplophaedia aureliae*				1	21	3	19	3	16	1	34	3	28	7	35	6	35	5	45	8
Trochilidae	*Haplophaedia assimilis*				14	9	7	15	7	18	7	25	2	16	7	23	8	24	7	31	2
Trochilidae	*Haplophaedia lugens*			x	1	29	11x	19	12x	40	12x	31	0x	29	11x	21	12x	43	12x	51	0x
Trochilidae	*Urosticte benjamini*				1	14	6	13	7	11	9	20	8	18	8	27	7	34	11	25	10
Trochilidae	*Urosticte ruficrissa*				1	20	10	19	9	13	13	27	11	25	11	34	14	49	16	34	16
Trochilidae	*Ocreatus underwoodii*				1	14	4	14	4	16	4	24	2	22	6	27	8	31	8	37	5
Trochilidae	*Lesbia victoriae*		H		1	0	4	0	4	0	5	0	3	0	6	0	5	0	11	0	7
Trochilidae	*Lesbia nuna*				1	14	9	10	12	12	12	13	11	16	13	15	18	23	20	22	16
Trochilidae	*Sappho sparganura*				1	4	16	4	18	1	18	4	15	5	21	5	28	5	28	5	24
Trochilidae	*Polyonymus caroli*				1	4	33	15	30	11	31	17	24	6	47	15	44	23	43	24	36
Trochilidae	*Ramphomicron microrhync*				1	7	5	13	3	11	4	12	5	10	10	15	10	18	10	19	7
Trochilidae	*Ramphomicron dorsale*			x	1	13	0x	29	0x	42	0x	13	0x	42	0x	29	0x	42	0x	42	0x

Family	Species	IUCN	Ag	R	Range Source	Percentage loss in geographic range 2050								2100							
						TG		AM		GO		OS		TG		AM		GO		OS	
						H	C	H	C	H	C	H	C	H	C	H	C	H	C	H	C
Trochilidae	*Metallura williami*				1	20	6	18	3	13	6	21	1	22	7	39	6	47	11	27	6
Trochilidae	*Metallura baroni*	T		x	1	96	0x	37	0x	0	0x	0	0x	96	0x	78	0x	63	37x	22	37x
Trochilidae	*Metallura odomae*			x	1	20	25x	15	22x	0	40x	0	38x	20	25x	15	22x	5	40x	5	38x
Trochilidae	*Metallura theresiae*				1	12	2	15	2	15	2	31	0	26	2	22	7	42	2	44	0
Trochilidae	*Metallura eupogon*				1	28	0	27	0	22	0	46	2	45	0	39	5	55	0	53	2
Trochilidae	*Metallura aeneocauda*				1	11	2	12	1	7	3	12	3	13	2	16	3	14	5	14	5
Trochilidae	*Metallura phoebe*				1	7	25	10	30	10	26	12	24	8	35	13	40	17	36	17	32
Trochilidae	*Metallura tyrianthina*				1	14	4	15	5	14	5	18	3	17	7	21	10	24	10	23	7
Trochilidae	*Metallura iracunda*	T		x	1	10	0x	10	0x	3	0x	0	0x	12	0x	12	0x	12	0x	10	0x
Trochilidae	*Chalcostigma ruficeps*				1	18	5	18	3	14	6	26	9	27	5	26	8	28	6	29	9
Trochilidae	*Chalcostigma olivaceum*				1	7	11	11	6	7	11	2	12	8	12	13	7	14	12	9	12
Trochilidae	*Chalcostigma stanleyi*				1	15	2	13	3	13	3	19	2	19	3	24	5	33	5	26	4
Trochilidae	*Chalcostigma heteropogon*				1	17	0	13	2	5	2	11	2	19	0	18	2	13	7	16	3
Trochilidae	*Chalcostigma herrani*				1	18	3	11	5	9	7	11	4	21	4	28	8	38	10	17	9
Trochilidae	*Oxypogon guerinii*				1	29	4	36	2	21	4	28	2	35	12	41	10	31	22	48	5
Trochilidae	*Opisthoprora euryptera*				1	13	8	10	7	12	10	21	7	18	9	22	9	33	14	26	7
Trochilidae	*Taphrolesbia griseiventris*	T		x	1	0	7x	0	7x	3	7x	0	7x	2	7x	0	7x	7	8x	7	7x
Trochilidae	*Aglaiocercus kingi*				1	17	5	18	4	17	5	27	4	24	7	28	8	36	8	36	6
Trochilidae	*Aglaiocercus berlepschi*			x	1	0	0x	0	0x	0	0x	0	0x	0	0x	11	0x	11	0x	11	0x
Trochilidae	*Aglaiocercus coelestis*				1	10	2	5	5	5	0	5	5	15	2	5	7	5	7	7	7
Trochilidae	*Oreonympha nobilis*			x	1	9	0x	5	0x	10	0x	12	5x	16	0x	10	7x	17	0x	12	5x
Trochilidae	*Augastes lumachellus*			x	1	1	4x	1	0x	1	5x	1	0x	1	11x	6	0x	6	5x	6	0x
Trochilidae	*Augastes scutatus*				1	21	0	18	0	23	0	23	0	29	0	18	0	24	0	23	0
Trochilidae	*Augastes geoffroyi*				1	20	3	19	3	18	4	26	4	27	6	30	8	34	8	33	5
Trochilidae	*Heliothryx barroti*				1	18	6	21	8	15	9	27	6	23	7	27	10	28	13	34	8
Trochilidae	*Heliothryx aurita*		H		1	0	13	0	12	0	13	0	10	0	16	0	15	0	16	0	13
Trochilidae	*Heliactin bilophum*				1	6	20	3	15	4	18	10	13	9	25	7	17	11	21	17	15
Trochilidae	*Loddigesia mirabilis*	T		x	1	0	0x	0	0x	0	0x	0	0x	0	0x	0	0x	0	0x	0	0x
Trochilidae	*Heliomaster constantii*				1	12	8	25	5	15	7	33	4	15	10	25	16	19	11	35	6
Trochilidae	*Heliomaster longirostris*				1	7	11	5	11	6	11	11	9	11	13	10	14	16	15	19	12
Trochilidae	*Heliomaster squamosus*				1	9	16	7	10	10	13	15	6	11	22	13	13	15	15	21	8
Trochilidae	*Heliomaster furcifer*				1	5	10	4	8	6	8	8	7	8	12	7	13	11	14	13	12
Trochilidae	*Rhodopis vesper*				1	3	20	4	22	5	22	9	17	3	30	9	39	13	41	18	30
Trochilidae	*Thaumastura cora*		H		1	0	25	0	28	0	29	0	22	0	37	0	47	0	48	0	38
Trochilidae	*Philodice bryantae*		H		1	0	10	0	21	0	21	0	21	0	10	0	32	0	30	0	21
Trochilidae	*Philodice mitchellii*		H		1	0	4	0	4	0	3	0	2	0	5	0	4	0	7	0	4
Trochilidae	*Doricha enicura*				1	7	2	18	2	11	2	23	2	11	2	20	9	14	3	23	2
Trochilidae	*Doricha eliza*			x	1	0	7x	10	0x	10	7x	10	0x	0	15x	10	28x	10	7x	10	7x
Trochilidae	*Tilmatura dupontii*				1	13	6	27	2	17	7	31	1	16	7	28	7	22	9	33	3
Trochilidae	*Microstilbon burmeisteri*				1	13	7	10	5	13	3	13	4	15	8	12	5	15	4	17	4
Trochilidae	*Calothorax lucifer*				1	5	20	11	14	7	16	13	15	6	22	11	26	9	23	15	21
Trochilidae	*Calothorax pulcher*			x	1	8	0x	12	0x	9	0x	9	0x	8	0x	12	8x	9	0x	9	0x
Trochilidae	*Archilochus colubris*				1	11	21	16	12	15	16	15	9	16	24	22	15	28	20	22	10
Trochilidae	*Archilochus alexandri*				1	1	20	4	17	2	19	4	15	2	25	4	31	4	33	6	25

Family	Species	IUCN	Ag	R	Range Source	Percentage loss in geographic range 2050 TG H	C	AM H	C	GO H	C	OS H	C	2100 TG H	C	AM H	C	GO H	C	OS H	C
Trochilidae	*Calypte anna*				1	5	37	4	36	5	38	5	30	5	45	6	53	9	59	9	48
Trochilidae	*Calypte costae*		H		1	0	15	0	16	0	19	0	16	0	19	0	27	0	34	0	28
Trochilidae	*Calliphlox evelynae*			x	1	31	7x	64	7x	63	9x	66	0x	49	7x	64	9x	67	9x	82	0x
Trochilidae	*Calliphlox amethystina*				1	6	13	4	11	6	12	10	10	10	16	9	15	16	16	19	13
Trochilidae	*Mellisuga helenae*				1	8	6	24	6	13	5	28	3	14	6	26	8	17	5	33	3
Trochilidae	*Mellisuga minima*				1	17	10	22	9	17	12	35	0	20	13	22	10	17	12	35	0
Trochilidae	*Stellula calliope*				1	3	22	8	15	5	20	8	13	4	28	9	26	11	33	16	20
Trochilidae	*Atthis heloisa*				1	16	7	32	1	18	4	33	1	19	7	34	11	25	10	36	4
Trochilidae	*Atthis ellioti*				1	9	2	25	2	17	2	26	2	10	2	26	5	21	2	30	2
Trochilidae	*Myrtis fanny*				1	8	20	9	20	12	22	17	13	9	26	15	28	24	29	26	19
Trochilidae	*Eulidia yarrellii*	T			1	0	21	0	21	0	24	4	18	0	32	7	70x	35	50x	29	49x
Trochilidae	*Myrmia micrura*				1	10	14	2	11	6	14	7	10	15	14	13	14	15	18	9	14
Trochilidae	*Acestrura mulsant*				1	19	6	16	5	14	7	20	5	21	7	23	6	27	8	24	6
Trochilidae	*Acestrura bombus*	T			1	9	11	12	8	8	14	14	12	10	12	23	9	28	15	20	13
Trochilidae	*Acestrura heliodor*				1	10	4	13	4	16	4	34	0	13	14	36	9	41	13	42	1
Trochilidae	*Acestrura astreans*			x	1	9	0x	34	0x	43	0x	9	0x	43	0x	34	0x	43	0x	43	0x
Trochilidae	*Acestrura berlepschi*	T		x	1	13	16x	5	16x	16	16x	21	0x	13	16x	5	16x	16	16x	33	0x
Trochilidae	*Chaetocercus jourdanii*				1	28	3	25	5	21	5	28	3	31	13	37	15	35	21	42	6
Trochilidae	*Selasphorus platycercus*				1	3	13	7	10	4	12	9	9	4	15	8	19	7	21	11	15
Trochilidae	*Selasphorus rufus*				1	4	23	6	20	5	22	7	19	5	29	8	30	10	33	14	26
Trochilidae	*Selasphorus sasin*				1	1	58	2	40	1	59	3	27	1	60	2	50	1	67	7	33
Trochilidae	*Selasphorus flammula*		H		1	0	13	0	22x	0	22x	0	18x	0	13	0	23x	0	22x	0	18x
Trochilidae	*Selasphorus scintilla*			x	1	9	18x	30	18x	24	18x	38	18x	24	18x	30	32x	48	32x	67	18x
Trochilidae	*Selasphorus ardens*	T		x	1	27	0x	68	0x	2	0x	68	0x	42	0x	68	0x	2	0x	68	0x
Musophagidae	*Tauraco persa*				1	27	5	17	7	24	7	42	4	38	6	25	8	43	8	48	4
Musophagidae	*Tauraco schuettii*				1	39	6	24	10	38	9	60	5	56	8	34	13	65	10	67	6
Musophagidae	*Tauraco schalowi*				1	58	0	44	1	59	0	63	0	62	1	46	4	62	0	63	0
Musophagidae	*Tauraco fischeri*				1	4	0	4	0	4	0	4	0	4	0	4	0	4	0	4	0
Musophagidae	*Tauraco livingstonii*				1	48	0	40	1	48	0	48	0	48	1	43	2	48	0	48	0
Musophagidae	*Tauraco corythaix*				1	4	4	4	4	4	4	4	4	4	6	4	6	5	4	5	4
Musophagidae	*Tauraco bannermani*	T			1	25	13x	4	15x	13	20x	11	27x	25	17x	25	17x	35	20x	11	30x
Musophagidae	*Tauraco erythrolophus*				1	66	0	55	1	66	0	67	0	66	0	59	1	66	0	67	0
Musophagidae	*Tauraco macrorhynchus*				1	26	5	14	7	21	6	43	4	40	6	23	9	45	7	50	5
Musophagidae	*Tauraco leucotis*				1	55	1	33	2	58	1	58	1	58	1	43	6	58	3	58	3
Musophagidae	*Tauraco ruspolii*	T			1	45	0	43	0	45	0	45	0	45	0	43	0	45	0	45	0
Musophagidae	*Tauraco hartlaubi*				1	30	1	27	3	31	0	31	0	30	1	28	3	32	0	31	0
Musophagidae	*Tauraco leucolophus*				1	34	5	14	11	40	7	50	4	45	7	21	14	52	8	52	6
Musophagidae	*Musophaga johnstoni*				1	30	3	27	6	33	5	44	0	44	3	30	16	40	7	48	0
Musophagidae	*Musophaga porphyreoloph*				1	42	0	36	1	42	0	42	0	42	2	39	4	43	1	43	1
Musophagidae	*Musophaga violacea*				1	24	3	13	4	30	4	38	3	34	4	19	9	43	6	40	5
Musophagidae	*Musophaga rossae*				1	50	5	33	7	49	5	60	3	57	6	38	11	58	6	62	4
Musophagidae	*Corythaixoides concolor*				1	34	1	28	2	34	1	34	1	34	3	29	8	34	2	34	2
Musophagidae	*Corythaixoides personatus*				1	38	2	30	4	40	1	39	1	38	3	34	6	41	1	41	2
Musophagidae	*Corythaixoides leucogaster*				1	41	4	15	21	47	1	47	1	44	4	19	25	47	2	47	2

Family	Species	IUCN	Ag	R	Range Source	2050 TG H	2050 TG C	2050 AM H	2050 AM C	2050 GO H	2050 GO C	2050 OS H	2050 OS C	2100 TG H	2100 TG C	2100 AM H	2100 AM C	2100 GO H	2100 GO C	2100 OS H	2100 OS C
Musophagidae	*Crinifer piscator*				1	13	3	8	4	15	3	22	2	20	4	11	6	24	5	23	4
Musophagidae	*Crinifer zonurus*				1	28	2	16	5	31	1	31	1	30	2	20	7	32	1	32	1
Musophagidae	*Corythaeola cristata*				1	34	7	21	9	32	9	54	5	48	8	30	13	55	11	60	6
Tytonidae	*Tyto tenebricosa*				1	6	4	8	3	18	2	18	2	12	7	15	7	29	6	30	4
Tytonidae	*Tyto multipunctata*				1	0	49	0	31	0	53	2	21	0	61	0	52	0	54	2	22
Tytonidae	*Tyto inexspectata*	T			1	11	29	27	14	2	28	24	18	18	29	31	29	13	39	32	33
Tytonidae	*Tyto nigrobrunnea*	T		x	1	3	0x	19	0x	24	0x	0	0x	3	0x	19	0x	24	0x	0	0x
Tytonidae	*Tyto sororcula*			x	1	8	3x	7	3x	16	0x	24	4x	27	3x	7	3x	25	0x	30	4x
Tytonidae	*Tyto manusi*	T		x	1	41	0x	0	0x	58	0x	95	0x	95	0x	0	0x	99	0x	95	0x
Tytonidae	*Tyto aurantia*				1	7	0	7	0	51	0	46	0	23	0	27	0	73	0x	59	0
Tytonidae	*Tyto novaehollandiae*				1	1	14	1	10	2	13	2	9	2	16	2	14	3	17	3	10
Tytonidae	*Tyto rosenbergii*				1	14	14	26	9	19	14	16	13	17	15	30	22	28	30	23	28
Tytonidae	*Tyto soumagnei*	T			1	70	0	46	1	72	0	72	0	72	0	71	1	72	0	72	0
Tytonidae	*Tyto alba*		H		1	0	9	0	8	0	8	0	6	0	11	0	12	0	13	0	9
Tytonidae	*Tyto glaucops*				1	17	13	24	12	18	16	36	0	17	18	24	13	18	16	36	0
Tytonidae	*Tyto capensis*				1	28	2	22	2	27	2	31	1	30	3	25	7	31	3	32	2
Tytonidae	*Tyto longimembris*				1	5	13	5	9	6	11	7	7	6	15	6	13	7	15	10	8
Tytonidae	*Phodilus prigoginei*	T			1	12	0	16	1	22	0	34	0	27	0	16	1	37	0	34	0
Tytonidae	*Phodilus badius*				1	18	5	21	5	19	6	22	6	23	7	24	8	27	9	29	8
Strigidae	*Otus sagittatus*	T			1	11	10	12	10	12	10	22	10	16	11	16	10	26	10	27	10
Strigidae	*Otus rufescens*				1	13	2	16	2	15	2	18	2	20	3	22	3	28	3	28	2
Strigidae	*Otus icterorhynchus*				1	38	7	27	8	36	7	66	5	57	7	36	8	67	8	73	5
Strigidae	*Otus ireneae*	T		x	1	5	0x	5	0x	5	0x	5	0x	5	0x	5	0x	5	0x	5	0x
Strigidae	*Otus balli*			x	1	10	0x	10	0x	10	0x	10	0x	10	0x	20	0x	20	0x	20	0x
Strigidae	*Otus spilocephalus*				1	18	8	21	7	17	9	21	8	21	10	24	10	25	12	28	11
Strigidae	*Otus umbra*			x	1	0	0x	0	0x	0	0x	0	0x	0	0x	0	0x	0	0x	0	0x
Strigidae	*Otus angelinae*	T		x	1	0	0x	0	0x	0	0x	0	0x	0	0x	0	0x	0	0x	0	0x
Strigidae	*Otus manadensis*				1	14	13	27	9	19	13	16	12	16	14	30	21	28	29	23	26
Strigidae	*Otus collari*			x	14	0	0x	0	0x	0	0x	0	0x	0	0x	0	0x	0	0x	0	0x
Strigidae	*Otus alius*			x	14	47	0x	100	0x	100	0x	47	0x	47	0x	100	0x	100	0x	100	0x
Strigidae	*Otus longicornis*				1	6	4	18	0	10	5	6	5	6	13	31	9x	16	20x	20	6
Strigidae	*Otus mindorensis*			x	1	0	5x	3	1x	0	5x	41	4x	0	23x	21	2x	3	23x	41	7x
Strigidae	*Otus mirus*				1	18	14	25	18	7	18	17	19	28	17	25	18	24	22	33	19
Strigidae	*Otus hartlaubi*	T		x	1	0	⊢02x	0	⊢02x	100	0x	100	0x	0	⊢02x	0	⊢02x	100	0x	100	0x
Strigidae	*Otus brucei*				1	4	12	4	12	5	13	6	12	7	15	5	21	8	23	11	22
Strigidae	*Otus flammeolus*				1	5	20	12	16	7	19	15	14	7	24	13	26	11	30	20	22
Strigidae	*Otus scops*				1	6	14	6	13	7	17	8	12	10	17	9	15	12	19	15	13
Strigidae	*Otus sunia*				1	15	5	16	5	16	6	17	6	17	8	18	9	18	9	23	8
Strigidae	*Otus senegalensis*				1	28	3	17	6	30	3	34	2	32	4	21	10	34	5	35	4
Strigidae	*Otus elegans*			x	1	30	5x	5	6x	11	6x	0	12x	36	5x	34	9x	36	9x	0	12x
Strigidae	*Otus mantananensis*			x	1	44	4x	26	5x	0	5x	26	5x	44	4x	62	31x	0	57x	52	5x
Strigidae	*Otus magicus*				1	17	3	19	4	15	4	18	2	27	9	19	10	28	6	33	2
Strigidae	*Otus beccarii*	T		x	1	0	9x	0	9x	0	9x	0	9x	0	9x	0	9x	0	9x	0	9x
Strigidae	*Otus alfredi*	T		x	1	24	6x	26	16x	8	16x	0	16x	24	6x	26	16x	8	16x	26	16x

Family	Species	IUCN	Ag	R	Range Source	2050 TG H	2050 TG C	2050 AM H	2050 AM C	2050 GO H	2050 GO C	2050 OS H	2050 OS C	2100 TG H	2100 TG C	2100 AM H	2100 AM C	2100 GO H	2100 GO C	2100 OS H	2100 OS C
Strigidae	*Otus enganensis*			x	1	0	0x	0	0x	0	0x	0	0x	0	0x	0	0x	0	0x	0	0x
Strigidae	*Otus rutilus*				1	49	0	27	5	50	0	50	0	50	0	37	5	50	0	50	0
Strigidae	*Otus pembaensis*			x	1	0	0x	0	0x	0	0x	0	0x	0	0x	0	0x	0	0x	0	0x
Strigidae	*Otus capnodes*	T		x	1	0	0x	0	0x	0	0x	0	0x	0	0x	0	0x	0	0x	0	0x
Strigidae	*Otus moheliensis*	T		x	14	100	0x	100	0x	100	0x	100	0x	100	0x	100	0x	100	0x	100	0x
Strigidae	*Otus pauliani*	T		x	1	0	0x	0	0x	0	0x	0	0x	0	0x	0	0x	0	0x	0	0x
Strigidae	*Otus brookii*				1	21	9	24	10	23	8	26	8	26	9	31	11	44	8	33	8
Strigidae	*Otus bakkamoena*				1	17	3	20	3	19	3	24	2	20	5	22	6	21	8	34	2
Strigidae	*Otus lettia*				1	11	4	11	4	10	5	11	5	12	6	13	7	12	8	14	6
Strigidae	*Otus lempiji*				1	12	2	15	3	14	3	16	3	20	3	21	3	27	3	25	3
Strigidae	*Otus semitorques*				1	7	18	7	17	9	19	7	18	9	28	9	33	12	32	10	31
Strigidae	*Otus mentawi*			x	1	0	0x	30	0x	30	0x	1	0x	24	0x	30	0x	45	0x	15	0x
Strigidae	*Otus fuliginosus*			x	1	18	26x	19	28x	25	14x	19	23x	18	26x	19	45x	52	30x	30	49x
Strigidae	*Otus megalotis*				1	14	12	24	11	13	12	21	12	20	18	30	14	26	21	34	13
Strigidae	*Otus silvicola*			x	1	19	7x	12	12x	4	12x	5	8x	19	32x	12	36x	23	18x	21	8x
Strigidae	*Otus leucotis*				1	15	3	7	6	18	2	21	1	19	3	9	9	22	4	22	3
Strigidae	*Ptilopsis granti*				14	21	4	16	5	21	4	23	4	22	6	17	11	23	7	23	6
Strigidae	*Otus kennicottii*				1	3	19	7	16	4	18	9	15	4	23	8	28	7	31	12	25
Strigidae	*Otus seductus*				1	13	3	39	0	22	3	43	0	15	3	39	13	27	3	45	0
Strigidae	*Otus cooperi*				1	9	2	17	0	17	1	19	0	11	2	17	5	20	4	20	0
Strigidae	*Otus asio*				1	13	24	21	13	20	17	21	9	21	27	29	18	36	22	32	11
Strigidae	*Otus trichopsis*				1	10	8	24	4	11	8	29	4	12	10	25	14	16	14	33	9
Strigidae	*Otus choliba*				1	7	12	5	10	7	11	11	9	11	14	10	14	16	15	19	12
Strigidae	*Otus koepckeae*				1	5	6	12	5	9	6	11	4	5	12	12	9	14	9	14	7
Strigidae	*Otus roboratus*				1	13	9	10	7	5	11	9	9	16	9	14	12	17	19	14	16
Strigidae	*Otus clarkii*				1	23	7	29	14	19	15	39	10	31	9	32	20	34	20	53	12
Strigidae	*Otus barbarus*			x	1	4	0x	5	0x	4	0x	5	0x	5	0x	9	6x	5	4x	5	0x
Strigidae	*Otus ingens*				1	17	6	16	6	15	5	29	5	27	7	29	9	33	8	44	6
Strigidae	*Otus colombianus*				14	11	7	8	6	16	6	14	1	15	8	10	6	18	6	20	1
Strigidae	*Otus huberi*				1	38	8	33	3	27	8	52	10x	52	8	42	14	60	8x	61	10x
Strigidae	*Otus petersoni*				14	3	7	9	6	13	9	10	9	7	7	20	6	21	10	35	10
Strigidae	*Otus watsonii*				1	5	9	3	8	4	9	7	8	8	10	7	10	13	11	15	10
Strigidae	*Otus atricapillus*				1	14	11	13	8	17	7	20	7	21	18	20	15	33	12	34	13
Strigidae	*Otus guatemalae*				1	9	8	18	5	10	7	27	5	12	10	21	19	15	12	31	7
Strigidae	*Otus hoyi*				1	6	3	7	0	3	2	7	2	7	3	8	1	7	3	8	2
Strigidae	*Otus vermiculatus*				1	12	9	14	10	11	11	18	10	18	12	21	15	20	18	27	16
Strigidae	*Otus sanctaecatarinae*				1	20	1	17	2	27	1	29	1	29	3	33	5	44	2	48	3
Strigidae	*Otus lawrencii*				1	9	15x	28	15x	14	10x	40	7x	19	15x	31	17x	20	11x	42	7x
Strigidae	*Otus nudipes*			x	1	24	0x	24	0x	24	0x	24	0x	24	0x	24	0x	24	0x	24	0x
Strigidae	*Otus albogularis*				1	16	6	15	5	14	6	25	5	22	8	24	10	30	10	33	7
Strigidae	*Mimizuku gurneyi*	T			1	18	16	26	18	8	18	19	18	28	19	26	18	24	23	35	18
Strigidae	*Bubo virginianus*				1	6	16	8	13	8	15	10	11	9	20	11	20	14	21	15	16
Strigidae	*Bubo magellanicus*				14	5	11	5	11	4	12	6	11	6	16	7	20	9	21	11	18
Strigidae	*Bubo bubo*		H		1	0	11	0	11	0	12	0	10	0	15	0	15	0	17	0	15

Family	Species	IUCN	Ag	R	Range Source	Percentage loss in geographic range 2050 TG H	TG C	AM H	AM C	GO H	GO C	OS H	OS C	2100 TG H	TG C	AM H	AM C	GO H	GO C	OS H	OS C
Strigidae	*Bubo bengalensis*				1	24	5	27	5	26	5	31	4	27	7	31	8	30	9	45	5
Strigidae	*Bubo ascalaphus*				1	2	2	2	2	3	2	3	2	3	2	2	3	3	3	3	3
Strigidae	*Bubo capensis*				1	15	4	12	5	15	4	15	4	15	5	13	7	16	6	16	6
Strigidae	*Bubo africanus*				1	31	3	23	4	31	3	35	2	34	5	26	8	35	5	36	4
Strigidae	*Bubo cinerascens*				14	24	4	10	9	27	3	32	2	30	4	14	12	33	6	34	4
Strigidae	*Bubo poensis*				1	35	5	21	8	32	7	55	4	52	6	31	10	58	8	64	4
Strigidae	*Bubo vosseleri*	T			1	70	0x	55	0x	70	0x	70	0x	70	0x	67	0x	70	0x	70	0x
Strigidae	*Bubo nipalensis*				1	23	6	28	6	25	7	27	6	27	8	30	8	29	9	35	8
Strigidae	*Bubo sumatranus*				1	18	6	19	6	19	6	23	7	24	8	26	7	36	7	32	7
Strigidae	*Bubo shelleyi*				1	35	11	19	12	29	13	58	7	50	12	28	17	53	15	65	8
Strigidae	*Bubo lacteus*				1	35	3	22	7	36	3	40	2	38	4	25	11	40	4	40	3
Strigidae	*Bubo coromandus*				1	14	4	16	4	14	4	19	3	16	6	18	10	15	12	27	5
Strigidae	*Bubo leucostictus*				1	30	7	19	9	26	8	52	4	46	8	28	10	51	9	60	5
Strigidae	*Bubo philippensis*	T			1	13	11	24	10	13	11	22	11	19	18	30	14	27	20	34	12
Strigidae	*Nyctea scandiaca*		H		1	0	14	0	14	0	14	0	14	0	22	0	29	0	31	0	27
Strigidae	*Strix seloputo*				1	10	5	15	5	10	5	13	5	13	7	17	9	18	9	18	9
Strigidae	*Strix ocellata*				1	14	2	18	3	18	2	21	1	17	3	21	5	22	6	33	2
Strigidae	*Strix leptogrammica*				1	17	4	20	4	19	4	21	4	21	5	23	5	25	6	28	5
Strigidae	*Strix aluco*				1	10	13	11	11	12	14	12	10	14	16	13	14	17	17	17	12
Strigidae	*Strix butleri*				1	0	2	0	1	0	1	1	1	1	2	1	1	1	2	1	1
Strigidae	*Strix occidentalis*				1	5	20	9	16	6	18	12	14	6	23	11	28	10	32	17	25
Strigidae	*Strix varia*				1	8	19	14	12	12	16	13	10	13	23	18	18	21	22	20	14
Strigidae	*Strix fulvescens*				1	10	2	22	2	16	2	22	2	12	2	23	7	19	2	25	3
Strigidae	*Strix hylophila*				1	17	8	15	8	23	6	24	7	25	14	27	13	38	9	39	12
Strigidae	*Strix rufipes*				1	5	8	4	8	4	9	5	8	7	14	7	17	8	18	10	18
Strigidae	*Strix chacoensis*				14	2	9	1	9	2	9	2	8	2	12	1	18	2	20	2	17
Strigidae	*Strix uralensis*				1	5	12	4	12	5	14	5	12	6	15	6	15	7	17	9	15
Strigidae	*Strix davidi*				1	15	0	16	0	15	1	15	1	15	2	17	3	16	2	16	2
Strigidae	*Strix nebulosa*				1	3	15	3	15	3	16	3	15	3	19	4	21	5	23	6	21
Strigidae	*Strix virgata*				1	7	10	7	10	7	11	12	10	11	13	11	14	17	14	20	12
Strigidae	*Strix nigrolineata*				1	14	4	17	4	14	5	21	3	17	5	22	9	22	10	26	6
Strigidae	*Strix huhula*				1	6	12	4	12	5	12	9	11	10	14	8	15	15	15	18	14
Strigidae	*Strix albitarsus*				1	16	4	19	3	18	4	29	4	24	5	31	8	34	7	42	5
Strigidae	*Strix woodfordii*				1	38	4	25	6	37	5	49	3	47	5	31	10	50	6	53	4
Strigidae	*Jubula lettii*				1	34	6	22	8	33	7	56	3	51	6	33	9	61	8	66	4
Strigidae	*Lophostrix cristata*				1	5	10	5	10	5	10	9	10	9	11	9	12	13	12	15	11
Strigidae	*Pulsatrix perspicillata*				1	8	12	6	11	7	11	12	9	11	15	10	14	16	15	19	12
Strigidae	*Pulsatrix koeniswaldiana*				1	20	11	17	10	25	8	29	9	27	18	29	16	44	11	45	15
Strigidae	*Pulsatrix melanota*				1	16	6	15	5	15	5	27	5	24	6	27	8	31	6	40	6
Strigidae	*Surnia ulula*				1	3	16	3	16	3	17	3	16	4	20	4	22	5	23	6	22
Strigidae	*Glaucidium passerinum*				1	3	17	4	16	4	17	5	16	5	21	5	21	8	22	9	19
Strigidae	*Glaucidium brodiei*				1	13	5	15	5	13	6	15	5	15	7	17	7	18	8	20	7
Strigidae	*Glaucidium perlatum*				1	27	3	17	7	29	3	33	2	32	4	20	11	34	4	34	4
Strigidae	*Glaucidium californicum*				1	5	24	10	18	7	22	12	16	6	32	12	33	13	38	20	26

Family	Species	IUCN	Ag	R	Range Source	2050 TG H	2050 TG C	2050 AM H	2050 AM C	2050 GO H	2050 GO C	2050 OS H	2050 OS C	2100 TG H	2100 TG C	2100 AM H	2100 AM C	2100 GO H	2100 GO C	2100 OS H	2100 OS C
Strigidae	*Glaucidium gnoma*				1	10	9	25	4	12	8	34	4	13	10	26	14	17	13	39	6
Strigidae	*Glaucidium cobanense*				1	10	2	22	2	14	2	21	2	12	2	23	7	19	2	25	4
Strigidae	*Glaucidium hoskinsii*			x	1	0	2x	0	2x	0	0x	30	0x	0	2x	0	2x	0	0x	30	0x
Strigidae	*Glaucidium jardinii*				1	14	4	15	4	15	5	25	3	21	6	24	9	30	8	34	5
Strigidae	*Glaucidium costaricanum*				14	25	8	36	17	16	17	42	17	35	8	36	26	31	24	56	17x
Strigidae	*Glaucidium nubicola*	T			14	17	6	14	6	12	9	21	3	22	7	25	9	33	10	27	7
Strigidae	*Glaucidium bolivianum*			x	1	7	5x	7	3x	3	5x	6	4x	9	7x	7	5x	9	7x	11	5x
Strigidae	*Glaucidium palmarum*				1	13	11	30	7	16	11	38	6	19	14	30	19	22	19	42	11
Strigidae	*Glaucidium sanchezi*			x	1	0	11x	6	9x	6	9x	6	9x	0	11x	6	48x	6	9x	6	9x
Strigidae	*Glaucidium griseiceps*				1	17	7	26	9	17	10	35	7	23	8	32	15	27	14	45	9
Strigidae	*Glaucidium parkeri*				1	13	5	17	5	20	5	32	3	24	5	29	9	40	7	46	5
Strigidae	*Glaucidium hardyi*				1	5	14	3	14	3	15	8	14	8	15	6	16	14	17	15	16
Strigidae	*Glaucidium minutissimum*				1	14	13	13	10	19	10	21	9	22	19	21	16	33	14	35	16
Strigidae	*Glaucidium brasilianum*				1	8	11	7	10	7	11	13	9	12	14	11	15	17	15	20	12
Strigidae	*Glaucidium tucumanum*				1	0	0	0	0	0	1	0	0	0	0	0	7	0	11	0	7
Strigidae	*Glaucidium peruanum*				1	5	15	6	16	6	16	9	12	6	20	12	24	12	24	19	18
Strigidae	*Glaucidium nanum*				1	4	10	3	10	3	11	4	11	5	16	6	20	7	22	8	21
Strigidae	*Glaucidium siju*				1	9	15x	28	16x	15	10x	41	7x	19	15x	32	17x	20	11x	43	7x
Strigidae	*Glaucidium tephronotum*				1	39	6	24	10	40	8	58	4	54	6	33	11	60	9	65	4
Strigidae	*Glaucidium sjostedti*				1	33	4	22	5	37	6	50	4	51	5	36	7	64	8	63	4
Strigidae	*Glaucidium cuculoides*				1	15	4	17	4	15	4	16	4	17	5	18	6	18	7	19	6
Strigidae	*Glaucidium castanopterum*				1	6	0	0	0	0	0	0	0	8	0	5	0	10	0	5	1
Strigidae	*Glaucidium radiatum*				1	28	2	32	2	32	2	39	1	31	3	37	2	37	3	59	1
Strigidae	*Glaucidium castanonotum*			x	1	50	0x	43	0x	43	0x	44	0x	51	0x	51	0x	50	0x	51	0x
Strigidae	*Glaucidium capense*				1	28	1	23	2	28	1	29	1	28	3	25	7	28	1	29	1
Strigidae	*Glaucidium castaneum*				1	34	5	27	9	33	6	47	0	39	5	30	9	35	8	47	0
Strigidae	*Glaucidium albertinum*	T			1	20	9x	15	14x	21	9x	25	0x	20	9x	19	15x	21	13x	25	0x
Strigidae	*Xenoglaux loweryi*	T		x	1	0	0x	0	0x	0	0x	0	0x	0	0x	0	0x	0	0x	0	0x
Strigidae	*Micrathene whitneyi*				1	3	22	9	20	3	22	14	19	5	25	9	34	5	36	15	31
Strigidae	*Athene noctua*		H		1	0	9	0	8	0	9	0	7	0	11	0	13	0	14	0	11
Strigidae	*Athene brama*		H		1	0	4	0	4	0	5	0	4	0	7	0	9	0	11	0	6
Strigidae	*Athene blewitti*	T	H	x	1	0	0x	0	0x	0	0x	0	0x	0	0x	0	0x	0	7x	0	0x
Strigidae	*Speotyto cunicularia*		H		1	0	13	0	11	0	12	0	9	0	17	0	16	0	19	0	14
Strigidae	*Aegolius funereus*				1	4	17	4	16	5	17	5	16	5	21	6	23	8	24	9	22
Strigidae	*Aegolius acadicus*				1	7	21	12	16	11	19	13	14	10	25	15	25	17	28	20	21
Strigidae	*Aegolius ridgwayi*				1	19	4	24	6	24	6	38	4	23	4	29	14	33	9	43	4
Strigidae	*Aegolius harrisii*				1	10	14	8	11	10	12	15	9	14	20	14	16	20	18	23	13
Strigidae	*Ninox rufa*				1	3	9	3	6	12	8	11	3	6	11	8	11	20	11	17	5
Strigidae	*Ninox strenua*				1	7	11	11	6	17	6	17	5	14	19	14	13	23	16	29	7
Strigidae	*Ninox connivens*				1	1	17	2	12	4	15	4	9	3	20	3	17	6	20	6	11
Strigidae	*Ninox rudolfi*		H	x	1	0	0x	0	0x	0	0x	0	0x	0	25x	0	26x	0	25x	0	0x
Strigidae	*Ninox boobook*		H		1	0	14	0	9	0	12	0	6	0	16	0	13	0	16	0	8
Strigidae	*Ninox novaeseelandiae*		H		1	0	24	0	12	0	20	0	10	0	27	0	15	0	25	0	13
Strigidae	*Ninox scutulata*		H		1	0	4	0	4	0	5	0	4	0	6	0	7	0	8	0	6

Family	Species	IUCN	Ag	R	Range Source	Percentage loss in geographic range: 2050 TG H	2050 TG C	2050 AM H	2050 AM C	2050 GO H	2050 GO C	2050 OS H	2050 OS C	2100 TG H	2100 TG C	2100 AM H	2100 AM C	2100 GO H	2100 GO C	2100 OS H	2100 OS C
Strigidae	*Ninox affinis*			x	1	20	5x	57	0x	50	0x	31	0x	20	5x	63	0x	63	0x	63	0x
Strigidae	*Ninox superciliaris*				1	44	0	20	3	45	0	45	0	45	0	31	3	45	0	45	0
Strigidae	*Ninox philippensis*				1	15	11	24	10	13	12	21	12	21	17	30	13	25	21	34	12
Strigidae	*Ninox ochracea*				1	13	14	28	8	18	14	18	10	16	15	32	22	28	32	27	27
Strigidae	*Ninox squamipila*				1	13	1	18	1	21	0	22	1	29	1	18	1	32	0	37	1
Strigidae	*Ninox theomacha*				1	5	1	5	1	17	1	14	1	11	4	13	5	27	4	26	2
Strigidae	*Ninox meeki*			x	1	31	0x	0	0x	67	0x	89	0x	89	0x	0	0x	98	0x	89	0x
Strigidae	*Ninox punctulata*		H		1	0	13	0	10	0	13	0	13	0	14	0	21	0	27	0	25
Strigidae	*Ninox variegata*				1	5	0	7	0	50	0	43	0	26	0	23	0	71	0	56	0
Strigidae	*Ninox odiosa*				1	7	0	7	0	51	0	46	0	23	0	27	0	73	0x	59	0
Strigidae	*Ninox jacquinoti*				1	16	4	14	1	23	1	30	1	30	9	23	1	51	2	46	1
Strigidae	*Uroglaux dimorpha*				1	5	0	2	1	11	1	12	1	11	4	11	9	18	8	23	4
Strigidae	*Pseudoscops grammicus*			x	1	26	0x	26	3x	22	3x	51	3x	51	0x	26	3x	22	3x	51	3x
Strigidae	*Asio stygius*				1	8	13	7	11	7	12	13	10	11	17	11	15	16	17	20	13
Strigidae	*Asio otus*				1	7	17	9	14	9	16	9	13	10	20	12	20	15	22	15	18
Strigidae	*Asio abyssinicus*		H		1	0	3	0	5	0	3	0	0	0	3	0	6	0	4	0	0
Strigidae	*Asio madagascariensis*				1	66	1	35	9	68	1	68	1	68	1	51	9	68	1	68	1
Strigidae	*Asio clamator*				1	8	12	6	10	8	11	12	9	11	15	11	14	17	15	21	12
Strigidae	*Asio flammeus*				1	4	18	5	16	5	18	5	15	6	22	6	22	8	25	9	21
Strigidae	*Asio capensis*				1	26	2	19	3	26	2	27	2	27	3	22	7	28	4	28	3
Strigidae	*Nesasio solomonensis*	T		x	1	13	3x	11	0x	19	0x	27	0x	24	11x	28	0x	54	0x	40	0x
Aegothelidae	*Aegotheles crinifrons*				1	3	19	4	12	6	17	6	9	5	23	5	19	8	23	10	11
Aegothelidae	*Aegotheles insignis*				1	5	4	5	3	17	2	16	2	11	7	12	7	29	6	26	3
Aegothelidae	*Aegotheles tatei*				14	3	10	4	5	14	8	12	3	8	13	10	11	23	12	21	5
Aegothelidae	*Aegotheles cristatus*				1	0	13	1	10	1	12	1	8	1	15	1	13	2	15	2	10
Aegothelidae	*Aegotheles savesi*	T		x	1	53	38x	42	0x	42	38x	57	0x	53	38x	80	10x	47	48x	57	0x
Aegothelidae	*Aegotheles bennettii*				1	4	7	8	4	33	2	23	2	15	10	16	10	46	6	39	4
Aegothelidae	*Aegotheles wallacii*			x	1	16	0x	5	2x	23	2x	12	2x	22	1x	8	3x	38	2x	24	2x
Aegothelidae	*Aegotheles archboldi*			x	1	0	0x	0	0x	1	0x	1	0x	0	5x	21	0x	1	0x	21	0x
Aegothelidae	*Aegotheles albertisi*				1	8	1	2	0	36	0	16	0	12	6	5	11	53	9	38	6
Podargidae	*Podargus strigoides*			x	1	0	17x	0	0x	2	4x	0	0x	0	24x	0	0x	2	4x	0	0x
Podargidae	*Podargus papuensis*		H		1	0	34	0	17	0	34	0	12	0	38	0	31	0	39	0	14
Podargidae	*Podargus ocellatus*				1	7	9	7	11	16	11	17	7	14	14	12	15	28	15	24	8
Batrachostomida	*Batrachostomus auritus*				1	9	0	13	1	12	2	15	1	16	1	18	2	21	2	24	1
Batrachostomida	*Batrachostomus harterti*			x	1	20	0x	23	2x	24	2x	28	0x	21	0x	27	2x	41	2x	47	0x
Batrachostomida	*Batrachostomus septimus*				1	14	10	24	10	13	13	20	12	20	16	30	12	25	20	32	12
Batrachostomida	*Batrachostomus stellatus*				1	12	1	15	2	15	2	18	2	19	2	22	2	27	3	27	2
Batrachostomida	*Batrachostomus moniliger*		H		1	0	0	0	0	0	0	0	1	0	1	0	0	0	0	0	1
Batrachostomida	*Batrachostomus hodgsoni*				1	32	11	34	12	32	13	33	14	36	13	36	15	39	18	38	18
Batrachostomida	*Batrachostomus poliolophu*				1	35	0	14	1	21	0	35	0	43	0	25	1	39	0	41	0
Batrachostomida	*Batrachostomus javensis*				1	11	11	13	12	14	11	24	9	13	12	16	15	26	14	27	13
Batrachostomida	*Batrachostomus cornutus*				1	10	1	14	2	12	2	14	1	16	1	19	2	24	2	23	1
Steatornithidae	*Steatornis caripensis*				1	15	8	13	7	13	8	19	7	20	10	21	12	25	13	27	12
Nyctibiidae	*Nyctibius grandis*				1	7	11	6	11	6	11	11	10	11	13	10	14	17	14	19	13

Family	Species	IUCN	Ag	R	Range Source	Percentage loss in geographic range															
						2050								2100							
						TG		AM		GO		OS		TG		AM		GO		OS	
						H	C	H	C	H	C	H	C	H	C	H	C	H	C	H	C
Nyctibiidae	*Nyctibius aethereus*				1	5	11	4	11	4	11	8	10	9	12	7	13	14	13	16	13
Nyctibiidae	*Nyctibius jamaicensis*				1	9	5	19	3	14	4	25	2	11	6	22	13	18	7	28	4
Nyctibiidae	*Nyctibius griseus*				1	8	12	6	10	7	11	12	9	12	15	11	14	17	15	20	12
Nyctibiidae	*Nyctibius maculosus*				1	16	8	15	5	16	6	28	8	23	9	25	12	39	10	37	11
Nyctibiidae	*Nyctibius leucopterus*				1	4	8	3	8	3	8	6	8	8	9	6	9	13	10	14	9
Nyctibiidae	*Nyctibius bracteatus*				1	4	7	3	7	3	7	7	6	8	8	7	8	13	9	15	8
Eurostopodidae	*Eurostopodus argus*				1	0	12	0	10	0	11	0	8	0	13	0	12	0	14	0	10
Eurostopodidae	*Eurostopodus mystacalis*				1	6	25	9	15	12	20	14	11	10	31	12	26	18	29	24	15
Eurostopodidae	*Eurostopodus diabolicus*	T		x	1	20	34x	9	22x	9	34x	21	34x	20	34x	21	61x	34	60x	21	72x
Eurostopodidae	*Eurostopodus papuensis*				1	1	3	6	3	14	1	15	1	7	5	13	5	23	4	27	1
Eurostopodidae	*Eurostopodus archboldi*				1	21	2	6	0	27	0	23	0	24	11	6	7	37	5	40	3
Eurostopodidae	*Eurostopodus temminckii*				1	14	3	17	3	16	3	18	3	21	4	23	4	29	4	28	3
Eurostopodidae	*Eurostopodus macrotis*				1	21	8	25	8	21	10	24	9	25	11	28	13	27	16	30	13
Caprimulgidae	*Lurocalis semitorquatus*				1	6	12	5	12	6	13	10	12	10	15	9	16	16	17	19	15
Caprimulgidae	*Lurocalis rufiventris*				1	9	6	11	6	10	6	20	6	14	8	19	10	25	10	32	8
Caprimulgidae	*Chordeiles pusillus*				1	6	15	4	11	5	13	10	9	9	19	7	14	13	16	17	11
Caprimulgidae	*Chordeiles vielliardi*			x	1	2	52x	6	0x	0	22x	8	0x	2	54x	6	0x	8	22x	8	0x
Caprimulgidae	*Chordeiles rupestris*				1	6	11	4	12	4	12	9	11	10	13	9	14	15	15	17	13
Caprimulgidae	*Chordeiles acutipennis*		H		1	0	13	0	12	0	13	0	10	0	17	0	18	0	19	0	15
Caprimulgidae	*Chordeiles minor*		H		1	0	17	0	12	0	15	0	10	0	20	0	18	0	21	0	15
Caprimulgidae	*Chordeiles gundlachii*				1	10	11	24	10	15	9	32	4	17	11	25	12	18	9	35	4
Caprimulgidae	*Nyctiprogne leucopyga*				1	5	14	3	12	3	14	7	11	8	17	6	15	12	16	14	13
Caprimulgidae	*Podager nacunda*				1	7	13	5	11	6	12	11	9	11	16	9	15	15	16	18	13
Caprimulgidae	*Nyctidromus albicollis*				1	8	11	6	10	7	11	12	9	11	14	11	14	17	15	21	12
Caprimulgidae	*Phalaenoptilus nuttallii*				1	2	17	5	13	2	16	6	12	3	20	5	23	4	26	8	19
Caprimulgidae	*Siphonorhis brewsteri*			x	1	7	7x	11	3x	8	6x	14	0x	7	7x	11	3x	8	6x	14	0x
Caprimulgidae	*Nyctiphrynus mcleodii*				1	13	14	29	10	13	14	41	9	17	16	30	21	20	22	45	15
Caprimulgidae	*Nyctiphrynus yucatanicus*				1	6	3	17	0	14	3	23	2	8	5	19	12	15	10	30	6
Caprimulgidae	*Nyctiphrynus ocellatus*				1	7	15	5	15	6	16	10	14	11	18	9	19	16	20	18	18
Caprimulgidae	*Nyctiphrynus rosenbergi*				1	12	2	13	4	11	0	9	4	24	2	14	4	16	4	21	4
Caprimulgidae	*Caprimulgus carolinensis*				1	15	20	26	10	21	11	28	7	25	23	33	12	47	14	45	7
Caprimulgidae	*Caprimulgus rufus*				1	8	13	7	10	8	12	11	10	12	18	12	15	17	17	18	14
Caprimulgidae	*Caprimulgus cubanensis*				1	8	6	24	6	13	5	28	3	14	6	26	8	17	5	32	3
Caprimulgidae	*Caprimulgus ekmani*				14	11	13	13	11	13	11	24	0	11	13	13	11	13	11	24	0
Caprimulgidae	*Caprimulgus salvini*				1	4	8	9	3	6	3	10	5	4	11	11	25	9	3	10	5
Caprimulgidae	*Caprimulgus badius*				1	3	4	20	0	15	4	27	2	6	5	23	16	16	13	31	7
Caprimulgidae	*Caprimulgus sericocaudatu*				1	11	5	8	5	10	5	17	4	18	7	17	7	30	6	35	5
Caprimulgidae	*Caprimulgus ridgwayi*				1	14	10	28	5	16	9	37	4	18	11	29	16	21	17	41	9
Caprimulgidae	*Caprimulgus vociferus*				1	14	23	24	16	19	19	27	14	19	26	30	24	31	26	36	20
Caprimulgidae	*Caprimulgus noctitherus*	T		x	1	0	0x	0	0x	0	0x	0	0x	0	0x	0	0x	0	0x	0	0x
Caprimulgidae	*Caprimulgus saturatus*				1	29	8	28	20	26	20	40	17	38	8	30	31	44	28	55	17
Caprimulgidae	*Caprimulgus longirostris*				1	8	8	7	8	9	8	11	7	10	12	11	14	16	14	17	12
Caprimulgidae	*Caprimulgus cayennensis*				1	17	4	13	6	14	4	19	3	20	7	19	9	28	11	29	7
Caprimulgidae	*Caprimulgus candicans*	T			1	8	16	6	11	7	14	10	11	15	19	10	13	15	16	16	12

Family	Species	IUCN	Ag	R	Range Source	Percentage loss in geographic range 2050 TG H		AM H		GO H		OS H		2100 TG H		AM H		GO H		OS H	
						H	C	H	C	H	C	H	C	H	C	H	C	H	C	H	C
Caprimulgidae	*Caprimulgus maculicaudus*				1	8	10	6	10	7	10	11	9	12	12	10	14	20	14	20	12
Caprimulgidae	*Caprimulgus parvulus*				1	7	13	5	13	6	13	11	12	12	16	10	17	17	18	20	16
Caprimulgidae	*Caprimulgus anthonyi*				1	6	12	3	10	6	13	9	7	9	13	9	12	10	15	10	9
Caprimulgidae	*Caprimulgus maculosus*			x	1	0	58x	0	58x	0	58x	0	58x	0	58x	0	58x	0	62x	0	58x
Caprimulgidae	*Caprimulgus nigrescens*				1	4	13	3	13	3	14	7	12	8	15	6	16	13	17	15	15
Caprimulgidae	*Caprimulgus whitelyi*				1	2	18	2	18	2	18	2	18	2	18	2	30	2	32	8	30
Caprimulgidae	*Caprimulgus hirundinaceus*				1	6	22	5	19	9	21	13	14	9	26	8	22	17	24	22	17
Caprimulgidae	*Caprimulgus binotatus*				1	34	4	22	5	41	3	52	3	49	5	38	6	68	3	63	3
Caprimulgidae	*Caprimulgus ruficollis*				1	14	6	14	4	14	6	15	4	14	7	15	6	14	8	16	4
Caprimulgidae	*Caprimulgus indicus*		H		1	0	3	0	3	0	4	0	3	0	5	0	6	0	6	0	5
Caprimulgidae	*Caprimulgus europaeus*				1	5	13	5	12	6	14	7	11	9	16	7	15	11	18	12	14
Caprimulgidae	*Caprimulgus fraenatus*				1	45	5	26	15	52	1	52	1	49	5	31	17	54	1	53	1
Caprimulgidae	*Caprimulgus rufigena*				1	11	4	7	6	11	5	11	4	11	6	7	12	11	9	11	7
Caprimulgidae	*Caprimulgus aegyptius*				1	5	4	4	4	5	4	8	4	9	5	7	6	9	7	12	6
Caprimulgidae	*Caprimulgus mahrattensis*		H		1	0	3	0	3	0	3	0	2	0	5	0	7	0	8	0	3
Caprimulgidae	*Caprimulgus centralasicus*			x	1	0	0x	0	0x	0	0x	0	0x	0	0x	0	0x	0	0x	0	0x
Caprimulgidae	*Caprimulgus nubicus*				1	11	1	3	6	13	0	13	0	13	1	5	7	13	2	13	1
Caprimulgidae	*Caprimulgus eximius*				1	11	6	5	6	10	6	18	4	16	6	6	12	17	13	19	8
Caprimulgidae	*Caprimulgus madagascarie*				1	52	0	31	5	52	0	52	0	52	0	41	5	52	0	52	0
Caprimulgidae	*Caprimulgus macrurus*				1	14	6	16	5	17	6	19	5	18	9	20	9	23	10	27	6
Caprimulgidae	*Caprimulgus atripennis*				1	20	0	29	0	28	1	32	0	23	2	33	0	34	2	46	0
Caprimulgidae	*Caprimulgus manillensis*				1	12	12	18	11	10	13	15	13	15	18	22	15	20	20	23	15
Caprimulgidae	*Caprimulgus celebensis*				1	20	38	27	16	7	37	25	25	20	39	36	19	22	40x	31	28
Caprimulgidae	*Caprimulgus donaldsoni*				1	9	1	7	2	11	0	11	0	10	1	8	3	11	0	11	0
Caprimulgidae	*Caprimulgus pectoralis*				1	37	1	28	2	37	1	39	1	38	3	30	6	38	2	39	2
Caprimulgidae	*Caprimulgus nigriscapulari*				14	32	6	19	8	34	6	49	4	44	7	26	11	49	8	54	4
Caprimulgidae	*Caprimulgus prigoginei*	T			1	3	14	19	14	47	1x	56	13x	55	14x	19	14	71	1x	56	13x
Caprimulgidae	*Caprimulgus poliocephalus*				1	21	2	16	5	23	1	22	2	22	2	18	7	25	1	24	2
Caprimulgidae	*Caprimulgus ruwenzorii*				14	21	1	18	3	20	2	27	0	26	2	20	7	25	3	28	0
Caprimulgidae	*Caprimulgus asiaticus*		H		1	0	4	0	4	0	4	0	3	0	7	0	9	0	11	0	5
Caprimulgidae	*Caprimulgus natalensis*				1	28	4	17	7	27	4	33	3	32	4	21	10	33	5	34	4
Caprimulgidae	*Caprimulgus inornatus*				1	14	4	7	5	14	4	19	2	18	4	9	9	19	8	19	6
Caprimulgidae	*Caprimulgus stellatus*				1	56	10	18	34	68	2	68	2	61	10	21	43	70	2	70	2
Caprimulgidae	*Caprimulgus affinis*				1	14	5	17	5	15	5	18	4	17	7	20	7	20	9	25	6
Caprimulgidae	*Caprimulgus tristigma*				1	30	3	18	8	33	3	36	2	34	4	21	13	36	5	36	4
Caprimulgidae	*Caprimulgus concretus*	T			1	13	2	16	2	16	2	18	2	20	2	22	3	28	3	28	2
Caprimulgidae	*Caprimulgus pulchellus*				1	18	0x	15	0x	13	0x	18	0x	18	0x	15	0x	34	0x	18	0x
Caprimulgidae	*Caprimulgus enarratus*				1	76	0	51	7	80	0	80	0	80	0	72	7	80	0	80	0
Caprimulgidae	*Caprimulgus batesi*				1	38	6	25	9	35	9	59	4	57	7	35	13	60	11	68	5
Caprimulgidae	*Caprimulgus climacurus*				1	21	4	12	6	22	5	32	3	30	5	16	9	32	7	35	5
Caprimulgidae	*Caprimulgus clarus*				1	45	4	22	18	50	1	50	1	48	4	27	21	51	2	51	1
Caprimulgidae	*Caprimulgus fossii*				1	40	3	29	4	39	3	44	2	43	4	31	9	43	5	45	3
Caprimulgidae	*Macrodipteryx longipennis*				1	18	4	10	6	18	6	27	3	25	5	14	9	27	8	28	5
Caprimulgidae	*Macrodipteryx vexillarius*				1	45	2	32	3	44	2	48	1	47	3	34	8	47	3	49	2

Family	Species	IUCN	Ag	R	Range Source	Percentage loss in geographic range 2050 TG H	TG C	AM H	AM C	GO H	GO C	OS H	OS C	2100 TG H	TG C	AM H	AM C	GO H	GO C	OS H	OS C
Caprimulgidae	*Hydropsalis climacocerca*				1	5	12	3	12	3	13	8	12	9	14	6	14	15	15	15	13
Caprimulgidae	*Hydropsalis brasiliana*				1	6	13	5	11	6	12	10	9	10	16	8	14	15	15	18	12
Caprimulgidae	*Uropsalis segmentata*				1	15	5	15	4	16	4	25	4	22	6	27	7	30	6	35	5
Caprimulgidae	*Uropsalis lyra*				1	18	10	16	8	15	9	27	7	24	11	27	11	31	12	37	9
Caprimulgidae	*Macropsalis creagra*				1	20	6	17	7	28	4	28	6	27	15	30	15	47	8	48	12
Caprimulgidae	*Eleothreptus anomalus*				1	8	6	5	5	10	3	10	4	12	8	9	9	16	6	14	8
Columbidae	*Columba livia*				1	8	14	9	12	9	14	10	11	10	17	11	17	14	20	15	15
Columbidae	*Columba rupestris*				1	15	15	16	15	17	15	17	14	17	23	19	28	23	28	24	26
Columbidae	*Columba leuconota*				1	34	4	35	3	35	3	36	2	35	6	37	7	37	7	38	6
Columbidae	*Columba guinea*		H		1	0	3	0	6	0	3	0	2	0	4	0	10	0	6	0	5
Columbidae	*Columba albitorques*				1	20	0	15	1	22	0	22	0	22	0	17	2	22	0	22	0
Columbidae	*Columba oenas*		H		1	0	21	0	17	0	21	0	16	0	24	0	21	0	25	0	19
Columbidae	*Columba oliviae*				1	0	0	0	0	0	0	0	0	0	0	0	0	0	0	0	0
Columbidae	*Columba eversmanni*	T	H		1	0	9	0	10	0	10	0	9	0	12	0	15	0	17	0	15
Columbidae	*Columba palumbus*				1	7	19	7	16	10	20	9	15	12	24	11	21	17	25	16	18
Columbidae	*Columba trocaz*			x	1	0	0x	0	0x	0	0x	0	0x	0	0x	0	0x	0	0x	0	0x
Columbidae	*Columba bollii*			x	1	0	0x	6	0x	0	0x	0	0x	0	0x	6	0x	0	0x	0	0x
Columbidae	*Columba junoniae*	T		x	1	0	0x	0	0x	0	0x	0	0x	0	0x	0	0x	0	0x	0	0x
Columbidae	*Columba unicincta*				1	34	5	23	7	34	6	55	3	52	5	35	8	60	7	64	4
Columbidae	*Columba sjostedti*				1	16	11	12	9	33	7	27	5	23	17	18	15	43	7	32	5
Columbidae	*Columba thomensis*	T		x	1	0	⊦02x	0	⊦02x	100	0x	100	0x	0	⊦02x	0	⊦02x	100	0x	100	0x
Columbidae	*Columba arquatrix*				1	47	1	35	5	51	1	56	1	55	2	38	5	57	1	58	1
Columbidae	*Columba pollenii*			x	1	32	0x	7	0x	32	0x	32	0x	32	0x	32	0x	32	0x	32	0x
Columbidae	*Columba hodgsonii*				1	39	6	40	5	38	6	39	5	43	8	43	7	42	8	43	7
Columbidae	*Columba albinucha*				1	39	7	27	12	35	11	43	6	44	7	32	13	38	13	43	6
Columbidae	*Columba pulchricollis*				1	37	7	39	6	36	8	37	7	41	10	42	9	41	10	42	9
Columbidae	*Columba elphinstonii*	T			1	28	2	51	1	43	1	53	0	30	3	60	1	51	3	88	0
Columbidae	*Columba torringtoni*	T		x	1	48	0x	41	0x	39	0x	43	0x	48	0x	47	0x	45	0x	48	0x
Columbidae	*Columba punicea*	T			1	24	8	25	8	21	10	24	10	26	10	26	13	25	16	31	15
Columbidae	*Columba argentina*	T		x	1	0	0x	16	0x	16	0x	1	0x	0	8x	32	0x	39	0x	8	0x
Columbidae	*Columba palumboides*			x	1	14	0x	28	0x	25	0x	16	0x	14	0x	38	0x	38	0x	38	0x
Columbidae	*Columba janthina*			x	1	20	3x	7	1x	7	1x	1	3x	22	31x	23	34x	44	16x	7	45x
Columbidae	*Columba vitiensis*				1	9	4	10	4	16	4	17	3	14	7	16	8	29	8	28	5
Columbidae	*Columba leucomela*				1	10	19	17	15	20	18	23	11	13	25	20	23	25	25	35	15
Columbidae	*Columba pallidiceps*	T			1	10	2	10	1	38	0	37	1	28	4	23	1	62	1	51	1
Columbidae	*Columba leucocephala*				1	13	10	26	9	18	9	36	4	21	10	28	11	21	9	38	4
Columbidae	*Columba speciosa*				1	7	12	6	12	6	12	11	10	11	14	10	15	17	15	20	13
Columbidae	*Columba squamosa*				1	12	10	26	9	18	8	35	4	20	10	28	11	21	9	38	4
Columbidae	*Columba corensis*		H		1	0	4	0	6	0	6	0	4	0	12	0	12	0	19	0	15
Columbidae	*Columba picazuro*		H		1	0	13	0	10	0	12	0	8	0	18	0	14	0	16	0	12
Columbidae	*Columba maculosa*		H		1	0	7	0	5	0	5	0	5	0	9	0	10	0	11	0	9
Columbidae	*Columba fasciata*				1	10	14	11	13	10	14	15	12	13	17	16	21	18	23	22	19
Columbidae	*Columba araucana*				1	9	13	8	11	7	13	7	12	12	20	13	20	17	22	16	22
Columbidae	*Columba caribaea*	T		x	1	18	0x	18	6x	10	6x	44	6x	44	0x	18	6x	10	6x	44	6x

Family	Species	IUCN	Ag	R	Range Source	2050 TG H	2050 TG C	2050 AM H	2050 AM C	2050 GO H	2050 GO C	2050 OS H	2050 OS C	2100 TG H	2100 TG C	2100 AM H	2100 AM C	2100 GO H	2100 GO C	2100 OS H	2100 OS C
						Percentage loss in geographic range															
Columbidae	*Columba cayennensis*				1	8	12	6	10	7	11	12	9	11	15	11	14	17	15	20	12
Columbidae	*Columba flavirostris*				1	9	7	21	3	12	6	28	3	11	8	22	17	16	11	32	6
Columbidae	*Columba oenops*	T			1	1	2	9	2	9	2	9	2	6	2	28	2	10	2	40	2
Columbidae	*Columba inornata*	T	H		1	0	7	0	7	0	8	0	2	0	8	0	8	0	8	0	2
Columbidae	*Columba plumbea*				1	5	12	3	12	4	13	8	12	9	14	7	15	14	16	16	14
Columbidae	*Columba subvinacea*				1	5	10	4	10	4	10	8	9	9	11	7	12	14	12	16	11
Columbidae	*Columba nigrirostris*				1	18	6	29	7	19	8	36	6	23	7	34	11	30	11	43	8
Columbidae	*Columba goodsoni*				1	10	3	13	5	10	2	8	5	18	3	13	5	13	5	16	5
Columbidae	*Columba iriditorques*				1	34	6	23	8	33	7	54	4	50	7	34	9	61	8	63	4
Columbidae	*Columba malherbii*			x	1	0	87x	0	87x	87	0x	87	0x	0	87x	0	87x	87	0x	87	0x
Columbidae	*Columba delegorguei*				1	21	0	19	1	21	0	21	0	21	1	20	3	22	0	22	0
Columbidae	*Columba mayeri*	T		x	1	0	0x	0	0x	0	0x	0	0x	0	0x	0	0x	0	0x	0	0x
Columbidae	*Aplopelia larvata*				1	36	3	29	4	35	4	42	2	42	4	31	6	43	4	44	2
Columbidae	*Columba simplex*			x	14	0	102x	0	102x	100	0x	100	0x	0	102x	0	102x	100	0x	100	0x
Columbidae	*Streptopelia picturata*				1	49	0	27	5	50	0	50	0	50	0	37	5	50	0	50	0
Columbidae	*Streptopelia turtur*		H		1	0	13	0	12	0	14	0	11	0	15	0	15	0	17	0	13
Columbidae	*Streptopelia hypopyrrha*				1	33	0	26	1	35	1	50	0	52	0	32	3	61	1	52	0
Columbidae	*Streptopelia lugens*		H		1	0	2	0	4	0	1	0	1	0	2	0	5	0	3	0	3
Columbidae	*Streptopelia orientalis*				1	11	7	11	7	11	8	12	7	12	10	12	11	14	12	16	10
Columbidae	*Streptopelia senegalensis*				1	14	3	9	5	15	3	17	2	16	4	11	9	18	6	19	4
Columbidae	*Streptopelia chinensis*		H		1	0	4	0	3	0	4	0	3	0	5	0	6	0	7	0	5
Columbidae	*Streptopelia decipiens*				1	28	3	15	9	31	2	34	2	32	3	18	12	34	3	35	3
Columbidae	*Streptopelia vinacea*		H		1	0	2	0	3	0	2	0	1	0	2	0	6	0	4	0	3
Columbidae	*Streptopelia capicola*		H		1	0	3	0	9	0	3	0	3	0	5	0	13	0	5	0	5
Columbidae	*Streptopelia tranquebarica*		H		1	0	3	0	3	0	4	0	3	0	6	0	7	0	8	0	5
Columbidae	*Streptopelia semitorquata*				1	34	3	22	5	34	4	42	2	40	4	27	9	43	5	45	3
Columbidae	*Streptopelia decaocto*				1	10	11	11	9	12	11	13	8	14	14	14	14	17	16	19	11
Columbidae	*Streptopelia roseogrisea*				1	8	3	4	3	8	3	12	2	11	3	6	7	12	6	13	4
Columbidae	*Streptopelia reichenowi*			x	1	60	0x	50	4x	60	0x	60	0x	60	0x	51	7x	60	0x	60	0x
Columbidae	*Streptopelia bitorquata*		H		1	0	9	0	9	0	9	0	10	0	13	0	12	0	13	0	11
Columbidae	*Macropygia unchall*		H		1	0	4	0	3	0	4	0	4	0	5	0	5	0	6	0	5
Columbidae	*Macropygia rufipennis*		H	x	1	0	2x	0	0x	0	0x	0	0x	0	2x	0	0x	0	0x	0	0x
Columbidae	*Macropygia tenuirostris*				1	12	9	19	9	11	10	17	10	16	14	24	13	21	18	27	12
Columbidae	*Macropygia emiliana*				1	11	1	18	2	12	3	17	2	19	2	22	3	27	4	30	2
Columbidae	*Macropygia amboinensis*				1	7	5	9	4	19	4	17	3	13	7	16	8	31	8	28	6
Columbidae	*Macropygia magna*			x	1	10	27x	9	28x	11	20x	19	25x	10	27x	9	28x	13	20x	19	25x
Columbidae	*Macropygia phasianella*				1	7	22	12	14	13	20	17	9	9	28	13	25	17	28	25	12
Columbidae	*Macropygia ruficeps*				1	12	5	15	5	14	5	18	5	18	6	20	7	25	7	26	6
Columbidae	*Macropygia nigrirostris*				1	5	3	6	2	19	1	16	1	11	5	13	6	31	4	28	2
Columbidae	*Macropygia mackinlayi*				1	12	2	14	0	32	0	28	0	30	5	25	0	53	1	46	0
Columbidae	*Reinwardtoena reinwardtii*				1	5	2	7	2	17	1	15	1	12	5	13	5	28	4	26	2
Columbidae	*Reinwardtoena browni*				1	6	0	6	0	50	0	44	0	28	0	21	0	70	0	56	0
Columbidae	*Reinwardtoena crassirostri*				1	16	5	14	1	25	1	31	1	31	9	23	1	52	2	48	1
Columbidae	*Turacoena manadensis*				1	14	13	27	9	19	13	16	12	16	14	30	21	28	29	23	27

Family	Species	IUCN	Ag	R	Range Source	Percentage loss in geographic range 2050 TG H	C	AM H	C	GO H	C	OS H	C	2100 TG H	C	AM H	C	GO H	C	OS H	C
Columbidae	*Turacoena modesta*	T		x	1	4	27x	5	27x	16	16x	3	24x	4	27x	5	27x	16	17x	3	24x
Columbidae	*Turtur abyssinicus*				1	27	1	11	3	33	0	38	1	36	1	17	4	40	1	39	1
Columbidae	*Turtur chalcospilos*				1	36	2	25	7	38	1	39	1	38	3	27	11	39	2	39	2
Columbidae	*Turtur afer*				1	38	4	24	7	38	5	50	3	47	5	30	11	51	6	53	4
Columbidae	*Turtur tympanistria*				1	38	4	29	5	37	4	50	3	47	5	35	8	51	5	54	3
Columbidae	*Turtur brehmeri*				1	31	5	19	7	31	6	52	4	49	5	31	8	59	6	61	4
Columbidae	*Oena capensis*		H		1	0	4	0	8	0	3	0	3	0	5	0	11	0	7	0	6
Columbidae	*Chalcophaps indica*				1	14	9	17	7	17	9	19	6	17	11	21	11	24	13	28	9
Columbidae	*Chalcophaps stephani*				1	7	4	9	3	19	3	17	3	13	6	16	8	31	8	28	6
Columbidae	*Henicophaps albifrons*				1	3	3	4	3	16	2	13	1	7	5	12	7	25	6	22	3
Columbidae	*Henicophaps foersteri*	T			1	7	0	7	0	50	0	45	0	23	0	26	0	72	0x	58	0
Columbidae	*Phaps chalcoptera*				1	1	14	1	10	2	13	2	8	2	17	1	14	2	17	3	10
Columbidae	*Phaps elegans*				1	4	16	7	9	8	14	10	7	8	19	8	11	13	17	16	9
Columbidae	*Phaps histrionica*				1	0	11	0	11	0	11	0	10	0	11	0	12	0	13	0	11
Columbidae	*Geophaps lophotes*		H		1	0	12	0	10	0	11	0	8	0	14	0	13	0	15	0	10
Columbidae	*Geophaps plumifera*				1	0	9	0	8	0	8	0	7	0	9	0	9	0	10	0	9
Columbidae	*Geophaps smithii*			x	1	0	41x	0	13x	0	38x	0	7x	0	48x	0	25x	0	48x	0	7x
Columbidae	*Geophaps scripta*				1	0	31	0	20	1	27	1	15	0	38	0	32	1	37	2	17
Columbidae	*Petrophassa albipennis*				1	0	21	0	14	0	19	0	9	0	22	0	18	0	27	0	14
Columbidae	*Petrophassa rufipennis*				1	0	0	0	0	0	0	0	0	0	0	0	0	0	0	0	0
Columbidae	*Geopelia cuneata*				1	0	14	1	11	1	13	1	9	1	16	1	15	1	17	1	11
Columbidae	*Geopelia striata*		H		1	0	6	0	6	0	5	0	6	0	7	0	8	0	7	0	7
Columbidae	*Geopelia maugeus*		H		1	0	11	0	13	0	8	0	11	0	19	0	21	0	13	0	11
Columbidae	*Geopelia placida*		H		1	0	13	0	11	0	11	0	9	0	14	0	13	0	15	0	11
Columbidae	*Geopelia humeralis*				1	0	20	1	13	2	17	1	9	1	24	1	20	2	25	3	11
Columbidae	*Leucosarcia melanoleuca*				1	8	16	13	9	17	9	20	7	13	23	17	17	24	19	33	9
Columbidae	*Zenaida macroura*		H		1	0	18	0	12	0	16	0	10	0	21	0	19	0	23	0	15
Columbidae	*Zenaida graysoni*			x	1	0	0x	0	0x	0	0x	0	0x	0	0x	0	0x	0	0x	0	0x
Columbidae	*Zenaida auriculata*		H		1	0	11	0	10	0	11	0	9	0	15	0	14	0	16	0	13
Columbidae	*Zenaida aurita*		H		1	0	7	0	6	0	8	0	2	0	9	0	10	0	8	0	2
Columbidae	*Zenaida asiatica*		H		1	0	12	0	8	0	11	0	7	0	14	0	21	0	17	0	11
Columbidae	*Zenaida meloda*				1	6	17	7	18	8	20	12	15	8	25	13	31	18	32	22	25
Columbidae	*Zenaida galapagoensis*			x	1	0	13x	0	13x	0	8x	0	13x	0	13x	0	31x	0	41x	0	31x
Columbidae	*Columbina inca*		H		1	0	15	0	12	0	13	0	11	0	17	0	27	0	26	0	21
Columbidae	*Columbina squammata*		H		1	0	14	0	10	0	11	0	7	0	19	0	14	0	16	0	11
Columbidae	*Columbina passerina*		H		1	0	15	0	11	0	13	0	9	0	18	0	19	0	21	0	14
Columbidae	*Columbina minuta*		H		1	0	15	0	13	0	15	0	11	0	20	0	18	0	20	0	16
Columbidae	*Columbina talpacoti*		H		1	0	11	0	10	0	11	0	9	0	14	0	14	0	15	0	12
Columbidae	*Columbina buckleyi*				1	20	10	14	11	16	13	26	3	23	10	21	17	38	16	38	9
Columbidae	*Columbina picui*		H		1	0	12	0	8	0	10	0	6	0	17	0	12	0	14	0	10
Columbidae	*Columbina cruziana*		H		1	0	22	0	23	0	25	0	19	0	31	0	40	0	41	0	34
Columbidae	*Columbina cyanopis*	T			1	1	27	0	5	0	15	2	4	4	36	2	8	3	19	3	5
Columbidae	*Claravis pretiosa*				1	7	11	6	11	6	11	11	10	11	14	10	15	17	16	19	14
Columbidae	*Claravis mondetoura*				1	15	5	16	5	14	5	23	4	20	6	25	8	28	8	34	6

Family	Species	IUCN	Ag	R	Range Source	Percentage loss in geographic range 2050 TG H	C	AM H	C	GO H	C	OS H	C	2100 TG H	C	AM H	C	GO H	C	OS H	C
Columbidae	*Claravis godefrida*	T			1	10	12	9	6	13	7	15	5	15	19	14	11	24	11	24	9
Columbidae	*Metriopelia ceciliae*				1	12	18	14	20	11	19	18	16	17	25	20	29	24	28	30	22
Columbidae	*Metriopelia morenoi*		H		1	0	12	0	12	0	16	0	12	0	16	0	21	0	25	0	21
Columbidae	*Metriopelia melanoptera*				1	8	12	8	12	7	12	10	10	11	15	13	17	17	17	18	15
Columbidae	*Metriopelia aymara*		H		1	0	17	0	19	0	19	0	17	0	22	0	24	0	26	0	21
Columbidae	*Uropelia campestris*				1	5	19	2	10	2	15	8	7	8	26	6	12	7	17	15	9
Columbidae	*Leptotila verreauxi*		H		1	0	11	0	10	0	11	0	8	0	14	0	14	0	15	0	12
Columbidae	*Leptotila megalura*				1	19	13	17	9	11	9	19	9	21	14	24	12	23	14	26	10
Columbidae	*Leptotila plumbeiceps*				1	11	4	18	5	13	5	22	4	15	5	21	11	19	6	26	5
Columbidae	*Leptotila battyi*	T		x	1	18	0 x	22	0 x	4	18 x	22	0 x	18	0 x	31	0 x	4	18 x	24	0 x
Columbidae	*Leptotila wellsi*	T		x	1	0	0 x	0	0 x	0	0 x	0	0 x	0	0 x	0	0 x	0	0 x	0	0 x
Columbidae	*Leptotila rufaxilla*				1	6	12	4	11	5	12	9	11	10	14	9	15	16	15	18	14
Columbidae	*Leptotila jamaicensis*				1	3	3	17	0	14	3	26	2	8	4	21	12	16	11	32	6
Columbidae	*Leptotila pallida*				1	14	6	10	5	15	4	26	4	20	7	20	8	34	5	37	6
Columbidae	*Leptotila cassini*				1	15	4	22	6	16	6	28	4	20	5	27	9	24	10	33	6
Columbidae	*Leptotila ochraceiventris*	T			1	14	13	11	8	13	15	30	14	15	13	17	17	37	17	53	20
Columbidae	*Leptotila conoveri*	T		x	1	0	4 x	0	4 x	1	4 x	1	4 x	0	4 x	0	4 x	1	4 x	1	4 x
Columbidae	*Geotrygon lawrencii*				1	24	10	33	20	18	20	42	13	28	13	36	20	36	23	55	16
Columbidae	*Geotrygon carrikeri*	T		x	1	0	0 x	0	0 x	0	0 x	0	0 x	0	0 x	0	13 x	0	0 x	0	0 x
Columbidae	*Geotrygon costaricensis*				1	29	5	34	13	26	15	48	10	37	5	36	23	36	20	57	10
Columbidae	*Geotrygon saphirina*				1	8	1	7	1	7	1	13	1	15	1	16	2	18	1	26	1
Columbidae	*Geotrygon caniceps*	T			1	8	11	20	11	10	7	28	5	15	11	23	12	14	8	30	5
Columbidae	*Geotrygon versicolor*			x	1	18	0 x	18	6 x	10	6 x	44	6 x	44	0 x	18	6 x	10	6 x	44	6 x
Columbidae	*Geotrygon veraguensis*				1	16	5	19	7	15	6	22	5	22	7	24	8	25	10	30	7
Columbidae	*Geotrygon albifacies*		H		1	0	2	0	1	0	2	0	1	0	2	0	9	0	3	0	1
Columbidae	*Geotrygon chiriquensis*				1	22	11 x	24	19 x	28	19 x	36	19 x	30	11 x	25	33 x	49	26 x	51	19 x
Columbidae	*Geotrygon goldmani*				1	8	9	20	15 x	1	15	24	9 x	18	19 x	22	15 x	24	25 x	45	19 x
Columbidae	*Geotrygon linearis*				1	21	9	23	11	23	7	29	6	25	13	26	15	40	20	45	11
Columbidae	*Geotrygon frenata*				1	15	6	12	5	15	4	22	4	23	7	24	9	29	8	36	7
Columbidae	*Geotrygon chrysia*				1	11	10	25	8	17	9	31	3	16	11	27	10	20	9	36	3
Columbidae	*Geotrygon mystacea*			x	1	4	13 x	38	8 x	18	5 x	38	0 x	4	13 x	50	8 x	33	5 x	63	0 x
Columbidae	*Geotrygon violacea*				1	15	14	13	14	15	13	21	12	21	17	19	21	29	20	32	18
Columbidae	*Geotrygon montana*				1	7	11	6	11	7	11	12	10	11	14	11	15	17	15	20	13
Columbidae	*Starnoenas cyanocephala*	T			1	10	16 x	27	16 x	13	10 x	40	7 x	20	16 x	30	17 x	18	11 x	42	7 x
Columbidae	*Caloenas nicobarica*				1	14	7	18	7	17	7	19	7	23	10	21	10	30	10	30	8
Columbidae	*Gallicolumba luzonica*				1	7	8	22	5	15	9	16	6	7	19	33	12	26	21	26	7
Columbidae	*Gallicolumba platenae*	T		x	1	0	5 x	3	1 x	0	5 x	41	4 x	0	23 x	21	2 x	3	23 x	41	7 x
Columbidae	*Gallicolumba keayi*	T		x	1	0	75 x	25	49 x	0	75 x	0	75 x	0	75 x	25	49 x	0	75 x	0	75 x
Columbidae	*Gallicolumba criniger*	T			1	20	14	27	15	12	14	27	16	32	16	27	15	27	20	41	16
Columbidae	*Gallicolumba menagei*	T		x	1	27	0 x	0	73 x	0	73 x	0	73 x	27	0 x	27	73 x	0	73 x	0	73 x
Columbidae	*Gallicolumba rufigula*				1	3	3	5	3	17	2	13	2	10	5	11	6	26	4	24	3
Columbidae	*Gallicolumba tristigmata*				1	14	14	26	9	19	13	16	12	16	15	30	22	29	30	23	27
Columbidae	*Gallicolumba jobiensis*				1	2	2	7	1	21	1	18	1	11	4	14	5	33	4	30	2
Columbidae	*Gallicolumba stairi*	T		x	1	16	5 x	14	5 x	23	1 x	34	1 x	25	5 x	31	5 x	62	1 x	61	1 x

Family	Species	IUCN	Ag	R	Range Source	Percentage loss in geographic range 2050 TG H	TG C	AM H	AM C	GO H	GO C	OS H	OS C	2100 TG H	TG C	AM H	AM C	GO H	GO C	OS H	OS C
Columbidae	*Gallicolumba sanctaecrucis*	T		x	1	15	0x	44	0x	22	0x	8	0x	49	0x	52	0x	22	0x	37	0x
Columbidae	*Gallicolumba salamonis*	T		x	1	44	0x	24	0x	44	0x	78	0x	52	0x	33	0x	50	0x	99	0x
Columbidae	*Gallicolumba beccarii*				1	8	1	4	1	23	1	22	1	15	5	13	6	40	6	36	2
Columbidae	*Gallicolumba hoedtii*	T		x	1	10	30x	3	30x	12	21x	8	23x	10	30x	3	31x	12	22x	8	23x
Columbidae	*Trugon terrestris*				1	3	3	5	2	16	2	14	1	9	4	13	5	26	3	24	2
Columbidae	*Otidiphaps nobilis*				1	5	3	5	2	17	1	14	1	10	5	13	6	28	5	26	3
Columbidae	*Phapitreron leucotis*		H		1	0	12	0	11	0	13	0	13	0	18	0	15	0	20	0	15
Columbidae	*Phapitreron amethystina*				1	14	12	24	11	13	12	21	12	20	19	29	15	26	21	34	13
Columbidae	*Phapitreron cinereiceps*	T		x	1	15	0x	14	9x	12	9x	12	9x	15	1x	17	9x	31	9x	12	9x
Columbidae	*Treron fulvicollis*				1	13	3	16	3	16	3	18	3	20	4	22	4	29	4	28	3
Columbidae	*Treron olax*				1	13	2	15	3	15	3	17	3	20	4	22	4	28	3	27	3
Columbidae	*Treron vernans*		H		1	0	6	0	6	0	6	0	6	0	8	0	9	0	9	0	8
Columbidae	*Treron bicincta*				1	22	7	26	6	23	7	27	7	25	9	28	10	27	11	37	9
Columbidae	*Treron pompadora*				1	24	7	28	7	23	8	28	8	27	10	31	11	28	14	40	11
Columbidae	*Treron curvirostra*				1	17	6	20	6	18	7	21	6	23	8	25	9	27	10	30	8
Columbidae	*Treron griseicauda*		H		1	0	8	0	6	0	8	0	8	0	9	0	12	0	16	0	15
Columbidae	*Treron floris*	T		x	1	15	7x	14	10x	4	10x	11	6x	15	27x	14	31x	18	16x	24	6x
Columbidae	*Treron teysmannii*			x	1	0	0x	0	0x	0	0x	0	0x	0	16x	0	20x	0	16x	0	0x
Columbidae	*Treron psittacea*	T		x	1	0	39x	5	39x	18	26x	0	39x	0	39x	5	39x	18	26x	0	39x
Columbidae	*Treron capellei*	T			1	13	2	16	3	15	3	18	2	19	3	21	3	27	3	27	2
Columbidae	*Treron phoenicoptera*				1	17	5	19	5	18	5	21	5	19	8	21	8	21	10	30	6
Columbidae	*Treron waalia*				1	21	2	10	5	24	2	28	1	27	2	15	7	30	3	30	2
Columbidae	*Treron calva*				1	36	4	25	6	36	4	45	2	43	4	30	9	46	5	48	3
Columbidae	*Treron sanctithomae*			x	1	0⊦02x		0⊦02x		100	0x	100	0x	0⊦02x		0⊦02x		100	0x	100	0x
Columbidae	*Treron pembaensis*			x	1	0	0x	0	0x	0	0x	0	0x	0	0x	0	0x	0	0x	0	0x
Columbidae	*Treron australis*				1	52	0	27	7	52	0	52	0	52	0	37	7	52	0	52	0
Columbidae	*Treron apicauda*				1	26	7	26	7	22	9	29	8	29	9	28	10	26	12	38	10
Columbidae	*Treron oxyura*				1	24	4	24	4	25	3	27	5	30	6	33	6	44	4	39	5
Columbidae	*Treron seimundi*				1	5	7	11	7	18	7	41	7	10	7	13	7	39	7	48	7
Columbidae	*Treron sphenura*				1	22	8	24	7	22	8	26	8	26	11	28	11	31	12	33	11
Columbidae	*Treron sieboldii*				1	4	14	12	8	8	12	12	11	6	16	14	9	12	14	15	13
Columbidae	*Treron formosae*				1	3	1	2	1	2	1	0	1	3	1	4	1	4	1	1	1
Columbidae	*Ptilinopus porphyreus*				1	8	0	0	9	0	9	14	0	9	0	11	9	20	9	20	0
Columbidae	*Ptilinopus cinctus*				1	6	10	8	11	6	8	4	9	6	16	8	18	12	10	10	9
Columbidae	*Ptilinopus dohertyi*	T		x	1	0	0x	0	0x	0	0x	0	0x	0	16x	0	20x	0	16x	0	0x
Columbidae	*Ptilinopus alligator*				1	0	0	0	0	0	0	0	0	0	0	0	0	0	0	0	0
Columbidae	*Ptilinopus marchei*	T			1	7	7	16	6	11	7	14	4	7	16	24	14	21	16	21	8
Columbidae	*Ptilinopus merrilli*				1	8	8	21	5	13	9	15	6	8	18	33	12	24	21	27	7
Columbidae	*Ptilinopus occipitalis*				1	14	12	24	10	13	12	22	11	20	18	30	14	25	21	34	12
Columbidae	*Ptilinopus fischeri*				1	14	14	26	9	19	13	16	12	16	15	30	22	29	30	23	27
Columbidae	*Ptilinopus jambu*				1	12	2	15	3	15	3	17	2	19	3	21	3	27	3	26	2
Columbidae	*Ptilinopus leclancheri*				1	15	13	23	12	14	13	22	12	20	19	29	17	26	23	34	15
Columbidae	*Ptilinopus subgularis*				1	15	13	26	9	19	13	15	12	17	15	30	22	28	30	23	27
Columbidae	*Ptilinopus bernsteinii*				1	15	0	26	0	26	0	24	0	31	0x	27	0	42	0x	37	0x

Family	Species	IUCN	Ag	R	Range Source	Percentage loss in geographic range 2050 TG H	C	AM H	C	GO H	C	OS H	C	2100 TG H	C	AM H	C	GO H	C	OS H	C
Columbidae	*Ptilinopus magnificus*				1	5	8	8	6	17	6	16	4	10	11	14	11	25	11	28	6
Columbidae	*Ptilinopus perlatus*				1	3	4	4	4	17	2	15	2	9	6	11	7	29	5	23	3
Columbidae	*Ptilinopus ornatus*				1	6	2	6	1	12	1	11	1	10	4	14	5	23	5	23	2
Columbidae	*Ptilinopus tannensis*			x	1	5	0x	22	0x	35	0x	6	0x	19	0x	36	0x	45	0x	39	0x
Columbidae	*Ptilinopus aurantiifrons*				1	4	2	5	2	15	1	13	1	12	5	12	6	24	5	24	2
Columbidae	*Ptilinopus wallacii*				1	2	0	2	2	7	2	9	2	4	0	12	2	9	2	10	2
Columbidae	*Ptilinopus superbus*				1	6	8	8	6	17	7	15	4	11	11	14	12	27	13	25	8
Columbidae	*Ptilinopus perousii*			x	1	19	6x	18	6x	27	1x	39	1x	28	6x	36	6x	68	1x	67	1x
Columbidae	*Ptilinopus monacha*				1	18	0x	26	0x	22	0x	21	0x	35	0x	27	0x	41	0x	40	0x
Columbidae	*Ptilinopus coronulatus*				1	4	5	6	4	18	3	15	2	10	7	12	8	28	6	25	4
Columbidae	*Ptilinopus pulchellus*				1	5	3	6	2	16	2	13	1	10	5	13	7	27	5	25	3
Columbidae	*Ptilinopus regina*				1	3	20	5	12	7	18	8	8	5	26	6	20	10	26	12	9
Columbidae	*Ptilinopus greyii*				1	10	22	29	12	30	14	21	9	16	22	45	13x	37	15x	37	9
Columbidae	*Ptilinopus richardsii*			x	1	0	0x	3	0x	92	0x	92	0x	92	0x	3	0x	92	0x	94	0x
Columbidae	*Ptilinopus porphyraceus*			x	1	0	0x	0	0x	1	0x	0	0x	37	0x	0	0x	1	0x	1	0x
Columbidae	*Ptilinopus rivoli*				1	7	0	7	0	22	0	21	1	16	4	16	6	37	5	34	2
Columbidae	*Ptilinopus solomonensis*				1	10	2	9	1	37	1	35	1	28	4	21	1	59	1	49	1
Columbidae	*Ptilinopus viridis*				1	9	1	6	1	9	1	10	1	19	2	14	2	18	2	23	1
Columbidae	*Ptilinopus eugeniae*			x	1	47	0x	34	0x	39	0x	73	0x	47	0x	34	0x	39	0x	99	0x
Columbidae	*Ptilinopus hyogastra*				1	18	0	27	0x	22	0	21	0	34	0x	28	0x	40	0x	39	0x
Columbidae	*Ptilinopus granulifrons*	T		x	1	42	0x	47	0x	42	0x	38	0x	42	0x	54	0x	48	0x	38	0x
Columbidae	*Ptilinopus iozonus*				1	3	4	3	3	17	2	14	1	8	5	9	7	27	5	23	3
Columbidae	*Ptilinopus insolitus*				1	5	0	6	0	48	0	40	0	25	0	21	0	68	0	53	0
Columbidae	*Ptilinopus naina*				1	3	3	3	4	14	1	13	2	5	4	7	7	25	5	19	2
Columbidae	*Ptilinopus melanospila*				1	13	9	19	8	13	10	13	9	17	12	22	17	22	19	21	17
Columbidae	*Ptilinopus arcanus*	T		x	1	0	75x	25	49x	0	75x	0	75x	0	75x	25	49x	0	75x	0	75x
Columbidae	*Ptilinopus victor*			x	1	15	14x	0	14x	29	0x	59	0x	15	14x	0	14x	98	0x	98	0x
Columbidae	*Ptilinopus luteovirens*			x	1	21	0x	24	0x	27	0x	32	0x	21	0x	51	0x	59	0x	60	0x
Columbidae	*Ptilinopus layardi*			x	1	0	43x	0	43x	57	43x	57	43x	0	43x	0	43x	57	43x	57	43x
Columbidae	*Drepanoptila holosericea*			x	1	14	28x	45	7x	24	16x	17	17x	14	28x	55	9x	26	18x	20	17x
Columbidae	*Alectroenas madagascarie*				1	68	0	45	5	69	0	69	0	69	0	63	5	69	0	69	0
Columbidae	*Alectroenas sganzini*			x	1	19	0x	0	0x	19	0x	19	0x	19	0x	19	0x	19	0x	19	0x
Columbidae	*Ducula poliocephala*				1	15	12	24	11	13	12	21	12	20	18	30	14	25	22	34	13
Columbidae	*Ducula forsteni*				1	14	13	26	9	19	13	15	12	16	15	29	22	28	29	23	27
Columbidae	*Ducula mindorensis*	T		x	1	0	4x	3	1x	0	4x	22	3x	0	28x	27	1x	9	28x	22	12x
Columbidae	*Ducula radiata*				1	14	14	26	9	19	13	16	12	16	15	30	22	29	30	23	27
Columbidae	*Ducula carola*	T			1	12	11	22	11	10	14	17	12	17	19	29	15	23	22	30	13
Columbidae	*Ducula aenea*				1	16	5	20	5	18	6	21	5	20	7	23	8	26	10	32	7
Columbidae	*Ducula perspicillata*				1	17	0	22	0	20	0	20	0	32	0	23	0	33	0	37	0
Columbidae	*Ducula concinna*			x	1	12	7x	20	14x	33	10x	31	16x	20	7x	21	14x	44	10x	37	16x
Columbidae	*Ducula pacifica*			x	1	7	1x	12	1x	27	1x	17	1x	30	1x	26	1x	36	1x	38	1x
Columbidae	*Ducula rubricera*				1	10	2	10	0	38	0	36	0	27	4	22	0	62	1	51	0
Columbidae	*Ducula myristicivora*			x	1	5	2x	1	2x	1	2x	1	2x	5	2x	6	2x	1	2x	1	2x
Columbidae	*Ducula pistrinaria*				1	11	2	11	1	40	1	39	0	30	4	24	1	62	1	52	0

Family	Species	IUCN	Ag	R	Range Source	Percentage loss in geographic range 2050 TG H	TG C	AM H	AM C	GO H	GO C	OS H	OS C	2100 TG H	TG C	AM H	AM C	GO H	GO C	OS H	OS C
Columbidae	*Ducula rosacea*				1	10	11	14	12	8	9	12	11	11	18	14	20	18	12	21	11
Columbidae	*Ducula pickeringii*	T		x	1	29	9x	10	24x	19	23x	17	14x	29	12x	20	24x	28	24x	17	15x
Columbidae	*Ducula basilica*				1	20	0	28	0	24	0	23	0	35	0x	29	0	42	0x	39	0x
Columbidae	*Ducula rufigaster*				1	5	3	5	2	17	1	14	1	10	5	13	6	28	4	26	2
Columbidae	*Ducula finschii*				1	5	0	7	0	50	0	42	0	26	0	23	0	70	0	55	0
Columbidae	*Ducula chalconota*				1	9	1	8	1	17	1	14	1	12	5	17	6	29	5	28	3
Columbidae	*Ducula latrans*			x	1	20	7x	18	7x	28	1x	42	1x	21	7x	40	7x	79	1x	77	1x
Columbidae	*Ducula brenchleyi*	T		x	1	13	1x	12	1x	33	0x	33	1x	35	1x	12	1x	54	1x	58	1x
Columbidae	*Ducula bakeri*	T		x	1	8	0x	22	0x	26	0x	7	0x	17	0x	37	0x	35	0x	36	0x
Columbidae	*Ducula goliath*			x	1	14	30x	44	9x	25	18x	16	19x	14	30x	55	11x	26	20x	20	19x
Columbidae	*Ducula pinon*				1	3	4	6	3	18	2	15	2	10	7	12	8	28	6	27	4
Columbidae	*Ducula melanochroa*				1	5	0	7	0	50	0	43	0	26	0	22	0	70	0	55	0
Columbidae	*Ducula mullerii*				1	1	5	4	4	18	2	14	1	7	5	9	6	30	4	26	1
Columbidae	*Ducula zoeae*				1	4	3	5	2	17	2	15	1	10	5	13	6	26	4	25	3
Columbidae	*Ducula badia*				1	17	5	20	6	18	7	22	6	23	8	24	9	27	10	30	8
Columbidae	*Ducula lacernulata*			x	1	13	7x	8	10x	3	10x	9	6x	13	25x	9	28x	25	14x	21	6x
Columbidae	*Ducula cineracea*	T		x	1	10	31x	3	31x	12	22x	8	23x	10	31x	3	31x	12	22x	8	23x
Columbidae	*Ducula bicolor*				1	13	7	15	7	14	7	15	7	19	8	21	10	26	10	25	10
Columbidae	*Ducula luctuosa*				1	14	13	25	9	17	13	16	13	16	14	28	21	26	27	23	25
Columbidae	*Ducula spilorrhoa*				1	1	21	1	12	10	19	9	6	5	25	4	21	15	26	14	8
Columbidae	*Lopholaimus antarcticus*				1	7	19	12	12	15	16	17	8	10	29	13	24	20	29	26	11
Columbidae	*Hemiphaga novaeseelandi*				1	3	16	5	10	12	15	11	8	6	20	10	14	12	21	17	12
Columbidae	*Cryptophaps poecilorrhoa*				1	10	20	34	12	16	19	16	15	15	20	36	21	25	33	27	34
Columbidae	*Gymnophaps albertisii*				1	6	1	6	1	22	1	17	1	13	4	15	5	35	4	31	2
Columbidae	*Gymnophaps mada*				1	10	0x	12	0x	18	0x	17	0x	26	0x	12	0x	24	0x	32	0x
Columbidae	*Gymnophaps solomonensi*			x	1	14	6x	13	1x	25	1x	30	1x	33	12x	27	1x	57	2x	42	1x
Columbidae	*Goura cristata*	T			1	11	1	3	2	4	2	2	3	13	1	11	3	7	3	14	3
Columbidae	*Goura victoria*	T			1	2	3	4	2	27	2	11	0	10	5	10	6	41	4	31	2
Columbidae	*Goura scheepmakeri*	T			1	1	8	3	6	14	3	21	2	5	11	9	14	26	11	27	5
Columbidae	*Didunculus strigirostris*	T		x	1	0	0x	0	0x	1	0x	0	0x	46	0x	0	0x	1	0x	1	0x
Otididae	*Tetrax tetrax*		H		1	0	11	0	9	0	13	0	8	0	15	0	13	0	16	0	9
Otididae	*Otis tarda*	T	H		1	0	7	0	6	0	9	0	5	0	9	0	8	0	11	0	6
Otididae	*Neotis denhami*				1	25	3	16	5	26	4	34	2	31	4	19	8	34	5	35	4
Otididae	*Neotis ludwigii*		H		1	0	7	0	9	0	9	0	9	0	10	0	16	0	14	0	14
Otididae	*Neotis nuba*				1	0	1	0	1	0	1	1	1	1	1	0	3	1	4	1	4
Otididae	*Neotis heuglinii*				1	34	4	9	21	41	1	42	1	38	4	13	28	41	5	42	4
Otididae	*Ardeotis arabs*				1	8	3	4	3	8	3	11	2	10	3	5	6	12	6	12	5
Otididae	*Ardeotis kori*				1	25	7	10	17	28	7	28	6	26	10	12	23	29	11	29	10
Otididae	*Ardeotis nigriceps*	T			1	12	1	10	3	10	2	14	1	12	4	12	6	11	9	18	3
Otididae	*Ardeotis australis*		H		1	0	11	0	9	0	10	0	8	0	12	0	12	0	13	0	10
Otididae	*Chlamydotis undulata*	T			1	3	5	3	5	3	6	4	5	5	6	4	9	5	10	7	9
Otididae	*Eupodotis savilei*				1	6	2	3	2	6	2	9	1	8	2	5	3	9	3	10	2
Otididae	*Eupodotis gindiana*				1	46	5	17	26	53	1	53	1	50	5	21	30	54	3	54	2
Otididae	*Eupodotis ruficrista*				1	14	6	7	9	13	7	14	6	14	9	7	18	13	13	14	12

Family	Species	IUCN	Ag	R	Range Source	Percentage loss in geographic range 2050 TG H	C	AM H	C	GO H	C	OS H	C	2100 TG H	C	AM H	C	GO H	C	OS H	C
Otididae	*Eupodotis afraoides*				1	6	4	2	6	5	5	5	4	6	7	2	11	5	10	5	8
Otididae	*Eupodotis afra*				1	4	6	1	7	4	10	2	8	4	9	1	13	4	16	4	15
Otididae	*Eupodotis rueppellii*				1	6	19	3	22	6	24	5	22	6	26	3	38	6	36	5	38
Otididae	*Eupodotis vigorsii*				1	2	7	1	8	2	8	1	8	2	8	1	9	3	10	2	10
Otididae	*Eupodotis humilis*				1	21	3	6	16	25	2	25	1	23	4	8	18	25	5	25	4
Otididae	*Eupodotis senegalensis*				1	24	3	12	10	27	3	31	2	29	4	15	13	32	4	33	3
Otididae	*Eupodotis caerulescens*				1	4	0	4	0	4	0	4	0	4	1	4	2	4	1	4	1
Otididae	*Eupodotis melanogaster*				1	35	4	23	6	36	4	45	3	42	5	27	10	45	5	47	3
Otididae	*Eupodotis hartlaubii*				1	32	3	16	13	35	1	35	1	34	3	20	15	36	2	36	2
Otididae	*Eupodotis bengalensis*	T			1	28	6	30	3	32	3	33	2	29	7	34	4	33	4	36	3
Otididae	*Eupodotis indica*	T	H		1	0	7	0	7	0	10	0	6	0	13	0	21	0	29	0	10
Gruidae	*Grus virgo*		H		1	0	10	0	12	0	13	0	12	0	14	0	20	0	23	0	22
Gruidae	*Grus paradisea*	T			1	3	3	3	4	3	4	3	4	3	5	3	8	3	6	3	7
Psophiidae	*Psophia crepitans*				1	4	9	3	8	3	9	6	8	8	10	6	11	11	12	13	11
Psophiidae	*Psophia leucoptera*				1	4	9	1	10	3	10	5	10	7	11	5	15	8	14	10	13
Psophiidae	*Psophia viridis*				1	6	17	4	18	4	18	12	17	13	18	8	18	23	19	24	17
Cariamidae	*Cariama cristata*				1	6	14	4	8	6	11	10	7	9	18	8	11	11	14	15	9
Cariamidae	*Chunga burmeisteri*				1	1	8	0	7	1	7	1	6	1	11	1	13	1	15	1	14
Rhynochetidae	*Rhynochetos jubatus*	T		x	1	19	32x	41	10x	29	22x	25	16x	19	32x	57	13x	32	25x	29	16x
Rallidae	*Himantornis haematopus*				1	35	6	21	9	33	8	57	4	52	7	31	12	59	11	65	6
Rallidae	*Canirallus oculeus*				1	40	6	22	12	39	8	55	5	53	7	30	14	57	9	60	6
Rallidae	*Canirallus kioloides*				1	62	0	38	4	64	0	64	0	64	0	57	4	64	0	64	0
Rallidae	*Sarothrura pulchra*				1	39	6	25	8	38	7	56	4	52	7	33	12	57	9	61	6
Rallidae	*Sarothrura elegans*				1	39	3	28	5	39	3	47	2	45	4	33	8	48	4	50	3
Rallidae	*Sarothrura lugens*				1	48	2	43	5	51	1	52	0	49	2	46	9	54	1	53	0
Rallidae	*Sarothrura boehmi*				1	38	0	33	2	38	0	38	0	38	0	34	6	38	0	38	0
Rallidae	*Sarothrura affinis*				1	21	3	15	7	21	2	22	2	21	4	16	10	23	3	23	3
Rallidae	*Sarothrura insularis*				1	56	0	39	3	57	0	57	0	57	0	52	3	57	0	57	0
Rallidae	*Coturnicops notatus*		H		1	0	6	0	5	0	3	0	4	0	7	0	7	0	5	0	5
Rallidae	*Micropygia schomburgkii*				1	8	17	5	11	7	13	11	8	10	22	9	14	14	17	17	11
Rallidae	*Rallina rubra*				1	3	0	9	0	9	0	11	0	7	0	24	0	19	0	20	0
Rallidae	*Rallina leucospila*				1	11	0	2	2	5	2	2	2	12	0	10	2	9	2	17	2
Rallidae	*Rallina forbesi*				1	6	3	10	2	21	2	17	1	12	8	18	11	33	9	33	4
Rallidae	*Rallina mayri*				1	4	7	12	0	27	0	19	7	29	7	26	7	34	0	36	7
Rallidae	*Rallina tricolor*				1	4	7	6	4	15	7	14	3	9	10	12	10	24	10	24	5
Rallidae	*Rallina eurizonoides*				1	26	7	30	6	28	7	31	6	29	9	34	9	33	12	43	9
Rallidae	*Anurolimnas castaneiceps*				1	6	2	3	2	3	2	9	2	12	2	12	2	13	2	25	2
Rallidae	*Anurolimnas viridis*				1	5	14	4	11	5	13	9	10	9	16	8	14	14	16	17	12
Rallidae	*Anurolimnas fasciatus*				1	3	3	1	3	2	3	4	2	5	4	5	4	7	4	11	3
Rallidae	*Laterallus melanophaius*				1	6	11	4	9	6	10	10	8	10	15	9	13	14	14	17	11
Rallidae	*Laterallus spilonotus*	T		x	1	0	12x	0	12x	0	8x	0	12x	0	12x	0	31x	0	41x	0	31x
Rallidae	*Laterallus leucopyrrhus*				1	8	4	7	4	10	3	12	3	12	7	12	8	19	6	18	6
Rallidae	*Laterallus xenopterus*	T			1	15	7	5	7	10	9	11	6	24	10	15	7	26	12	21	9
Rallidae	*Nesoclopeus woodfordi*		H		1	0	3	0	2	0	0	0	2	0	9	0	2	0	2	0	2

Family	Species	IUCN	Ag	R	Range Source	Percentage loss in geographic range															
						2050								2100							
						TG		AM		GO		OS		TG		AM		GO		OS	
						H	C	H	C	H	C	H	C	H	C	H	C	H	C	H	C
Rallidae	*Nesoclopeus poecilopterus*		H	x	1	0	0x	0	0x	0	0x	0	0x	0	0x	0	0x	0	0x	0	0x
Rallidae	*Gallirallus australis*	T	H		1	0	18	0	15	0	18	0	15	0	23	0	24	0	30	0	22
Rallidae	*Gallirallus okinawae*	T		x	1	60	0x	0	0x	0	0x	0	0x	95	0x	0	0x	0	0x	0	0x
Rallidae	*Gallirallus torquatus*				1	12	10	17	8	11	10	13	10	15	13	23	14	19	17	21	15
Rallidae	*Gallirallus insignis*				1	7	0	8	0	51	0	46	0	24	0	27	0	73	0x	58	0
Rallidae	*Gallirallus rovianae*			x	1	32	16x	27	0x	25	6x	51	0x	32	16x	33	0x	40	6x	51	0x
Rallidae	*Lewinia mirificus*				1	6	6	14	5	10	6	11	4	6	14	19	14	19	13	17	8
Rallidae	*Crecopsis egregia*		H		1	0	4	0	6	0	4	0	3	0	5	0	10	0	5	0	4
Rallidae	*Crex crex*				1	4	18	4	16	5	20	4	14	7	21	6	18	10	22	10	16
Rallidae	*Aramidopsis plateni*	T			1	10	19	30	11	15	18	14	16	14	20	32	27	25	35	24	40
Rallidae	*Aramides axillaris*				1	11	8	16	8	13	9	23	7	16	9	21	15	21	14	29	10
Rallidae	*Aramides wolfi*	T			1	14	7	14	7	13	7	19	6	21	8	19	12	29	11	28	10
Rallidae	*Aramides ypecaha*				1	6	11	4	5	6	7	8	4	9	16	8	7	11	10	14	6
Rallidae	*Aramides saracura*				1	19	8	17	7	26	6	27	7	29	14	30	13	42	8	43	11
Rallidae	*Gymnocrex rosenbergii*	T	H		1	0	33	0	18	0	36	0	25	0	34	0	28	0	43	0	35
Rallidae	*Gymnocrex plumbeiventris*				1	5	3	7	3	15	2	15	1	12	5	14	6	25	4	25	3
Rallidae	*Amaurornis isabellinus*		H		1	0	13	0	11	0	13	0	14	0	14	0	20	0	21	0	20
Rallidae	*Amaurornis phoenicurus*		H		1	0	4	0	4	0	5	0	4	0	7	0	7	0	9	0	6
Rallidae	*Amaurornis olivieri*	T		x	1	18	4x	7	36x	18	4x	18	4x	18	4x	7	36x	18	4x	18	4x
Rallidae	*Porzana albicollis*		H		1	0	14	0	13	0	13	0	11	0	19	0	19	0	19	0	17
Rallidae	*Porzana paykullii*				1	12	2	12	2	12	3	12	3	12	3	12	3	12	3	12	3
Rallidae	*Aenigmatolimnas marginali*		H		1	0	2	0	3	0	1	0	1	0	3	0	6	0	2	0	2
Rallidae	*Cyanolimnas cerverai*	T		x	1	5	22x	4	22x	26	0x	95	0x	9	22x	4	22x	26	0x	95	0x
Rallidae	*Neocrex colombianus*		H		1	0	3	0	6	0	4	0	3	0	5	0	7	0	9	0	4
Rallidae	*Neocrex erythrops*		H		1	0	12	0	13	0	13	0	10	0	17	0	19	0	19	0	17
Rallidae	*Megacrex inepta*				1	1	7	4	6	22	3	19	1	9	8	9	7	35	5	29	2
Rallidae	*Porphyrio mantelli*	T		x	1	0	84x	0	84x	0	84x	0	84x	0	84x	0	84x	0	84x	0	84x
Rallidae	*Porphyrio mantelli*	T		x	1	0	84x	0	84x	0	84x	0	84x	0	84x	0	84x	0	84x	0	84x
Rallidae	*Gallinula silvestris*	T		x	1	48	0x	33	0x	40	0x	77	0x	48	0x	33	0x	40	0x	99	0x
Mesitornithidae	*Mesitornis variegata*	T			1	82	0	49	17	82	0	82	0	82	0	65	17	82	0	82	0
Mesitornithidae	*Mesitornis unicolor*	T			1	64	0	41	3	67	0	67	0	67	0	63	3	67	0	67	0
Mesitornithidae	*Monias benschi*	T			1	48	0x	8	0	48	0x	48	0x	48	0x	29	0x	48	0x	48	0x
Pteroclidae	*Syrrhaptes tibetanus*				1	17	20	19	19	19	19	19	18	19	32	23	40	28	40	28	35
Pteroclidae	*Syrrhaptes paradoxus*				1	5	8	5	9	5	10	5	9	5	11	6	16	7	18	8	17
Pteroclidae	*Pterocles alchata*				1	6	5	5	4	6	6	7	4	8	7	7	8	8	11	10	8
Pteroclidae	*Pterocles namaqua*				1	6	8	2	10	6	9	6	9	6	11	2	18	6	16	6	15
Pteroclidae	*Pterocles exustus*				1	16	5	6	10	18	4	21	3	19	6	8	14	21	8	22	5
Pteroclidae	*Pterocles senegallus*				1	2	1	2	1	2	1	3	1	3	1	2	2	3	2	3	1
Pteroclidae	*Pterocles gutturalis*				1	27	3	16	10	28	3	29	3	27	5	18	13	30	4	30	4
Pteroclidae	*Pterocles orientalis*		H		1	0	6	0	6	0	7	0	6	0	8	0	11	0	13	0	10
Pteroclidae	*Pterocles coronatus*				1	2	1	2	1	2	1	2	1	3	1	2	1	3	1	3	1
Pteroclidae	*Pterocles personatus*				1	40	0	18	6	40	0	40	0	40	0	25	6	40	0	40	0
Pteroclidae	*Pterocles decoratus*				1	44	5	13	31	51	2	51	2	48	5	17	34	52	2	52	2
Pteroclidae	*Pterocles bicinctus*				1	14	4	10	5	14	4	13	4	14	6	10	12	14	7	13	7

Family	Species	IUCN	Ag	R	Range Source	Percentage loss in geographic range 2050 TG H	TG C	AM H	AM C	GO H	GO C	OS H	OS C	2100 TG H	TG C	AM H	AM C	GO H	GO C	OS H	OS C
Pteroclidae	*Pterocles quadricinctus*				1	17	3	8	5	19	2	22	2	21	3	11	9	23	5	23	4
Pteroclidae	*Pterocles indicus*				1	13	7	13	7	10	7	16	5	14	10	14	14	11	16	21	8
Pteroclidae	*Pterocles lichtensteinii*				1	35	5	10	22	42	2	43	2	39	6	13	28	42	5	43	4
Pteroclidae	*Pterocles burchelli*				1	6	5	1	7	5	5	6	5	6	8	1	13	5	11	6	9
Thinocoridae	*Attagis gayi*				1	6	12	5	11	4	11	8	10	8	17	9	19	12	19	16	17
Thinocoridae	*Attagis malouinus*				1	1	13	1	12	2	13	1	13	1	26	2	31	4	32	3	30
Thinocoridae	*Thinocorus orbignyianus*				1	6	13	6	13	4	13	8	11	8	18	9	20	11	21	15	17
Thinocoridae	*Thinocorus rumicivorus*				1	5	14	6	16	4	16	7	13	6	22	8	26	10	25	14	20
Pedionomidae	*Pedionomus torquatus*	T	H		1	0	17	0	9	0	15	0	4	0	20	0	16	0	21	0	6
Scolopacidae	*Scolopax rusticola*				1	7	16	6	15	8	17	7	14	9	19	8	18	12	20	12	16
Scolopacidae	*Scolopax mira*	T	H	x	1	0	21x	0	0x	0	0x	0	21x	0	21x	0	8x	0	0x	0	21x
Scolopacidae	*Scolopax saturata*				1	8	1	9	1	24	0	15	1	15	5	16	6	35	3	32	3
Scolopacidae	*Scolopax celebensis*				1	18	13	25	9	21	14	22	13	18	15	32	30	35	40	23	29
Scolopacidae	*Scolopax rochussenii*	T		x	1	39	0x	44	0x	39	0x	38	0x	39	0x	54	0x	48	0x	38	0x
Scolopacidae	*Scolopax minor*		H		1	0	22	0	13	0	17	0	10	0	25	0	17	0	21	0	12
Scolopacidae	*Gallinago solitaria*				1	16	12	16	12	17	13	17	12	17	18	19	23	20	24	21	22
Scolopacidae	*Gallinago hardwickii*		H		1	0	20	0	21	0	22	0	22	0	28	0	32	0	31	0	32
Scolopacidae	*Gallinago nemoricola*	T			1	27	4	29	2	29	2	29	2	28	5	32	10	30	11	31	6
Scolopacidae	*Gallinago stenura*		H		1	0	14	0	14	0	14	0	14	0	17	0	21	0	22	0	21
Scolopacidae	*Gallinago megala*		H		1	0	7	0	8	0	8	0	8	0	9	0	10	0	10	0	10
Scolopacidae	*Gallinago media*				1	3	18	2	17	5	22	4	15	6	21	4	20	6	24	8	18
Scolopacidae	*Gallinago paraguaiae*				1	6	10	4	8	6	9	8	7	9	14	8	12	12	13	14	10
Scolopacidae	*Gallinago nobilis*				1	11	3	5	4	11	2	16	2	12	3	9	4	24	3	24	2
Scolopacidae	*Gallinago undulata*				1	9	15	7	8	9	11	13	7	13	21	12	12	19	15	21	10
Scolopacidae	*Gallinago jamesoni*				1	13	4	10	3	12	3	20	3	19	4	21	4	28	4	34	3
Scolopacidae	*Gallinago stricklandii*				1	4	10	3	11	3	12	4	10	6	17	6	22	6	23	8	22
Scolopacidae	*Gallinago imperialis*				1	3	13	6	9	10	14	14	12	5	13	10	14	15	17	21	12
Scolopacidae	*Coenocorypha pusilla*	T		x	1	0	0x	0	0x	0	0x	0	0x	0	0x	0	0x	0	0x	0	0x
Scolopacidae	*Coenocorypha aucklandica*			x	1	0⊦02x		0⊦02x		0⊦02x		0⊦02x		0⊦02x		0⊦02x		0⊦02x		0⊦02x	
Scolopacidae	*Limosa limosa*				1	5	19	4	16	6	21	5	14	8	22	7	18	10	23	10	16
Scolopacidae	*Limosa haemastica*				1	0	46	0	46	0	46	0	46	0	48	0	49	0	49	0	49
Scolopacidae	*Limosa lapponica*				1	0	25	0	25	0	25	0	25	0	33	0	40	0	42	0	39
Scolopacidae	*Limosa fedoa*				1	1	13	1	7	3	12	1	5	2	15	2	10	3	14	1	8
Scolopacidae	*Numenius minutus*				1	0	20	0	21	0	21	0	21	0	25	0	31	0	34	0	31
Scolopacidae	*Numenius borealis*	T	H		1	0	15	0	19	0	21	0	21	0	22	0	34	0	39	0	25
Scolopacidae	*Numenius phaeopus*				1	2	25	2	25	3	26	3	24	3	31	3	34	5	35	5	32
Scolopacidae	*Numenius tahitiensis*	T	H		1	0	25	0	25	0	25	0	25	0	25	0	25	0	25	0	25
Scolopacidae	*Numenius tenuirostris*	T			1	2	11	6	11	5	11	5	11	6	11	8	11	9	11	10	11
Scolopacidae	*Numenius americanus*		H		1	0	15	0	13	0	15	0	11	0	18	0	18	0	21	0	15
Scolopacidae	*Bartramia longicauda*		H		1	0	19	0	13	0	17	0	10	0	21	0	15	0	19	0	12
Scolopacidae	*Arenaria interpres*				1	0	16	0	16	0	16	0	16	0	25	1	30	1	32	1	28
Scolopacidae	*Arenaria melanocephala*				1	0	21	0	21	0	21	0	21	0	25	0	30	0	30	0	29
Scolopacidae	*Aphriza virgata*				1	0	22	0	21	0	21	0	21	0	29	0	36	0	38	1	35
Scolopacidae	*Calidris tenuirostris*				1	0	22	0	22	0	22	0	22	0	31	0	53	0	62	0	55

Family	Species	IUCN	Ag	R	Range Source	Percentage loss in geographic range 2050 TG		AM		GO		OS		2100 TG		AM		GO		OS	
						H	C	H	C	H	C	H	C	H	C	H	C	H	C	H	C
Scolopacidae	*Calidris canutus*				1	0	11	0	10	0	10	0	10	0	19	0	28	0	32	0	26
Scolopacidae	*Calidris alba*				1	0	6	0	5	0	5	0	5	0	11	0	13	0	15	0	11
Scolopacidae	*Calidris mauri*				1	0	25	0	25	0	25	0	25	0	28	0	30	0	32	0	30
Scolopacidae	*Calidris minuta*				1	0	31	0	30	0	29	0	29	0	49	0	58	0	61	0	55
Scolopacidae	*Calidris ruficollis*				1	0	22	0	22	0	22	0	22	0	36	0	44	0	48	0	42
Scolopacidae	*Calidris temminckii*				1	0	27	0	27	0	27	0	27	0	37	0	44	1	46	1	43
Scolopacidae	*Calidris fuscicollis*				1	0	2	0	2	0	2	0	2	0	5	0	7	0	8	0	5
Scolopacidae	*Calidris bairdii*		H		1	0	11	0	11	0	11	0	11	0	17	0	21	0	23	0	18
Scolopacidae	*Calidris melanotos*				1	0	21	0	21	0	20	0	21	0	29	0	36	0	38	0	33
Scolopacidae	*Calidris acuminata*		H		1	0	27	0	27	0	26	0	27	0	35	0	40	0	41	0	38
Scolopacidae	*Calidris maritima*				1	0	8	0	8	0	8	0	8	0	14	0	18	0	20	0	17
Scolopacidae	*Calidris ptilocnemis*				1	0	17	0	17	0	17	0	17	0	18	0	19	0	22	0	19
Scolopacidae	*Calidris ferruginea*				1	0	29	0	28	0	28	0	27	0	42	0	51	0	54	0	49
Scolopacidae	*Tryngites subruficollis*		H		1	0	7	0	8	0	8	0	8	0	9	0	13	0	15	0	12
Scolopacidae	*Limicola falcinellus*				1	0	30	0	30	1	29	0	30	0	36	1	39	2	39	2	38
Burhinidae	*Burhinus oedicnemus*				1	8	6	9	5	9	6	11	5	11	8	11	8	12	10	16	7
Burhinidae	*Burhinus capensis*				1	26	2	17	5	27	2	29	2	28	3	19	9	29	4	29	3
Burhinidae	*Burhinus bistriatus*				1	13	7	12	8	12	8	17	7	15	10	16	13	21	15	23	12
Burhinidae	*Burhinus superciliaris*		H		1	0	26	0	27	0	28	0	23	0	36	0	42	0	42	0	36
Burhinidae	*Burhinus grallarius*		H		1	0	20	0	13	0	17	0	9	0	23	0	20	0	24	0	11
Charadriidae	*Pluvialis apricaria*				1	3	29	3	29	4	28	4	28	4	38	5	40	8	40	9	38
Charadriidae	*Pluvialis fulva*				1	0	29	0	29	0	29	0	28	0	41	0	51	0	54	0	49
Charadriidae	*Pluvialis dominica*				1	0	14	0	14	0	13	0	14	0	21	0	25	0	27	0	23
Charadriidae	*Pluvialis squatarola*				1	0	13	0	13	0	13	0	13	0	21	0	27	0	28	0	25
Charadriidae	*Charadrius hiaticula*		H		1	0	25	0	22	0	23	0	21	0	33	0	36	0	39	0	34
Charadriidae	*Charadrius semipalmatus*				1	0	19	0	19	0	19	0	19	0	28	0	35	0	37	0	33
Charadriidae	*Charadrius vociferus*		H		1	0	18	0	14	0	16	0	12	0	21	0	21	0	24	0	17
Charadriidae	*Charadrius thoracicus*	T			1	42	1	12	9	42	1	42	1	42	1	21	9	42	1	42	1
Charadriidae	*Charadrius forbesi*				1	23	4	15	5	25	4	34	2	31	4	21	7	36	5	37	3
Charadriidae	*Charadrius leschenaultii*				1	4	9	4	10	4	11	5	11	5	11	5	16	6	18	10	17
Charadriidae	*Charadrius asiaticus*				1	1	9	1	10	1	11	1	10	2	11	1	14	2	16	6	15
Charadriidae	*Charadrius veredus*			x	1	6	1x	15	1x	6	1x	5	2x	6	1x	15	1x	6	1x	5	2x
Charadriidae	*Charadrius montanus*	T			1	1	14	1	13	1	14	1	9	1	15	1	13	1	15	2	10
Charadriidae	*Charadrius modestus*				1	2	7	2	7	2	8	3	7	4	13	5	17	4	20	6	18
Charadriidae	*Charadrius australis*		H		1	0	8	0	7	0	8	0	6	0	8	0	8	0	8	0	7
Charadriidae	*Eudromias morinellus*		H		1	0	24	0	24	0	24	0	23	0	34	0	40	0	42	0	39
Charadriidae	*Oreopholus ruficollis*				1	3	12	4	13	3	13	4	11	4	17	6	21	8	22	9	19
Charadriidae	*Vanellus vanellus*		H		1	0	15	0	14	0	17	0	14	0	18	0	18	0	20	0	17
Charadriidae	*Vanellus malarbaricus*		H		1	0	4	0	4	0	3	0	2	0	6	0	9	0	10	0	3
Charadriidae	*Vanellus tricolor*		H		1	0	11	0	8	0	10	0	6	0	13	0	10	0	13	0	7
Charadriidae	*Vanellus miles*		H		1	0	15	0	11	0	13	0	9	0	17	0	15	0	17	0	11
Charadriidae	*Vanellus tectus*				1	21	4	8	10	23	3	26	2	25	4	11	13	27	6	27	4
Charadriidae	*Vanellus melanocephalus*				1	25	1	18	1	28	1	28	1	28	1	21	2	28	2	28	2
Charadriidae	*Vanellus cinereus*				1	2	4	2	3	4	4	3	4	3	7	2	7	4	7	3	7

Family	Species	IUCN	Ag	R	Range Source	Percentage loss in geographic range 2050 TG H	2050 TG C	2050 AM H	2050 AM C	2050 GO H	2050 GO C	2050 OS H	2050 OS C	2100 TG H	2100 TG C	2100 AM H	2100 AM C	2100 GO H	2100 GO C	2100 OS H	2100 OS C
Charadriidae	*Vanellus indicus*		H		1	0	5	0	5	0	5	0	4	0	8	0	10	0	12	0	7
Charadriidae	*Vanellus senegallus*				1	19	2	12	4	20	2	23	2	22	3	14	7	23	4	23	3
Charadriidae	*Vanellus lugubris*				1	35	4	24	8	36	3	41	2	39	4	29	10	42	3	43	2
Charadriidae	*Vanellus melanopterus*				1	31	3	18	7	33	2	33	2	32	3	22	10	34	3	34	4
Charadriidae	*Vanellus coronatus*		H		1	0	5	0	11	0	4	0	4	0	6	0	17	0	8	0	7
Charadriidae	*Vanellus superciliosus*				1	22	7	13	10	22	11	34	9	36	12	19	22	40	19	38	14
Charadriidae	*Vanellus gregarius*	T			1	2	12	0	11	0	16	0	10	2	14	2	11	2	16	6	10
Charadriidae	*Vanellus cayanus*				1	9	14	7	10	8	12	12	8	13	20	11	13	18	16	19	11
Charadriidae	*Vanellus chilensis*		H		1	0	11	0	9	0	10	0	7	0	15	0	13	0	15	0	11
Charadriidae	*Vanellus resplendens*		H		1	0	10	0	10	0	11	0	9	0	14	0	15	0	15	0	11
Glareolidae	*Dromas ardeola*				1	9	11	25	1x	14	11x	30	11x	9	11	25	5x	17	13x	37	11x
Glareolidae	*Rhinoptilus africanus*				1	17	6	7	12	19	6	19	6	19	8	9	18	20	11	20	11
Glareolidae	*Rhinoptilus chalcopterus*				1	37	3	25	4	38	3	44	2	42	4	28	8	44	4	45	3
Glareolidae	*Rhinoptilus cinctus*				1	41	5	15	23	46	2	47	1	43	6	19	28	47	4	47	3
Glareolidae	*Rhinoptilus bitorquatus*	T		x	1	6	0x	19	0x	18	0x	6	0x	6	1x	19	0x	18	6x	24	0x
Glareolidae	*Cursorius cursor*				1	14	3	7	7	16	2	17	1	16	3	9	9	17	4	18	2
Glareolidae	*Cursorius rufus*				1	2	6	1	7	2	8	2	7	2	8	1	9	2	10	2	10
Glareolidae	*Cursorius temminckii*				1	26	4	16	8	28	4	31	3	29	5	19	13	31	6	31	5
Glareolidae	*Cursorius coromandelicus*		H		1	0	3	0	3	0	3	0	2	0	6	0	7	0	9	0	3
Glareolidae	*Glareola pratincola*				1	10	6	6	8	10	7	12	5	12	8	7	12	13	13	16	9
Glareolidae	*Glareola maldivarum*				1	11	4	12	5	11	5	12	5	11	7	12	9	12	11	14	8
Glareolidae	*Glareola nordmanni*				1	2	12	1	11	1	16	1	11	3	14	3	12	3	16	7	11
Glareolidae	*Glareola ocularis*	T			1	47	0	28	5	47	0	47	0	47	0	37	5	47	0	47	0
Glareolidae	*Glareola nuchalis*				1	45	5	36	5	46	3	51	2	50	5	42	8	55	4	53	3
Glareolidae	*Glareola cinerea*				1	33	8	28	7	41	6	46	5	41	9	33	10	56	7	51	5
Glareolidae	*Glareola lactea*				1	13	2	17	1	18	2	19	2	14	3	19	3	20	4	28	2
Glareolidae	*Stiltia isabella*		H		1	0	14	0	12	0	13	0	11	0	14	0	14	0	16	0	13
Laridae	*Stercorarius longicaudus*				1	0	17	0	16	0	17	0	16	0	24	0	33	1	36	1	32
Laridae	*Larus modestus*				1	0	4	0	4	0	4	0	4	0	4	0	8	0	13	1	8
Accipitridae	*Aviceda cuculoides*				1	37	4	25	6	37	5	48	3	45	5	30	9	49	6	51	4
Accipitridae	*Aviceda madagascariensis*				1	51	0	31	5	52	0	52	0	52	0	41	5	52	0	52	0
Accipitridae	*Aviceda jerdoni*				1	21	7	25	7	21	9	24	8	25	9	29	10	28	12	31	10
Accipitridae	*Aviceda subcristata*				1	3	16	4	10	9	14	9	6	6	20	6	18	15	20	15	8
Accipitridae	*Aviceda leuphotes*				1	17	4	19	3	17	5	19	4	19	5	21	4	20	6	22	5
Accipitridae	*Leptodon cayanensis*				1	8	11	6	11	7	11	12	10	12	13	11	15	18	16	20	14
Accipitridae	*Leptodon forbesi*	T		x	1	34	38x	39	37x	33	35x	43	26x	38	38x	42	39x	42	39x	45	34x
Accipitridae	*Chondrohierax uncinatus*				1	8	12	7	10	8	11	13	9	11	15	12	15	17	15	21	12
Accipitridae	*Henicopernis longicauda*				1	4	3	5	2	17	1	14	1	10	5	12	6	27	4	26	2
Accipitridae	*Henicopernis infuscatus*	T			1	7	0	7	0	51	0	45	0	23	0	27	0	72	0x	58	0
Accipitridae	*Pernis apivorus*				1	7	23	7	21	9	24	8	20	11	27	10	26	16	29	17	24
Accipitridae	*Pernis ptilorhyncus*				1	13	6	14	6	14	6	15	6	16	7	17	8	18	9	22	8
Accipitridae	*Pernis celebensis*				1	15	13	26	10	17	13	18	12	18	16	30	19	27	27	28	22
Accipitridae	*Lophoictinia isura*				1	1	15	2	11	3	13	3	9	2	18	2	16	4	19	5	11
Accipitridae	*Hamirostra melanosternon*				1	0	13	0	11	0	12	0	9	0	13	0	13	0	15	0	11

Family	Species	IUCN	Ag	R	Range Source	2050 TG H	2050 TG C	2050 AM H	2050 AM C	2050 GO H	2050 GO C	2050 OS H	2050 OS C	2100 TG H	2100 TG C	2100 AM H	2100 AM C	2100 GO H	2100 GO C	2100 OS H	2100 OS C
						Percentage loss in geographic range															
Accipitridae	*Elanoides forficatus*				1	8	12	7	10	8	11	12	9	12	16	12	15	18	16	20	12
Accipitridae	*Macheiramphus alcinus*				1	31	3	22	5	33	4	40	3	38	4	27	9	42	5	43	4
Accipitridae	*Gampsonyx swainsonii*				1	7	15	5	13	6	14	11	11	11	19	9	17	17	19	19	15
Accipitridae	*Elanus caeruleus*				1	22	4	15	6	23	4	29	3	27	5	19	9	30	7	32	5
Accipitridae	*Elanus axillaris*		H		1	0	12	0	9	0	11	0	8	0	14	0	12	0	15	0	9
Accipitridae	*Elanus leucurus*		H		1	0	14	0	11	0	13	0	9	0	18	0	17	0	18	0	14
Accipitridae	*Elanus scriptus*				1	0	7	0	7	0	7	0	7	0	8	0	9	0	8	0	8
Accipitridae	*Chelictinia riocourii*				1	7	3	3	5	8	2	9	2	9	3	4	7	10	6	9	5
Accipitridae	*Rostrhamus sociabilis*				1	9	10	9	9	10	10	14	7	13	14	13	14	20	14	23	11
Accipitridae	*Rostrhamus hamatus*				1	13	9	16	5	14	9	16	4	23	10	22	6	28	11	30	6
Accipitridae	*Harpagus bidentatus*				1	6	11	5	11	6	12	11	10	10	13	9	14	16	15	19	13
Accipitridae	*Harpagus diodon*				1	6	14	4	14	6	15	10	13	10	17	8	17	17	18	19	16
Accipitridae	*Ictinia mississippiensis*				1	5	13	8	7	7	10	8	6	10	16	12	13	17	18	12	11
Accipitridae	*Ictinia plumbea*				1	8	11	6	10	7	11	12	9	12	13	11	14	17	15	20	12
Accipitridae	*Milvus milvus*		H		1	0	23	0	17	0	22	0	15	0	28	0	24	0	28	0	19
Accipitridae	*Milvus migrans*		H		1	0	8	0	8	0	8	0	7	0	10	0	12	0	12	0	9
Accipitridae	*Haliastur sphenurus*				1	1	12	1	9	2	11	2	7	2	14	2	12	4	14	4	9
Accipitridae	*Haliastur indus*				1	12	5	14	5	15	5	17	4	16	8	18	8	22	9	26	6
Accipitridae	*Gypohierax angolensis*				1	37	4	25	6	37	5	48	3	45	5	31	9	50	6	52	4
Accipitridae	*Gypaetus barbatus*				1	9	7	7	8	9	7	10	6	11	9	10	11	12	11	14	10
Accipitridae	*Neophron percnopterus*				1	9	4	6	5	10	4	12	3	11	5	8	9	12	7	14	5
Accipitridae	*Necrosyrtes monachus*				1	28	3	17	6	29	3	34	2	32	4	19	10	34	5	35	3
Accipitridae	*Gyps africanus*				1	22	3	12	7	24	2	26	2	25	4	14	11	26	4	26	3
Accipitridae	*Gyps bengalensis*	T	H		1	0	5	0	5	0	5	0	5	0	8	0	9	0	11	0	7
Accipitridae	*Gyps indicus*	T	H		1	0	4	0	4	0	4	0	3	0	6	0	8	0	10	0	5
Accipitridae	*Gyps rueppellii*				1	18	3	8	8	21	2	23	1	22	3	11	10	25	4	24	3
Accipitridae	*Gyps himalayensis*				1	32	9	35	10	35	10	37	9	35	16	39	23	44	20	45	19
Accipitridae	*Gyps fulvus*				1	20	13	21	10	21	11	22	9	22	18	25	16	29	18	28	13
Accipitridae	*Gyps coprotheres*	T			1	4	4	4	6	4	6	4	5	4	8	4	10	4	8	4	7
Accipitridae	*Aegypius monachus*				1	25	6	26	6	26	6	28	6	28	8	29	9	30	9	32	7
Accipitridae	*Torgos tracheliotus*	T			1	14	2	8	5	15	2	16	2	15	3	9	9	16	5	16	4
Accipitridae	*Trigonoceps occipitalis*				1	26	2	15	7	28	1	30	1	29	3	17	10	31	2	30	2
Accipitridae	*Sarcogyps calvus*				1	13	4	15	4	14	4	16	4	14	6	16	7	16	9	22	5
Accipitridae	*Circaetus gallicus*				1	7	9	7	8	9	10	10	7	10	12	10	13	12	16	15	11
Accipitridae	*Circaetus beaudouini*				14	12	2	6	2	15	2	19	1	17	2	8	4	20	3	20	2
Accipitridae	*Circaetus pectoralis*				1	34	3	22	8	34	3	37	2	36	4	24	11	37	4	38	4
Accipitridae	*Circaetus cinereus*				1	23	2	14	6	25	2	27	1	25	3	16	9	27	3	27	3
Accipitridae	*Circaetus fasciolatus*				1	32	1	28	1	33	1	33	1	32	1	29	3	33	1	33	1
Accipitridae	*Circaetus cinerascens*				1	36	3	23	5	37	4	44	2	42	4	27	9	45	5	46	3
Accipitridae	*Terathopius ecaudatus*				1	22	3	12	7	24	2	26	2	25	3	15	10	26	5	27	4
Accipitridae	*Spilornis cheela*				1	15	4	18	4	17	4	19	4	19	5	21	5	23	6	28	5
Accipitridae	*Spilornis minimus*			x	1	40	0x	100	0x	100	0x	40	0x	40	0x	100	0x	100	0x	100	0x
Accipitridae	*Spilornis kinabaluensis*	T			1	14	0	20	3	18	6	18	3	24	0	24	3	31	6	35	3
Accipitridae	*Spilornis rufipectus*				1	14	13	27	9	19	13	16	12	16	14	30	20	28	29	23	26

Family	Species	IUCN	Ag	R	Range Source	Percentage loss in geographic range 2050 TG		AM		GO		OS		2100 TG		AM		GO		OS	
						H	C	H	C	H	C	H	C	H	C	H	C	H	C	H	C
Accipitridae	*Spilornis holospilus*				1	15	12	24	10	13	12	22	12	20	18	30	14	25	21	34	13
Accipitridae	*Spilornis elgini*			x	1	11	0x	11	0x	11	0x	11	0x	11	0x	20	0x	20	0x	20	0x
Accipitridae	*Dryotriorchis spectabilis*				1	35	5	24	7	34	6	54	3	51	6	33	9	59	7	63	4
Accipitridae	*Eutriorchis astur*	T			1	91	0	57	5	95	0	95	0	95	0	90	5	95	0	95	0
Accipitridae	*Circus aeruginosus*		H		1	0	15	0	13	0	17	0	12	0	18	0	16	0	20	0	15
Accipitridae	*Circus ranivorus*				1	32	3	22	8	33	2	34	2	33	4	24	13	34	3	35	3
Accipitridae	*Circus spilonotus*				1	8	6	8	6	9	6	9	6	9	7	9	7	10	7	10	7
Accipitridae	*Circus approximans*				1	1	12	2	7	3	10	3	5	4	14	3	9	5	13	5	6
Accipitridae	*Circus maillardi*	T			1	48	0	30	4	48	0	48	0	48	0	38	4	48	0	48	0
Accipitridae	*Circus buffoni*				1	8	9	6	6	8	7	10	5	11	13	10	10	15	12	17	9
Accipitridae	*Circus assimilis*				1	1	12	1	9	1	11	1	8	1	14	1	13	2	15	2	9
Accipitridae	*Circus maurus*	T	H		1	0	6	0	7	0	7	0	6	0	7	0	8	0	8	0	8
Accipitridae	*Circus cyaneus*		H		1	0	16	0	14	0	17	0	13	0	20	0	20	0	23	0	18
Accipitridae	*Circus cinereus*				1	6	8	5	8	6	8	7	7	8	12	8	14	12	15	13	13
Accipitridae	*Circus macrourus*		H		1	0	14	0	14	0	19	0	14	0	17	0	16	0	20	0	15
Accipitridae	*Circus melanoleucos*		H		1	0	4	0	4	0	5	0	5	0	5	0	5	0	6	0	5
Accipitridae	*Circus pygargus*		H		1	0	17	0	15	0	20	0	13	0	21	0	18	0	23	0	16
Accipitridae	*Polyboroides typus*				1	20	3	13	5	21	3	23	2	22	3	15	8	24	4	24	3
Accipitridae	*Polyboroides radiatus*				1	51	0	28	6	52	0	52	0	52	0	38	6	52	0	52	0
Accipitridae	*Kaupifalco monogrammicu*				1	32	3	21	5	32	4	41	2	38	4	25	9	42	5	43	3
Accipitridae	*Melierax metabates*				1	30	3	20	4	32	3	37	2	35	3	23	8	37	4	38	3
Accipitridae	*Melierax poliopterus*				1	42	3	18	18	47	1	47	1	44	4	23	22	48	2	48	2
Accipitridae	*Melierax canorus*				1	10	5	5	8	9	7	9	6	10	8	5	15	9	12	9	11
Accipitridae	*Melierax gabar*				1	20	3	11	7	22	3	23	2	23	4	13	11	24	5	24	4
Accipitridae	*Accipiter poliogaster*				1	6	15	4	15	5	16	9	15	10	18	8	19	16	20	17	18
Accipitridae	*Accipiter trivirgatus*				1	19	6	22	5	20	6	23	6	23	7	26	7	28	8	30	7
Accipitridae	*Accipiter griseiceps*				1	14	13	25	10	18	13	17	12	16	14	28	21	27	27	24	25
Accipitridae	*Accipiter tachiro*				1	43	2	31	4	43	2	47	1	46	3	34	7	46	3	48	2
Accipitridae	*Accipiter toussenelii*				14	32	6	20	8	31	8	51	5	46	7	29	11	53	9	57	6
Accipitridae	*Accipiter castanilius*				1	35	7	22	9	32	8	56	4	51	8	32	13	59	10	64	6
Accipitridae	*Accipiter badius*		H		1	0	4	0	6	0	4	0	3	0	5	0	10	0	7	0	5
Accipitridae	*Accipiter butleri*	T		x	1	0	0x	65	0x	0	0x	35	0x	0	0x	100	0x	100	0x	100	0x
Accipitridae	*Accipiter brevipes*		H		1	0	11	0	11	0	12	0	10	0	14	0	14	0	14	0	12
Accipitridae	*Accipiter soloensis*		H		1	0	1	0	0	0	1	0	1	0	1	0	0	0	1	0	1
Accipitridae	*Accipiter francesiae*				1	52	0	31	5	52	0	52	0	52	0	41	5	52	0	52	0
Accipitridae	*Accipiter trinotatus*				1	13	13	26	9	19	13	16	12	15	15	30	22	28	29	24	27
Accipitridae	*Accipiter novaehollandiae*				1	4	13	6	8	12	11	12	5	9	16	10	14	19	15	21	7
Accipitridae	*Accipiter fasciatus*		H		1	0	12	0	9	0	11	0	7	0	14	0	12	0	14	0	9
Accipitridae	*Accipiter melanochlamys*				1	4	1	7	0	24	0	16	1	13	7	16	9	37	7	35	4
Accipitridae	*Accipiter albogularis*				1	16	5	15	1	25	1	31	1	32	9	24	1	51	2	46	1
Accipitridae	*Accipiter haplochrous*			x	1	13	28x	43	7x	23	18x	16	16x	13	28x	55	9x	25	20x	19	16x
Accipitridae	*Accipiter rufitorques*			x	1	20	7x	17	7x	27	2x	40	2x	21	7x	39	7x	77	2x	75	2x
Accipitridae	*Accipiter henicogrammus*				1	19	0x	25	0x	23	0x	21	0x	37	0x	25	0x	41	0x	40	0x
Accipitridae	*Accipiter luteoschistaceus*	T			1	7	0	8	0	51	0	46	0	24	0	27	0	72	0x	58	0

Family	Species	IUCN	Ag	R	Range Source	Percentage loss in geographic range															
						2050								2100							
						TG		AM		GO		OS		TG		AM		GO		OS	
						H	C	H	C	H	C	H	C	H	C	H	C	H	C	H	C
Accipitridae	*Accipiter imitator*	T		x	1	13	3x	13	0x	15	0x	24	0x	25	12x	31	0x	52	0x	37	0x
Accipitridae	*Accipiter poliocephalus*				1	5	3	4	2	16	2	14	1	10	5	11	5	28	4	24	2
Accipitridae	*Accipiter princeps*				1	7	0	8	0	51	0	46	0	24	0	27	0	72	0x	58	0
Accipitridae	*Accipiter superciliosus*				1	7	12	5	12	6	12	11	11	11	15	9	16	17	16	20	14
Accipitridae	*Accipiter collaris*				1	12	2	11	2	12	2	21	2	20	3	23	4	28	4	38	2
Accipitridae	*Accipiter erythropus*				1	35	8	23	10	33	8	55	5	51	8	32	12	57	10	63	5
Accipitridae	*Accipiter gularis*				1	12	6	10	6	10	6	10	6	12	7	11	8	12	8	14	8
Accipitridae	*Accipiter virgatus*				1	19	3	21	3	20	3	21	3	22	4	24	5	26	6	28	5
Accipitridae	*Accipiter nanus*				1	14	16	26	10	19	15	15	13	16	16	29	24	28	33	24	31
Accipitridae	*Accipiter erythrauchen*				1	17	0	22	0	21	0	21	0	33	0	23	0	33	0	37	0
Accipitridae	*Accipiter cirrocephalus*				1	1	12	1	9	3	11	2	8	2	14	2	13	4	15	4	9
Accipitridae	*Accipiter brachyurus*	T			1	7	0	8	0	51	0	46	0	24	0	27	0	72	0x	58	0
Accipitridae	*Accipiter rhodogaster*				1	14	13	27	9	18	13	16	12	16	14	30	21	27	29	23	27
Accipitridae	*Accipiter madagascariensis*				1	40	0	18	6	40	0	40	0	40	0	27	6	40	0	40	0
Accipitridae	*Accipiter ovampensis*				1	40	2	30	3	40	1	42	1	41	3	32	6	42	2	42	2
Accipitridae	*Accipiter nisus*		H		1	0	15	0	14	0	16	0	13	0	19	0	19	0	21	0	17
Accipitridae	*Accipiter rufiventris*				1	33	2	26	4	35	2	39	1	37	2	29	7	37	2	39	1
Accipitridae	*Accipiter striatus*				1	6	22	10	18	9	20	10	17	8	27	13	26	15	28	16	23
Accipitridae	*Accipiter chionogaster*				1	13	1	23	1	19	2	27	1	15	1	26	5	23	5	29	1
Accipitridae	*Accipiter ventralis*				1	18	5	14	5	15	5	24	4	24	7	24	9	30	9	36	7
Accipitridae	*Accipiter erythronemius*				1	8	10	6	7	9	7	12	6	12	14	12	12	18	13	20	10
Accipitridae	*Accipiter cooperii*				1	8	22	14	16	12	19	14	13	12	26	18	24	21	28	21	19
Accipitridae	*Accipiter gundlachi*	T			1	8	12	25	12	12	8	37	5	17	12	28	15	16	9	40	5
Accipitridae	*Accipiter bicolor*				1	7	11	6	10	7	11	11	9	11	14	10	13	16	14	18	11
Accipitridae	*Accipiter chilensis*				1	7	13	6	11	9	12	7	12	9	22	11	22	14	23	12	23
Accipitridae	*Accipiter melanoleucus*				1	37	4	26	5	37	4	47	3	44	5	31	9	48	5	51	4
Accipitridae	*Accipiter henstii*				1	55	0	31	6	55	0	55	0	55	0	43	6	55	0	55	0
Accipitridae	*Accipiter gentilis*				1	5	17	6	15	6	17	7	15	7	21	8	21	10	23	11	20
Accipitridae	*Accipiter meyerianus*				1	5	2	6	1	29	1	21	1	15	6	12	7	44	5	39	3
Accipitridae	*Erythrotriorchis buergersi*				1	3	2	3	1	22	1	17	1	10	6	12	8	36	7	29	3
Accipitridae	*Erythrotriorchis radiatus*	T			1	0	25	1	14	2	21	2	8	1	31	1	25	3	31	4	11
Accipitridae	*Megatriorchis doriae*				1	4	3	6	2	16	2	14	1	10	4	13	5	27	3	26	2
Accipitridae	*Urotriorchis macrourus*				1	33	6	22	8	32	7	54	4	49	7	32	10	57	9	62	5
Accipitridae	*Butastur rufipennis*				1	5	2	2	3	6	2	8	1	8	3	4	5	9	5	9	4
Accipitridae	*Butastur teesa*		H		1	0	3	0	3	0	3	0	2	0	5	0	6	0	8	0	3
Accipitridae	*Butastur liventer*		H		1	0	8	0	7	0	8	0	9	0	12	0	13	0	18	0	14
Accipitridae	*Butastur indicus*				1	13	13	11	14	12	15	10	15	14	18	14	21	16	20	15	21
Accipitridae	*Geranospiza caerulescens*				1	7	11	7	10	7	10	12	8	11	14	11	13	16	14	20	11
Accipitridae	*Leucopternis plumbea*				1	13	8	18	9	14	10	17	8	19	11	23	11	27	14	28	12
Accipitridae	*Leucopternis schistacea*				1	4	8	3	8	3	8	6	8	8	9	5	9	12	10	12	9
Accipitridae	*Leucopternis princeps*				1	17	6	21	9	17	8	28	7	25	8	28	12	33	12	44	10
Accipitridae	*Leucopternis melanops*				1	4	9	2	9	3	9	5	9	7	10	4	12	10	12	10	12
Accipitridae	*Leucopternis kuhli*				1	5	8	3	7	4	8	8	7	9	8	7	8	17	9	18	8
Accipitridae	*Leucopternis lacernulata*	T			1	22	10	21	10	25	7	28	9	28	18	30	17	45	9	44	16

Family	Species	IUCN	Ag	R	Range Source	Percentage loss in geographic range 2050 TG H	TG C	AM H	AM C	GO H	GO C	OS H	OS C	2100 TG H	TG C	AM H	AM C	GO H	GO C	OS H	OS C
Accipitridae	*Leucopternis semiplumbea*				1	19	4	28	7	22	7	33	5	27	6	35	9	34	10	42	6
Accipitridae	*Leucopternis albicollis*				1	5	11	5	11	5	12	9	11	9	13	8	14	14	15	16	13
Accipitridae	*Leucopternis occidentalis*	T			1	3	7	9	5	10	8	11	8	6	7	17	6	16	9	32	9
Accipitridae	*Leucopternis polionota*				1	20	8	17	8	26	6	24	6	29	16	29	15	43	11	43	12
Accipitridae	*Buteogallus aequinoctialis*				1	9	19	10	18	11	18	15	15	12	22	14	25	25	24	25	22
Accipitridae	*Buteogallus anthracinus*				1	10	10	17	8	11	9	22	7	13	12	19	19	16	18	26	13
Accipitridae	*Buteogallus subtilis*				1	12	6	11	6	10	7	18	5	15	6	17	9	23	10	20	8
Accipitridae	*Buteogallus urubitinga*				1	8	12	7	11	8	11	13	9	11	14	11	15	17	15	20	13
Accipitridae	*Buteogallus meridionalis*				1	9	11	7	8	8	10	13	6	12	15	11	12	17	14	20	10
Accipitridae	*Parabuteo unicinctus*				1	8	12	8	10	8	11	13	9	11	15	12	16	15	16	19	13
Accipitridae	*Busarellus nigricollis*		H		1	0	13	0	10	0	11	0	7	0	17	0	14	0	15	0	11
Accipitridae	*Geranoaetus melanoleucus*				1	7	9	7	9	9	8	11	7	10	14	11	15	16	15	18	12
Accipitridae	*Harpyhaliaetus solitarius*				1	14	6	17	5	14	6	25	4	19	8	25	10	23	10	34	7
Accipitridae	*Harpyhaliaetus coronatus*	T			1	6	14	4	9	7	11	9	8	9	19	8	14	12	16	14	13
Accipitridae	*Buteo nitidus*		H		1	0	13	0	11	0	12	0	9	0	16	0	16	0	17	0	13
Accipitridae	*Buteo magnirostris*		H		1	0	11	0	10	0	10	0	8	0	14	0	13	0	14	0	11
Accipitridae	*Buteo lineatus*				1	12	22	19	11	17	17	18	7	18	26	25	16	31	22	26	9
Accipitridae	*Buteo ridgwayi*	T		x	1	9	6x	14	3x	10	7x	18	0x	9	7x	14	4x	10	7x	18	0x
Accipitridae	*Buteo platypterus*				1	13	23	20	14	19	18	20	12	20	28	28	20	34	24	30	16
Accipitridae	*Buteo leucorrhous*				1	12	7	11	7	12	6	17	6	18	10	18	11	24	12	27	10
Accipitridae	*Buteo brachyurus*				1	8	12	6	11	7	11	12	9	11	15	10	14	16	15	20	12
Accipitridae	*Buteo albigula*				1	12	5	14	5	14	4	23	4	20	7	24	8	29	8	35	6
Accipitridae	*Buteo swainsoni*		H		1	0	14	0	11	0	13	0	9	0	17	0	17	0	20	0	14
Accipitridae	*Buteo galapagoensis*	T		x	1	0	13x	0	13x	0	8x	0	13x	0	13x	0	31x	0	42x	0	31x
Accipitridae	*Buteo albicaudatus*				1	7	10	7	7	8	8	11	6	10	14	11	13	15	13	17	9
Accipitridae	*Buteo polyosoma*				1	5	11	4	11	4	12	6	10	6	15	7	19	9	20	10	17
Accipitridae	*Buteo poecilochrous*				1	11	5	12	4	11	5	22	3	19	6	24	8	25	7	38	5
Accipitridae	*Buteo albonotatus*				1	9	14	10	13	9	13	16	11	12	17	13	22	18	23	23	19
Accipitridae	*Buteo solitarius*			x	1	0	0x	22	0x	18	0x	21	0x	30	0x	78	0x	57	0x	92	0x
Accipitridae	*Buteo jamaicensis*		H		1	0	19	0	14	0	17	0	13	0	23	0	22	0	25	0	18
Accipitridae	*Buteo ventralis*				1	3	6	3	6	3	6	3	6	5	10	5	14	5	16	6	15
Accipitridae	*Buteo buteo*		H		1	0	17	0	15	0	18	0	14	0	21	0	20	0	22	0	18
Accipitridae	*Buteo oreophilus*				1	33	1	26	4	33	2	36	0	34	2	28	6	34	2	36	0
Accipitridae	*Buteo brachypterus*				1	52	0	31	5	52	0	52	0	52	0	41	5	52	0	52	0
Accipitridae	*Buteo rufinus*				1	4	8	3	8	4	10	5	8	6	10	5	11	6	13	9	11
Accipitridae	*Buteo hemilasius*				1	12	8	12	9	13	9	13	9	12	13	14	17	16	18	16	16
Accipitridae	*Buteo regalis*				1	0	12	0	10	0	12	0	8	0	14	1	11	1	14	1	9
Accipitridae	*Buteo auguralis*				1	34	6	21	9	31	8	51	4	45	7	28	13	49	10	55	6
Accipitridae	*Buteo augur*				1	34	3	23	7	36	1	36	2	35	3	26	11	37	3	36	3
Accipitridae	*Buteo archeri*				1	0	0	0	0	0	0	0	0	0	0	0	0	0	0	0	0
Accipitridae	*Buteo rufofuscus*				1	5	5	4	6	4	6	4	5	5	7	4	9	5	10	5	9
Accipitridae	*Morphnus guianensis*				1	6	12	5	12	5	12	10	11	10	14	8	15	15	16	17	14
Accipitridae	*Harpia harpyja*				1	6	11	5	11	5	11	10	10	10	13	9	14	15	15	18	13
Accipitridae	*Harpyopsis novaeguineae*	T			1	5	3	5	2	17	1	14	1	10	5	13	6	28	5	26	2

Family	Species	IUCN	Ag R	Range Source	2050 TG H	2050 TG C	2050 AM H	2050 AM C	2050 GO H	2050 GO C	2050 OS H	2050 OS C	2100 TG H	2100 TG C	2100 AM H	2100 AM C	2100 GO H	2100 GO C	2100 OS H	2100 OS C
Accipitridae	*Pithecophaga jefferyi*	T		1	14	11	23	11	12	12	21	11	20	17	29	14	25	20	33	12
Accipitridae	*Ictinaetus malayensis*			1	18	6	22	6	20	7	23	6	23	8	26	9	28	11	31	9
Accipitridae	*Aquila pomarina*			1	16	20	16	17	19	22	21	16	22	23	22	20	27	24	29	18
Accipitridae	*Aquila clanga*	T		1	6	14	6	13	8	17	7	12	9	16	8	14	11	18	12	13
Accipitridae	*Aquila rapax*			1	18	3	11	6	19	2	21	2	20	4	13	10	22	5	23	4
Accipitridae	*Aquila nipalensis*			1	2	12	2	12	2	14	2	11	2	14	2	17	4	20	6	17
Accipitridae	*Aquila adalberti*	T		1	0	23	3	12	2	19	11	8	0	28	3	25	2	25	11	10
Accipitridae	*Aquila heliaca*	T		1	6	12	4	11	5	14	6	11	8	14	7	13	9	16	11	12
Accipitridae	*Aquila gurneyi*			1	5	3	6	2	17	2	14	1	11	4	12	5	28	4	25	2
Accipitridae	*Aquila chrysaetos*			1	6	14	7	13	7	14	8	13	8	18	8	20	10	22	11	19
Accipitridae	*Aquila audax*			1	0	12	1	9	1	11	1	8	1	14	1	12	2	15	2	9
Accipitridae	*Aquila verreauxii*			1	18	3	12	5	19	3	19	3	19	4	13	9	19	5	19	5
Accipitridae	*Aquila wahlbergi*			1	21	3	15	5	22	3	24	2	23	4	17	9	25	5	25	4
Accipitridae	*Hieraaetus fasciatus*			1	9	5	10	4	11	4	13	3	11	7	12	7	13	9	18	4
Accipitridae	*Hieraaetus spilogaster*			1	31	2	20	5	33	2	36	1	34	3	23	8	36	3	37	2
Accipitridae	*Hieraaetus pennatus*			1	7	10	7	9	8	10	8	8	9	13	9	12	12	14	13	11
Accipitridae	*Hieraaetus morphnoides*			1	1	12	1	9	2	10	2	7	2	13	2	12	3	14	4	9
Accipitridae	*Hieraaetus ayresii*			1	43	4	30	6	43	4	51	3	49	5	35	10	51	5	54	3
Accipitridae	*Hieraaetus kienerii*			1	18	6	22	5	19	6	22	6	23	7	27	8	29	10	31	8
Accipitridae	*Polemaetus bellicosus*			1	21	3	12	7	23	3	25	2	24	4	14	11	26	4	26	4
Accipitridae	*Spizastur melanoleucus*			1	9	12	7	12	8	12	14	11	13	15	12	16	21	17	24	15
Accipitridae	*Lophaetus occipitalis*		H	1	0	2	0	4	0	2	0	1	0	3	0	7	0	4	0	3
Accipitridae	*Spizaetus africanus*			1	34	5	23	7	34	6	53	4	50	5	33	8	58	7	61	4
Accipitridae	*Spizaetus cirrhatus*		H	1	0	4	0	5	0	5	0	4	0	6	0	7	0	7	0	5
Accipitridae	*Spizaetus nipalensis*			1	16	5	18	4	16	5	18	5	18	7	19	8	19	9	21	8
Accipitridae	*Spizaetus alboniger*			1	16	5	20	6	19	5	25	5	22	6	27	7	34	7	33	6
Accipitridae	*Spizaetus bartelsi*	T		1	4	0	0	0	0	0	0	0	7	0	3	0	3	0	3	0
Accipitridae	*Spizaetus lanceolatus*			1	14	14	24	9	16	14	16	13	16	15	27	20	25	27	22	25
Accipitridae	*Spizaetus philippensis*	T		1	15	13	24	12	14	13	22	12	20	19	29	17	26	23	34	15
Accipitridae	*Spizaetus nanus*	T		1	13	3	16	4	15	4	18	3	19	4	21	4	27	5	27	4
Accipitridae	*Spizaetus tyrannus*			1	7	11	6	11	6	12	11	10	10	14	10	15	16	16	19	14
Accipitridae	*Spizaetus ornatus*			1	7	12	6	11	6	12	11	10	10	14	10	15	16	16	19	14
Accipitridae	*Stephanoaetus coronatus*			1	38	4	27	5	37	4	49	3	46	5	34	8	50	5	53	3
Accipitridae	*Oroaetus isidori*			1	11	3	10	2	11	2	21	2	19	3	22	4	26	3	37	3
Sagittariidae	*Sagittarius serpentarius*			1	18	3	11	6	19	3	21	3	20	4	12	10	22	6	22	5
Falconidae	*Herpetotheres cachinnans*			1	7	12	6	11	6	12	11	10	10	14	10	15	16	16	19	13
Falconidae	*Micrastur ruficollis*			1	6	11	5	11	5	12	10	10	10	13	9	15	15	15	17	13
Falconidae	*Micrastur plumbeus*	T		1	22	4	20	4	31	4	27	0	22	9	30	4	38	4	33	0
Falconidae	*Micrastur gilvicollis*			1	4	12	3	12	3	12	7	12	8	13	6	15	13	15	14	14
Falconidae	*Micrastur mirandollei*			1	4	10	3	10	4	10	7	9	8	11	6	12	14	12	15	11
Falconidae	*Micrastur semitorquatus*			1	7	11	6	10	6	11	11	9	11	13	10	14	16	15	19	12
Falconidae	*Micrastur buckleyi*			1	1	2	1	2	1	2	3	2	4	3	8	3	4	3	12	2
Falconidae	*Daptrius ater*			1	4	13	2	13	3	14	6	13	7	15	5	16	12	17	13	16
Falconidae	*Daptrius americanus*			1	6	12	5	12	5	12	10	11	10	13	9	14	15	15	18	13

Family	Species	IUCN	Ag	R	Range Source	2050 TG H	2050 TG C	2050 AM H	2050 AM C	2050 GO H	2050 GO C	2050 OS H	2050 OS C	2100 TG H	2100 TG C	2100 AM H	2100 AM C	2100 GO H	2100 GO C	2100 OS H	2100 OS C
Falconidae	*Phalcoboenus carunculatu*		H		1	0	1	0	1	0	0	0	1	0	2	0	3	0	2	0	3
Falconidae	*Phalcoboenus megalopteru*				1	6	13	6	13	4	13	8	11	7	16	8	18	11	19	14	15
Falconidae	*Phalcoboenus albogularis*				1	3	10	3	9	3	10	3	10	4	17	5	19	6	19	7	18
Falconidae	*Phalcoboenus australis*				1	0	17	0	17	0	17	0	17	0	26	0	26x	0	26x	0	26x
Falconidae	*Caracara plancus*				1	7	12	6	10	7	11	11	9	9	16	10	17	13	18	16	14
Falconidae	*Milvago chimachima*		H		1	0	13	0	10	0	11	0	7	0	17	0	14	0	15	0	11
Falconidae	*Milvago chimango*				1	4	7	3	7	4	7	5	6	6	10	6	13	8	14	9	13
Falconidae	*Spiziapteryx circumcinctus*				1	2	8	1	7	2	8	3	6	2	10	1	12	2	14	4	11
Falconidae	*Polihierax semitorquatus*				1	29	4	9	17	33	2	33	2	31	5	11	22	33	5	33	4
Falconidae	*Polihierax insignis*				1	12	8	16	9	11	10	12	10	13	10	17	15	15	16	19	16
Falconidae	*Microhierax caerulescens*		H		1	0	5	0	5	0	6	0	6	0	8	0	10	0	12	0	9
Falconidae	*Microhierax fringillarius*				1	13	3	15	4	14	3	16	4	20	4	20	4	26	4	25	4
Falconidae	*Microhierax latifrons*				1	7	0	24	1	18	2	26	1	11	0	32	1	35	2	33	1
Falconidae	*Microhierax erythrogenys*				1	3	18	5	12	6	16	8	9	6	22	7	19	9	23	12	11
Falconidae	*Microhierax melanoleucus*				1	11	2	12	2	11	3	12	3	13	4	12	3	12	4	13	4
Falconidae	*Falco berigora*		H		1	0	12	0	9	0	11	0	7	0	14	0	12	0	14	0	9
Falconidae	*Falco naumanni*	T	H		1	0	10	0	9	0	11	0	9	0	12	0	11	0	14	0	10
Falconidae	*Falco tinnunculus*		H		1	0	8	0	9	0	9	0	7	0	10	0	13	0	12	0	10
Falconidae	*Falco newtoni*				1	52	1	30	6	53	1	53	1	53	1	40	6	53	1	53	1
Falconidae	*Falco punctatus*	T		x	1	0	0x	0	0x	0	0x	0	0x	0	0x	0	0x	0	0x	0	0x
Falconidae	*Falco moluccensis*		H		1	0	6	0	6	0	5	0	7	0	8	0	11	0	9	0	8
Falconidae	*Falco cenchroides*		H		1	0	11	0	9	0	10	0	7	0	13	0	11	0	13	0	9
Falconidae	*Falco sparverius*		H		1	0	15	0	12	0	14	0	10	0	19	0	18	0	21	0	16
Falconidae	*Falco rupicoloides*				1	15	6	7	11	16	6	16	6	16	8	8	17	16	10	16	9
Falconidae	*Falco alopex*				1	14	1	6	2	17	1	20	0	18	1	9	4	21	1	21	1
Falconidae	*Falco dickinsoni*				1	28	2	22	3	28	2	28	2	28	4	23	9	28	3	28	3
Falconidae	*Falco zoniventris*				1	50	0	25	6	51	0	51	0	51	0	37	6	51	0	51	0
Falconidae	*Falco chicquera*				1	20	3	14	4	21	3	23	2	22	4	16	8	23	5	25	4
Falconidae	*Falco vespertinus*		H		1	0	15	0	14	0	17	0	13	0	17	0	16	0	19	0	15
Falconidae	*Falco amurensis*				1	10	2	10	2	10	3	10	3	10	3	10	3	10	3	10	3
Falconidae	*Falco eleonorae*				1	6	3	7	2	7	3	7	2	7	3	7	2	7	3	8	2
Falconidae	*Falco concolor*				1	0	1	0	0	1	0	1	0	1	1	1	0	1	1	1	0
Falconidae	*Falco femoralis*				1	7	11	6	10	7	11	11	9	10	14	10	16	14	16	18	13
Falconidae	*Falco columbarius*				1	2	19	3	18	3	20	3	17	3	24	4	24	5	27	6	23
Falconidae	*Falco rufigularis*				1	8	12	7	10	7	11	13	9	11	15	11	15	16	15	20	12
Falconidae	*Falco deiroleucus*				1	7	12	6	10	7	11	12	9	11	14	11	14	17	15	20	12
Falconidae	*Falco subbuteo*		H		1	0	12	0	11	0	13	0	10	0	15	0	14	0	16	0	13
Falconidae	*Falco cuvierii*				1	30	2	21	4	32	2	36	1	34	3	24	7	37	3	37	2
Falconidae	*Falco severus*		H		1	0	7	0	6	0	7	0	7	0	10	0	10	0	13	0	9
Falconidae	*Falco longipennis*				1	1	12	1	9	1	11	1	8	1	14	1	12	2	15	2	9
Falconidae	*Falco novaeseelandiae*				1	4	21	4	17	12	16	11	13	6	26	6	23	14	23	14	18
Falconidae	*Falco hypoleucos*				1	0	10	0	9	0	10	0	8	0	10	0	11	0	12	0	10
Falconidae	*Falco subniger*		H		1	0	14	0	11	0	12	0	9	0	16	0	14	0	16	0	10
Falconidae	*Falco biarmicus*				1	10	3	6	4	11	2	13	2	12	3	8	6	13	4	13	3

Family	Species	IUCN	Ag	R	Range Source	Percentage loss in geographic range 2050 TG H	C	AM H	C	GO H	C	OS H	C	2100 TG H	C	AM H	C	GO H	C	OS H	C
Falconidae	*Falco jugger*				1	13	3	13	3	12	3	16	2	14	5	15	8	14	10	22	3
Falconidae	*Falco cherrug*	T			1	8	11	8	11	9	13	9	11	11	15	10	17	13	19	14	16
Falconidae	*Falco rusticolus*				1	0	19	0	19	0	19	0	18	0	26	0	31	0	33	0	30
Falconidae	*Falco mexicanus*				1	0	16	1	14	0	16	1	13	0	19	1	21	1	25	2	20
Falconidae	*Falco peregrinus*				1	8	10	6	10	9	10	9	8	9	13	7	15	11	15	11	13
Falconidae	*Falco fasciinucha*				1	31	1	22	4	32	1	32	1	32	2	25	10	32	2	32	2
Ardeidae	*Bubulcus ibis*		H		1	0	10	0	8	0	9	0	7	0	13	0	16	0	16	0	11
Threskiornithidae	*Theristicus caudatus*				1	10	14	7	11	9	12	15	8	13	19	12	15	19	17	23	12
Threskiornithidae	*Theristicus melanopis*				1	6	9	6	9	6	10	8	9	8	14	10	17	12	18	13	16
Threskiornithidae	*Bostrychia hagedash*				1	21	3	11	9	23	3	25	2	24	4	13	12	26	4	26	3
Threskiornithidae	*Bostrychia carunculata*				1	46	0	24	6	51	0	51	0	51	0	32	10	51	0	51	0
Threskiornithidae	*Geronticus eremita*	T			1	23	0	23	0	23	0	23	0	23	0	23	0	23	0	23	0
Threskiornithidae	*Geronticus calvus*	T			1	6	0	6	0	6	0	6	0	6	2	6	0	6	0	6	0
Threskiornithidae	*Lophotibis cristata*				1	66	1	34	9	67	1	67	1	67	1	50	9	67	1	67	1
Threskiornithidae	*Threskiornis aethiopicus*		H		1	0	3	0	6	0	2	0	2	0	3	0	9	0	4	0	3
Threskiornithidae	*Threskiornis spinicollis*		H		1	0	7	0	3	0	6	0	2	0	12	0	7	0	12	0	3
Threskiornithidae	*Pseudibis davisoni*	T			1	0	0	0	0	0	0	0	0	10	0	10	0	10	0	0	0
Ciconiidae	*Coragyps atratus*				1	8	12	8	10	8	11	13	8	12	15	12	14	18	15	20	12
Ciconiidae	*Cathartes aura*				1	6	13	7	10	7	12	10	9	9	16	11	16	15	18	16	13
Ciconiidae	*Cathartes melambrotus*				1	5	9	4	9	6	8	13	7	11	9	10	11	24	11	26	9
Ciconiidae	*Gymnogyps californianus*	T		x	1	0	30x	0	30x	0	30x	0	30x	0	30x	0	30x	0	30x	0	30x
Ciconiidae	*Vultur gryphus*				1	5	11	5	12	4	12	7	11	7	16	8	20	10	21	12	18
Ciconiidae	*Sarcoramphus papa*				1	8	12	7	10	7	11	12	9	11	14	11	14	16	14	20	11
Ciconiidae	*Ciconia abdimii*				1	17	3	7	8	19	2	21	1	20	3	9	12	22	5	22	3
Ciconiidae	*Ciconia stormi*	T			1	10	0	19	0	23	0	14	0	19	0	23	2	31	1	25	0
Ciconiidae	*Ciconia maguari*				1	8	13	6	9	8	11	12	7	11	17	10	13	16	15	19	11
Ciconiidae	*Ciconia ciconia*		H		1	0	15	0	12	0	16	0	11	0	18	0	16	0	19	0	12
Ciconiidae	*Jabiru mycteria*				1	9	14	8	10	9	12	15	8	13	19	12	14	17	17	22	11
Ciconiidae	*Leptoptilos crumeniferus*				1	24	3	14	7	26	2	29	2	28	3	16	11	30	5	30	3
Acanthisittidae	*Acanthisitta chloris*		H		12,13	0	23	0	15	0	17	0	12	0	28	0	20	0	25	0	16
Acanthisittidae	*Xenicus gilviventris*			x	12,13	0	23x	0	19x	10	21x	0	19x	0	43x	0	40x	10	32x	1	39x
Pittidae	*Pitta phayrei*				7	19	11	23	10	19	11	21	11	22	13	25	15	23	17	28	15
Pittidae	*Pitta nipalensis*				7	32	10	36	8	31	12	34	11	37	12	40	11	38	14	42	12
Pittidae	*Pitta soror*				7	12	10	15	8	14	10	17	9	14	13	15	13	18	16	21	14
Pittidae	*Pitta oatesi*				7	19	14	22	12	19	15	21	14	23	17	24	19	24	21	28	20
Pittidae	*Pitta schneideri*	T			7	25	7	25	7	23	4	28	7	31	7	34	9	47	4	40	7
Pittidae	*Pitta caerulea*				7	14	4	17	4	15	4	19	4	20	4	21	5	28	5	29	5
Pittidae	*Pitta cyanea*				7	18	9	22	9	18	11	21	11	21	14	24	15	23	19	27	14
Pittidae	*Pitta guajana*				7	13	2	15	3	15	3	18	2	19	3	21	3	28	3	27	2
Pittidae	*Pitta elliotii*				7	9	15	12	11	11	14	16	12	11	20	12	22	16	28	25	21
Pittidae	*Pitta gurneyi*	T			7	2	13	2	13	2	13	7	13	2	13	2	13	8	13	9	13
Pittidae	*Pitta baudii*	T			7	8	0	13	1	12	2	14	1	16	1	18	1	21	2	23	1
Pittidae	*Pitta sordida*				7,8	18	6	22	6	21	7	23	7	24	8	26	9	30	10	31	8
Pittidae	*Pitta maxima*				7	22	0	27	0	25	0	23	0	39	0x	28	0	42	0x	39	0x

Family	Species	IUCN	Ag	R	Range Source	2050 TG H	2050 TG C	2050 AM H	2050 AM C	2050 GO H	2050 GO C	2050 OS H	2050 OS C	2100 TG H	2100 TG C	2100 AM H	2100 AM C	2100 GO H	2100 GO C	2100 OS H	2100 OS C
Pittidae	*Pitta superba*	T		x	8	31	0x	0	0x	66	0x	90	0x	90	0x	0	0x	97	0x	90	0x
Pittidae	*Pitta steerii*	T			7	20	14	27	16	13	14	27	16	31	17	27	16	27	20	41	17
Pittidae	*Pitta kochi*	T			7	7	8	21	5	13	9	16	6	7	19	34	12	24	22	27	8
Pittidae	*Pitta erythrogaster*				7,8,10	8	6	12	5	18	5	17	4	15	8	18	9	29	10	27	7
Pittidae	*Pitta dohertyi*			x	7	26	7x	28	5x	13	18x	1	18x	26	13x	30	11x	15	24x	1	24x
Pittidae	*Pitta arquata*				7	10	1	13	1	12	2	15	0	18	1	18	1	21	2	24	0
Pittidae	*Pitta granatina*				7	13	3	17	3	17	3	17	3	22	5	24	4	30	4	27	3
Pittidae	*Pitta ussheri*				14	0	0	8	0	24	0	21	0	0	0	8	0	30	0	22	0
Pittidae	*Pitta venusta*	T			7	24	7	25	7	23	5	28	7	31	7	34	8	47	5	40	7
Pittidae	*Pitta angolensis*				5	38	4	25	7	34	5	50	3	47	5	30	11	49	7	54	4
Pittidae	*Pitta reichenowi*				5	36	7	26	10	39	9	62	6	55	7	36	11	65	9	68	6
Pittidae	*Pitta brachyura*				7	19	7	18	7	17	6	25	3	20	9	18	11	17	13	34	5
Pittidae	*Pitta nympha*	T			7	6	3	7	2	6	2	6	2	6	4	7	4	7	4	7	4
Pittidae	*Pitta moluccensis*				7	11	12	14	11	11	12	15	12	12	15	15	19	16	21	22	18
Pittidae	*Pitta megarhyncha*				7	16	4	16	4	28	4	30	4	19	8	23	8	34	8	34	6
Pittidae	*Pitta elegans*				7	13	14x	10	16x	7	13x	15	12x	13	25x	10	28x	16	16x	23	12x
Pittidae	*Pitta iris*				10	0	19	1	7	0	15	2	4	0	19	1	11	0	19	2	4
Pittidae	*Pitta versicolor*				8,10	8	21	13	17	17	19	18	11	10	26	16	25	22	26	30	15
Pittidae	*Pitta anerythra*	T		x	9	13	3x	12	0x	16	0x	23	0x	25	12x	31	0x	53	0x	36	0x
Eurylaimidae	*Smithornis capensis*				5	42	2	35	3	43	2	48	2	45	3	39	6	47	2	49	2
Eurylaimidae	*Smithornis sharpei*				5	28	5	19	6	34	6	51	2	43	6	28	9	50	7	57	2
Eurylaimidae	*Smithornis rufolateralis*				5	34	6	21	8	34	6	53	5	51	7	31	9	58	7	62	5
Eurylaimidae	*Pseudocalyptomena grauer*	T		x	5	6	2x	4	1x	21	1x	7	0x	6	2x	7	1x	21	1x	7	0x
Eurylaimidae	*Corydon sumatranus*				7	14	6	17	6	15	6	18	6	19	7	21	8	25	10	27	8
Eurylaimidae	*Cymbirhynchus macrorhyn*		H		7	0	5	0	5	0	5	0	5	0	7	0	8	0	9	0	7
Eurylaimidae	*Eurylaimus javanicus*				7	12	5	15	5	14	5	17	5	17	7	20	8	24	9	26	7
Eurylaimidae	*Eurylaimus ochromalus*				7	13	3	15	4	15	4	18	3	19	4	21	5	27	5	27	4
Eurylaimidae	*Eurylaimus steerii*	T			7	17	15	27	17	8	17	19	17	27	18	27	17	25	22	35	17
Eurylaimidae	*Eurylaimus samarensis*	T		x	14	25	10x	23	10x	28	3x	54	10x	44	12x	23	10x	35	12x	61	12x
Eurylaimidae	*Serilophus lunatus*				7	18	8	22	7	19	9	22	8	22	11	25	10	27	13	29	10
Eurylaimidae	*Psarisomus dalhousiae*				7	20	7	24	7	20	8	24	8	24	11	27	11	28	13	31	11
Eurylaimidae	*Calyptomena viridis*				7	13	3	16	4	15	4	18	3	19	4	21	5	27	5	27	4
Eurylaimidae	*Calyptomena hosii*				7	10	0	12	1	9	2	13	1	15	0	15	1	19	2	22	1
Eurylaimidae	*Calyptomena whiteheadi*				7	10	0	13	1	9	2	13	1	15	0	15	1	20	2	22	1
Philepittidae	*Philepitta castanea*				6	76	0	47	6	79	0	79	0	79	0	70	6	79	0	79	0
Philepittidae	*Philepitta schlegeli*			x	6	57	0x	46	2x	57	0x	57	0x	57	0x	46	2x	57	0x	57	0x
Philepittidae	*Neodrepanis coruscans*				6	75	0	45	4	78	0	78	0	78	0	71	4	78	0	78	0
Philepittidae	*Neodrepanis hypoxantha*	T		x	6	93	0x	93	0x	93	0x	93	0x	93	0x	93	0x	93	0x	93	0x
Philepittidae	*Sapayoa aenigma*				2	10	5	16	7	12	6	13	5	20	9	18	7	20	11	28	8
Tyrannidae	*Mionectes striaticollis*				2	14	6	13	4	13	6	21	6	19	6	21	9	27	9	27	8
Tyrannidae	*Mionectes olivaceus*				2	18	5	18	6	19	6	30	4	27	7	28	9	34	10	42	6
Tyrannidae	*Mionectes oleagineus*				2	7	11	6	11	6	12	11	10	11	13	10	15	17	15	19	13
Tyrannidae	*Mionectes macconnelli*				2	7	12	5	12	6	12	11	11	12	13	10	14	20	15	21	14
Tyrannidae	*Mionectes rufiventris*				2	22	8	20	8	30	6	31	7	33	15	34	14	49	9	49	13

Family	Species	IUCN	Ag	R	Range Source	2050 TG H	2050 TG C	2050 AM H	2050 AM C	2050 GO H	2050 GO C	2050 OS H	2050 OS C	2100 TG H	2100 TG C	2100 AM H	2100 AM C	2100 GO H	2100 GO C	2100 OS H	2100 OS C
Tyrannidae	*Leptopogon rufipectus*				2	18	2	24	3	10	4	24	4	24	2	31	4	27	11	30	8
Tyrannidae	*Leptopogon taczanowskii*				2	3	3	3	1	4	3	5	5	4	3	3	8	10	9	8	5
Tyrannidae	*Leptopogon amaurocephal*				2	8	14	7	14	8	14	13	13	13	17	13	18	18	18	22	17
Tyrannidae	*Leptopogon superciliaris*				2	17	6	17	6	15	7	29	5	24	7	23	11	31	10	38	7
Tyrannidae	*Pseudotriccus pelzelni*				2	18	8	17	7	17	9	30	7	24	10	29	14	35	10	35	10
Tyrannidae	*Pseudotriccus simplex*				2	23	5	3	5	13	5	36	0	25	5	18	7	28	7	40	2
Tyrannidae	*Pseudotriccus ruficeps*				2	12	5	15	3	13	5	19	7	15	7	22	7	26	8	21	9
Tyrannidae	*Poecilotriccus ruficeps*				2	16	9	25	7	13	12	27	11	26	13	36	16	38	17	33	17
Tyrannidae	*Poecilotriccus luluae*	T		x	14	0	0x	0	0x	0	0x	0	0x	0	0x	0	0x	0	0x	0	0x
Tyrannidae	*Poecilotriccus capitalis*				2	6	2	6	2	6	1	12	2	12	3	20	2	18	1	29	2
Tyrannidae	*Poecilotriccus albifacies*				2	6	7	0	7	0	7	19	0	6	7	0	7	6	7	23	0
Tyrannidae	*Taeniotriccus andrei*				2	9	9	7	10	9	9	17	9	18	11	13	13	35	14	35	13
Tyrannidae	*Hemitriccus minor*				2	5	15	3	16	3	16	8	15	10	16	7	17	17	17	17	17
Tyrannidae	*Hemitriccus spodiops*				2	12	0	6	0	3	0	13	0	12	0	14	0	16	0	13	0
Tyrannidae	*Hemitriccus josephinae*				2	2	15	2	14	3	14	7	14	5	15	3	15	13	17	11	15
Tyrannidae	*Hemitriccus flammulatus*				2	11	15	6	17	8	16	12	16	16	17	14	22	18	20	19	21
Tyrannidae	*Hemitriccus diops*				2	16	9	16	9	23	5	26	7	23	18	24	18	41	11	41	13
Tyrannidae	*Hemitriccus obsoletus*				2	22	3	19	3	30	2	31	2	31	5	38	5	53	3	63	4
Tyrannidae	*Hemitriccus zosterops*				2	3	5	2	4	2	5	5	4	5	5	5	5	9	6	12	5
Tyrannidae	*Hemitriccus griseipectus*				14	7	9	5	8	5	9	11	8	12	10	10	10	19	10	20	9
Tyrannidae	*Hemitriccus minimus*			x	14	3	18x	0	18x	0	18x	0	18x	3	18x	13	18x	23	18x	13	18x
Tyrannidae	*Hemitriccus orbitatus*				2	24	10	17	13	27	9	23	12	28	24	26	24	41	14	35	22
Tyrannidae	*Hemitriccus iohannis*				2	2	8	1	8	2	8	5	7	5	9	5	10	5	11	11	9
Tyrannidae	*Hemitriccus striaticollis*				2	7	20	4	20	4	21	10	19	11	22	8	23	18	24	19	21
Tyrannidae	*Hemitriccus nidipendulus*				2	20	13	14	15	23	9	21	12	23	24	21	28	41	16	30	24
Tyrannidae	*Hemitriccus margaritaceive*				2	8	16	5	12	6	14	12	9	11	20	9	16	14	19	19	14
Tyrannidae	*Hemitriccus inornatus*			x	14	0	0x	0	0x	0	0x	0	0x	0	0x	0	0x	0	0x	0	0x
Tyrannidae	*Hemitriccus granadensis*				2	12	6	13	5	7	7	15	9	18	6	17	10	23	12	21	13
Tyrannidae	*Hemitriccus rufigularis*				2	37	7	10	4	15	6	40	6	40	7	25	10	39	7	44	7
Tyrannidae	*Hemitriccus cinnamomeipe*			x	2	0	21x	4	17x	0	21x	0	21x	0	21x	4	17x	0	21x	0	21x
Tyrannidae	*Hemitriccus mirandae*	T			14	9	14	15	10	12	14	9	14	10	20	21	11	24	16	16	23
Tyrannidae	*Hemitriccus kaempferi*	T		x	2	38	0x	9	16x	93	0x	76	0x	38	60x	60	38x	93	5x	81	17x
Tyrannidae	*Todirostrum senex*			x	14	0	0x	0	0x	0	0x	0	0x	0	0x	0	0x	0	6x	0	6x
Tyrannidae	*Todirostrum russatum*			x	2	13	1x	0	14x	19	3x	0	14x	13	20x	14	30x	30	15x	9	45x
Tyrannidae	*Todirostrum plumbeiceps*				2	19	8	16	5	23	5	28	5	26	11	27	10	40	8	41	8
Tyrannidae	*Todirostrum latirostre*				2	6	19	3	15	4	18	7	14	10	24	7	18	11	21	14	16
Tyrannidae	*Todirostrum fumifrons*				2	9	12	5	12	6	12	14	11	15	13	10	14	28	15	29	12
Tyrannidae	*Todirostrum sylvia*				2	13	7	11	8	12	7	20	6	17	9	16	13	23	12	30	9
Tyrannidae	*Todirostrum maculatum*				2	5	9	4	9	4	9	9	8	10	10	8	11	16	11	18	9
Tyrannidae	*Todirostrum poliocephalum*				2	15	7	9	8	17	5	19	7	19	13	15	15	29	8	25	11
Tyrannidae	*Todirostrum cinereum*				2	10	13	8	11	9	12	15	9	14	17	12	15	21	16	25	12
Tyrannidae	*Todirostrum viridanum*				2	17	17x	23	18x	10	22x	23	17x	17	46x	27	31x	10	49x	23	45x
Tyrannidae	*Todirostrum nigriceps*				2	17	4	18	7	15	6	22	4	24	6	21	10	26	12	33	5
Tyrannidae	*Todirostrum pictum*				2	3	13	3	13	3	13	5	13	6	15	5	17	11	18	10	17

Family	Species	IUCN	Ag	R	Range Source	2050 TG H	2050 TG C	2050 AM H	2050 AM C	2050 GO H	2050 GO C	2050 OS H	2050 OS C	2100 TG H	2100 TG C	2100 AM H	2100 AM C	2100 GO H	2100 GO C	2100 OS H	2100 OS C
Tyrannidae	*Todirostrum chrysocrotaph*				2	6	9	4	9	5	9	9	8	10	10	9	10	15	10	18	10
Tyrannidae	*Todirostrum calopterum*				2	10	2	3	2	4	1	14	2	15	2	15	3	19	1	27	2
Tyrannidae	*Todirostrum pulchellum*			x	2	13	13x	0	13x	0	13x	39	0x	13	13x	0	13x	13	13x	42	0x
Tyrannidae	*Corythopis torquata*				2	5	10	4	10	4	10	8	10	9	11	7	12	14	13	16	12
Tyrannidae	*Corythopis delalandi*				2	12	17	9	15	13	16	21	12	18	23	18	22	31	20	36	18
Tyrannidae	*Phyllomyias fasciatus*				2	11	14	8	12	14	13	18	8	15	20	15	16	24	16	30	12
Tyrannidae	*Phyllomyias zeledoni*				2	29	15	17	18	24	21	31	17	29	15	28	18	41	21	37	17
Tyrannidae	*Phyllomyias burmeisteri*				2	21	8	17	7	25	5	29	6	28	15	31	15	44	9	47	12
Tyrannidae	*Phyllomyias reiseri*				2	7	20	4	6	4	11	10	4	12	26	8	6	8	13	15	4
Tyrannidae	*Phyllomyias urichi*	T		x	2	0	0x	0	0x	0	0x	0	0x	0	0x	7	0x	7	0x	7	0x
Tyrannidae	*Phyllomyias virescens*				2	22	9	19	8	30	6	32	7	31	16	34	15	49	10	50	13
Tyrannidae	*Phyllomyias sclateri*				2	6	2	7	0	3	0	8	2	7	2	9	3	11	2	9	4
Tyrannidae	*Phyllomyias griseocapilla*				2	27	14	15	23	23	16	20	21	29	32	24	36	47	21	32	35
Tyrannidae	*Phyllomyias griseiceps*				2	15	6	12	7	17	6	24	3	23	8	23	11	32	11	38	7
Tyrannidae	*Phyllomyias plumbeiceps*				2	5	19	3	14	2	20	8	18	5	19	8	18	12	25	13	18
Tyrannidae	*Phyllomyias nigrocapillus*				2	14	4	14	4	13	6	21	6	19	8	21	12	28	13	26	8
Tyrannidae	*Phyllomyias cinereiceps*				2	13	6	13	3	11	5	19	8	19	7	21	12	29	8	22	11
Tyrannidae	*Phyllomyias uropygialis*				2	13	6	16	2	13	5	16	8	18	10	24	9	27	9	21	12
Tyrannidae	*Zimmerius vilissimus*				2	19	5	31	8	22	9	38	6	26	6	36	11	34	12	45	8
Tyrannidae	*Zimmerius improbus*				2	21	0x	11	2	24	2x	26	2x	31	2x	24	9x	35	9x	31	3x
Tyrannidae	*Zimmerius bolivianus*				2	16	5	7	1	13	2	20	4	20	5	18	7	25	6	24	8
Tyrannidae	*Zimmerius cinereicapillus*				2	12	7x	10	0	9	7x	26	9x	26	7x	18	20x	37	7x	37	9x
Tyrannidae	*Zimmerius gracilipes*				2	5	10	3	10	4	10	7	10	8	11	6	12	13	13	14	12
Tyrannidae	*Zimmerius viridiflavus*			x	2	22	0x	26	0x	11	0x	11	11x	37	0x	26	0x	37	0x	26	11x
Tyrannidae	*Zimmerius chrysops*				2	12	3	10	4	9	4	21	2	16	4	19	7	24	7	33	4
Tyrannidae	*Zimmerius flavidifrons*			x	14	6	16x	9	1x	0	17x	0	10x	6	16x	9	3x	0	19x	0	12x
Tyrannidae	*Ornithion inerme*				2	5	10	3	10	4	11	8	10	9	12	7	12	14	13	15	12
Tyrannidae	*Ornithion semiflavum*				2	17	5	27	5	19	5	35	3	22	6	34	10	29	8	42	5
Tyrannidae	*Ornithion brunneicapillum*				2	20	4	18	7	19	6	24	4	26	6	22	7	27	9	32	5
Tyrannidae	*Camptostoma imberbe*				2	11	8	21	4	13	6	28	3	14	9	22	18	16	13	31	6
Tyrannidae	*Camptostoma obsoletum*				2	8	11	6	11	7	11	11	9	11	14	10	14	17	15	20	13
Tyrannidae	*Phaeomyias murina*				2	7	14	5	12	6	13	11	11	11	17	9	15	16	17	19	13
Tyrannidae	*Phaeomyias tumbezana*				14	4	9	1	10	0	13	2	10	4	13	2	18	4	20	3	18
Tyrannidae	*Capsiempis flaveola*				2	9	10	7	8	10	8	14	6	13	14	12	12	20	13	22	10
Tyrannidae	*Sublegatus arenarum*				2	17	6	12	8	16	5	20	4	21	8	18	10	30	11	31	7
Tyrannidae	*Sublegatus obscurior*				2	5	9	3	8	4	8	7	8	8	9	7	11	12	11	14	10
Tyrannidae	*Sublegatus modestus*				2	7	17	4	14	6	15	9	12	9	21	7	18	12	20	14	16
Tyrannidae	*Suiriri affinis*				2	7	19	5	12	6	16	11	9	9	24	9	14	12	18	18	11
Tyrannidae	*Suiriri suiriri*				2	3	6	2	5	3	5	4	4	3	8	3	10	5	12	6	10
Tyrannidae	*Suiriri islerorum*				14	5	24	1	15	1	22	8	15	7	33	4	19	7	25	12	17
Tyrannidae	*Tyrannulus elatus*				2	6	9	4	9	5	9	9	8	10	10	9	11	15	12	17	10
Tyrannidae	*Myiopagis gaimardii*				2	6	12	4	12	4	12	9	11	10	13	8	14	15	15	17	13
Tyrannidae	*Myiopagis olallai*			x	14	33	10x	5	44x	0	44x	16	44x	33	10x	56	44x	56	44x	16	44x
Tyrannidae	*Myiopagis caniceps*				2	5	11	4	11	4	12	7	11	9	13	8	15	10	15	14	14

Family	Species	IUCN	Ag	R	Range Source	Percentage loss in geographic range 2050 TG H		2050 AM H		2050 GO H		2050 OS H		2100 TG H		2100 AM H		2100 GO H		2100 OS H	
						H	C	H	C	H	C	H	C	H	C	H	C	H	C	H	C
Tyrannidae	*Myiopagis subplacens*				2	6	8	2	6	5	10	5	2	6	8	3	8	6	13	8	5
Tyrannidae	*Myiopagis flavivertex*				2	6	11	4	11	5	11	9	10	10	12	8	14	14	15	17	13
Tyrannidae	*Myiopagis cotta*			x	2	16	0x	16	4x	11	4x	40	4x	40	0x	16	4x	11	4x	40	4x
Tyrannidae	*Myiopagis viridicata*				2	10	14	9	13	9	14	16	12	14	17	15	19	21	19	26	16
Tyrannidae	*Pseudelaenia leucospodia*				2	0	13	0	17	0	17	0	17	0	17	0	25	0	25	0	25
Tyrannidae	*Elaenia martinica*			x	2	7	4x	27	3x	10	3x	26	1x	18	4x	31	3x	18	3x	34	1x
Tyrannidae	*Elaenia flavogaster*				2	10	14	8	12	9	13	15	10	14	17	13	16	20	17	25	13
Tyrannidae	*Elaenia spectabilis*				2	5	17	4	13	4	14	9	11	8	21	8	20	12	23	16	18
Tyrannidae	*Elaenia albiceps*				2	4	9	5	8	4	9	6	8	6	13	8	15	10	16	10	15
Tyrannidae	*Elaenia parvirostris*				2	10	9	8	7	11	7	13	6	14	12	14	14	21	14	22	13
Tyrannidae	*Elaenia strepera*				2	10	9	12	3	4	7	9	5	11	9	16	6	11	14	15	7
Tyrannidae	*Elaenia mesoleuca*				2	17	9	15	6	23	6	24	6	25	15	26	11	38	8	38	10
Tyrannidae	*Elaenia gigas*				2	16	4	17	3	19	4	31	4	26	4	30	6	36	4	47	4
Tyrannidae	*Elaenia pelzelni*				2	8	5	8	3	6	4	13	3	13	5	13	3	20	4	22	3
Tyrannidae	*Elaenia cristata*				2	8	17	5	15	6	16	12	12	11	21	9	18	17	20	21	15
Tyrannidae	*Elaenia ruficeps*				2	4	10	3	10	3	10	5	11	5	13	6	15	8	16	8	17
Tyrannidae	*Elaenia chiriquensis*				2	9	13	6	12	8	13	13	10	13	17	11	16	19	17	22	14
Tyrannidae	*Elaenia frantzii*		H		2	0	2	0	3	0	4	0	3	0	3	0	5	0	9	0	5
Tyrannidae	*Elaenia obscura*				2	17	7	16	6	21	6	25	6	25	11	26	11	36	8	37	10
Tyrannidae	*Elaenia dayi*			x	2	11	11x	0	23x	9	14x	0	23x	11	14x	5	29x	11	23x	2	34x
Tyrannidae	*Elaenia pallatangae*				2	8	16	10	16	6	18	10	19	15	20	14	22	14	26	14	27
Tyrannidae	*Elaenia fallax*			x	2	9	2x	11	2x	8	4x	20	1x	18	2x	11	2x	8	4x	20	1x
Tyrannidae	*Mecocerculus leucophrys*				2	9	9	9	9	6	9	10	10	12	12	14	14	16	15	13	15
Tyrannidae	*Mecocerculus hellmayri*				2	11	0	7	0	8	0	11	0	15	0	14	4	14	4	14	4
Tyrannidae	*Mecocerculus calopterus*				2	4	14	8	9	8	15	11	8	5	14	11	9	10	15	12	8
Tyrannidae	*Mecocerculus minor*				2	9	10	17	7	9	11	20	11	17	11	24	14	33	18	29	15
Tyrannidae	*Mecocerculus stictopterus*				2	14	4	16	3	13	5	21	6	20	6	20	11	29	9	25	9
Tyrannidae	*Serpophaga cinerea*			x	2	17	3x	19	2x	15	4x	22	6x	24	5x	24	12x	30	9x	28	11x
Tyrannidae	*Serpophaga nigricans*				2	6	8	5	6	8	6	8	5	9	11	8	10	12	11	12	9
Tyrannidae	*Serpophaga hypoleuca*				2	18	9	22	5	19	9	19	5	32	10	31	5	39	9	41	5
Tyrannidae	*Serpophaga subcristata*				2	7	9	6	8	8	8	10	7	10	12	10	13	15	15	17	12
Tyrannidae	*Serpophaga munda*				2	4	9	9	5	3	7	9	5	4	9	10	7	8	10	10	7
Tyrannidae	*Serpophaga griseiceps*				14	3	10	3	7	2	7	4	7	3	13	4	15	6	19	8	16
Tyrannidae	*Inezia tenuirostris*				2	26	11	19	20	20	17	21	11	27	28	21	28	21	38	25	32
Tyrannidae	*Inezia inornata*				2	10	20	6	23	6	22	10	21	14	25	10	33	17	34	19	32
Tyrannidae	*Inezia subflava*				2	10	12	6	12	7	13	11	11	15	13	12	14	23	15	23	13
Tyrannidae	*Inezia caudata*				14	14	8	12	8	13	8	17	6	17	11	17	12	23	16	25	11
Tyrannidae	*Stigmatura napensis*			x	2	12	9x	15	1x	13	5x	10	1x	22	9x	23	1x	25	5x	28	1x
Tyrannidae	*Stigmatura bahiae*				14	16	23	12	21	16	21	26	8	18	33	17	26	20	25	31	12
Tyrannidae	*Stigmatura budytoides*				2	2	10	1	10	2	10	3	9	2	13	2	16	3	18	4	15
Tyrannidae	*Stigmatura gracilis*				14	17	23	8	23	18	20	28	11	20	33	15	30	25	26	33	16
Tyrannidae	*Uromyias agilis*			x	2	24	2x	31	2x	28	2x	43	0x	33	11x	50	17x	64	5x	50	10x
Tyrannidae	*Uromyias agraphia*				2	7	0	6	0	6	0	8	6	11	0	6	10	17	5	12	6
Tyrannidae	*Anairetes alpinus*	T		x	2	0	0x	18	0x	18	0x	18	0x	0	0x	18	0x	42	0x	42	0x

Family	Species	IUCN	Ag	R	Range Source	Percentage loss in geographic range 2050 TG H	TG C	AM H	AM C	GO H	GO C	OS H	OS C	2100 TG H	TG C	AM H	AM C	GO H	GO C	OS H	OS C
Tyrannidae	*Anairetes nigrocristatus*		H	x	2	0	0x	0	0x	0	0x	0	0x	0	0x	0	0x	0	0x	0	0x
Tyrannidae	*Anairetes reguloides*				2	0	33	14	23	14	36	18	17	0	56	15	54	20	53	29	38
Tyrannidae	*Anairetes flavirostris*				2	5	13	4	14	4	14	6	12	6	17	6	22	8	24	10	19
Tyrannidae	*Anairetes fernandezianus*			x	2	0	0x	0	0x	0	0x	0	0x	0	0x	0	0x	0	0x	0	0x
Tyrannidae	*Anairetes parulus*				2	4	9	4	9	3	9	5	8	6	12	6	15	8	17	8	15
Tyrannidae	*Tachuris rubrigastra*				2	3	7	3	7	3	7	3	6	4	9	4	11	5	13	6	11
Tyrannidae	*Culicivora caudacuta*	T			2	7	15	5	6	7	10	11	5	11	23	9	10	15	13	16	7
Tyrannidae	*Polystictus pectoralis*				2	6	9	3	6	5	8	8	5	8	13	6	8	11	10	12	7
Tyrannidae	*Polystictus superciliaris*				2	13	4	5	2	9	3	13	0	14	4	8	4	14	5	21	0
Tyrannidae	*Pseudocolopteryx sclateri*				2	2	9	2	6	3	6	5	6	3	10	7	8	7	9	9	9
Tyrannidae	*Pseudocolopteryx dinellian*				2	0	0	0	0	0	0	0	0	0	0	0	0	0	0	0	0
Tyrannidae	*Pseudocolopteryx acutipen*				2	5	8	4	9	4	9	10	6	6	10	7	13	10	13	13	8
Tyrannidae	*Pseudocolopteryx flaviventr*				2	3	4	2	5	4	5	4	4	4	6	4	9	6	10	6	9
Tyrannidae	*Euscarthmus meloryphus*				2	9	18	7	16	9	17	13	14	13	22	12	21	18	21	21	18
Tyrannidae	*Euscarthmus rufomarginat*				2	4	16	2	5	2	11	7	3	7	24	6	7	6	14	12	5
Tyrannidae	*Phylloscartes ophthalmicus*				2	17	6	13	5	15	6	24	6	22	7	23	10	31	10	28	9
Tyrannidae	*Phylloscartes venezuelanu*			x	2	40	0x	1	0x	41	0x	16	0x	41	0x	41	1x	42	0x	16	1x
Tyrannidae	*Phylloscartes lanyoni*	T		x	2	10	7x	0	0x	0	7x	1	0x	10	7x	10	7x	0	7x	1	7x
Tyrannidae	*Phylloscartes orbitalis*				2	18	8	12	6	12	8	26	7	23	8	18	12	29	11	30	8
Tyrannidae	*Phylloscartes poecilotis*				2	14	8	14	5	12	8	24	9	23	10	20	17	32	15	32	13
Tyrannidae	*Phylloscartes eximius*				2	22	10	20	9	29	7	33	8	31	18	33	16	47	11	48	14
Tyrannidae	*Phylloscartes nigrifrons*				2	6	24	9	26	2	28	3	30	14	32	11	34	5	40	5	43
Tyrannidae	*Phylloscartes chapmani*				2	5	25	8	27	2	29	2	31	11	32	10	35	4	41	5	45
Tyrannidae	*Phylloscartes gualaquizae*			x	2	24	15x	20	12x	12	17x	26	17x	35	15x	42	32x	62	20x	39	30x
Tyrannidae	*Phylloscartes flaviventris*			x	2	47	0x	0	0x	47	0x	22	0x	47	0x	47	0x	47	0x	22	0x
Tyrannidae	*Phylloscartes parkeri*				14	23	13	14	8	13	13	39	8	39	13	23	21	45	13	57	8
Tyrannidae	*Phylloscartes roquettei*	T		x	14	0	0x	0	0x	0	0x	0	0x	0	0x	0	0x	0	0x	0	0x
Tyrannidae	*Phylloscartes paulistus*				2	20	13	18	14	28	10	31	12	30	25	33	25	53	15	54	22
Tyrannidae	*Phylloscartes oustaleti*				2	15	19x	12	26x	19	18x	18	22x	15	49x	24	43x	56	22x	34	45x
Tyrannidae	*Phylloscartes difficilis*				2	26	10	15	20	31	12	25	17	27	32	32	35	53	18	38	31
Tyrannidae	*Phylloscartes ceciliae*	T		x	2	0	97x	0	97x	0	97x	0	97x	0	97x	0	97x	0	97x	0	97x
Tyrannidae	*Phylloscartes ventralis*				2	16	6	15	5	21	4	25	5	25	10	26	10	36	7	37	9
Tyrannidae	*Phylloscartes kronei*	T		x	2	44	20x	4	40x	3	40x	4	40x	44	23x	10	57x	12	54x	50	43x
Tyrannidae	*Phylloscartes beckeri*	T		x	14	26	72x	26	72x	99	0x	99	0x	26	72x	26	72x	99	0x	99	0x
Tyrannidae	*Phylloscartes flavovirens*			x	2	6	26x	21	32x	9	32x	41	26x	6	31x	21	32x	31	37x	51	31x
Tyrannidae	*Phylloscartes virescens*				2	2	19	2	18	3	18	5	18	4	19	4	21	7	22	7	20
Tyrannidae	*Phylloscartes superciliaris*			x	2	12	3x	22	6x	20	6x	32	4x	18	3x	25	7x	35	6x	39	4x
Tyrannidae	*Phylloscartes sylviolus*				2	21	11	19	11	30	8	36	9	31	20	34	19	53	11	54	17
Tyrannidae	*Myiornis albiventris*				2	21	0	14	0	14	0	30	1	29	0	25	4	38	0	42	2
Tyrannidae	*Myiornis auricularis*				2	21	8	18	8	29	6	29	7	31	14	31	14	46	10	47	13
Tyrannidae	*Myiornis atricapillus*				2	17	5	21	8	17	6	23	5	25	7	23	9	26	11	35	8
Tyrannidae	*Myiornis ecaudatus*				2	5	11	3	11	4	11	8	11	9	12	7	14	14	14	15	13
Tyrannidae	*Lophotriccus pileatus*				2	21	7	19	7	22	8	35	6	29	9	27	13	37	12	43	8
Tyrannidae	*Lophotriccus vitiosus*				2	4	5	3	5	4	5	8	5	8	5	8	5	12	6	16	5

Family	Species	IUCN	Ag	R	Range Source	Percentage loss in geographic range 2050 TG H	C	AM H	C	GO H	C	OS H	C	2100 TG H	C	AM H	C	GO H	C	OS H	C
Tyrannidae	*Lophotriccus eulophotes*				2	1	10	0	10	2	10	4	10	4	13	7	17	6	19	8	13
Tyrannidae	*Lophotriccus galeatus*				2	5	12	3	12	4	11	8	11	9	13	6	15	16	15	15	14
Tyrannidae	*Atalotriccus pilaris*				2	18	3	11	4	15	2	20	2	20	4	18	6	32	4	33	2
Tyrannidae	*Oncostoma cinereigulare*				2	13	3	21	3	18	5	30	3	16	4	26	10	24	8	36	5
Tyrannidae	*Oncostoma olivaceum*				2	13	5	17	9	12	8	23	5	19	8	20	9	25	14	33	9
Tyrannidae	*Cnipodectes subbrunneus*				2	3	2	2	2	2	2	5	1	7	2	8	2	7	2	13	2
Tyrannidae	*Rhynchocyclus brevirostris*				2	14	4	25	4	17	6	32	4	18	5	30	10	25	10	39	6
Tyrannidae	*Rhynchocyclus olivaceus*				2	7	10	5	10	6	9	10	9	11	11	10	12	17	13	19	12
Tyrannidae	*Rhynchocyclus fulvipectus*				2	13	7	12	4	12	6	24	7	22	7	20	13	31	10	32	10
Tyrannidae	*Rhynchocyclus pacificus*				2	15	3	17	5	16	1	14	4	26	3	17	5	18	5	25	4
Tyrannidae	*Tolmomyias sulphurescens*				2	7	12	6	12	7	12	12	11	11	14	11	16	17	16	20	14
Tyrannidae	*Tolmomyias traylori*				14	5	2	2	1	5	0	9	1	9	2	15	1	10	0	25	1
Tyrannidae	*Tolmomyias assimilis*				2	5	10	3	10	4	10	7	9	9	11	7	12	12	12	14	11
Tyrannidae	*Tolmomyias flavotectus*				14	17	5	23	8	15	6	24	6	26	7	24	8	24	10	33	8
Tyrannidae	*Tolmomyias poliocephalus*				2	5	9	3	9	5	9	8	8	9	10	8	11	15	11	17	11
Tyrannidae	*Tolmomyias flaviventris*				2	7	15	5	15	6	16	12	12	11	18	9	18	17	20	20	16
Tyrannidae	*Tolmomyias viridiceps*				14	6	5	4	5	5	5	10	4	10	6	10	6	13	6	18	6
Tyrannidae	*Platyrinchus saturatus*				2	5	12	3	11	4	12	7	11	8	13	6	14	15	15	13	14
Tyrannidae	*Platyrinchus cancrominus*				2	12	4	20	3	14	4	27	3	15	5	26	10	21	9	34	5
Tyrannidae	*Platyrinchus mystaceus*				2	11	15	8	12	11	13	16	10	15	19	14	16	21	18	26	14
Tyrannidae	*Platyrinchus coronatus*				2	5	9	4	8	4	8	8	8	8	9	8	10	13	11	15	10
Tyrannidae	*Platyrinchus flavigularis*				2	24	5	20	4	22	5	31	9	40	5	36	13	52	6	41	12
Tyrannidae	*Platyrinchus platyrhynchos*				2	4	9	3	9	3	9	7	9	8	10	6	11	12	11	14	11
Tyrannidae	*Platyrinchus leucoryphus*	T			2	21	13	20	13	30	10	32	11	31	25	34	24	54	15	52	22
Tyrannidae	*Onychorhynchus coronatus*				2	5	10	4	10	4	10	8	9	9	11	8	12	14	12	16	11
Tyrannidae	*Onychorhynchus mexicanu*				14	14	4	22	5	17	6	30	4	18	5	28	10	25	11	37	6
Tyrannidae	*Onychorhynchus occidenta*	T		x	2	12	16x	2	14x	4	16x	21	0x	14	16x	4	14x	7	16x	21	0x
Tyrannidae	*Onychorhynchus swainsoni*	T			2	23	17	20	18	28	12	20	17	25	33	30	30	50	16	33	31
Tyrannidae	*Myiotriccus ornatus*				2	14	7	10	7	13	7	21	7	19	7	18	11	25	8	27	8
Tyrannidae	*Myiophobus phoenicomitra*				2	16	6	14	4	14	6	19	6	22	6	23	12	35	7	29	11
Tyrannidae	*Myiophobus inornatus*				2	15	6	9	0	12	2	19	5	19	6	17	10	25	7	23	9
Tyrannidae	*Myiophobus roraimae*				2	7	17	7	19	3	20	3	22	13	23	10	25	8	29	8	33
Tyrannidae	*Myiophobus pulcher*				2	14	10	14	5	15	6	22	7	14	13	24	6	27	6	22	7
Tyrannidae	*Myiophobus lintoni*			x	2	40	39x	42	30x	0	59x	5	59x	40	39x	43	30x	20	68x	20	68x
Tyrannidae	*Myiophobus ochraceiventri*				2	5	3	6	0	7	0	11	5	10	3	10	8	18	7	13	8
Tyrannidae	*Myiophobus fasciatus*		H		2	0	13	0	11	0	12	0	10	0	17	0	16	0	17	0	13
Tyrannidae	*Myiophobus cryptoxanthus*				2	7	9	7	8	13	9	27	6	10	9	21	12	33	10	38	8
Tyrannidae	*Neopipo cinnamomea*				2	3	8	3	7	3	8	7	7	6	8	7	8	9	9	13	8
Tyrannidae	*Myiobius erythrurus*				2	6	11	5	11	5	11	10	10	10	12	9	13	16	14	18	12
Tyrannidae	*Myiobius villosus*				2	13	7	13	5	13	6	27	5	22	7	20	11	28	10	33	7
Tyrannidae	*Myiobius barbatus*				2	4	8	3	8	4	8	8	7	9	9	8	9	14	10	16	9
Tyrannidae	*Myiobius mastacalis*				14	12	26	8	25	14	23	11	23	18	38	13	36	27	32	22	33
Tyrannidae	*Myiobius sulphureipygius*				14	14	5	23	5	17	6	30	4	20	6	29	11	25	10	38	6
Tyrannidae	*Myiobius atricaudus*				2	7	7	5	7	6	8	12	6	12	8	11	9	19	9	24	7

Family	Species	IUCN	Ag	R	Range Source	Percentage loss in geographic range 2050 TG H	TG C	AM H	AM C	GO H	GO C	OS H	OS C	2100 TG H	TG C	AM H	AM C	GO H	GO C	OS H	OS C
Tyrannidae	*Pyrrhomyias cinnamomea*				2	13	5	13	4	12	5	17	6	17	7	19	8	21	9	21	7
Tyrannidae	*Hirundinea ferruginea*				2	10	16	7	13	9	14	14	10	13	21	12	17	18	18	22	14
Tyrannidae	*Cnemotriccus fuscatus*				2	7	12	5	12	6	12	10	11	11	15	9	16	17	17	19	15
Tyrannidae	*Lathrotriccus euleri*				2	8	13	6	13	7	13	12	12	13	16	11	17	19	17	23	15
Tyrannidae	*Lathrotriccus griseipectus*	T			2	7	13	1	9	1	15	7	2	7	13	1	11	2	17	8	4
Tyrannidae	*Aphanotriccus capitalis*	T			2	32	0	51	7	59	7	79	0	50	0	64	16	64	7	83	0x
Tyrannidae	*Aphanotriccus audax*				2	14	6	23	13	10	13	25	6	15	13	25	14	22	22	30	13
Tyrannidae	*Xenotriccus callizonus*			x	2	2	0x	31	0x	20	0x	34	0x	5	0x	34	3x	22	0x	34	0x
Tyrannidae	*Xenotriccus mexicanus*			x	2	9	1x	13	0x	10	1x	11	0x	9	1x	13	8x	11	1x	11	0x
Tyrannidae	*Mitrephanes phaeocercus*				2	16	8	28	5	17	7	34	5	21	9	30	11	23	15	41	9
Tyrannidae	*Mitrephanes olivaceus*				2	13	3	6	0	11	1	17	5	18	3	13	9	23	7	19	6
Tyrannidae	*Contopus cooperi*				2	5	19	7	16	7	18	8	15	7	23	10	24	12	26	13	22
Tyrannidae	*Contopus pertinax*				2	9	9	20	6	10	9	26	6	12	13	20	18	14	21	29	14
Tyrannidae	*Contopus lugubris*			x	2	15	12x	25	22x	21	22x	24	22x	18	12x	25	25x	49	25x	48	22x
Tyrannidae	*Contopus fumigatus*				2	14	8	11	9	10	10	14	10	19	10	16	13	20	14	19	14
Tyrannidae	*Contopus ochraceus*			x	2	16	26x	37	39x	7	39x	7	39x	16	26x	37	39x	57	39x	44	39x
Tyrannidae	*Contopus sordidulus*				2	3	19	6	17	4	19	8	16	4	24	8	26	7	30	12	24
Tyrannidae	*Contopus virens*				2	12	19	19	9	18	14	18	6	19	21	26	11	32	16	26	6
Tyrannidae	*Contopus cinereus*				2	12	14	11	11	12	12	19	9	16	17	17	16	23	17	28	12
Tyrannidae	*Contopus punensis*				14	6	6	8	6	7	6	8	4	8	8	12	10	15	9	11	8
Tyrannidae	*Contopus nigrescens*				2	9	2	5	2	17	2	33	2	14	2	23	5	41	2	51	3
Tyrannidae	*Contopus albogularis*				2	0	26	0	26	0	26	0	26	0	26	0	26	0	31	0	26
Tyrannidae	*Contopus caribaeus*				2	11	11	33	10	23	8	40	4	21	11	36	13	28	8	46	4
Tyrannidae	*Contopus pallidus*			x	2	17	0x	17	5x	11	5x	44	5x	44	0x	17	5x	11	5x	44	5x
Tyrannidae	*Contopus hispaniolensis*				2	11	9	16	6	11	11	23	0	11	11	16	7	11	11	23	0
Tyrannidae	*Contopus latirostris*			x	2	2	7x	28	0x	2	7x	28	0x	2	7x	54	0x	21	7x	54	0x
Tyrannidae	*Empidonax flaviventris*				2	5	16	8	14	9	15	8	13	7	20	11	19	13	20	12	18
Tyrannidae	*Empidonax virescens*				2	15	21	23	9	21	14	21	6	24	26	31	14	42	19	33	7
Tyrannidae	*Empidonax alnorum*				2	5	20	8	17	8	18	8	16	7	25	11	25	13	26	13	23
Tyrannidae	*Empidonax traillii*				2	2	14	3	11	3	13	3	9	3	16	4	16	6	19	5	14
Tyrannidae	*Empidonax albigularis*		H		2	0	4	0	2	0	4	0	1	0	4	0	5	0	5	0	2
Tyrannidae	*Empidonax minimus*				2	6	20	9	16	9	19	9	14	8	24	12	21	14	24	14	19
Tyrannidae	*Empidonax hammondii*				2	4	24	8	21	6	23	9	20	5	32	11	33	12	37	17	28
Tyrannidae	*Empidonax wrightii*				2	1	7	1	7	2	7	2	6	1	8	2	10	3	13	4	9
Tyrannidae	*Empidonax oberholseri*				2	2	20	4	17	3	19	5	15	2	24	5	24	6	28	9	21
Tyrannidae	*Empidonax affinis*				2	14	7	29	3	17	5	37	2	18	8	30	13	22	11	41	5
Tyrannidae	*Empidonax difficilis*				2	8	33	15	26	13	29	19	21	10	42	21	39	24	43	32	29
Tyrannidae	*Empidonax occidentalis*				2	4	13	10	11	5	12	12	10	6	17	11	21	10	25	18	18
Tyrannidae	*Empidonax flavescens*				2	11	2	19	4	15	6	22	4	13	2	19	7	21	7	28	4
Tyrannidae	*Empidonax fulvifrons*				2	11	11	24	8	13	11	30	7	14	13	25	21	17	23	35	18
Tyrannidae	*Empidonax atriceps*			x	2	14	23x	34	33x	6	33x	9	33x	16	23x	34	36x	52	36x	43	33x
Tyrannidae	*Sayornis phoebe*				2	7	16	12	10	11	13	11	8	11	19	16	13	19	17	16	11
Tyrannidae	*Sayornis saya*				2	0	21	1	20	1	21	1	19	1	26	1	30	1	33	2	29
Tyrannidae	*Sayornis nigricans*				2	9	14	14	12	9	14	19	11	11	16	17	22	15	24	23	19

Family	Species	IUCN	Ag	R	Range Source	Percentage loss in geographic range 2050 TG H	TG C	AM H	AM C	GO H	GO C	OS H	OS C	2100 TG H	TG C	AM H	AM C	GO H	GO C	OS H	OS C
Tyrannidae	*Pyrocephalus rubinus*		H		2	0	11	0	10	0	11	0	9	0	14	0	18	0	19	0	16
Tyrannidae	*Pyrocephalus nanus*			x	14	0	13x	0	13x	0	8x	0	13x	0	13x	0	31x	0	41x	0	31x
Tyrannidae	*Silvicultrix frontalis*				14	21	7	26	5	15	8	32	6	28	9	43	9	48	15	36	12
Tyrannidae	*Ochthoeca spodionota*				14	4	8	6	3	6	2	6	4	6	8	8	4	12	3	6	5
Tyrannidae	*Silvicultrix jelskii*				2	4	3	12	5	5	6	7	6	4	7	12	6	11	7	11	7
Tyrannidae	*Silvicultrix diadema*				2	25	2	29	3	20	4	34	2	35	5	40	12	44	14	40	9
Tyrannidae	*Silvicultrix pulchella*				2	6	4	6	1	7	1	6	3	7	4	8	4	13	3	8	4
Tyrannidae	*Ochthoeca cinnamomeiven*				2	18	5	21	4	12	6	26	5	26	6	29	8	29	8	31	9
Tyrannidae	*Ochthoeca thoracica*				14	9	3	4	0	7	1	11	4	13	3	10	6	16	5	13	7
Tyrannidae	*Ochthoeca nigrita*			x	14	30	0x	9	2x	24	2x	61	2x	30	9x	9	31x	47	27x	63	2x
Tyrannidae	*Ochthoeca rufipectoralis*				2	14	4	15	2	11	3	16	3	18	4	24	4	29	6	20	5
Tyrannidae	*Ochthoeca fumicolor*				2	17	5	18	2	12	4	22	5	22	7	28	5	30	9	25	7
Tyrannidae	*Ochthoeca leucophrys*		H		2	0	19	0	21	0	21	0	16	0	26	0	28	0	29	0	22
Tyrannidae	*Ochthoeca piurae*			x	2	0	11x	14	11x	6	12x	14	11x	0	20x	14	20x	14	20x	14	20x
Tyrannidae	*Ochthoeca salvini*			x	14	0	12x	0	35x	0	35x	0	35x	0	35x	0	38x	0	39x	0	38x
Tyrannidae	*Colorhamphus parvirostris*				2	7	19	6	17	9	18	6	18	8	29	10	34	13	34	11	33
Tyrannidae	*Ochthornis littoralis*				2	11	12	11	7	12	9	12	4	21	12	19	7	27	9	28	5
Tyrannidae	*Cnemarchus erythropygius*				2	15	5	13	5	11	5	20	4	18	6	28	6	36	6	24	6
Tyrannidae	*Myiotheretes striaticollis*				2	10	5	11	4	8	4	15	3	13	7	17	8	20	9	19	6
Tyrannidae	*Myiotheretes pernix*	T		x	2	0	0x	68	0x	68	0x	0	0x	68	0x	68	0x	68	0x	68	0x
Tyrannidae	*Myiotheretes fumigatus*				2	19	4	21	5	14	6	28	7	26	6	32	11	38	12	32	10
Tyrannidae	*Myiotheretes fuscorufus*				2	12	4	8	1	9	1	18	4	19	4	13	7	23	4	18	7
Tyrannidae	*Xolmis pyrope*				2	7	12	6	11	6	12	6	12	9	20	10	21	12	22	11	21
Tyrannidae	*Xolmis cinerea*				2	8	13	5	11	7	12	12	9	12	18	10	15	17	16	21	13
Tyrannidae	*Xolmis coronata*				2	2	11	0	12	1	13	1	11	2	15	1	22	2	25	2	22
Tyrannidae	*Xolmis velata*				2	8	17	5	12	5	14	11	10	11	22	9	15	13	18	18	13
Tyrannidae	*Xolmis irupero*				2	7	8	5	7	7	8	9	6	9	11	8	11	11	12	13	9
Tyrannidae	*Xolmis dominicana*	T			2	11	1	10	1	17	0	16	1	16	1	19	1	24	0	26	1
Tyrannidae	*Xolmis rubetra*				2	1	8	1	10	1	10	1	9	2	12	2	18	2	22	3	19
Tyrannidae	*Xolmis salinarum*			x	2	0	0x	0	0x	0	0x	0	0x	0	0x	0	0x	0	0x	0	0x
Tyrannidae	*Xolmis rufiventris*				2	0	2	0	2	0	2	0	2	0	3	0	3	0	3	1	3
Tyrannidae	*Agriornis montana*				2	5	10	4	11	3	11	6	10	6	13	7	17	8	19	10	15
Tyrannidae	*Agriornis andicola*	T			2	11	14	11	15	7	16	15	12	14	20	16	23	20	23	24	16
Tyrannidae	*Agriornis livida*		H		2	0	8	0	7	0	8	0	8	0	15	0	15	0	18	0	16
Tyrannidae	*Agriornis microptera*				2	1	3	0	5	0	5	1	5	1	5	1	9	1	13	1	12
Tyrannidae	*Agriornis murina*				2	1	6	0	7	0	7	1	7	1	8	2	13	1	17	2	15
Tyrannidae	*Polioxolmis rufipennis*				2	13	13	13	14	12	13	16	13	16	19	17	21	21	21	24	16
Tyrannidae	*Muscisaxicola maculirostris*				2	5	11	5	12	4	12	7	10	7	15	7	18	10	19	12	16
Tyrannidae	*Muscisaxicola fluviatilis*				2	6	12	4	10	5	10	6	11	9	12	6	13	12	11	10	13
Tyrannidae	*Muscisaxicola macloviana*				2	3	13	4	13	3	14	3	13	6	23	6	25	7	25	9	24
Tyrannidae	*Muscisaxicola capistrata*				2	0	2	0	2	0	2	0	2	0	5	0	6	0	6	0	6
Tyrannidae	*Muscisaxicola rufivertex*				2	6	15	6	16	4	16	8	13	8	20	9	24	12	25	16	20
Tyrannidae	*Muscisaxicola juninensis*				2	11	17	10	19	7	18	15	14	14	23	15	27	18	28	25	19
Tyrannidae	*Muscisaxicola albilora*				2	7	13	6	12	4	13	10	12	11	22	10	21	14	21	16	22

Family	Species	IUCN	Ag	R	Range Source	Percentage loss in geographic range 2050 TG H	TG C	AM H	AM C	GO H	GO C	OS H	OS C	2100 TG H	TG C	AM H	AM C	GO H	GO C	OS H	OS C
Tyrannidae	*Muscisaxicola alpina*				2	20	1	29	1	27	1	49	0	26	4	53	2	66	3	49	1
Tyrannidae	*Muscisaxicola grisea*				14	5	23	7	24	7	22	6	21	5	30	11	33	14	31	14	27
Tyrannidae	*Muscisaxicola cinerea*				2	8	15	7	16	4	16	11	14	9	19	11	21	12	23	17	18
Tyrannidae	*Muscisaxicola albifrons*				2	11	23	9	24	9	24	12	22	12	32	15	33	20	33	23	28
Tyrannidae	*Muscisaxicola flavinucha*				2	4	7	4	8	2	8	4	8	6	12	7	15	8	18	11	16
Tyrannidae	*Muscisaxicola frontalis*				2	5	8	4	7	3	8	7	8	7	10	9	13	8	16	14	17
Tyrannidae	*Muscigralla brevicauda*				2	3	20	3	17	4	23	5	15	3	29	4	37	9	35	10	30
Tyrannidae	*Lessonia oreas*				2	7	14	7	16	5	15	9	13	9	18	10	22	12	22	16	16
Tyrannidae	*Lessonia rufa*				2	2	7	2	7	1	8	1	8	2	11	3	14	3	17	3	16
Tyrannidae	*Knipolegus striaticeps*				2	3	11	3	10	3	10	4	10	4	14	5	19	9	23	10	20
Tyrannidae	*Knipolegus hudsoni*				2	1	13	0	15	1	15	1	14	1	18	0	22	1	24	1	21
Tyrannidae	*Knipolegus poecilocercus*				2	6	10	4	10	4	10	7	9	8	12	7	13	13	13	14	12
Tyrannidae	*Knipolegus signatus*				2	7	4	7	1	6	2	10	4	9	4	10	6	14	7	12	7
Tyrannidae	*Knipolegus cyanirostris*				2	13	4	11	4	18	3	18	3	18	10	19	8	30	5	31	6
Tyrannidae	*Knipolegus poecilurus*		H		2	0	5	0	5	0	7	0	7	0	7	0	11	0	13	0	12
Tyrannidae	*Knipolegus orenocensis*				2	20	8	24	4	19	10	20	4	35	9	34	5	42	10	42	4
Tyrannidae	*Knipolegus aterrimus*				2	6	8	5	8	4	8	7	7	7	10	7	14	8	16	9	14
Tyrannidae	*Knipolegus franciscanus*				14	0	3	4	5	0	6	4	0	0	12	7	5	3	6	7	0
Tyrannidae	*Knipolegus nigerrimus*				2	15	16	11	17	18	13	24	9	18	23	14	23	26	17	29	10
Tyrannidae	*Knipolegus lophotes*				2	11	13	7	7	13	9	15	5	15	20	15	11	21	12	22	8
Tyrannidae	*Hymenops perspicillatus*				2	3	4	2	4	4	5	4	4	4	6	4	8	6	9	6	8
Tyrannidae	*Fluvicola pica*				2	16	4	11	5	14	4	18	3	18	7	17	9	27	10	26	7
Tyrannidae	*Fluvicola albiventer*				2	7	16	4	11	5	14	11	8	10	20	8	14	12	18	18	12
Tyrannidae	*Fluvicola nengeta*				2	9	18	8	15	9	16	15	10	10	22	12	20	17	21	22	15
Tyrannidae	*Arundinicola leucocephala*				2	10	13	8	9	9	11	14	7	13	18	12	13	19	15	23	10
Tyrannidae	*Alectrurus tricolor*	T			2	7	14	6	6	8	9	11	5	11	21	10	9	15	12	16	7
Tyrannidae	*Alectrurus risora*	T			2	2	2	1	1	2	1	2	1	2	2	1	2	2	2	2	2
Tyrannidae	*Gubernetes yetapa*				2	7	14	5	5	8	9	10	4	12	20	9	8	14	12	14	6
Tyrannidae	*Satrapa icterophrys*				2	8	13	6	10	8	11	12	9	11	18	10	15	16	16	19	13
Tyrannidae	*Colonia colonus*				2	9	15	7	12	8	13	14	10	13	19	12	15	18	17	23	13
Tyrannidae	*Machetornis rixosus*		H		2	0	11	0	9	0	9	0	7	0	14	0	13	0	13	0	10
Tyrannidae	*Muscipipra vetula*				2	18	6	17	5	25	3	25	5	25	11	28	12	39	8	41	9
Tyrannidae	*Attila phoenicurus*				2	25	9	22	9	34	7	35	8	35	18	39	17	54	11	57	15
Tyrannidae	*Attila cinnamomeus*				2	4	9	3	9	3	9	7	8	8	10	6	11	12	12	14	10
Tyrannidae	*Attila torridus*	T			2	9	11	3	8	4	13	10	0	9	11	4	8	7	13	12	0
Tyrannidae	*Attila citriniventris*				2	1	1	1	1	0	1	2	1	2	2	4	2	2	2	8	2
Tyrannidae	*Attila bolivianus*				2	7	14	4	15	5	15	9	14	12	16	9	19	16	18	18	17
Tyrannidae	*Attila rufus*				2	19	11	13	16	28	10	18	14	27	25	24	30	47	17	33	28
Tyrannidae	*Casiornis rufa*				2	7	17	4	11	5	14	9	10	10	22	8	16	13	20	14	15
Tyrannidae	*Casiornis fusca*				2	9	18	5	17	7	17	15	12	12	21	9	20	18	22	27	16
Tyrannidae	*Rhytipterna holerythra*				2	18	5	23	6	18	6	30	4	23	6	29	10	27	9	37	6
Tyrannidae	*Rhytipterna simplex*				2	5	11	4	11	4	12	8	11	9	13	8	13	14	14	16	13
Tyrannidae	*Rhytipterna immunda*				2	7	17	3	14	4	18	7	13	10	19	8	17	12	20	14	16
Tyrannidae	*Laniocera rufescens*				2	18	5	27	7	22	6	36	4	25	6	34	11	33	10	43	6

Family	Species	IUCN	Ag	R	Range Source	Percentage loss in geographic range 2050 TG H	TG C	AM H	AM C	GO H	GO C	OS H	OS C	2100 TG H	TG C	AM H	AM C	GO H	GO C	OS H	OS C
Tyrannidae	*Laniocera hypopyrra*				14	5	10	3	10	4	10	8	9	9	11	7	12	14	12	16	11
Tyrannidae	*Sirystes sibilator*				2	7	13	5	13	6	13	11	12	12	15	11	16	20	16	22	15
Tyrannidae	*Sirystes albogriseus*				14	15	6	8	10	6	10	17	0	20	10	12	10	18	14	23	4
Tyrannidae	*Myiarchus semirufus*			x	2	0	14x	0	14x	2	15x	0	14x	0	22x	0	31x	2	30x	2	29x
Tyrannidae	*Myiarchus yucatanensis*				2	6	3	19	0	14	3	26	2	8	4	22	11	16	9	32	5
Tyrannidae	*Myiarchus tuberculifer*				2	8	10	8	10	7	11	13	10	12	12	12	14	17	14	21	12
Tyrannidae	*Myiarchus barbirostris*			x	2	17	0x	17	5x	11	5x	44	5x	44	0x	17	5x	11	5x	44	5x
Tyrannidae	*Myiarchus swainsoni*				2	6	14	5	10	6	11	10	8	9	18	8	13	13	15	16	11
Tyrannidae	*Myiarchus venezuelensis*				2	21	8	17	9	22	11	16	5	31	14	22	13	32	23	28	16
Tyrannidae	*Myiarchus panamensis*				2	12	4	15	5	10	6	16	2	17	6	18	8	18	12	24	5
Tyrannidae	*Myiarchus ferox*				2	7	13	5	11	6	12	11	10	11	16	9	15	17	15	20	13
Tyrannidae	*Myiarchus cephalotes*				2	14	5	12	4	11	6	17	6	18	6	18	10	25	10	22	9
Tyrannidae	*Myiarchus phaeocephalus*				2	1	8	0	6	0	8	4	3	1	8	0	11	1	13	4	8
Tyrannidae	*Myiarchus apicalis*				2	0	0	0	0	0	0	0	0	0	0	0	0	0	0	0	0
Tyrannidae	*Myiarchus cinerascens*				2	2	18	4	15	2	17	6	14	3	21	5	27	4	31	8	24
Tyrannidae	*Myiarchus nuttingi*				2	15	12	24	6	14	10	36	5	19	12	24	18	17	16	38	10
Tyrannidae	*Myiarchus crinitus*		H		2	0	21	0	11	0	16	0	8	0	24	0	14	0	19	0	9
Tyrannidae	*Myiarchus tyrannulus*				2	9	14	8	13	8	13	14	11	12	18	12	19	18	20	22	16
Tyrannidae	*Myiarchus nugator*			x	2	0	0x	51	0x	0	0x	0	0x	0	0x	51	0x	0	0x	51	0x
Tyrannidae	*Myiarchus magnirostris*			x	2	0	13x	0	13x	0	8x	0	13x	0	13x	0	31x	0	41x	0	31x
Tyrannidae	*Myiarchus validus*			x	2	17	0x	17	5x	11	5x	44	5x	44	0x	17	5x	11	5x	44	5x
Tyrannidae	*Myiarchus sagrae*				2	12	11	34	11	23	8	42	5	22	11	37	13	28	9	49	5
Tyrannidae	*Myiarchus stolidus*				2	11	8	16	6	11	10	25	1	14	10	16	7	11	10	25	1
Tyrannidae	*Myiarchus antillarum*			x	2	18	0x	18	0x	18	0x	18	0x	18	0x	18	0x	18	0x	18	0x
Tyrannidae	*Myiarchus oberi*			x	2	1	16x	34	12x	18	5x	43	0x	1	16x	50	12x	38	5x	67	0x
Tyrannidae	*Deltarhynchus flammulatus*				2	3	12	14	5	7	11	24	5	3	16	14	31	9	15	27	5
Tyrannidae	*Ramphotrigon megacephal*				2	8	9	6	10	7	9	10	9	13	13	11	13	20	12	18	13
Tyrannidae	*Ramphotrigon fuscicauda*				2	12	5	10	5	13	5	20	4	19	6	20	6	25	6	29	6
Tyrannidae	*Ramphotrigon ruficauda*				2	4	11	3	11	3	12	7	11	8	13	7	13	13	14	15	13
Tyrannidae	*Tyrannus niveigularis*				2	1	5	0	6	0	6	1	6	1	6	0	15	1	16	1	16
Tyrannidae	*Tyrannus albogularis*				2	6	18	3	15	3	18	8	14	9	22	6	18	14	21	15	17
Tyrannidae	*Tyrannus melancholicus*		H		2	0	11	0	10	0	10	0	8	0	14	0	14	0	14	0	12
Tyrannidae	*Tyrannus couchii*		H		2	0	8	0	5	0	6	0	3	0	13	0	25	0	13	0	5
Tyrannidae	*Tyrannus vociferans*				2	7	16	15	13	8	15	19	12	9	18	15	23	11	26	21	21
Tyrannidae	*Tyrannus crassirostris*				2	13	8	27	4	16	8	35	3	16	10	27	15	21	13	39	6
Tyrannidae	*Tyrannus verticalis*		H		2	0	16	0	14	0	16	0	12	0	19	0	20	0	24	0	18
Tyrannidae	*Tyrannus forficatus*		H		2	0	11	0	8	0	10	0	6	0	15	0	17	0	19	0	11
Tyrannidae	*Tyrannus savana*		H		2	0	11	0	7	0	9	0	6	0	16	0	11	0	13	0	10
Tyrannidae	*Tyrannus tyrannus*		H		2	0	19	0	13	0	16	0	11	0	23	0	17	0	21	0	13
Tyrannidae	*Tyrannus dominicensis*				2	17	14	22	11	20	13	28	8	22	16	27	12	29	14	36	9
Tyrannidae	*Tyrannus caudifasciatus*				2	11	10	24	9	16	8	33	4	20	10	26	11	19	8	35	4
Tyrannidae	*Tyrannus cubensis*	T			2	8	6	24	6	13	5	28	3	14	6	26	8	17	5	34	3
Tyrannidae	*Empidonomus varius*				2	7	15	5	14	6	15	10	13	11	18	9	19	18	19	19	17
Tyrannidae	*Griseotyrannus aurantioatr*				2	7	13	4	11	5	12	10	10	10	16	7	15	14	17	17	14

Family	Species	IUCN	Ag	R	Range Source	Percentage loss in geographic range															
						2050								2100							
						TG		AM		GO		OS		TG		AM		GO		OS	
						H	C	H	C	H	C	H	C	H	C	H	C	H	C	H	C
Tyrannidae	*Tyrannopsis sulphurea*				2	5	11	3	11	3	11	8	10	8	12	6	13	14	14	16	12
Tyrannidae	*Megarynchus pitangua*				2	7	11	6	11	7	12	12	10	11	14	11	15	17	15	21	13
Tyrannidae	*Conopias albovittata*				2	16	4	23	8	22	6	31	5	24	6	28	9	33	9	41	7
Tyrannidae	*Conopias parva*				2	2	7	2	7	2	7	4	7	5	8	4	8	7	10	8	9
Tyrannidae	*Conopias trivirgata*				2	11	9	9	8	11	9	14	7	17	12	16	12	24	11	25	10
Tyrannidae	*Conopias cinchoneti*				2	11	5	14	5	14	5	27	4	24	7	22	13	32	10	36	5
Tyrannidae	*Myiodynastes hemichrysus*			x	2	19	11x	34	16x	14	16x	42	16x	25	11x	34	19x	34	19x	50	16x
Tyrannidae	*Myiodynastes chrysocepha*				2	14	4	14	4	14	4	22	3	21	5	19	9	25	8	27	5
Tyrannidae	*Myiodynastes bairdii*				2	0	10	0	10	1	11	1	10	0	13	0	21	2	22	2	21
Tyrannidae	*Myiodynastes maculatus*				2	7	11	5	10	6	11	10	9	10	13	9	13	15	14	18	12
Tyrannidae	*Myiodynastes luteiventris*				2	11	8	23	5	13	7	33	4	14	9	26	15	18	13	37	8
Tyrannidae	*Myiozetetes cayanensis*				2	8	14	5	13	6	14	11	12	12	16	9	16	18	18	20	15
Tyrannidae	*Myiozetetes similis*				2	8	11	7	9	8	10	13	8	12	14	12	13	17	13	21	10
Tyrannidae	*Myiozetetes granadensis*				2	7	6	6	7	6	7	10	6	11	8	11	9	13	9	17	8
Tyrannidae	*Myiozetetes luteiventris*				2	4	7	3	7	3	7	7	6	8	8	7	8	12	9	15	8
Tyrannidae	*Philohydor lictor*				2	8	16	5	13	6	14	12	10	11	20	9	16	16	19	20	13
Tyrannidae	*Pitangus sulphuratus*				2,13	7	11	6	10	7	10	12	8	11	14	10	14	16	15	19	12
Tyrannidae	*Phelpsia inornata*		H		2	0	2	0	2	0	1	0	1	0	2	0	2	0	1	0	1
Tyrannidae	*Schiffornis major*				2	4	8	2	7	2	8	5	7	6	9	6	9	8	9	11	8
Tyrannidae	*Schiffornis turdinus*				2	6	11	5	11	5	12	10	11	10	13	9	14	15	15	17	13
Tyrannidae	*Schiffornis virescens*				2	19	8	16	7	24	6	25	6	27	16	27	14	42	10	42	12
Tyrannidae	*Xenopsaris albinucha*				2	8	14	4	12	4	13	10	9	10	17	7	15	11	18	18	12
Tyrannidae	*Pachyramphus viridis*				2	8	17	6	15	10	17	13	14	12	22	12	21	20	22	22	19
Tyrannidae	*Pachyramphus xanthogeny*				2	16	6	21	4	18	6	34	7	31	6	41	10	57	7	51	12
Tyrannidae	*Pachyramphus versicolor*				2	10	5	14	6	12	6	23	5	20	6	21	12	31	10	31	8
Tyrannidae	*Pachyramphus cinnamome*				2	15	5	20	6	16	6	27	3	21	6	25	10	26	11	33	5
Tyrannidae	*Pachyramphus castaneus*				2	6	7	5	6	6	6	10	5	11	8	11	8	17	8	20	7
Tyrannidae	*Pachyramphus polychopter*				2	7	11	6	10	7	11	11	9	11	13	10	14	17	15	20	13
Tyrannidae	*Pachyramphus major*				2	12	8	24	5	16	7	33	4	16	10	28	14	22	15	38	8
Tyrannidae	*Pachyramphus albogriseus*				2	16	7	17	7	18	9	27	7	24	9	25	13	35	14	36	10
Tyrannidae	*Pachyramphus marginatus*				2	5	10	3	11	4	11	7	10	8	12	7	13	13	13	14	12
Tyrannidae	*Pachyramphus surinamus*				2	3	13	2	12	2	14	7	11	6	13	3	12	11	16	12	12
Tyrannidae	*Pachyramphus rufus*				2	7	9	5	9	6	8	11	7	11	10	9	10	19	11	20	9
Tyrannidae	*Pachyramphus spodiurus*	T	H		2	0	8	0	9	0	12	0	0	0	8	0	11	0	14	0	2
Tyrannidae	*Pachyramphus aglaiae*				2	10	7	22	4	13	7	31	4	14	9	23	15	17	12	35	6
Tyrannidae	*Pachyramphus homochrou*				2	13	5	13	8	13	8	17	3	18	6	16	9	21	13	25	4
Tyrannidae	*Pachyramphus minor*				2	5	11	4	12	4	12	8	11	9	13	8	14	14	15	16	14
Tyrannidae	*Pachyramphus niger*			x	2	17	0x	17	5x	11	5x	44	5x	44	0x	17	5x	11	5x	44	5x
Tyrannidae	*Pachyramphus validus*				2	9	17	6	14	9	15	13	11	12	22	11	18	18	19	23	15
Tyrannidae	*Tityra cayana*				2	7	13	5	11	6	12	11	10	11	16	9	14	16	15	19	12
Tyrannidae	*Tityra semifasciata*				2	8	12	7	12	7	13	12	11	13	14	12	15	18	15	22	13
Tyrannidae	*Tityra inquisitor*				2	8	13	7	13	8	13	13	11	13	15	12	17	19	17	23	15
Tyrannidae	*Phoenicircus nigricollis*				2	5	5	3	5	3	5	7	5	9	5	8	5	13	5	16	5
Tyrannidae	*Phoenicircus carnifex*				2	7	12	4	12	5	12	10	11	12	13	8	14	22	15	20	12

Family	Species	IUCN	Ag	R	Range Source	Percentage loss in geographic range 2050 TG H	C	AM H	C	GO H	C	OS H	C	2100 TG H	C	AM H	C	GO H	C	OS H	C
Tyrannidae	*Laniisoma elegans*				2	20	15	16	17	25	10	18	16	22	30	25	28	45	14	29	29
Tyrannidae	*Laniisoma buckleyi*				14	10	8	12	7	17	9	29	6	22	8	23	13	35	9	42	8
Tyrannidae	*Phibalura flavirostris*				2	23	9	20	8	32	6	33	7	33	16	36	15	51	9	52	13
Tyrannidae	*Tijuca atra*			x	2	26	17 x	40	17 x	10	17 x	8	17 x	26	26 x	49	25 x	42	30 x	32	25 x
Tyrannidae	*Tijuca condita*	T		x	14	0	33 x	0	33 x	0	33 x	0	33 x	0	33 x	0	33 x	33	33 x	0	33 x
Tyrannidae	*Carpornis cucullatus*				2	29	10	13	17	33	10	28	14	34	24	33	30	53	16	46	25
Tyrannidae	*Carpornis melanocephalus*	T			2	14	14	10	21	23	14	13	19	24	29	17	37	46	22	32	35
Tyrannidae	*Doliornis sclateri*	T		x	2	30	0 x	20	0 x	20	0 x	13	20 x	30	0 x	20	3 x	33	0 x	13	20 x
Tyrannidae	*Doliornis remseni*	T		x	2	46	54 x	43	57 x	0	-102 x	0	-102 x	46	54 x	43	57 x	0	-102 x	0	-102 x
Tyrannidae	*Ampelion rubrocristatus*				2	12	6	14	4	11	6	18	6	16	7	20	8	25	10	21	8
Tyrannidae	*Ampelion rufaxilla*				2	7	3	5	1	5	2	6	2	8	3	9	2	8	4	8	2
Tyrannidae	*Phytotoma raimondii*	T		x	2	0	11 x	0	14 x	2	16 x	0	14 x	0	24 x	0	35 x	3	35 x	3	33 x
Tyrannidae	*Phytotoma rutila*				2	3	6	2	5	2	5	3	4	3	7	2	8	3	10	4	7
Tyrannidae	*Phytotoma rara*		H		2	0	9	0	7	0	9	0	8	0	14	0	15	0	16	0	15
Tyrannidae	*Zaratornis stresemanni*	T		x	2	0	10 x	24	12 x	9	11 x	26	0 x	0	31 x	34	23 x	19	29 x	29	18 x
Tyrannidae	*Pipreola riefferii*				2	13	5	15	5	13	6	25	4	18	7	22	12	28	12	29	7
Tyrannidae	*Pipreola intermedia*				2	7	11	6	5	6	9	13	14	12	11	10	17	21	12	18	17
Tyrannidae	*Pipreola arcuata*				2	13	6	17	4	14	6	23	8	22	8	23	10	31	12	27	10
Tyrannidae	*Pipreola aureopectus*			x	2	22	0 x	20	2 x	25	2 x	32	2 x	38	3 x	31	10 x	41	8 x	42	2 x
Tyrannidae	*Pipreola jucunda*			x	2	19	2 x	12	6 x	25	6 x	14	0 x	20	2 x	12	6 x	28	6 x	28	0 x
Tyrannidae	*Pipreola lubomirskii*			x	2	15	13 x	23	9 x	16	14 x	33	14 x	22	17 x	40	19 x	52	16 x	42	22 x
Tyrannidae	*Pipreola pulchra*				2	12	0	6	0	10	0	16	6	19	0	10	11	23	6	16	6
Tyrannidae	*Pipreola chlorolepidota*				2	8	7	6	7	13	7	24	4	17	7	23	10	32	8	37	5
Tyrannidae	*Pipreola frontalis*				2	11	7	5	3	8	6	22	7	20	7	13	14	28	8	29	11
Tyrannidae	*Pipreola formosa*			x	2	18	0 x	3	0 x	18	0 x	9	0 x	18	0 x	21	0 x	22	0 x	11	0 x
Tyrannidae	*Pipreola whitelyi*			x	2	14	3 x	0	17 x	14	4 x	0	17 x	14	7 x	6	26 x	16	16 x	11	32 x
Tyrannidae	*Ampelioides tschudii*				2	16	9	12	7	12	9	29	6	23	9	20	12	29	11	37	6
Tyrannidae	*Iodopleura pipra*		H		2	0	27	0	28	0	22	0	27	0	40	0	37	0	27	0	38
Tyrannidae	*Iodopleura isabellae*				2	5	6	3	6	4	6	8	6	9	7	8	8	14	8	17	7
Tyrannidae	*Iodopleura fusca*				2	3	19	2	19	4	18	5	19	5	20	6	24	10	24	9	24
Tyrannidae	*Calyptura cristata*	T		x	2	0	30 x	0	30 x	0	30 x	0	30 x	0	30 x	3	30 x	24	30 x	3	30 x
Tyrannidae	*Lipaugus subalaris*			x	2	14	12 x	10	12 x	10	12 x	25	12 x	21	12 x	31	18 x	48	12 x	47	14 x
Tyrannidae	*Lipaugus cryptolophus*				2	2	8	6	7	13	8	21	3	15	8	28	12	33	10	33	5
Tyrannidae	*Lipaugus fuscocinereus*			x	2	20	13 x	27	7 x	16	14 x	31	14 x	25	17 x	48	16 x	52	18 x	40	22 x
Tyrannidae	*Lipaugus uropygialis*	T		x	2	13	0 x	13	0 x	13	0 x	13	0 x	13	0 x	13	0 x	13	0 x	13	0 x
Tyrannidae	*Lipaugus vociferans*				2	5	12	4	12	4	13	8	12	9	14	8	15	14	15	16	14
Tyrannidae	*Lipaugus unirufus*				2	19	5	27	6	20	6	33	5	25	6	33	10	29	9	41	6
Tyrannidae	*Lipaugus lanioides*				2	26	15	11	24	23	16	23	22	27	34	21	40	48	21	34	35
Tyrannidae	*Lipaugus streptophorus*				2	11	4	0	15	11	8	0	15	11	15	11	24	17	19	7	35
Tyrannidae	*Porphyrolaema porphyrola*				2	3	6	3	5	3	5	6	5	6	7	7	7	8	7	13	6
Tyrannidae	*Cotinga amabilis*				2	13	4	21	4	15	4	29	2	15	5	28	9	24	6	34	4
Tyrannidae	*Cotinga ridgwayi*	T		x	2	43	9 x	30	29 x	25	29 x	37	29 x	54	9 x	30	48 x	28	38 x	58	29 x
Tyrannidae	*Cotinga nattererii*				2	12	6	17	9	13	6	17	6	19	9	18	9	23	11	29	9
Tyrannidae	*Cotinga maynana*				2	4	6	3	5	3	6	6	5	8	6	8	7	9	7	14	6

Family	Species	IUCN	Ag	R	Range Source	2050 TG H	2050 TG C	2050 AM H	2050 AM C	2050 GO H	2050 GO C	2050 OS H	2050 OS C	2100 TG H	2100 TG C	2100 AM H	2100 AM C	2100 GO H	2100 GO C	2100 OS H	2100 OS C
Tyrannidae	*Cotinga cotinga*				2	5	10	3	9	5	10	9	9	10	10	7	11	20	12	19	10
Tyrannidae	*Cotinga maculata*	T		x	2	0	7x	6	9x	14	4x	5	7x	16	9x	10	22x	33	19x	26	20x
Tyrannidae	*Cotinga cayana*				2	5	9	4	9	4	10	9	9	9	11	8	11	15	12	17	11
Tyrannidae	*Xipholena punicea*				2	2	15	2	16	2	16	4	15	5	17	3	18	7	19	7	18
Tyrannidae	*Xipholena lamellipennis*				2	11	7	7	7	9	7	20	5	20	7	15	9	42	9	42	7
Tyrannidae	*Xipholena atropurpurea*	T			2	2	32	5	32	10	28	6	30	8	34	8	41	23	38	15	40
Tyrannidae	*Carpodectes nitidus*				2	21	3	33	5	33	5	46	3	29	3	41	8	45	5	52	3
Tyrannidae	*Carpodectes antoniae*	T		x	2	36	0x	43	7x	34	7x	55	7x	57	0x	43	39x	39	23x	72	7x
Tyrannidae	*Carpodectes hopkei*				2	12	5	14	7	13	3	9	5	23	7	15	7	18	9	26	7
Tyrannidae	*Conioptilon mcilhennyi*				2	6	3	2	3	6	3	11	3	13	4	19	4	14	4	21	4
Tyrannidae	*Gymnoderus foetidus*				2	6	13	4	13	5	13	9	13	11	14	9	16	17	16	19	15
Tyrannidae	*Haematoderus militaris*				2	3	15	2	13	3	14	7	13	6	15	4	16	13	18	13	15
Tyrannidae	*Pyroderus scutatus*				2	16	7	15	7	23	5	25	5	27	11	25	11	38	9	38	8
Tyrannidae	*Cephalopterus glabricollis*	T		x	2	15	5x	33	8x	23	8x	40	8x	19	5x	33	8x	51	8x	53	8x
Tyrannidae	*Cephalopterus penduliger*	T			2	14	8	9	3	17	3	12	0	23	8	9	3	18	3	20	0
Tyrannidae	*Cephalopterus ornatus*				2	5	11	4	11	3	12	7	11	8	13	7	13	12	14	14	13
Tyrannidae	*Perissocephalus tricolor*				2	3	13	3	12	3	13	5	12	6	14	5	15	11	17	10	16
Tyrannidae	*Procnias tricarunculata*	T		x	2	18	7x	25	16x	12	16x	28	16x	28	7x	25	22x	28	22x	41	16x
Tyrannidae	*Procnias alba*				2	5	19	3	20	6	19	8	19	9	20	7	24	15	24	13	23
Tyrannidae	*Procnias averano*				2	7	11	5	12	5	13	8	11	11	14	7	16	12	20	18	19
Tyrannidae	*Procnias nudicollis*	T			2	22	9	18	9	29	7	30	7	33	15	31	15	47	11	48	13
Tyrannidae	*Rupicola rupicola*				2	3	11	2	11	2	12	4	11	5	13	3	15	8	16	8	15
Tyrannidae	*Rupicola peruviana*				2	14	7	12	5	11	6	22	6	19	8	20	12	28	11	29	8
Tyrannidae	*Oxyruncus cristatus*				2	8	16	6	17	9	16	13	16	14	19	10	21	22	21	24	21
Tyrannidae	*Pipra aureola*				2	7	11	5	11	7	10	9	9	11	11	11	13	19	13	19	11
Tyrannidae	*Pipra fasciicauda*				2	7	17	6	17	6	17	13	16	14	19	12	20	21	20	24	19
Tyrannidae	*Pipra filicauda*				2	3	3	2	2	2	2	4	2	5	3	5	3	6	4	9	3
Tyrannidae	*Pipra mentalis*				2	15	4	24	5	19	5	31	4	21	5	29	11	26	10	39	6
Tyrannidae	*Pipra erythrocephala*				2	4	8	3	8	3	8	7	8	7	9	7	10	11	11	13	10
Tyrannidae	*Pipra rubrocapilla*				2	5	14	3	15	3	15	8	14	9	16	6	17	16	17	17	16
Tyrannidae	*Pipra chloromeros*				2	16	5	14	5	16	5	24	4	24	6	27	7	30	6	36	5
Tyrannidae	*Pipra cornuta*				2	7	14	4	17	3	16	4	17	10	18	7	21	10	22	9	25
Tyrannidae	*Pipra pipra*				2	4	8	3	8	4	8	8	7	8	8	7	9	14	10	16	9
Tyrannidae	*Lepidothrix coronata*				2	5	5	4	5	4	5	7	5	9	6	8	7	10	7	13	7
Tyrannidae	*Lepidothrix serena*				2	2	13	1	12	2	13	6	12	4	13	2	13	11	16	11	13
Tyrannidae	*Lepidothrix suavissima*				2	6	13	6	14	4	15	4	16	9	17	9	19	9	21	9	23
Tyrannidae	*Lepidothrix iris*				2	11	18	5	20	6	20	14	18	18	20	12	23	27	24	32	21
Tyrannidae	*Lepidothrix vilasboasi*	T		x	14	0	0x	0	0x	0	0x	51	0x	0	0x	0	0x	51	0x	62	0x
Tyrannidae	*Lepidothrix nattereri*				2	3	24	3	26	2	28	5	25	5	28	6	27	8	28	9	27
Tyrannidae	*Lepidothrix isidorei*				2	6	9	10	9	8	9	25	5	10	10	15	16	26	15	32	7
Tyrannidae	*Lepidothrix coeruleocapilla*				2	28	14	13	8	19	14	47	6	40	14	27	18	45	14	59	7
Tyrannidae	*Antilophia galeata*				2	8	18	3	15	4	19	13	13	13	25	8	18	11	22	21	15
Tyrannidae	*Chiroxiphia linearis*				2	4	0	6	0	10	0	15	0	10	0	6	7	13	7	18	0
Tyrannidae	*Chiroxiphia lanceolata*				2	20	6	20	8	18	9	23	6	24	11	27	12	30	20	31	12

Family	Species	IUCN	Ag	R	Range Source	2050 TG H	2050 TG C	2050 AM H	2050 AM C	2050 GO H	2050 GO C	2050 OS H	2050 OS C	2100 TG H	2100 TG C	2100 AM H	2100 AM C	2100 GO H	2100 GO C	2100 OS H	2100 OS C
Tyrannidae	*Chiroxiphia pareola*				2	4	11	3	11	4	11	8	10	8	12	7	13	15	14	17	12
Tyrannidae	*Chiroxiphia boliviana*				2	22	11	10	7	12	9	27	4	22	11	18	8	23	10	35	5
Tyrannidae	*Chiroxiphia caudata*				2	22	8	18	8	28	6	29	7	32	15	31	14	45	9	46	13
Tyrannidae	*Masius chrysopterus*				2	10	6	13	7	11	7	23	6	16	10	19	15	28	13	31	9
Tyrannidae	*Ilicura militaris*				2	20	8	13	11	26	7	21	9	25	19	20	22	40	12	37	18
Tyrannidae	*Corapipo gutturalis*				2	3	15	3	15	3	15	6	15	7	17	5	18	12	19	11	19
Tyrannidae	*Corapipo leucorrhoa*				2	19	4	28	8	21	8	37	5	26	6	34	12	33	12	47	7
Tyrannidae	*Manacus candei*				2	17	4	27	5	21	5	37	3	22	5	34	10	31	7	44	4
Tyrannidae	*Manacus aurantiacus*			x	2	32	6x	41	16x	17	22x	45	16x	42	6x	42	29x	19	30x	63	16x
Tyrannidae	*Manacus vitellinus*				2	12	6	22	11	10	7	17	9	22	10	23	11	22	14	32	12
Tyrannidae	*Manacus manacus*				2	5	12	4	13	5	13	9	12	9	14	8	16	16	16	17	15
Tyrannidae	*Machaeropterus pyrocepha*				2	8	20	6	21	6	22	10	21	12	23	11	23	16	23	17	22
Tyrannidae	*Machaeropterus regulus*				2	4	4	3	4	3	4	5	4	9	5	8	6	9	6	14	7
Tyrannidae	*Machaeropterus deliciosus*			x	2	18	1x	10	1x	22	1x	11	0x	23	1x	10	1x	23	1x	23	0x
Tyrannidae	*Xenopipo atronitens*				2	7	12	5	12	5	13	10	12	13	14	10	15	21	16	20	15
Tyrannidae	*Chloropipo unicolor*				2	14	12	9	7	12	12	28	12	25	12	18	23	38	16	36	16
Tyrannidae	*Chloropipo uniformis*				2	12	1	0	12	12	4	0	12	12	12	16	21	21	16	10	35
Tyrannidae	*Chloropipo holochlora*				2	15	4	18	4	20	3	29	3	25	5	29	6	33	5	45	4
Tyrannidae	*Chloropipo flavicapilla*			x	2	1	0x	19	0x	15	0x	21	0x	17	0x	35	1x	35	0x	21	1x
Tyrannidae	*Heterocercus flavivertex*				2	1	8	1	7	2	8	3	6	3	9	2	9	4	10	5	8
Tyrannidae	*Heterocercus aurantiivertex*				2	5	2	0	0	0	0	6	0	7	2	10	0	5	0	18	0
Tyrannidae	*Heterocercus linteatus*				2	5	15	3	16	3	17	6	16	8	18	6	17	14	18	13	17
Tyrannidae	*Neopelma chrysocephalum*				2	4	13	4	11	3	13	6	12	7	15	7	16	9	18	9	17
Tyrannidae	*Neopelma sulphureiventer*				2	8	11	5	12	7	11	9	11	13	12	11	17	15	16	18	16
Tyrannidae	*Neopelma pallescens*				2	9	20	4	20	4	23	14	17	12	27	8	24	15	28	23	21
Tyrannidae	*Neopelma aurifrons*	T			2	13	6	12	6	25	4	15	6	30	7	17	17	40	13	35	17
Tyrannidae	*Neopelma chrysolophum*				2	12	16	12	16	21	11	18	14	15	27	16	28	35	16	27	21
Tyrannidae	*Tyranneutes stolzmanni*				2	5	9	3	10	4	10	8	9	9	11	8	11	14	12	16	11
Tyrannidae	*Tyranneutes virescens*				2	4	14	2	13	4	14	7	13	7	15	5	16	14	17	13	15
Tyrannidae	*Piprites pileatus*	T			2	25	6	20	8	31	5	33	7	33	12	37	14	50	9	57	11
Tyrannidae	*Piprites griseiceps*				2	21	3	35	4	34	5	45	3	29	3	43	7	45	5	50	4
Tyrannidae	*Piprites chloris*				2	5	10	4	10	5	11	8	10	9	12	8	12	14	13	16	12
Thamnophilidae	*Cymbilaimus lineatus*				2	5	10	4	10	4	11	8	10	9	12	8	12	14	13	16	12
Thamnophilidae	*Cymbilaimus sanctaemaria*				2	4	8	3	6	3	7	8	7	10	10	8	11	12	10	15	9
Thamnophilidae	*Hypoedaleus guttatus*				2	12	10	12	7	16	6	19	6	19	17	17	13	30	9	29	9
Thamnophilidae	*Batara cinerea*				2	18	6	16	7	25	4	26	6	24	16	29	16	45	9	47	11
Thamnophilidae	*Mackenziaena severa*				2	14	8	14	7	20	5	23	6	21	16	21	15	35	9	36	11
Thamnophilidae	*Mackenziaena leachii*				2	19	7	18	7	26	4	28	5	27	16	29	16	44	9	46	11
Thamnophilidae	*Frederickena viridis*				2	4	16	3	15	4	16	7	15	7	17	6	19	13	20	12	19
Thamnophilidae	*Frederickena unduligera*				2	3	2	2	2	3	2	5	2	6	2	7	2	6	2	12	2
Thamnophilidae	*Taraba major*				2	7	12	5	11	6	12	11	9	10	15	9	14	15	15	18	12
Thamnophilidae	*Sakesphorus canadensis*		H		2	0	11	0	12	0	11	0	10	0	13	0	16	0	17	0	15
Thamnophilidae	*Sakesphorus cristatus*				2	12	18	9	14	11	17	18	9	13	24	14	17	16	20	24	11
Thamnophilidae	*Sakesphorus bernardi*				2	2	11	0	14	0	15	1	14	2	14	0	21	1	23	1	22

Percentage loss in geographic range

Family	Species	IUCN	Ag	R	Range Source	Percentage loss in geographic range															
						2050								2100							
						TG		AM		GO		OS		TG		AM		GO		OS	
						H	C	H	C	H	C	H	C	H	C	H	C	H	C	H	C
Thamnophilidae	*Sakesphorus melanonotus*				2	26	7	14	19	17	12	20	7	26	22	18	27	19	38	25	26
Thamnophilidae	*Sakesphorus melanothorax*				2	0	18	0	18	0	18	0	18	0	18	0	19	1	23	0	19
Thamnophilidae	*Sakesphorus luctuosus*				2	6	37	3	23	3	29	10	23	11	44	7	26	16	32	17	24
Thamnophilidae	*Biatas nigropectus*	T			2	21	12	19	15	28	9	32	12	27	25	36	24	57	12	56	22
Thamnophilidae	*Thamnophilus doliatus*				2	9	14	7	10	8	12	13	8	11	18	11	14	15	17	20	11
Thamnophilidae	*Thamnophilus zarumae*			x	2	4	9x	3	5x	0	13x	0	8x	4	9x	3	11x	0	19x	0	14x
Thamnophilidae	*Thamnophilus multistriatus*				2	16	4	22	4	9	4	24	4	25	4	25	4	17	5	25	5
Thamnophilidae	*Thamnophilus tenuepuncta*				2	8	7	9	6	15	7	29	4	12	7	23	7	35	8	40	5
Thamnophilidae	*Thamnophilus bridgesi*			x	2	29	6x	30	16x	16	21x	42	16x	42	6x	31	32x	20	32x	56	16x
Thamnophilidae	*Thamnophilus nigriceps*				2	16	4	17	9	11	9	24	4	17	8	21	10	19	16	26	8
Thamnophilidae	*Thamnophilus praecox*			x	2	63	0x	0	0x	4	0x	36	0x	73	0x	23	0x	57	0x	65	0x
Thamnophilidae	*Thamnophilus nigrocinereu*				2	6	9	5	9	5	9	10	8	11	10	9	10	20	11	21	10
Thamnophilidae	*Thamnophilus cryptoleucus*			x	2	7	8x	7	0x	0	8x	9	0x	8	8x	15	0x	8	8x	18	0x
Thamnophilidae	*Thamnophilus aethiops*				2	5	10	4	11	4	11	8	11	9	12	7	13	14	13	15	13
Thamnophilidae	*Thamnophilus unicolor*				2	12	6	10	5	8	6	19	6	17	6	19	11	24	10	27	7
Thamnophilidae	*Thamnophilus schistaceus*				2	6	10	4	11	5	11	9	10	11	12	9	13	15	13	18	12
Thamnophilidae	*Thamnophilus murinus*				2	3	8	3	8	3	8	5	8	7	9	6	10	9	11	12	10
Thamnophilidae	*Thamnophilus aroyae*				2	28	2	3	2	12	2	20	1	34	2	19	8	24	8	25	7
Thamnophilidae	*Thamnophilus punctatus*				2	5	15	4	15	5	15	9	14	9	17	7	19	16	20	16	18
Thamnophilidae	*Thamnophilus ambiguus*				14	5	9	6	9	10	6	12	8	9	11	8	16	25	11	17	14
Thamnophilidae	*Thamnophilus leucogaster*			x	14	0	23x	1	22x	0	23x	23	0x	0	23x	1	22x	0	23x	23	0x
Thamnophilidae	*Thamnophilus pelzelni*				14	7	19	4	11	5	15	11	7	8	24	8	13	10	18	17	9
Thamnophilidae	*Thamnophilus stictocephal*				14	9	14	10	15	9	17	18	14	19	18	19	17	30	18	34	15
Thamnophilidae	*Thamnophilus sticturus*				14	22	17	6	21	8	20	14	18	25	20	11	30	28	30	32	25
Thamnophilidae	*Thamnophilus atrinucha*				2	14	4	16	5	15	5	21	3	19	4	20	7	23	9	26	4
Thamnophilidae	*Thamnophilus insignis*				2	7	16	1	23	5	18	1	23	8	24	5	38	8	35	4	42
Thamnophilidae	*Thamnophilus amazonicus*				2	4	11	3	11	3	12	7	11	8	13	6	13	13	14	15	13
Thamnophilidae	*Thamnophilus caerulescen*				2	8	10	6	6	9	7	12	5	11	14	11	10	17	11	18	8
Thamnophilidae	*Thamnophilus torquatus*				2	8	20	5	15	6	18	13	11	11	26	9	17	13	21	20	13
Thamnophilidae	*Thamnophilus ruficapillus*				2	13	6	12	4	16	3	19	4	18	8	20	7	27	5	29	6
Thamnophilidae	*Pygiptila stellaris*				2	4	8	3	9	3	9	7	8	8	10	7	11	12	11	14	10
Thamnophilidae	*Megastictus margaritatus*				2	3	6	2	5	2	6	4	6	7	6	6	6	7	7	12	7
Thamnophilidae	*Neoctantes niger*				2	4	1	1	1	2	0	5	0	7	1	6	1	9	0	14	0
Thamnophilidae	*Clytoctantes alixii*	T		x	2	18	2x	13	2x	14	4x	20	0x	21	2x	20	4x	18	4x	20	2x
Thamnophilidae	*Clytoctantes atrogularis*	T		x	14	0	0x	0	0x	0	0x	0	0x	0	0x	0	0x	0	0x	0	0x
Thamnophilidae	*Xenornis setifrons*	T		x	2	18	2x	32	19x	19	19x	47	2x	18	5x	36	19x	73	22x	51	5x
Thamnophilidae	*Thamnistes anabatinus*				2	20	5	25	6	21	6	36	4	28	6	33	11	34	9	44	6
Thamnophilidae	*Dysithamnus stictothorax*				2	20	11	18	13	28	8	30	11	28	20	33	21	53	11	52	19
Thamnophilidae	*Dysithamnus mentalis*				2	12	16	10	17	12	18	17	17	18	21	16	23	24	23	27	22
Thamnophilidae	*Dysithamnus striaticeps*				2	23	3	34	5	35	6	50	3	32	3	45	9	48	6	57	3
Thamnophilidae	*Dysithamnus puncticeps*				2	16	5	20	8	16	6	16	5	25	8	22	8	24	11	31	8
Thamnophilidae	*Dysithamnus xanthopterus*			x	2	32	26x	19	33x	32	18x	16	33x	32	37x	31	43x	51	22x	24	46x
Thamnophilidae	*Dysithamnus leucostictus*				2	18	11x	21	10x	20	12x	33	12x	24	15x	34	21x	53	13x	45	19x
Thamnophilidae	*Dysithamnus tucuyensis*			x	14	27	0x	3	0x	25	0x	13	0x	27	2x	27	2x	25	4x	14	4x

Family	Species	IUCN	Ag	R	Range Source	2050 TG H	2050 TG C	2050 AM H	2050 AM C	2050 GO H	2050 GO C	2050 OS H	2050 OS C	2100 TG H	2100 TG C	2100 AM H	2100 AM C	2100 GO H	2100 GO C	2100 OS H	2100 OS C
Thamnophilidae	*Dysithamnus plumbeus*	T			2	7	3	8	3	24	1	8	3	25	4	11	14	39	8x	37	13x
Thamnophilidae	*Dysithamnus occidentalis*	T		x	2	0	0x	0	0x	0	0x	0	0x	4	0x	0	0x	0	0x	0	0x
Thamnophilidae	*Thamnomanes saturninus*				2	2	12	2	12	1	12	3	12	5	13	3	13	6	13	8	13
Thamnophilidae	*Thamnomanes ardesiacus*				2	4	8	3	7	4	7	7	7	8	9	8	9	12	10	14	9
Thamnophilidae	*Thamnomanes caesius*				2	5	12	3	12	3	12	8	11	9	13	7	14	15	15	16	13
Thamnophilidae	*Thamnomanes schistogynu*				2	6	4	4	4	5	4	8	4	9	5	9	7	10	6	15	5
Thamnophilidae	*Myrmotherula brachyura*				2	5	9	4	9	4	10	8	9	9	11	8	11	14	12	16	11
Thamnophilidae	*Myrmotherula ignota*				14	10	5	16	8	12	5	14	7	18	8	17	8	22	11	25	10
Thamnophilidae	*Myrmotherula obscura*				2	4	0	3	0	4	0	7	0	8	0	9	0	11	0	18	0
Thamnophilidae	*Myrmotherula sclateri*				2	3	12	2	12	2	13	4	12	6	14	5	14	8	15	9	13
Thamnophilidae	*Myrmotherula klagesi*				14	22	19	17	2	13	18	22	2	43	19x	35	2	41	18x	46	2x
Thamnophilidae	*Myrmotherula ambigua*				2	0	0	0	0	0	0	0	0	0	0	0	0	0	0	0	0
Thamnophilidae	*Myrmotherula surinamensi*				2	7	13	4	14	10	10	7	11	9	16	11	21	25	18	18	17
Thamnophilidae	*Myrmotherula multostriata*				14	8	20	5	19	6	22	15	17	13	25	11	23	21	26	26	20
Thamnophilidae	*Myrmotherula pacifica*				14	16	6	12	7	9	9	22	2	20	7	18	9	17	11	25	5
Thamnophilidae	*Myrmotherula cherriei*				2	1	7	2	7	2	6	4	6	2	9	3	11	5	10	6	10
Thamnophilidae	*Myrmotherula longicauda*				2	28	7	17	6	19	6	39	3	38	7	31	9	43	7	47	6
Thamnophilidae	*Myrmotherula hauxwelli*				2	5	9	4	10	4	10	9	9	10	11	9	11	16	12	19	11
Thamnophilidae	*Myrmotherula guttata*				2	3	13	3	13	3	13	5	12	6	15	5	16	11	18	10	16
Thamnophilidae	*Myrmotherula gularis*				2	22	10	17	14	27	9	26	12	28	25	30	25	48	14	47	23
Thamnophilidae	*Myrmotherula gutturalis*				2	3	13	3	12	3	13	7	12	6	14	5	14	14	16	13	14
Thamnophilidae	*Myrmotherula fulviventris*				2	17	4	26	7	22	5	31	4	26	5	31	8	31	8	40	6
Thamnophilidae	*Myrmotherula leucophthal*				2	5	13	3	13	4	13	9	12	10	14	7	15	17	15	18	14
Thamnophilidae	*Myrmotherula fjeldsaai*				14	4	1	0	0	0	0	5	0	4	1	4	0	2	0	10	0
Thamnophilidae	*Myrmotherula haematonota*				2	2	7	2	7	1	7	3	7	5	8	3	9	5	10	7	9
Thamnophilidae	*Myrmotherula spodionota*				2	18	6	13	4	15	6	29	5	27	7	25	15	43	10	39	8
Thamnophilidae	*Myrmotherula ornata*				2	9	10	7	9	8	10	17	9	16	11	15	11	27	11	29	10
Thamnophilidae	*Myrmotherula erythrura*				2	5	1	4	1	4	1	8	0	10	1	10	1	12	1	18	1
Thamnophilidae	*Myrmotherula axillaris*				2	6	11	4	11	5	12	9	11	10	13	8	14	15	14	17	13
Thamnophilidae	*Myrmotherula luctuosa*				14	6	8	8	8	20	6	9	7	21	10	12	20	35	15	31	19
Thamnophilidae	*Myrmotherula fluminensis*	T		x	14	0	0x	0	0x	0	0x	0	0x	0	0x	0	0x	0	0x	0	0x
Thamnophilidae	*Myrmotherula schisticolor*				2	17	6	18	5	17	7	27	6	23	7	25	10	30	10	35	7
Thamnophilidae	*Myrmotherula sunensis*				2	10	1	5	1	4	0	12	1	14	1	14	1	17	0	21	1
Thamnophilidae	*Myrmotherula longipennis*				2	4	8	3	8	3	8	7	7	8	9	6	10	13	10	14	9
Thamnophilidae	*Myrmotherula minor*	T			2	14	18	10	24	15	18	12	21	14	45x	17	44x	52	24x	30	43x
Thamnophilidae	*Myrmotherula iheringi*				2	3	14	2	14	2	13	4	13	6	15	5	16	7	17	8	15
Thamnophilidae	*Myrmotherula grisea*	T			2	14	0	9	0	14	0	17	0	14	0	22	0	22	0	22	0
Thamnophilidae	*Myrmotherula unicolor*				2	27	11	15	22	30	14	23	16	29	38	31	41	51	23	37	34
Thamnophilidae	*Myrmotherula snowi*	T		x	14	0	⊦02x	0	⊦02x	0	⊦02x	0	⊦02x	0	⊦02x	0	⊦02x	0	⊦02x	0	⊦02x
Thamnophilidae	*Myrmotherula behni*				2	4	13	5	14	2	15	1	17	8	16	7	19	4	22	2	25
Thamnophilidae	*Myrmotherula urosticta*	T			2	3	6	7	7	23	4	4	6	25	7	10	22	37	17x	32	21x
Thamnophilidae	*Myrmotherula menetriesii*				2	5	9	4	9	4	9	8	9	9	10	8	11	15	12	16	11
Thamnophilidae	*Myrmotherula assimilis*				2	5	11	4	10	2	11	6	9	9	11	7	10	7	11	12	9
Thamnophilidae	*Dichrozona cincta*				2	5	6	4	5	4	5	9	5	10	6	9	6	15	7	18	6

Family	Species	IUCN	Ag	R	Range Source	Percentage loss in geographic range															
						2050								2100							
						TG		AM		GO		OS		TG		AM		GO		OS	
						H	C	H	C	H	C	H	C	H	C	H	C	H	C	H	C
Thamnophilidae	*Myrmorchilus strigilatus*				2	6	16	5	14	6	15	10	11	8	20	9	23	14	25	16	20
Thamnophilidae	*Herpsilochmus parkeri*	T		x	14	0	0x	28	0x	0	0x	28	0x	0	0x	28	0x	0	0x	28	0x
Thamnophilidae	*Herpsilochmus motacilloide*			x	2	31	0x	16	0x	30	0x	43	5x	49	0x	33	8x	50	0x	46	5x
Thamnophilidae	*Herpsilochmus atricapillus*				2	9	17	6	12	7	14	14	9	12	21	10	16	16	19	23	13
Thamnophilidae	*Herpsilochmus pileatus*	T		x	2	1	0x	21	1x	8	0x	0	0x	11	0x	21	5x	33	4x	33	4x
Thamnophilidae	*Herpsilochmus sellowi*				14	13	24	10	21	10	22	20	12	14	30	15	24	17	25	27	15
Thamnophilidae	*Herpsilochmus sticturus*				2	3	19	3	19	6	17	9	18	8	19	7	24	16	24	15	21
Thamnophilidae	*Herpsilochmus dugandi*				2	10	1	2	1	5	0	16	1	14	1	15	2	17	0	27	2
Thamnophilidae	*Herpsilochmus stictocephal*				2	1	17	2	17	4	16	6	16	5	17	4	20	10	20	9	18
Thamnophilidae	*Herpsilochmus gentryi*				14	0	5	0	0	0	0	0	0	0	5	13	0	0	0	10	0
Thamnophilidae	*Herpsilochmus dorsimacul*				2	2	12	3	11	1	13	2	11	5	14	3	14	3	17	5	15
Thamnophilidae	*Herpsilochmus roraimae*				2	6	15	6	16	1	18	4	18	11	20	8	21	8	25	9	27
Thamnophilidae	*Herpsilochmus pectoralis*	T			2	7	30	7	30	7	30	33	13	8	35	13	41	23	40	39	19
Thamnophilidae	*Herpsilochmus longirostris*				2	7	23	3	20	4	24	9	19	10	30	6	24	11	26	16	21
Thamnophilidae	*Herpsilochmus axillaris*				2	12	12	16	9	16	11	28	8	23	12	23	15	34	12	36	10
Thamnophilidae	*Herpsilochmus rufimarginat*				2	11	16	8	17	11	17	17	16	17	19	15	21	25	20	28	19
Thamnophilidae	*Microrhopias quixensis*				2	6	10	5	10	5	11	11	9	10	11	10	12	15	12	19	11
Thamnophilidae	*Formicivora iheringi*				2	26	17	16	21	25	21	34	13	30	17	24	25	34	25	40	15
Thamnophilidae	*Formicivora intermedia*				14	9	9	7	10	8	9	12	9	12	11	11	14	17	15	17	13
Thamnophilidae	*Formicivora melanogaster*				2	7	20	6	16	7	19	12	12	10	25	11	20	15	23	22	16
Thamnophilidae	*Formicivora serrana*				2	12	5	10	2	12	2	17	1	17	7	10	3	20	3	23	1
Thamnophilidae	*Formicivora littoralis*	T		x	2	0	0x	0	0x	0	0x	10	0x	0	0x	0	0x	26	0x	10	0x
Thamnophilidae	*Formicivora erythronotos*	T		x	2	0	1x	0	1x	0	1x	9	1x	0	10x	26	9x	37	1x	34	1x
Thamnophilidae	*Formicivora rufa*				2	7	16	3	8	5	12	9	6	9	21	7	10	10	14	15	8
Thamnophilidae	*Stymphalornis acutirostris*	T		x	14	93	0x	0	67x	0	67x	0	67x	93	0x	0	93x	0	93x	26	67x
Thamnophilidae	*Drymophila ferruginea*				2	11	9	9	9	18	6	15	8	16	15	14	18	29	11	22	15
Thamnophilidae	*Drymophila rubricollis*				2	20	8	22	8	26	5	32	7	29	23	32	21	52	12	57	16
Thamnophilidae	*Drymophila genei*			x	2	11	3x	27	3x	3	3x	21	3x	12	15x	40	13x	32	11x	42	13x
Thamnophilidae	*Drymophila ochropyga*				2	27	16	14	27	27	16	21	23	28	38	26	43	49	23	34	40
Thamnophilidae	*Drymophila devillei*				2	5	13	4	14	4	15	8	14	9	17	8	17	12	17	14	16
Thamnophilidae	*Drymophila caudata*				2	16	3	16	3	14	3	21	3	25	5	25	10	32	8	30	7
Thamnophilidae	*Drymophila malura*				2	23	9	20	9	32	6	33	7	31	16	35	15	49	9	51	13
Thamnophilidae	*Drymophila squamata*				2	16	8	11	13	23	9	14	11	27	20	18	28	42	18	34	26
Thamnophilidae	*Terenura maculata*				2	19	11	18	11	30	8	28	9	31	21	30	22	48	15	48	20
Thamnophilidae	*Terenura sicki*	T		x	2	0	69x	0	69x	0	69x	0	69x	0	69x	0	69x	0	69x	0	69x
Thamnophilidae	*Terenura callinota*				2	14	9	15	11	15	11	31	7	20	10	25	17	39	12	43	11
Thamnophilidae	*Terenura humeralis*				2	2	6	1	5	1	5	4	5	5	7	6	8	5	8	12	7
Thamnophilidae	*Terenura sharpei*	T		x	2	18	0x	10	0x	13	0x	26	0x	26	0x	22	9x	26	9x	26	9x
Thamnophilidae	*Terenura spodioptila*				2	4	12	3	12	3	12	6	11	8	13	5	15	12	16	11	15
Thamnophilidae	*Cercomacra cinerascens*				2	5	11	3	11	4	11	8	10	9	12	7	13	14	13	16	12
Thamnophilidae	*Cercomacra brasiliana*			x	2	10	7x	7	7x	11	6x	19	7x	14	18x	16	24x	36	11x	38	16x
Thamnophilidae	*Cercomacra tyrannina*				2	6	10	6	10	6	10	10	9	9	11	8	13	13	14	15	12
Thamnophilidae	*Cercomacra laeta*				14	1	21	1	19	1	22	2	19	2	23	1	25	10	30	9	25
Thamnophilidae	*Cercomacra parkeri*			x	14	9	7x	6	5x	4	7x	8	5x	13	7x	10	7x	4	7x	8	7x

Family	Species	IUCN	Ag	R	Range Source	2050 TG H	2050 TG C	2050 AM H	2050 AM C	2050 GO H	2050 GO C	2050 OS H	2050 OS C	2100 TG H	2100 TG C	2100 AM H	2100 AM C	2100 GO H	2100 GO C	2100 OS H	2100 OS C
Thamnophilidae	*Cercomacra nigrescens*				2	6	13	5	13	4	14	10	12	11	14	10	15	16	15	19	13
Thamnophilidae	*Cercomacra ferdinandi*	T			2	24	28x	2	33x	4	41x	14	31x	29	47x	7	38x	31	48x	19	35x
Thamnophilidae	*Cercomacra serva*				2	7	2	5	2	7	1	13	1	13	2	15	2	16	2	25	2
Thamnophilidae	*Cercomacra nigricans*				2	18	6	18	9	17	7	26	6	24	9	25	12	28	15	38	8
Thamnophilidae	*Cercomacra carbonaria*	T		x	2	0	30x	0	30x	0	30x	0	32x	0	30x	0	30x	0	39x	0	41x
Thamnophilidae	*Cercomacra manu*				2	4	5	0	5	3	5	8	4	6	7	11	7	7	7	14	6
Thamnophilidae	*Cercomacra melanaria*				2	18	15	11	15	8	16	17	14	23	15	19	19	25	19	31	17
Thamnophilidae	*Pyriglena leuconota*				2	13	11	9	12	10	12	22	11	20	14	17	19	39	18	40	16
Thamnophilidae	*Pyriglena leucoptera*				2	15	8	14	7	21	5	22	6	22	14	21	14	36	9	35	11
Thamnophilidae	*Pyriglena atra*	T		x	2	33	17x	33	17x	33	17x	33	17x	33	17x	33	17x	33	17x	33	17x
Thamnophilidae	*Rhopornis ardesiaca*	T		x	2	23	11x	8	11x	11	11x	23	11x	25	11x	15	11x	18	11x	25	11x
Thamnophilidae	*Myrmoborus leucophrys*				2	5	13	4	14	5	14	9	13	10	15	8	16	15	16	17	15
Thamnophilidae	*Myrmoborus lugubris*				2	8	7	6	4	5	7	8	2	11	7	13	4	16	7	21	3
Thamnophilidae	*Myrmoborus myotherinus*				2	6	9	4	9	4	10	9	9	10	10	9	11	15	11	18	10
Thamnophilidae	*Myrmoborus melanurus*				2	0	0	0	0	0	0	1	0	0	0	0	0	0	0	28	0
Thamnophilidae	*Hypocnemis cantator*				2	5	12	3	12	4	13	8	12	9	14	8	14	14	15	16	14
Thamnophilidae	*Hypocnemis hypoxantha*				2	6	1	3	0	4	0	7	0	11	1	10	0	13	0	19	0
Thamnophilidae	*Hypocnemoides melanopo*				2	5	10	3	9	4	9	6	9	8	11	6	12	12	12	13	11
Thamnophilidae	*Hypocnemoides maculicau*				2	6	16	3	16	4	17	9	15	11	19	7	19	17	19	19	17
Thamnophilidae	*Myrmochanes hemileucus*			x	2	12	13x	5	4x	1	13x	12	4x	12	13x	11	4x	11	13x	20	4x
Thamnophilidae	*Gymnocichla nudiceps*				2	21	5	30	8	25	7	37	5	29	6	36	10	38	11	46	7
Thamnophilidae	*Sclateria naevia*				2	5	9	3	9	4	9	9	8	9	10	8	11	15	12	17	10
Thamnophilidae	*Percnostola rufifrons*				2	3	14	3	12	3	14	8	12	7	14	5	14	15	16	13	13
Thamnophilidae	*Percnostola minor*				14	0	0	0	0	0	0	0	0	1	0	2	0	0	0	1	0
Thamnophilidae	*Percnostola schistacea*				2	2	0	1	0	2	0	4	0	6	0	5	0	6	0	11	0
Thamnophilidae	*Percnostola leucostigma*				2	5	8	4	7	4	7	8	7	9	9	8	9	15	10	17	9
Thamnophilidae	*Percnostola caurensis*				2	6	18	4	21	2	22	3	23	6	21	4	26	5	28	6	29
Thamnophilidae	*Percnostola lophotes*		H		2	0	4	0	3	0	3	0	2	0	6	0	5	0	5	0	4
Thamnophilidae	*Myrmeciza berlepschi*				2	13	3	14	6	17	0	4	6	23	3	14	6	17	6	18	6
Thamnophilidae	*Myrmeciza longipes*				2	7	11	5	12	7	12	8	11	12	14	10	18	15	18	15	17
Thamnophilidae	*Myrmeciza exsul*				2	18	4	26	7	21	6	32	5	27	5	31	9	31	9	42	6
Thamnophilidae	*Myrmeciza ferruginea*				2	4	15	3	15	4	15	8	13	8	15	6	16	15	17	15	15
Thamnophilidae	*Myrmeciza ruficauda*	T		x	2	8	13x	3	13x	27	8x	9	12x	16	15x	8	32x	42	22x	21	31x
Thamnophilidae	*Myrmeciza loricata*			x	2	7	12x	10	12x	9	9x	15	11x	9	24x	20	19x	34	14x	22	21x
Thamnophilidae	*Myrmeciza squamosa*				2	26	11	16	16	33	10	27	14	30	27	34	28	53	15	44	25
Thamnophilidae	*Myrmeciza laemosticta*				2	20	5	21	7	13	8	26	4	25	9	24	9	28	12	36	9
Thamnophilidae	*Myrmeciza nigricauda*				2	15	6	9	7	16	3	12	4	22	6	9	7	16	7	19	4
Thamnophilidae	*Myrmeciza disjuncta*				2	0	2	0	2	0	2	0	2	0	5	0	5	0	5	0	5
Thamnophilidae	*Myrmeciza pelzelni*				2	0	0	0	0	0	0	0	0	0	0	0	0	0	0	0	0
Thamnophilidae	*Myrmeciza hemimelaena*				2	6	10	5	10	5	11	10	10	10	12	10	13	14	12	18	12
Thamnophilidae	*Myrmeciza hyperythra*				2	4	4	2	4	3	3	6	4	7	5	7	5	8	5	12	5
Thamnophilidae	*Myrmeciza melanoceps*				2	5	0	4	0	4	0	8	0	10	0	10	0	12	0	18	0
Thamnophilidae	*Myrmeciza goeldii*				2	4	6	0	5	3	6	8	5	9	10	10	13	12	14	17	9
Thamnophilidae	*Myrmeciza fortis*				2	4	3	2	3	3	3	6	2	7	4	8	4	9	4	15	3

Family	Species	IUCN	Ag	R	Range Source	2050 TG H	2050 TG C	2050 AM H	2050 AM C	2050 GO H	2050 GO C	2050 OS H	2050 OS C	2100 TG H	2100 TG C	2100 AM H	2100 AM C	2100 GO H	2100 GO C	2100 OS H	2100 OS C
Thamnophilidae	*Myrmeciza immaculata*				2	13	3	17	6	13	3	18	4	23	7	19	10	23	13	31	7
Thamnophilidae	*Myrmeciza griseiceps*	T		x	2	5	10x	0	2x	0	11x	0	2x	5	10x	0	3x	0	11x	0	3x
Thamnophilidae	*Myrmeciza atrothorax*				2	5	11	3	12	4	12	8	11	9	13	7	14	14	14	15	14
Thamnophilidae	*Pithys albifrons*				2	5	8	4	8	5	8	8	7	9	9	8	10	14	11	15	10
Thamnophilidae	*Pithys castanea*			x	14	0	0x	36	0x	36	0x	0	0x	36	0x	36	0x	36	0x	36	0x
Thamnophilidae	*Gymnopithys rufigula*				2	3	13	2	13	3	13	5	13	6	15	5	17	11	18	10	17
Thamnophilidae	*Gymnopithys leucaspis*				2	7	1	7	1	6	1	11	1	11	1	12	2	14	2	20	1
Thamnophilidae	*Gymnopithys lunulata*				2	6	1	4	0	4	0	11	0	10	1	18	0	10	0	27	0
Thamnophilidae	*Gymnopithys salvini*				2	3	5	3	5	3	5	5	5	6	7	6	7	7	7	10	6
Thamnophilidae	*Myrmornis torquata*				2	6	11	4	11	4	11	10	10	10	12	8	13	19	14	19	13
Thamnophilidae	*Rhegmatorhina melanostict*				2	5	4	3	4	4	4	8	3	8	5	9	5	10	6	16	5
Thamnophilidae	*Rhegmatorhina cristata*				2	0	0	0	0	0	0	0	0	0	0	0	0	0	0	1	0
Thamnophilidae	*Rhegmatorhina hoffmannsi*				2	2	24	2	26	1	28	3	25	3	28	3	27	5	28	6	27
Thamnophilidae	*Rhegmatorhina berlepschi*				2	28	19x	5	27x	37	10x	31	10x	38	19x	19	27x	65	10x	69	10x
Thamnophilidae	*Rhegmatorhina gymnops*				2	12	0	6	0	4	0	17	0	17	0	19	0	38	0	35	0
Thamnophilidae	*Hylophylax naevioides*				2	18	4	26	6	22	5	31	4	26	5	31	7	32	7	40	5
Thamnophilidae	*Hylophylax naevia*				2	5	8	4	8	4	8	8	8	9	9	8	9	14	10	16	9
Thamnophilidae	*Hylophylax punctulata*				2	4	9	3	9	2	9	5	9	7	10	6	11	10	12	13	11
Thamnophilidae	*Hylophylax poecilinota*				2	5	10	4	10	4	10	8	10	9	11	8	12	14	13	16	12
Thamnophilidae	*Phlegopsis nigromaculata*				2	6	11	4	11	4	11	10	10	11	12	9	12	17	13	19	12
Thamnophilidae	*Skutchia borbae*				2	12	33	5	33	7	32	10	29	17	33	13	33	18	34	21	31
Thamnophilidae	*Phaenostictus mcleannani*				2	17	4	26	6	23	5	30	4	25	5	31	7	33	8	40	6
Furnariidae	*Geobates poecilopterus*				2	3	18	2	6	4	12	7	4	5	26	7	9	8	15	11	6
Furnariidae	*Geositta cunicularia*				2	4	7	3	8	4	8	5	7	5	10	6	13	8	15	9	13
Furnariidae	*Geositta maritima*				2	0	19	2	18	2	24	3	15	0	29	2	45	9	51	8	42
Furnariidae	*Geositta peruviana*				2	0	26	0	27	2	31	2	24	0	35	0	52	5	52	7	46
Furnariidae	*Geositta punensis*				2	5	11	3	14	2	14	10	9	6	15	7	20	9	21	17	12
Furnariidae	*Geositta saxicolina*				2	2	8	4	9	8	2	5	2	3	8	6	17	11	4	5	11
Furnariidae	*Geositta isabellina*				2	7	19	5	19	1	19	15	19	7	24	7	26	14	26	20	31
Furnariidae	*Geositta antarctica*				2	0	4	0	4	0	4	0	4	0	10	0	12	0	12	0	12
Furnariidae	*Geositta rufipennis*				2	8	10	7	10	4	10	9	8	11	13	10	14	11	16	17	13
Furnariidae	*Geositta crassirostris*			x	2	0	71x	7	65x	8	69x	12	57x	0	76x	9	69x	15	75x	19	60x
Furnariidae	*Geositta tenuirostris*				2	12	17	12	20	9	19	17	15	15	25	16	28	20	27	27	20
Furnariidae	*Upucerthia harterti*				2	23	4	16	4	22	4	25	1	30	4	23	11	30	4	36	1
Furnariidae	*Upucerthia certhioides*				2	2	11	0	10	2	11	2	9	2	15	1	19	2	20	2	17
Furnariidae	*Upucerthia ruficauda*				2	5	10	5	12	3	12	8	9	8	14	8	18	8	21	14	16
Furnariidae	*Upucerthia andaecola*			x	2	52	19x	3	19x	2	19x	52	6x	52	24x	39	24x	3	24x	57	6x
Furnariidae	*Upucerthia serrana*				2	0	4	7	4	6	1	4	0	0	6	7	8	10	1	7	4
Furnariidae	*Upucerthia dumetaria*				2	3	8	3	9	2	10	4	8	5	12	4	15	5	18	7	15
Furnariidae	*Upucerthia albigula*				2	0	67	0	71	6	65	1	63	0	87x	0	98x	20	80x	16	80x
Furnariidae	*Upucerthia jelskii*				2	12	20	12	23	9	22	16	18	15	28	17	31	22	30	29	22
Furnariidae	*Upucerthia validirostris*				2	0	7	1	6	0	7	2	6	1	8	2	10	1	13	3	9
Furnariidae	*Cinclodes fuscus*				2	4	10	4	10	3	10	6	9	6	13	7	16	8	18	10	15
Furnariidae	*Cinclodes comechingonus*			x	2	27	0x	0	0x	27	0x	4	0x	27	0x	0	0x	27	0x	14	0x

Family	Species	IUCN	Ag	R	Range Source	Percentage loss in geographic range															
						2050								2100							
						TG		AM		GO		OS		TG		AM		GO		OS	
						H	C	H	C	H	C	H	C	H	C	H	C	H	C	H	C
Furnariidae	*Cinclodes pabsti*		H	x	2	0	0x	0	1x	0	0x	0	0x	0	6x	0	1x	0	0x	0	0x
Furnariidae	*Cinclodes oustaleti*				2	3	9	3	8	3	9	4	8	6	14	6	15	9	18	9	17
Furnariidae	*Cinclodes excelsior*				2	20	0	27	0	23	0	44	0	31	7	56	3	70	0	44	2
Furnariidae	*Cinclodes aricomae*	T		x	14	7	15x	12	3x	8	7x	4	11x	13	17x	16	10x	18	13x	8	16x
Furnariidae	*Cinclodes patagonicus*				2	4	19	3	17	5	18	3	18	6	30	6	34	8	34	7	33
Furnariidae	*Cinclodes taczanowskii*				2	0	33	1	27	4	39x	4	24	0	52x	1	74x	17	62x	15	61x
Furnariidae	*Cinclodes nigrofumosus*				2	0	1	2	1	4	3	1	1	4	4	7	12	8	23	7	13
Furnariidae	*Cinclodes antarcticus*			x	2	0	12x	0	12x	0	12x	0	12x	0	20x	0	21x	0	21x	0	21x
Furnariidae	*Cinclodes atacamensis*				2	7	15	6	16	5	16	9	13	9	19	9	23	12	24	16	18
Furnariidae	*Cinclodes palliatus*	T			2	0	13	4	13	11	1	5	0	0	13	8	25x	15	5	5	15
Furnariidae	*Chilia melanura*			x	2	1	8x	7	0x	0	7x	0	7x	2	10x	9	0x	19	7x	17	15x
Furnariidae	*Furnarius minor*				2	17	8	22	2	21	7	18	1	33	8	35	2	42	7	43	1
Furnariidae	*Furnarius figulus*				2	9	22	9	18	9	20	16	13	12	28	13	20	17	23	23	16
Furnariidae	*Furnarius leucopus*		H		2	0	13	0	10	0	12	0	8	0	15	0	13	0	15	0	11
Furnariidae	*Furnarius cinnamomeus*				14	6	10	2	12	4	14	6	8	6	12	3	18	7	20	8	13
Furnariidae	*Furnarius longirostris*				14	15	4	14	7	14	7	14	3	19	9	18	12	19	19	17	11
Furnariidae	*Furnarius torridus*				2	3	1	4	0	2	0	6	0	5	1	13	0	3	0	26	0
Furnariidae	*Furnarius rufus*				2	8	12	5	9	7	10	10	7	10	17	10	14	14	15	16	12
Furnariidae	*Furnarius cristatus*				2	2	5	0	2	3	2	1	2	3	5	1	5	3	5	2	5
Furnariidae	*Sylviorthorhynchus desmur*				2	8	14	7	13	8	14	7	13	10	24	11	26	15	27	13	25
Furnariidae	*Aphrastura spinicauda*				2	6	12	5	12	6	12	5	12	9	21	9	22	12	23	11	22
Furnariidae	*Leptasthenura fuliginiceps*				2	9	10	8	14	5	13	12	8	12	13	9	18	11	18	17	11
Furnariidae	*Leptasthenura yanacensis*				2	17	15	21	22	11	22	32	5	25	21	24	27	29	26	48	5
Furnariidae	*Leptasthenura platensis*				2	1	5	0	4	1	5	1	4	1	6	1	8	2	9	1	8
Furnariidae	*Leptasthenura aegithaloide*				2	3	9	3	10	2	11	4	9	4	13	5	18	5	20	7	17
Furnariidae	*Leptasthenura striolata*				2	21	3	20	4	30	0	31	2	31	12	38	11	55	2	66	7
Furnariidae	*Leptasthenura pileata*				2	3	9	11	14	9	9	8	8	3	14	15	18	18	14	16	15
Furnariidae	*Leptasthenura xenothorax*	T		x	2	0	1x	0	0x	0	1x	0	1x	0	1x	0	43x	42	1x	42	1x
Furnariidae	*Leptasthenura striata*				2	5	33	6	38	8	35	11	29	5	46	9	57	16	52	22	43
Furnariidae	*Leptasthenura andicola*				2	10	7	13	6	10	5	14	5	13	12	20	14	25	13	20	10
Furnariidae	*Leptasthenura setaria*				2	23	7	21	7	31	4	33	6	31	16	37	15	56	7	62	11
Furnariidae	*Schizoeaca perijana*	T		x	2	0	0x	0	0x	14	0x	0	0x	14	0x	14	0x	14	0x	0	0x
Furnariidae	*Schizoeaca coryi*			x	2	37	0x	12	9x	10	9x	50	9x	37	12x	12	47x	36	45x	59	9x
Furnariidae	*Schizoeaca fuliginosa*				2	16	0	23	0	18	0	26	0	22	4	39	3	41	7	29	6
Furnariidae	*Schizoeaca griseomurina*			x	2	53	13x	21	12x	7	25x	7	24x	60	13x	44	12x	43	32x	25	31x
Furnariidae	*Schizoeaca palpebralis*			x	2	41	1x	40	1x	40	0x	1	40x	41	1x	40	1x	41	0x	1	40x
Furnariidae	*Schizoeaca vilcabambae*			x	14	0	0x	0	0x	0	0x	0	0x	0	0x	0	0x	0	0x	0	0x
Furnariidae	*Schizoeaca helleri*			x	2	0	22x	14	0x	14	1x	0	15x	7	22x	14	15x	29	8x	7	23x
Furnariidae	*Schizoeaca harterti*				2	12	8	9	6	12	4	13	2	12	8	10	6	12	4	14	2
Furnariidae	*Schizoeaca moreirae*			x	2	12	8x	27	8x	6	8x	20	8x	14	13x	31	24x	22	22x	43	11x
Furnariidae	*Schoeniophylax phryganop*				2	8	9	6	7	7	7	10	7	12	10	10	11	15	13	16	12
Furnariidae	*Synallaxis infuscata*	T		x	2	0	56x	1	55x	0	56x	0	56x	0	56x	1	55x	0	56x	0	56x
Furnariidae	*Synallaxis ruficapilla*				2	22	8	19	8	29	6	31	7	31	16	32	14	47	10	48	13
Furnariidae	*Synallaxis whitneyi*	T		x	14	26	72x	26	72x	99	0x	99	0x	26	72x	26	72x	99	0x	99	0x

Family	Species	IUCN	Ag	R	Range Source	Percentage loss in geographic range 2050 TG H	C	AM H	C	GO H	C	OS H	C	2100 TG H	C	AM H	C	GO H	C	OS H	C
Furnariidae	*Synallaxis frontalis*				2	6	15	4	9	5	12	10	7	8	19	7	12	10	15	15	9
Furnariidae	*Synallaxis azarae*				2	12	7	13	4	9	6	17	6	15	8	20	8	22	10	21	8
Furnariidae	*Synallaxis courseni*	T		x	14	0	0x	0	0x	0	0x	0	0x	0	0x	0	0x	0	0x	0	0x
Furnariidae	*Synallaxis albescens*		H		2	0	13	0	10	0	11	0	7	0	17	0	14	0	16	0	11
Furnariidae	*Synallaxis spixi*				2	19	7	16	6	26	5	27	5	28	12	29	12	41	8	42	11
Furnariidae	*Synallaxis brachyura*				2	16	4	19	5	19	5	24	3	21	5	23	7	25	7	31	5
Furnariidae	*Synallaxis albigularis*				2	20	7	9	5	18	6	30	1	25	7	27	7	38	7	51	2
Furnariidae	*Synallaxis hypospodia*				2	7	18	4	9	4	14	10	6	9	23	8	11	9	16	16	8
Furnariidae	*Synallaxis moesta*			x	2	33	1x	34	1x	20	1x	39	1x	35	1x	38	2x	43	1x	45	1x
Furnariidae	*Synallaxis macconnelli*				2	3	22	5	23	1	24	2	25	8	26	6	27	3	33	5	32
Furnariidae	*Synallaxis cabanisi*				2	26	7	24	4	20	6	39	5	34	7	32	8	39	7	49	6
Furnariidae	*Synallaxis subpudica*			x	2	31	0x	33	0x	33	0x	33	0x	31	0x	33	0x	33	0x	33	0x
Furnariidae	*Synallaxis tithys*	T		x	2	1	0x	0	0x	0	0x	1	0x	1	0x	0	7x	1	7x	1	7x
Furnariidae	*Synallaxis cinerascens*				2	22	7	20	7	30	6	31	6	33	14	34	13	49	8	49	11
Furnariidae	*Synallaxis propinqua*				2	10	16x	5	3x	1	16x	11	3x	14	16x	12	3x	13	16x	19	3x
Furnariidae	*Synallaxis hellmayri*				2	6	21	5	17	7	18	12	10	7	27	8	19	11	20	18	12
Furnariidae	*Synallaxis maranonica*	T		x	2	0	6x	6	0x	5	6x	5	6x	0	6x	10	0x	5	6x	5	6x
Furnariidae	*Synallaxis gujanensis*				2	7	12	4	12	5	12	10	11	11	13	9	15	17	15	18	14
Furnariidae	*Synallaxis simoni*				14	0	54	0	4	0	11	0	11	0	73	0	11	13	24	0	11
Furnariidae	*Synallaxis albilora*				2	2	10	1	11	1	11	10	8	6	10	6	12	11	13	23	10
Furnariidae	*Synallaxis rutilans*				2	4	10	3	11	3	11	7	10	8	12	7	13	12	13	14	12
Furnariidae	*Synallaxis cherriei*				2	4	10	3	11	4	13	10	12	10	14	6	13	24	13	21	13
Furnariidae	*Synallaxis unirufa*				2	14	5	13	5	12	6	21	7	22	6	20	13	29	10	25	9
Furnariidae	*Synallaxis castanea*			x	2	49	0x	0	0x	49	0x	17	0x	49	0x	49	0x	49	0x	17	0x
Furnariidae	*Synallaxis fuscorufa*	T		x	2	19	0x	49	0x	68	0x	19	0x	68	0x	49	0x	68	0x	68	0x
Furnariidae	*Synallaxis zimmeri*	T		x	2	0	0x	22	0x	1	0x	0	0x	0	38x	22	27x	2	26x	2	26x
Furnariidae	*Synallaxis erythrothorax*				2	8	5	18	3	12	5	26	3	10	6	23	13	17	10	32	6
Furnariidae	*Synallaxis cinnamomea*				2	23	0	24	4	20	4	29	4	29	5	30	10	33	13	34	8
Furnariidae	*Synallaxis stictothorax*				2	0	8	0	10	0	10	1	10	0	10	0	20	1	21	1	20
Furnariidae	*Synallaxis chinchipensis*			x	2	0	0x	0	0x	0	0x	0	0x	0	0x	0	0x	0	0x	0	0x
Furnariidae	*Synallaxis candei*		H		2	0	4	0	9	0	8	0	5	0	14	0	16	0	29	0	18
Furnariidae	*Synallaxis kollari*	T			2	0	12	0	12	0	12	0	12	0	23	0	24	0	15	0	13
Furnariidae	*Synallaxis scutata*				2	8	21	4	19	4	22	13	16	10	26	9	24	16	27	25	21
Furnariidae	*Hellmayrea gularis*				2	17	4	16	5	15	7	23	8	24	8	24	14	38	15	30	12
Furnariidae	*Cranioleuca erythrops*				2	17	7	23	10	17	10	29	7	22	7	26	11	29	12	40	8
Furnariidae	*Cranioleuca antisiensis*			x	2	17	19x	4	7x	2	22x	2	9x	19	19x	11	7x	11	22x	5	9x
Furnariidae	*Cranioleuca baroni*				2	0	2	7	2	3	0	5	0	0	4	7	3	8	2	8	1
Furnariidae	*Cranioleuca curtata*				2	12	7	7	4	8	6	19	9	16	7	15	13	26	10	25	11
Furnariidae	*Cranioleuca demissa*				2	15	16	10	22	4	23	7	26	21	22	18	27	15	31	16	38
Furnariidae	*Cranioleuca hellmayri*			x	2	17	0x	46	0x	64	0x	17	0x	64	0x	46	0x	64	0x	64	0x
Furnariidae	*Cranioleuca subcristata*				2	24	3	21	6	21	6	31	5	33	12	33	17	39	25	42	12
Furnariidae	*Cranioleuca pyrrhophia*				2	4	9	3	8	4	8	6	7	5	11	5	14	7	15	8	13
Furnariidae	*Cranioleuca henricae*	T		x	2	0	0x	0	0x	0	0x	0	0x	0	0x	0	0x	0	0x	0	0x
Furnariidae	*Cranioleuca obsoleta*				2	23	7	20	7	32	5	35	6	35	11	37	11	49	8	55	10

Family	Species	IUCN	Ag	R	Range Source	Percentage loss in geographic range 2050 TG H	C	AM H	C	GO H	C	OS H	C	2100 TG H	C	AM H	C	GO H	C	OS H	C
Furnariidae	*Cranioleuca marcapatae*			x	2	0	18x	0	0x	0	18x	0	18x	0	18x	0	50x	33	18x	33	18x
Furnariidae	*Cranioleuca albiceps*				2	10	1	9	1	10	1	15	1	11	1	16	2	16	2	15	2
Furnariidae	*Cranioleuca semicinerea*				2	10	22	8	17	9	19	16	11	11	26	12	19	14	21	23	13
Furnariidae	*Cranioleuca albicapilla*			x	2	10	2x	10	0x	10	2x	7	11x	17	2x	10	26x	34	2x	24	11x
Furnariidae	*Cranioleuca dissita*			x	2	0	0x	60	0x	60	0x	60	0x	0	0x	60	0x	60	0x	100	0x
Furnariidae	*Cranioleuca vulpina*				2	7	16	4	13	5	15	11	11	11	20	9	16	16	18	21	13
Furnariidae	*Cranioleuca vulpecula*				14	5	6	2	4	1	5	7	4	6	7	8	5	6	6	15	4
Furnariidae	*Cranioleuca muelleri*				2	21	1	25	0	12	1	21	0	31	1	34	0	47	1	46	0
Furnariidae	*Cranioleuca gutturata*				2	5	10	4	10	4	10	7	10	8	11	8	12	11	12	14	12
Furnariidae	*Cranioleuca sulphurifera*				2	5	1	3	1	7	1	7	1	7	1	7	1	10	1	9	1
Furnariidae	*Certhiaxis cinnamomea*				2	9	13	7	10	9	11	13	7	12	18	11	14	17	16	21	11
Furnariidae	*Certhiaxis mustelina*				2	15	13	19	9	14	11	14	9	25	14	27	11	30	14	33	10
Furnariidae	*Acrobatornis fonsecai*	T		x	14	0	0x	0	0x	0	0x	0	0x	82	0x	0	18x	0	18x	25	18x
Furnariidae	*Asthenes pyrrholeuca*				2	1	7	1	8	1	9	1	8	2	11	2	15	2	18	3	17
Furnariidae	*Asthenes baeri*				2	1	7	1	5	2	4	2	4	1	9	2	10	4	11	4	9
Furnariidae	*Asthenes pudibunda*				2	5	30	6	37	8	30	4	30	5	38	11	45	15	39	11	41
Furnariidae	*Asthenes ottonis*				2	3	1	3	0	0	4	0	4	3	1	3	1	1	4	1	4
Furnariidae	*Asthenes heterura*			x	2	8	12x	0	13x	5	8x	5	8x	8	12x	0	13x	5	8x	5	8x
Furnariidae	*Asthenes cactorum*				2	0	50	9	40	8	55	11	36	0	59	15	60	17	67	27	47
Furnariidae	*Asthenes modesta*				2	4	10	3	11	3	11	5	10	5	14	5	17	6	19	9	16
Furnariidae	*Asthenes luizae*	T		x	14	15	0x	7	0x	7	0x	15	0x	15	0x	7	0x	11	0x	19	0x
Furnariidae	*Asthenes dorbignyi*				2	12	9	11	8	10	6	18	3	16	10	13	12	16	11	22	3
Furnariidae	*Asthenes arequipae*				2	10	23	4	26	8	25	17	21	13	34	18	38	20	44	36	31
Furnariidae	*Asthenes huancavelicae*	T			2	4	0	9	5	5	4	9	4	5	0	16	7	10	12	10	12
Furnariidae	*Asthenes berlepschi*			x	2	1	70x	1	0x	1	0x	1	0x	1	70x	1	0x	1	0x	1	0x
Furnariidae	*Asthenes steinbachi*				2	0	11	0	10	0	12	0	10	2	13	1	14	1	17	2	16
Furnariidae	*Asthenes humicola*				2	0	2	5	0	6	1	1	1	8	3	13	2	17	7	13	6
Furnariidae	*Asthenes patagonica*				2	1	7	0	8	0	8	1	8	1	8	2	14	1	19	2	17
Furnariidae	*Asthenes humilis*				2	7	11	7	11	9	5	4	10	7	12	11	14	18	8	8	15
Furnariidae	*Asthenes wyatti*				2	11	13	16	6	12	9	17	10	17	16	23	11	33	12	27	11
Furnariidae	*Asthenes sclateri*				2	12	20	13	22	7	23	16	18	14	24	15	24	16	28	23	21
Furnariidae	*Asthenes anthoides*				2	1	5	3	5	2	6	1	5	3	8	4	9	4	8	4	8
Furnariidae	*Asthenes urubambensis*				2	13	6	13	0	14	0	13	7	14	6	18	4	24	1	14	9
Furnariidae	*Asthenes flammulata*				2	24	1	23	1	16	2	26	2	28	2	40	2	44	6	30	4
Furnariidae	*Asthenes virgata*			x	2	2	15x	2	15x	6	0x	1	2x	3	15x	3	19x	7	0x	2	5x
Furnariidae	*Asthenes maculicauda*			x	2	6	15x	6	13x	6	13x	6	13x	6	15x	14	13x	14	13x	6	13x
Furnariidae	*Asthenes hudsoni*				2	2	0	1	0	2	0	1	0	2	0	2	0	2	0	2	0
Furnariidae	*Thripophaga cherriei*	T		x	14	0	0x	0	0x	0	0x	0	0x	0	0x	0	0x	0	0x	0	0x
Furnariidae	*Thripophaga macroura*	T			2	6	4	7	6	22	2	7	4	25	5	10	19	36	14	32	18
Furnariidae	*Thripophaga berlepschi*	T		x	2	0	0x	0	0x	0	0x	6	0x	0	0x	0	6x	6	11x	6	0x
Furnariidae	*Thripophaga fusciceps*				2	14	10	8	9	9	9	16	8	20	11	14	10	24	10	26	10
Furnariidae	*Siptornopsis hypochondria*	T		x	2	0	0x	11	0x	11	0x	11	0x	0	0x	11	0x	11	0x	11	0x
Furnariidae	*Phacellodomus rufifrons*				2	11	17	9	14	10	16	18	9	14	21	14	18	17	20	26	12
Furnariidae	*Phacellodomus inornatus*				14	22	2	16	2	18	1	26	1	26	4	23	4	37	3	39	2

Family	Species	IUCN	Ag	R	Range Source	2050 TG H	2050 TG C	2050 AM H	2050 AM C	2050 GO H	2050 GO C	2050 OS H	2050 OS C	2100 TG H	2100 TG C	2100 AM H	2100 AM C	2100 GO H	2100 GO C	2100 OS H	2100 OS C
Furnariidae	*Phacellodomus sibilatrix*				2	1	6	1	4	1	4	1	3	1	9	1	11	2	13	2	12
Furnariidae	*Phacellodomus striaticeps*				2	13	13	14	15	8	16	15	11	18	15	16	19	18	20	25	13
Furnariidae	*Phacellodomus striaticollis*				2	7	2	4	2	9	1	7	1	9	3	10	3	14	1	12	2
Furnariidae	*Phacellodomus maculipect*				2	3	4	4	3	1	6	5	3	3	4	4	8	3	9	5	7
Furnariidae	*Phacellodomus ruber*				2	7	17	4	11	4	14	9	9	10	21	8	15	11	19	15	14
Furnariidae	*Phacellodomus dorsalis*	T		x	2	0	0x	0	0x	0	0x	0	0x	0	0x	0	0x	0	0x	0	0x
Furnariidae	*Phacellodomus erythrophth*				2	16	9	11	12	21	8	19	11	20	19	23	23	43	14	35	19
Furnariidae	*Clibanornis dendrocolaptoi*				2	25	6	22	6	32	4	35	4	36	12	38	12	50	8	58	10
Furnariidae	*Spartonoica maluroides*				2	3	5	1	5	3	5	3	5	4	6	2	8	4	9	4	8
Furnariidae	*Phleocryptes melanops*				2	4	8	4	8	4	8	5	7	5	11	6	13	8	14	9	12
Furnariidae	*Limnornis curvirostris*				2	4	1	5	1	5	1	4	0	6	1	6	1	10	1	9	1
Furnariidae	*Limnornis rectirostris*				2	7	0	7	0	12	0	9	0	11	0	15	0	21	0	19	0
Furnariidae	*Anumbius annumbi*				2	6	7	4	5	8	5	8	4	8	10	8	8	11	9	13	7
Furnariidae	*Coryphistera alaudina*				2	2	8	1	6	2	6	3	6	2	10	2	12	3	14	4	11
Furnariidae	*Eremobius phoenicurus*				2	1	3	1	4	0	4	1	4	2	5	2	9	1	12	2	11
Furnariidae	*Siptornis striaticollis*			x	2	11	10x	14	10x	8	10x	23	10x	19	10x	23	25x	43	13x	31	20x
Furnariidae	*Metopothrix aurantiacus*				2	3	4	2	3	3	3	6	3	6	5	8	5	7	5	13	4
Furnariidae	*Xenerpestes minlosi*			x	2	8	0x	10	0x	8	0x	15	0x	16	2x	17	0x	18	2x	21	2x
Furnariidae	*Xenerpestes singularis*			x	2	16	19x	18	16x	8	20x	27	20x	27	19x	28	38x	52	22x	42	34x
Furnariidae	*Roraimia adusta*				2	15	9	12	16	7	17	4	21	24	18	23	28	18	33	10	45
Furnariidae	*Premnornis guttuligera*				2	16	5	15	4	14	4	25	5	25	6	24	11	32	8	30	8
Furnariidae	*Premnoplex brunnescens*				2	14	6	13	5	13	6	23	6	22	6	20	12	28	11	29	8
Furnariidae	*Premnoplex tatei*	T		x	2	0	0x	0	0x	0	0x	0	0x	0	0x	6	0x	6	0x	6	0x
Furnariidae	*Margarornis rubiginosus*			x	2	17	20x	29	32x	5	32x	10	32x	19	20x	29	34x	39	33x	39	32x
Furnariidae	*Margarornis stellatus*			x	2	0	0x	16	1x	5	0x	16	0x	13	0x	17	1x	5	1x	16	0x
Furnariidae	*Margarornis bellulus*			x	2	46	0x	0	46x	0	46x	46	0x	46	0x	0	46x	0	47x	100	0x
Furnariidae	*Margarornis squamiger*				2	17	5	17	4	17	6	28	4	26	7	26	10	35	9	34	7
Furnariidae	*Lochmias nematura*				2	16	9	14	8	18	8	22	8	23	13	23	12	33	10	34	12
Furnariidae	*Pseudoseisura cristata*				2	9	20	8	16	9	19	17	9	10	27	12	18	14	21	23	11
Furnariidae	*Pseudoseisura unirufa*				14	4	15	1	15	1	17	9	14	9	15	6	20	11	21	19	18
Furnariidae	*Pseudoseisura lophotes*				2	1	6	1	5	2	5	3	4	2	8	2	10	3	11	4	9
Furnariidae	*Pseudoseisura gutturalis*				2	1	8	1	9	1	9	1	9	1	10	2	16	1	20	2	17
Furnariidae	*Pseudocolaptes lawrencii*			x	2	19	20x	30	31x	7	31x	12	31x	21	20x	30	33x	41	33x	41	31x
Furnariidae	*Pseudocolaptes johnsoni*			x	2	9	11x	8	11x	9	11x	22	0x	30	11x	9	11x	11	11x	22	0x
Furnariidae	*Pseudocolaptes boissonne*				2	15	4	14	3	14	4	19	5	21	6	22	7	25	8	22	7
Furnariidae	*Berlepschia rikeri*				2	5	12	3	12	3	12	8	11	9	13	6	14	15	15	16	13
Furnariidae	*Ancistrops strigilatus*				2	5	6	4	6	4	6	8	6	9	7	9	7	13	7	16	7
Furnariidae	*Cichlocolaptes leucophrus*				2	25	12	14	19	27	11	22	16	27	26	24	32	48	17	35	29
Furnariidae	*Hyloctistes subulatus*				2	4	6	3	6	3	6	6	6	8	7	7	7	10	7	13	7
Furnariidae	*Hyloctistes virgatus*				14	21	4	26	9	26	7	32	6	31	6	30	12	31	11	43	8
Furnariidae	*Syndactyla guttulata*			x	2	4	0x	0	0x	4	0x	0	0x	4	0x	7	0x	7	0x	3	0x
Furnariidae	*Syndactyla subalaris*				2	15	5	17	6	13	7	23	6	23	7	23	12	32	12	31	9
Furnariidae	*Syndactyla rufosuperciliata*				2	11	6	11	4	16	3	17	4	16	10	18	9	27	6	27	7
Furnariidae	*Syndactyla ruficollis*	T		x	2	3	8x	2	6x	0	10x	0	8x	3	8x	2	7x	0	11x	0	8x

Family	Species	IUCN	Ag	R	Range Source	2050 TG (H C)	2050 AM (H C)	2050 GO (H C)	2050 OS (H C)	2100 TG (H C)	2100 AM (H C)	2100 GO (H C)	2100 OS (H C)
Furnariidae	*Anabacerthia variegaticeps*				2	10 2	18 3	15 5	22 2	12 2	19 5	21 5	27 2
Furnariidae	*Anabacerthia striaticollis*				2	15 5	13 4	15 5	23 5	23 7	20 12	28 9	28 8
Furnariidae	*Philydor pyrrhodes*				2	4 11	3 10	3 11	6 10	7 12	5 13	11 13	12 12
Furnariidae	*Philydor dimidiatus*				2	9 17	4 10	6 13	14 7	16 20	9 11	16 15	23 8
Furnariidae	*Philydor fuscipennis*				2	16 5	21 8	13 7	24 6	22 9	23 9	26 12	36 10
Furnariidae	*Philydor erythrocercus*				2	6 11	4 12	4 12	9 11	10 13	9 13	16 14	18 12
Furnariidae	*Philydor erythropterus*				2	5 9	4 9	4 10	8 9	10 10	9 11	13 11	17 10
Furnariidae	*Philydor amaurotis*				2	26 11	23 11	32 8	38 9	36 18	39 19	54 12	60 16
Furnariidae	*Philydor lichtensteini*				2	18 10	16 9	22 7	26 8	27 17	26 14	38 10	40 13
Furnariidae	*Philydor rufus*				2	18 11	16 10	17 10	27 8	25 14	24 14	33 13	39 11
Furnariidae	*Philydor atricapillus*				2	19 10	18 10	27 7	29 9	29 18	30 18	47 12	46 17
Furnariidae	*Philydor novaesi*	T		x	14	0⊦02x	0⊦02x	0⊦02x	0⊦02x	0⊦02x	0⊦02x	0⊦02x	0⊦02x
Furnariidae	*Simoxenops ucayalae*				2	7 7	4 6	7 7	12 6	16 9	15 8	16 8	22 8
Furnariidae	*Simoxenops striatus*	T		x	2	22 0x	12 0x	22 0x	12 0x	22 0x	22 0x	22 0x	22 0x
Furnariidae	*Anabazenops fuscus*				2	21 12	15 15	20 12	17 14	25 26	19 28	39 18	28 25
Furnariidae	*Thripadectes ignobilis*				2	19 4	15 9	16 4	22 5	23 4	16 9	17 9	27 5
Furnariidae	*Thripadectes rufobrunneus*			x	2	17 12x	30 18x	18 18x	30 18x	22 12x	30 20x	47 20x	48 18x
Furnariidae	*Thripadectes virgaticeps*			x	2	3 2x	13 2x	6 2x	21 0x	12 2x	25 4x	19 3x	23 1x
Furnariidae	*Thripadectes melanorhync*				2	12 9	9 6	13 9	24 9	21 10	18 15	33 11	34 10
Furnariidae	*Thripadectes holostictus*				2	15 5	11 3	13 5	23 5	22 6	21 11	29 9	28 8
Furnariidae	*Thripadectes flammulatus*				2	22 11	34 8x	18 15	33 15x	40 13x	48 19x	54 19x	41 24x
Furnariidae	*Thripadectes scrutator*				2	10 1	9 0	10 0	15 3	19 1	11 8	22 4	19 5
Furnariidae	*Automolus ochrolaemus*				2	6 9	5 10	5 10	10 9	10 11	10 12	15 12	17 11
Furnariidae	*Automolus dorsalis*				14	10 2	8 2	10 2	18 2	18 3	23 3	22 2	35 2
Furnariidae	*Automolus infuscatus*				2	4 9	3 9	3 9	7 9	8 10	7 11	13 12	15 11
Furnariidae	*Automolus leucophthalmus*				2	12 12	11 7	16 7	17 6	18 18	17 13	29 11	27 11
Furnariidae	*Automolus roraimae*				2	10 23	7 28	4 29	1 31	14 30	12 39	8 43	4 50
Furnariidae	*Automolus melanopezus*				2	6 11	3 10	3 10	10 10	10 13	9 15	13 15	19 13
Furnariidae	*Automolus rubiginosus*				2	6 9	6 9	6 9	10 9	10 10	12 11	14 12	19 11
Furnariidae	*Automolus rufipileatus*				2	6 8	5 8	5 8	10 8	9 9	9 10	16 11	18 10
Furnariidae	*Hylocryptus rectirostris*				2	6 16	5 5	5 10	10 3	11 22	9 6	12 11	14 5
Furnariidae	*Hylocryptus erythrocephalu*	T		x	2	1 9x	1 5x	0 10x	0 5x	1 9x	1 11x	0 17x	0 12x
Furnariidae	*Sclerurus mexicanus*				2	6 9	5 9	5 9	9 8	10 10	9 11	16 11	18 10
Furnariidae	*Sclerurus rufigularis*				2	4 10	3 9	3 10	6 9	7 11	6 11	11 12	13 11
Furnariidae	*Sclerurus albigularis*				2	20 7	19 6	15 7	29 5	27 7	27 9	30 10	38 7
Furnariidae	*Sclerurus caudacutus*				2	4 8	3 8	4 8	7 7	8 9	7 10	13 10	15 9
Furnariidae	*Sclerurus scansor*				2	13 12	11 9	18 10	21 8	18 17	19 13	31 12	35 11
Furnariidae	*Sclerurus guatemalensis*				2	19 5	27 7	21 7	37 4	24 6	34 11	32 10	45 6
Furnariidae	*Heliobletus contaminatus*				2	24 8	23 7	34 5	37 6	36 14	40 12	54 8	58 10
Furnariidae	*Xenops milleri*				2	3 9	2 8	2 9	4 8	5 10	5 10	7 11	10 10
Furnariidae	*Xenops tenuirostris*				2	5 12	4 13	4 13	8 13	10 14	9 15	14 16	17 15
Furnariidae	*Xenops minutus*				2	7 11	6 11	6 11	11 10	11 13	10 14	17 15	20 13
Furnariidae	*Xenops rutilans*				2	9 17	7 17	8 18	14 16	15 20	12 21	22 21	25 19
Furnariidae	*Megaxenops parnaguae*				2	8 23	5 18	7 20	16 8	9 29	10 19	11 22	23 10

Family	Species	IUCN	Ag	R	Range Source	2050 TG H	2050 TG C	2050 AM H	2050 AM C	2050 GO H	2050 GO C	2050 OS H	2050 OS C	2100 TG H	2100 TG C	2100 AM H	2100 AM C	2100 GO H	2100 GO C	2100 OS H	2100 OS C
Furnariidae	*Pygarrhichas albogularis*				2	7	15	6	14	8	15	6	15	8	24	10	27	13	27	12	26
Furnariidae	*Drymornis bridgesii*				2	1	5	0	3	1	4	1	3	1	7	0	8	2	10	1	9
Furnariidae	*Nasica longirostris*				2	5	10	3	10	3	11	7	10	9	12	7	12	14	13	16	11
Furnariidae	*Dendrocincla tyrannina*				2	18	4	21	3	17	6	28	7	27	6	30	9	36	9	32	10
Furnariidae	*Dendrocincla fuliginosa*				2	6	11	5	11	5	12	9	11	10	13	9	14	15	14	17	13
Furnariidae	*Dendrocincla turdina*				2	21	9	19	9	29	7	29	8	32	16	32	17	47	11	48	15
Furnariidae	*Dendrocincla anabatina*				2	13	5	24	4	17	5	32	4	17	5	30	11	25	10	39	6
Furnariidae	*Dendrocincla merula*				2	4	9	3	9	3	9	7	9	8	10	6	11	13	12	14	11
Furnariidae	*Dendrocincla homochroa*				2	13	4	25	4	17	6	34	4	18	6	31	12	26	12	42	6
Furnariidae	*Deconychura longicauda*				2	4	9	3	9	4	9	8	9	8	10	7	11	13	11	15	10
Furnariidae	*Deconychura stictolaema*				2	4	7	3	6	2	6	6	6	7	7	6	7	11	8	13	6
Furnariidae	*Sittasomus griseicapillus*				2	7	12	5	11	6	12	11	10	11	14	10	15	16	16	20	14
Furnariidae	*Glyphorynchus spirurus*				2	6	10	5	10	5	10	9	9	10	11	9	12	15	12	17	11
Furnariidae	*Dendrexetastes rufigula*				2	4	10	4	10	4	10	8	9	8	11	9	11	11	12	15	11
Furnariidae	*Hylexetastes stresemanni*				2	1	3	2	3	2	3	3	3	4	4	4	5	4	6	6	4
Furnariidae	*Hylexetastes perrotii*				2	3	14	2	12	3	13	6	12	6	14	5	15	14	16	13	15
Furnariidae	*Hylexetastes uniformis*				2	5	19	3	20	3	22	8	19	8	22	8	22	14	22	15	21
Furnariidae	*Hylexetastes brigidai*				2	9	12	5	16	5	17	23	14	19	15	11	17	44	17	50	14
Furnariidae	*Xiphocolaptes promeropirh*				2	7	8	6	8	6	9	11	8	12	10	12	11	17	11	20	10
Furnariidae	*Xiphocolaptes albicollis*				2	18	9	16	7	25	6	26	6	27	14	28	12	42	9	41	11
Furnariidae	*Xiphocolaptes falcirostris*	T			2	6	12	2	11	5	11	12	5	7	12	5	11	13	13	25	6
Furnariidae	*Xiphocolaptes major*				2	7	13	5	12	4	12	9	11	10	16	8	20	14	22	16	19
Furnariidae	*Dendrocolaptes certhia*				2	5	11	3	11	4	11	8	11	9	12	7	13	14	13	15	12
Furnariidae	*Dendrocolaptes sanctithom*				14	14	4	22	5	17	5	28	3	19	5	27	10	25	10	36	5
Furnariidae	*Dendrocolaptes hoffmannsi*				2	7	19	4	20	3	21	8	19	11	21	9	21	15	22	17	20
Furnariidae	*Dendrocolaptes picumnus*				2	6	9	5	9	5	9	10	9	10	10	9	12	15	13	18	12
Furnariidae	*Dendrocolaptes platyrostris*				2	10	13	7	8	11	9	16	5	14	18	13	11	20	13	26	8
Furnariidae	*Xiphorhynchus picus*				2	7	13	5	13	5	13	11	11	11	15	9	16	16	17	19	14
Furnariidae	*Xiphorhynchus necopinus*				2	6	7	5	4	4	5	7	4	11	7	7	4	10	5	13	4
Furnariidae	*Xiphorhynchus obsoletus*				2	5	12	3	12	4	12	8	12	9	14	7	14	14	15	16	14
Furnariidae	*Xiphorhynchus ocellatus*				2	6	4	4	4	5	4	8	4	9	5	10	5	12	6	16	5
Furnariidae	*Xiphorhynchus spixii*				2	5	8	4	9	4	9	9	8	10	10	9	10	15	10	17	10
Furnariidae	*Xiphorhynchus pardalotus*				2	3	14	3	14	3	14	5	13	6	15	5	17	11	18	10	17
Furnariidae	*Xiphorhynchus guttatus*				2	4	13	3	12	4	13	7	12	8	15	7	16	11	16	13	15
Furnariidae	*Xiphorhynchus eytoni*				14	8	9	5	9	6	8	15	8	15	9	10	10	30	11	31	9
Furnariidae	*Xiphorhynchus susurrans*				2	21	4	25	7	23	7	33	5	27	7	33	11	34	12	41	8
Furnariidae	*Xiphorhynchus lachrymosu*				2	21	4	28	7	25	6	34	4	31	5	33	9	33	9	45	6
Furnariidae	*Xiphorhynchus erythropygi*				2	14	2	20	4	17	4	23	3	21	3	22	6	21	7	31	4
Furnariidae	*Xiphorhynchus triangularis*				2	13	3	13	2	12	3	19	5	20	5	20	8	27	8	26	6
Furnariidae	*Lepidocolaptes leucogaster*				2	15	12	35	6	18	11	44	6	22	15	37	14	26	22	50	12
Furnariidae	*Lepidocolaptes souleyetii*				2	16	5	17	6	15	6	23	3	19	7	22	10	26	11	30	7
Furnariidae	*Lepidocolaptes angustirostr*				2	7	14	5	11	6	13	11	9	10	19	8	15	14	17	18	13
Furnariidae	*Lepidocolaptes affinis*				2	9	3	18	4	12	6	21	4	11	3	19	9	19	7	26	4
Furnariidae	*Lepidocolaptes lacrymiger*				2	15	4	15	3	12	5	20	5	22	6	23	9	28	10	24	8

Percentage loss in geographic range

Family	Species	IUCN	Ag	R	Range Source	Percentage loss in geographic range 2050								2100							
						TG		AM		GO		OS		TG		AM		GO		OS	
						H	C	H	C	H	C	H	C	H	C	H	C	H	C	H	C
Furnariidae	*Lepidocolaptes squamatus*				2	15	11	14	4	18	4	22	3	19	16	20	7	29	7	28	4
Furnariidae	*Lepidocolaptes falcinellus*				14	26	10	19	10	35	7	38	8	35	16	38	16	55	9	58	13
Furnariidae	*Lepidocolaptes wagleri*			x	14	0	72x	0	35x	0	49x	14	0x	0	72x	14	35x	0	72x	14	0x
Furnariidae	*Lepidocolaptes fuscus*				2	15	13	14	11	20	10	23	9	21	20	22	16	34	14	36	14
Furnariidae	*Lepidocolaptes albolineatu*				2	5	13	4	13	4	13	8	12	9	14	8	15	14	16	16	15
Furnariidae	*Campylorhamphus pucher*			x	2	18	6x	22	9x	9	9x	27	9x	29	6x	38	26x	57	14x	36	24x
Furnariidae	*Campylorhamphus trochilir*				2	7	14	5	14	6	14	10	12	11	17	10	18	14	19	18	17
Furnariidae	*Campylorhamphus falculari*				2	23	9	20	9	33	6	32	7	31	16	35	15	50	9	50	13
Furnariidae	*Campylorhamphus pusillus*				2	18	6	20	8	16	9	27	7	24	8	26	13	32	10	37	9
Furnariidae	*Campylorhamphus procurv*				2	4	7	3	7	3	7	7	6	7	8	5	8	14	8	14	7
Formicariidae	*Formicarius colma*				2	5	11	3	11	4	11	8	11	9	12	7	13	14	14	16	13
Formicariidae	*Formicarius moniliger*				2	10	5	20	3	12	5	27	3	13	6	25	12	18	11	34	6
Formicariidae	*Formicarius analis*				2	5	8	4	8	4	8	9	7	10	9	9	9	16	10	18	9
Formicariidae	*Formicarius hoffmanni*				14	19	4	26	7	22	7	33	4	27	7	33	11	35	13	44	7
Formicariidae	*Formicarius rufifrons*				2	1	0	0	0	0	0	0	0	1	0	0	0	1	0	0	0
Formicariidae	*Formicarius nigricapillus*				2	13	4	16	7	17	3	13	5	21	4	16	7	21	7	24	5
Formicariidae	*Formicarius rufipectus*				2	10	8	9	5	10	7	17	8	15	8	16	11	23	10	24	9
Formicariidae	*Chamaeza campanisona*				2	16	8	13	8	18	7	22	7	22	13	22	14	34	11	34	12
Formicariidae	*Chamaeza nobilis*				2	4	6	2	6	3	6	6	5	8	7	7	7	10	7	14	6
Formicariidae	*Chamaeza turdina*			x	2	29	0x	1	0x	31	0x	7	0x	31	0x	29	1x	31	0x	7	2x
Formicariidae	*Chamaeza meruloides*				2	25	16	19	28	20	21	19	26	26	42x	28	45x	50	28x	35	44x
Formicariidae	*Chamaeza ruficauda*				2	25	4	19	8	29	5	33	6	34	14	38	15	53	8	55	13
Formicariidae	*Chamaeza mollissima*				2	11	9	15	3	13	7	19	9	16	11	23	12	31	9	25	14
Formicariidae	*Pittasoma michleri*				2	15	9	26	13	10	13	33	9	23	14	29	13	31	18	49	14
Formicariidae	*Pittasoma rufopileatum*				2	19	1	18	7	23	0	9	7	27	1	18	7	27	7	29	7
Formicariidae	*Grallaria squamigera*				2	12	6	12	4	13	6	19	6	17	8	18	12	26	10	24	9
Formicariidae	*Grallaria gigantea*	T		x	2	8	13x	31	17x	32	17x	59	0x	20	25x	65	17x	66	17x	59	0x
Formicariidae	*Grallaria excelsa*	T		x	2	18	0x	10	5x	16	5x	37	5x	21	7x	11	26x	36	24x	45	6x
Formicariidae	*Grallaria varia*				2	8	11	7	10	9	9	12	9	13	13	12	14	23	13	23	12
Formicariidae	*Grallaria guatimalensis*				2	10	8	11	8	9	9	18	9	15	11	16	14	19	14	24	13
Formicariidae	*Grallaria alleni*	T		x	2	6	77x	6	82x	6	82x	94	0x	6	77x	18	82x	18	82x	94	0x
Formicariidae	*Grallaria chthonia*	T		x	14	100	0x	0⊦02x		0⊦02x		0⊦02x		100	0x	0⊦02x		0⊦02x		0⊦02x	
Formicariidae	*Grallaria haplonota*				2	13	12	11	12	18	12x	30	10x	20	15x	33	22x	49	14x	44	16x
Formicariidae	*Grallaria dignissima*				2	9	0	1	0	2	0	11	0	14	0	12	0	14	0	21	0
Formicariidae	*Grallaria eludens*				2	3	0	0	0	5	0	9	0	9	0	20	0	12	0	25	0
Formicariidae	*Grallaria kaestneri*	T		x	14	12	0x	12	0x	12	0x	12	0x	12	0x	12	0x	12	0x	12	0x
Formicariidae	*Grallaria bangsi*	T		x	2	0	0x	50	0x	50	0x	0	0x	50	0x	50	0x	50	0x	50	0x
Formicariidae	*Grallaria ruficapilla*				2	17	5	18	5	12	8	23	6	22	9	29	9	31	14	28	8
Formicariidae	*Grallaria watkinsi*			x	2	14	17x	0	0x	0	17x	8	0x	14	17x	0	0x	8	17x	8	0x
Formicariidae	*Grallaria andicola*				2	3	5	8	3	8	1	6	4	4	5	9	8	13	5	9	7
Formicariidae	*Grallaria rufocinerea*	T		x	2	23	0x	15	0x	17	0x	16	0x	23	2x	24	0x	17	0x	16	0x
Formicariidae	*Grallaria nuchalis*			x	2	16	11x	20	9x	15	12x	32	11x	23	16x	41	20x	50	15x	38	19x
Formicariidae	*Grallaria carrikeri*			x	2	0	0x	0	0x	0	0x	0	0x	0	0x	0	0x	0	0x	0	0x
Formicariidae	*Grallaria albigula*				2	9	4	8	0	10	1	8	4	9	4	11	1	12	1	11	4

Family	Species	IUCN	Ag	R	Range Source	2050 TG H	2050 TG C	2050 AM H	2050 AM C	2050 GO H	2050 GO C	2050 OS H	2050 OS C	2100 TG H	2100 TG C	2100 AM H	2100 AM C	2100 GO H	2100 GO C	2100 OS H	2100 OS C
Formicariidae	*Grallaria flavotincta*				2	10	2	17	2x	8	2	22	0x	19	2x	20	2x	8	2	22	0x
Formicariidae	*Grallaria hypoleuca*				2	12	11	12	8	9	11	21	11	16	12	25	16	35	12	31	16
Formicariidae	*Grallaria przewalskii*			x	2	0	0x	0	0x	0	0x	1	0x	0	0x	0	1x	1	12x	1	0x
Formicariidae	*Grallaria capitalis*			x	2	0	0x	0	0x	0	0x	0	0x	0	0x	0	0x	0	0x	0	0x
Formicariidae	*Grallaria erythroleuca*			x	2	0	5x	0	0x	0	5x	0	5x	0	5x	0	19x	13	5x	14	5x
Formicariidae	*Grallaria griseonucha*			x	2	25	0x	21	8x	20	8x	57	8x	27	18x	21	38x	47	41x	71	8x
Formicariidae	*Grallaria rufula*				2	13	7	19	4	14	6	22	8	20	9	26	11	32	11	25	13
Formicariidae	*Grallaria blakei*			x	2	7	0x	4	0x	4	0x	3	4x	7	0x	4	0x	7	0x	3	4x
Formicariidae	*Grallaria erythrotis*			x	2	11	3x	11	2x	11	2x	18	1x	11	3x	17	2x	17	2x	18	1x
Formicariidae	*Grallaria quitensis*				2	14	7	25	4	17	8	30	8	20	12	36	8	41	13	31	13
Formicariidae	*Grallaria milleri*	T		x	2	0	0x	0	0x	0	0x	0	0x	0	0x	0	0x	0	0x	0	0x
Formicariidae	*Hylopezus perspicillatus*				2	19	2	25	5	24	4	30	3	28	3	30	7	31	7	39	5
Formicariidae	*Hylopezus macularius*				2	4	11	3	10	3	10	7	10	7	11	5	12	14	13	14	11
Formicariidae	*Hylopezus auricularis*	T		x	14	0	0x	0	0x	0	0x	0	0x	0	0x	0	0x	0	0x	0	0x
Formicariidae	*Hylopezus dives*				2	17	2	25	5	24	3	31	3	27	3	32	7	34	7	41	4
Formicariidae	*Hylopezus fulviventris*				2	10	2	2	2	4	0	12	2	16	2	13	2	18	0	21	2
Formicariidae	*Hylopezus berlepschi*				2	9	14	6	14	7	15	12	14	15	17	13	17	21	18	23	16
Formicariidae	*Hylopezus ochroleucus*				2	10	21	8	19	10	19	19	12	11	25	11	21	17	22	24	13
Formicariidae	*Hylopezus nattereri*				2	21	7	19	8	29	5	29	6	30	19	32	20	50	11	54	14
Formicariidae	*Myrmothera campanisona*				2	4	8	3	7	3	7	7	7	7	8	7	9	11	9	13	8
Formicariidae	*Myrmothera simplex*				2	10	18	6	22	6	24	1	27	12	24	12	36	10	40	4	47
Formicariidae	*Grallaricula flavirostris*				2	15	6	17	4	14	6	27	7	23	6	26	9	29	8	32	9
Formicariidae	*Grallaricula ferrugineipectu*				2	26	0	23	2	28	2	36	2	40	10	39	15x	44	20x	50	4x
Formicariidae	*Grallaricula leymebambae*				14	5	11	6	0	6	8	11	9	6	11	7	14x	13	9x	16	9x
Formicariidae	*Grallaricula nana*				2	21	5	14	12	14	11	18	14	26	14	27	24	36	21	25	28
Formicariidae	*Grallaricula loricata*			x	2	26	0x	10	0x	21	0x	5	0x	28	1x	27	1x	21	7x	5	11x
Formicariidae	*Grallaricula peruviana*			x	2	13	29x	36	11x	0	40x	15	40x	13	29x	36	11x	5	42x	17	42x
Formicariidae	*Grallaricula ochraceifrons*	T		x	2	0	0x	0	0x	0	0x	0	0x	0	0x	0	0x	0	0x	0	0x
Formicariidae	*Grallaricula lineifrons*			x	2	26	0x	30	0x	25	0x	47	0x	26	25x	87	0x	84	16x	60	16x
Formicariidae	*Grallaricula cucullata*	T		x	2	0	0x	11	0x	0	0x	18	0x	11	0x	14	0x	11	3x	21	0x
Conopophagidae	*Conopophaga lineata*				2	12	12	10	5	15	6	17	4	17	19	17	10	27	9	27	7
Conopophagidae	*Conopophaga cearae*				14	18	32	26	28	20	27	19	28	22	38	32	38	38	32	21	48
Conopophagidae	*Conopophaga aurita*				2	4	8	3	7	3	8	7	7	8	8	6	8	14	9	14	8
Conopophagidae	*Conopophaga roberti*				2	6	11	1	11	5	11	14	6	8	13	3	17	14	19	28	13
Conopophagidae	*Conopophaga peruviana*				2	4	3	3	3	3	3	8	3	8	4	10	5	8	4	17	4
Conopophagidae	*Conopophaga ardesiaca*				2	14	8	9	3	11	4	22	4	16	8	16	6	20	7	24	7
Conopophagidae	*Conopophaga castaneicep*				2	14	5	13	5	16	6	27	6	25	6	26	7	31	6	36	6
Conopophagidae	*Conopophaga melanops*				2	13	13	9	18	19	14	10	16	23	27	15	35	42	24	31	34
Conopophagidae	*Conopophaga melanogast*				2	8	17	4	17	4	17	9	16	14	17	9	17	22	17	20	16
Rhinocryptidae	*Pteroptochos tarnii*				2	10	18	8	17	11	19	9	18	11	27	13	32	18	33	16	31
Rhinocryptidae	*Pteroptochos castaneus*				2	10	0	5	0	0	0	6	0	10	6	10	0	12	0	6	0
Rhinocryptidae	*Pteroptochos megapodius*				2	0	4	5	0	4	2	0	2	7	5	11	0	14	3	10	5
Rhinocryptidae	*Scelorchilus albicollis*				2	0	2	4	0	6	1	1	1	7	3	12	2	15	7	11	6
Rhinocryptidae	*Scelorchilus rubecula*				2	11	18	10	17	12	19	9	18	14	29	15	32	21	33	18	31

Family	Species	IUCN	Ag	R	Range Source	2050 TG H	2050 TG C	2050 AM H	2050 AM C	2050 GO H	2050 GO C	2050 OS H	2050 OS C	2100 TG H	2100 TG C	2100 AM H	2100 AM C	2100 GO H	2100 GO C	2100 OS H	2100 OS C
Rhinocryptidae	*Rhinocrypta lanceolata*				2	1	11	0	11	1	12	2	10	1	14	1	19	2	22	2	18
Rhinocryptidae	*Teledromas fuscus*				2	1	12	1	13	1	14	1	13	1	16	1	23	1	27	2	25
Rhinocryptidae	*Liosceles thoracicus*				2	4	7	4	7	4	6	8	6	8	8	9	8	10	8	15	7
Rhinocryptidae	*Psilorhamphus guttatus*				2	14	9	12	10	20	7	22	9	20	21	20	20	42	11	44	17
Rhinocryptidae	*Merulaxis ater*				2	24	13	11	27	27	16	19	22	26	35	21	43	52	21	35	39
Rhinocryptidae	*Eugralla paradoxa*				2	9	11	6	10	13	12	8	9	9	17	14	13	19	12	16	12
Rhinocryptidae	*Myornis senilis*				2	11	4	15	3	10	5	20	4	17	5	26	8	31	9	24	8
Rhinocryptidae	*Melanopareia torquata*				2	6	20	2	14	4	18	9	12	9	26	6	17	10	21	14	14
Rhinocryptidae	*Melanopareia maximiliani*				2	1	6	1	4	1	4	1	4	1	8	1	8	2	9	2	9
Rhinocryptidae	*Melanopareia elegans*				2	5	18	0	21	1	24	1	21	6	21	1	26	2	29	2	26
Rhinocryptidae	*Melanopareia maranonica*			x	2	0	5x	5	0x	0	5x	0	5x	0	5x	5	0x	0	5x	0	5x
Rhinocryptidae	*Scytalopus latrans*				14	19	4	21	3	11	6	24	4	26	7	34	8	34	12	31	8
Rhinocryptidae	*Scytalopus unicolor*			x	2	0	0x	0	0x	0	0x	0	0x	0	0x	0	0x	0	0x	0	0x
Rhinocryptidae	*Scytalopus parvirostris*				2	9	2	8	0	10	0	13	3	13	2	13	7	19	4	16	5
Rhinocryptidae	*Scytalopus macropus*			x	2	0	0x	0	0x	2	0x	3	0x	2	0x	0	3x	4	5x	4	0x
Rhinocryptidae	*Scytalopus femoralis*				2	8	0	6	0	6	0	9	6	13	0	6	7	15	6	9	6
Rhinocryptidae	*Scytalopus micropterus*				2	13	12	19	9	15	13	27	13	17	15	29	15	40	14x	38	17x
Rhinocryptidae	*Scytalopus bolivianus*				2	18	5	11	0	18	1	17	4	23	5	22	7	28	6	24	9
Rhinocryptidae	*Scytalopus atratus*				2	8	4	12	4	6	4	17	5	15	6	18	8	24	11	25	7
Rhinocryptidae	*Scytalopus sanctaemartae*			x	2	31	0x	46	0x	77	0x	31	0x	77	0x	46	0x	77	0x	77	0x
Rhinocryptidae	*Scytalopus panamensis*	T		x	2	100	0x	0	+02x	0	+02x	100	0x	100	0x	0	+02x	0	+02x	100	0x
Rhinocryptidae	*Scytalopus vicinior*			x	2	5	7x	5	11x	7	11x	13	0x	9	7x	8	11x	9	11x	13	0x
Rhinocryptidae	*Scytalopus argentifrons*			x	2	20	11x	36	18x	16	18x	33	18x	23	11x	36	19x	45	19x	52	18x
Rhinocryptidae	*Scytalopus latebricola*			x	2	33	0x	51	0x	84	0x	33	0x	84	0x	51	0x	84	0x	84	0x
Rhinocryptidae	*Scytalopus meridanus*			x	2	23	0x	35	7x	16	7x	48	7x	41	5x	40	16x	46	21x	51	13x
Rhinocryptidae	*Scytalopus caracae*			x	2	37	0x	0	0x	37	0x	13	0x	37	0x	37	0x	37	0x	13	0x
Rhinocryptidae	*Scytalopus spillmani*				14	19	7x	18	7x	21	7x	36	3x	27	10x	40	19x	50	7x	36	10x
Rhinocryptidae	*Scytalopus zimmeri*			x	2	29	1x	24	3x	27	3x	30	1x	30	1x	25	6x	27	3x	32	1x
Rhinocryptidae	*Scytalopus simonsi*				2	14	14x	16	0	19	5	11	11	18	14x	20	11x	24	10x	15	16x
Rhinocryptidae	*Scytalopus iraiensis*	T		x	14	47	0x	1	0x	3	0x	47	0x	49	0x	50	0x	50	0x	100	0x
Rhinocryptidae	*Scytalopus urubambae*			x	14	0	0x	0	0x	0	0x	0	0x	0	0x	0	0x	0	0x	0	0x
Rhinocryptidae	*Scytalopus altirostris*			x	2	0	0x	0	0x	0	0x	2	0x	0	0x	0	2x	2	10x	2	0x
Rhinocryptidae	*Scytalopus affinis*			x	2	0	1x	11	1x	11	1x	11	1x	0	1x	11	1x	20	1x	20	1x
Rhinocryptidae	*Scytalopus canus*			x	2	26	2x	28	3x	17	4x	24	4x	29	7x	36	10x	42	12x	28	15x
Rhinocryptidae	*Scytalopus magellanicus*				2	7	13	6	12	7	13	6	12	9	20	10	22	13	23	12	22
Rhinocryptidae	*Scytalopus griseicollis*			x	2	15	0x	39	0x	39	0x	39	0x	15	0x	39	0x	39	0x	39	0x
Rhinocryptidae	*Scytalopus fuscicauda*			x	14	2	0x	2	0x	49	0x	49	0x	2	0x	2	0x	49	3x	49	0x
Rhinocryptidae	*Scytalopus superciliaris*				2	0	6	0	6	0	13	0	6	0	6	0	13	0	13	0	13
Rhinocryptidae	*Scytalopus fuscus*			x	2	2	0x	2	0x	1	0x	2	0x	3	10x	3	0x	4	0x	3	0x
Rhinocryptidae	*Scytalopus acutirostris*			x	2	0	0x	0	0x	0	0x	7	0x	0	0x	0	7x	7	11x	7	0x
Rhinocryptidae	*Scytalopus speluncae*				2	19	5	17	6	23	4	26	5	25	14	27	14	42	7	47	10
Rhinocryptidae	*Scytalopus novacapitalis*			x	2	3	54x	0	9x	1	32x	3	7x	5	59x	4	9x	4	32x	3	7x
Rhinocryptidae	*Scytalopus psychopompus*	T		x	14	0	0x	0	0x	0	0x	0	0x	44	0x	0	0x	0	0x	44	0x
Rhinocryptidae	*Scytalopus indigoticus*				2	17	10	15	10	27	6	24	9	22	18	23	19	39	10	38	14

Family	Species	IUCN	Ag	R	Range Source	2050 TG H	2050 TG C	2050 AM H	2050 AM C	2050 GO H	2050 GO C	2050 OS H	2050 OS C	2100 TG H	2100 TG C	2100 AM H	2100 AM C	2100 GO H	2100 GO C	2100 OS H	2100 OS C
Rhinocryptidae	*Scytalopus schulenbergi*				2	7	6	7	0	7	2	16	6	12	6	16	10	23	7	19	12
Rhinocryptidae	*Scytalopus chocoensis*			x	2	19	1x	4	11x	8	0x	4	11x	23	1x	4	11x	8	11x	8	11x
Rhinocryptidae	*Scytalopus robbinsi*			x	14	0	67x	0	0x	33	67x	33	0x	33	67x	33	0x	33	67x	33	0x
Rhinocryptidae	*Scytalopus parkeri*			x	2	58	28x	43	23x	0	51x	17	51x	58	28x	50	23x	28	62x	34	62x
Rhinocryptidae	*Acropternis orthonyx*				2	18	9	19	10	16	12	30	9	23	15	32	19	41	17	36	15
Climacteridae	*Cormobates placens*				8	6	0	5	0	10	0	8	0	9	11	13	14	15	13	26	6
Climacteridae	*Cormobates leucophaeus*				10	8	14	11	9	15	11	18	8	13	22	15	15	22	19	28	11
Climacteridae	*Climacteris affinis*				10	0	10	0	9	0	10	0	9	0	11	0	10	0	11	0	10
Climacteridae	*Climacteris erythrops*				10	10	8	16	5	19	4	21	4	18	11	20	6	27	6	33	5
Climacteridae	*Climacteris picumnus*				10	3	20	4	12	6	17	6	9	5	26	5	21	8	25	10	12
Climacteridae	*Climacteris melanura*				10	0	14	0	9	0	12	0	4	0	14	0	12	0	16	0	5
Climacteridae	*Climacteris rufa*				10	0	11	0	9	0	12	0	8	0	12	0	11	0	15	0	8
Menuridae	*Menura alberti*	T		x	10	0	4x	7	4x	7	4x	7	4x	0	4x	7	4x	7	4x	7	4x
Menuridae	*Menura novaehollandiae*				10	11	10	18	8	21	7	24	8	18	12	24	9	30	8	39	9
Menuridae	*Atrichornis rufescens*				10	18	0	36	0	32	0	35	0	18	0	43	0	36	0	61	0
Menuridae	*Atrichornis clamosus*	T		x	10	0	12x	0	0x	0	0x	0	0x	0	12x	0	0x	0	12x	0	0x
Ptilonorhynchida	*Ailuroedus buccoides*				8	5	2	6	2	16	2	15	1	11	4	13	5	26	4	24	2
Ptilonorhynchida	*Ailuroedus melanotis*				8,10	4	7	4	5	16	5	12	4	7	13	10	13	26	12	25	8
Ptilonorhynchida	*Ailuroedus crassirostris*				10	15	6	25	7	27	4	32	5	18	10	30	9	37	6	51	6
Ptilonorhynchida	*Scenopooetes dentirostris*				10	0	26x	0	20x	0	33x	4	22x	0	46x	0	39x	0	54x	4	43x
Ptilonorhynchida	*Archboldia papuensis*				8	8	0	4	0	15	0	17	0	15	4	16	0	34	0	33	0
Ptilonorhynchida	*Archboldia sanfordi*			x	8	15	0x	0	0x	20	0x	25	0x	22	7x	0	0x	48	0x	53	0x
Ptilonorhynchida	*Amblyornis inornatus*			x	8	8	0x	4	0x	0	0x	0	0x	8	0x	4	0x	4	0x	37	0x
Ptilonorhynchida	*Amblyornis macgregoriae*				8	7	1	4	1	22	1	13	0	10	5	10	7	39	6	30	3
Ptilonorhynchida	*Amblyornis subalaris*			x	8	0	0x	0	0x	14	0x	36	0x	16	25x	12	39x	34	39x	69	12x
Ptilonorhynchida	*Amblyornis flavifrons*			x	8	2	0x	0	0x	2	0x	62	0x	2	0x	63	0x	14	0x	92	0x
Ptilonorhynchida	*Prionodura newtoniana*			x	10	0	29x	0	23x	0	34x	5	23x	0	42x	0	41x	0	59x	5	47x
Ptilonorhynchida	*Sericulus aureus*				8	7	1	5	1	7	1	9	0	9	1	13	1	15	1	14	0
Ptilonorhynchida	*Sericulus bakeri*	T		x	8	0	78x	10	78x	15	78x	78	0x	0	78x	10	78x	17	78x	78	0x
Ptilonorhynchida	*Sericulus chrysocephalus*				10	15	7	25	7	26	6	31	6	18	9	28	8	36	6	50	6
Ptilonorhynchida	*Ptilonorhynchus violaceus*				10	10	10	15	6	19	7	22	6	18	15	20	9	27	11	34	9
Ptilonorhynchida	*Chlamydera guttata*				10	0	8	0	8	0	8	0	9	0	8	0	9	0	9	0	10
Ptilonorhynchida	*Chlamydera maculata*				10	0	22	0	16	0	19	0	14	0	27	0	25	0	28	1	16
Ptilonorhynchida	*Chlamydera nuchalis*				10	0	22	0	13	0	20	0	8	0	23	0	19	0	24	0	9
Ptilonorhynchida	*Chlamydera lauterbachi*			x	8	5	2x	4	0x	41	0x	20	2x	23	2x	9	2x	58	0x	33	2x
Ptilonorhynchida	*Chlamydera cerviniventris*				8,10	3	12	4	8	25	5	23	3	8	13	11	13	37	8	34	5
Maluridae	*Clytomyias insignis*				8	4	3	6	3	16	2	15	1	10	5	13	6	27	4	25	2
Maluridae	*Sipodotus wallacii*				8	4	2	7	2	9	1	14	2	10	4	15	4	17	3	23	2
Maluridae	*Malurus grayi*				8	6	1	7	1	6	1	8	2	12	2	16	2	13	1	16	2
Maluridae	*Malurus campbelli*			x	8	0	0x	1	0x	4	0x	32	0x	0	1x	24	0x	34	0x	55	0x
Maluridae	*Malurus alboscapulatus*			x	8	2	11x	5	8x	12	7x	16	8x	5	16x	11	16x	27	15x	27	11x
Maluridae	*Malurus melanocephalus*				10	0	21	0	12	1	18	1	7	0	25	0	22	1	26	2	10
Maluridae	*Malurus leucopterus*				10	0	8	0	7	0	8	0	7	0	8	0	8	0	9	0	8
Maluridae	*Malurus cyaneus*				10	3	20	4	11	6	16	6	7	6	27	5	19	9	25	10	10

Family	Species	IUCN	Ag	R	Range Source	Percentage loss in geographic range 2050								2100							
						TG		AM		GO		OS		TG		AM		GO		OS	
						H	C	H	C	H	C	H	C	H	C	H	C	H	C	H	C
Maluridae	*Malurus splendens*				10	0	8	0	8	0	8	0	7	0	9	0	8	0	9	0	7
Maluridae	*Malurus lamberti*				10	1	12	1	10	1	11	1	9	1	14	1	13	2	15	2	11
Maluridae	*Malurus amabilis*				10	0	29	0	19	0	30	1	12	0	36	0	32	0	37	1	16
Maluridae	*Malurus elegans*			x	10	0	49x	0	19x	0	31x	0	1x	0	65x	0	19x	0	49x	0	1x
Maluridae	*Malurus pulcherrimus*				10	0	7	0	6	0	7	0	6	0	7	0	10	0	11	0	7
Maluridae	*Malurus coronatus*				10	0	9	0	8	0	9	0	8	0	10	0	14	0	17	0	15
Maluridae	*Malurus cyanocephalus*				8	4	4	6	3	15	2	15	2	11	5	13	5	25	3	24	2
Maluridae	*Stipiturus ruficeps*				10	0	12	0	12	0	12	0	12	0	12	0	14	0	14	0	14
Maluridae	*Stipiturus malachurus*				10	7	16	11	6	12	12	14	4	11	17	14	6	19	13	22	4
Maluridae	*Stipiturus mallee*	T			10	0	8	0	0	0	8	0	0	0	12	0	3	0	12	0	3
Maluridae	*Amytornis barbatus*				10	0	0	0	0	0	0	0	0	0	0	0	2	0	2	0	2
Maluridae	*Amytornis woodwardi*	T		x	10	0	0x	0	0x	0	0x	0	0x	0	0x	0	0x	0	0x	0	0x
Maluridae	*Amytornis dorotheae*				10	0	9	0	9	0	9	0	9	0	9	0	13	0	9	0	9
Maluridae	*Amytornis striatus*				10	0	9	0	9	0	8	0	8	0	9	0	9	0	9	0	9
Maluridae	*Amytornis goyderi*				10	0	0	0	0	0	0	0	0	0	0	0	2	0	2	0	2
Maluridae	*Amytornis textilis*				10	0	0	0	0	0	0	0	0	0	0	0	0	0	0	0	0
Maluridae	*Amytornis purnelli*				10	0	13	0	13	0	11	0	11	0	13	0	13	0	13	0	13
Maluridae	*Amytornis housei*			x	10	0	89x	0	45x	0	78x	0	43x	0	89x	0	59x	0	89x	0	43x
Meliphagidae	*Myzomela blasii*			x	7	16	0x	19	0x	21	0x	21	0x	34	0x	19	0x	25	0x	44	0x
Meliphagidae	*Myzomela albigula*			x	8	48	0x	0	0x	32	0x	71	0x	57	0x	59	0x	32	0x	91	0x
Meliphagidae	*Myzomela eques*				8	4	2	6	2	17	2	16	1	11	3	13	3	28	3	25	1
Meliphagidae	*Myzomela cineracea*		H		8	0	0	0	0	0	0	0	0	0	0	0	0	0	0	0	0
Meliphagidae	*Myzomela obscura*				7,8,10	0	18	1	12	3	16	4	5	2	21	2	18	4	20	5	6
Meliphagidae	*Myzomela cruentata*				8	7	1	7	1	22	1	15	0	13	5	14	6	34	5	30	2
Meliphagidae	*Myzomela nigrita*				8	7	3	8	3	22	2	15	1	13	6	15	7	32	6	29	3
Meliphagidae	*Myzomela pulchella*			x	8	0	0x	1	0x	72	0x	71	0x	80	0x	2	0x	91	0x	72	0x
Meliphagidae	*Myzomela adolphinae*		H		8	0	1	0	1	0	1	0	0	0	6	0	11	0	10	0	4
Meliphagidae	*Myzomela kuehni*			x	7	23	19x	0	19x	0	19x	19	0x	23	19x	0	19x	0	19x	19	0x
Meliphagidae	*Myzomela dammermani*			x	7	0	0x	0	0x	0	0x	0	0x	0	18x	0	19x	0	18x	0	0x
Meliphagidae	*Myzomela erythrocephala*				8,10	1	22	1	11	3	20	6	7	3	22	3	17	4	23	7	7
Meliphagidae	*Myzomela chloroptera*				7	15	14	24	10	17	14	17	12	17	15	28	23	27	30	24	26
Meliphagidae	*Myzomela wakoloensis*				7	15	0	16	0	17	0	18	0	29	0	16	0	24	0	34	0
Meliphagidae	*Myzomela boiei*			x	7	18	19x	8	19x	23	2x	47	25x	18	19x	8	19x	33	2x	47	25x
Meliphagidae	*Myzomela sanguinolenta*				10	6	23	10	13	15	17	15	8	10	33	12	25	19	29	25	12
Meliphagidae	*Myzomela caledonica*			x	9	13	28x	34	10x	30	16x	27	9x	13	28x	51	11x	36	17x	33	9x
Meliphagidae	*Myzomela cardinalis*			x	9,11	11	9x	17	8x	28	7x	27	4x	30	9x	27	8x	36	7x	48	4x
Meliphagidae	*Myzomela sclateri*			x	8	0	0x	35	0x	35	0x	6	0x	0	0x	35	0x	35	0x	6	0x
Meliphagidae	*Myzomela pammelaena*			x	8	13	0x	1	0x	20	0x	41	0x	36	0x	1	0x	40	0x	45	0x
Meliphagidae	*Myzomela lafargei*			x	9	13	3x	12	0x	15	0x	23	0x	24	12x	30	0x	52	0x	36	0x
Meliphagidae	*Myzomela eichhorni*			x	9	30	19x	29	0x	22	10x	44	0x	30	19x	35	0x	37	10x	44	0x
Meliphagidae	*Myzomela melanocephala*			x	9	0	0x	9	0x	13	0x	34	0x	20	0x	9	0x	54	0x	71	0x
Meliphagidae	*Myzomela malaitae*			x	9	4	9x	0	9x	53	0x	0	9x	43	9x	0	9x	62	9x	13	9x
Meliphagidae	*Myzomela tristrami*			x	9	47	0x	34	0x	39	0x	76	0x	47	0x	34	0x	39	0x	99	0x
Meliphagidae	*Myzomela jugularis*				11	22	6	20	6	31	1	44	1	25	6	41	6	77	1	76	1

Family	Species	IUCN	Ag	R	Range Source	2050 TG H	2050 TG C	2050 AM H	2050 AM C	2050 GO H	2050 GO C	2050 OS H	2050 OS C	2100 TG H	2100 TG C	2100 AM H	2100 AM C	2100 GO H	2100 GO C	2100 OS H	2100 OS C
Meliphagidae	*Myzomela erythromelas*				8	8	0	9	0	45	0	49	0x	26	0	28	0	69	0x	61	0x
Meliphagidae	*Myzomela vulnerata*			x	7	0	40x	5	40x	19	26x	0	40x	0	40x	5	41x	19	26x	0	40x
Meliphagidae	*Myzomela rosenbergii*				8	6	1	4	1	19	1	13	0	10	6	12	8	33	7	31	3
Meliphagidae	*Certhionyx pectoralis*				10	0	20	0	13	0	19	0	7	0	22	0	18	0	22	0	9
Meliphagidae	*Certhionyx niger*				10	0	8	0	8	0	8	0	7	0	9	0	9	0	10	0	9
Meliphagidae	*Certhionyx variegatus*				10	0	9	0	8	0	9	0	8	0	9	0	9	0	10	0	9
Meliphagidae	*Timeliopsis fulvigula*				8	5	0	5	0	22	0	11	0	9	6	12	7	36	6	30	3
Meliphagidae	*Timeliopsis griseigula*				8	6	2	7	1	9	1	14	3	15	4	17	7	17	5	24	3
Meliphagidae	*Stresemannia bougainvillei*			x	9	16	0x	16	0x	1	0x	14	0x	17	0x	16	0x	34	0x	47	0x
Meliphagidae	*Glycichaera fallax*				8,10	4	3	5	3	16	2	15	2	10	5	13	5	26	4	23	2
Meliphagidae	*Lichmera lombokia*			x	7	16	8x	10	11x	4	11x	12	6x	16	28x	10	32x	20	16x	25	6x
Meliphagidae	*Lichmera argentauris*				7,8	14	0	17	0	21	0	19	0	29	0	18	0	30	0	33	0
Meliphagidae	*Lichmera limbata*				7	7	10	7	11	6	8	4	9	7	17	7	20	12	13	9	9
Meliphagidae	*Lichmera indistincta*				8,10	1	16	1	12	1	14	1	10	1	18	1	17	2	19	2	12
Meliphagidae	*Lichmera incana*				9	10	21	29	11	31	14	21	9	15	21	45	12	39	15	39	9
Meliphagidae	*Lichmera squamata*			x	7	18	22x	5	22x	16	10x	31	21x	18	22x	5	22x	23	10x	31	21x
Meliphagidae	*Lichmera alboauricularis*				8	8	3	9	0	40	0	43	3	29	11	22	8	49	5	53	3
Meliphagidae	*Lichmera deningeri*			x	7	9	0x	7	0x	9	0x	14	0x	15	0x	7	0x	22	0x	14	0x
Meliphagidae	*Lichmera monticola*			x	7	16	0x	19	0x	20	0x	20	0x	34	0x	19	0x	24	0x	43	0x
Meliphagidae	*Lichmera flavicans*			x	7	0	24x	5	24x	15	14x	0	24x	0	24x	5	24x	15	14x	0	24x
Meliphagidae	*Lichmera notabilis*			x	7	25	18x	0	18x	0	18x	18	0x	25	18x	0	18x	0	18x	18	0x
Meliphagidae	*Trichodere cockerelli*			x	10	0	43x	0	30x	0	43x	0	4x	0	44x	0	43x	0	43x	0	4x
Meliphagidae	*Meliphaga montana*				8	7	2	9	2	17	2	13	0	11	3	17	3	23	2	28	2
Meliphagidae	*Meliphaga mimikae*				8	8	0	14	0	15	0	17	0	14	8	22	11	32	11	27	2
Meliphagidae	*Meliphaga orientalis*				8	7	1	6	1	19	1	12	0	11	5	13	7	30	6	28	2
Meliphagidae	*Meliphaga albonotata*				8	3	1	4	1	18	1	19	0	7	5	10	7	33	6	27	2
Meliphagidae	*Meliphaga aruensis*				8	4	3	6	2	16	2	15	1	11	4	13	5	27	4	24	2
Meliphagidae	*Meliphaga analoga*				8	4	3	6	2	15	2	15	1	11	5	13	5	26	4	23	2
Meliphagidae	*Meliphaga vicina*			x	8	45	0x	0	0x	0	0x	85	0x	60	0x	85	0x	0	0x	85	0x
Meliphagidae	*Meliphaga gracilis*				8,10	0	19	1	11	10	16	13	5	2	23	5	21	16	21	17	9
Meliphagidae	*Meliphaga notata*		H		10	0	28	0	17	0	29	0	10	0	34	0	28	0	35	0	15
Meliphagidae	*Meliphaga flavirictus*				8	5	2	6	2	13	1	13	1	10	4	13	4	22	3	21	1
Meliphagidae	*Meliphaga lewinii*				10	8	18	13	13	17	13	19	10	12	25	17	20	24	22	31	13
Meliphagidae	*Meliphaga albilineata*				10	0	50	0	22	0	41	0	11	0	50	0	27	0	50	0	11
Meliphagidae	*Meliphaga reticulata*			x	7	0	39x	9	39x	20	27x	0	39x	0	39x	9	39x	20	27x	0	39x
Meliphagidae	*Guadalcanaria inexpectata*			x	9	0	0x	0	0x	16	0x	30	0x	23	0x	0	0x	53	0x	77	0x
Meliphagidae	*Foulehaio carunculata*				11	16	4	14	4	21	0	32	0	26	4	30	4	60	0	59	0
Meliphagidae	*Lichenostomus subfrenatus*				8	8	0	3	0	19	0	13	0	12	6	13	8	31	7	34	4
Meliphagidae	*Lichenostomus obscurus*				8	6	2	6	1	13	1	12	1	11	4	13	5	24	4	21	2
Meliphagidae	*Lichenostomus frenatus*				10	0	31	0	25	0	35	3	27	0	49	0	43	0	57x	3	45
Meliphagidae	*Lichenostomus hindwoodi*			x	10	0	63x	0	63x	4	63x	4	63x	0	63x	0	81x	4	63x	4	63x
Meliphagidae	*Lichenostomus chrysops*				10	5	23	7	14	10	17	11	10	9	32	9	24	14	28	18	14
Meliphagidae	*Lichenostomus versicolor*		H		8,10	0	10	0	7	0	6	0	3	0	13	0	11	0	10	0	4
Meliphagidae	*Lichenostomus fasciogulari*				10	3	24	7	12	16	16	21	6	7	29	7	18	25	26	28	7

Family	Species	IUCN	Ag	R	Range Source	Percentage loss in geographic range															
						2050								2100							
						TG		AM		GO		OS		TG		AM		GO		OS	
						H	C	H	C	H	C	H	C	H	C	H	C	H	C	H	C
Meliphagidae	*Lichenostomus virescens*				10	0	11	0	9	0	10	0	7	0	12	0	11	0	13	0	9
Meliphagidae	*Lichenostomus flavus*				10	0	35	0	20	0	34	1	13	0	38	0	31	0	38	1	15
Meliphagidae	*Lichenostomus unicolor*				10	0	22	0	13	0	22	0	8	0	24	0	20	0	25	0	10
Meliphagidae	*Lichenostomus leucotis*				10	3	16	4	10	6	13	6	8	5	22	5	19	8	22	10	11
Meliphagidae	*Lichenostomus flavicollis*				10	0	43	0	13	5	36	1	13	2	43	4	13	9	36	8	13
Meliphagidae	*Lichenostomus melanops*				10	5	12	8	7	10	8	12	5	12	19	10	12	16	15	19	6
Meliphagidae	*Lichenostomus cratitius*				10	0	13	0	10	0	12	0	10	0	13	0	12	0	14	0	11
Meliphagidae	*Lichenostomus keartlandi*				10	0	15	0	15	0	15	0	14	0	16	0	17	0	19	0	18
Meliphagidae	*Lichenostomus flavescens*				8,10	0	16	0	10	0	14	1	5	0	16	0	13	0	18	1	6
Meliphagidae	*Lichenostomus fuscus*				10	7	12	12	9	15	10	17	7	13	21	14	17	21	19	28	11
Meliphagidae	*Lichenostomus plumulus*				10	0	11	0	10	0	10	0	9	0	12	0	13	0	13	0	10
Meliphagidae	*Lichenostomus ornatus*				10	0	12	0	9	0	12	0	8	1	14	0	12	1	18	0	9
Meliphagidae	*Lichenostomus penicillatus*				10	2	15	2	10	3	13	4	8	4	19	3	14	5	18	6	9
Meliphagidae	*Xanthotis flaviventer*		H		8,10	0	4	0	3	0	3	0	1	0	5	0	5	0	4	0	2
Meliphagidae	*Xanthotis polygramma*				8	3	5	6	3	15	2	12	2	8	7	12	7	25	5	22	3
Meliphagidae	*Xanthotis macleayana*				10	0	33	0	20	0	38	3	24	0	50	0	44	0	57	3	41
Meliphagidae	*Xanthotis provocator*			x	11	0	36x	0	36x	64	36x	64	36x	0	36x	0	36x	64	36x	64	36x
Meliphagidae	*Oreornis chrysogenys*			x	8	0	0x	0	0x	0	0x	0	0x	0	0x	23	0x	0	0x	1	0x
Meliphagidae	*Melithreptus lunatus*				10	6	16	9	8	12	12	14	5	11	23	11	15	17	20	21	8
Meliphagidae	*Melithreptus affinis*				10	1	43	0	14	6	36	1	14	3	44	6	14	9	36	10	14
Meliphagidae	*Melithreptus albogularis*				8,10	1	23	1	14	7	19	8	8	2	27	3	25	9	28	11	11
Meliphagidae	*Melithreptus gularis*				10	2	19	3	11	4	15	4	7	3	23	3	19	6	23	7	9
Meliphagidae	*Melithreptus validirostris*				10	0	39	0	15	5	35	1	15	2	40	4	15	6	35	8	15
Meliphagidae	*Melithreptus brevirostris*				10	2	13	4	10	5	11	5	8	5	17	5	13	7	16	9	9
Meliphagidae	*Notiomystis cincta*	T		x	12,13	0	0x	0	0x	38	0x	38	0x	0	0x	0	0x	38	0x	38	0x
Meliphagidae	*Pycnopygius ixoides*				8	4	2	6	1	16	1	14	1	11	3	13	4	26	3	23	2
Meliphagidae	*Pycnopygius cinereus*		H		8	0	0	0	0	0	0	0	0	0	6	0	7	0	6	0	3
Meliphagidae	*Pycnopygius stictocephalu*		H		8	0	5	0	4	0	2	0	2	0	6	0	6	0	4	0	3
Meliphagidae	*Melitograis gilolensis*				7	18	0x	26	0x	22	0x	21	0x	35	0x	26	0x	41	0x	40	0x
Meliphagidae	*Philemon meyeri*				8	3	3	6	2	19	1	19	1	11	4	14	4	31	3	28	1
Meliphagidae	*Philemon inornatus*			x	7	0	40x	6	40x	19	27x	0	40x	0	40x	6	40x	19	27x	0	40x
Meliphagidae	*Philemon kisserensis*			x	7	0	35x	0	35x	0	35x	0	35x	0	35x	0	35x	0	35x	0	35x
Meliphagidae	*Philemon brassi*			x	8	18	18x	45	0x	18	0x	34	18x	26	18x	48	18x	18	0x	36	18x
Meliphagidae	*Philemon citreogularis*				8,10	0	19	1	13	2	17	2	9	1	23	1	20	2	23	3	11
Meliphagidae	*Philemon fuscicapillus*	T			7	29	0	26	0	23	0	34	0	42	0	33	0	47	0	47	0
Meliphagidae	*Philemon moluccensis*			x	7	8	4x	7	4x	11	1x	24	5x	14	4x	7	4x	23	1x	24	5x
Meliphagidae	*Philemon subcorniculatus*			x	7	15	0x	17	0x	20	0x	20	0x	33	0x	17	0x	24	0x	43	0x
Meliphagidae	*Philemon buceroides*				7,8,10	3	9	5	7	14	8	14	4	9	12	10	12	22	11	21	5
Meliphagidae	*Philemon novaeguineae*				8	4	4	6	3	18	2	18	2	11	5	13	6	30	4	27	3
Meliphagidae	*Philemon albitorques*			x	8	37	0x	0	0x	59	0x	90	0x	90	0x	0	0x	97	0x	90	0x
Meliphagidae	*Philemon cockerelli*		H		8	0	0	0	0	0	0	0	0	0	0	0	0	0	0	0	0
Meliphagidae	*Philemon eichhorni*			x	8	0	0x	5	0x	84	0x	67	0x	67	0x	5	0x	97	0x	75	0x
Meliphagidae	*Philemon argenticeps*				10	0	18	0	12	0	17	0	6	0	19	0	16	0	20	0	7
Meliphagidae	*Philemon corniculatus*				8,10	0	20	0	12	1	17	1	8	2	25	1	19	2	25	2	10

Family	Species	IUCN	Ag	R	Range Source	2050 TG H	2050 TG C	2050 AM H	2050 AM C	2050 GO H	2050 GO C	2050 OS H	2050 OS C	2100 TG H	2100 TG C	2100 AM H	2100 AM C	2100 GO H	2100 GO C	2100 OS H	2100 OS C
						Percentage loss in geographic range															
Meliphagidae	*Philemon diemenensis*			x	9	13	28x	34	10x	30	16x	26	9x	13	28x	51	11x	35	17x	33	9x
Meliphagidae	*Ptiloprora plumbea*				8	6	0	10	0	25	0	16	0	15	13	20	13	32	13	35	4
Meliphagidae	*Ptiloprora meekiana*				8	5	0	4	0	19	0	15	0	11	13	11	20	30	20	38	7
Meliphagidae	*Ptiloprora erythropleura*				8	4	0	7	0	4	0	5	0	7	0	24	0	8	0	17	0
Meliphagidae	*Ptiloprora mayri*			x	8	1	0x	0	0x	1	0x	34	0x	2	2x	34	0x	8	0x	46	0x
Meliphagidae	*Ptiloprora guisei*				8	7	2	3	1	32	1	19	0	11	10	4	17	51	15	44	7
Meliphagidae	*Ptiloprora perstriata*				8	7	0	2	0	15	0	12	0	13	7	14	8	27	8	26	5
Meliphagidae	*Melidectes fuscus*				8	10	0	2	0	18	0	17	0	16	8	9	11	34	10	37	5
Meliphagidae	*Melidectes whitemanensis*			x	8	0	0x	0	0x	62	0x	22	0x	13	0x	10	0x	82	0x	42	0x
Meliphagidae	*Melidectes nouhuysi*			x	8	0	0x	0	0x	13	0x	10	0x	10	0x	18	0x	16	0x	10	0x
Meliphagidae	*Melidectes princeps*	T		x	8	27	0x	0	0x	6	0x	38	0x	29	0x	0	0x	40	0x	82	0x
Meliphagidae	*Melidectes ochromelas*				8	7	0	7	0	12	0	12	0	13	5	19	7	19	7	32	3
Meliphagidae	*Melidectes leucostephes*			x	8	24	0x	5	0x	7	0x	0	0x	26	0x	5	0x	7	0x	38	0x
Meliphagidae	*Melidectes belfordi*				8	6	0	2	0	19	0	12	0	11	7	11	9	34	8	28	4
Meliphagidae	*Melidectes rufocrissalis*		H		8	0	0	0	0	0	0	0	0	0	7	0	6	0	4	0	1
Meliphagidae	*Melidectes foersteri*			x	8	0	5x	28	0x	50	0x	28	0x	0	5x	28	0x	50	0x	53	0x
Meliphagidae	*Melidectes torquatus*		H		8	0	0	0	0	0	0	0	0	0	6	0	7	0	6	0	3
Meliphagidae	*Melidectes sclateri*			x	9	48	0x	33	0x	40	0x	77	0x	48	0x	33	0x	40	0x	99	0x
Meliphagidae	*Melipotes gymnops*			x	8	18	0x	4	0x	5	0x	0	0x	19	0x	5	0x	6	0x	50	0x
Meliphagidae	*Melipotes fumigatus*		H		8	0	0	0	0	0	0	0	0	0	7	0	8	0	8	0	4
Meliphagidae	*Melipotes ater*			x	8	0	5x	25	0x	49	0x	27	0x	0	5x	25	0x	49	0x	53	0x
Meliphagidae	*Myza celebensis*				7	15	13	26	9	19	13	16	11	17	14	29	22	29	31	24	27
Meliphagidae	*Myza sarasinorum*				7	14	13	26	9	19	13	16	11	17	14	29	22	28	31	24	27
Meliphagidae	*Gymnomyza viridis*	T			11	27	4	20	4	30	0	42	0	28	4	39	4	74	0	73	0
Meliphagidae	*Gymnomyza samoensis*	T		x	11	0	0x	0	0x	1	0x	0	0x	45	0x	0	0x	1	0x	1	0x
Meliphagidae	*Gymnomyza aubryana*	T		x	9	12	27x	44	8x	21	17x	16	14x	12	27x	56	9x	23	18x	18	14x
Meliphagidae	*Phylidonyris pyrrhoptera*				10	7	19	11	7	15	15	17	7	16	21	16	7	22	15	27	7
Meliphagidae	*Phylidonyris novaehollandi*				10	2	20	2	9	3	16	4	4	6	22	3	10	7	18	6	5
Meliphagidae	*Phylidonyris nigra*				10	2	9	3	5	5	6	5	3	3	11	3	7	6	9	7	4
Meliphagidae	*Phylidonyris albifrons*				10	0	6	0	6	0	6	0	6	0	7	0	7	0	8	0	7
Meliphagidae	*Phylidonyris undulata*			x	9	12	27x	44	8x	21	17x	16	14x	12	27x	56	9x	23	18x	18	14x
Meliphagidae	*Phylidonyris notabilis*			x	9	4	0x	14	0x	30	0x	9	0x	20	0x	29	0x	43	0x	40	0x
Meliphagidae	*Phylidonyris melanops*				10	1	17	1	9	1	15	2	5	3	19	1	9	3	16	3	5
Meliphagidae	*Ramsayornis modestus*				8,10	2	17	4	13	12	16	13	7	6	20	8	19	17	19	16	8
Meliphagidae	*Ramsayornis fasciatus*				10	0	18	0	13	0	17	0	8	0	19	0	17	0	21	0	9
Meliphagidae	*Plectorhyncha lanceolata*				10	1	19	2	14	3	16	3	11	2	25	2	22	4	24	5	13
Meliphagidae	*Conopophila albogularis*		H		8,10	0	13	0	10	0	12	0	3	0	14	0	14	0	13	0	3
Meliphagidae	*Conopophila rufogularis*				10	0	22	0	13	0	20	1	8	0	23	0	20	0	25	1	10
Meliphagidae	*Conopophila whitei*				10	0	5	0	5	0	4	0	4	0	5	0	5	0	5	0	5
Meliphagidae	*Grantiella picta*				10	0	20	0	14	1	17	1	11	1	25	0	21	1	25	1	14
Meliphagidae	*Xanthomyza phrygia*	T			10	9	8	16	5	17	5	16	5	17	13	20	6	25	7	28	6
Meliphagidae	*Acanthorhynchus tenuirostr*				10	5	16	7	7	10	12	12	6	11	21	9	9	16	16	18	7
Meliphagidae	*Acanthorhynchus supercilio*				10	0	28	0	16	0	24	0	5	0	32	0	16	0	28	0	5
Meliphagidae	*Entomyzon cyanotis*				8,10	1	20	2	13	3	17	3	10	2	24	2	20	4	24	5	12

Family	Species	IUCN	Ag	R	Range Source	Percentage loss in geographic range															
						2050								2100							
						TG		AM		GO		OS		TG		AM		GO		OS	
						H	C	H	C	H	C	H	C	H	C	H	C	H	C	H	C
Meliphagidae	*Manorina melanophrys*				10	13	8	21	6	26	4	29	5	20	10	27	7	37	5	45	6
Meliphagidae	*Manorina melanocephala*				10	3	20	4	10	6	17	6	7	6	27	5	19	9	26	10	9
Meliphagidae	*Manorina melanotis*	T			10	0	32	0	24	0	32	0	24	0	37	0	29	0	37	0	29
Meliphagidae	*Anthornis melanura*		H		12,13	0	21	0	13	0	18	0	10	0	25	0	18	0	24	0	14
Meliphagidae	*Acanthagenys rufogularis*				10	0	10	0	9	0	9	0	7	0	12	0	11	0	12	1	9
Meliphagidae	*Anthochaera chrysoptera*				10	7	16	10	8	13	13	14	6	12	19	12	10	18	15	22	6
Meliphagidae	*Anthochaera carunculata*				10	3	13	5	8	6	12	7	5	7	15	6	9	9	14	11	6
Meliphagidae	*Anthochaera paradoxa*				10	0	51	1	15	5	43	2	15	4	52	6	15	9	43	6	15
Meliphagidae	*Prosthemadera novaeseela*				12,13	5	23	6	20	17	19	15	15	7	28	10	29	17	30	22	22
Meliphagidae	*Epthianura tricolor*		H		10	0	7	0	7	0	7	0	7	0	8	0	8	0	9	0	8
Meliphagidae	*Epthianura aurifrons*				10	0	7	0	6	0	7	0	6	0	7	0	7	0	8	0	7
Meliphagidae	*Epthianura crocea*				10	0	14	0	13	0	14	0	11	0	14	0	15	0	16	0	13
Meliphagidae	*Epthianura albifrons*		H		10	0	10	0	7	0	9	0	5	0	12	0	8	0	12	0	6
Meliphagidae	*Ashbyia lovensis*				10	0	0	0	0	0	0	0	0	0	0	0	1	0	1	0	1
Pardalotidae	*Pardalotus punctatus*				10	3	15	4	8	6	12	6	7	5	21	5	16	9	21	11	9
Pardalotidae	*Pardalotus quadragintus*	T		x	10	0	55x	0	3x	0	55x	0	3x	2	55x	0	3x	0	55x	0	3x
Pardalotidae	*Pardalotus rubricatus*				10	0	13	0	10	0	12	0	9	0	14	0	13	0	14	0	10
Pardalotidae	*Pardalotus striatus*				10	1	14	1	10	1	12	1	8	1	16	1	13	2	16	2	9
Pardalotidae	*Dasyornis longirostris*	T		x	10	0	0x	0	0x	0	0x	0	0x	0	0x	0	0x	0	0x	0	0x
Pardalotidae	*Dasyornis brachypterus*	T		x	10	0	0x	13	0x	3	0x	9	0x	0	0x	13	0x	3	0x	13	0x
Pardalotidae	*Dasyornis broadbenti*			x	10	13	26x	3	0x	3	35x	3	0x	27	26x	3	0x	3	35x	3	0x
Pardalotidae	*Pycnoptilus floccosus*				10	10	10	15	7	22	5	27	7	17	15	21	10	33	7	39	10
Pardalotidae	*Origma solitaria*				10	20	15	37	12	26	7	37	12	20	15	38	12	47	9	51	12
Pardalotidae	*Oreoscopus gutturalis*			x	10	0	34x	0	26x	0	37x	5	23x	0	49x	0	43x	0	57x	5	44x
Pardalotidae	*Crateroscelis murina*				8	4	2	6	1	17	1	14	1	10	4	13	5	29	4	25	2
Pardalotidae	*Crateroscelis nigrorufa*				8	4	0	4	0	23	0	11	0	9	7	11	8	39	7	28	3
Pardalotidae	*Crateroscelis robusta*				8	6	0	3	0	19	0	12	0	11	6	11	8	32	7	31	3
Pardalotidae	*Sericornis citreogularis*				10	14	13	21	11	22	11	26	11	16	16	26	15	33	15	43	15
Pardalotidae	*Sericornis frontalis*				10	4	16	5	9	9	12	9	7	7	22	7	17	12	21	15	9
Pardalotidae	*Sericornis humilis*				10	0	40	0	14	5	34	1	14	2	40	4	14	8	34	8	14
Pardalotidae	*Sericornis keri*			x	10	0	33x	0	33x	0	40x	7	33x	0	48x	0	52x	0	54x	7	47x
Pardalotidae	*Sericornis beccarii*				8,10	1	19	3	14	13	12	10	4	3	20	5	20	19	15	15	7
Pardalotidae	*Sericornis virgatus*				8	10	0	12	0	9	0	12	0	15	2	20	0	18	0	25	0
Pardalotidae	*Sericornis nouhuysi*				8	9	0	3	0	18	0	13	0	13	6	11	8	29	8	32	4
Pardalotidae	*Sericornis magnirostris*				10	11	13	19	11	25	9	29	9	15	19	24	15	35	15	44	13
Pardalotidae	*Sericornis rufescens*			x	8	25	0x	5	0x	7	0x	0	0x	27	0x	5	0x	7	0x	40	0x
Pardalotidae	*Sericornis perspicillatus*				8	6	1	3	0	21	0	15	0	10	7	12	9	35	8	32	4
Pardalotidae	*Sericornis arfakianus*				8	7	1	7	1	21	1	13	0	12	6	12	7	35	6	30	3
Pardalotidae	*Sericornis papuensis*				8	8	0	1	0	22	0	14	0	13	7	13	9	36	9	32	4
Pardalotidae	*Sericornis spilodera*				8	5	2	7	2	17	2	15	2	11	5	13	6	29	5	25	2
Pardalotidae	*Acanthornis magnus*				10	0	37	0	17	6	33	1	17	0	37	6	17	7	33	9	17
Pardalotidae	*Pyrrholaemus brunneus*				10	0	5	0	5	0	5	0	5	0	5	0	5	0	5	0	5
Pardalotidae	*Chthonicola sagittatus*				10	4	20	6	10	7	14	7	7	8	30	7	23	11	27	13	10
Pardalotidae	*Calamanthus campestris*				10	0	8	0	6	0	7	0	5	0	8	0	8	0	10	0	6

Family	Species	IUCN	Ag	R	Range Source	Percentage loss in geographic range 2050 TG H	TG C	AM H	AM C	GO H	GO C	OS H	OS C	2100 TG H	TG C	AM H	AM C	GO H	GO C	OS H	OS C
Pardalotidae	*Calamanthus fuliginosus*				10	7	24	7	6	15	20	15	5	13	25	13	6	20	20	21	5
Pardalotidae	*Hylacola pyrrhopygia*				10	2	11	2	3	2	8	5	1	10	15	4	3	9	9	6	1
Pardalotidae	*Hylacola cautus*				10	0	10	0	8	0	11	0	8	0	11	0	9	0	12	1	9
Pardalotidae	*Acanthiza murina*				8	9	0	2	0	18	0	15	0	14	7	13	9	31	9	31	5
Pardalotidae	*Acanthiza katherina*				10	0	29x	0	22x	0	35x	4	24x	0	46x	0	41x	0	57x	4	46x
Pardalotidae	*Acanthiza pusilla*				10	1	12	1	9	2	10	2	8	2	15	2	13	3	14	4	9
Pardalotidae	*Acanthiza pusilla*				10	1	12	1	9	2	10	2	8	2	15	2	13	3	14	4	9
Pardalotidae	*Acanthiza ewingii*				10	0	37	0	15	5	32	1	15	2	38	4	15	8	32	8	15
Pardalotidae	*Acanthiza reguloides*				10	3	18	4	9	6	14	7	5	6	25	5	18	9	24	11	7
Pardalotidae	*Acanthiza inornata*				10	0	35	0	22	0	31	0	7	0	41	0	22	0	35	0	7
Pardalotidae	*Acanthiza iredalei*				10	0	2	0	2	0	2	0	3	0	2	0	3	0	3	0	3
Pardalotidae	*Acanthiza chrysorrhoa*				10	1	12	1	9	2	11	2	8	2	14	2	12	3	14	3	9
Pardalotidae	*Acanthiza uropygialis*				10	0	7	0	6	0	7	0	6	0	7	0	7	0	8	0	6
Pardalotidae	*Acanthiza nana*		H		10	0	17	0	11	0	14	0	7	0	23	0	18	0	22	0	10
Pardalotidae	*Acanthiza lineata*				10	7	11	10	6	14	8	16	4	13	18	13	9	20	12	25	5
Pardalotidae	*Acanthiza robustirostris*				10	0	5	0	5	0	5	0	5	0	5	0	5	0	5	0	5
Pardalotidae	*Smicrornis brevirostris*				10	1	17	2	11	3	15	3	8	2	20	2	17	4	21	5	9
Pardalotidae	*Gerygone cinerea*				8	6	0	3	0	16	0	12	0	11	6	12	8	30	8	31	4
Pardalotidae	*Gerygone chloronotus*				8,10	3	11	4	6	9	9	9	3	6	12	9	10	16	13	15	4
Pardalotidae	*Gerygone palpebrosa*				8,10	3	10	4	7	12	9	12	4	7	13	9	12	20	12	19	6
Pardalotidae	*Gerygone olivacea*				8,10	2	22	2	12	4	18	4	7	3	27	3	21	5	26	7	9
Pardalotidae	*Gerygone chrysogaster*				8	4	3	6	3	16	2	15	2	10	5	13	5	26	4	24	2
Pardalotidae	*Gerygone magnirostris*				8,10	3	14	4	9	11	13	11	5	7	17	9	14	18	16	17	6
Pardalotidae	*Gerygone hypoxantha*	T		x	8	0	5x	0	5x	0	5x	0	5x	0	5x	0	5x	0	5x	0	5x
Pardalotidae	*Gerygone tenebrosa*				10	0	4	0	4	0	4	0	4	0	4	0	4	0	4	0	4
Pardalotidae	*Gerygone sulphurea*		H		7,8	0	8	0	7	0	8	0	8	0	10	0	10	0	12	0	11
Pardalotidae	*Gerygone inornata*			x	7	2	27x	5	27x	12	20x	4	24x	2	27x	5	28x	12	21x	4	24x
Pardalotidae	*Gerygone dorsalis*			x	7	19	16x	9	16x	17	6x	44	23x	19	16x	9	16x	24	6x	44	23x
Pardalotidae	*Gerygone ruficollis*				8	6	0	4	0	20	0	12	0	11	6	12	8	34	7	32	4
Pardalotidae	*Gerygone levigaster*				8,10	1	26	2	12	4	21	6	5	3	28	3	19	5	28	7	7
Pardalotidae	*Gerygone fusca*				10	0	17	0	12	1	14	1	10	1	19	1	17	1	19	1	13
Pardalotidae	*Gerygone mouki*				10	10	16	16	12	22	12	24	9	12	20	19	17	29	17	37	12
Pardalotidae	*Gerygone igata*				12,13	4	19	4	13	11	15	10	10	7	22	7	16	12	20	13	13
Pardalotidae	*Gerygone albofrontata*			x	12,13	0	0x	0	0x	0	0x	0	0x	0	0x	0	0x	0	0x	0	0x
Pardalotidae	*Gerygone flavolateralis*			x	9	8	11x	25	3x	30	7x	16	7x	22	11x	38	4x	38	8x	35	7x
Pardalotidae	*Aphelocephala leucopsis*				10	0	6	0	5	0	6	0	4	0	7	0	6	0	7	0	5
Pardalotidae	*Aphelocephala pectoralis*				10	0	0	0	0	0	0	0	0	0	0	0	0	0	0	0	0
Pardalotidae	*Aphelocephala nigricincta*				10	0	2	0	2	0	2	0	2	0	2	0	3	0	3	0	3
Petroicidae	*Amalocichla sclateriana*			x	8	0	0x	1	0x	0	0x	3	0x	0	7x	20	18x	1	18x	10	11x
Petroicidae	*Amalocichla incerta*				8	6	0	2	0	20	0	13	0	10	7	11	9	35	8	30	3
Petroicidae	*Monachella muelleriana*				8	5	2	7	1	20	1	17	1	11	5	13	6	35	4	29	3
Petroicidae	*Microeca fascinans*				8,10	1	15	1	10	1	13	2	8	2	17	1	14	2	17	3	9
Petroicidae	*Microeca hemixantha*			x	13	7	22x	7	22x	27	2x	54	29x	7	22x	7	22x	39	2x	54	29x
Petroicidae	*Microeca flavigaster*				8,10	0	22	1	12	2	21	3	7	1	24	1	19	3	24	4	8

Family	Species	IUCN	Ag	R	Range Source	Percentage loss in geographic range 2050 TG H	TG C	AM H	AM C	GO H	GO C	OS H	OS C	2100 TG H	TG C	AM H	AM C	GO H	GO C	OS H	OS C
Petroicidae	*Microeca griseoceps*				8,10	3	6	2	5	18	4	16	0	7	13	6	16	26	13	33	4
Petroicidae	*Microeca flavovirescens*				8	4	2	6	2	16	2	15	1	11	4	13	5	26	4	24	2
Petroicidae	*Microeca papuana*				8	7	0	4	0	19	0	13	0	11	6	13	8	31	8	32	4
Petroicidae	*Eugerygone rubra*				8	6	0	4	0	19	0	14	0	11	6	14	8	31	8	32	4
Petroicidae	*Petroica bivittata*			x	8	12	0x	1	0x	6	0x	16	0x	13	5x	13	10x	21	10x	38	8x
Petroicidae	*Petroica archboldi*			x	8	0	0x	0	0x	0	0x	0	0x	0	0x	21	0x	1	0x	0	0x
Petroicidae	*Petroica multicolor*				9,10,11	6	16	8	6	10	12	11	4	13	19	11	7	18	14	20	4
Petroicidae	*Petroica macrocephala*				12,13	4	21	5	16	13	15	11	12	7	26	8	22	14	23	15	18
Petroicidae	*Petroica goodenovii*				10	0	10	0	8	1	9	1	7	1	12	1	10	1	12	1	8
Petroicidae	*Petroica phoenicea*				10	7	14	10	4	12	10	12	4	16	18	14	5	19	11	21	5
Petroicidae	*Petroica rosea*				10	12	10	18	7	22	7	26	6	19	13	23	8	32	8	41	7
Petroicidae	*Petroica rodinogaster*				10	3	20	3	8	11	16	8	8	8	24	10	9	14	17	15	9
Petroicidae	*Petroica australis*				12,13	6	27	6	26	17	24	14	22	7	34	8	38	17	39	20	32
Petroicidae	*Petroica traversi*	T		x	12,13	0	0x	0	0x	0	0x	0	0x	0	0x	0	0x	0	0x	0	0x
Petroicidae	*Melanodryas cucullata*				10	0	8	0	7	1	8	1	6	1	9	1	8	1	9	1	7
Petroicidae	*Melanodryas vittata*				10	0	41	0	14	5	35	1	14	2	41	4	14	8	35	8	14
Petroicidae	*Tregellasia capito*				10	9	10	16	8	17	11	24	9	11	16	19	14	26	19	39	15
Petroicidae	*Tregellasia leucops*				8,10	5	2	3	1	17	2	8	0	9	6	8	7	28	7	23	2
Petroicidae	*Eopsaltria australis*				10	4	16	5	10	7	13	8	7	8	23	7	16	11	22	13	10
Petroicidae	*Eopsaltria griseogularis*				10	0	11	0	8	0	12	0	7	0	12	0	11	0	15	0	8
Petroicidae	*Eopsaltria flaviventris*			x	9	17	30x	40	9x	27	20x	25	13x	17	30x	56	11x	32	22x	29	13x
Petroicidae	*Eopsaltria georgiana*				10	0	32	0	16	0	26	0	4	0	38	0	16	0	32	0	4
Petroicidae	*Eopsaltria pulverulenta*				8,10	1	32	1	18	4	30	6	9	3	34	3	26	5	34	7	10
Petroicidae	*Poecilodryas brachyura*				8	6	2	8	1	12	1	9	3	15	3	16	3	21	1	22	3
Petroicidae	*Poecilodryas hypoleuca*				8	4	2	6	2	14	2	14	1	11	3	13	4	24	2	22	2
Petroicidae	*Poecilodryas superciliosa*				10	0	23	0	15	0	22	1	8	0	24	0	19	0	26	1	10
Petroicidae	*Poecilodryas placens*				8	6	0	0	0	20	0	18	0	6	8	0	11	37	11	29	3
Petroicidae	*Poecilodryas albonotata*				8	6	0	3	0	20	0	13	0	11	6	12	7	34	7	31	3
Petroicidae	*Peneothello sigillatus*				8	8	0	2	0	19	0	16	0	12	7	12	10	34	9	30	4
Petroicidae	*Peneothello cryptoleucus*				8	0	0	3	0	5	0	6	0	4	0	23	0	11	0	22	0
Petroicidae	*Peneothello cyanus*				8	6	0	4	0	21	0	13	0	11	6	13	7	35	7	33	4
Petroicidae	*Peneothello bimaculatus*				8	7	2	8	2	13	2	15	1	12	6	15	7	23	7	26	2
Petroicidae	*Heteromyias albispecularis*				8,10	3	11	3	9	12	14	8	10	6	23	8	21	20	26	20	20
Petroicidae	*Pachycephalopsis hattame*				8	10	0	10	0	4	0	7	1	14	0	22	0	6	1	18	1
Petroicidae	*Pachycephalopsis polioso*				8	5	0	3	0	21	0	13	0	9	7	11	9	37	8	27	3
Petroicidae	*Drymodes superciliaris*				8,10	4	4	6	3	14	3	14	2	9	6	12	7	23	5	22	2
Petroicidae	*Drymodes brunneopygia*				10	0	14	0	9	0	14	0	7	0	15	0	11	0	15	1	8
Irenidae	*Irena puella*				7	19	5	23	5	21	6	24	6	24	7	27	8	30	9	35	8
Irenidae	*Irena cyanogaster*				7	14	11	24	10	13	11	22	11	20	17	30	14	26	20	34	12
Irenidae	*Chloropsis flavipennis*	T			7	19	15	28	18	7	16	16	19	28	18	28	18	25	23	33	19
Irenidae	*Chloropsis palawanensis*			x	7	24	28x	23	31x	22	24x	30	19x	24	28x	23	48x	42	41x	39	45x
Irenidae	*Chloropsis sonnerati*				7	13	2	16	3	15	3	18	2	20	3	22	3	28	3	28	2
Irenidae	*Chloropsis cyanopogon*				7	13	2	16	3	15	3	18	2	20	3	22	3	28	3	28	2
Irenidae	*Chloropsis aurifrons*				7	26	7	30	7	27	8	31	8	30	10	34	11	35	13	40	11

Family	Species	IUCN	Ag	R	Range Source	Percentage loss in geographic range 2050 TG H	C	AM H	C	GO H	C	OS H	C	2100 TG H	C	AM H	C	GO H	C	OS H	C
Irenidae	*Chloropsis hardwickii*				7	32	11	36	10	32	13	35	11	37	14	39	13	38	16	42	14
Irenidae	*Chloropsis venusta*				7	26	5	24	5	25	3	27	6	31	6	32	7	46	4	40	6
Orthonychidae	*Orthonyx temminckii*				8,10	12	1	16	1	18	1	21	1	15	6	27	9	27	8	43	4
Orthonychidae	*Orthonyx spaldingii*				10	0	26x	0	19	0	33x	4	24x	0	48x	0	40x	0	57x	4	46x
Pomatostomidae	*Pomatostomus isidorei*				8	4	2	5	2	14	2	14	1	10	3	13	4	24	3	22	1
Pomatostomidae	*Pomatostomus temporalis*				8,10	1	16	1	12	1	15	2	9	1	19	1	17	2	19	2	11
Pomatostomidae	*Pomatostomus superciliosu*				10	0	5	0	4	1	4	1	4	1	6	0	5	1	6	1	4
Pomatostomidae	*Pomatostomus halli*				10	0	19	0	19	0	19	0	19	0	19	0	19	0	19	0	19
Pomatostomidae	*Pomatostomus ruficeps*				10	0	9	0	9	0	9	0	9	0	10	0	10	0	11	0	10
Laniidae	*Lanius tigrinus*				7	11	3	11	3	11	3	10	3	11	4	11	5	11	5	11	5
Laniidae	*Lanius bucephalus*				7	7	5	7	4	8	5	7	5	8	7	8	8	9	7	8	7
Laniidae	*Lanius collurio*		H		3,4,5,7	0	19	0	17	0	21	0	15	0	22	0	20	0	24	0	18
Laniidae	*Lanius isabellinus*		H		4,5,7	0	10	0	10	0	11	0	10	0	12	0	16	0	17	0	16
Laniidae	*Lanius cristatus*		H		7,8	0	8	0	8	0	9	0	8	0	10	0	12	0	13	0	12
Laniidae	*Lanius collurioides*		H		7	0	6	0	6	0	7	0	6	0	8	0	7	0	9	0	7
Laniidae	*Lanius gubernator*			x	5	21	25x	18	25x	27	17x	40	17x	38	25x	20	25x	50	18x	40	17x
Laniidae	*Lanius souzae*				5	54	4	36	5	54	3	61	2	59	5	39	7	57	4	62	2
Laniidae	*Lanius vittatus*		H		4,7	0	4	0	4	0	3	0	2	0	6	0	8	0	9	0	3
Laniidae	*Lanius schach*				4,7,8	17	5	19	4	18	5	21	4	21	6	22	6	23	7	25	6
Laniidae	*Lanius tephronotus*				7	28	9	31	8	30	9	32	8	30	16	34	22	39	21	40	17
Laniidae	*Lanius validirostris*				7	10	11	15	12	7	14	12	13	12	18	19	17	18	21	19	16
Laniidae	*Lanius minor*		H		3,4,5,7	0	15	0	14	0	19	0	13	0	18	0	16	0	20	0	14
Laniidae	*Lanius ludovicianus*				2	3	16	5	12	4	14	4	10	4	18	6	17	7	21	6	14
Laniidae	*Lanius excubitor*				2,3,4,7	3	15	2	14	3	16	3	13	4	18	3	20	5	23	6	19
Laniidae	*Lanius meridionalis*				3,4,5	4	2	3	2	4	2	5	2	5	3	3	4	6	5	6	3
Laniidae	*Lanius sphenocercus*				7	26	3	26	4	27	4	27	4	27	6	28	10	32	7	31	6
Laniidae	*Lanius excubitoroides*				5	22	5	13	7	23	4	27	4	25	5	16	9	28	5	28	4
Laniidae	*Lanius cabanisi*				5	21	3	16	5	23	1	23	1	21	3	20	5	25	1	24	1
Laniidae	*Lanius dorsalis*				5	43	7	14	31	51	2	51	2	46	7	16	34	52	3	52	3
Laniidae	*Lanius somalicus*				5	45	6	13	22	54	2	55	2	50	6	20	31	54	5	55	4
Laniidae	*Lanius mackinnoni*				5	30	4	18	6	31	5	48	3	42	5	29	7	52	5	55	3
Laniidae	*Lanius collaris*		H		5	0	4	0	6	0	5	0	4	0	5	0	10	0	7	0	6
Laniidae	*Lanius newtoni*	T	H	x	5	0	-02x	0	-02x	0	0x	0	0x	0	-02x	0	-02x	0	0x	0	0x
Laniidae	*Lanius senator*		H		3,4,5	0	14	0	10	0	13	0	8	0	20	0	17	0	20	0	11
Laniidae	*Lanius nubicus*		H		3,4,5	0	2	0	1	0	2	0	1	0	3	0	2	0	3	0	1
Laniidae	*Corvinella corvina*				5	8	3	5	3	9	2	12	1	11	3	7	4	13	3	13	2
Laniidae	*Corvinella melanoleuca*				5	17	4	14	6	18	5	18	5	18	7	16	12	20	8	19	8
Laniidae	*Eurocephalus rueppelli*				5	31	2	22	8	34	0	33	1	32	2	25	10	35	0	34	1
Laniidae	*Eurocephalus anguitimens*				5	15	2	11	3	15	3	15	3	15	5	12	11	15	8	15	6
Vireonidae	*Cyclarhis gujanensis*				2	7	12	5	11	7	12	11	10	11	15	10	15	16	16	19	13
Vireonidae	*Cyclarhis nigrirostris*				2	21	2	19	4	14	2	23	3	25	3	31	4	25	5	25	3
Vireonidae	*Vireolanius melitophrys*				2	5	4	19	1	8	4	26	0	5	4	21	4	17	4	28	0
Vireonidae	*Vireolanius pulchellus*				2	18	4	28	5	21	6	36	4	22	5	34	11	29	9	44	5
Vireonidae	*Vireolanius eximius*				2	17	1	20	6	14	7	20	1	23	7	24	8	24	15	29	7

Family	Species	IUCN	Ag	R	Range Source	Percentage loss in geographic range 2050 TG H	TG C	AM H	AM C	GO H	GO C	OS H	OS C	2100 TG H	TG C	AM H	AM C	GO H	GO C	OS H	OS C
Vireonidae	*Vireolanius leucotis*				2	6	10	5	10	5	10	10	9	11	11	10	11	17	12	19	11
Vireonidae	*Vireo brevipennis*				2	8	3	20	2	14	3	34	0x	8	3	20	9	18	3	34	0x
Vireonidae	*Vireo bellii*				2	3	16	7	14	4	16	9	13	6	20	8	23	8	27	11	22
Vireonidae	*Vireo atricapillus*	T			2	2	7	2	8	1	8	4	6	4	9	2	12	1	14	4	9
Vireonidae	*Vireo nelsoni*			x	2	8	4x	14	3x	10	4x	16	0x	9	4x	14	8x	13	4x	16	1x
Vireonidae	*Vireo huttoni*				2	9	19	18	14	11	17	22	11	11	22	20	25	17	29	28	20
Vireonidae	*Vireo carmioli*			x	2	12	22x	35	31x	7	31x	9	31x	13	22x	35	33x	55	33x	45	31x
Vireonidae	*Vireo masteri*	T		x	14	0	0x	8	0x	0	0x	8	0x	8	0x	8	0x	0	0x	8	0x
Vireonidae	*Vireo griseus*				2,13	13	22	20	9	18	14	19	7	22	27	28	17	38	20	29	8
Vireonidae	*Vireo pallens*				2	7	8	15	3	9	6	24	3	9	9	17	16	12	9	27	3
Vireonidae	*Vireo bairdi*			x	2	0	0x	0	0x	0	0x	0	0x	0	0x	0	0x	0	0x	0	0x
Vireonidae	*Vireo gundlachii*				2	9	15x	28	16x	15	10x	40	7x	19	15x	31	18x	20	11x	42	7x
Vireonidae	*Vireo crassirostris*			x	2	24	7x	62	4x	60	8x	50	1x	39	7x	63	5x	67	8x	82	1x
Vireonidae	*Vireo vicinior*				2	0	13	0	15	0	16	0	15	0	20	0	31	1	35	0	33
Vireonidae	*Vireo hypochryseus*				2	13	9	28	6	16	9	37	4	18	11	28	18	23	14	40	7
Vireonidae	*Vireo modestus*			x	2	27	0x	27	3x	23	3x	52	3x	52	0x	27	3x	23	3x	52	3x
Vireonidae	*Vireo nanus*			x	2	9	17x	12	13x	9	17x	26	0x	9	17x	12	14x	9	17x	26	0x
Vireonidae	*Vireo latimeri*			x	2	0	0x	0	0x	0	0x	0	0x	0	0x	0	0x	0	0x	0	0x
Vireonidae	*Vireo osburni*			x	2	17	0x	17	5x	11	5x	44	5x	44	0x	17	5x	11	5x	44	5x
Vireonidae	*Vireo cassinii*				2	6	30	10	24	8	28	12	21	8	38	14	36	15	42	22	29
Vireonidae	*Vireo plumbeus*				2	4	8	10	6	5	8	13	6	5	10	11	13	9	19	16	13
Vireonidae	*Vireo solitarius*				2	10	20	15	17	14	18	15	15	13	25	20	23	23	24	23	21
Vireonidae	*Vireo flavifrons*				2	15	25	25	12	24	17	25	8	24	29	34	15	43	21	37	8
Vireonidae	*Vireo philadelphicus*				2	7	19	10	16	10	18	10	16	10	24	13	22	16	24	16	21
Vireonidae	*Vireo olivaceus*				2	9	21	13	16	12	18	14	14	14	24	18	22	23	24	22	18
Vireonidae	*Vireo flavoviridis*				2	11	6	20	5	14	6	29	4	15	7	23	20	18	12	34	6
Vireonidae	*Vireo altiloquus*				2	13	16	24	14	16	16	31	8	17	17	26	16	19	17	36	8
Vireonidae	*Vireo magister*			x	2	1	0x	15	0x	0	0x	16	0x	15	0x	22	0x	12	0x	21	0x
Vireonidae	*Vireo gilvus*				2	6	17	10	12	9	15	11	10	9	20	14	17	16	21	17	14
Vireonidae	*Vireo leucophrys*				2	14	6	14	5	14	7	21	6	18	6	20	11	24	10	25	8
Vireonidae	*Hylophilus amaurocephalu*		H		2	0	14	0	8	0	11	0	4	0	19	0	10	0	13	0	6
Vireonidae	*Hylophilus poicilotis*				2	22	9	21	7	30	5	34	6	34	12	36	11	50	8	56	10
Vireonidae	*Hylophilus thoracicus*				2	4	8	3	8	3	7	7	7	6	9	7	10	10	10	13	8
Vireonidae	*Hylophilus semicinereus*				2	4	9	3	9	3	9	7	8	8	10	6	11	14	12	15	10
Vireonidae	*Hylophilus pectoralis*				2	7	20	3	20	4	22	11	17	10	25	7	24	15	27	21	21
Vireonidae	*Hylophilus sclateri*				2	5	16	5	18	2	19	2	20	7	20	8	23	5	27	6	29
Vireonidae	*Hylophilus muscicapinus*				2	4	17	3	18	3	19	6	17	7	20	5	21	11	22	11	20
Vireonidae	*Hylophilus brunneiceps*				2	0	4	0	4	0	4	0	4	2	7	1	9	0	9	0	9
Vireonidae	*Hylophilus semibrunneus*			x	2	19	0x	16	0x	16	0x	14	0x	22	0x	31	0x	34	2x	16	0x
Vireonidae	*Hylophilus aurantiifrons*				2	19	5	16	6	18	5	24	4	23	8	24	10	32	14	34	8
Vireonidae	*Hylophilus flavipes*				2	19	5	13	7	16	5	20	3	22	7	19	10	30	11	32	6
Vireonidae	*Hylophilus olivaceus*				2	6	8	6	7	10	8	19	6	14	8	19	13	30	11	30	9
Vireonidae	*Hylophilus ochraceiceps*				2	5	8	5	7	5	8	9	7	9	9	9	10	15	10	17	9
Vireonidae	*Hylophilus decurtatus*				2	14	5	20	5	15	6	26	4	19	5	24	10	22	10	33	6

Family	Species	IUCN	Ag	R	Range Source	2050 TG H	2050 TG C	2050 AM H	2050 AM C	2050 GO H	2050 GO C	2050 OS H	2050 OS C	2100 TG H	2100 TG C	2100 AM H	2100 AM C	2100 GO H	2100 GO C	2100 OS H	2100 OS C
Corvidae	*Androphobus viridis*				8	5	1	11	0	1	0	5	1	8	1	39	1	6	0	8	1
Corvidae	*Psophodes olivaceus*				10	9	12	15	10	20	9	23	9	15	17	20	14	28	14	37	13
Corvidae	*Psophodes nigrogularis*			x	10	0	6x	0	0x	0	0x	0	0x	0	6x	0	0x	0	0x	0	0x
Corvidae	*Psophodes occidentalis*				10	0	5	0	5	0	5	0	5	0	5	0	5	0	5	0	5
Corvidae	*Psophodes cristatus*				10	0	7	0	7	0	7	0	7	0	8	0	8	0	8	0	8
Corvidae	*Cinclosoma punctatum*				10	8	12	12	5	16	7	17	3	14	18	15	11	22	14	26	5
Corvidae	*Cinclosoma castanotus*				10	0	5	0	4	0	5	0	5	0	5	0	6	0	8	0	6
Corvidae	*Cinclosoma castaneothora*				10	0	7	0	7	0	7	0	8	0	7	0	7	0	7	0	8
Corvidae	*Cinclosoma cinnamomeum*				10	0	1	0	1	0	1	0	1	0	1	0	1	0	1	0	1
Corvidae	*Cinclosoma ajax*				8	2	5	2	5	17	1	30	1	8	8	17	8	33	4	41	1
Corvidae	*Ptilorrhoa leucosticta*				8	7	0	4	0	20	0	14	0	12	7	13	8	33	8	33	4
Corvidae	*Ptilorrhoa caerulescens*				8	4	2	6	2	15	2	15	1	11	4	13	4	26	3	24	2
Corvidae	*Ptilorrhoa castanonota*				8	6	1	4	0	20	0	11	0	11	6	10	7	34	6	28	3
Corvidae	*Eupetes macrocerus*				7	13	2	16	3	15	3	19	3	20	3	22	3	28	3	28	3
Corvidae	*Ifrita kowaldi*				8	6	0	4	0	22	0	13	0	11	6	12	8	36	7	30	4
Corvidae	*Corcorax melanorhamphos*				10	3	19	4	12	7	15	7	9	6	26	6	21	9	24	12	11
Corvidae	*Struthidea cinerea*				10	0	23	0	15	2	19	2	11	1	29	0	25	2	29	3	13
Corvidae	*Daphoenositta chrysoptera*				8,10	1	13	1	9	2	12	2	7	2	16	2	13	3	16	3	8
Corvidae	*Daphoenositta miranda*				8	8	0	4	0	20	0	16	0	14	6	15	9	32	9	33	5
Corvidae	*Mohoua albicilla*				12,13	11	21	15	21	34	11	33	8	11	21	21	21	34	14	41	8
Corvidae	*Mohoua ochrocephala*	T		x	12,13	0	22x	0	19x	1	22x	0	19x	0	32x	0	36x	1	43x	4	31x
Corvidae	*Mohoua novaeseelandiae*				12,13	0	28	0	23	6	27	1	23	2	39	0	39	7	46	7	36
Corvidae	*Falcunculus frontatus*				10	6	11	8	6	11	9	13	4	11	16	11	9	16	14	20	6
Corvidae	*Oreoica gutturalis*				10	0	9	0	9	0	9	0	8	0	10	0	10	0	10	0	9
Corvidae	*Rhagologus leucostigma*				8	6	0	4	0	20	0	13	0	10	6	12	8	33	7	32	4
Corvidae	*Pachycare flavogrisea*				8	7	1	6	1	20	1	12	0	11	6	12	7	34	6	30	3
Corvidae	*Hylocitrea bonensis*				7	14	14	26	9	19	13	16	12	17	15	30	22	29	30	24	27
Corvidae	*Aleadryas rufinucha*				8	6	0	3	0	20	0	12	0	10	6	11	8	34	7	31	3
Corvidae	*Coracornis raveni*				7	17	17	25	10	17	17	22	12	20	19	30	22	25	32	29	21
Corvidae	*Pachycephala olivacea*				10	7	19	11	7	17	15	16	7	15	21	17	8	21	16	29	7
Corvidae	*Pachycephala rufogularis*				10	0	19	0	12	0	19	0	12	0	19	0	12	0	19	0	12
Corvidae	*Pachycephala inornata*				10	0	9	0	7	0	9	1	7	1	12	0	10	1	15	1	8
Corvidae	*Pachycephala grisola*				7	11	3	14	3	14	3	16	3	17	4	19	5	25	4	25	4
Corvidae	*Pachycephala albiventris*				7	6	7	19	3	10	8	16	5	6	19	31	11	19	22	28	6
Corvidae	*Pachycephala homeyeri*				7	18	11x	18	10x	8	15x	8	14x	22	12x	22	11x	14	18x	18	14x
Corvidae	*Pachycephala phaionotus*			x	7,8	7	2x	9	6x	30	6x	28	6x	36	6x	19	10x	42	11x	32	6x
Corvidae	*Pachycephala hyperythra*				8	6	1	9	0	16	0	13	1	13	4	16	3	26	2	24	1
Corvidae	*Pachycephala modesta*				8	6	0	3	0	27	0	16	0	9	7	4	13	47	12	42	6
Corvidae	*Pachycephala hypoxantha*				7	9	0	12	1	10	2	13	1	15	0	16	1	18	2	21	1
Corvidae	*Pachycephala sulfuriventer*				7	14	14	26	9	19	13	16	12	16	15	30	22	29	30	24	27
Corvidae	*Pachycephala philippinensi*				7	15	11	24	11	13	12	22	12	21	18	30	14	26	21	34	12
Corvidae	*Pachycephala meyeri*			x	8	23	0x	5	0x	7	0x	5	0x	24	0x	10	0x	8	0x	53	0x
Corvidae	*Pachycephala griseiceps*				8	4	3	6	3	17	2	15	1	10	5	13	6	28	4	24	3
Corvidae	*Pachycephala simplex*				10	0	18	0	11	0	20	2	7	0	22	0	19	0	22	2	9

Family	Species	IUCN	Ag	R	Range Source	2050 TG H	2050 TG C	2050 AM H	2050 AM C	2050 GO H	2050 GO C	2050 OS H	2050 OS C	2100 TG H	2100 TG C	2100 AM H	2100 AM C	2100 GO H	2100 GO C	2100 OS H	2100 OS C
Corvidae	*Pachycephala orpheus*			x	7	10	31x	3	31x	11	24x	8	23x	10	31x	3	31x	11	24x	8	23x
Corvidae	*Pachycephala pectoralis*				8,9,10,11	4	15	5	8	10	11	10	6	8	21	8	15	14	19	16	8
Corvidae	*Pachycephala soror*				8	6	0	3	0	21	0	10	0	10	6	10	7	35	7	29	3
Corvidae	*Pachycephala lorentzi*				8	6	0	5	0	13	0	8	0	12	5	27	0	18	0	14	0
Corvidae	*Pachycephala melanura*				8,9,10	2	16	4	9	19	14	20	5	10	16	9	14	26	17	25	7
Corvidae	*Pachycephala caledonica*			x	9	14	30x	44	9x	25	18x	17	19x	14	30x	55	11x	26	21x	20	19x
Corvidae	*Pachycephala flavifrons*			x	11	0	0x	0	0x	2	0x	0	0x	50	0x	0	0x	2	0x	2	0x
Corvidae	*Pachycephala jacquinoti*			x	11	0	0x	0	0x	0	0x	0	0x	0	0x	0	0x	0	0x	0	0x
Corvidae	*Pachycephala schlegelii*				8	7	0	4	0	19	0	13	0	11	6	13	8	31	8	32	4
Corvidae	*Pachycephala nudigula*			x	7	18	9x	12	12x	4	12x	7	7x	18	32x	12	35x	22	18x	23	7x
Corvidae	*Pachycephala implicata*			x	9	10	0x	10	0x	8	0x	17	0x	19	0x	10	0x	41	0x	57	0x
Corvidae	*Pachycephala aurea*				8	3	0	5	0	20	0	33	0	7	5	14	6	43	5	38	3
Corvidae	*Pachycephala griseonota*				7	17	0	22	0	20	0	19	0	30	1	23	1	31	1	34	1
Corvidae	*Pachycephala arctitorquis*			x	7	15	22x	6	22x	19	7x	40	27x	15	22x	6	22x	28	7x	40	27x
Corvidae	*Pachycephala monacha*				8	8	0	5	2	27	2	14	1	14	5	9	6	41	6	31	3
Corvidae	*Pachycephala leucogastra*			x	8	7	0x	0	0x	40	0x	40	0x	7	0x	0	0x	40	0x	58	0x
Corvidae	*Pachycephala rufiventris*				8,9,10	1	15	1	10	2	13	2	7	2	18	2	15	3	18	4	9
Corvidae	*Pachycephala lanioides*				10	0	23	1	14	0	22	1	13	0	23	1	21	0	26	1	16
Corvidae	*Colluricincla umbrina*				8	0	0	6	0	10	0	6	0	3	4	23	0	15	0	12	0
Corvidae	*Colluricincla megarhyncha*				8,10	3	10	5	7	13	9	11	4	7	13	9	12	20	13	20	6
Corvidae	*Colluricincla boweri*			x	10	0	27x	0	20x	0	33x	4	23x	0	47x	0	41x	0	56x	4	45x
Corvidae	*Colluricincla woodwardi*			x	10	0	0x	0	0x	0	0x	0	0x	0	0x	0	8x	0	8x	0	8x
Corvidae	*Colluricincla harmonica*				8,10	1	12	1	9	2	10	2	7	2	13	2	12	3	13	4	8
Corvidae	*Pitohui kirhocephalus*				8	5	2	6	2	15	2	14	1	12	4	13	5	25	4	23	2
Corvidae	*Pitohui dichrous*				8	4	2	6	2	21	2	15	0	8	7	13	8	37	7	29	2
Corvidae	*Pitohui incertus*				8	5	0	3	0	1	0	9	0	5	0	12	0	10	0	14	0
Corvidae	*Pitohui ferrugineus*				8	4	3	6	3	16	2	15	1	10	5	13	6	26	4	24	2
Corvidae	*Pitohui cristatus*				8	5	1	9	1	13	0	15	0	10	7	18	6	26	6	25	1
Corvidae	*Pitohui nigrescens*				8	7	0	3	0	18	0	12	0	11	7	11	9	31	8	31	4
Corvidae	*Eulacestoma nigropectus*				8	7	0	2	0	18	0	12	0	11	7	11	9	32	9	28	4
Corvidae	*Platylophus galericulatus*				7	13	2	16	3	15	3	18	2	20	3	22	3	28	3	28	2
Corvidae	*Platysmurus leucopterus*				7	14	3	19	3	18	3	21	3	23	4	26	4	33	4	32	3
Corvidae	*Gymnorhinus cyanocephal*	T			2	0	13	2	12	2	13	2	11	1	15	2	19	3	22	4	17
Corvidae	*Cyanocitta cristata*				2	9	19	14	11	14	15	13	8	14	23	19	15	24	19	19	10
Corvidae	*Cyanocitta stelleri*				2	6	20	13	16	8	19	17	14	8	26	15	28	14	32	24	23
Corvidae	*Aphelocoma insularis*			x	2	0	73x	0	73x	0	73x	0	0x	0	73x	0	73x	0	73x	0	0x
Corvidae	*Aphelocoma californica*				2	2	14	5	11	3	13	6	9	2	15	5	19	5	22	9	15
Corvidae	*Aphelocoma coerulescens*	T		x	2	14	85x	17	71x	3	93x	11	73x	14	86x	22	78x	4	95x	13	74x
Corvidae	*Aphelocoma ultramarina*				2	8	10	20	5	9	8	27	5	11	12	21	18	14	18	31	13
Corvidae	*Aphelocoma unicolor*				2	9	0	19	0	12	2	27	0	9	0	21	3	19	2	28	0
Corvidae	*Cyanolyca armillata*				2	17	4	23	6	13	6	30	6	26	10	29	14	32	15	35	9
Corvidae	*Cyanolyca turcosa*			x	2	33	11x	41	8x	30	18x	42	17x	42	22x	61	20x	67	21x	48	28x
Corvidae	*Cyanolyca viridicyana*				2	13	8	8	3	14	6	17	10	19	8	15	14	26	9	22	12
Corvidae	*Cyanolyca cucullata*				2	12	1	23	1	19	1	27	1	15	1	25	2	27	2	32	1

Family	Species	IUCN	Ag	R	Range Source	Percentage loss in geographic range 2050 TG H	C	AM H	C	GO H	C	OS H	C	2100 TG H	C	AM H	C	GO H	C	OS H	C
Corvidae	*Cyanolyca pulchra*			x	2	0	3x	10	3x	4	3x	13	0x	10	3x	12	3x	4	3x	13	0x
Corvidae	*Cyanolyca pumilo*				2	12	1	17	1	15	4	22	1	13	1	18	2	17	4	25	1
Corvidae	*Cyanolyca nana*	T		x	2	0	0x	1	0x	1	0x	1	0x	0	0x	1	0x	1	0x	1	0x
Corvidae	*Cyanolyca mirabilis*	T		x	2	4	0x	28	0x	9	0x	20	0x	4	0x	28	11x	32	0x	20	0x
Corvidae	*Cyanolyca argentigula*			x	2	14	26x	31	38x	6	38x	6	38x	14	26x	31	38x	56	38x	38	38x
Corvidae	*Cyanocorax melanocyaneu*				2	15	1	24	1	24	4	32	1	17	1	25	1	25	5	35	1
Corvidae	*Cyanocorax sanblasianus*				2	6	1	20	1	10	1	33	0	7	1	20	28	22	1	37	0
Corvidae	*Cyanocorax yucatanicus*			x	2	0	7x	9	1x	3	7x	14	1x	1	12x	15	16x	11	7x	15	7x
Corvidae	*Cyanocorax beecheii*			x	2	4	13x	6	9x	1	12x	19	6x	12	13x	6	51x	5	14x	20	8x
Corvidae	*Cyanocorax cyanomelas*				2	11	15	7	15	8	16	14	14	15	18	12	22	19	21	22	20
Corvidae	*Cyanocorax caeruleus*				2	22	5	17	6	29	3	31	5	30	9	33	11	50	4	54	8
Corvidae	*Cyanocorax violaceus*				2	5	5	3	5	4	5	8	5	8	7	8	7	11	8	15	7
Corvidae	*Cyanocorax cristatellus*				2	8	23	3	21	4	24	10	19	11	28	7	23	14	26	17	20
Corvidae	*Cyanocorax heilprini*				2	0	6	0	6	0	6	0	6	0	9	0	11	0	11	0	11
Corvidae	*Cyanocorax cayanus*				2	4	16	3	15	4	15	7	14	8	17	6	18	14	20	13	18
Corvidae	*Cyanocorax affinis*				2	16	5	18	8	11	9	17	6	21	8	22	11	20	17	24	11
Corvidae	*Cyanocorax dickeyi*				2	24	12	40	5	18	5	40	5	24	27	40	28	23	40	43	20
Corvidae	*Cyanocorax chrysops*				2	10	12	8	11	9	11	13	10	15	15	13	18	22	18	24	17
Corvidae	*Cyanocorax cyanopogon*				2	8	20	5	16	6	18	13	11	10	24	8	19	15	22	22	15
Corvidae	*Cyanocorax mystacalis*				2	2	12	0	12	0	15	1	12	2	14	0	18	2	21	2	18
Corvidae	*Cyanocorax luxuosus*				2	5	8	13	5	8	6	18	3	6	12	16	24	12	13	21	5
Corvidae	*Cyanocorax yncas*				2	19	4	15	4	15	5	22	3	23	6	23	9	26	12	28	7
Corvidae	*Psilorhinus morio*				2	10	5	18	3	14	5	25	3	13	6	22	16	19	8	29	4
Corvidae	*Calocitta colliei*				2	17	18	26	12	13	17	37	10	25	21	26	24	20	28	43	17
Corvidae	*Calocitta formosa*				2	11	1	26	0	20	1	32	0	13	1	27	11	24	3	33	0
Corvidae	*Garrulus glandarius*				3,4,5,7	9	14	8	12	10	15	10	11	12	17	11	15	14	18	14	14
Corvidae	*Garrulus lidthi*	T		x	7	30	7x	7	0x	10	0x	0	7x	39	7x	43	2x	45	0x	0	7x
Corvidae	*Perisoreus infaustus*				3,7	1	13	1	13	1	13	1	13	2	16	2	17	3	17	3	17
Corvidae	*Perisoreus internigrans*	T			7	19	0	20	1	19	1	19	1	19	3	23	3	21	3	20	2
Corvidae	*Perisoreus canadensis*				2	1	16	3	15	2	15	3	15	2	21	4	23	5	24	6	21
Corvidae	*Urocissa ornata*	T		x	7	47	0x	42	0x	35	0x	43	0x	48	0x	47	0x	40	0x	48	0x
Corvidae	*Urocissa caerulea*				7	0	0	0	0	0	0	0	0	0	0	0	0	0	0	0	0
Corvidae	*Urocissa flavirostris*				7	41	3	43	2	41	2	44	2	44	4	46	3	46	3	48	2
Corvidae	*Urocissa erythrorhyncha*				7	13	5	15	4	13	6	14	6	15	7	16	7	16	9	17	8
Corvidae	*Urocissa whiteheadi*				7	6	4	7	3	6	4	8	2	6	4	7	3	8	4	10	2
Corvidae	*Cissa chinensis*				7	26	6	29	6	26	7	30	6	31	8	32	8	35	8	37	8
Corvidae	*Cissa hypoleuca*				7	5	16	7	16	10	17	11	13	6	17	8	19	16	20	16	16
Corvidae	*Cissa thalassina*				7	9	0	8	2	6	2	10	0	12	0	11	2	14	2	18	0
Corvidae	*Cyanopica cyana*				3,7	12	5	12	5	12	5	12	5	12	7	12	9	13	9	13	8
Corvidae	*Dendrocitta vagabunda*				7	24	8	27	7	25	8	28	7	27	10	30	10	30	12	38	9
Corvidae	*Dendrocitta formosae*				7	18	4	19	3	18	5	19	4	20	5	21	5	20	5	22	5
Corvidae	*Dendrocitta occipitalis*				7	16	2	16	3	16	2	17	3	21	3	21	4	30	3	27	3
Corvidae	*Dendrocitta leucogastra*				7	22	1	43	2	35	2	44	2	24	2	50	2	41	4	74	2
Corvidae	*Dendrocitta frontalis*				7	53	4	54	2	56	0	58	0	56	5	59	3	60	1	63	1

Family	Species	IUCN	Ag	R	Range Source	Percentage loss in geographic range 2050 TG H	C	AM H	C	GO H	C	OS H	C	2100 TG H	C	AM H	C	GO H	C	OS H	C
Corvidae	*Dendrocitta bayleyi*			x	7	9	0x	9	0x	9	0x	9	0x	9	0x	20	0x	20	0x	20	0x
Corvidae	*Crypsirina temia*				7	13	8	17	7	14	9	16	9	15	13	18	13	18	17	21	13
Corvidae	*Crypsirina cucullata*				7	24	16	25	16	22	22	23	19	31	23	29	24	28	25	34	23
Corvidae	*Temnurus temnurus*				7	7	14	14	10	12	12	19	10	10	16	17	13	20	17	24	14
Corvidae	*Pica pica*				3,4,5,7	8	12	8	11	9	13	9	10	10	15	10	14	13	16	13	13
Corvidae	*Pica hudsonia*				14	1	17	2	15	2	16	3	13	2	21	3	20	4	22	6	17
Corvidae	*Pica nuttalli*				2	0	66	3	40	0	63	6	27	0	68	3	51	0	68	10	29
Corvidae	*Zavattariornis stresemanni*	T		x	5	80	20x	12	32x	100	0x	100	0x	80	20x	33	32x	100	0x	100	0x
Corvidae	*Podoces hendersoni*				7	1	10	1	11	1	11	1	10	1	14	1	20	2	23	2	22
Corvidae	*Podoces biddulphi*				7	1	26	2	26	2	28	1	28	2	30	2	31	4	33	6	32
Corvidae	*Podoces panderi*			x	7	10	14x	0	14x	0	28x	0	28x	10	28x	0	28x	0	28x	10	28x
Corvidae	*Podoces pleskei*				4	2	5	1	5	5	4	13	3	15	7	5	6	19	5	20	4
Corvidae	*Pseudopodoces humilis*				7	16	20	18	19	18	19	19	18	19	32	22	40	27	40	27	36
Corvidae	*Nucifraga columbiana*				2	4	20	8	16	6	19	8	16	5	28	10	31	11	35	16	26
Corvidae	*Nucifraga caryocatactes*				3,4,7	6	18	5	18	7	19	6	17	8	20	8	21	11	22	13	20
Corvidae	*Pyrrhocorax pyrrhocorax*				3,4,5,7	12	10	12	11	13	11	13	10	14	15	14	18	17	19	18	17
Corvidae	*Pyrrhocorax graculus*				3,4,5,7	13	16	14	16	15	16	15	15	16	25	18	31	23	31	23	27
Corvidae	*Ptilostomus afer*				5	10	2	7	3	12	1	13	1	12	2	8	4	14	2	14	1
Corvidae	*Corvus monedula*		H		3,4,5,7	0	17	0	16	0	18	0	14	0	21	0	20	0	24	0	19
Corvidae	*Corvus dauuricus*		H		7	0	3	0	4	0	4	0	4	0	4	0	6	0	7	0	6
Corvidae	*Corvus splendens*				4,5,7,13	11	2	12	2	12	2	14	1	12	4	13	6	13	8	19	2
Corvidae	*Corvus moneduloides*			x	9	14	31x	35	13x	32	18x	26	13x	14	31x	52	14x	36	19x	34	13x
Corvidae	*Corvus enca*				7	13	4	18	4	15	5	18	4	20	6	23	6	28	8	27	6
Corvidae	*Corvus typicus*				7	7	5	26	7	20	1	6	11	7	5	26	7	23	8	13	18
Corvidae	*Corvus unicolor*	T		x	7	65	23x	73	23x	0	83x	4	83x	65	23x	73	23x	0	83x	4	83x
Corvidae	*Corvus florensis*	T		x	7	19	6x	29	15x	8	15x	1	15x	19	6x	29	15x	8	15x	23	15x
Corvidae	*Corvus validus*				7	20	0	28	0	24	0	23	0	36	0x	29	0	42	0x	39	0x
Corvidae	*Corvus meeki*			x	9	10	0x	10	0x	11	0x	12	0x	20	0x	10	0x	40	0x	37	0x
Corvidae	*Corvus woodfordi*			x	9	12	4x	12	0x	16	0x	34	0x	29	15x	35	0x	62	0x	50	0x
Corvidae	*Corvus fuscicapillus*			x	8	9	0x	24	6x	22	6x	12	6x	16	0x	37	6x	28	6x	17	6x
Corvidae	*Corvus tristis*				8	5	2	6	2	17	2	15	1	11	4	13	4	27	4	25	2
Corvidae	*Corvus capensis*		H		5	0	4	0	7	0	5	0	4	0	6	0	13	0	8	0	8
Corvidae	*Corvus frugilegus*		H		3,4,7,12,13	0	12	0	12	0	14	0	11	0	15	0	15	0	18	0	14
Corvidae	*Corvus caurinus*		H		2	0	25	0	22	0	23	0	16	0	29	0	29	0	32	0	22
Corvidae	*Corvus brachyrhynchos*		H		2,13	0	18	0	13	0	16	0	11	0	22	0	18	0	21	0	15
Corvidae	*Corvus ossifragus*		H		2	0	39	0	16	0	29	0	10	0	45	0	22	0	37	0	10
Corvidae	*Corvus imparatus*		H		2	0	8	0	4	0	4	0	3	0	12	0	45	0	5	0	3
Corvidae	*Corvus sinaloae*		H		2	0	18	0	14	0	16	0	9	0	24	0	31	0	30	0	16
Corvidae	*Corvus palmarum*				2	15	12	21	10	16	14	32	0	15	15	21	11	16	14	32	0
Corvidae	*Corvus minutus*	T		x	14	0	0x	39	0x	21	0x	23	0x	9	0x	41	18x	21	0x	23	0x
Corvidae	*Corvus jamaicensis*		H	x	2	0	0x	0	3x	0	3x	0	3x	0	0x	0	3x	0	3x	0	3x
Corvidae	*Corvus nasicus*				2	9	15x	27	16x	14	10x	39	7x	19	15x	31	18x	19	11x	42	7x
Corvidae	*Corvus leucognaphalus*	T		x	2	11	6x	16	3x	11	6x	20	0x	11	7x	16	4x	11	6x	20	0x
Corvidae	*Corvus corone*				3,4,5,7	6	14	5	14	6	15	6	13	7	17	7	18	10	19	10	17

Family	Species	IUCN	Ag	R	Range Source	2050 TG H	2050 TG C	2050 AM H	2050 AM C	2050 GO H	2050 GO C	2050 OS H	2050 OS C	2100 TG H	2100 TG C	2100 AM H	2100 AM C	2100 GO H	2100 GO C	2100 OS H	2100 OS C
Corvidae	*Corvus macrorhynchos*		H		4,7	0	5	0	4	0	5	0	5	0	7	0	7	0	8	0	7
Corvidae	*Corvus orru*		H		7,8,10	0	14	0	10	0	12	0	8	0	16	0	15	0	16	0	10
Corvidae	*Corvus bennetti*				10	0	8	0	7	0	8	0	8	0	9	0	11	0	13	0	9
Corvidae	*Corvus coronoides*				10	1	15	1	11	2	14	2	9	2	18	2	16	3	19	4	10
Corvidae	*Corvus mellori*		H		10	0	14	0	9	0	13	0	6	0	17	0	11	0	17	0	7
Corvidae	*Corvus tasmanicus*				10	9	25	12	6	13	22	14	5	19	26	17	6	20	22	26	5
Corvidae	*Corvus torquatus*		H		7	0	1	0	0	0	1	0	1	0	1	0	0	0	1	0	1
Corvidae	*Corvus hawaiiensis*				11	1	1	18	1	15	1	32	0	41	1	64	1	42	1	92	1
Corvidae	*Corvus cryptoleucus*				2	1	21	2	18	2	19	3	16	1	25	2	33	2	34	4	28
Corvidae	*Corvus albus*				4,5,6,13	20	3	12	6	21	3	24	2	23	4	15	9	25	5	25	4
Corvidae	*Corvus ruficollis*				3,4,5,7	2	2	2	2	2	2	4	1	3	2	2	2	4	3	5	2
Corvidae	*Corvus ruficollis*				14	37	5	9	25	44	1	44	1	41	5	12	30	44	4	44	3
Corvidae	*Corvus corax*				2,3,4,5,7	5	15	6	14	6	16	7	14	7	19	7	22	9	24	10	20
Corvidae	*Corvus rhipidurus*				4,5	17	3	6	9	20	2	20	2	19	3	8	12	20	4	20	4
Corvidae	*Corvus albicollis*				5	26	3	22	4	27	2	27	2	27	4	24	6	28	3	28	3
Corvidae	*Corvus crassirostris*				5	30	0	19	1	32	0	32	0	32	0	23	4	32	1	32	1
Corvidae	*Melampitta lugubris*				8	6	0	5	0	19	0	14	0	10	8	14	10	29	9	35	5
Corvidae	*Melampitta gigantea*			x	8	4	0x	17	0x	10	0x	10	0x	6	0x	29	0x	17	0x	34	0x
Corvidae	*Loboparadisea sericea*				8	5	1	3	0	34	0	9	0	7	7	3	4	44	4	23	0
Corvidae	*Cnemophilus macgregorii*			x	8	15	0x	1	0x	26	0x	28	0x	26	6x	8	12x	48	12x	52	9x
Corvidae	*Cnemophilus loriae*				8	3	0	2	0	16	0	10	0	8	8	12	11	32	11	27	5
Corvidae	*Macgregoria pulchra*	T		x	8	3	0x	1	0x	15	0x	12	0x	12	10x	13	15x	19	15x	16	10x
Corvidae	*Lycocorax pyrrhopterus*				7	20	0	28	0	23	0	23	0	35	0	30	0	42	0x	40	0
Corvidae	*Manucodia atra*				8	4	6	7	5	20	3	21	2	12	10	16	11	31	8	33	4
Corvidae	*Manucodia chalybata*				8	6	3	4	3	27	2	19	1	11	6	9	10	43	8	35	3
Corvidae	*Manucodia comrii*			x	8	14	4x	51	4x	72	4x	73	0x	51	4x	51	4x	72	4x	73	0x
Corvidae	*Manucodia jobiensis*				8	4	4	7	2	26	2	16	1	16	4	12	4	37	2	33	1
Corvidae	*Manucodia keraudrenii*				8,10	3	4	4	4	22	2	15	1	5	7	9	10	35	7	27	2
Corvidae	*Semioptera wallacii*			x	7	12	0x	22	0x	24	0x	23	0x	31	0x	22	0x	42	0x	38	0x
Corvidae	*Paradigalla carunculata*			x	8	0	0x	12	0x	0	0x	5	0x	4	0x	15	0x	0	0x	16	0x
Corvidae	*Paradigalla brevicauda*				8	7	0	2	0	22	0	10	0	14	3	10	0	39	0	20	0
Corvidae	*Epimachus fastuosus*	T			8	13	0	1	0	24	0	9	0	19	4	7	0	41	0	24	0
Corvidae	*Epimachus meyeri*				8	10	0	1	0	28	0	18	0	18	10	8	13	41	12	37	6
Corvidae	*Epimachus albertisi*				8	6	0	3	0	27	0	13	0	10	5	6	10	46	9	34	5
Corvidae	*Epimachus bruijnii*				8	4	0	9	0	13	0	3	0	19	0	12	0	19	0	21	0
Corvidae	*Lophorina superba*				8	6	0	4	0	22	0	13	0	11	8	10	10	37	9	29	4
Corvidae	*Parotia sefilata*			x	8	42	0x	9	0x	14	0x	0	0x	44	0x	9	0x	14	0x	29	0x
Corvidae	*Parotia carolae*				8	7	0	5	0	24	0	12	0	13	3	15	0	41	0	26	0
Corvidae	*Parotia lawesii*				8	4	0	0	0	29	0	16	0	10	9	3	16	49	14	37	6
Corvidae	*Parotia helenae*			x	8	0	0x	0	0x	5	0x	16	0x	11	29x	0	57x	26	47x	41	28x
Corvidae	*Parotia wahnesi*	T		x	8	0	7x	17	2x	44	2x	24	0x	0	7x	17	2x	44	2x	50	0x
Corvidae	*Ptiloris magnificus*				8,10	4	5	4	4	21	4	18	1	11	8	10	11	34	10	30	3
Corvidae	*Ptiloris intercedens*				8	4	3	6	3	16	2	15	1	10	5	13	6	26	4	25	2
Corvidae	*Ptiloris victoriae*				10	0	26x	0	19	0	34x	4	23x	0	47x	0	40x	0	57x	4	45x

Family	Species	IUCN	Ag	R	Range Source	Percentage loss in geographic range 2050 TG H	2050 TG C	2050 AM H	2050 AM C	2050 GO H	2050 GO C	2050 OS H	2050 OS C	2100 TG H	2100 TG C	2100 AM H	2100 AM C	2100 GO H	2100 GO C	2100 OS H	2100 OS C
Corvidae	*Ptiloris paradiseus*				10	19	5	25	7	30	3	33	3	22	7	30	8	37	4	56	4
Corvidae	*Cicinnurus magnificus*				8	6	2	4	2	22	2	18	1	11	9	7	13	35	11	36	5
Corvidae	*Cicinnurus respublica*			x	8	10	0x	0	0x	0	0x	0	0x	10	0x	10	0x	0	0x	0	0x
Corvidae	*Cicinnurus regius*				8	5	4	8	4	26	3	25	2	13	8	15	10	38	7	37	3
Corvidae	*Astrapia nigra*			x	8	11	0x	6	0x	0	0x	0	0x	11	0x	6	0x	0	0x	75	0x
Corvidae	*Astrapia splendidissima*				8	6	0	3	0	13	0	8	0	13	5	21	0	18	0	16	0
Corvidae	*Astrapia mayeri*			x	8	24	0x	0	0x	21	0x	32	0x	27	0x	0	0x	51	0x	71	0x
Corvidae	*Astrapia stephaniae*				8	6	0	0	0	26	0	15	0	11	10	1	18	50	16	36	8
Corvidae	*Astrapia rothschildi*			x	8	0	5x	27	0x	50	0x	28	0x	0	5x	27	0x	50	0x	53	0x
Corvidae	*Pteridophora alberti*				8	11	0	3	0	25	0	17	0	18	3	10	0	42	0	33	0
Corvidae	*Seleucidis melanoleuca*				8	2	1	3	1	15	1	16	0	13	3	13	2	24	2	28	0
Corvidae	*Paradisaea rubra*			x	8	12	0x	0	0x	0	0x	0	0x	12	0x	12	0x	0	0x	0	0x
Corvidae	*Paradisaea minor*				8	4	4	7	2	26	2	14	1	16	6	14	4	36	2	30	1
Corvidae	*Paradisaea apoda*				8	1	0	5	1	10	1	12	1	3	0	11	2	17	1	16	1
Corvidae	*Paradisaea raggiana*				8	2	5	4	4	30	1	28	0	6	10	9	13	50	9	42	3
Corvidae	*Paradisaea decora*			x	8	21	0x	69	0x	100	0x	93	0x	69	0x	69	0x	100	0x	93	0x
Corvidae	*Paradisaea guilielmi*			x	8	0	6x	17	0x	45	0x	18	0x	0	6x	17	0x	49	0x	39	0x
Corvidae	*Paradisaea rudolphi*	T			8	1	0	0	0	37	0x	12	0	7	13x	1	24x	53	20x	30	11x
Corvidae	*Cracticus mentalis*				8,10	0	28	2	18	10	20	13	6	1	28	4	24	15	23	18	8
Corvidae	*Cracticus torquatus*				10	1	13	1	9	2	12	2	8	1	15	1	13	2	16	3	9
Corvidae	*Cracticus louisiadensis*			x	8	45	0x	0	0x	0	0x	85	0x	60	0x	85	0x	0	0x	85	0x
Corvidae	*Cracticus nigrogularis*		H		10	0	15	0	10	0	13	0	7	0	17	0	15	0	18	0	9
Corvidae	*Gymnorhina tibicen*		H		8,10,11,12,13	0	13	0	9	0	11	0	7	0	15	0	13	0	15	0	8
Corvidae	*Strepera graculina*		H		10	0	18	0	10	0	15	0	6	0	24	0	16	0	21	0	8
Corvidae	*Strepera fuliginosa*		H		10	0	39	0	14	0	34	0	14	0	40	0	14	0	34	0	14
Corvidae	*Strepera versicolor*				10	1	12	2	7	2	11	3	6	4	13	2	9	4	13	5	7
Corvidae	*Artamus fuscus*				7	20	7	24	6	22	7	25	7	23	9	27	9	26	11	34	9
Corvidae	*Artamus leucorynchus*				7,8,9,10,11	5	11	7	8	8	10	8	6	8	14	10	12	14	14	14	7
Corvidae	*Artamus monachus*				7	15	13	26	9	17	13	17	13	16	14	29	21	27	27	23	25
Corvidae	*Artamus maximus*				8	6	1	5	1	19	1	13	0	11	6	13	7	31	7	27	3
Corvidae	*Artamus insignis*			x	8	1	0x	2	0x	45	0x	32	0x	27	0x	19	0x	72	0x	55	0x
Corvidae	*Artamus mentalis*			x	14	22	6x	21	6x	31	0x	44	0x	26	6x	42	6x	80	0x	78	0x
Corvidae	*Artamus personatus*				10,12	0	12	0	10	0	11	0	9	1	14	0	11	1	13	1	9
Corvidae	*Artamus superciliosus*				10,12	0	12	0	10	0	11	0	9	1	14	0	12	1	13	1	9
Corvidae	*Artamus cinereus*				7,8,10	0	10	0	9	0	10	0	8	0	11	0	11	0	11	0	9
Corvidae	*Artamus cyanopterus*		H		10	0	14	0	8	0	13	0	5	0	17	0	9	0	16	0	6
Corvidae	*Artamus minor*				10	0	16	0	13	1	15	1	11	0	19	0	18	1	19	2	13
Corvidae	*Pityriasis gymnocephala*				7	8	0	13	1	12	2	14	1	16	1	18	1	21	2	23	1
Corvidae	*Peltops blainvillii*				8	4	2	6	2	15	2	15	1	11	3	14	4	26	3	23	1
Corvidae	*Peltops montanus*				8	6	1	5	1	19	1	13	0	11	6	13	8	32	7	28	3
Corvidae	*Oriolus melanotis*			x	7	10	31x	3	31x	11	23x	8	23x	10	31x	3	32x	11	23x	8	23x
Corvidae	*Oriolus bouroensis*			x	7	8	4x	7	4x	11	1x	24	5x	14	4x	7	4x	22	1x	24	5x
Corvidae	*Oriolus forsteni*			x	7	11	0x	11	0x	20	0x	20	0x	29	0x	11	0x	22	0x	44	0x
Corvidae	*Oriolus phaeochromus*			x	7	14	0x	17	0x	28	0x	24	0x	32	0x	17	0x	45	0x	34	0x

Family	Species	IUCN	Ag	R	Range Source	2050 TG H	2050 TG C	2050 AM H	2050 AM C	2050 GO H	2050 GO C	2050 OS H	2050 OS C	2100 TG H	2100 TG C	2100 AM H	2100 AM C	2100 GO H	2100 GO C	2100 OS H	2100 OS C
						Percentage loss in geographic range															
Corvidae	*Oriolus szalayi*				8	4	3	6	2	16	2	15	1	10	5	13	5	26	4	24	2
Corvidae	*Oriolus sagittatus*				8,10	2	20	3	11	4	17	5	6	4	26	3	20	6	26	7	9
Corvidae	*Oriolus flavocinctus*				7,8,10	0	24	0	14	0	24	2	8	0	26	0	23	1	27	2	11
Corvidae	*Oriolus xanthonotus*				7	13	2	16	3	15	3	18	3	20	3	22	3	28	3	28	3
Corvidae	*Oriolus steerii*				7	21	14	26	16	13	15	26	17	31	17	27	16	27	21	39	18
Corvidae	*Oriolus albiloris*				7	6	7	20	3	10	9	15	6	6	17	33	10	22	22	27	6
Corvidae	*Oriolus isabellae*	T			7	6	8	20	4	10	9	16	5	6	19	32	12	21	22	28	6
Corvidae	*Oriolus oriolus*				3,4,5,6,7	12	15	11	14	13	16	15	12	16	18	15	17	20	19	25	14
Corvidae	*Oriolus auratus*				5	39	1	24	1	41	1	46	1	44	1	28	5	47	1	47	1
Corvidae	*Oriolus chinensis*				7	11	3	12	3	12	3	12	3	13	4	14	4	15	4	15	4
Corvidae	*Oriolus tenuirostris*				7	28	11	32	10	28	13	30	12	33	13	35	14	34	16	37	14
Corvidae	*Oriolus chlorocephalus*			x	5	26	0x	17	0x	26	0x	26	0x	26	0x	26	0x	26	0x	26	0x
Corvidae	*Oriolus crassirostris*	T		x	5	0⊦02x		0⊦02x		100	0x	100	0x	0⊦02x		0⊦02x		100	0x	100	0x
Corvidae	*Oriolus brachyrhynchus*				5	34	5	21	8	31	6	52	4	50	6	31	9	57	7	61	5
Corvidae	*Oriolus monacha*				5	42	0	31	1	42	0	42	0	42	0	35	7	42	0	42	0
Corvidae	*Oriolus larvatus*				5	38	1	30	2	39	1	41	1	40	2	32	6	41	1	41	1
Corvidae	*Oriolus percivali*				5	31	4	23	8	36	3	32	0	31	4	27	8	36	3	32	0
Corvidae	*Oriolus nigripennis*				5	35	6	20	8	32	6	55	4	51	6	30	9	56	7	63	5
Corvidae	*Oriolus xanthornus*				7	17	5	20	5	19	5	22	5	20	8	22	8	22	10	30	6
Corvidae	*Oriolus hosii*				7	14	0	17	2	7	3	12	2	21	0	20	2	16	3	24	2
Corvidae	*Oriolus cruentus*				7	15	2	16	3	14	3	18	3	20	2	21	3	27	3	27	3
Corvidae	*Oriolus traillii*				7	31	9	34	8	31	10	34	9	35	12	37	12	36	14	40	12
Corvidae	*Oriolus mellianus*	T			7	1	0	1	0	1	0	1	0	1	0	1	0	1	0	1	0
Corvidae	*Sphecotheres hypoleucus*			x	7	25	18x	0	18x	0	18x	18	0x	25	18x	0	18x	0	18x	18	0x
Corvidae	*Sphecotheres viridis*				7,8,10	2	28	4	16	7	23	6	8	3	36	5	30	8	35	11	11
Corvidae	*Sphecotheres vieilloti*				8	6	1	6	6	31	6x	50	6x	17	9	12	15	71	15x	56	6x
Corvidae	*Coracina maxima*				10	0	11	0	8	1	9	0	6	0	13	0	12	1	13	1	8
Corvidae	*Coracina larvata*				7	14	2	15	3	11	2	17	2	19	2	20	4	25	2	24	2
Corvidae	*Coracina macei*				7	15	4	17	3	16	4	19	3	17	6	19	6	19	7	26	4
Corvidae	*Coracina javensis*				7	3	0	0	0	0	0	0	0	5	0	2	2	4	0	2	0
Corvidae	*Coracina schistacea*			x	7	27	8x	27	5x	13	19x	1	19x	27	13x	29	11x	14	25x	1	25x
Corvidae	*Coracina personata*				7	7	11	6	12	6	8	6	10	7	18	6	20	11	12	11	10
Corvidae	*Coracina atriceps*				7	18	0	25	0	21	0	22	0	35	0	26	0	35	0	41	0
Corvidae	*Coracina fortis*			x	7	8	0x	8	0x	8	0x	13	0x	16	0x	8	0x	21	0x	13	0x
Corvidae	*Coracina caledonica*				9	14	10	17	5	26	5	27	4	27	13	27	5	47	6	44	4
Corvidae	*Coracina novaehollandiae*				8,10,12	1	13	1	9	2	12	2	7	2	16	1	13	2	16	3	9
Corvidae	*Coracina caeruleogrisea*				8	6	2	6	3	19	2	18	1	10	6	12	7	34	6	31	2
Corvidae	*Coracina temminckii*				7	16	13	28	10	20	14	17	12	18	15	31	24	30	33	25	29
Corvidae	*Coracina striata*				7	13	3	16	4	15	4	18	3	19	4	22	4	28	5	28	4
Corvidae	*Coracina bicolor*				7	14	13	28	9	19	13	16	12	16	14	31	21	28	29	24	27
Corvidae	*Coracina lineata*				8,9,10	7	10	8	7	18	9	21	5	15	15	15	13	29	14	31	7
Corvidae	*Coracina boyeri*				8	4	3	6	3	16	2	15	1	10	5	13	6	26	4	24	2
Corvidae	*Coracina leucopygia*		H		7	0	13	0	10	0	13	0	13	0	14	0	20	0	27	0	25
Corvidae	*Coracina papuensis*				7,8,9,10	4	11	6	6	12	9	12	3	9	15	9	10	19	13	18	4

Family	Species	IUCN	Ag	R	Range Source	Percentage loss in geographic range															
						2050								2100							
						TG		AM		GO		OS		TG		AM		GO		OS	
						H	C	H	C	H	C	H	C	H	C	H	C	H	C	H	C
Corvidae	Coracina longicauda				8	7	0	4	0	20	0	14	0	11	7	13	8	33	8	31	4
Corvidae	Coracina parvula			x	7	14	0x	17	0x	28	0x	24	0x	31	0x	17	0x	45	0x	34	0x
Corvidae	Coracina abbotti				7	14	13	26	9	19	13	16	11	16	14	29	22	28	31	24	27
Corvidae	Coracina analis			x	9	14	29x	45	8x	24	17x	16	19x	14	29x	55	11x	26	20x	20	19x
Corvidae	Coracina pectoralis				5	35	2	24	4	37	2	42	1	40	3	27	8	43	3	43	2
Corvidae	Coracina caesia				5	26	3	22	5	28	3	29	2	28	5	24	7	30	3	30	2
Corvidae	Coracina azurea				5	33	6	21	7	29	6	58	4	51	7	32	9	61	6	66	4
Corvidae	Coracina graueri				5	41	4	29	7	26	12	50	8	48	4	33	7	37	12	50	8
Corvidae	Coracina cinerea				6,13	57	0	27	8	57	0	57	0	57	0	39	8	57	0	57	0
Corvidae	Coracina typica	T		x	13	0	0x	0	0x	0	0x	0	0x	0	0x	0	0x	0	0x	0	0x
Corvidae	Coracina newtoni	T		x	13	49	0x	49	0x	49	0x	49	0x	49	0x	49	0x	49	0x	49	0x
Corvidae	Coracina coerulescens				7	8	9	23	5	16	10	15	7	8	18	36	11	27	22	25	8
Corvidae	Coracina tenuirostris				7,8,9,10,11	6	11	9	8	13	8	14	6	13	16	12	12	19	14	22	8
Corvidae	Coracina dohertyi			x	7	21	7x	24	15x	8	15x	0	15x	21	8x	24	16x	8	16x	25	15x
Corvidae	Coracina sula			x	7	16	3x	14	0x	17	0x	0	0x	16	10x	17	7x	18	7x	0	7x
Corvidae	Coracina dispar			x	14	20	0x	0	0x	20	0x	82	0x	20	0x	0	0x	20	0x	82	0x
Corvidae	Coracina mindanensis	T			7	15	11	23	11	13	12	22	12	20	18	30	14	25	21	34	13
Corvidae	Coracina morio				7	14	13	27	9	19	12	16	11	16	14	30	21	29	29	24	26
Corvidae	Coracina ceramensis				7	16	0	19	0	19	0	20	0	30	0	19	0	26	0	35	0
Corvidae	Coracina incerta				8	7	1	6	1	18	1	12	0	11	5	12	6	29	6	28	2
Corvidae	Coracina schisticeps				8	6	1	8	1	12	1	14	1	11	4	15	5	23	5	21	1
Corvidae	Coracina melas				8	4	3	6	3	16	2	15	1	10	5	13	6	26	4	24	2
Corvidae	Coracina montana				8	7	1	5	0	20	0	12	0	11	6	12	8	33	7	31	3
Corvidae	Coracina holopolia				9	10	6	8	2	22	1	23	2	28	11x	19	2	55	3x	40	2x
Corvidae	Coracina mcgregori				7	18	14	25	18	8	18	17	19	28	17	25	18	24	22	34	19
Corvidae	Coracina ostenta	T		x	7	34	21x	36	20x	0	27x	6	21x	34	21x	36	20x	0	27x	34	21x
Corvidae	Coracina polioptera				7	16	12	19	12	15	14	17	14	19	17	20	19	20	22	25	19
Corvidae	Coracina melaschistos				7	13	3	14	3	13	4	15	3	14	4	16	4	15	4	17	4
Corvidae	Coracina fimbriata				7	13	2	16	3	15	3	18	2	19	3	21	3	27	3	27	2
Corvidae	Coracina melanoptera				7	21	3	24	2	24	2	30	1	23	3	28	3	28	4	46	1
Corvidae	Campochaera sloetii				8	4	1	8	0	15	0	15	1	12	4	16	4	27	3	26	1
Corvidae	Lalage melanoleuca				7	14	10	23	10	13	12	21	11	20	17	29	14	25	20	34	12
Corvidae	Lalage nigra		H		7	0	4	0	5	0	5	0	5	0	6	0	6	0	6	0	5
Corvidae	Lalage leucopygialis		H		7	0	13	0	9	0	13	0	13	0	14	0	20	0	26	0	25
Corvidae	Lalage sueurii				7,10	1	12	2	9	2	11	2	8	2	14	2	12	3	14	3	9
Corvidae	Lalage tricolor			x	8,12	0	9x	1	9x	33	9x	45	9x	4	16x	5	17x	60	17x	52	10x
Corvidae	Lalage aurea				7	21	0	28	0	24	0	23	0	37	0x	28	0	42	0x	39	0x
Corvidae	Lalage moesta			x	7	7	16x	7	16x	20	3x	66	23x	7	16x	7	16x	28	3x	66	23x
Corvidae	Lalage atrovirens				8	6	3	7	2	17	2	12	2	16	4	16	3	26	2	24	2
Corvidae	Lalage leucomela				8,10	2	17	4	10	14	13	14	5	6	21	7	18	21	21	21	7
Corvidae	Lalage maculosa				9,11	12	4	14	4	24	1	26	1x	24	4x	30	4x	56	1x	54	1x
Corvidae	Lalage sharpei			x	11	0	0x	0	0x	2	0x	0	0x	47	0x	0	0x	2	0x	2	0x
Corvidae	Lalage leucopyga			x	9	15	20x	27	12x	30	14x	30	10x	20	20x	37	13x	36	15x	49	10x
Corvidae	Campephaga petiti				5	35	5	23	7	29	6	51	3	45	6	32	8	53	6	56	4

Family	Species	IUCN	Ag	R	Range Source	Percentage loss in geographic range 2050								2100							
						TG		AM		GO		OS		TG		AM		GO		OS	
						H	C	H	C	H	C	H	C	H	C	H	C	H	C	H	C
Corvidae	*Campephaga flava*				5,8	42	2	31	5	42	2	46	1	44	3	34	9	45	3	46	2
Corvidae	*Campephaga phoenicea*				5	28	4	14	7	30	4	39	3	36	4	20	10	40	6	42	4
Corvidae	*Campephaga quiscalina*				14	33	4	22	5	32	5	47	3	43	5	31	6	49	5	51	3
Corvidae	*Campephaga lobata*	T			14	29	4	16	2	19	5	38	2	36	6	22	7	35	6	40	3
Corvidae	*Campephaga oriolina*				14	31	4	23	4	32	7	59	5	51	4	38	5	59	8	68	5
Corvidae	*Pericrocotus roseus*				7	27	5	30	4	27	6	30	5	30	7	33	6	31	7	34	6
Corvidae	*Pericrocotus cantonensis*				7	4	0	4	0	4	0	4	0	4	0	5	0	4	0	4	0
Corvidae	*Pericrocotus divaricatus*				7	24	7	23	7	23	8	22	8	25	10	24	12	25	11	25	11
Corvidae	*Pericrocotus tegimae*			x	7	5	18x	6	18x	3	18x	5	18x	5	19x	10	18x	24	18x	9	19x
Corvidae	*Pericrocotus cinnamomeus*		H		7	0	5	0	5	0	5	0	4	0	8	0	9	0	12	0	6
Corvidae	*Pericrocotus igneus*				7	13	3	16	3	16	3	18	3	20	4	22	4	29	4	28	3
Corvidae	*Pericrocotus lansbergei*				7	19	8x	13	13x	4	13x	6	8x	19	26x	13	31x	18	18x	23	8x
Corvidae	*Pericrocotus erythropygius*				7	11	2	10	2	12	2	15	1	12	5	11	8	13	8	22	1
Corvidae	*Pericrocotus solaris*				7	15	4	18	4	16	5	18	5	18	6	20	6	21	7	23	6
Corvidae	*Pericrocotus ethologus*				7	27	3	28	3	26	4	27	4	29	5	30	5	29	5	30	5
Corvidae	*Pericrocotus brevirostris*				7	26	4	28	4	25	5	27	4	29	6	30	6	28	7	30	6
Corvidae	*Pericrocotus miniatus*				7	21	5	22	5	21	3	23	6	26	6	29	6	41	3	35	6
Corvidae	*Pericrocotus flammeus*				7	19	4	22	4	20	4	24	4	23	5	25	6	27	7	32	5
Corvidae	*Hemipus picatus*				7	21	6	25	5	23	7	26	6	25	8	28	8	29	10	34	8
Corvidae	*Hemipus hirundinaceus*				7	13	1	15	2	15	2	15	2	20	3	21	3	26	2	25	2
Corvidae	*Rhipidura hypoxantha*				7	35	8	37	7	34	10	37	9	38	11	41	10	39	12	43	11
Corvidae	*Rhipidura cyaniceps*				7	10	9	23	6	14	10	15	7	10	19	35	13	24	22	27	9
Corvidae	*Rhipidura phoenicura*				7	5	0	0	0	0	0	0	0	7	0	5	0	5	0	5	1
Corvidae	*Rhipidura nigrocinnamome*				7	19	14	25	18	8	18	17	19	29	17	25	18	24	22	34	19
Corvidae	*Rhipidura albicollis*				7	21	5	24	4	22	5	26	4	24	6	27	6	28	6	34	6
Corvidae	*Rhipidura euryura*				7	7	0	0	0	0	0	0	0	8	0	5	0	6	0	5	1
Corvidae	*Rhipidura aureola*		H		7	0	4	0	4	0	4	0	3	0	6	0	8	0	10	0	5
Corvidae	*Rhipidura javanica*				7	11	5	15	5	13	5	15	5	16	9	19	9	22	11	22	8
Corvidae	*Rhipidura perlata*				7	13	2	16	3	16	3	19	2	20	3	22	3	29	3	28	3
Corvidae	*Rhipidura leucophrys*		H		8,9,10	0	11	0	9	0	10	0	7	0	13	0	12	0	13	0	8
Corvidae	*Rhipidura diluta*			x	7	15	9x	10	12x	4	12x	6	7x	15	23x	10	26x	15	15x	19	7x
Corvidae	*Rhipidura rufiventris*				7,8,10	2	12	3	8	9	11	8	4	6	14	7	12	14	14	13	6
Corvidae	*Rhipidura fuscorufa*			x	7	7	23x	7	23x	28	2x	52	30x	7	23x	7	23x	40	2x	52	30x
Corvidae	*Rhipidura cockerelli*				9	13	6	12	1	22	1	26	1	29	10	22	1	52	2	41	1
Corvidae	*Rhipidura threnothorax*				8	4	2	6	2	17	2	16	1	12	4	13	5	29	4	25	2
Corvidae	*Rhipidura maculipectus*				8	3	4	3	5	9	2	14	2	6	5	10	8	15	5	20	3
Corvidae	*Rhipidura leucothorax*				8	4	2	6	1	16	1	15	1	10	4	13	5	27	4	24	2
Corvidae	*Rhipidura atra*				8	7	0	5	0	20	0	12	0	12	6	12	8	32	7	32	3
Corvidae	*Rhipidura hyperythra*				8	4	2	6	2	16	2	14	1	10	4	13	5	26	4	24	2
Corvidae	*Rhipidura albolimbata*				8	7	1	4	1	19	1	14	0	11	7	13	9	31	8	32	4
Corvidae	*Rhipidura phasiana*				8,10	0	22	1	11	1	21	2	8	0	23	1	19	1	25	2	10
Corvidae	*Rhipidura fuliginosa*				8,9,10,12,13	2	16	3	8	6	13	6	6	5	20	5	14	8	20	9	8
Corvidae	*Rhipidura drownei*			x	9	7	0x	7	0x	5	0x	18	0x	17	0x	7	0x	39	0x	58	0x
Corvidae	*Rhipidura tenebrosa*			x	9	47	0x	26	0x	27	0x	74	0x	47	0x	26	0x	27	0x	100	0x

Family	Species	IUCN	Ag	R	Range Source	Percentage loss in geographic range 2050 TG H		AM H		GO H		OS H		2100 TG H		AM H		GO H		OS H	
						H	C	H	C	H	C	H	C	H	C	H	C	H	C	H	C
Corvidae	*Rhipidura rennelliana*			x	9	0	0x	0	0x	93	0x	93	0x	93	0x	0	0x	93	0x	93	0x
Corvidae	*Rhipidura spilodera*		H		9,11	0	13	0	9	0	7	0	5	0	13	0	9	0	7	0	5
Corvidae	*Rhipidura personata*			x	11	0	37x	0	37x	63	37x	63	37x	0	37x	0	37x	63	37x	63	37x
Corvidae	*Rhipidura nebulosa*			x	11	0	0x	0	0x	0	0x	0	0x	39	0x	0	0x	0	0x	0	0x
Corvidae	*Rhipidura brachyrhyncha*				8	6	0	4	0	19	0	14	0	11	6	14	8	31	8	33	4
Corvidae	*Rhipidura teysmanni*				7	14	13	26	9	19	13	15	12	16	15	29	21	28	29	23	27
Corvidae	*Rhipidura superflua*			x	7	9	0x	8	0x	9	0x	12	0x	16	0x	8	0x	21	0x	12	0x
Corvidae	*Rhipidura dedemi*			x	7	16	0x	19	0x	21	0x	21	0x	34	0x	19	0x	25	0x	44	0x
Corvidae	*Rhipidura opistherythra*			x	7	7	17x	7	17x	20	3x	67	23x	7	17x	7	17x	26	3x	67	23x
Corvidae	*Rhipidura rufidorsa*				8	4	2	6	2	16	2	15	1	11	4	13	5	27	4	24	2
Corvidae	*Rhipidura dahli*				8	4	0	6	0	44	0x	48	0x	24	0	25	0	68	0x	60	0x
Corvidae	*Rhipidura matthiae*			x	8	0	0x	0	0x	0	0x	0	0x	0	0x	0	0x	0	0x	0	0x
Corvidae	*Rhipidura malaitae*	T		x	9	0	0x	0	0x	60	0x	0	0x	41	0x	0	0x	70	0x	10	0x
Corvidae	*Rhipidura rufifrons*				7,8,9,10,11	6	14	8	8	12	11	14	5	11	18	11	13	18	15	21	7
Corvidae	*Rhipidura semirubra*	T		x	8	29	0x	0	0x	61	0x	84	0x	84	0x	0	0x	91	0x	84	0x
Corvidae	*Chaetorhynchus papuensis*				8	6	1	6	1	19	1	14	0	10	6	13	7	31	6	27	3
Corvidae	*Dicrurus ludwigii*				5	33	4	22	5	33	5	45	3	42	5	28	9	46	7	48	5
Corvidae	*Dicrurus atripennis*				5	33	5	19	7	30	6	53	4	51	5	30	9	59	7	64	4
Corvidae	*Dicrurus adsimilis*				4,5	27	3	16	7	29	3	33	2	31	4	19	11	33	5	34	4
Corvidae	*Dicrurus modestus*				5	35	5	23	7	34	6	55	3	51	6	33	8	59	7	63	4
Corvidae	*Dicrurus fuscipennis*	T		x	13	0	0x	0	0x	0	0x	0	0x	0	0x	0	0x	0	0x	0	0x
Corvidae	*Dicrurus forficatus*				6,13	54	0	29	6	54	0	54	0	54	0	39	6	54	0	54	0
Corvidae	*Dicrurus waldenii*	T		x	13	100	0x	0	0x	100	0x	100	0x	100	0x	100	0x	100	0x	100	0x
Corvidae	*Dicrurus macrocercus*				4,7,11	13	3	15	3	14	3	16	3	14	5	16	5	16	7	20	4
Corvidae	*Dicrurus leucophaeus*				7	14	4	16	3	15	4	16	4	16	5	18	5	19	6	20	5
Corvidae	*Dicrurus caerulescens*				7	15	2	16	2	16	1	20	1	16	3	18	3	19	3	30	1
Corvidae	*Dicrurus annectans*				7	34	3	42	1	37	1	41	1	34	3	45	2	41	1	48	1
Corvidae	*Dicrurus aeneus*				7	18	5	21	5	20	6	22	5	21	8	25	7	27	9	31	7
Corvidae	*Dicrurus remifer*				7	23	8	25	7	22	8	25	8	27	10	28	11	30	12	32	11
Corvidae	*Dicrurus hottentottus*				7,8,10	15	7	17	5	16	7	18	5	17	9	20	9	21	10	23	6
Corvidae	*Dicrurus balicassius*				7	9	9	22	5	13	10	17	7	9	19	34	13	24	22	28	9
Corvidae	*Dicrurus montanus*				7	14	14	26	9	19	13	16	12	16	15	30	22	29	30	24	27
Corvidae	*Dicrurus sumatranus*				7	23	4	24	4	24	3	25	4	30	6	33	6	44	4	38	4
Corvidae	*Dicrurus densus*				7	14	15x	10	17x	7	14	12	13	14	26x	10	29x	16	17x	20	13x
Corvidae	*Dicrurus megarhynchus*			x	8	0	0x	6	0x	49	0x	31	0x	37	0x	7	0x	64	0x	45	0x
Corvidae	*Dicrurus bracteatus*				7,8,9	7	3	9	2	18	2	17	2	15	5	15	5	30	5	29	3
Corvidae	*Dicrurus andamanensis*			x	7	10	0x	10	0x	10	0x	10	0x	10	0x	20	0x	20	0x	20	0x
Corvidae	*Dicrurus paradiseus*		H		7	0	4	0	4	0	4	0	3	0	6	0	6	0	7	0	5
Corvidae	*Stenostira scita*				5	4	8	3	9	4	10	4	8	4	10	3	11	5	12	5	11
Corvidae	*Erythrocercus mccallii*				5	32	7	19	8	29	7	56	5	50	7	30	9	56	8	64	5
Corvidae	*Erythrocercus holochlorus*				5	8	0	6	2	8	0	8	0	8	0	6	2	8	0	8	0
Corvidae	*Erythrocercus livingstonei*				5	43	0	37	0	43	0	43	0	43	1	39	4	43	1	43	1
Corvidae	*Elminia longicauda*				5	33	4	20	6	34	5	50	3	45	5	28	9	53	7	55	4
Corvidae	*Elminia albicauda*				5	51	2	35	2	52	2	57	1	55	3	38	6	55	2	57	1

Family	Species	IUCN	Ag	R	Range Source	Percentage loss in geographic range 2050 TG H	TG C	AM H	AM C	GO H	GO C	OS H	OS C	2100 TG H	TG C	AM H	AM C	GO H	GO C	OS H	OS C
Corvidae	*Elminia nigromitrata*				5	33	5	23	6	32	5	57	4	51	6	34	8	62	5	66	5
Corvidae	*Elminia albiventris*				5	35	9	26	15	37	13	55	2	44	15	29	18	51	13	58	5
Corvidae	*Elminia albonotata*				5	44	1	41	3	44	1	45	0	45	1	43	5	45	1	45	0
Corvidae	*Trochocercus nitens*				5	34	6	24	8	32	7	55	4	51	7	34	9	59	7	63	5
Corvidae	*Trochocercus cyanomelas*				5	33	1	27	1	34	1	36	1	36	2	29	4	36	1	37	1
Corvidae	*Hypothymis helenae*				7	12	8	21	5	16	8	24	5	19	14	30	9	28	17	36	6
Corvidae	*Hypothymis coelestis*	T			7	14	11	23	10	14	12	22	11	20	17	30	13	26	20	34	12
Corvidae	*Hypothymis azurea*				7	15	5	17	4	16	5	18	4	18	7	20	7	21	8	26	6
Corvidae	*Eutrichomyias rowleyi*	T		x	7	0	0x	0	0x	0	0x	0	0x	0	0x	0	0x	0	0x	0	0x
Corvidae	*Terpsiphone rufiventer*				5	35	7	23	10	34	8	56	5	51	8	31	12	57	9	62	6
Corvidae	*Terpsiphone bedfordi*				5	23	10	18	10	13	13	30	12	31	10	26	10	19	13	30	12
Corvidae	*Terpsiphone rufocinerea*				5	54	3	33	4	51	1	66	1	62	4	41	5	68	1	72	1
Corvidae	*Terpsiphone viridis*				4,5	31	3	20	5	31	4	39	2	37	4	25	8	40	5	42	3
Corvidae	*Terpsiphone atrochalybeia*		H	x	5	0	102x	0	102x	0	0x	0	0x	0	102x	0	102x	0	0x	0	0x
Corvidae	*Terpsiphone mutata*				6,13	66	1	34	9	67	1	67	1	67	1	50	9	67	1	67	1
Corvidae	*Terpsiphone bourbonnensi*			x	13	38	0x	38	0x	38	0x	38	0x	38	0x	38	0x	38	0x	38	0x
Corvidae	*Terpsiphone paradisi*				7	12	3	14	3	13	4	15	3	14	5	16	5	17	6	20	4
Corvidae	*Terpsiphone atrocaudata*				7	6	15	10	13	9	15	8	14	7	24	14	28	14	29	14	26
Corvidae	*Terpsiphone cinnamomea*				7	15	11	24	11	13	12	22	12	21	17	30	14	25	21	34	13
Corvidae	*Terpsiphone cyanescens*			x	7	24	25x	24	29x	23	20x	28	18x	24	25x	24	48x	45	39x	38	47x
Corvidae	*Chasiempis sandwichensis*	T			11	6	3	20	8x	20	9x	28	5x	36	3x	59	8x	53	9x	84	5x
Corvidae	*Mayrornis versicolor*	T		x	11	0	0x	0	0x	0	0x	0	0x	100	0x	0	0x	0	0x	0	0x
Corvidae	*Mayrornis lessoni*				11	19	6	17	6	27	1	41	1	21	6	38	6	77	1	75	1
Corvidae	*Mayrornis schistaceus*			x	9	0	0x	100	0x	0	0x	0	0x	0	0x	100	0x	0	0x	0	0x
Corvidae	*Neolalage banksiana*			x	9	6	0x	25	0x	34	0x	7	0x	21	0x	42	0x	45	0x	40	0x
Corvidae	*Clytorhynchus pachycephal*			x	9	8	23x	25	14x	28	16x	19	12x	13	23x	37	15x	36	17x	37	12x
Corvidae	*Clytorhynchus vitiensis*				11	19	6	17	6	27	1	40	1	21	6	37	6	75	1	73	1
Corvidae	*Clytorhynchus nigrogularis*	T		x	9,11	18	6x	18	6x	26	1x	39	1x	23	6x	38	6x	73	1x	71	1x
Corvidae	*Clytorhynchus hamlini*			x	9	0	0x	0	0x	93	0x	93	0x	93	0x	0	0x	93	0x	93	0x
Corvidae	*Monarcha axillaris*				8	4	1	4	1	20	1	11	0	8	7	12	8	34	7	31	3
Corvidae	*Monarcha rubiensis*				8	3	4	8	1	12	1	10	2	12	4	14	4	22	1	24	2
Corvidae	*Monarcha cinerascens*				7,8,9	15	3	17	3	22	3	20	4	28	3	19	4	33	4	30	4
Corvidae	*Monarcha frater*				8,10	5	2	6	1	17	2	13	0	10	6	13	7	28	6	24	2
Corvidae	*Monarcha melanopsis*				8,10	10	15	15	11	20	12	22	9	13	21	18	16	27	17	35	12
Corvidae	*Monarcha erythrostictus*			x	9	9	0x	9	0x	11	0x	12	0x	20	0x	9	0x	40	0x	37	0x
Corvidae	*Monarcha castaneiventris*			x	9	15	5x	15	1x	28	0x	34	1x	34	11x	29	1x	58	1x	51	1x
Corvidae	*Monarcha richardsii*			x	9	35	18x	25	0x	23	7x	48	0x	35	18x	31	0x	39	7x	48	0x
Corvidae	*Monarcha pileatus*				7	13	0	14	0	22	0	21	0	27	0	14	0	39	0x	28	0
Corvidae	*Monarcha castus*			x	7	9	16x	9	16x	23	3x	61	25x	9	16x	9	16x	31	3x	61	25x
Corvidae	*Monarcha leucotis*				10	3	27	4	21	10	24	14	14	6	34	4	29	14	33	15	18
Corvidae	*Monarcha guttulus*				8	4	3	6	3	17	2	15	1	11	5	13	5	27	4	25	2
Corvidae	*Monarcha mundus*			x	7	17	14x	8	14x	18	2x	56	20x	17	14x	8	14x	24	2x	56	20x
Corvidae	*Monarcha trivirgatus*				7,8,10	7	18	11	13	12	16	13	7	11	22	11	21	16	23	22	10
Corvidae	*Monarcha sacerdotum*	T		x	7	18	8x	30	19x	1	19x	0	19x	18	8x	30	19x	1	19x	22	19x

Family	Species	IUCN	Ag	R	Range Source	Percentage loss in geographic range 2050 TG H	C	AM H	C	GO H	C	OS H	C	2100 TG H	C	AM H	C	GO H	C	OS H	C
Corvidae	*Monarcha everetti*	T		x	7	0	⊦02x	0	⊦02x	0	⊦02x	0	⊦02x	0	⊦02x	0	⊦02x	0	⊦02x	0	⊦02x
Corvidae	*Monarcha loricatus*		H	x	7	0	0x	0	0x	0	0x	0	0x	0	0x	0	0x	0	0x	0	0x
Corvidae	*Monarcha boanensis*	T		x	14	100	0x	100	0x	0	0x	100	0x	100	0x	100	0x	0	0x	100	0x
Corvidae	*Monarcha leucurus*			x	13	19	0x	0	0x	19	0x	80	0x	19	0x	0	0x	19	0x	80	0x
Corvidae	*Monarcha julianae*			x	8	0	0x	0	0x	0	0x	0	0x	0	0x	0	0x	0	0x	0	0x
Corvidae	*Monarcha manadensis*				8	4	2	6	2	13	2	14	2	9	3	13	4	24	3	22	2
Corvidae	*Monarcha brehmii*	T		x	8	0	5x	0	5x	0	5x	0	5x	0	5x	0	5x	0	5x	0	5x
Corvidae	*Monarcha infelix*			x	8	34	0x	0	0x	63	0x	90	0x	90	0x	0	0x	97	0x	90	0x
Corvidae	*Monarcha menckei*			x	8	0	0x	0	0x	0	0x	0	0x	0	0x	0	0x	0	0x	0	0x
Corvidae	*Monarcha verticalis*				8	5	0	7	0	50	0	43	0	26	0	23	0	71	0	56	0
Corvidae	*Monarcha barbatus*				9	10	4	10	2	21	0	20	2	28	9x	21	2	54	2x	39	2x
Corvidae	*Monarcha browni*			x	9	42	25x	17	0x	16	7x	44	0x	42	25x	26	0x	43	7x	44	0x
Corvidae	*Monarcha viduus*			x	9	47	0x	34	0x	40	0x	76	0x	47	0x	34	0x	40	0x	99	0x
Corvidae	*Monarcha chrysomela*				8	4	3	6	3	17	2	15	1	11	5	13	5	27	4	25	2
Corvidae	*Arses telescophthalmus*				8,10	4	4	3	4	13	3	13	1	7	6	9	7	23	6	20	3
Corvidae	*Arses kaupi*				10	0	31x	0	25x	0	32x	5	24x	0	46x	0	44x	0	61x	5	48x
Corvidae	*Myiagra atra*			x	8	0	3x	0	3x	0	3x	0	3x	0	3x	0	3x	0	3x	0	3x
Corvidae	*Myiagra galeata*				7	17	0	22	0	21	0	21	0	32	0	23	0	33	0	37	0
Corvidae	*Myiagra rubecula*				8,10	2	21	3	11	4	17	5	6	4	26	4	20	6	25	8	8
Corvidae	*Myiagra ferrocyanea*				9	13	6	12	1	22	1	25	1	29	10	22	1	52	2	40	1
Corvidae	*Myiagra cervinicauda*			x	9	47	0x	33	0x	40	0x	77	0x	47	0x	33	0x	40	0x	99	0x
Corvidae	*Myiagra caledonica*				9	9	18x	27	8x	34	10x	24	5x	15	18x	45	8x	44	10x	43	5x
Corvidae	*Myiagra vanikorensis*			x	9,11	22	5x	21	5x	30	0x	45	0x	24	5x	41	5x	76	0x	75	0x
Corvidae	*Myiagra albiventris*	T		x	11	0	0x	0	0x	2	0x	0	0x	47	0x	0	0x	2	0x	2	0x
Corvidae	*Myiagra azureocapilla*				11	28	5	21	5	33	0	44	0	29	5	50	5	83	0x	82	0x
Corvidae	*Myiagra ruficollis*				7,8,10	0	24	1	16	2	23	2	8	1	25	2	23	2	24	3	8
Corvidae	*Myiagra cyanoleuca*				8,10,12	9	14	13	5	18	9	19	4	16	18	17	7	25	12	31	5
Corvidae	*Myiagra inquieta*				8,10	2	18	2	11	4	15	4	7	3	22	3	18	5	22	7	10
Corvidae	*Myiagra alecto*				8,10	2	13	2	8	9	10	9	3	5	15	5	10	14	14	13	4
Corvidae	*Myiagra hebetior*				8	5	0	7	0	49	0	42	0	26	0	22	0	69	0	54	0
Corvidae	*Lamprolia victoriae*	T		x	11	13	12x	0	12x	25	0x	55	0x	13	12x	0	12x	89	0x	89	0x
Corvidae	*Machaerirhynchus flavivent*				8,10	4	5	5	4	15	4	14	2	10	7	12	8	25	7	23	4
Corvidae	*Machaerirhynchus nigripect*				8	7	1	3	1	20	1	13	0	10	7	11	9	34	8	31	3
Corvidae	*Grallina cyanoleuca*		H		8,10	0	14	0	10	0	12	0	8	0	16	0	14	0	16	0	10
Corvidae	*Grallina bruijni*			x	8	2	3x	26	0x	7	0x	17	3x	3	7x	28	4x	12	3x	29	4x
Corvidae	*Aegithina tiphia*				7	20	6	23	6	21	7	25	6	24	8	27	9	28	10	35	8
Corvidae	*Aegithina nigrolutea*				7	12	7	16	6	11	7	16	5	12	11	16	14	12	19	19	8
Corvidae	*Aegithina viridissima*				7	13	3	16	4	15	4	18	3	19	4	22	4	28	4	27	4
Corvidae	*Aegithina lafresnayei*				7	16	11	19	11	16	13	19	12	20	15	21	17	22	19	27	17
Corvidae	*Lanioturdus torquatus*				5	21	4	14	4	20	2	19	4	21	6	16	8	20	5	19	6
Corvidae	*Nilaus afer*				5	27	3	17	6	28	3	32	2	31	4	20	9	33	4	33	3
Corvidae	*Dryoscopus gambensis*				5	30	4	16	7	34	5	45	3	40	5	23	11	46	7	47	5
Corvidae	*Dryoscopus pringlii*				5	46	5	16	23	52	0	52	0	47	5	19	26	52	0	52	0
Corvidae	*Dryoscopus cubla*				5	40	2	30	2	40	2	44	1	43	3	32	7	43	3	44	2

Family	Species	IUCN	Ag	R	Range Source	Percentage loss in geographic range 2050 TG H	C	AM H	C	GO H	C	OS H	C	2100 TG H	C	AM H	C	GO H	C	OS H	C
Corvidae	*Dryoscopus senegalensis*				5	31	5	19	8	30	6	60	4	53	5	31	8	58	6	71	4
Corvidae	*Dryoscopus angolensis*				5	40	4	32	8	45	2	50	0	42	5	37	8	47	3	51	0
Corvidae	*Dryoscopus sabini*				5	35	4	18	8	27	7	50	6	56	5	30	9	58	8	62	7
Corvidae	*Tchagra minuta*				5	32	2	22	3	34	2	40	1	37	3	27	7	39	2	42	1
Corvidae	*Tchagra senegala*				3,4,5	20	2	13	4	21	2	24	2	23	3	15	7	25	4	25	3
Corvidae	*Tchagra australis*				5	23	4	17	6	23	4	24	3	24	5	19	11	24	6	25	5
Corvidae	*Tchagra jamesi*				5	36	5	8	28	42	1	42	1	38	5	9	31	42	2	42	1
Corvidae	*Tchagra tchagra*				5	0	5	0	5	0	5	0	5	0	7	0	10	0	10	0	10
Corvidae	*Laniarius ruficeps*				5	12	1	9	0	14	0	14	0	13	1	10	1	14	0	14	0
Corvidae	*Laniarius luehderi*				5	31	5	21	7	28	6	50	4	47	5	31	7	49	7	58	4
Corvidae	*Laniarius brauni*	T		x	14	64	0x	64	0x	64	0x	64	0x	64	0x	64	0x	64	0x	64	0x
Corvidae	*Laniarius amboimensis*	T		x	14	0	0x	0	0x	0	0x	0	0x	0	0x	0	0x	0	0x	0	0x
Corvidae	*Laniarius liberatus*	T		x	5	0	0x	0	0x	0	0x	0	0x	0	0x	0	0x	0	0x	0	0x
Corvidae	*Laniarius turatii*				5	13	0	22	0	29	0	33	0	20	0	26	0	35	0	33	0
Corvidae	*Laniarius aethiopicus*				5	37	3	23	8	37	4	44	3	41	5	28	13	43	6	44	4
Corvidae	*Laniarius bicolor*				5	18	1	14	3	19	1	19	1	18	1	15	8	19	1	19	1
Corvidae	*Laniarius ferrugineus*				5	2	3	1	3	2	3	2	3	2	5	2	7	2	5	2	5
Corvidae	*Laniarius barbarus*				5	14	3	7	3	16	3	23	2	21	3	10	6	25	6	25	4
Corvidae	*Laniarius erythrogaster*				5	10	1	8	3	12	0	12	1	11	1	9	5	13	1	13	1
Corvidae	*Laniarius atrococcineus*				5	7	5	2	7	6	5	7	5	7	7	2	15	6	10	7	9
Corvidae	*Laniarius mufumbiri*				5	37	3	24	8	41	0	41	0	37	3	31	11	41	0	41	0
Corvidae	*Laniarius atroflavus*				5	21	4x	9	5	14	3	18	7x	27	9x	19	11x	42	3x	22	7x
Corvidae	*Laniarius funebris*				5	37	3	19	13	41	1	41	1	39	3	22	16	42	1	42	1
Corvidae	*Laniarius leucorhynchus*				5	35	4	22	6	32	5	55	3	52	5	33	8	60	6	65	3
Corvidae	*Laniarius poensis*				5	20	12	9	13	32	6	28	6	25	14	16	16	44	6	29	7
Corvidae	*Laniarius fuelleborni*				5	29	0	28	0	29	0	29	0	29	0	29	8	29	0	29	0
Corvidae	*Rhodophoneus cruentus*				5	39	5	14	19	45	1	45	2	42	5	18	23	46	3	45	3
Corvidae	*Telophorus zeylonus*				5	3	6	2	7	3	7	2	7	3	8	2	11	3	12	3	11
Corvidae	*Telophorus bocagei*				5	35	4	23	6	30	6	55	4	52	5	34	8	56	7	66	4
Corvidae	*Telophorus sulfureopectus*				5	31	2	21	4	33	3	38	2	36	3	25	8	39	4	39	3
Corvidae	*Telophorus olivaceus*				5	11	2	10	2	11	2	11	2	11	4	10	4	12	2	12	2
Corvidae	*Telophorus multicolor*				5	28	6	20	8	26	10	38	6	35	8	29	11	37	11	43	7
Corvidae	*Telophorus nigrifrons*				5	46	0	38	0	48	0	55	0	53	0	41	2	53	0	56	0
Corvidae	*Telophorus kupeensis*	T		x	5	0	20x	0	20x	100	0x	100	0x	0	20x	0	20x	100	0x	100	0x
Corvidae	*Telophorus viridis*				5	33	3	26	2	32	3	39	2	37	5	27	6	37	3	40	3
Corvidae	*Telophorus dohertyi*				5	18	6	13	7	25	3	26	0	20	6	18	10	25	5	26	0
Corvidae	*Malaconotus cruentus*				5	30	5	16	6	27	5	53	3	47	5	27	8	55	6	62	3
Corvidae	*Malaconotus lagdeni*				5	22	6	20	4	23	5	29	0	24	6	25	6	27	8	29	0
Corvidae	*Malaconotus gladiator*	T			5	0	13x	2	11x	44	0x	23	0x	14	24x	2	23x	44	0x	34	0x
Corvidae	*Malaconotus blanchoti*				5	37	1	24	3	40	2	44	1	42	2	28	7	45	2	45	2
Corvidae	*Malaconotus monteiri*			x	5	77	0x	77	0x	77	0x	77	0x	77	0x	77	0x	77	0x	77	0x
Corvidae	*Malaconotus alius*	T		x	5	53	0x	53	0x	53	0x	53	0x	53	0x	53	0x	53	0x	53	0x
Corvidae	*Prionops plumatus*				5	29	2	18	5	30	2	35	2	33	3	21	9	35	4	36	3
Corvidae	*Prionops poliolophus*				5	75	4x	65	19x	81	4x	75	10x	81	4x	65	19x	81	4x	75	10x

Family	Species	IUCN	Ag	R	Range Source	Percentage loss in geographic range 2050 TG H	TG C	AM H	AM C	GO H	GO C	OS H	OS C	2100 TG H	TG C	AM H	AM C	GO H	GO C	OS H	OS C
Corvidae	*Prionops alberti*	T		x	5	23	6x	14	13x	24	14x	29	2x	30	6x	17	13x	28	15x	29	2x
Corvidae	*Prionops caniceps*				5	26	8	14	10	27	7	32	7	32	9	19	14	36	8	34	9
Corvidae	*Prionops rufiventris*				14	32	3	22	5	29	5	56	4	52	4	32	6	55	6	65	4
Corvidae	*Prionops retzii*				5	38	1	28	1	38	1	40	1	40	2	29	6	39	1	40	1
Corvidae	*Prionops gabela*	T		x	5	30	0x	26	0x	30	0x	30	0x	30	0x	30	0x	30	0x	30	0x
Corvidae	*Prionops scopifrons*				5	36	0	26	0	36	0	36	0	36	0	30	0	36	0	36	0
Corvidae	*Bias flammulatus*				14	37	6	26	8	35	6	60	4	53	7	36	10	61	7	65	4
Corvidae	*Bias musicus*				5	39	5	28	6	38	5	55	3	51	6	36	9	57	6	61	4
Corvidae	*Pseudobias wardi*				6	75	0	45	4	78	0	78	0	78	0	71	4	78	0	78	0
Corvidae	*Batis diops*				5	26	6	19	9	29	4	32	0	27	6	26	10	34	6	32	0
Corvidae	*Batis margaritae*				5	60	0	42	0	57	0	60	0	60	0	47	0	57	0	60	0
Corvidae	*Batis mixta*				5	26	0	26	0	27	0	27	0	26	2	26	3	27	0	27	0
Corvidae	*Batis capensis*				5	19	1	17	1	19	1	19	1	19	2	18	2	19	1	20	1
Corvidae	*Batis fratrum*				5	44	0	40	0	44	0	44	0	44	1	40	2	45	0	45	0
Corvidae	*Batis molitor*				5	37	1	30	3	37	1	40	1	39	3	32	7	39	2	40	2
Corvidae	*Batis soror*				5	50	0	43	0	50	0	50	0	50	1	46	2	50	0	50	0
Corvidae	*Batis pririt*				5	8	7	2	9	7	7	7	7	8	10	2	15	7	13	7	12
Corvidae	*Batis senegalensis*				5	14	4	8	4	17	4	24	2	21	4	11	8	25	7	25	5
Corvidae	*Batis orientalis*				5	19	2	8	6	22	1	22	1	21	2	11	9	23	2	23	2
Corvidae	*Batis minor*				5	32	4	17	7	32	5	43	3	40	5	22	11	44	7	45	4
Corvidae	*Batis perkeo*				5	60	7	14	41	70	2	70	2	65	7	19	48	70	2	70	2
Corvidae	*Batis minima*				5	31	14x	27	14x	27	14x	76	13x	44	14x	46	14x	63	14x	80	13x
Corvidae	*Batis ituriensis*				5	41	9	27	13	34	11	66	8	59	9	33	13	58	11	74	8
Corvidae	*Batis poensis*				5	20	1	22	1	22	3	39	0	41	2	32	1	42	3	50	2
Corvidae	*Batis minulla*				5	49	2	37	3	44	2	55	0	50	4	45	5	51	4	57	0
Corvidae	*Platysteira cyanea*				5	33	5	20	8	33	7	52	4	48	6	28	12	54	9	58	5
Corvidae	*Platysteira peltata*				5	48	2	36	2	49	2	54	1	52	3	39	6	53	3	55	2
Corvidae	*Platysteira albifrons*				5	50	1	46	1	52	0	52	1	50	1	49	1	52	0	52	1
Corvidae	*Platysteira castanea*				14	37	6	24	8	34	7	57	4	52	6	33	11	59	8	64	4
Corvidae	*Platysteira tonsa*				14	30	4	21	5	28	5	55	4	51	5	32	5	56	5	65	4
Corvidae	*Platysteira blissetti*				14	32	5	21	7	30	6	56	4	50	6	30	8	54	6	63	4
Corvidae	*Platysteira concreta*				14	36	4	23	5	30	5	53	3	49	5	33	6	53	5	59	3
Corvidae	*Philentoma pyrhopterum*				7	13	2	16	3	15	3	18	2	20	3	22	3	28	3	28	2
Corvidae	*Philentoma velatum*				7	12	3	15	3	14	3	17	3	19	4	21	4	26	4	26	4
Corvidae	*Tephrodornis pondicerianu*				7	20	8	23	7	20	8	24	7	22	10	26	12	25	14	35	11
Corvidae	*Calicalicus madagascarien*				6	54	0	27	9	54	0	54	0	54	0	36	9	54	0	54	0
Corvidae	*Schetba rufa*				6	69	1	37	10	71	1	71	1	71	1	51	10	71	1	71	1
Corvidae	*Vanga curvirostris*				6	54	0	29	7	54	0	54	0	54	0	39	7	54	0	54	0
Corvidae	*Xenopirostris xenopirostris*				6	20	0	3	1	20	0	20	0	20	0	9	1	20	0	20	0
Corvidae	*Xenopirostris damii*	T		x	6	0	0x	0	0x	0	0x	0	0x	0	0x	0	0x	0	0x	0	0x
Corvidae	*Xenopirostris polleni*				6	75	0	45	4	78	0	78	0	78	0	71	4	78	0	78	0
Corvidae	*Falculea palliata*				6	44	0	18	9	44	0	44	0	44	0	24	9	44	0	44	0
Corvidae	*Artamella viridis*				6	54	0	30	6	54	0	54	0	54	0	40	6	54	0	54	0
Corvidae	*Leptopterus chabert*		H		6	0	0	0	8	0	0	0	0	0	0	0	8	0	0	0	0

Family	Species	IUCN	Ag	R	Range Source	2050 TG H	2050 TG C	2050 AM H	2050 AM C	2050 GO H	2050 GO C	2050 OS H	2050 OS C	2100 TG H	2100 TG C	2100 AM H	2100 AM C	2100 GO H	2100 GO C	2100 OS H	2100 OS C
Corvidae	*Cyanolanius madagascarin*				6,13	59	1	29	10	60	1	60	1	60	1	38	10	60	1	60	1
Corvidae	*Oriolia bernieri*	T			6	89	0x	50	1x	96	0x	96	0x	96	0x	95	1x	96	0x	96	0x
Corvidae	*Euryceros prevostii*	T			6	89	0x	50	1x	96	0x	96	0x	96	0x	95	1x	96	0x	96	0x
Corvidae	*Tylas eduardi*				6	75	0	45	5	78	0	78	0	78	0	70	5	78	0	78	0
Corvidae	*Hypositta corallirostris*				6	73	0	43	4	77	0	77	0	77	0	71	4	77	0	77	0
Callaeatidae	*Callaeas cinerea*	T		x	12,13	18	10x	23	10x	33	5x	39	6x	24	10x	31	10x	33	6x	43	6x
Callaeatidae	*Philesturnus carunculatus*			x	12,13	0	0x	0	0x	0	0x	0	0x	0	0x	0	30x	0	30x	0	0x
Picathartidae	*Chaetops frenatus*				5	0	0	0	0	0	0	0	0	0	0	0	0	0	0	0	0
Picathartidae	*Picathartes gymnocephalu*	T			5	37	4	22	0	28	4	53	0	43	5	36	2	51	4	55	0
Picathartidae	*Picathartes oreas*	T			5	32	6	18	4	40	3	52	3	51	8	30	7	56	3	62	3
Bombycillidae	*Dulus dominicus*			x	2	28	33x	34	21x	33	28x	61	0x	28	33x	34	28x	33	28x	61	0x
Bombycillidae	*Ptilogonys cinereus*				2	15	9	26	5	16	7	31	3	19	9	27	17	21	13	34	7
Bombycillidae	*Ptilogonys caudatus*			x	2	13	17x	27	27x	12	27x	19	27x	21	17x	27	34x	49	34x	48	27x
Bombycillidae	*Phainopepla nitens*				2	4	26	8	24	4	26	10	23	5	29	8	37	5	40	12	35
Bombycillidae	*Phainoptila melanoxantha*			x	2	18	13x	41	19x	7	19x	28	19x	26	13x	41	20x	35	20x	49	19x
Bombycillidae	*Bombycilla garrulus*				2,3,4,7	1	14	1	14	1	14	1	14	1	17	2	19	2	20	3	19
Bombycillidae	*Bombycilla japonica*				7	0	6	0	6	0	6	0	6	0	7	0	7	0	7	1	7
Bombycillidae	*Bombycilla cedrorum*		H		2	0	20	0	14	0	17	0	12	0	24	0	20	0	23	0	16
Muscicapidae	*Neocossyphus finschii*				5	25	6	13	8	22	6	31	4	30	7	20	11	33	7	34	6
Muscicapidae	*Neocossyphus fraseri*				5	40	6	27	9	38	6	59	4	55	7	36	10	61	7	66	4
Muscicapidae	*Neocossyphus rufus*				5	32	6	20	8	27	8	52	3	49	6	32	9	52	9	63	4
Muscicapidae	*Neocossyphus poensis*				5	34	6	24	8	35	6	54	4	50	6	35	9	60	7	62	4
Muscicapidae	*Pseudocossyphus sharpei*				6	73	0	43	4	77	0	77	0	77	0	71	4	77	0	77	0
Muscicapidae	*Pseudocossyphus bensoni*			x	6	52	0x	23	0x	52	0x	52	0x	52	0x	52	0x	52	0x	52	0x
Muscicapidae	*Pseudocossyphus imerinus*			x	6	32	0x	13	0x	32	0x	32	0x	32	0x	15	0x	32	0x	32	0x
Muscicapidae	*Monticola rupestris*				5	2	1	2	2	2	3	2	2	2	4	2	5	2	4	2	4
Muscicapidae	*Monticola explorator*				5	4	0	4	0	4	1	4	0	4	1	4	2	4	1	4	1
Muscicapidae	*Monticola brevipes*				5	6	1	3	2	5	2	5	1	6	4	3	7	5	6	5	4
Muscicapidae	*Monticola angolensis*				5	53	1	42	2	54	1	57	0	56	2	45	5	56	1	58	1
Muscicapidae	*Monticola saxatilis*				3,4,5,7	10	13	10	13	11	14	12	12	15	19	13	18	18	20	18	16
Muscicapidae	*Monticola rufocinereus*				4,5	24	0	16	2	25	0	25	0	25	0	18	4	25	0	25	0
Muscicapidae	*Monticola cinclorhynchus*				7	69	4	71	2	70	2	76	1	71	7	77	4	77	4	79	3
Muscicapidae	*Monticola gularis*				7	12	4	11	5	11	5	11	5	13	5	12	6	12	6	12	6
Muscicapidae	*Monticola rufiventris*				7	23	3	24	2	23	3	24	3	25	4	26	3	25	4	26	4
Muscicapidae	*Monticola solitarius*				3,4,5,7,8	14	6	15	6	16	6	17	6	17	8	17	9	19	9	21	8
Muscicapidae	*Myophonus blighi*	T		x	7	28	0x	26	0x	28	0x	28	0x	28	0x	26	0x	28	0x	28	0x
Muscicapidae	*Myophonus melanurus*				7	24	6	25	6	24	4	27	6	29	6	33	7	47	4	40	6
Muscicapidae	*Myophonus glaucinus*				7	12	2	15	2	14	2	16	2	19	2	21	3	26	2	26	2
Muscicapidae	*Myophonus robinsoni*	T			7	17	8	20	8	21	8	38	8	21	9	26	8	41	8	45	8
Muscicapidae	*Myophonus horsfieldii*				7	29	4	35	2	36	2	42	1	31	4	41	2	40	3	67	1
Muscicapidae	*Myophonus caeruleus*				7	18	4	20	4	18	4	20	4	20	5	22	6	23	6	24	6
Muscicapidae	*Myophonus insularis*				7	0	0	0	0	0	0	0	0	0	0	0	0	0	0	0	0
Muscicapidae	*Geomalia heinrichi*				7	14	14	28	9	19	14	17	11	17	15	31	23	29	33	26	28
Muscicapidae	*Zoothera schistacea*			x	7	7	18x	7	18x	22	2x	64	24x	7	18x	7	18x	30	2x	64	24x

Family	Species	IUCN	Ag	R	Range Source	2050 TG H	2050 TG C	2050 AM H	2050 AM C	2050 GO H	2050 GO C	2050 OS H	2050 OS C	2100 TG H	2100 TG C	2100 AM H	2100 AM C	2100 GO H	2100 GO C	2100 OS H	2100 OS C
Muscicapidae	*Zoothera dumasi*				7	14	0	15	0	17	0	18	0	29	0x	15	0	24	0x	34	0x
Muscicapidae	*Zoothera interpres*				7	13	2	15	3	14	3	17	3	19	3	21	4	27	3	27	3
Muscicapidae	*Zoothera erythronota*				7	14	14	26	9	19	13	16	12	16	15	30	22	29	30	24	27
Muscicapidae	*Zoothera dohertyi*			x	7	12	14x	9	16x	7	14x	9	13x	12	30x	9	34x	19	18x	20	13x
Muscicapidae	*Zoothera wardii*				7	72	5	74	3	76	2	81	1	77	7	82	4	84	3	84	3
Muscicapidae	*Zoothera cinerea*	T			7	7	8	20	5	13	9	17	6	7	19	33	11	23	22	27	8
Muscicapidae	*Zoothera peronii*			x	7	14	30x	4	30x	10	23x	8	23x	14	30x	4	30x	10	23x	8	23x
Muscicapidae	*Zoothera citrina*		H		7	0	5	0	4	0	4	0	4	0	7	0	7	0	8	0	6
Muscicapidae	*Zoothera everetti*				7	17	1	26	2	18	5	26	2	28	1	31	3	37	5	43	2
Muscicapidae	*Zoothera sibirica*				4,7	6	5	6	5	6	6	6	6	7	7	6	8	7	8	7	8
Muscicapidae	*Zoothera naevia*				2	2	25	4	23	3	24	4	23	3	33	5	36	6	38	8	34
Muscicapidae	*Zoothera pinicola*				2	12	11	32	5	16	10	42	5	19	13	34	13	23	19	49	11
Muscicapidae	*Zoothera piaggiae*				5	28	2	16	8	28	2	30	1	29	2	19	9	30	2	31	1
Muscicapidae	*Zoothera crossleyi*				5	24	5	27	6	34	9	52	4	35	5	32	10	42	10	52	5
Muscicapidae	*Zoothera gurneyi*				5	37	0	32	2	37	0	37	0	37	0	34	5	37	0	37	0
Muscicapidae	*Zoothera oberlaenderi*				5	46	12	26	17	27	23	51	15x	52	12x	37	17	41	23x	51	15x
Muscicapidae	*Zoothera cameronensis*				5	35	2	21	4	43	1	62	2	58	2	27	4	61	1	69	2
Muscicapidae	*Zoothera princei*				5	32	5	21	7	30	8	51	7	46	5	31	9	52	10	59	8
Muscicapidae	*Zoothera guttata*	T			5	18	0	15	1	15	0	18	0	18	0	16	1	17	0	20	0
Muscicapidae	*Zoothera spiloptera*				7	52	0	55	0	40	3	54	0	53	0	60	0	52	3	63	0
Muscicapidae	*Zoothera andromedae*				7	20	6	21	6	19	5	22	6	25	9	29	9	36	8	34	6
Muscicapidae	*Zoothera mollissima*				7	39	6	41	4	41	3	42	3	41	9	45	10	45	11	44	9
Muscicapidae	*Zoothera dixoni*				7	43	6	44	6	43	7	46	5	47	9	48	10	47	11	50	9
Muscicapidae	*Zoothera dauma*				7,8	12	6	13	6	13	7	13	7	14	8	15	9	16	10	17	9
Muscicapidae	*Zoothera major*	T		x	7	25	7x	7	0x	9	0x	1	7x	31	7x	35	2x	37	0x	1	7x
Muscicapidae	*Zoothera machiki*			x	7	7	23x	7	23x	28	2x	53	30x	7	23x	7	23x	39	2x	53	30x
Muscicapidae	*Zoothera lunulata*				8,9,10	8	14	12	6	18	10	18	5	14	17	17	9	27	14	29	8
Muscicapidae	*Zoothera heinei*				10	12	11	21	9	23	11	28	8	15	15	25	15	31	16	46	13
Muscicapidae	*Zoothera talaseae*				8,9	8	0	8	0	42	0x	27	0x	20	0x	19	0x	63	0x	45	0x
Muscicapidae	*Zoothera margaretae*			x	9	17	0x	15	0x	25	0x	52	0x	30	0x	15	0x	52	0x	85	0x
Muscicapidae	*Zoothera monticola*				7	56	5	57	6	54	8	62	3	62	9	63	9	64	11	69	7
Muscicapidae	*Zoothera marginata*				7	30	9	34	8	31	10	34	9	35	12	37	12	37	14	40	12
Muscicapidae	*Cataponera turdoides*				7	14	6	23	7	24	7	11	9	14	8	27	24	35	29	18	29
Muscicapidae	*Cichlherminia lherminieri*	T		x	2	6	11x	38	11x	17	0x	41	0x	6	11x	38	11x	41	0x	49	0x
Muscicapidae	*Sialia sialis*		H		2,13	0	17	0	9	0	13	0	6	0	20	0	13	0	17	0	8
Muscicapidae	*Sialia mexicana*		H		2	0	17	0	11	0	15	0	10	0	20	0	24	0	29	0	20
Muscicapidae	*Sialia currucoides*				2	1	20	2	17	2	19	3	16	2	24	3	25	4	28	6	22
Muscicapidae	*Myadestes myadestinus*			x	11	3	2x	3	2x	60	5x	38	0x	38	2x	3	2x	60	5x	97	0x
Muscicapidae	*Myadestes lanaiensis*	T		x	11	80	0x	80	0x	94	0x	0	0x	80	6x	94	6x	94	6x	80	6x
Muscicapidae	*Myadestes obscurus*	T		x	11	0	0x	12	0x	12	0x	31	0x	44	0x	69	0x	40	0x	100	0x
Muscicapidae	*Myadestes palmeri*	T		x	11	6	0x	6	0x	77	6x	17	0x	23	0x	6	0x	77	6x	94	0x
Muscicapidae	*Myadestes townsendi*				2	3	22	9	18	5	21	11	18	5	29	11	31	10	35	17	27
Muscicapidae	*Myadestes occidentalis*				2	16	9	29	5	19	8	34	4	21	10	30	16	24	15	37	8
Muscicapidae	*Myadestes elisabeth*			x	2	23	14x	38	19x	4	24x	39	4x	29	14x	38	19x	7	25x	42	4x

Family	Species	IUCN	Ag	R	Range Source	Percentage loss in geographic range 2050 TG H	TG C	AM H	AM C	GO H	GO C	OS H	OS C	2100 TG H	TG C	AM H	AM C	GO H	GO C	OS H	OS C
Muscicapidae	*Myadestes genibarbis*			x	2	10	6x	15	4x	10	7x	23	1x	15	6x	16	5x	10	7x	25	1x
Muscicapidae	*Myadestes coloratus*			x	2	24	1x	7	25x	0	25x	24	1x	24	8x	7	25x	0	32x	92	8x
Muscicapidae	*Myadestes ralloides*				2	13	8	16	5	14	8	24	8	19	10	22	12	28	13	29	10
Muscicapidae	*Myadestes unicolor*				2	13	0	18	0	17	3	25	0	14	0	21	1	18	3	27	1
Muscicapidae	*Cichlopsis leucogenys*				2	16	8	8	13	15	11	9	15	25	12	17	19	31	16	26	25
Muscicapidae	*Entomodestes leucotis*				2	11	3	8	0	11	0	11	4	14	3	11	5	18	6	14	7
Muscicapidae	*Entomodestes coracinus*			x	2	2	2x	6	2x	10	2x	8	0x	6	2x	7	2x	10	2x	11	0x
Muscicapidae	*Catharus gracilirostris*		H	x	2	0	22x	0	32x	0	32x	0	32x	0	22x	0	33x	0	33x	0	32x
Muscicapidae	*Catharus aurantiirostris*				2	16	7	24	6	17	8	28	5	22	9	26	15	23	18	33	11
Muscicapidae	*Catharus fuscater*				2	12	9	14	5	12	9	21	10	19	11	23	13	29	15	28	12
Muscicapidae	*Catharus occidentalis*				2	13	11	31	4	15	8	40	4	17	12	33	13	22	14	45	7
Muscicapidae	*Catharus frantzii*				2	9	4	22	2	14	5	30	1	10	4	24	6	23	5	32	1
Muscicapidae	*Catharus mexicanus*				2	10	2	15	2	11	3	16	2	11	2	16	10	13	3	19	2
Muscicapidae	*Catharus dryas*				2	13	6	18	4	14	6	26	5	18	7	24	9	29	9	32	7
Muscicapidae	*Catharus fuscescens*				2	11	24	18	18	16	21	17	15	15	28	23	23	25	28	26	19
Muscicapidae	*Catharus minimus*				2,7	1	21	1	21	1	21	1	20	1	28	1	34	1	35	1	33
Muscicapidae	*Catharus bicknelli*	T			2	26	24	35	19	32	15	36	20	36	25	46	21	52	16	44	26
Muscicapidae	*Catharus ustulatus*				2	5	19	7	17	7	18	8	16	6	24	10	24	12	26	13	22
Muscicapidae	*Catharus guttatus*				2	5	18	8	16	8	17	8	15	7	23	11	23	12	25	13	21
Muscicapidae	*Catharus mustelinus*				2	17	25	28	13	26	18	28	10	27	29	38	18	46	23	41	13
Muscicapidae	*Platycichla flavipes*				2	22	11	16	14	22	12	26	13	29	17	28	20	38	18	40	20
Muscicapidae	*Platycichla leucops*				2	10	9	11	9	8	11	12	12	16	12	16	15	17	18	17	17
Muscicapidae	*Psophocichla litsipsirupa*				5	19	4	11	7	19	4	19	4	19	6	12	12	19	7	19	7
Muscicapidae	*Turdus pelios*				5	36	5	23	7	36	6	53	4	50	6	30	11	54	8	58	5
Muscicapidae	*Turdus tephronotus*				5	49	3	13	31	55	0	55	0	52	3	18	33	56	0	56	0
Muscicapidae	*Turdus libonyanus*				5	42	1	32	1	42	1	44	1	44	2	34	5	44	1	44	1
Muscicapidae	*Turdus olivaceofuscus*			x	5	0	87x	0	87x	87	0x	87	0x	0	87x	0	87x	87	0x	87	0x
Muscicapidae	*Turdus olivaceus*				5	20	4	12	8	22	4	23	3	22	4	14	10	23	5	23	4
Muscicapidae	*Turdus ludoviciae*	T		x	14	0	0x	0	0x	0	0x	0	0x	0	0x	0	0x	0	0x	0	0x
Muscicapidae	*Turdus helleri*	T		x	14	0	0x	0	0x	0	0x	0	0x	0	0x	0	0x	0	0x	0	0x
Muscicapidae	*Turdus menachensis*	T			4	36	2	27	7	36	2	40	0	39	2	30	9	38	3	40	0
Muscicapidae	*Turdus bewsheri*			x	13	8	0x	8	0x	8	0x	8	0x	8	0x	8	0x	8	0x	8	0x
Muscicapidae	*Turdus hortulorum*				7	4	8	3	9	3	9	3	9	5	10	4	12	5	11	5	11
Muscicapidae	*Turdus unicolor*		H		7	0	5	0	3	0	2	0	1	0	7	0	4	0	4	0	3
Muscicapidae	*Turdus dissimilis*				7	33	4	36	4	33	6	36	3	37	6	38	5	38	7	43	4
Muscicapidae	*Turdus cardis*				7	6	16	5	15	6	17	6	16	7	23	5	28	8	27	8	28
Muscicapidae	*Turdus albocinctus*				7	65	4	68	3	67	2	70	3	68	5	72	5	71	4	72	5
Muscicapidae	*Turdus torquatus*				3,4,5	9	25	8	23	11	23	13	21	13	33	13	34	22	33	19	29
Muscicapidae	*Turdus boulboul*				7	43	6	46	5	44	5	47	5	48	8	51	6	49	7	51	7
Muscicapidae	*Turdus merula*				3,4,7,10,12,13	10	15	11	13	13	15	13	11	14	19	14	18	19	20	19	15
Muscicapidae	*Turdus poliocephalus*				7,8,9,11	14	6	18	6	20	5	18	6	19	9	24	12	37	12	30	11
Muscicapidae	*Turdus rubrocanus*				7	41	3	42	2	41	3	43	2	43	4	45	5	45	5	46	4
Muscicapidae	*Turdus kessleri*				7	32	1	32	3	32	2	33	2	33	3	34	7	35	6	36	4
Muscicapidae	*Turdus feae*	T			7	2	0	2	0	2	0	2	0	2	0	2	0	2	0	2	0

Family	Species	IUCN	Ag	R	Range Source	Percentage loss in geographic range 2050 TG H	TG C	AM H	AM C	GO H	GO C	OS H	OS C	2100 TG H	TG C	AM H	AM C	GO H	GO C	OS H	OS C
Muscicapidae	*Turdus obscurus*				4,7	0	2	0	2	0	2	0	3	0	3	0	3	0	3	0	3
Muscicapidae	*Turdus pallidus*				7	21	8	19	10	19	9	19	10	22	12	20	13	22	12	22	13
Muscicapidae	*Turdus chrysolaus*				7	8	20	6	19	7	22	4	20	9	28	8	34	10	33	21	31
Muscicapidae	*Turdus celaenops*	T		x	7	0	0x	0	0x	0	0x	0	0x	0	0x	0	24x	0	24x	0	24x
Muscicapidae	*Turdus ruficollis*				4,7	2	6	1	6	2	7	2	6	3	7	2	7	3	8	6	7
Muscicapidae	*Turdus naumanni*				4,7	1	12	0	12	0	12	0	12	1	15	1	19	1	20	2	19
Muscicapidae	*Turdus pilaris*				3,4,5,7	4	16	3	15	5	17	4	14	6	19	5	18	9	20	9	17
Muscicapidae	*Turdus iliacus*				3,4,7	2	15	2	14	4	16	3	13	4	18	4	18	6	19	7	17
Muscicapidae	*Turdus philomelos*				3,4,7,10,12,13	5	18	4	16	7	19	6	14	8	22	7	19	12	22	11	18
Muscicapidae	*Turdus mupinensis*				7	6	1	6	1	4	1	5	0	6	1	6	2	7	1	7	1
Muscicapidae	*Turdus viscivorus*				3,4,5,7	7	21	7	19	10	22	9	17	11	25	10	23	16	26	16	21
Muscicapidae	*Turdus aurantius*			x	2	29	0x	29	3x	25	3x	55	3x	55	0x	29	3x	25	3x	55	3x
Muscicapidae	*Turdus plumbeus*				2	17	11	22	10	19	13	33	0	17	15	22	11	19	13	33	0
Muscicapidae	*Turdus chiguanco*		H		2	0	11	0	11	0	12	0	10	0	15	0	17	0	18	0	15
Muscicapidae	*Turdus nigrescens*		H	x	2	0	29x	0	39x	0	39x	0	39x	0	29x	0	39x	0	39x	0	39x
Muscicapidae	*Turdus fuscater*				2	14	5	14	3	10	5	17	4	18	6	21	6	24	9	20	7
Muscicapidae	*Turdus infuscatus*				2	6	2	17	2	10	3	19	2	7	2	18	6	16	3	21	2
Muscicapidae	*Turdus serranus*				2	13	6	14	4	11	6	20	6	18	7	20	8	24	9	22	8
Muscicapidae	*Turdus nigriceps*				2	19	11	19	7	10	11	20	9	22	11	24	10	20	16	27	12
Muscicapidae	*Turdus subalaris*				2	24	6	22	5	36	2	38	3	37	10	41	9	55	5	61	8
Muscicapidae	*Turdus reevei*				2	4	5	0	3	1	6	1	2	4	5	1	7	3	10	2	6
Muscicapidae	*Turdus olivater*				2	10	11	5	16	8	14	6	15	13	17	10	25	13	26	11	28
Muscicapidae	*Turdus maranonicus*				2	0	6	6	0	0	6	0	6	0	6	6	0	0	8	0	6
Muscicapidae	*Turdus fulviventris*				2	14	7	19	6	12	7	31	7	25	11	31	17	40	14	41	12
Muscicapidae	*Turdus rufiventris*				2	8	13	7	9	10	10	13	7	12	18	12	13	18	15	21	11
Muscicapidae	*Turdus falcklandii*		H		2	0	7	0	6	0	7	0	7	0	11	0	14	0	17	0	16
Muscicapidae	*Turdus leucomelas*				2	9	17	6	17	8	17	13	14	13	21	11	21	21	23	24	19
Muscicapidae	*Turdus amaurochalinus*				2	8	9	6	7	9	7	11	6	11	12	11	11	17	12	18	10
Muscicapidae	*Turdus plebejus*				2	16	3	24	6	18	9	29	6	19	3	24	11	27	11	35	6
Muscicapidae	*Turdus ignobilis*				2	6	8	5	8	5	8	9	8	10	9	10	10	13	11	17	10
Muscicapidae	*Turdus lawrencii*				2	4	6	3	6	3	6	5	6	8	7	7	7	9	8	12	7
Muscicapidae	*Turdus obsoletus*				2	13	2	15	5	15	2	13	3	22	4	16	6	20	7	25	5
Muscicapidae	*Turdus fumigatus*				2	7	15	5	15	6	15	13	13	12	18	10	18	22	19	23	16
Muscicapidae	*Turdus hauxwelli*				2	5	9	3	10	3	10	6	10	9	11	8	12	9	12	13	12
Muscicapidae	*Turdus grayi*				2	13	4	19	4	15	5	26	3	16	5	23	15	21	9	31	4
Muscicapidae	*Turdus nudigenis*				2	14	9	10	10	12	9	17	8	18	12	17	15	28	15	29	12
Muscicapidae	*Turdus maculirostris*				2	16	10	9	10	18	13	18	0	17	10	11	10	22	13	26	0
Muscicapidae	*Turdus haplochrous*				2	16	25	6	31	9	27	9	31	24	27	12	51	23	44	17	51
Muscicapidae	*Turdus jamaicensis*			x	2	17	0x	17	5x	11	5x	44	5x	44	0x	17	5x	11	5x	44	5x
Muscicapidae	*Turdus daguae*				14	15	3	14	7	13	4	12	3	24	5	15	7	18	9	24	5
Muscicapidae	*Turdus albicollis*				2	7	10	6	10	7	10	11	10	11	13	11	13	18	13	19	12
Muscicapidae	*Turdus rufopalliatus*				2	15	7	31	5	20	7	41	4	20	9	31	17	28	12	45	7
Muscicapidae	*Turdus graysoni*			x	2	0	0x	0	0x	0	0x	0	0x	0	0x	0	0x	0	0x	0	0x
Muscicapidae	*Turdus swalesi*	T		x	2	0	0x	0	0x	0	0x	0	0x	0	0x	0	0x	0	0x	0	0x

Family	Species	IUCN	Ag	R	Range Source	Percentage loss in geographic range 2050 TG H	C	AM H	C	GO H	C	OS H	C	2100 TG H	C	AM H	C	GO H	C	OS H	C
Muscicapidae	*Turdus migratorius*		H		2	0	19	0	15	0	17	0	13	0	23	0	22	0	25	0	19
Muscicapidae	*Turdus rufitorques*		H		2	0	1	0	1	0	1	0	1	0	1	0	1	0	1	0	1
Muscicapidae	*Chlamydochaera jefferyi*				7	9	0	10	1	7	1	12	0	13	0	12	1	16	1	18	0
Muscicapidae	*Brachypteryx stellata*				7	56	4	59	4	56	5	63	1	61	7	63	7	64	8	67	4
Muscicapidae	*Brachypteryx hyperythra*	T			7	66	2	73	0	71	1	74	1	73	3	80	1	74	2	77	2
Muscicapidae	*Brachypteryx major*	T			7	29	0	52	0	43	0	56	0	32	1	64	0	53	0	88	0
Muscicapidae	*Brachypteryx leucophrys*				7	20	5	21	5	19	6	22	5	22	7	23	8	25	8	26	7
Muscicapidae	*Brachypteryx montana*				7	18	4	20	4	17	4	19	4	20	5	22	5	22	6	24	5
Muscicapidae	*Alethe poliocephala*				5	32	7	21	9	27	8	49	6	47	8	28	11	50	9	56	7
Muscicapidae	*Alethe poliophrys*				5	27	6	23	10	34	6	32	0	28	6	27	10	34	9	32	0
Muscicapidae	*Alethe fuelleborni*				5	41	0	33	0	41	0	41	0	41	3	40	3	41	0	41	0
Muscicapidae	*Alethe choloensis*	T		x	5	53	0x	51	0x	53	0x	53	0x	53	0x	51	1x	53	0x	53	0x
Muscicapidae	*Alethe diademata*				5	35	7	21	9	31	8	54	4	50	7	31	11	56	9	61	5
Muscicapidae	*Empidornis semipartitus*				5	18	1	14	3	20	0	20	0	19	1	16	5	22	0	21	1
Muscicapidae	*Bradornis pallidus*				14	35	3	24	5	37	3	43	2	40	3	27	8	43	4	44	3
Muscicapidae	*Bradornis infuscatus*				14	8	7	2	9	7	7	7	7	8	10	2	17	7	14	7	12
Muscicapidae	*Bradornis mariquensis*				14	8	8	4	11	8	9	8	8	8	12	4	16	8	13	8	13
Muscicapidae	*Bradornis microrhynchus*				14	41	3	20	18	46	1	46	1	43	4	24	21	47	2	47	2
Muscicapidae	*Dioptrornis chocolatinus*				5	33	0x	25	1x	35	0x	35	0x	35	0x	31	4x	35	0x	35	0x
Muscicapidae	*Dioptrornis brunneus*				5	60	0	43	0	60	0	60	0	60	0	45	0	60	0	60	0
Muscicapidae	*Melaenornis edolioides*		H		5	0	3	0	8	0	3	0	2	0	3	0	11	0	4	0	3
Muscicapidae	*Melaenornis pammelaina*		H		5	0	2	0	3	0	2	0	2	0	3	0	8	0	3	0	3
Muscicapidae	*Melaenornis ardesiacus*				5	17	3	19	5	21	3	37	0	29	3	20	5	30	5	37	0
Muscicapidae	*Melaenornis annamarulae*	T		x	5	6	0x	29	0x	7	0x	52	0x	6	0x	29	0x	36	0x	52	0x
Muscicapidae	*Fraseria ocreata*				5	35	6	22	9	33	6	55	4	51	7	33	10	59	7	63	4
Muscicapidae	*Fraseria cinerascens*				5	32	6	19	8	30	7	52	4	48	7	29	12	57	9	61	6
Muscicapidae	*Sigelus silens*		H		5	0	2	0	3	0	3	0	2	0	4	0	5	0	6	0	4
Muscicapidae	*Rhinomyias addita*			x	7	8	0x	7	0x	8	0x	12	0x	15	0x	7	0x	20	0x	12	0x
Muscicapidae	*Rhinomyias oscillans*			x	7	24	7x	26	16x	10	16x	2	16x	24	7x	26	16x	10	16x	27	16x
Muscicapidae	*Rhinomyias brunneata*	T			7	5	0	5	0	5	0	5	0	5	0	5	0	5	0	5	0
Muscicapidae	*Rhinomyias olivacea*				7	13	3	15	3	15	3	16	3	19	4	21	4	27	4	26	3
Muscicapidae	*Rhinomyias umbratilis*				7	13	2	16	2	15	2	18	2	20	3	22	3	28	3	27	2
Muscicapidae	*Rhinomyias ruficauda*				7	15	2	22	4	12	5	20	4	24	3	24	4	26	6	33	4
Muscicapidae	*Rhinomyias colonus*			x	7	34	18x	8	17x	4	21x	13	21x	34	19x	8	19x	12	23x	26	23x
Muscicapidae	*Rhinomyias gularis*				7	10	0	17	4	16	4	26	0	14	0	24	4	36	4	28	0
Muscicapidae	*Rhinomyias insignis*	T			7	6	8	19	4	10	8	15	5	6	18	31	12	20	22	27	6
Muscicapidae	*Rhinomyias albigularis*	T		x	7	0	67x	23	43x	0	67x	0	67x	0	67x	23	43x	0	67x	0	67x
Muscicapidae	*Rhinomyias goodfellowi*				7	18	14	25	18	8	18	17	18	29	17	25	18	24	22	34	18
Muscicapidae	*Muscicapa striata*		H		3,4,5,7	0	19	0	17	0	20	0	16	0	23	0	21	0	24	0	18
Muscicapidae	*Muscicapa gambagae*				4,5	16	2	7	4	17	1	18	1	19	2	8	5	21	3	19	2
Muscicapidae	*Muscicapa griseisticta*				7,8	12	8	10	9	11	10	10	10	12	11	12	12	12	12	14	12
Muscicapidae	*Muscicapa sibirica*				7	12	6	12	6	12	6	12	6	13	8	13	8	13	8	14	8
Muscicapidae	*Muscicapa dauurica*				7	12	5	13	5	13	5	13	5	15	7	15	8	17	8	17	8
Muscicapidae	*Muscicapa randi*	T			7	7	9	18	6	11	9	12	7	7	17	26	13	21	18	20	9

Family	Species	IUCN	Ag	R	Range Source	Percentage loss in geographic range 2050 TG H	C	AM H	C	GO H	C	OS H	C	2100 TG H	C	AM H	C	GO H	C	OS H	C
Muscicapidae	*Muscicapa segregata*			x	7	0	0x	0	0x	0	0x	0	0x	0	15x	0	15x	0	15x	0	0x
Muscicapidae	*Muscicapa ruficauda*				7	68	5	68	4	69	2	76	1	70	7	75	5	78	4	79	3
Muscicapidae	*Muscicapa muttui*				7	21	4	22	4	20	5	19	5	22	5	23	5	22	6	22	6
Muscicapidae	*Muscicapa ferruginea*				7	33	2	34	2	33	2	34	1	36	2	37	3	35	4	36	3
Muscicapidae	*Muscicapa ussheri*				5	26	3	13	1	18	4	37	1	31	5	19	5	35	5	39	1
Muscicapidae	*Muscicapa infuscata*				5	35	5	25	6	35	5	56	4	52	6	36	8	60	6	64	4
Muscicapidae	*Muscicapa boehmi*				5	58	0	48	0	59	0	59	0	60	0	48	5	60	0	60	0
Muscicapidae	*Muscicapa aquatica*				5	20	2	12	4	23	2	27	1	24	2	16	7	28	3	28	2
Muscicapidae	*Muscicapa olivascens*				5	31	5	20	6	28	6	53	4	50	6	30	8	55	6	61	5
Muscicapidae	*Muscicapa lendu*	T		x	5	15	0x	4	0x	15	0x	15	0x	15	0x	15	0x	15	0x	15	0x
Muscicapidae	*Muscicapa adusta*				5	35	1	27	3	34	2	39	1	38	3	30	6	38	2	40	2
Muscicapidae	*Muscicapa epulata*				5	31	7	16	9	27	7	56	6	50	7	30	9	57	7	66	6
Muscicapidae	*Muscicapa sethsmithi*				5	30	8	20	9	28	9	56	5	49	8	30	10	55	9	63	5
Muscicapidae	*Muscicapa comitata*		H		5	0	5	0	8	0	6	0	3	0	6	0	10	0	8	0	4
Muscicapidae	*Muscicapa tessmanni*				5	49	1	35	10	39	11	53	12	70	1	44	10	63	11	63	12
Muscicapidae	*Muscicapa cassini*				5	35	6	23	9	32	8	53	4	49	7	31	12	54	9	60	5
Muscicapidae	*Muscicapa caerulescens*				5	39	3	28	4	39	3	47	2	45	4	33	7	48	4	50	2
Muscicapidae	*Myioparus griseigularis*				5	36	6	26	8	32	8	58	5	53	8	35	10	58	8	65	5
Muscicapidae	*Myioparus plumbeus*				5	34	4	24	5	36	4	47	3	44	5	30	9	48	6	50	4
Muscicapidae	*Humblotia flavirostris*	T		x	13	0	0x	0	0x	0	0x	0	0x	0	0x	0	0x	0	0x	0	0x
Muscicapidae	*Ficedula hypoleuca*		H		3,4,5	0	23	0	20	0	25	0	18	0	27	0	24	0	29	0	22
Muscicapidae	*Ficedula albicollis*		H		3,4,5	0	12	0	10	0	13	0	8	0	14	0	11	0	14	0	9
Muscicapidae	*Ficedula semitorquata*				3,4,5	9	29	7	32	7	32	8	32	11	36	11	40	17	38	19	35
Muscicapidae	*Ficedula zanthopygia*				7	20	3	19	4	19	4	19	4	20	4	20	4	20	4	20	4
Muscicapidae	*Ficedula narcissina*				7	7	14	6	13	6	15	5	14	8	21	9	25	10	25	16	23
Muscicapidae	*Ficedula mugimaki*				7	1	1	1	1	1	2	1	2	1	2	1	2	1	2	2	2
Muscicapidae	*Ficedula hodgsonii*				7	31	6	32	7	30	8	32	6	34	11	35	15	37	14	38	11
Muscicapidae	*Ficedula strophiata*				7	28	3	29	3	28	4	29	3	30	5	31	5	31	6	32	5
Muscicapidae	*Ficedula parva*				3,4,7	2	14	2	14	3	15	2	13	3	16	3	18	5	20	5	18
Muscicapidae	*Ficedula subrubra*	T			7	66	7	71	0	73	0x	70	0	70	7x	79	0x	80	0x	82	0x
Muscicapidae	*Ficedula monileger*				7	30	9	36	7	32	10	34	9	34	11	38	9	38	12	40	11
Muscicapidae	*Ficedula solitaris*				7	18	8	19	8	19	7	23	8	23	8	24	10	35	9	33	10
Muscicapidae	*Ficedula hyperythra*				7	19	5	23	4	20	6	22	5	24	7	26	7	28	8	28	7
Muscicapidae	*Ficedula dumetoria*				7	13	3	15	3	15	3	16	3	19	4	21	4	27	3	26	3
Muscicapidae	*Ficedula rufigula*				7	14	14	26	9	19	13	16	12	16	15	30	22	29	30	24	27
Muscicapidae	*Ficedula buruensis*				7	14	0	15	0	17	0	18	0	29	0x	15	0	24	0x	34	0x
Muscicapidae	*Ficedula basilanica*	T			7	20	12	26	15	12	14	25	16	32	15	26	15	27	19	40	16
Muscicapidae	*Ficedula henrici*	T		x	7	0	0x	0	0x	0	0x	0	0x	0	0x	0	0x	0	0x	0	0x
Muscicapidae	*Ficedula harterti*			x	7	0	0x	0	0x	0	0x	0	0x	0	12x	0	14x	0	12x	0	0x
Muscicapidae	*Ficedula platenae*	T		x	7	19	21x	19	25x	22	12x	18	19x	19	21x	19	48x	48	35x	31	52x
Muscicapidae	*Ficedula crypta*				7	16	14	24	17	10	18	24	15	24	22	24	20	26	22	38	15
Muscicapidae	*Ficedula disposita*			x	14	19	1x	33	1x	11	1x	26	0x	19	26x	38	17x	25	30x	50	6x
Muscicapidae	*Ficedula bonthaina*	T		x	7	2	0x	0	0x	0	0x	0	0x	2	0x	0	0x	0	0x	0	0x
Muscicapidae	*Ficedula westermanni*				7	20	7	23	7	20	8	22	7	23	10	26	10	27	12	28	10

Family	Species	IUCN	Ag	R	Range Source	Percentage loss in geographic range 2050 TG H	C	AM H	C	GO H	C	OS H	C	2100 TG H	C	AM H	C	GO H	C	OS H	C
Muscicapidae	*Ficedula superciliaris*				7	44	6	46	6	43	7	45	7	49	8	50	8	48	9	49	9
Muscicapidae	*Ficedula tricolor*				7	29	4	31	4	29	5	30	4	31	6	33	6	32	7	33	6
Muscicapidae	*Ficedula sapphira*				7	25	4	28	3	25	4	25	4	29	4	30	4	28	5	28	5
Muscicapidae	*Ficedula nigrorufa*				7	33	0	52	4	42	4	55	4	41	0	65	4	51	4	75	4
Muscicapidae	*Ficedula timorensis*			x	7	0	16x	2	16x	12	5x	0	16x	0	16x	2	16x	12	5x	0	16x
Muscicapidae	*Cyanoptila cyanomelana*				4,7	18	11	16	12	16	13	16	12	19	16	18	19	20	18	20	18
Muscicapidae	*Eumyias thalassina*				7	19	5	21	5	20	5	22	5	22	6	24	7	25	8	27	7
Muscicapidae	*Eumyias sordida*			x	7	33	0x	31	0x	33	0x	33	0x	33	0x	32	0x	33	0x	33	0x
Muscicapidae	*Eumyias panayensis*				7	13	12	21	11	13	13	14	12	15	16	25	18	24	23	22	19
Muscicapidae	*Eumyias albicaudata*		H		7	0	0	0	0	0	0	0	0	0	2	0	0	0	1	0	0
Muscicapidae	*Eumyias indigo*				7	14	2	16	2	12	2	18	2	20	2	21	3	26	2	28	3
Muscicapidae	*Niltava grandis*				7	26	9	29	8	26	9	30	9	31	11	33	12	35	14	38	12
Muscicapidae	*Niltava macgrigoriae*				7	26	7	28	7	26	8	28	7	29	9	30	9	30	11	33	9
Muscicapidae	*Niltava davidi*				7	10	3	11	3	11	3	12	2	11	3	11	3	12	4	13	3
Muscicapidae	*Niltava sundara*				7	29	4	30	3	28	4	29	4	32	5	33	4	31	5	32	5
Muscicapidae	*Niltava sumatrana*				7	22	7	23	7	22	6	30	7	28	8	31	8	44	6	41	7
Muscicapidae	*Niltava vivida*				7	32	8	34	7	30	10	32	9	35	11	37	10	35	12	38	11
Muscicapidae	*Cyornis sanfordi*	T			7	13	35	32	16	2	35	31	20	23	36x	37	17	7	36	41	20x
Muscicapidae	*Cyornis hoevelli*				7	15	7	25	7	25	7	12	9	15	8	29	26	37	32	20	31
Muscicapidae	*Cyornis hyacinthinus*			x	7	10	32x	3	32x	11	24x	9	23x	10	32x	3	32x	11	24x	9	23x
Muscicapidae	*Cyornis concretus*				7	17	5	19	5	18	6	22	5	24	7	25	7	30	7	31	6
Muscicapidae	*Cyornis ruckii*	T			7	25	4	26	4	26	3	28	5	31	6	34	6	46	4	40	5
Muscicapidae	*Cyornis herioti*				7	8	8	22	5	15	9	15	6	8	19	35	12	26	21	26	7
Muscicapidae	*Cyornis hainanus*				7	10	7	13	6	11	7	12	7	11	10	14	10	14	13	16	10
Muscicapidae	*Cyornis poliogenys*				7	38	9	38	10	36	13	41	10	45	12	44	14	43	16	49	12
Muscicapidae	*Cyornis unicolor*				7	18	5	22	5	19	6	21	5	23	7	25	7	27	8	28	7
Muscicapidae	*Cyornis rubeculoides*		H		7	0	4	0	4	0	5	0	4	0	7	0	6	0	8	0	6
Muscicapidae	*Cyornis banyumas*				7	18	5	21	5	19	6	21	5	22	7	24	7	25	8	27	7
Muscicapidae	*Cyornis lemprieri*			x	7	23	25x	23	29x	22	20x	28	19x	23	25x	23	47x	45	39x	38	46x
Muscicapidae	*Cyornis superbus*				7	8	0	13	1	12	2	14	1	16	1	18	1	21	2	23	1
Muscicapidae	*Cyornis caerulatus*	T			7	13	2	16	2	16	2	18	2	20	2	22	3	28	3	28	2
Muscicapidae	*Cyornis turcosus*				7	14	2	17	3	17	2	18	2	21	3	23	3	30	3	28	2
Muscicapidae	*Cyornis tickelliae*		H		7	0	5	0	5	0	5	0	5	0	7	0	8	0	10	0	7
Muscicapidae	*Cyornis rufigastra*				7	11	3	13	4	12	3	13	3	17	4	18	6	25	5	22	4
Muscicapidae	*Cyornis omissus*				7	14	14	25	10	17	14	17	13	16	15	29	21	26	28	23	26
Muscicapidae	*Muscicapella hodgsoni*				7	22	6	25	6	23	7	27	6	28	7	30	8	33	8	35	7
Muscicapidae	*Culicicapa ceylonensis*				7	19	5	22	5	21	6	23	5	23	7	26	7	27	8	30	7
Muscicapidae	*Culicicapa helianthea*				7	15	13	26	10	17	13	19	12	18	16	31	18	27	26	28	21
Muscicapidae	*Horizorhinus dohrni*		H	x	5	0	0x	0	0x	0	0x	0	0x	0	0x	0	0x	0	0x	0	0x
Muscicapidae	*Pogonocichla stellata*				5	29	1	25	3	29	1	30	0	29	2	26	4	30	1	30	0
Muscicapidae	*Swynnertonia swynnertoni*	T			5	60	0	59	0	60	0	60	0	60	0	60	7	60	0	60	0
Muscicapidae	*Stiphrornis erythrothorax*				5	34	7	23	9	32	7	55	5	51	7	33	10	59	8	63	5
Muscicapidae	*Sheppardia bocagei*				5	48	1	39	1	50	1	52	1	51	2	41	4	53	1	53	1
Muscicapidae	*Sheppardia cyornithopsis*				5	31	4	20	6	27	5	53	5	50	4	30	6	54	6	62	5

Family	Species	IUCN	Ag	R	Range Source	Percentage loss in geographic range 2050 TG H		AM H		GO H		OS H		2100 TG H		AM H		GO H		OS H	
						H	C	H	C	H	C	H	C	H	C	H	C	H	C	H	C
Muscicapidae	*Sheppardia aequatorialis*				5	29	0	14	13	29	0	29	0	29	0	16	13	29	0	29	0
Muscicapidae	*Sheppardia sharpei*				5	31	0	23	0	31	0	31	0	31	0	28	6	31	0	31	0
Muscicapidae	*Sheppardia gunningi*	T			5	22	0	22	0	22	0	22	0	22	0	22	0	22	0	22	0
Muscicapidae	*Sheppardia gabela*	T		x	5	25	0x	2	0x	25	0x	25	0x	25	0x	2	0x	25	0x	25	0x
Muscicapidae	*Sheppardia montana*	T		x	5	0	0x	0	0x	0	0x	0	0x	0	0x	0	0x	0	0x	0	0x
Muscicapidae	*Sheppardia lowei*	T		x	5	15	0x	15	0x	15	0x	15	0x	15	0x	15	11x	15	0x	15	0x
Muscicapidae	*Erithacus rubecula*				3,4	6	24	6	21	8	25	8	19	10	29	9	25	15	30	14	22
Muscicapidae	*Erithacus akahige*				7	8	18	6	17	7	19	5	18	9	25	10	31	11	30	19	28
Muscicapidae	*Erithacus komadori*			x	7	16	10x	15	5x	14	5x	1	10x	20	10x	35	6x	34	5x	9	10x
Muscicapidae	*Luscinia sibilans*				7	1	3	0	4	0	4	0	4	1	4	1	4	1	4	2	4
Muscicapidae	*Luscinia luscinia*				3,4,5,7	18	20	16	19	20	22	19	18	21	24	19	24	25	25	27	21
Muscicapidae	*Luscinia megarhynchos*				3,4,5,7	8	21	9	16	10	20	11	14	13	27	13	24	23	26	18	19
Muscicapidae	*Luscinia calliope*				7,8	5	7	4	7	4	7	4	7	5	8	4	10	5	10	6	10
Muscicapidae	*Luscinia pectoralis*				7	20	15	22	16	23	16	23	15	22	26	26	33	31	32	32	28
Muscicapidae	*Luscinia svecica*				2,3,4,5,7	4	15	4	15	5	17	4	14	6	18	5	20	7	23	8	20
Muscicapidae	*Luscinia ruficeps*	T			7	0	0	0	0	0	0	0	0	0	0	0	0	0	0	0	0
Muscicapidae	*Luscinia obscura*	T			7	4	0	4	0	4	0	4	0	4	0	8	0	4	0	4	0
Muscicapidae	*Luscinia pectardens*				7	39	9	43	7	44	5	43	6	39	14	47	15	46	14	45	13
Muscicapidae	*Luscinia brunnea*				7	42	5	44	3	44	3	45	3	44	7	49	8	47	9	48	7
Muscicapidae	*Luscinia cyane*				7	8	6	8	6	8	7	8	7	9	8	8	9	9	9	10	9
Muscicapidae	*Tarsiger cyanurus*				3,4,7	10	7	10	7	10	7	10	7	11	9	12	10	12	10	12	10
Muscicapidae	*Tarsiger chrysaeus*				7	43	5	44	4	43	5	46	4	45	7	48	7	48	9	49	7
Muscicapidae	*Tarsiger indicus*				7	45	2	46	1	45	2	48	1	50	3	50	3	51	3	53	2
Muscicapidae	*Tarsiger hyperythrus*				7	53	4	57	3	53	4	59	1	59	5	60	5	59	6	62	2
Muscicapidae	*Tarsiger johnstoniae*				7	0	0	0	0	0	0	0	0	0	0	0	0	0	0	0	0
Muscicapidae	*Irania gutturalis*				3,4,5	9	2	9	1	12	1	17	1	19	2	14	1	19	1	20	1
Muscicapidae	*Cossyphicula roberti*				5	12	13	14	8	27	5	29	3	20	16	17	12	30	7	32	3
Muscicapidae	*Cossypha isabellae*				5	20	16	14	15	21	17	25	12	23	24	21	22	34	17	29	16
Muscicapidae	*Cossypha archeri*				5	40	4	36	14	44	10	71	0	57	4	36	14	50	10	71	0
Muscicapidae	*Cossypha anomala*				5	38	0	29	0	38	0	38	0	38	0	36	4	38	0	38	0
Muscicapidae	*Cossypha caffra*				5	15	3	13	4	16	3	16	2	16	4	14	6	17	5	16	4
Muscicapidae	*Cossypha humeralis*				5	16	3	14	3	16	4	16	3	16	7	15	9	17	5	17	5
Muscicapidae	*Cossypha cyanocampter*				5	31	4	20	7	31	5	52	4	48	5	29	8	52	6	59	4
Muscicapidae	*Cossypha polioptera*				5	42	1	33	5	38	4	54	2	53	2	36	7	57	4	56	3
Muscicapidae	*Cossypha semirufa*				5	45	0	28	1	46	0	46	0	46	0	37	4	46	0	46	0
Muscicapidae	*Cossypha heuglini*				5	49	2	33	4	48	3	54	1	53	3	36	8	53	3	55	2
Muscicapidae	*Cossypha natalensis*				5	43	4	31	5	43	4	50	2	48	5	34	9	49	4	51	3
Muscicapidae	*Cossypha dichroa*				5	2	0	2	0	2	0	2	0	2	1	2	0	3	0	3	0
Muscicapidae	*Cossypha heinrichi*	T			5	79	0x	60	0x	69	0x	88	0x	86	0x	76	0x	88	0x	88	0x
Muscicapidae	*Cossypha niveicapilla*				5	31	5	17	8	32	6	49	4	43	6	25	12	49	9	52	6
Muscicapidae	*Cossypha albicapilla*				5	18	1	9	2	20	1	25	0	24	1	12	3	28	1	26	0
Muscicapidae	*Xenocopsychus ansorgei*				5	50	0	44	1	50	0	50	0	50	0	45	1	50	0	50	0
Muscicapidae	*Cichladusa arquata*		H		5	0	2	0	3	0	2	0	2	0	3	0	9	0	2	0	2
Muscicapidae	*Cichladusa ruficauda*		H		5	0	1	0	3	0	0	0	0	0	1	0	4	0	0	0	0

Family	Species	IUCN	Ag	R	Range Source	Percentage loss in geographic range 2050								2100							
						TG		AM		GO		OS		TG		AM		GO		OS	
						H	C	H	C	H	C	H	C	H	C	H	C	H	C	H	C
Muscicapidae	*Cichladusa guttata*				5	43	4	18	21	48	1	48	1	45	4	22	24	49	1	49	1
Muscicapidae	*Cercotrichas leucosticta*				5	34	8	23	11	32	9	57	8	53	8	30	12	55	9	62	9
Muscicapidae	*Cercotrichas quadrivirgata*				5	26	2	21	3	26	1	26	1	26	3	22	9	26	3	26	3
Muscicapidae	*Cercotrichas barbata*				5	53	1	35	2	53	1	59	1	58	2	37	7	57	2	60	1
Muscicapidae	*Cercotrichas signata*				5	1	0	1	0	1	0	1	0	1	1	1	1	1	0	1	0
Muscicapidae	*Cercotrichas hartlaubi*				5	35	2	24	5	37	0	38	0	35	2	29	9	38	1	38	0
Muscicapidae	*Cercotrichas leucophrys*				5	38	3	24	8	38	3	43	2	41	4	27	12	42	4	44	3
Muscicapidae	*Cercotrichas galactotes*				3,4,5	6	3	5	3	7	3	10	2	10	4	7	5	11	5	12	4
Muscicapidae	*Cercotrichas paena*				5	8	5	2	7	7	6	7	5	8	8	2	14	7	11	7	10
Muscicapidae	*Cercotrichas coryphaeus*				5	2	5	1	5	2	6	1	5	2	5	1	6	2	7	2	7
Muscicapidae	*Cercotrichas podobe*				4,5	9	4	4	4	8	4	15	3	13	5	6	8	14	8	15	5
Muscicapidae	*Namibornis herero*			x	5	13	0x	2	0x	13	0x	13	0x	13	0x	2	0x	13	0x	13	0x
Muscicapidae	*Copsychus albospecularis*				6	49	0	27	5	50	0	50	0	50	0	37	5	50	0	50	0
Muscicapidae	*Copsychus saularis*		H		7	0	4	0	3	0	4	0	3	0	6	0	6	0	7	0	5
Muscicapidae	*Copsychus malabaricus*				7,11	18	5	22	5	20	6	23	5	22	8	26	8	27	9	33	7
Muscicapidae	*Copsychus stricklandii*				7	6	2	30	2	16	2	36	2	7	2	41	2	30	2	43	2
Muscicapidae	*Copsychus luzoniensis*				7	9	9	24	5	15	10	15	8	9	17	37	11	26	22	26	9
Muscicapidae	*Copsychus niger*			x	7	23	25x	23	28x	22	20x	27	18x	23	25x	23	48x	44	40x	38	46x
Muscicapidae	*Copsychus cebuensis*	T		x	7	0	10x	7	3x	0	10x	0	10x	0	10x	7	3x	0	10x	0	10x
Muscicapidae	*Trichixos pyrropyga*				7	13	2	16	3	16	3	19	2	20	3	22	3	29	3	29	2
Muscicapidae	*Saxicoloides fulicata*		H		7	0	3	0	3	0	3	0	2	0	5	0	7	0	9	0	2
Muscicapidae	*Phoenicurus alaschanicus*				7	13	5	11	15	14	12	16	5	18	5	13	15	22	12x	29	5x
Muscicapidae	*Phoenicurus erythronota*				4,7	4	11	2	13	3	13	3	13	4	14	3	15	6	15	9	15
Muscicapidae	*Phoenicurus caeruleoceph*				7	19	14	20	15	21	15	21	14	21	18	22	20	26	20	29	19
Muscicapidae	*Phoenicurus ochruros*		H		3,4,5,7	0	14	0	12	0	14	0	11	0	18	0	19	0	22	0	17
Muscicapidae	*Phoenicurus phoenicurus*		H		3,4,5,7	0	19	0	17	0	20	0	16	0	23	0	21	0	24	0	19
Muscicapidae	*Phoenicurus hodgsoni*		H		7	0	9	0	9	0	9	0	8	0	18	0	24	0	21	0	19
Muscicapidae	*Phoenicurus schisticeps*				7	28	9	31	9	30	9	31	8	30	19	34	26	38	23	38	20
Muscicapidae	*Phoenicurus auroreus*				7	19	6	19	6	20	7	20	6	20	10	21	12	23	11	24	10
Muscicapidae	*Phoenicurus moussieri*				3,4	10	4	10	4	9	4	10	4	10	4	10	4	10	4	10	4
Muscicapidae	*Phoenicurus erythrogaster*				3,4,7	9	18	10	18	10	18	10	17	11	26	13	31	17	32	17	30
Muscicapidae	*Phoenicurus frontalis*				7	21	9	24	8	24	9	25	8	23	15	26	20	31	18	32	16
Muscicapidae	*Chaimarrornis leucocephal*				7	25	3	26	3	25	4	25	3	26	5	27	5	27	5	28	5
Muscicapidae	*Rhyacornis fuliginosus*				7	8	2	9	1	8	1	8	1	8	2	9	3	9	2	9	2
Muscicapidae	*Rhyacornis bicolor*	T		x	7	0	0x	1	0x	9	0x	9	0x	0	9x	1	9x	10	0x	9	0x
Muscicapidae	*Hodgsonius phaenicuroide*				7	31	8	33	7	32	8	34	7	33	13	37	16	37	17	38	14
Muscicapidae	*Cinclidium leucurum*				7	17	6	19	5	18	6	20	6	20	7	21	8	21	9	24	8
Muscicapidae	*Cinclidium diana*				7	22	6	21	6	19	4	23	6	27	6	29	7	40	4	34	6
Muscicapidae	*Cinclidium frontale*				7	32	13	35	10	36	13	36	14	36	15	38	11	44	14	46	15
Muscicapidae	*Grandala coelicolor*				7	30	11	33	10	33	10	34	9	32	22	36	30	42	29	42	24
Muscicapidae	*Enicurus scouleri*				7	7	1	8	0	8	0	7	0	8	1	8	2	9	1	8	2
Muscicapidae	*Enicurus velatus*			x	7	7	3x	7	1x	8	1x	7	2x	8	3x	10	1x	16	1x	11	2x
Muscicapidae	*Enicurus ruficapillus*				7	12	3	16	3	15	4	18	3	19	4	21	4	27	5	27	4
Muscicapidae	*Enicurus immaculatus*			x	7	29	4x	37	4x	27	4x	28	4x	29	4x	38	4x	29	4x	37	4x

Family	Species	IUCN	Ag	R	Range Source	Percentage loss in geographic range 2050 TG		AM		GO		OS		2100 TG		AM		GO		OS	
						H	C	H	C	H	C	H	C	H	C	H	C	H	C	H	C
Muscicapidae	*Enicurus schistaceus*				7	11	4	15	3	12	4	14	4	11	5	15	6	17	5	16	6
Muscicapidae	*Enicurus maculatus*				7	33	7	35	6	33	7	35	6	37	9	37	8	38	10	39	8
Muscicapidae	*Cochoa purpurea*				7	35	5	37	5	34	6	36	6	39	7	40	7	38	8	40	8
Muscicapidae	*Cochoa viridis*				7	27	10	30	9	27	12	29	11	31	13	33	13	32	16	35	14
Muscicapidae	*Cochoa beccarii*	T			7	25	7	25	7	23	5	28	7	31	7	34	8	47	5	40	7
Muscicapidae	*Cochoa azurea*	T			7	0	0	0	0	0	0	0	0	2	0x	0	0	0	0	0	0
Muscicapidae	*Saxicola rubetra*				3,4,5,7	6	20	5	17	9	21	7	16	11	23	9	21	15	24	13	18
Muscicapidae	*Saxicola macrorhyncha*	T			7	8	4	9	5	6	5	10	4	8	9	9	14	6	19	12	6
Muscicapidae	*Saxicola insignis*	T			7	0	5	0	7	0	7	0	5	0	8	0	10	0	9	0	9
Muscicapidae	*Saxicola dacotiae*	T	H	x	13	0	44x	0	44x	0	44x	0	44x	0	44x	0	44x	0	44x	0	44x
Muscicapidae	*Saxicola torquata*				3,4,5,6,7	14	10	11	9	14	10	15	8	16	12	13	14	17	14	18	12
Muscicapidae	*Saxicola axillaris*			x	6,13	0	0x	0	0x	0	0x	0	0x	0	0x	0	0x	0	0x	0	0x
Muscicapidae	*Saxicola tectes*			x	13	51	0x	51	0x	51	0x	51	0x	51	0x	51	0x	51	0x	51	0x
Muscicapidae	*Saxicola leucura*				7	19	4	21	4	19	5	21	4	22	6	24	7	22	10	25	4
Muscicapidae	*Saxicola caprata*				4,7,8	11	5	12	5	11	5	14	5	13	7	14	9	14	11	19	7
Muscicapidae	*Saxicola aethiops*				8	5	1	5	1	29	1	20	0	11	7	13	10	45	9	34	4
Muscicapidae	*Saxicola jerdoni*				7	32	8	34	7	29	10	33	8	35	9	37	9	34	11	40	9
Muscicapidae	*Saxicola ferrea*		H		7	0	5	0	4	0	5	0	4	0	6	0	6	0	7	0	6
Muscicapidae	*Saxicola gutturalis*			x	7	0	21x	2	21x	13	10x	0	21x	0	21x	2	21x	13	10x	0	21x
Muscicapidae	*Oenanthe bifasciata*				14	0	0	0	0	0	0	0	0	0	1	0	1	0	1	0	1
Muscicapidae	*Oenanthe leucopyga*				3,4,5	1	2	1	1	1	2	1	1	1	2	1	2	1	2	1	2
Muscicapidae	*Oenanthe monacha*				3,4,5	2	3	1	1	3	1	6	2	8	3	4	2	9	1	9	2
Muscicapidae	*Oenanthe alboniger*				4,7	7	3	7	3	10	2	12	2	16	5	11	4	17	3	19	3
Muscicapidae	*Oenanthe leucura*				3,4,5	9	6	9	4	8	6	9	4	9	8	9	7	9	11	10	5
Muscicapidae	*Oenanthe monticola*				5	4	5	2	5	4	5	3	5	4	7	2	8	4	8	4	8
Muscicapidae	*Oenanthe phillipsi*				5	30	4	7	19	37	1	37	1	34	4	10	24	37	5	37	4
Muscicapidae	*Oenanthe oenanthe*		H		2,3,4,5,7	0	16	0	15	0	17	0	14	0	19	0	21	0	24	0	21
Muscicapidae	*Oenanthe lugens*				3,4,5	6	5	5	6	8	6	9	5	10	6	7	10	10	11	11	10
Muscicapidae	*Oenanthe lugentoides*				4	0	0	0	0	0	0	0	0	0	0	0	1	0	2	0	2
Muscicapidae	*Oenanthe finschii*		H		3,4,5	0	2	0	2	0	2	0	2	0	2	0	2	0	3	0	2
Muscicapidae	*Oenanthe picata*		H		4,7	0	6	0	6	0	6	0	5	0	9	0	9	0	10	0	7
Muscicapidae	*Oenanthe moesta*				3,4	15	3	15	2	15	3	15	2	15	3	15	3	15	4	15	3
Muscicapidae	*Oenanthe hispanica*		H		3,4,5	0	8	0	7	0	8	0	6	0	11	0	10	0	12	0	7
Muscicapidae	*Oenanthe pleschanka*		H		3,4,5,7	0	10	0	10	0	12	0	10	0	13	0	15	0	17	0	15
Muscicapidae	*Oenanthe cypriaca*		H	x	4	0	0x	0	0x	0	0x	0	0x	0	0x	0	0x	0	0x	0	0x
Muscicapidae	*Oenanthe xanthoprymna*				3,4,5	9	7	10	7	13	7	15	6	18	8	15	8	20	8	28	7
Muscicapidae	*Oenanthe deserti*				3,4,5,7	7	10	7	11	7	12	8	11	8	15	9	20	11	22	13	20
Muscicapidae	*Oenanthe pileata*				5	19	4	14	6	19	4	20	4	20	6	15	11	20	7	20	7
Muscicapidae	*Oenanthe isabellina*				3,4,5,7	6	9	6	10	6	11	7	10	8	12	7	15	9	17	11	15
Muscicapidae	*Oenanthe bottae*				4,5	15	1	8	3	17	1	18	0	18	1	10	5	19	2	19	2
Muscicapidae	*Cercomela sinuata*		H		5	0	3	0	3	0	4	0	3	0	4	0	5	0	6	0	6
Muscicapidae	*Cercomela schlegelii*				5	4	5	2	6	4	7	3	6	4	7	2	8	4	9	3	9
Muscicapidae	*Cercomela tractrac*				5	4	8	2	10	4	11	4	10	4	11	2	17	4	17	4	17
Muscicapidae	*Cercomela familiaris*				4,5	29	2	21	3	30	2	33	2	33	3	23	8	34	5	34	4

Family	Species	IUCN	Ag	R	Range Source	Percentage loss in geographic range 2050 TG H	TG C	AM H	AM C	GO H	GO C	OS H	OS C	2100 TG H	TG C	AM H	AM C	GO H	GO C	OS H	OS C
Muscicapidae	*Cercomela scotocerca*				5	30	4	9	18	35	2	35	2	32	4	12	21	35	2	35	2
Muscicapidae	*Cercomela fusca*				7	7	2	7	2	6	2	8	1	8	3	8	7	6	9	10	2
Muscicapidae	*Cercomela dubia*				5	57	0x	20	31x	64	0x	64	3x	64	0x	26	38x	64	3x	64	3x
Muscicapidae	*Cercomela melanura*				3,4,5	5	5	2	5	6	5	7	5	6	5	2	8	9	9	8	9
Muscicapidae	*Cercomela sordida*				5	17	2	10	5	21	1	21	1	20	2	13	6	22	1	21	1
Muscicapidae	*Myrmecocichla tholloni*				5	45	6	25	10	37	7	52	5	50	6	31	10	49	7	54	5
Muscicapidae	*Myrmecocichla aethiops*				5	7	4	4	4	8	4	13	3	11	4	6	6	12	6	14	4
Muscicapidae	*Myrmecocichla formicivora*				5	6	6	2	7	5	6	6	6	6	8	2	13	5	11	6	10
Muscicapidae	*Myrmecocichla nigra*				5	35	3	24	7	35	4	42	2	40	4	28	9	40	4	42	2
Muscicapidae	*Myrmecocichla melaena*				5	8	0	6	0	10	0	10	0	10	0	8	0	10	0	10	0
Muscicapidae	*Myrmecocichla albifrons*		H		5	0	3	0	5	0	4	0	3	0	4	0	9	0	6	0	5
Muscicapidae	*Myrmecocichla arnotti*				5	44	1	33	2	43	1	47	1	46	2	35	7	46	2	47	1
Muscicapidae	*Thamnolaea cinnamomeiv*				5	33	2	26	5	34	2	36	1	35	3	29	8	37	3	37	2
Muscicapidae	*Thamnolaea semirufa*				5	35	0	19	3	36	0	36	0	36	0	25	5	36	0	36	0
Muscicapidae	*Pinarornis plumosus*				5	19	2	18	2	19	2	19	2	19	4	18	9	19	3	19	3
Sturnidae	*Aplonis zelandica*			x	9	4	0x	14	0x	29	0x	9	0x	21	0x	29	0x	42	0x	39	0x
Sturnidae	*Aplonis santovestris*	T		x	9	0	0x	100	0x	0	0x	0	0x	0	0x	100	0x	0	0x	100	0x
Sturnidae	*Aplonis atrifusca*			x	11	0	0x	0	0x	1	0x	0	0x	44	0x	0	0x	1	0x	1	0x
Sturnidae	*Aplonis tabuensis*				9,11	17	6x	17	6x	26	1x	36	1x	28	6x	33	6x	64	1x	62	1x
Sturnidae	*Aplonis striata*		H	x	9	0	34x	0	18x	0	22x	0	14x	0	34x	0	19x	0	23x	0	14x
Sturnidae	*Aplonis crassa*			x	7	7	14x	7	14x	20	1x	67	20x	7	14x	7	14x	28	1x	67	20x
Sturnidae	*Aplonis cantoroides*				8,9	5	2	7	2	21	1	18	1	14	4	16	4	32	2	28	2
Sturnidae	*Aplonis insularis*			x	9	0	0x	0	0x	92	0x	92	0x	92	0x	0	0x	92	0x	92	0x
Sturnidae	*Aplonis grandis*				9	13	6	12	1	22	1	26	1	29	10	23	1	53	2	41	1
Sturnidae	*Aplonis dichroa*			x	9	48	0x	34	0x	40	0x	77	0x	48	0x	34	0x	40	0x	99	0x
Sturnidae	*Aplonis mysolensis*				7,8	13	8	18	7	16	5	21	7	24	8	22	8	25	6	30	8
Sturnidae	*Aplonis minor*				7	12	7	21	8	17	7	11	9	14	10	24	15	29	17	18	16
Sturnidae	*Aplonis panayensis*		H		7	0	5	0	5	0	5	0	5	0	7	0	6	0	7	0	6
Sturnidae	*Aplonis metallica*				7,8,9,10	6	5	8	4	17	4	18	2	14	7	14	6	28	5	27	4
Sturnidae	*Aplonis magna*			x	8	0	7x	0	7x	0	7x	0	7x	0	7x	0	7x	0	7x	0	7x
Sturnidae	*Aplonis mystacea*				8	4	0	4	0	5	0	9	0	8	1	13	0	11	0	11	0
Sturnidae	*Aplonis brunneicapilla*	T		x	9	14	2x	12	0x	10	0x	24	0x	26	2x	18	0x	51	0x	48	0x
Sturnidae	*Poeoptera stuhlmanni*				5	33	5	19	10	33	6	39	1	35	5	24	10	37	7	39	1
Sturnidae	*Poeoptera kenricki*			x	5	8	0x	3	0x	8	0x	8	0x	8	0x	8	0x	8	0x	8	0x
Sturnidae	*Poeoptera lugubris*				5	30	6	20	7	33	7	46	5	47	8	28	9	48	9	52	6
Sturnidae	*Onychognathus neumanni*				14	26	4	13	5	29	5	48	6	37	4	25	7	56	9	52	6
Sturnidae	*Grafisia torquata*			x	5	18	0x	2	15x	36	15x	23	0x	51	0x	3	48x	39	15x	24	33x
Sturnidae	*Onychognathus walleri*				5	20	7	16	9	32	3	30	2	25	10	20	11	36	4	32	3
Sturnidae	*Onychognathus nabouroup*				5	4	9	1	10	3	11	3	10	4	12	1	18	4	17	3	17
Sturnidae	*Onychognathus tristramii*				3,4	0	1	0	0	0	0	0	0	1	1	1	1	1	2	1	2
Sturnidae	*Onychognathus morio*				5	23	4	18	6	23	4	23	4	23	6	20	10	24	5	24	5
Sturnidae	*Onychognathus blythii*				4,5	12	0	12	0	12	0	12	0	12	0	12	0	12	0	12	0
Sturnidae	*Onychognathus frater*			x	4,5	0	0x	0	0x	0	0x	0	0x	0	0x	0	0x	0	0x	0	0x
Sturnidae	*Onychognathus fulgidus*				5	34	5	21	8	31	6	53	4	52	6	31	9	58	7	62	5

Family	Species	IUCN	Ag	R	Range Source	Percentage loss in geographic range 2050 TG H	TG C	AM H	AM C	GO H	GO C	OS H	OS C	2100 TG H	TG C	AM H	AM C	GO H	GO C	OS H	OS C
Sturnidae	*Onychognathus tenuirostris*				5	28	3	25	5	29	4	33	0	30	4	26	5	32	6	33	0
Sturnidae	*Onychognathus albirostris*				5	13	0	13	0	17	0	17	0	17	0	13	0	17	0	17	0
Sturnidae	*Onychognathus salvadorii*				5	60	10	17	33	74	2	74	2	67	10	21	46	74	2	74	2
Sturnidae	*Coccycolius iris*			x	5	2	4x	0	4x	1	4x	5	4x	3	4x	1	6x	3	4x	5	4x
Sturnidae	*Lamprotornis cupreocauda*				5	32	5	17	1	17	6	44	1	36	7	27	8	39	8	46	1
Sturnidae	*Lamprotornis purpureiceps*				5	35	5	19	6	31	6	53	3	52	6	28	9	58	8	63	4
Sturnidae	*Lamprotornis corruscus*				5	36	0	28	0	36	0	36	0	36	0	32	0	36	0	36	0
Sturnidae	*Lamprotornis purpureus*				5	14	3	9	5	15	2	18	1	17	3	11	6	20	3	19	2
Sturnidae	*Lamprotornis nitens*				5	18	2	13	3	17	2	18	2	18	4	13	9	17	6	18	5
Sturnidae	*Lamprotornis chalcurus*				5	11	2	9	1	17	1	20	1	17	2	11	3	21	1	21	1
Sturnidae	*Lamprotornis chalybaeus*				5	18	3	11	6	19	3	22	2	21	4	13	10	22	6	23	5
Sturnidae	*Lamprotornis chloropterus*				5	26	3	17	5	29	3	36	2	33	4	21	9	37	5	37	4
Sturnidae	*Lamprotornis acuticaudus*				5	46	0	30	1	45	0	49	0	49	0	31	6	48	0	49	0
Sturnidae	*Lamprotornis splendidus*				5	37	6	26	7	37	7	55	4	51	7	34	10	57	9	62	5
Sturnidae	*Lamprotornis ornatus*			x	5	0	56x	0	56x	56	0x	56	0x	0	56x	0	56x	56	0x	56	0x
Sturnidae	*Lamprotornis australis*				5	8	5	2	8	7	6	8	5	8	9	2	17	7	12	8	10
Sturnidae	*Lamprotornis mevesii*				5	28	2	24	2	28	2	28	2	28	5	25	10	28	5	28	4
Sturnidae	*Lamprotornis caudatus*				5	8	3	4	3	8	3	12	2	11	3	6	5	13	5	13	3
Sturnidae	*Lamprotornis purpuropteru*				5	36	2	20	9	39	1	40	1	39	2	24	12	40	2	40	2
Sturnidae	*Lamprotornis superbus*				5	39	4	15	21	44	1	44	1	42	4	18	24	45	2	45	2
Sturnidae	*Lamprotornis pulcher*				5	7	3	3	3	7	3	10	2	10	3	5	6	11	6	11	4
Sturnidae	*Lamprotornis shelleyi*				5	61	1	27	12	66	0	66	0	64	1	36	17	66	0	66	0
Sturnidae	*Lamprotornis hildebrandti*				5	34	4	30	6	37	3	37	3	35	5	35	6	41	3	40	3
Sturnidae	*Cinnyricinclus sharpii*				5	31	1	26	6	33	0	33	0	32	1	27	6	33	0	33	0
Sturnidae	*Cinnyricinclus femoralis*	T		x	5	20	10x	20	10x	30	0x	30	0x	20	10x	20	10x	30	0x	30	0x
Sturnidae	*Cinnyricinclus leucogaster*				4,5	33	3	20	4	35	3	41	2	39	4	24	9	41	5	42	4
Sturnidae	*Speculipastor bicolor*			x	5	11	3x	9	5x	12	3x	12	3x	12	3x	9	6x	12	3x	12	3x
Sturnidae	*Neocichla gutturalis*				5	44	0	37	2	44	0	44	0	44	0	37	9	44	0	44	0
Sturnidae	*Spreo fischeri*				5	36	5	10	28	41	0	41	0	37	5	12	30	42	1	42	0
Sturnidae	*Spreo bicolor*				5	2	4	2	5	2	5	2	4	2	5	2	5	3	6	3	6
Sturnidae	*Spreo albicapillus*				5	42	4	12	22	50	1	50	0	47	4	17	28	50	4	50	2
Sturnidae	*Cosmopsarus regius*				5	32	5	10	21	38	1	39	1	34	5	12	25	38	4	39	3
Sturnidae	*Cosmopsarus unicolor*				5	38	4	33	5	38	4	38	4	38	4	37	5	40	4	38	4
Sturnidae	*Saroglossa aurata*				6	59	0	32	8	59	0	59	0	59	0	44	8	59	0	59	0
Sturnidae	*Saroglossa spiloptera*		H		7	0	3	0	2	0	0	0	1	0	4	0	3	0	0	0	1
Sturnidae	*Creatophora cinerea*				4,5	26	3	16	9	27	3	29	3	28	5	17	14	29	6	29	5
Sturnidae	*Sturnus senex*	T	H		7	0	0	0	0	0	0	0	2	0	0	0	0	0	0	0	2
Sturnidae	*Sturnus malabaricus*		H		7	0	5	0	4	0	5	0	4	0	7	0	8	0	10	0	6
Sturnidae	*Sturnus erythropygius*		H	x	7	0	2x	0	0x	0	0x	0	0x	0	2x	0	0x	0	0x	0	0x
Sturnidae	*Sturnus pagodarum*		H		7	0	3	0	3	0	2	0	2	0	5	0	7	0	8	0	2
Sturnidae	*Sturnus sericeus*		H		7	0	0	0	0	0	0	0	0	0	0	0	0	0	0	0	0
Sturnidae	*Sturnus sturninus*				4,7	11	4	10	4	10	4	10	4	11	5	11	6	11	5	11	6
Sturnidae	*Sturnus philippensis*		H		7	0	20	0	21	0	22	0	22	0	32	0	42	0	35	0	38
Sturnidae	*Sturnus sinensis*				7	7	6	9	3	8	5	10	5	7	6	11	4	12	5	13	6

Family	Species	IUCN	Ag	R	Range Source	Percentage loss in geographic range 2050								2100							
						TG		AM		GO		OS		TG		AM		GO		OS	
						H	C	H	C	H	C	H	C	H	C	H	C	H	C	H	C
Sturnidae	*Sturnus roseus*				3,4,5,7	3	9	3	10	3	12	4	10	5	11	4	15	5	17	10	16
Sturnidae	*Sturnus vulgaris*		H		,4,5,7,8,10,11,12	0	18	0	15	0	18	0	14	0	22	0	20	0	24	0	18
Sturnidae	*Sturnus unicolor*		H		3,4,5	0	14	0	8	0	13	0	7	0	21	0	19	0	25	0	9
Sturnidae	*Sturnus cineraceus*		H		7	0	4	0	4	0	4	0	4	0	5	0	6	0	6	0	6
Sturnidae	*Sturnus contra*		H		7	0	4	0	4	0	4	0	3	0	5	0	7	0	8	0	4
Sturnidae	*Sturnus nigricollis*		H		7	0	6	0	6	0	7	0	7	0	11	0	10	0	13	0	10
Sturnidae	*Sturnus burmannicus*				7	13	10	17	10	13	12	12	11	15	15	19	17	17	20	17	17
Sturnidae	*Sturnus melanopterus*	T			7	2	0	0	0	0	0	0	0	5	0	1	2	2	0	1	0
Sturnidae	*Leucopsar rothschildi*	T		x	7	0	0x	0	0x	0	0x	0	0x	0	0x	0	2x	38	0x	0	0x
Sturnidae	*Acridotheres tristis*		H		,5,6,7,9,10,11,12	0	4	0	4	0	4	0	3	0	7	0	7	0	9	0	5
Sturnidae	*Acridotheres ginginianus*		H		4,7	0	2	0	2	0	2	0	2	0	5	0	9	0	11	0	3
Sturnidae	*Acridotheres fuscus*		H		7,11	0	1	0	2	0	2	0	1	0	2	0	3	0	4	0	2
Sturnidae	*Acridotheres grandis*		H		7	0	8	0	7	0	9	0	8	0	13	0	12	0	16	0	12
Sturnidae	*Acridotheres cinereus*		H	x	7	0	0x	0	0x	0	0x	0	0x	0	0x	0	0x	0	0x	0	0x
Sturnidae	*Acridotheres albocinctus*		H		7	0	17	0	15	0	27	0	20	0	25	0	25	0	28	0	22
Sturnidae	*Acridotheres cristatellus*		H		2,7	0	4	0	2	0	3	0	3	0	4	0	3	0	3	0	4
Sturnidae	*Ampeliceps coronatus*				7	23	11	25	10	21	13	23	12	25	14	27	16	26	20	30	17
Sturnidae	*Mino anais*				8	4	2	6	2	15	2	14	1	11	3	13	4	25	3	23	1
Sturnidae	*Mino dumontii*				7,8,9	5	3	6	3	18	2	17	1	12	5	13	5	30	4	26	2
Sturnidae	*Basilornis celebensis*				7	15	14	26	10	17	14	17	13	16	15	29	21	27	28	24	26
Sturnidae	*Basilornis galeatus*			x	7	64	24x	71	24x	0	84x	4	84x	64	24x	71	24x	0	84x	4	84x
Sturnidae	*Basilornis corythaix*			x	7	16	0x	19	0x	21	0x	21	0x	34	0x	19	0x	25	0x	44	0x
Sturnidae	*Basilornis miranda*			x	7	27	2x	27	2x	11	2x	27	2x	27	2x	27	2x	32	2x	27	2x
Sturnidae	*Streptocitta albicollis*				7	15	14	24	10	18	13	17	13	17	15	28	22	27	28	24	26
Sturnidae	*Streptocitta albertinae*			x	7	63	0x	20	42x	24	37x	19	37x	63	0x	41	42x	24	42x	19	42x
Sturnidae	*Sarcops calvus*				7	15	11	24	11	13	12	22	12	21	18	31	14	25	22	34	13
Sturnidae	*Gracula ptilogenys*			x	7	33	0x	33	0x	34	0x	33	0x	34	0x	33	0x	34	0x	33	0x
Sturnidae	*Gracula religiosa*				7	16	7	19	6	17	8	20	7	20	9	24	9	26	11	28	9
Sturnidae	*Enodes erythrophris*				7	16	14	28	10	20	14	16	13	18	16	32	23	30	32	25	29
Sturnidae	*Scissirostrum dubium*				7	15	15	24	10	17	14	17	13	16	15	27	22	27	29	23	26
Sturnidae	*Buphagus africanus*				5	11	4	7	4	12	3	16	2	14	4	9	6	17	6	17	4
Sturnidae	*Buphagus erythrorhynchus*				5	33	4	15	14	37	2	37	2	35	5	18	19	37	4	38	4
Sturnidae	*Dumetella carolinensis*		H		2,13	0	19	0	11	0	15	0	9	0	22	0	15	0	20	0	11
Sturnidae	*Melanoptila glabrirostris*			x	2	0	7x	9	0x	3	7x	13	0x	0	13x	14	17x	10	7x	14	7x
Sturnidae	*Melanotis caerulescens*				2	11	10	23	6	12	9	28	5	15	11	24	16	17	15	32	8
Sturnidae	*Melanotis hypoleucus*				2	12	0	22	0	20	2	28	0	13	0	24	1	22	2	29	0
Sturnidae	*Mimus polyglottos*		H		2,11	0	18	0	12	0	15	0	10	0	21	0	19	0	23	0	14
Sturnidae	*Mimus gilvus*				2	13	8	12	8	13	7	18	6	16	10	17	13	26	13	26	10
Sturnidae	*Mimus gundlachii*			x	2	20	7x	56	5x	52	9x	45	1x	34	7x	56	7x	60	9x	76	1x
Sturnidae	*Mimus saturninus*				2	7	12	5	8	7	10	10	6	9	17	8	11	12	13	16	9
Sturnidae	*Mimus patagonicus*				2	1	7	1	8	1	8	1	7	2	11	1	15	2	18	3	16
Sturnidae	*Mimus dorsalis*				2	18	16	19	22	10	18	24	10	26	19	21	26	24	24	37	10
Sturnidae	*Mimus triurus*				2	1	10	0	11	1	11	1	10	1	13	0	17	1	20	2	17
Sturnidae	*Mimus longicaudatus*		H		2	0	24	0	22	0	29	0	21	0	32	0	39	0	41	0	35

Family	Species	IUCN	Ag	R	Range Source	2050 TG H	2050 TG C	2050 AM H	2050 AM C	2050 GO H	2050 GO C	2050 OS H	2050 OS C	2100 TG H	2100 TG C	2100 AM H	2100 AM C	2100 GO H	2100 GO C	2100 OS H	2100 OS C
Sturnidae	*Mimus thenca*				2	0	0	4	0	6	0	1	0	9	2	12	0	14	2	10	1
Sturnidae	*Nesomimus parvulus*			x	2	0	5x	0	5x	0	0x	0	5x	0	5x	0	26x	0	35x	0	26x
Sturnidae	*Nesomimus trifasciatus*	T		x	2	0	0x	0	0x	0	0x	0	0x	0	0x	0	0x	0	102x	0	0x
Sturnidae	*Nesomimus macdonaldi*	T		x	2	0	102x	0	102x	0	102x	0	102x	0	102x	0	102x	0	102x	0	102x
Sturnidae	*Nesomimus melanotis*			x	2	0	102x	0	102x	0	102x	0	102x	0	102x	0	102x	0	102x	0	102x
Sturnidae	*Mimodes graysoni*	T		x	2	0	0x	0	0x	0	0x	0	0x	0	0x	0	0x	0	0x	0	0x
Sturnidae	*Oreoscoptes montanus*				2	0	12	1	11	1	12	1	10	0	14	1	13	2	15	2	12
Sturnidae	*Toxostoma rufum*				2	9	20	15	11	14	16	14	7	15	23	20	14	25	19	20	8
Sturnidae	*Toxostoma longirostre*				2	0	21	0	10	0	13	1	4	0	32	1	50	0	35	1	7
Sturnidae	*Toxostoma guttatum*	T		x	2	0	0x	0	0x	0	0x	0	0x	0	0x	0	0x	0	0x	0	0x
Sturnidae	*Toxostoma bendirei*	T			2	1	18	2	20	0	22	4	20	2	23	2	37	0	43	4	38
Sturnidae	*Toxostoma cinereum*				2	0	2	2	0	0	2	2	1	0	2	2	0	0	2	2	1
Sturnidae	*Toxostoma curvirostre*				2	5	17	10	13	5	15	14	12	6	20	11	29	7	31	15	23
Sturnidae	*Toxostoma ocellatum*				2	5	0	5	0	6	0	7	0	5	0	6	5x	6	0	7	0
Sturnidae	*Toxostoma lecontei*				2	0	31	1	37	0	39	1	37	0	40	1	54	0	63	1	57
Sturnidae	*Toxostoma redivivum*				2	0	52	5	36	2	51	6	24	1	54	5	41	4	56	9	29
Sturnidae	*Toxostoma crissale*				2	1	27	4	26	2	28	5	27	1	30	4	42	3	47	6	45
Sturnidae	*Cinclocerthia ruficauda*			x	2	6	13x	39	9x	20	4x	35	0x	6	13x	52	9x	31	4x	66	0x
Sturnidae	*Cinclocerthia gutturalis*			x	2	0	36x	28	26x	26	11x	36	0x	0	36x	63	26x	26	11x	88	0x
Sturnidae	*Ramphocinclus brachyurus*	T		x	2	0	36x	28	26x	26	11x	36	0x	0	36x	63	26x	26	11x	88	0x
Sturnidae	*Margarops fuscus*			x	2	5	17x	40	10x	22	7x	41	0x	7	17x	53	10x	34	7x	67	0x
Sturnidae	*Margarops fuscatus*			x	2	23	9x	44	8x	33	8x	51	0x	27	9x	48	10x	41	8x	62	0x
Sittidae	*Sitta europaea*				3,4,5,7	5	14	5	13	6	15	6	12	7	17	6	17	9	19	9	16
Sittidae	*Sitta nagaensis*				7	33	4	34	4	31	5	30	5	35	4	36	5	34	6	34	6
Sittidae	*Sitta cashmirensis*				7	69	4	69	1	76	0	73	2	70	5	76	1	80	0	79	2
Sittidae	*Sitta castanea*				7	29	8	31	7	29	8	32	8	33	10	35	10	34	12	43	10
Sittidae	*Sitta himalayensis*				7	64	5	62	3	63	2	69	1	68	5	70	3	72	3	74	1
Sittidae	*Sitta victoriae*	T		x	7	1	8x	1	8x	1	8x	9	0x	1	8x	1	8x	37	8x	10	0x
Sittidae	*Sitta pygmaea*				2	6	18	15	13	9	17	19	12	8	23	17	24	15	30	26	19
Sittidae	*Sitta pusilla*				2	13	26	21	9	20	18	16	7	25	33	29	16	43	26	23	8
Sittidae	*Sitta whiteheadi*			x	3	0	15x	0	15x	0	15x	0	15x	0	48x	5	48x	5	48x	0	48x
Sittidae	*Sitta ledanti*	T		x	3,5	0	0x	0	0x	0	0x	0	0x	0	0x	0	0x	0	0x	0	0x
Sittidae	*Sitta krueperi*				3,4	24	1	24	1	23	2	29	1	33	1	31	1	33	2	34	1
Sittidae	*Sitta villosa*				7	4	1	3	3	4	2	4	1	5	1	3	3	4	2	5	1
Sittidae	*Sitta yunnanensis*				7	25	0	24	0	24	0	24	0	25	0	24	0	25	0	24	0
Sittidae	*Sitta canadensis*				2	3	19	5	17	5	18	6	16	4	25	8	26	9	28	11	23
Sittidae	*Sitta leucopsis*				7	55	3	58	1	60	2	59	3	57	5	65	4	63	4	62	4
Sittidae	*Sitta carolinensis*				2	7	20	12	13	10	17	12	11	11	24	17	20	19	24	18	15
Sittidae	*Sitta neumayer*				3,4	17	7	17	7	19	8	27	6	29	8	24	9	29	10	31	8
Sittidae	*Sitta tephronota*				3,4,7	7	7	7	7	9	7	14	6	15	8	11	8	16	8	23	7
Sittidae	*Sitta frontalis*				7	19	6	22	5	20	6	23	6	23	7	26	8	28	9	33	7
Sittidae	*Sitta solangiae*			x	7	11	0x	2	0x	1	0x	8	0x	11	0x	2	0x	15	0x	16	0x
Sittidae	*Sitta oenochlamys*				7	13	12	20	12	11	13	16	13	17	17	24	15	22	19	26	14
Sittidae	*Sitta azurea*				7	18	6	18	6	19	5	29	6	22	6	25	7	39	5	35	6

Family	Species	IUCN	Ag	R	Range Source	Percentage loss in geographic range 2050 TG H	C	AM H	C	GO H	C	OS H	C	2100 TG H	C	AM H	C	GO H	C	OS H	C
Sittidae	*Sitta magna*	T			7	23	5	26	6	21	6	19	7	26	5	28	6	23	7	23	8
Sittidae	*Sitta formosa*	T			7	57	2	61	2	58	5	61	2	67	2	63	3	65	6	69	3
Sittidae	*Tichodroma muraria*				3,4,7	10	18	10	17	11	17	11	17	13	24	14	27	18	28	20	25
Certhiidae	*Certhia familiaris*				3,4,7	7	14	7	13	8	15	7	13	9	17	8	17	11	18	12	16
Certhiidae	*Certhia americana*				2	8	20	14	16	12	19	15	14	11	25	18	25	19	29	22	22
Certhiidae	*Certhia brachydactyla*				3,4,5	7	20	7	14	10	18	9	11	12	26	11	21	19	25	13	14
Certhiidae	*Certhia himalayana*				7	34	2	34	1	35	1	36	1	35	3	37	4	38	4	37	3
Certhiidae	*Certhia nipalensis*				7	63	5	66	4	62	3	70	1	66	7	70	5	70	5	74	3
Certhiidae	*Certhia discolor*				7	48	8	50	7	44	8	48	8	52	9	55	9	50	10	53	10
Certhiidae	*Salpornis spilonotus*				5,7	31	1	26	1	32	1	36	1	34	2	28	5	35	2	39	1
Certhiidae	*Donacobius atricapillus*				2	10	13	7	10	9	11	14	8	14	18	12	15	20	16	22	12
Certhiidae	*Campylorhynchus gularis*				2	19	16	35	10	22	15	45	7	27	19	35	22	28	23	49	12
Certhiidae	*Campylorhynchus brunneic*				2	1	22	4	19	2	21	5	19	2	26	4	36	3	39	5	32
Certhiidae	*Campylorhynchus jocosus*				2	9	4	21	0	11	4	17	0	9	4	21	5	11	4	17	0
Certhiidae	*Campylorhynchus yucatani*			x	2	0	44x	10	0x	10	44x	10	0x	0	65x	10	83x	10	44x	10	44x
Certhiidae	*Campylorhynchus chiapen*			x	2	0	0x	0	0x	0	0x	0	0x	0	0x	0	1x	0	0x	0	0x
Certhiidae	*Campylorhynchus griseus*				2	16	4	12	5	13	4	17	3	18	6	17	8	25	9	26	6
Certhiidae	*Campylorhynchus rufinuch*				2	11	0	19	0	17	0	28	0	14	0	20	12	22	3	30	0
Certhiidae	*Campylorhynchus turdinus*				2	7	10	5	11	6	11	11	10	12	12	10	14	18	14	22	13
Certhiidae	*Campylorhynchus megalop*				2	3	10	20	1	8	10	21	0	4	10	20	1	10	10	23	0
Certhiidae	*Campylorhynchus zonatus*		H		2	0	3	0	4	0	5	0	1	0	3	0	9	0	7	0	2
Certhiidae	*Campylorhynchus albobrun*				2	9	6	17	9	9	6	13	8	19	9	18	9	20	13	28	11
Certhiidae	*Campylorhynchus nuchalis*		H		2	0	4	0	6	0	5	0	3	0	5	0	9	0	11	0	3
Certhiidae	*Campylorhynchus fasciatus*				2	5	8	0	4	0	10	1	4	5	10	0	9	2	14	2	9
Certhiidae	*Odontorchilus branickii*				2	10	10	10	7	16	10	20	8	19	10	20	12	32	11	31	10
Certhiidae	*Odontorchilus cinereus*				2	5	17	4	19	4	20	9	18	10	20	8	20	19	20	18	19
Certhiidae	*Salpinctes obsoletus*				2	1	17	2	15	1	17	3	14	1	20	3	24	2	27	4	21
Certhiidae	*Catherpes mexicanus*				2	1	17	4	15	2	17	5	15	2	20	4	26	3	29	6	24
Certhiidae	*Hylorchilus sumichrasti*			x	2	0	0x	0	0x	0	0x	0	0x	0	0x	0	0x	0	0x	0	0x
Certhiidae	*Hylorchilus navai*	T		x	2	0	0x	0	0x	0	0x	0	0x	0	0x	0	82x	0	0x	0	0x
Certhiidae	*Cinnycerthia unirufa*				2	24	4	28	3	18	5	34	3	32	7	47	6	51	10	38	9
Certhiidae	*Cinnycerthia olivascens*				2	11	8	10	6	9	9	16	6	14	9	21	12	24	9	21	10
Certhiidae	*Cinnycerthia peruana*				2	7	0	5	0	6	0	10	5	12	0	5	8	16	3	11	5
Certhiidae	*Cinnycerthia fulva*				2	11	14	10	5	15	9	20	11	15	14	17	15	26	12	26	15
Certhiidae	*Cistothorus platensis*				2	5	9	4	5	6	7	6	4	6	12	7	9	10	11	9	7
Certhiidae	*Cistothorus apolinari*	T		x	2	0	0x	0	0x	0	0x	0	0x	0	0x	0	0x	0	0x	0	0x
Certhiidae	*Cistothorus meridae*			x	2	38	0x	16	0x	4	0x	57	0x	38	16x	16	38x	32	50x	57	0x
Certhiidae	*Cistothorus palustris*				2	2	12	4	8	4	11	3	6	3	14	4	10	6	14	4	7
Certhiidae	*Thryomanes bewickii*				2	2	17	3	14	2	16	4	13	2	21	4	26	3	28	5	22
Certhiidae	*Ferminia cerverai*	T		x	2	0	20x	0	20x	20	0x	73	0x	0	20x	0	20x	20	0x	73	0x
Certhiidae	*Thryothorus atrogularis*				2	37	0	44	8x	57	8x	81	0x	53	0x	56	17x	60	8x	82	0x
Certhiidae	*Thryothorus spadix*			x	2	9	1x	14	3x	1	3x	13	0x	20	7x	17	3x	1	9x	17	6x
Certhiidae	*Thryothorus fasciatoventris*				2	16	5	21	12	14	9	20	9	22	7	23	13	25	16	33	12
Certhiidae	*Thryothorus euophrys*			x	2	26	12x	37	7x	24	18x	43	17x	36	23x	62	21x	70	22x	50	29x

Family	Species	IUCN	Ag	R	Range Source	Percentage loss in geographic range 2050 TG H	C	AM H	C	GO H	C	OS H	C	2100 TG H	C	AM H	C	GO H	C	OS H	C
Certhiidae	*Thryothorus eisenmanni*			x	2	0	0x	0	0x	0	0x	0	0x	0	0x	0	0x	0	0x	0	0x
Certhiidae	*Thryothorus mystacalis*				2	19	2	17	4	17	3	21	2	24	4	23	8	26	12	27	5
Certhiidae	*Thryothorus genibarbis*				2	7	16	4	14	6	15	11	12	11	19	9	17	17	18	21	15
Certhiidae	*Thryothorus coraya*				2	5	7	4	7	5	7	10	7	10	8	9	9	17	10	20	9
Certhiidae	*Thryothorus felix*				2	12	12	27	8	13	12	36	7	17	14	27	19	20	20	41	11
Certhiidae	*Thryothorus maculipectus*				2	1	5	3	2	2	2	4	2	1	6	4	54x	3	3	4	2
Certhiidae	*Thryothorus rutilus*				2	23	5	22	9	17	10	28	8	33	10	29	18	29	24	39	15
Certhiidae	*Thryothorus sclateri*			x	2	0	4x	4	0x	0	4x	0	4x	0	4x	4	0x	0	4x	0	4x
Certhiidae	*Thryothorus colombianus*			x	14	0	0x	0	0x	0	0x	0	0x	0	0x	0	0x	0	0x	0	0x
Certhiidae	*Thryothorus paucimaculatu*				14	5	5	0	1	1	5	1	0	5	5	0	3	3	8	3	2
Certhiidae	*Thryothorus semibadius*			x	2	44	9x	34	30x	26	30x	37	30x	55	9x	34	46x	29	38x	57	30x
Certhiidae	*Thryothorus nigricapillus*				2	20	4	27	7	24	6	33	4	29	5	32	9	33	9	43	5
Certhiidae	*Thryothorus thoracicus*				2	34	1	48	4	46	5	64	1	47	1	59	8	54	5	69	1
Certhiidae	*Thryothorus leucopogon*				2	11	5	17	8	14	5	14	5	20	8	18	8	24	11	29	8
Certhiidae	*Thryothorus pleurostictus*				2	9	0	26	0	17	0	28	0	11	0	27	6	22	2	30	0
Certhiidae	*Thryothorus ludovicianus*				2	11	20	17	8	15	13	16	6	18	24	24	15	32	18	24	7
Certhiidae	*Thryothorus rufalbus*				2	19	6	17	7	18	7	23	5	23	8	24	11	32	14	33	8
Certhiidae	*Thryothorus nicefori*	T		x	14	0	0x	0	0x	0	0x	0	0x	0	0x	0	0x	0	0x	0	0x
Certhiidae	*Thryothorus sinaloa*				2	12	11	27	7	14	10	40	6	18	13	27	20	21	19	47	10
Certhiidae	*Thryothorus modestus*		H		2	0	1	0	2	0	3	0	1	0	1	0	8	0	7	0	2
Certhiidae	*Thryothorus leucotis*				2	6	11	4	9	4	10	9	8	9	14	8	12	14	14	17	10
Certhiidae	*Thryothorus superciliaris*		H		2	0	6	0	8	0	10	0	8	0	10	0	20	0	23	0	20
Certhiidae	*Thryothorus guarayanus*				2	13	27	7	31	8	29	12	30	17	30	12	43	22	38	21	41
Certhiidae	*Thryothorus longirostris*				2	10	19	8	18	13	17	17	12	13	24	12	22	22	20	26	16
Certhiidae	*Thryothorus griseus*				2	0	2	0	2	0	2	0	2	0	2	0	2	0	2	0	2
Certhiidae	*Troglodytes troglodytes*				2,3,4,7	11	17	12	15	13	17	13	13	14	21	15	20	19	22	18	17
Certhiidae	*Troglodytes aedon*		H		2	0	13	0	10	0	12	0	9	0	16	0	15	0	16	0	12
Certhiidae	*Troglodytes beani*			x	14	0	0x	0	0x	0	0x	0	0x	0	0x	0	0x	0	0x	0	0x
Certhiidae	*Troglodytes cobbi*	T		x	2	0	0x	0	0x	0	0x	0	0x	0	0x	0	0x	0	0x	0	0x
Certhiidae	*Troglodytes sissonii*			x	2	0	0x	0	0x	0	0x	0	0x	0	0x	0	0x	0	0x	0	0x
Certhiidae	*Troglodytes rufociliatus*				2	15	0	23	0	21	3	30	0	17	0	24	1	24	4	30	0
Certhiidae	*Troglodytes ochraceus*			x	2	19	11x	33	19x	14	19x	31	17x	26	11x	33	20x	40	20x	50	17x
Certhiidae	*Troglodytes monticola*			x	2	35	0x	54	0x	90	0x	35	0x	90	0x	54	0x	90	0x	90	0x
Certhiidae	*Troglodytes solstitialis*				2	11	7	14	5	11	7	21	7	17	9	20	10	25	11	26	9
Certhiidae	*Troglodytes rufulus*				2	8	1	3	7	10	3	3	7	11	11	12	21	18	16	8	30
Certhiidae	*Thryorchilus browni*			x	2	14	29x	39	43x	1	43x	1	43x	14	29x	39	43x	51	43x	46	43x
Certhiidae	*Uropsila leucogastra*				2	5	4	14	3	10	4	22	3	6	6	18	19	13	9	27	5
Certhiidae	*Henicorhina leucosticta*				2	6	7	7	8	6	8	10	8	10	8	12	10	14	11	18	10
Certhiidae	*Henicorhina leucophrys*				2	13	4	18	3	15	5	24	3	17	5	24	9	27	8	29	5
Certhiidae	*Henicorhina leucoptera*			x	2	0	46x	6	40x	1	46x	1	46x	0	46x	6	40x	1	46x	1	46x
Certhiidae	*Microcerculus philomela*				2	22	2	35	4	29	4	45	2	29	3	44	8	42	6	50	4
Certhiidae	*Microcerculus marginatus*				2	6	9	5	9	5	9	10	8	11	9	10	10	17	10	19	9
Certhiidae	*Microcerculus ustulatus*				2	4	15	5	16	2	17	2	18	8	18	6	22	4	24	5	27
Certhiidae	*Microcerculus bambla*				2	3	13	3	13	2	13	5	13	6	15	5	16	10	18	10	17

Family	Species	IUCN	Ag	R	Range Source	2050 TG H	2050 TG C	2050 AM H	2050 AM C	2050 GO H	2050 GO C	2050 OS H	2050 OS C	2100 TG H	2100 TG C	2100 AM H	2100 AM C	2100 GO H	2100 GO C	2100 OS H	2100 OS C
Certhiidae	*Cyphorhinus phaeocephalu*				2	19	4	27	6	25	6	33	4	28	5	32	8	36	8	44	5
Certhiidae	*Cyphorhinus thoracicus*				2	11	10	8	6	11	9	20	9	20	10	16	17	29	14	27	13
Certhiidae	*Cyphorhinus aradus*				2	5	8	3	8	4	8	7	7	9	9	8	9	13	9	15	8
Certhiidae	*Auriparus flaviceps*				2	1	21	4	19	2	20	5	19	1	25	4	36	2	37	5	31
Certhiidae	*Microbates collaris*				2	3	9	2	8	2	9	4	8	6	10	4	10	8	12	8	11
Certhiidae	*Microbates cinereiventris*				2	12	3	13	4	13	3	21	1	18	3	20	5	20	5	35	2
Certhiidae	*Ramphocaenus melanurus*				2	7	11	5	11	6	12	11	10	11	13	10	14	17	15	19	13
Certhiidae	*Polioptila caerulea*				2	7	18	13	12	10	15	15	10	11	21	17	21	19	24	20	15
Certhiidae	*Polioptila californica*				2	0	3	5	1	1	3	7	3	2	3	5	1	5	3	8	4
Certhiidae	*Polioptila melanura*				2	2	27	6	24	3	26	7	23	3	31	6	41	4	44	8	38
Certhiidae	*Polioptila lembeyei*			x	2	10	5x	23	6x	8	6x	22	3x	16	5x	25	10x	11	7x	27	3x
Certhiidae	*Polioptila nigriceps*				2	8	13	15	7	4	11	31	7	12	14	15	19	6	17	35	11
Certhiidae	*Polioptila albiloris*				2	9	2	27	0	20	2	30	0	11	3	27	15	25	3	31	1
Certhiidae	*Polioptila plumbea*				2	8	9	7	9	7	9	13	7	12	11	11	12	18	13	22	10
Certhiidae	*Polioptila maranonica*				2	0	0	1	0	1	0	1	0	0	0	1	0	5	1	5	0
Certhiidae	*Polioptila lactea*				2	17	9	15	9	23	6	28	8	25	20	27	19	48	11	50	16
Certhiidae	*Polioptila guianensis*				2	5	14	4	13	4	14	7	12	8	14	7	14	13	16	14	13
Certhiidae	*Polioptila schistaceigula*				2	16	3	13	5	14	1	12	4	24	5	14	5	19	7	22	6
Certhiidae	*Polioptila dumicola*				2	5	11	2	7	4	9	7	6	7	15	5	11	9	13	11	9
Paridae	*Remiz pendulinus*				3,4,7	5	16	3	13	6	18	5	11	9	18	7	15	11	20	11	12
Paridae	*Remiz coronatus*				4,7	2	5	1	6	1	7	1	6	3	8	1	9	2	9	3	9
Paridae	*Remiz consobrinus*				7	4	0	4	0	4	0	4	0	4	0	4	0	4	0	4	0
Paridae	*Anthoscopus punctifrons*				5	6	5	2	5	7	5	11	4	11	6	4	9	13	11	12	9
Paridae	*Anthoscopus parvulus*				5	35	2	12	5	40	2	47	1	45	2	19	6	49	3	49	2
Paridae	*Anthoscopus musculus*				5	48	4	16	25	53	1	53	1	51	4	21	32	55	2	55	2
Paridae	*Anthoscopus flavifrons*				5	33	6	22	9	37	7	59	4	51	6	31	10	61	8	69	4
Paridae	*Anthoscopus caroli*				5	44	1	36	2	43	1	44	1	45	2	37	6	45	2	44	1
Paridae	*Anthoscopus minutus*				5	6	7	2	8	5	7	5	7	6	10	2	13	5	11	5	11
Paridae	*Cephalopyrus flammiceps*				7	44	2	45	0	45	0	47	1	45	2	48	1	48	0	49	1
Paridae	*Pholidornis rushiae*				5	36	5	20	7	30	7	46	6	50	6	28	11	50	9	53	7
Paridae	*Parus palustris*				3,4,7	9	15	9	12	10	15	10	11	12	18	11	15	16	18	14	14
Paridae	*Parus lugubris*		H		3,4	0	11	0	9	0	11	0	7	0	15	0	13	0	15	0	10
Paridae	*Parus montanus*				3,7	5	14	5	14	6	15	5	13	7	17	6	18	9	19	10	17
Paridae	*Parus carolinensis*				2	10	18	16	7	14	13	14	5	19	23	23	12	33	18	22	6
Paridae	*Parus atricapillus*				2	5	19	8	15	8	17	8	13	7	22	11	20	13	22	13	17
Paridae	*Parus gambeli*				2	3	19	6	16	5	18	7	15	4	25	9	28	10	32	14	24
Paridae	*Parus sclateri*			x	2	5	1x	20	0x	13	1x	33	0x	7	1x	20	11x	26	2x	33	1x
Paridae	*Parus superciliosus*				7	34	1	34	5	33	5	36	2	35	6	37	9	38	6	39	3
Paridae	*Parus davidi*			x	7	0	0x	0	0x	0	0x	0	0x	0	0x	0	0x	0	0x	0	0x
Paridae	*Parus cinctus*				2,3,7	0	15	0	15	0	15	0	15	0	18	0	22	0	23	0	22
Paridae	*Parus hudsonicus*				2	4	18	6	17	6	18	6	17	5	23	8	24	10	24	10	23
Paridae	*Parus rufescens*				2	13	24	16	21	13	24	21	16	16	32	25	29	24	37	30	25
Paridae	*Parus rufonuchalis*				7	71	4	70	1	74	0	74	2	73	5	77	2	81	1	80	3
Paridae	*Parus rubidiventris*				7	43	2	43	2	43	2	44	2	44	4	46	6	46	5	47	4

Family	Species	IUCN	Ag	R	Range Source	Percentage loss in geographic range 2050 TG H	C	AM H	C	GO H	C	OS H	C	2100 TG H	C	AM H	C	GO H	C	OS H	C
Paridae	*Parus melanolophus*				7	66	3	66	0	69	0	71	2	68	5	73	1	77	1	75	3
Paridae	*Parus ater*				3,4,5,7	7	15	6	14	7	16	7	14	8	18	8	18	11	19	12	17
Paridae	*Parus venustulus*				7	0	0	0	0	0	0	0	0	0	0	0	0	0	0	0	0
Paridae	*Parus elegans*				7	14	12	23	11	14	12	21	11	19	18	30	14	26	21	33	12
Paridae	*Parus amabilis*			x	7	22	25x	22	29x	21	20x	24	20x	22	25x	22	48x	44	39x	35	48x
Paridae	*Parus cristatus*				3,4	8	28	8	25	11	29	9	23	12	35	12	32	19	36	19	29
Paridae	*Parus dichrous*				7	42	2	42	2	42	2	44	2	43	4	45	6	46	5	46	4
Paridae	*Parus leucomelas*				5	20	2	14	4	21	2	24	1	23	2	16	5	26	2	25	2
Paridae	*Parus carpi*				5	12	5	5	7	11	4	10	5	12	10	5	14	11	7	10	8
Paridae	*Parus niger*				5	22	1	19	2	22	1	22	2	22	4	19	9	22	3	22	3
Paridae	*Parus albiventris*				5	33	1	26	3	32	1	31	1	36	1	27	3	38	1	31	1
Paridae	*Parus leuconotus*			x	5	57	0x	46	0x	58	0x	58	0x	58	0x	46	2x	58	0x	58	0x
Paridae	*Parus funereus*				5	36	7	24	10	33	8	49	6	50	9	33	12	52	9	60	6
Paridae	*Parus rufiventris*				5	54	1	38	2	54	1	56	1	56	1	40	6	56	1	57	1
Paridae	*Parus fringillinus*				5	43	11	36	15	49	9	49	10	46	12	46	15	60	9	55	11
Paridae	*Parus fasciiventer*				5	19	10	24	8	27	6	29	0	23	10	26	8	29	9	29	0
Paridae	*Parus thruppi*				5	59	6	13	36	69	1	69	1	64	6	19	42	69	1	69	1
Paridae	*Parus griseiventris*				5	48	0	41	0	48	0	52	0	51	1	42	5	51	0	52	0
Paridae	*Parus cinerascens*				5	8	5	3	6	7	5	8	4	8	7	3	13	7	10	8	8
Paridae	*Parus afer*				5	2	7	1	9	2	10	1	9	2	10	1	12	3	13	2	13
Paridae	*Parus major*				3,4,5,7	10	12	10	11	11	12	11	10	12	15	12	14	15	15	16	12
Paridae	*Parus bokharensis*			x	4,7	13	10x	8	9x	8	9x	10	9x	13	10x	12	9x	17	9x	15	12x
Paridae	*Parus monticolus*				7	24	1	24	1	24	1	25	0	25	1	26	1	26	1	27	1
Paridae	*Parus nuchalis*	T			7	12	1	8	7	3	7	14	6	13	3	8	7	4	8	14	6
Paridae	*Parus xanthogenys*				7	35	3	39	2	40	1	48	1	39	4	45	3	46	3	68	1
Paridae	*Parus spilonotus*				7	22	3	23	3	21	4	21	4	24	4	24	4	23	5	24	5
Paridae	*Parus holsti*			x	7	0	0x	0	0x	0	0x	0	0x	0	0x	0	0x	0	0x	0	0x
Paridae	*Parus caeruleus*				3,4,5	7	21	7	18	9	21	9	16	11	26	10	22	16	26	15	19
Paridae	*Parus ultramarinus*				13	0	0	0	0	0	0	0	0	0	0	0	0	0	0	0	0
Paridae	*Parus degener*			x	13	0	31x	0	31x	0	31x	15	31x	0	31x	0	31x	0	31x	15	31x
Paridae	*Parus teneriffae*			x	13	0	0x	0	0x	0	0x	0	0x	0	0x	0	0x	0	0x	0	0x
Paridae	*Parus ombriosus*			x	13	0	0x	100	0x	0	0x	0	0x	0	0x	100	0x	0	0x	0	0x
Paridae	*Parus palmensis*			x	13	0	0x	0	0x	0	0x	0	0x	0	0x	0	0x	0	0x	0	0x
Paridae	*Parus cyanus*				3,4,7	8	14	7	14	8	16	8	14	10	17	8	17	11	19	13	16
Paridae	*Parus flavipectus*				7	5	12	2	12	4	13	2	14	5	14	3	16	5	15	15	16
Paridae	*Parus varius*				7	14	11	15	10	15	12	14	11	15	17	16	20	17	20	17	19
Paridae	*Parus semilarvatus*			x	7	16	14x	37	7x	14	15x	17	16x	16	17x	53	7x	25	27x	37	16x
Paridae	*Parus wollweberi*				2	13	10	27	6	14	10	36	6	17	13	28	20	19	24	40	16
Paridae	*Parus inornatus*				2	0	49	4	33	2	48	4	24	1	52	4	39	3	52	7	29
Paridae	*Parus ridgwayi*				2	1	11	1	11	1	11	1	11	1	14	2	19	3	22	3	19
Paridae	*Parus bicolor*				2	11	20	18	9	16	15	16	6	19	24	25	12	32	18	24	6
Paridae	*Parus atricristatus*				2	0	14	2	9	1	10	2	6	0	20	2	30	2	23	2	10
Paridae	*Sylviparus modestus*				7	34	3	35	2	33	2	34	3	35	4	38	2	36	3	37	3
Paridae	*Melanochlora sultanea*				7	26	8	29	8	26	10	29	9	31	11	32	11	33	13	35	12

Family	Species	IUCN	Ag	R	Range Source	Percentage loss in geographic range 2050								2100							
						TG		AM		GO		OS		TG		AM		GO		OS	
						H	C	H	C	H	C	H	C	H	C	H	C	H	C	H	C
Aegithalidae	*Aegithalos caudatus*				3,4,7	8	16	7	15	8	17	8	14	10	19	9	19	13	20	14	18
Aegithalidae	*Aegithalos leucogenys*				7	37	6	41	5	43	3	40	2	39	7	45	5	50	3	51	3
Aegithalidae	*Aegithalos concinnus*				7	14	2	15	2	14	3	15	2	16	3	16	3	16	3	17	3
Aegithalidae	*Aegithalos niveogularis*				7	71	3	71	3	71	3	76	2	73	12	79	9	81	12	80	8
Aegithalidae	*Aegithalos iouschistos*				7	41	2	42	2	42	1	42	2	42	3	45	3	44	2	43	3
Aegithalidae	*Aegithalos fuliginosus*				7	12	0	12	0	12	0	12	0	12	0	13	1	13	0	12	1
Aegithalidae	*Psaltriparus minimus*				2	5	14	10	12	6	14	12	9	6	17	11	20	9	23	15	16
Aegithalidae	*Psaltria exilis*			x	7	0	0x	0	0x	0	0x	0	0x	2	0x	0	0x	0	0x	0	0x
Hirundinidae	*Pseudochelidon eurystomi*				5	47	8	29	14	38	9	71	2	67	8	38	16	72	11	81	4
Hirundinidae	*Tachycineta bicolor*				2	2	18	4	15	4	17	3	13	3	22	5	21	6	24	5	18
Hirundinidae	*Tachycineta albilinea*				2	6	9	13	4	7	7	26	3	8	10	15	16	10	7	28	4
Hirundinidae	*Tachycineta stolzmanni*				2	0	5	0	8	0	8	0	8	0	8	0	25	0	30	0	25
Hirundinidae	*Tachycineta albiventer*				2	7	13	5	11	7	12	11	10	11	16	10	14	17	15	20	12
Hirundinidae	*Tachycineta leucorrhoa*		H		2	0	13	0	10	0	11	0	9	0	18	0	14	0	16	0	13
Hirundinidae	*Tachycineta meyeni*				2	5	9	5	10	5	10	4	10	7	16	8	18	11	20	10	18
Hirundinidae	*Tachycineta thalassina*				2	5	22	10	18	6	21	12	17	6	28	12	30	11	34	18	26
Hirundinidae	*Tachycineta cyaneoviridis*	T		x	2	30	0x	63	0x	78	0x	54	0x	54	0x	63	0x	79	0x	70	0x
Hirundinidae	*Tachycineta euchrysea*	T		x	2	0	3x	3	0x	0	3x	17	0x	14	3x	3	0x	0	3x	17	0x
Hirundinidae	*Progne subis*				2	4	14	7	8	6	12	5	5	7	17	9	12	12	16	8	7
Hirundinidae	*Progne cryptoleuca*				2	8	4	23	3	12	4	25	2	13	4	25	5	16	4	30	2
Hirundinidae	*Progne dominicensis*				2	17	11	24	10	19	13	36	0	20	14	24	10	20	13	37	0
Hirundinidae	*Progne sinaloae*				2	17	14	33	8	20	12	43	8	27	17	34	18	28	27	52	16
Hirundinidae	*Progne chalybea*				2	9	12	7	9	9	10	14	7	12	15	12	13	17	14	21	10
Hirundinidae	*Progne modesta*			x	2	0	14x	0	14x	0	8x	0	14x	0	14x	0	32x	0	42x	0	32x
Hirundinidae	*Progne elegans*				14	2	8	2	8	2	8	3	7	2	10	2	13	3	16	4	13
Hirundinidae	*Progne murphyi*				14	0	25	1	25	3	33	3	22	0	42	1	63	11	60	8	57
Hirundinidae	*Progne tapera*				2	9	13	7	11	9	11	12	9	12	17	11	15	18	16	19	13
Hirundinidae	*Notiochelidon murina*				2	11	7	12	7	9	7	12	7	14	10	19	12	22	12	18	10
Hirundinidae	*Notiochelidon cyanoleuca*		H		2	0	12	0	9	0	11	0	7	0	17	0	14	0	16	0	11
Hirundinidae	*Notiochelidon flavipes*				2	17	6	14	4	13	6	21	9	22	7	23	13	32	13	27	13
Hirundinidae	*Notiochelidon pileata*		H		2	0	1	0	1	0	1	0	1	0	1	0	1	0	1	0	1
Hirundinidae	*Atticora fasciata*				2	5	11	3	11	4	11	8	10	9	12	7	12	14	13	16	12
Hirundinidae	*Atticora melanoleuca*				2	5	11	4	12	4	11	10	11	10	12	7	14	19	13	20	13
Hirundinidae	*Neochelidon tibialis*				2	6	8	5	8	5	8	10	7	12	9	10	10	17	10	20	9
Hirundinidae	*Stelgidopteryx fucata*				2	8	11	6	9	9	9	11	8	12	16	11	14	17	15	17	13
Hirundinidae	*Stelgidopteryx andecola*				2	11	23	10	25	7	23	14	19	12	30	15	33	19	34	26	24
Hirundinidae	*Stelgidopteryx serripennis*		H		2	0	18	0	12	0	15	0	9	0	21	0	18	0	22	0	14
Hirundinidae	*Stelgidopteryx ridgwayi*				2	4	3	9	2	5	3	12	2	4	6	12	15	9	6	13	4
Hirundinidae	*Stelgidopteryx ruficollis*				2	10	13	8	10	9	11	14	7	13	17	13	13	19	15	23	11
Hirundinidae	*Cheramoeca leucosternus*				10	0	8	0	6	0	7	0	5	0	9	0	7	0	9	0	6
Hirundinidae	*Pseudhirundo griseopyga*				5	27	2	21	3	29	1	30	1	29	2	24	7	31	2	31	2
Hirundinidae	*Riparia riparia*				2,3,4,5,6,7,8	5	14	5	13	6	15	6	12	7	18	7	20	9	23	9	18
Hirundinidae	*Riparia paludicola*				3,4,5,6,7	19	4	14	6	20	4	22	3	20	5	16	10	22	7	24	5
Hirundinidae	*Riparia congica*				5	32	3	20	4	28	1	44	1	40	3	27	5	57	1	50	1

Family	Species	IUCN	Ag	R	Range Source	Percentage loss in geographic range 2050 TG		AM		GO		OS		2100 TG		AM		GO		OS	
						H	C	H	C	H	C	H	C	H	C	H	C	H	C	H	C
Hirundinidae	*Riparia cincta*				4,5	22	2	15	4	22	2	24	2	23	4	18	9	24	4	24	4
Hirundinidae	*Phedina borbonica*				5,6,13	66	1	34	9	67	1	67	1	67	1	50	9	67	1	67	1
Hirundinidae	*Phedina brazzae*				5	39	8	18	10	22	12	43	6	46	8	23	12	40	12	48	7
Hirundinidae	*Hirundo rupestris*				3,4,5,7	8	9	9	8	10	9	11	8	11	12	11	13	14	15	14	12
Hirundinidae	*Hirundo obsoleta*				4	4	4	4	4	6	3	9	3	11	5	7	4	12	4	14	4
Hirundinidae	*Hirundo fuligula*				3,4,5,7	7	2	5	3	7	2	8	2	7	2	6	5	8	5	8	4
Hirundinidae	*Hirundo concolor*				7	17	5	22	4	18	6	18	6	17	7	23	7	21	8	23	7
Hirundinidae	*Hirundo rustica*		H		,3,4,5,6,7,8,10,1	0	13	0	12	0	14	0	11	0	17	0	18	0	21	0	16
Hirundinidae	*Hirundo lucida*				5	22	5	12	5	23	5	31	3	28	5	16	7	33	6	32	5
Hirundinidae	*Hirundo aethiopica*				4,5	20	3	9	9	23	2	25	2	24	3	11	12	25	4	25	3
Hirundinidae	*Hirundo angolensis*				5	34	1	24	4	34	1	38	0	36	2	29	7	38	1	39	0
Hirundinidae	*Hirundo albigularis*				5	11	4	9	4	11	4	11	4	11	6	9	9	11	8	11	7
Hirundinidae	*Hirundo domicola*				7	18	1	16	0	19	1	21	1	21	2	20	0	21	1	23	1
Hirundinidae	*Hirundo tahitica*				7,8,9,11	10	5	13	5	14	5	15	5	15	8	18	9	24	9	24	8
Hirundinidae	*Hirundo neoxena*				8,10,12	0	11	0	8	1	10	1	6	1	14	0	11	1	14	1	7
Hirundinidae	*Hirundo smithii*				4,5,7	19	3	15	5	20	3	23	2	21	5	17	8	23	6	26	3
Hirundinidae	*Hirundo nigrita*				5	34	7	21	9	33	8	53	4	49	7	31	11	57	9	61	5
Hirundinidae	*Hirundo nigrorufa*				5	42	0	31	1	41	0	47	0	46	1	33	3	43	0	47	0
Hirundinidae	*Hirundo atrocaerulea*	T			5	44	0	40	0	44	0	44	0	44	0	41	3	44	0	44	0
Hirundinidae	*Hirundo leucosoma*				5	14	3	9	4	18	3	23	3	19	3	12	7	24	5	23	3
Hirundinidae	*Hirundo megaensis*	T			5	90	0 x	51	12 x	96	0 x	96	0 x	96	0 x	70	17 x	96	0 x	96	0 x
Hirundinidae	*Hirundo dimidiata*				5	14	4	10	6	14	5	14	4	15	7	10	11	14	7	14	7
Hirundinidae	*Hirundo cucullata*				5	6	3	6	4	6	4	6	3	6	5	6	5	6	5	6	5
Hirundinidae	*Hirundo abyssinica*				4,5	27	3	15	9	28	3	31	2	30	4	18	12	32	4	33	3
Hirundinidae	*Hirundo semirufa*				5	20	3	14	4	21	3	26	2	24	4	16	7	27	4	27	3
Hirundinidae	*Hirundo senegalensis*				5	33	3	22	6	33	4	42	2	40	4	27	9	43	5	45	3
Hirundinidae	*Hirundo daurica*				3,4,5,7,8,10	12	5	11	5	13	5	15	5	14	7	13	10	16	9	18	8
Hirundinidae	*Hirundo striolata*				7,8	11	7	15	7	13	8	14	8	15	12	18	11	20	14	21	11
Hirundinidae	*Hirundo preussi*				5	18	5	10	6	22	5	30	4	28	6	14	9	33	8	32	6
Hirundinidae	*Hirundo rufigula*				5	44	2	30	3	42	3	55	1	51	3	36	5	51	3	56	2
Hirundinidae	*Hirundo spilodera*				5	3	4	2	5	3	5	3	4	3	5	2	6	4	6	3	6
Hirundinidae	*Hirundo pyrrhonota*				2	2	17	4	14	3	16	4	12	3	21	5	21	5	24	6	19
Hirundinidae	*Hirundo fulva*				2	10	6	21	6	14	7	27	2	13	8	22	12	17	7	29	2
Hirundinidae	*Petrochelidon pallida*				14	0	26	3	21	2	22	4	20	1	30	3	35	3	36	4	28
Hirundinidae	*Hirundo rufocollaris*				2	3	12	1	11	1	17	1	11	3	18	1	27	3	31	3	26
Hirundinidae	*Hirundo nigricans*				8,9,10,12	1	14	2	9	3	12	3	7	3	18	3	13	5	17	6	9
Hirundinidae	*Hirundo fluvicola*				7	10	3	11	2	11	2	14	1	11	5	12	8	12	11	20	2
Hirundinidae	*Hirundo ariel*				8,10,12	1	13	2	9	3	11	3	6	3	16	2	13	4	16	5	8
Hirundinidae	*Hirundo fuliginosa*				5	22	12	12	13	17	12	55	9	33	14	33	14	59	12	70	9
Hirundinidae	*Delichon urbica*				3,4,5,7	6	16	6	15	7	17	7	14	9	20	8	19	11	22	11	18
Hirundinidae	*Delichon dasypus*				4,7	14	11	16	10	16	10	16	10	16	18	18	22	21	22	21	20
Hirundinidae	*Delichon nipalensis*				7	34	7	39	4	37	5	38	5	36	9	43	11	41	14	42	12
Hirundinidae	*Psalidoprocne nitens*				5	34	5	22	7	32	6	54	4	51	5	32	8	59	6	63	4
Hirundinidae	*Psalidoprocne fuliginosa*			x	5	26	10 x	23	10 x	84	0 x	51	0 x	40	58 x	23	58 x	87	0 x	100	0 x

Family	Species	IUCN	Ag	R	Range Source	Percentage loss in geographic range 2050 TG		AM		GO		OS		2100 TG		AM		GO		OS	
						H	C	H	C	H	C	H	C	H	C	H	C	H	C	H	C
Hirundinidae	*Psalidoprocne albiceps*				5	34	4	25	9	39	2	39	2	36	4	31	11	43	2	42	2
Hirundinidae	*Psalidoprocne pristoptera*				5	40	4	29	5	39	4	52	3	49	5	35	8	52	5	55	3
Hirundinidae	*Psalidoprocne obscura*				5	24	6	14	7	26	7	36	5	32	6	20	11	37	9	39	7
Regulidae	*Regulus calendula*				2	3	19	4	18	4	19	5	17	4	25	6	26	7	28	8	25
Regulidae	*Regulus regulus*				3,4,5,7	9	20	8	19	10	21	10	19	12	25	12	25	16	26	18	23
Regulidae	*Regulus teneriffae*			x	14	0	0x	0	0x	0	0x	0	0x	0	0x	0	0x	0	0x	0	0x
Regulidae	*Regulus goodfellowi*				7	0	0	0	0	0	0	0	0	0	0	0	0	0	0	0	0
Regulidae	*Regulus ignicapillus*				3,4	12	25	10	17	15	22	13	15	16	33	16	25	27	30	20	20
Regulidae	*Regulus satrapa*				2	7	20	11	17	10	18	12	15	9	25	14	25	16	27	18	22
Pycnonotidae	*Spizixos canifrons*				7	22	8	23	6	21	9	21	8	25	9	25	8	25	10	26	9
Pycnonotidae	*Spizixos semitorques*				7	4	0	4	0	4	0	4	0	4	0	5	0	4	0	4	0
Pycnonotidae	*Pycnonotus zeylanicus*	T			7	6	1	8	2	8	2	6	1	9	1	11	4	17	2	13	1
Pycnonotidae	*Pycnonotus striatus*				7	33	7	36	6	32	8	33	8	37	9	38	9	36	10	39	10
Pycnonotidae	*Pycnonotus leucogrammic*				7	24	6	25	6	24	4	27	7	29	7	33	8	47	4	40	7
Pycnonotidae	*Pycnonotus tympanistrigus*				7	26	5	24	5	25	4	27	6	31	6	32	7	46	4	40	6
Pycnonotidae	*Pycnonotus melanoleucos*				7	13	2	16	3	16	3	19	2	20	3	22	3	28	3	28	3
Pycnonotidae	*Pycnonotus priocephalus*				7	26	0	49	1	43	1	51	0	29	1	59	1	51	1	88	0
Pycnonotidae	*Pycnonotus atriceps*				7	17	7	20	7	18	8	20	8	22	9	24	10	26	12	28	10
Pycnonotidae	*Pycnonotus melanicterus*				7	20	6	23	6	21	7	24	6	24	8	27	9	29	10	33	8
Pycnonotidae	*Pycnonotus squamatus*				7	13	2	16	3	15	3	18	2	19	3	22	3	28	3	28	2
Pycnonotidae	*Pycnonotus cyaniventris*				7	13	2	16	3	16	3	18	3	20	4	23	3	29	3	29	3
Pycnonotidae	*Pycnonotus jocosus*				4,7,10,11,13	15	4	17	4	16	4	19	4	17	6	19	6	20	8	26	5
Pycnonotidae	*Pycnonotus xanthorrhous*				7	14	3	14	3	13	4	13	4	15	4	15	4	15	4	15	4
Pycnonotidae	*Pycnonotus sinensis*		H		7	0	1	0	1	0	1	0	1	0	1	0	1	0	1	0	1
Pycnonotidae	*Pycnonotus taivanus*	T	H	x	7	0	0x	0	0x	0	0x	0	0x	0	0x	0	0x	0	0x	0	0x
Pycnonotidae	*Pycnonotus barbatus*				3,4,5	27	3	18	4	28	3	35	2	33	4	22	7	35	4	37	3
Pycnonotidae	*Pycnonotus nigricans*				5	7	5	2	8	7	6	7	6	7	8	2	13	7	11	7	10
Pycnonotidae	*Pycnonotus capensis*				5	2	4	0	4	2	6	0	5	2	6	0	9	2	13	0	13
Pycnonotidae	*Pycnonotus xanthopygos*				3,4	2	2	2	2	2	2	3	2	3	2	3	2	3	3	3	2
Pycnonotidae	*Pycnonotus leucotis*		H		4,7	0	4	0	4	0	4	0	3	0	7	0	8	0	9	0	4
Pycnonotidae	*Pycnonotus leucogenys*		H		3,4,7	0	3	0	2	0	2	0	1	0	5	0	3	0	3	0	3
Pycnonotidae	*Pycnonotus cafer*				4,7,11,12,13	20	3	21	3	20	3	23	2	22	5	24	6	23	8	31	3
Pycnonotidae	*Pycnonotus aurigaster*				7,8	10	4	13	4	11	5	12	5	11	7	14	7	13	9	15	7
Pycnonotidae	*Pycnonotus eutilotus*				7	13	2	16	3	15	3	18	2	19	3	21	3	28	3	27	2
Pycnonotidae	*Pycnonotus nieuwenhuisii*				7	13	2	16	2	16	2	18	2	20	2	22	3	28	3	28	2
Pycnonotidae	*Pycnonotus urostictus*				7	13	12	21	13	12	13	17	13	17	17	25	16	24	20	26	15
Pycnonotidae	*Pycnonotus bimaculatus*				7	21	4	19	4	20	3	21	5	25	5	26	6	38	3	32	5
Pycnonotidae	*Pycnonotus finlaysoni*				7	14	10	19	10	16	11	17	11	17	13	21	15	21	17	24	15
Pycnonotidae	*Pycnonotus xantholaemus*	T			7	15	0	27	1	24	1	28	1	17	2	31	1	28	3	46	1
Pycnonotidae	*Pycnonotus penicillatus*			x	7	41	0x	34	0x	40	1x	41	0x	42	0x	36	0x	41	1x	42	0x
Pycnonotidae	*Pycnonotus flavescens*				7	21	9	23	9	20	11	23	10	25	12	25	14	27	16	30	13
Pycnonotidae	*Pycnonotus luteolus*				7	18	3	21	3	22	2	28	1	21	4	25	4	26	4	42	1
Pycnonotidae	*Pycnonotus goiavier*				7	12	5	15	6	13	6	15	6	17	7	19	8	24	9	23	8
Pycnonotidae	*Pycnonotus plumosus*				7	12	2	15	3	14	3	17	2	18	3	20	3	27	3	26	3

Family	Species	IUCN	Ag	R	Range Source	2050 TG H	2050 TG C	2050 AM H	2050 AM C	2050 GO H	2050 GO C	2050 OS H	2050 OS C	2100 TG H	2100 TG C	2100 AM H	2100 AM C	2100 GO H	2100 GO C	2100 OS H	2100 OS C
Pycnonotidae	*Pycnonotus blanfordi*				7	12	10	17	9	13	11	15	11	14	14	18	15	18	19	21	15
Pycnonotidae	*Pycnonotus simplex*				7	13	2	15	3	15	3	18	2	20	3	22	3	28	3	27	2
Pycnonotidae	*Pycnonotus brunneus*				7	12	2	16	3	15	3	17	2	19	3	21	3	27	3	27	2
Pycnonotidae	*Pycnonotus erythropthalmo*				7	13	2	16	3	15	3	18	2	20	3	22	3	28	3	28	2
Pycnonotidae	*Andropadus montanus*				5	18	15	14	15	22	18	29	9	24	20	21	19	32	18	31	12
Pycnonotidae	*Andropadus masukuensis*				5	33	2	25	4	31	4	35	1	34	5	29	5	33	5	35	1
Pycnonotidae	*Andropadus virens*				5	38	6	25	8	37	7	55	4	51	7	33	11	57	9	61	5
Pycnonotidae	*Andropadus gracilis*				5	33	6	21	8	31	7	53	4	49	6	31	10	56	8	61	5
Pycnonotidae	*Andropadus ansorgei*				5	32	8	22	9	34	7	56	5	49	8	33	10	59	8	63	5
Pycnonotidae	*Andropadus curvirostris*				5	37	6	23	8	36	6	56	4	52	7	33	10	61	7	63	4
Pycnonotidae	*Andropadus gracilirostris*				5	37	7	25	9	36	7	56	4	51	7	34	11	58	7	63	5
Pycnonotidae	*Andropadus importunus*				5	29	1	25	2	29	1	29	1	29	3	26	5	29	1	29	1
Pycnonotidae	*Andropadus latirostris*				5	34	6	22	9	34	6	49	4	46	7	30	11	53	7	55	4
Pycnonotidae	*Andropadus tephrolaemus*				5	31	6	27	6	32	7	35	4	33	8	31	8	36	7	35	5
Pycnonotidae	*Andropadus milanjensis*				5	47	0	41	2	47	0	47	0	47	1	43	8	47	0	47	0
Pycnonotidae	*Calyptocichla serina*				5	32	6	17	8	28	7	50	5	46	7	26	10	53	7	59	5
Pycnonotidae	*Baeopogon indicator*				5	35	7	24	9	36	7	55	5	51	8	33	12	59	9	61	6
Pycnonotidae	*Baeopogon clamans*				5	28	4	15	5	29	3	46	5	49	5	25	5	58	3	57	5
Pycnonotidae	*Ixonotus guttatus*				5	33	5	21	7	32	6	54	4	50	6	32	9	58	8	62	4
Pycnonotidae	*Chlorocichla simplex*				5	37	6	24	8	36	7	57	4	53	7	34	11	60	8	64	5
Pycnonotidae	*Chlorocichla flavicollis*				5	42	6	27	7	40	6	57	4	53	6	34	11	57	8	62	5
Pycnonotidae	*Chlorocichla falkensteini*				5	39	1	28	2	38	2	52	1	51	1	38	3	53	2	60	1
Pycnonotidae	*Chlorocichla flaviventris*				5	46	0	35	1	46	0	49	0	48	1	37	4	48	1	49	1
Pycnonotidae	*Chlorocichla laetissima*				5	37	4	44	8	38	8	55	3	51	4	45	8	53	10	59	3
Pycnonotidae	*Chlorocichla prigoginei*	T		x	5	57	0x	43	0x	51	0x	60	0x	60	0x	43	0x	51	0x	60	0x
Pycnonotidae	*Thescelocichla leucopleura*		H		5	0	5	0	6	0	6	0	4	0	6	0	8	0	8	0	5
Pycnonotidae	*Pyrrhurus scandens*				5	30	8	18	10	28	10	49	6	44	10	25	16	49	13	55	9
Pycnonotidae	*Phyllastrephus cabanisi*				5	53	2	38	3	54	3	61	2	59	3	40	6	59	3	62	2
Pycnonotidae	*Phyllastrephus fischeri*				5	35	0	26	0	35	0	35	0	35	0	29	0	35	0	35	0
Pycnonotidae	*Phyllastrephus terrestris*				5	26	2	22	3	26	2	26	2	26	4	22	9	26	3	26	3
Pycnonotidae	*Phyllastrephus strepitans*				5	46	4	16	26	52	1	52	1	48	4	20	29	53	1	52	1
Pycnonotidae	*Phyllastrephus fulviventris*			x	5	60	0x	60	0x	69	0x	69	0x	60	0x	69	3x	69	0x	69	0x
Pycnonotidae	*Phyllastrephus cerviniventri*				5	50	0	43	1	51	0	52	0	51	1	44	5	51	0	52	0
Pycnonotidae	*Phyllastrephus baumanni*				5	15	11	15	9	22	9	29	7	22	11	21	12	29	10	31	8
Pycnonotidae	*Phyllastrephus poensis*				5	19	11	20	7	14	13	24	11	20	14	25	11	24	13	25	13
Pycnonotidae	*Phyllastrephus hypochloris*				5	51	2	39	11	48	3	60	0	56	2	41	11	55	4	60	0
Pycnonotidae	*Phyllastrephus lorenzi*				5	13	0x	6	0x	16	0x	20	0x	19	0x	6	0x	21	0x	20	0x
Pycnonotidae	*Phyllastrephus poliocephal*				5	7	13	7	7	26	6	22	6	15	18	12	12	34	6	27	6
Pycnonotidae	*Phyllastrephus flavostriatus*				5	41	1	36	3	41	2	45	0	44	1	38	5	43	2	45	0
Pycnonotidae	*Phyllastrephus debilis*				5	45	0	38	0	45	0	45	0	45	0	41	1	45	0	45	0
Pycnonotidae	*Phyllastrephus albigularis*				5	32	7	22	9	32	7	54	5	48	8	31	11	55	8	60	5
Pycnonotidae	*Phyllastrephus icterinus*				5	34	6	22	8	34	6	55	4	50	7	33	10	60	8	63	5
Pycnonotidae	*Phyllastrephus leucolepis*	T		x	5	23	1x	21	0x	17	5x	31	3x	23	11x	24	10x	30	5x	31	3x
Pycnonotidae	*Phyllastrephus xavieri*				5	35	4	26	6	36	5	62	4	56	4	36	7	66	5	69	4

Percentage loss in geographic range (2050: TG, AM, GO, OS; 2100: TG, AM, GO, OS; H, C)

Family	Species	IUCN	Ag	R	Range Source	2050 TG H	2050 TG C	2050 AM H	2050 AM C	2050 GO H	2050 GO C	2050 OS H	2050 OS C	2100 TG H	2100 TG C	2100 AM H	2100 AM C	2100 GO H	2100 GO C	2100 OS H	2100 OS C
Pycnonotidae	*Phyllastrephus madagasca*				6	70	1	34	10	71	1	71	1	71	1	51	10	71	1	71	1
Pycnonotidae	*Phyllastrephus zosterops*				6	76	0	46	6	79	0	79	0	79	0	71	6	79	0	79	0
Pycnonotidae	*Phyllastrephus apperti*	T		x	6	0	0x	0	0x	0	0x	0	0x	0	0x	0	0x	0	0x	0	0x
Pycnonotidae	*Phyllastrephus tenebrosus*	T		x	6	85	0x	83	0x	85	0x	85	0x	85	0x	85	0x	85	0x	85	0x
Pycnonotidae	*Phyllastrephus cinereiceps*			x	6	67	0x	62	0x	67	0x	67	0x	67	0x	67	0x	67	0x	67	0x
Pycnonotidae	*Bleda syndactyla*				5	38	7	25	9	36	8	57	5	53	7	34	12	59	9	64	5
Pycnonotidae	*Bleda eximia*	T			5	36	5	22	8	33	7	56	4	53	6	33	10	60	8	64	5
Pycnonotidae	*Bleda canicapilla*				5	26	7	14	10	28	8	42	6	35	8	20	13	41	11	44	9
Pycnonotidae	*Nicator chloris*				5	34	7	21	9	32	8	54	5	49	7	31	12	56	9	61	6
Pycnonotidae	*Nicator gularis*				5	31	1	26	1	31	1	31	1	31	3	27	5	31	2	31	2
Pycnonotidae	*Nicator vireo*				5	35	5	19	8	27	7	53	5	52	5	31	8	54	7	63	5
Pycnonotidae	*Criniger barbatus*				5	26	9	11	13	24	11	36	8	35	10	19	19	35	17	40	13
Pycnonotidae	*Criniger chloronotus*				5	34	5	21	8	30	7	62	3	54	5	32	9	60	7	70	4
Pycnonotidae	*Criniger calurus*				5	34	6	22	8	32	6	54	4	50	7	32	9	57	7	61	4
Pycnonotidae	*Criniger olivaceus*	T			5	19	2	12	1	23	2	27	1	24	8	18	7	31	2	27	1
Pycnonotidae	*Criniger ndussumensis*				5	36	6	22	8	32	7	57	4	53	6	32	9	61	8	64	4
Pycnonotidae	*Alophoixus finschii*				7	13	2	16	3	16	3	19	2	20	3	22	3	29	3	29	2
Pycnonotidae	*Alophoixus flaveolus*				7	34	8	37	8	32	11	35	9	39	10	41	11	37	13	41	11
Pycnonotidae	*Alophoixus pallidus*				7	14	7	17	6	14	8	15	7	16	9	18	10	17	13	20	11
Pycnonotidae	*Alophoixus ochraceus*				7	17	5	20	6	17	6	23	6	24	7	26	9	33	9	33	8
Pycnonotidae	*Alophoixus bres*				7	13	2	16	2	15	2	17	2	20	2	22	3	27	3	27	2
Pycnonotidae	*Alophoixus phaeocephalus*				7	13	2	16	3	15	3	18	2	20	3	22	3	28	3	28	2
Pycnonotidae	*Alophoixus affinis*				7	17	1	23	1	19	2	18	2	31	1	23	1	30	2	33	2
Pycnonotidae	*Setornis criniger*	T			7	13	1	16	1	16	2	15	1	21	3	23	3	28	3	26	1
Pycnonotidae	*Tricholestes criniger*				7	13	2	16	3	15	3	19	2	20	3	22	3	28	3	28	3
Pycnonotidae	*Iole virescens*				7	29	8	32	9	25	12	26	11	35	11	35	13	31	15	34	14
Pycnonotidae	*Iole propinqua*				7	16	10	20	9	17	11	18	10	18	13	22	14	21	17	24	14
Pycnonotidae	*Iole olivacea*				7	13	2	16	3	16	3	18	3	20	4	22	3	29	3	28	3
Pycnonotidae	*Iole indica*				7	34	3	46	2	40	2	52	0	37	4	53	2	47	4	73	0
Pycnonotidae	*Ixos palawanensis*			x	7	24	19x	24	23x	25	11x	22	18x	24	19x	24	44x	50	32x	33	50x
Pycnonotidae	*Ixos philippinus*				7	14	9	25	7	14	9	22	8	19	15	33	10	26	18	35	9
Pycnonotidae	*Ixos rufigularis*			x	7	22	42x	9	58x	7	58x	5	63x	38	43x	9	58x	29	63x	8	63x
Pycnonotidae	*Ixos siquijorensis*	T		x	7	39	6x	17	4x	0	6x	24	6x	39	6x	56	27x	0	45x	39	6x
Pycnonotidae	*Ixos amaurotis*				7	15	14	15	13	16	14	14	14	16	21	16	26	19	24	17	24
Pycnonotidae	*Ixos everetti*				7	21	9	29	11	14	9	30	10	31	12	29	11	27	15	44	11
Pycnonotidae	*Ixos malaccensis*				7	13	2	16	3	15	3	18	2	20	3	22	3	28	3	28	2
Pycnonotidae	*Hemixos flavala*				7	27	7	29	7	26	7	29	7	32	9	33	9	35	10	36	10
Pycnonotidae	*Hemixos castanonotus*				7	5	0	6	0	5	0	6	0	5	0	6	0	6	0	6	0
Pycnonotidae	*Hypsipetes mcclellandii*				7	20	5	21	4	19	6	21	5	22	6	23	6	23	8	25	7
Pycnonotidae	*Hypsipetes virescens*				7	14	2	17	3	14	3	17	3	19	2	21	3	28	3	28	3
Pycnonotidae	*Hypsipetes madagascarien*				6,13	67	0	42	7	68	0	68	0	68	0	56	7	68	0	68	0
Pycnonotidae	*Hypsipetes parvirostris*			x	13	19	0x	19	0x	19	0x	19	0x	19	0x	19	0x	19	0x	19	0x
Pycnonotidae	*Hypsipetes borbonicus*			x	13	45	0x	45	0x	45	0x	45	0x	45	0x	45	0x	45	0x	45	0x
Pycnonotidae	*Hypsipetes olivaceus*	T		x	13	0	0x	0	0x	0	0x	0	0x	0	0x	0	0x	0	0x	0	0x

Family	Species	IUCN	Ag	R	Range Source	Percentage loss in geographic range 2050 TG H	C	AM H	C	GO H	C	OS H	C	2100 TG H	C	AM H	C	GO H	C	OS H	C
Pycnonotidae	*Hypsipetes leucocephalus*				4,7	16	3	19	3	17	3	19	3	17	5	20	5	20	6	23	4
Pycnonotidae	*Hypsipetes nicobariensis*	T		x	7	49	0x	91	0x	78	0x	58	0x	49	0x	100	0x	100	0x	100	0x
Pycnonotidae	*Hypsipetes thompsoni*				7	19	12	25	11	15	15	17	15	25	16	28	17	21	19	24	19
Pycnonotidae	*Neolestes torquatus*				5	14	5	9	6	8	9	21	0	17	5	11	6	13	9	22	0
Pycnonotidae	*Malia grata*				7	14	13	26	9	19	13	16	11	16	14	29	22	28	31	24	27
Hypocoliidae	*Hypocolius ampelinus*				3,4	17	3	11	1	16	2	22	1	27	4	19	2	28	3	29	2
Priniidae	*Cryptosylvicola randrianas*				6	55	0	45	0	55	0	55	0	55	0	55	0	55	0	55	0
Priniidae	*Cisticola erythrops*				5	35	3	24	5	36	3	43	2	40	4	29	9	43	5	44	3
Priniidae	*Cisticola cantans*				5	29	3	18	6	31	4	39	2	37	4	22	10	40	6	41	4
Priniidae	*Cisticola lateralis*				5	33	6	21	9	33	8	49	5	45	7	27	15	46	11	51	7
Priniidae	*Cisticola anonymus*				5	34	6	22	8	33	6	56	4	52	6	34	9	61	7	65	4
Priniidae	*Cisticola woosnami*				5	44	1	36	1	45	0	47	0	46	1	39	4	46	1	47	0
Priniidae	*Cisticola bulliens*		H		5	0	1	0	1	0	0	0	1	0	1	0	3	0	0	0	1
Priniidae	*Cisticola chubbi*				5	30	6	21	11	26	9	39	1	33	7	25	12	34	10	39	2
Priniidae	*Cisticola hunteri*				5	50	3	30	14	51	1	51	2	50	4	35	15	51	1	51	2
Priniidae	*Cisticola nigriloris*				5	42	0	36	0	42	0	42	0	42	2	41	3	42	0	42	0
Priniidae	*Cisticola aberrans*				5	19	2	16	4	19	3	21	2	20	3	17	8	21	4	21	3
Priniidae	*Cisticola bodessa*				5	49	3	27	12	54	0	54	0	51	3	31	14	54	0	54	0
Priniidae	*Cisticola chiniana*				5	41	3	29	6	42	2	47	2	45	4	32	10	47	3	48	2
Priniidae	*Cisticola cinereolus*				5	45	6	15	24	52	1	52	1	48	6	22	27	53	1	53	1
Priniidae	*Cisticola ruficeps*				5	11	2	5	4	12	2	14	1	13	2	6	5	15	3	15	2
Priniidae	*Cisticola dorsti*		H		5	0	0	0	0	0	0	0	0	0	0	0	0	0	6	0	0
Priniidae	*Cisticola rufilatus*				5	15	3	10	6	15	4	15	4	16	6	10	13	15	8	15	7
Priniidae	*Cisticola subruficapillus*				5	4	6	2	6	4	6	3	6	4	7	2	9	4	9	3	9
Priniidae	*Cisticola lais*				5	31	1	24	4	31	1	31	1	31	2	25	7	31	1	31	1
Priniidae	*Cisticola restrictus*				5	1	0	1	0	1	0	1	0	1	0	1	0	1	0	1	0
Priniidae	*Cisticola njombe*				5	47	0	32	6	47	0	47	0	47	0	40	8	47	0	47	0
Priniidae	*Cisticola galactotes*				5	38	4	31	6	42	2	45	2	42	4	35	8	47	3	46	2
Priniidae	*Cisticola pipiens*				5	26	0	18	2	24	0	28	0	28	1	20	8	26	2	28	1
Priniidae	*Cisticola carruthersi*				5	28	2	19	4	31	0	32	0	29	2	24	8	31	1	32	0
Priniidae	*Cisticola tinniens*				5	12	3	11	4	12	4	13	3	13	5	12	7	13	5	13	5
Priniidae	*Cisticola robustus*				5	31	2	21	5	34	1	34	2	33	2	26	7	35	3	35	2
Priniidae	*Cisticola aberdare*	T			5	23	0	22	0	23	0	23	0	23	0	22	0	23	0	23	0
Priniidae	*Cisticola natalensis*				5	31	2	21	5	33	2	36	1	34	2	25	7	36	2	37	2
Priniidae	*Cisticola fulvicapillus*				5	19	2	15	2	18	2	19	2	19	4	16	7	19	4	19	4
Priniidae	*Cisticola angusticauda*				5	49	2	39	4	48	2	49	2	49	3	44	4	49	2	49	2
Priniidae	*Cisticola melanura*				5	75	7x	32	15x	64	15x	75	14x	76	7x	37	15x	73	15x	80	14x
Priniidae	*Cisticola brachypterus*				5	27	2	17	4	28	2	31	1	30	2	21	6	32	2	32	2
Priniidae	*Cisticola rufus*				5	11	2	5	3	11	2	16	1	16	2	8	3	18	2	17	2
Priniidae	*Cisticola troglodytes*				5	29	2	17	6	32	1	32	1	31	2	21	8	33	1	33	1
Priniidae	*Cisticola nanus*				5	49	6	15	24	54	1	54	1	50	6	19	29	55	2	55	2
Priniidae	*Cisticola juncidis*				3,4,5,7,8,10	14	4	13	5	15	4	17	3	16	6	14	8	17	8	19	5
Priniidae	*Cisticola haesitatus*	T		x	4,5	0	0x	0	0x	0	0x	0	0x	0	0x	0	0x	0	0x	0	0x
Priniidae	*Cisticola cherinus*				6,13	44	0	25	4	44	0	44	0	44	0	33	4	44	0	44	0

Family	Species	IUCN	Ag	R	Range Source	Percentage loss in geographic range 2050 TG H	TG C	AM H	AM C	GO H	GO C	OS H	OS C	2100 TG H	TG C	AM H	AM C	GO H	GO C	OS H	OS C
Priniidae	*Cisticola aridulus*				5	17	5	8	9	18	4	20	4	19	6	10	14	20	8	20	7
Priniidae	*Cisticola textrix*				5	5	2	5	2	5	2	5	2	5	3	5	3	6	4	6	3
Priniidae	*Cisticola eximius*				5	12	2	10	2	16	1	18	1	16	2	12	4	18	2	19	2
Priniidae	*Cisticola dambo*				5	35	0	20	1	26	0	44	1	37	0	23	4	35	0	45	1
Priniidae	*Cisticola brunnescens*				5	31	2	23	3	34	2	34	1	34	2	27	5	35	2	35	2
Priniidae	*Cisticola ayresii*				5	21	1	17	2	21	0	22	0	22	1	19	4	22	1	22	1
Priniidae	*Cisticola exilis*				7,8,10	9	10	11	6	10	9	11	5	11	13	12	10	13	14	14	6
Priniidae	*Incana incana*			x	4,5	0	0x	0	0x	0	0x	0	0x	0	0x	0	0x	0	0x	0	0x
Priniidae	*Scotocerca inquieta*				3,4,5	6	3	6	3	7	3	9	2	10	4	8	4	10	4	13	3
Priniidae	*Rhopophilus pekinensis*				7	7	5	8	6	8	7	8	6	8	8	8	15	10	19	10	17
Priniidae	*Prinia burnesii*				7	8	1	10	1	9	1	11	1	8	4	10	5	10	11	11	1
Priniidae	*Prinia criniger*				7	16	1	17	1	16	1	17	1	17	1	18	1	18	1	19	1
Priniidae	*Prinia polychroa*				7	13	6	19	6	15	7	16	7	15	15	20	10	19	17	20	9
Priniidae	*Prinia atrogularis*				7	17	5	20	5	18	6	20	6	20	7	22	8	23	9	25	8
Priniidae	*Prinia cinereocapilla*	T			7	64	7	62	2	66	0	71	0	65	7	70	4	72	1	76	1
Priniidae	*Prinia buchanani*				7	12	4	11	4	11	4	15	2	12	7	12	11	11	13	21	3
Priniidae	*Prinia rufescens*				7	22	9	26	8	22	10	25	9	26	12	28	12	28	15	32	13
Priniidae	*Prinia hodgsonii*				7	19	5	22	4	20	5	24	4	21	7	24	8	23	10	30	6
Priniidae	*Prinia gracilis*				3,4,5,7	6	2	6	2	7	2	9	1	8	3	8	5	9	6	12	2
Priniidae	*Prinia sylvatica*				7	16	5	15	5	14	5	20	2	17	8	17	9	16	12	27	4
Priniidae	*Prinia familiaris*				7	10	2	9	1	10	1	9	3	17	3	13	4	18	1	15	3
Priniidae	*Prinia flaviventris*				7	13	5	17	5	15	6	16	5	16	8	19	8	20	10	22	7
Priniidae	*Prinia socialis*		H		7	0	2	0	2	0	2	0	1	0	4	0	6	0	7	0	2
Priniidae	*Prinia subflava*				5	21	3	14	4	23	2	26	2	25	3	17	7	27	4	28	3
Priniidae	*Prinia somalica*				5	34	5	8	26	41	1	41	1	37	5	10	29	41	2	41	1
Priniidae	*Prinia inornata*				7	11	3	13	3	11	4	14	3	12	6	14	7	13	9	18	4
Priniidae	*Prinia fluviatilis*				5	11	5	7	2	11	5	16	3	14	5	7	12	14	16	19	12
Priniidae	*Prinia flavicans*				5	8	6	4	8	8	7	8	7	8	9	4	15	8	13	8	12
Priniidae	*Prinia maculosa*				5	3	6	2	6	3	7	2	6	3	7	2	8	3	10	3	9
Priniidae	*Prinia molleri*			x	5	0	0x	0	0x	0	0x	0	0x	0	0x	0	0x	0	0x	0	0x
Priniidae	*Prinia robertsi*				14	67	0x	67	0x	67	0x	67	0x	67	0x	67	5x	67	0x	67	0x
Priniidae	*Prinia leontica*	T		x	14	4	0x	3	0x	19	0x	43	0x	4	0x	3	0x	21	0x	43	0x
Priniidae	*Prinia leucopogon*				14	38	6	22	9	33	8	56	4	53	7	31	11	55	9	63	5
Priniidae	*Prinia bairdii*				5	34	7	24	9	36	8	55	5	49	8	32	13	56	10	62	6
Priniidae	*Phragmacia substriata*				5	2	6	1	7	2	7	1	7	2	7	1	8	2	8	2	9
Priniidae	*Heliolais erythroptera*				5	26	2	19	3	31	2	36	2	33	3	22	6	39	3	37	3
Priniidae	*Malcorus pectoralis*				5	7	2	2	5	6	3	6	2	7	5	2	14	6	11	6	9
Priniidae	*Drymocichla incana*				5	36	5	17	10	38	8	54	5	51	8	22	17	54	12	57	8
Priniidae	*Urolais epichlora*				5	27	14	16	17	22	19	33	8	32	18	23	20	42	19	33	12
Priniidae	*Spiloptila clamans*				5	9	5	3	5	8	5	15	3	13	5	4	10	15	11	16	7
Priniidae	*Spiloptila rufifrons*				14	30	4	7	21	36	1	37	1	34	4	9	27	37	5	37	4
Priniidae	*Phyllolais pulchella*				5	22	2	13	7	24	1	24	1	22	2	16	8	25	2	25	2
Priniidae	*Apalis pulchra*				5	31	11	20	17	30	14	48	4	40	14	23	17	41	14	48	6
Priniidae	*Apalis ruwenzorii*				5	32	4	29	8	31	7	45	0	40	4	31	13	38	9	45	0

Family	Species	IUCN	Ag	R	Range Source	2050 TG H	2050 TG C	2050 AM H	2050 AM C	2050 GO H	2050 GO C	2050 OS H	2050 OS C	2100 TG H	2100 TG C	2100 AM H	2100 AM C	2100 GO H	2100 GO C	2100 OS H	2100 OS C
Priniidae	*Apalis thoracica*				5	25	1	23	1	26	1	26	1	25	3	24	5	26	1	26	1
Priniidae	*Apalis nigriceps*				5	35	5	26	8	38	7	53	6	49	5	35	12	52	10	58	8
Priniidae	*Apalis jacksoni*				5	43	5	36	7	48	5	55	3	54	6	42	10	56	6	58	3
Priniidae	*Apalis chariessa*	T			5	39	0x	37	0x	39	0x	39	0x	39	0x	39	2x	39	0x	39	0x
Priniidae	*Apalis binotata*				5	44	5	40	7	55	2	64	2	53	7	52	8	66	2	70	2
Priniidae	*Apalis personata*				5	32	4	22	7	28	5	37	1	34	4	27	7	33	6	37	1
Priniidae	*Apalis flavida*				5	36	3	24	8	39	2	41	2	39	4	27	13	42	3	42	3
Priniidae	*Apalis ruddi*				5	31	0	27	0	31	0	31	0	31	0	27	0	31	0	31	0
Priniidae	*Apalis sharpii*				5	26	4	12	3	23	5	38	3	32	5	18	7	38	6	39	3
Priniidae	*Apalis rufogularis*				5	41	5	27	9	42	5	55	4	52	6	35	11	56	6	61	4
Priniidae	*Apalis bamendae*			x	5	3	0x	0	2x	3	0x	3	0x	3	0x	0	2x	3	0x	3	0x
Priniidae	*Apalis goslingi*				5	36	5	25	8	39	6	58	5	56	5	37	9	64	7	68	6
Priniidae	*Apalis porphyrolaema*				5	33	5	29	11	38	5	38	1	35	5	31	12	38	7	38	1
Priniidae	*Apalis chapini*				5	43	0	34	0	43	0	43	0	43	0	39	0	43	0	43	0
Priniidae	*Apalis melanocephala*				5	37	0	33	0	37	0	37	0	37	0	34	1	37	0	37	0
Priniidae	*Apalis chirindensis*				5	64	0	60	0	64	0	64	0	64	1	63	6	64	0	64	0
Priniidae	*Apalis moreaui*	T		x	14	20	0x	19	0x	20	0x	20	0x	20	0x	20	0x	20	0x	20	0x
Priniidae	*Apalis cinerea*				5	34	7	26	9	34	7	45	4	43	9	31	11	47	7	46	5
Priniidae	*Apalis alticola*				5	66	0	50	1	64	0	68	0	67	0	53	3	66	0	68	0
Priniidae	*Apalis karamojae*	T			5	58	4	36	11	61	0	61	0	58	4	44	13	61	0	61	0
Priniidae	*Hypergerus atriceps*				5	24	6	16	6	30	7	46	4	37	7	23	14	48	12	49	8
Priniidae	*Eminia lepida*				5	43	3	30	12	47	2	48	1	45	3	34	13	49	2	48	1
Priniidae	*Camaroptera brachyura*				5	40	4	26	6	40	5	53	3	49	5	32	9	53	6	56	4
Priniidae	*Camaroptera superciliaris*				5	34	6	22	8	33	6	54	4	50	6	32	10	58	7	62	5
Priniidae	*Camaroptera chloronota*				5	33	6	21	8	32	7	53	3	48	6	32	9	57	8	61	4
Priniidae	*Calamonastes simplex*				5	40	4	13	24	46	1	46	1	43	4	17	27	46	1	46	1
Priniidae	*Calamonastes undosus*				5	44	1	35	2	44	1	48	1	47	3	38	7	47	2	48	2
Priniidae	*Calamonastes fasciolatus*				5	12	5	4	8	11	6	12	6	12	10	4	19	11	15	12	12
Priniidae	*Poliolais lopezi*				5	13	14	6	10	33	6	26	9	22	20	15	17	47	6	32	9
Priniidae	*Graueria vittata*				5	14	6	11	8	14	5	18	0	14	6	13	9	16	8	18	0
Priniidae	*Euryptila subcinnamomea*				5	2	8	1	9	2	10	1	9	2	10	1	11	3	13	2	13
Zosteropidae	*Speirops melanocephalus*	T		x	14	0	13x	0	13x	85	0x	29	0x	15	84x	0	84x	85	0x	100	0x
Zosteropidae	*Speirops lugubris*			x	14	0	100x	0	100x	100	0x	100	0x	0	100x	0	100x	100	0x	100	0x
Zosteropidae	*Speirops brunneus*	T		x	5	100	0x	100	0x	100	0x	100	0x	100	0x	100	0x	100	0x	100	0x
Zosteropidae	*Speirops leucophoeus*			x	5	0	0x	0	0x	0	0x	0	0x	0	0x	0	0x	0	0x	0	0x
Zosteropidae	*Zosterops senegalensis*				5	34	3	22	5	35	4	43	2	40	4	26	9	42	5	44	3
Zosteropidae	*Zosterops vaughani*			x	5	0	0x	0	0x	0	0x	0	0x	0	0x	0	0x	0	0x	0	0x
Zosteropidae	*Zosterops mayottensis*			x	13	100	0x	0	0x	100	0x	100	0x	100	0x	100	0x	100	0x	100	0x
Zosteropidae	*Zosterops poliogaster*			x	5	33	2x	31	2x	36	0x	36	0x	34	2x	34	2x	36	0x	36	0x
Zosteropidae	*Zosterops winifredae*	T		x	14	0	0x	0	0x	0	0x	0	0x	0	0x	0	0x	0	0x	0	0x
Zosteropidae	*Zosterops abyssinicus*				4,5	31	2	18	8	36	0	35	0	34	2	21	11	36	1	36	1
Zosteropidae	*Zosterops pallidus*		H		5	0	3	0	5	0	5	0	4	0	6	0	8	0	7	0	7
Zosteropidae	*Zosterops maderaspatanus*				6,13	49	0	27	5	50	0	50	0	50	0	37	5	50	0	50	0
Zosteropidae	*Zosterops mouroniensis*	T		x	13	0	0x	0	0x	0	0x	0	0x	0	0x	0	0x	0	0x	0	0x

Family	Species	IUCN	Ag	R	Range Source	2050 TG H	2050 TG C	2050 AM H	2050 AM C	2050 GO H	2050 GO C	2050 OS H	2050 OS C	2100 TG H	2100 TG C	2100 AM H	2100 AM C	2100 GO H	2100 GO C	2100 OS H	2100 OS C
						Percentage loss in geographic range															
Zosteropidae	*Zosterops ficedulinus*	T		x	5	0	⊦02x	0	⊦02x	100	0x	100	0x	0	⊦02x	0	⊦02x	100	0x	100	0x
Zosteropidae	*Zosterops griseovirescens*	T		x	5	0	⊦02x	0	⊦02x	100	0x	100	0x	0	⊦02x	0	⊦02x	100	0x	100	0x
Zosteropidae	*Zosterops borbonicus*		H	x	13	0	0x	0	0x	0	0x	0	0x	0	0x	0	0x	0	0x	0	0x
Zosteropidae	*Zosterops olivaceus*			x	13	49	0x	49	0x	49	0x	49	0x	49	0x	49	0x	49	0x	49	0x
Zosteropidae	*Zosterops chloronothos*	T		x	13	0	0x	0	0x	0	0x	0	0x	0	0x	0	0x	0	0x	0	0x
Zosteropidae	*Zosterops ceylonensis*			x	7	43	0x	39	0x	43	0x	43	0x	43	0x	40	0x	43	0x	43	0x
Zosteropidae	*Zosterops erythropleurus*				7	8	7	6	8	6	8	6	9	9	10	7	11	9	10	9	11
Zosteropidae	*Zosterops palpebrosus*				7	16	4	19	4	17	4	20	4	19	7	21	7	22	9	27	5
Zosteropidae	*Zosterops japonicus*		H		7,11	0	3	0	2	0	2	0	2	0	4	0	4	0	4	0	4
Zosteropidae	*Zosterops meyeni*		H		7	0	6	0	5	0	7	0	5	0	15	0	13	0	16	0	8
Zosteropidae	*Zosterops salvadorii*			x	7	0	0x	0	0x	0	0x	0	0x	0	0x	0	0x	0	0x	0	0x
Zosteropidae	*Zosterops atricapillus*				7,8	20	5	20	7	19	4	25	6	25	6	31	8	40	4	36	6
Zosteropidae	*Zosterops everetti*				7	13	7	20	8	16	8	26	8	18	8	25	8	33	10	32	8
Zosteropidae	*Zosterops nigrorum*				7	10	9	22	5	14	10	17	7	10	19	34	13	24	22	28	8
Zosteropidae	*Zosterops montanus*				7	9	8	10	9	7	9	8	9	11	12	14	12	18	12	15	11
Zosteropidae	*Zosterops flavus*				7	14	0	22	0	14	0	0	0	18	7	28	0	36	0	17	0
Zosteropidae	*Zosterops chloris*		H		7,8	0	7	0	8	0	8	0	10	0	10	0	22	0	22	0	21
Zosteropidae	*Zosterops citrinellus*		H		7,10	0	17	0	17	0	10	0	17	0	21	0	22	0	14	0	17
Zosteropidae	*Zosterops grayi*		H	x	13	0	0x	0	0x	0	0x	0	0x	0	0x	0	0x	0	0x	0	0x
Zosteropidae	*Zosterops uropygialis*		H	x	13	0	0x	0	0x	0	0x	0	0x	0	0x	0	0x	0	0x	0	0x
Zosteropidae	*Zosterops consobrinorum*				7	13	11x	39	13x	45	3x	0	24x	13	11x	39	14x	47	7x	15	39x
Zosteropidae	*Zosterops anomalus*			x	7	21	0x	32	0x	23	0x	30	0x	21	4x	35	11x	35	10x	30	1x
Zosteropidae	*Zosterops wallacei*				7	12	6	7	9	3	9	5	5	12	21x	7	23x	12	14x	15	5
Zosteropidae	*Zosterops atrifrons*				7	15	12	27	7	19	13	17	10	19	13	30	19	28	29	27	24
Zosteropidae	*Zosterops atriceps*				7	18	0x	32	0x	18	0x	22	0x	38	0x	32	0x	43	0x	41	0x
Zosteropidae	*Zosterops minor*				8	6	1	7	1	19	1	14	0	11	5	14	6	29	5	27	2
Zosteropidae	*Zosterops meeki*			x	8	46	0x	0	0x	0	0x	85	0x	61	0x	85	0x	0	0x	85	0x
Zosteropidae	*Zosterops hypoxanthus*				8	6	0	7	0	50	0	44	0	28	0	22	0	71	0	56	0
Zosteropidae	*Zosterops mysorensis*			x	8	0	5x	0	5x	0	5x	0	5x	0	5x	0	5x	0	5x	0	5x
Zosteropidae	*Zosterops fuscicapillus*				8	10	0	9	0	7	0	9	0	14	3	20	0	11	0	25	0
Zosteropidae	*Zosterops buruensis*		H	x	7	0	0x	0	0x	0	0x	0	0x	0	0x	0	0x	0	0x	0	0x
Zosteropidae	*Zosterops kuehni*			x	7	100	0x	79	0x	0	0x	21	0x	100	0x	79	0x	21	0x	100	0x
Zosteropidae	*Zosterops novaeguineae*		H		8	0	17	0	17	0	10	0	8	0	17	0	20	0	13	0	11
Zosteropidae	*Zosterops luteus*				10	0	24	1	11	0	22	2	9	0	24	1	21	0	28	2	12
Zosteropidae	*Zosterops griseotinctus*			x	8,9	7	0x	0	0x	22	0x	18	0x	14	0x	5	0x	22	0x	32	0x
Zosteropidae	*Zosterops rennellianus*			x	9	0	0x	0	0x	94	0x	94	0x	94	0x	0	0x	94	0x	94	0x
Zosteropidae	*Zosterops vellalavella*			x	9	0	0x	0	0x	100	0x	100	0x	0	0x	0	0x	100	0x	100	0x
Zosteropidae	*Zosterops splendidus*	T		x	9	85	0x	0	0x	15	0x	15	0x	85	0x	0	0x	15	0x	15	0x
Zosteropidae	*Zosterops luteirostris*	T		x	9	100	0x	0	0x	0	0x	0	0x	100	0x	0	0x	0	0x	0	0x
Zosteropidae	*Zosterops kulambangrae*			x	9	39	22x	30	0x	9	9x	40	0x	39	22x	38	0x	29	9x	40	0x
Zosteropidae	*Zosterops murphyi*			x	9	48	0x	0	0x	52	0x	95	0x	48	0x	0	0x	52	0x	95	0x
Zosteropidae	*Zosterops metcalfii*			x	9	8	1x	11	0x	17	0x	16	0x	20	10x	28	0x	46	0x	31	0x
Zosteropidae	*Zosterops rendovae*			x	9	13	3x	12	0x	16	0x	23	0x	25	12x	30	0x	53	0x	36	0x
Zosteropidae	*Zosterops stresemanni*			x	9	7	7x	0	7x	53	0x	0	7x	45	7x	0	7x	63	7x	14	7x

Family	Species	IUCN	Ag	R	Range Source	2050 TG H	2050 TG C	2050 AM H	2050 AM C	2050 GO H	2050 GO C	2050 OS H	2050 OS C	2100 TG H	2100 TG C	2100 AM H	2100 AM C	2100 GO H	2100 GO C	2100 OS H	2100 OS C
Zosteropidae	*Zosterops sanctaecrucis*			x	9	0	0x	25	0x	0	0x	0	0x	75	0x	25	0x	0	0x	0	0x
Zosteropidae	*Zosterops lateralis*				9,10,11,12,13	3	16	4	9	6	13	7	6	6	20	6	13	9	18	11	8
Zosteropidae	*Zosterops inornatus*			x	9	0	102x	0	102x	0	102x	58	42x	0	102x	0	102x	0	102x	58	42x
Zosteropidae	*Zosterops explorator*		H		11	0	4	0	4	0	0	0	0	0	4	0	4	0	0	0	0
Zosteropidae	*Zosterops flavifrons*		H	x	9	0	0x	0	0x	0	0x	0	0x	0	0x	0	0x	0	0x	0	0x
Zosteropidae	*Zosterops xanthochrous*			x	9	13	38x	43	15x	28	20x	21	22x	13	38x	53	17x	30	23x	24	22x
Zosteropidae	*Zosterops minutus*		H	x	9	0	102x	0	102x	0	102x	0	41x	0	102x	0	102x	0	102x	0	41x
Zosteropidae	*Zosterops samoensis*	T		x	11	0	0x	0	0x	0	0x	0	0x	55	0x	0	0x	0	0x	0	0x
Zosteropidae	*Tephrozosterops stalkeri*			x	7	13	0x	17	0x	22	0x	21	0x	32	0x	17	0x	26	0x	42	0x
Zosteropidae	*Madanga ruficollis*	T		x	7	8	0x	8	0x	8	0x	13	0x	16	0x	8	0x	21	0x	13	0x
Zosteropidae	*Lophozosterops javanicus*		H		7	0	0	0	0	0	0	0	0	0	0	0	0	0	0	0	0
Zosteropidae	*Lophozosterops squamicep*				7	14	13	26	9	19	13	16	11	17	14	29	22	28	31	24	27
Zosteropidae	*Lophozosterops goodfellow*				7	18	14	25	18	8	18	17	19	28	17	25	18	24	22	34	19
Zosteropidae	*Lophozosterops superciliari*			x	7	17	9x	11	13x	4	13x	7	7x	17	32x	11	36x	22	18x	22	7x
Zosteropidae	*Lophozosterops pinaiae*			x	7	13	0x	17	0x	22	0x	21	0x	32	0x	17	0x	26	0x	42	0x
Zosteropidae	*Lophozosterops dohertyi*			x	7	18	9x	12	12x	4	12x	7	7x	18	32x	12	36x	22	18x	22	7x
Zosteropidae	*Oculocincta squamifrons*				7	11	0	13	3	11	3	17	0	19	0	16	3	26	3	28	0
Zosteropidae	*Heleia crassirostris*			x	7	18	9x	12	13x	4	13x	7	7x	18	32x	12	36x	22	18x	22	7x
Zosteropidae	*Heleia muelleri*			x	7	0	41x	5	41x	20	27x	0	41x	0	41x	5	42x	20	27x	0	41x
Zosteropidae	*Chlorocharis emiliae*				7	9	0	12	1	6	2	10	1	15	0	14	1	17	2	18	1
Zosteropidae	*Woodfordia superciliosa*			x	9	0	0x	0	0x	71	0x	71	0x	71	0x	0	0x	71	0x	71	0x
Zosteropidae	*Woodfordia lacertosa*				9	0	0x	27	0x	0	0x	0	0x	73	0x	27	0x	0	0x	0	0x
Zosteropidae	*Hypocryptadius cinnamom*				7	19	14	25	18	8	18	17	19	28	17	25	18	24	22	33	19
Sylviidae	*Tesia castaneocoronata*				7	40	3	42	3	38	4	40	3	43	4	45	4	42	5	44	4
Sylviidae	*Tesia olivea*				7	29	3	31	3	28	3	27	4	33	4	33	3	30	4	31	5
Sylviidae	*Tesia cyaniventer*				7	29	4	31	3	29	3	31	2	31	5	34	6	33	8	35	5
Sylviidae	*Tesia superciliaris*				7	6	0	0	0	0	0	0	0	8	0	5	0	6	0	5	1
Sylviidae	*Tesia everetti*			x	7	15	7x	14	11x	4	11x	11	7x	15	27x	14	31x	18	16x	23	7x
Sylviidae	*Urosphena subulata*			x	7	13	30x	3	30x	10	23x	8	23x	13	30x	3	30x	10	23x	8	23x
Sylviidae	*Urosphena whiteheadi*				7	11	0	14	1	8	3	12	1	17	0	16	1	17	3	20	1
Sylviidae	*Urosphena squameiceps*				7	18	15	16	15	16	16	15	16	20	20	18	24	21	23	21	24
Sylviidae	*Cettia pallidipes*				7	40	7	43	6	39	7	42	6	44	8	47	7	44	9	47	7
Sylviidae	*Cettia canturians*				7	3	0	3	0	3	0	3	0	3	0	3	0	3	0	3	0
Sylviidae	*Cettia diphone*				7,11	14	14	12	14	13	15	12	15	16	19	14	23	17	22	17	22
Sylviidae	*Cettia seebohmi*				7	7	8	22	5	15	9	15	6	7	18	35	12	26	21	26	7
Sylviidae	*Cettia parens*			x	9	55	0x	24	0x	34	0x	83	0x	55	0x	24	0x	34	0x	100	0x
Sylviidae	*Cettia ruficapilla*				11	19	7	17	7	28	2	41	2	21	7	38	7	77	2	76	2
Sylviidae	*Cettia fortipes*				7	15	1	15	1	15	1	16	1	16	2	17	2	16	2	17	2
Sylviidae	*Cettia vulcania*				7	12	3	14	4	11	3	15	3	17	4	17	6	23	4	23	4
Sylviidae	*Cettia carolinae*			x	7	6	16x	6	16x	20	2x	65	23x	6	16x	6	16x	27	2x	65	23x
Sylviidae	*Cettia major*				7	43	2	45	1	44	2	45	2	47	3	48	3	47	3	48	3
Sylviidae	*Cettia flavolivacea*				7	27	3	28	3	26	4	28	3	30	4	30	4	29	5	30	4
Sylviidae	*Cettia acanthizoides*				7	19	1	20	1	19	1	21	0	20	2	21	1	21	1	21	1
Sylviidae	*Cettia brunnifrons*				7	49	3	50	3	50	3	52	2	51	5	55	6	54	6	54	5

Family	Species	IUCN	Ag	R	Range Source	Percentage loss in geographic range 2050 TG H	2050 TG C	2050 AM H	2050 AM C	2050 GO H	2050 GO C	2050 OS H	2050 OS C	2100 TG H	2100 TG C	2100 AM H	2100 AM C	2100 GO H	2100 GO C	2100 OS H	2100 OS C
Sylviidae	*Cettia cetti*				3,4,5,7	6	11	7	9	8	12	9	8	10	14	9	12	12	15	13	10
Sylviidae	*Bradypterus baboecala*				5	29	1	24	2	29	1	31	1	30	2	26	6	30	1	31	1
Sylviidae	*Bradypterus grandis*			x	5	40	1x	4	1x	27	1x	44	1x	40	1x	30	1x	68	1x	78	1x
Sylviidae	*Bradypterus carpalis*				5	24	2	13	3	26	0	26	0	24	2	21	5	26	0	26	0
Sylviidae	*Bradypterus graueri*	T		x	5	31	21x	18	19x	49	4x	58	0x	31	21x	34	28x	49	12x	58	0x
Sylviidae	*Bradypterus alfredi*				5	45	6	37	5	50	3	53	0	46	6	44	6	50	5	53	0
Sylviidae	*Bradypterus sylvaticus*	T			5	0	0	0	0	0	0	0	0	0	0	0	0	0	0	0	0
Sylviidae	*Bradypterus lopezi*				5	39	4	34	5	40	5	45	3	42	6	37	8	43	5	45	3
Sylviidae	*Bradypterus barratti*				5	17	0	14	0	17	0	17	0	17	0	15	1	17	0	17	0
Sylviidae	*Bradypterus cinnamomeus*				5	35	3	27	8	40	3	44	2	42	4	30	10	44	3	45	2
Sylviidae	*Bradypterus victorini*				5	0	4	0	4	0	4	0	4	0	4	0	5	0	5	0	5
Sylviidae	*Bradypterus thoracicus*				7	17	5	18	5	19	5	19	4	18	8	20	10	23	8	23	7
Sylviidae	*Bradypterus major*				7	40	11	40	10	44	10	43	8	42	17	46	16	51	14	50	14
Sylviidae	*Bradypterus tacsanowskius*				7	16	2	17	2	16	2	16	2	16	3	17	4	17	4	17	4
Sylviidae	*Bradypterus luteoventris*				7	14	4	15	3	14	4	15	4	15	5	17	5	17	6	17	5
Sylviidae	*Bradypterus seebohmi*				7	6	8	8	6	7	8	7	10	7	11	8	10	11	11	11	13
Sylviidae	*Bradypterus palliseri*				7	49	0	43	0	43	7	54	0	50	0	45	0	47	7	54	0
Sylviidae	*Bradypterus caudatus*				7	19	2	25	2x	10	3	17	3	19	9x	25	9x	25	9x	22	3x
Sylviidae	*Bradypterus accentor*			x	7	0	0x	19	0x	6	0x	41	0x	0	0x	19	0x	24	0x	41	0x
Sylviidae	*Bradypterus castaneus*				7	14	12	24	8	19	11	16	10	18	12	27	18	28	25	25	23
Sylviidae	*Dromaeocercus brunneus*			x	6	86	0x	64	0x	86	0x	86	0x	86	0x	86	0x	86	0x	86	0x
Sylviidae	*Dromaeocercus seebohmi*			x	6	72	0x	70	0x	72	0x	72	0x	72	0x	72	0x	72	0x	72	0x
Sylviidae	*Bathmocercus cerviniventri*			x	5	70	0x	0	30x	70	0x	30	0x	70	0x	0	30x	70	0x	70	0x
Sylviidae	*Bathmocercus rufus*				5	32	6	25	8	35	7	51	6	49	6	35	8	52	8	60	6
Sylviidae	*Bathmocercus winifredae*	T			5	47	0	34	0	47	0	47	0	47	0	47	0	47	0	47	0
Sylviidae	*Nesillas longicaudata*			x	13	0	0x	0	0x	0	0x	0	0x	0	0x	0	0x	0	0x	0	0x
Sylviidae	*Nesillas typica*				6,13	66	1	34	9	67	1	67	1	67	1	50	9	67	1	67	1
Sylviidae	*Nesillas moheliensis*			x	13	100	0x	100	0x	100	0x	100	0x	100	0x	100	0x	100	0x	100	0x
Sylviidae	*Nesillas brevicaudata*			x	13	0	0x	0	0x	0	0x	0	0x	0	0x	0	0x	0	0x	0	0x
Sylviidae	*Nesillas mariae*			x	13	100	0x	100	0x	100	0x	100	0x	100	0x	100	0x	100	0x	100	0x
Sylviidae	*Thamnornis chloropetoides*				6	24	0	3	2	24	0	24	0	24	0	12	2	24	0	24	0
Sylviidae	*Melocichla mentalis*				5	34	2	22	3	37	2	42	1	40	2	28	7	44	2	44	2
Sylviidae	*Achaetops pycnopygius*				14	14	11	6	13	13	10	12	11	14	13	6	17	13	12	12	13
Sylviidae	*Sphenoeacus afer*				5	4	0	4	1	4	2	4	1	4	3	4	4	4	4	4	4
Sylviidae	*Locustella lanceolata*				7	3	17	2	17	3	19	2	16	4	20	3	24	4	27	5	24
Sylviidae	*Locustella naevia*		H		3,4,5,7	0	21	0	19	0	24	0	18	0	25	0	23	0	27	0	21
Sylviidae	*Locustella certhiola*				4,7	6	11	6	12	7	13	6	12	7	14	7	17	9	20	9	18
Sylviidae	*Locustella ochotensis*				7	1	23	0	24	1	26	1	25	2	31	1	37	1	37	3	36
Sylviidae	*Locustella pleskei*	T			7	25	7	24	6	26	5	23	6	25	10	24	14	27	12	24	12
Sylviidae	*Locustella fluviatilis*				3,4,5,7	7	27	5	25	10	31	7	23	11	30	9	27	16	34	18	26
Sylviidae	*Locustella luscinioides*				3,4,5,7	4	13	3	9	6	15	4	9	8	14	5	11	9	16	9	10
Sylviidae	*Locustella fasciolata*				7,8	8	6	6	7	7	7	7	7	8	8	7	9	8	9	9	9
Sylviidae	*Locustella pryeri*	T			7	1	1	0	3	5	1	2	3	1	5	0	10	5	3	2	8
Sylviidae	*Acrocephalus melanopogo*				3,4,5,7	3	6	2	6	3	8	3	7	5	8	4	8	5	10	7	9

Family	Species	IUCN	Ag	R	Range Source	Percentage loss in geographic range 2050 TG H	C	AM H	C	GO H	C	OS H	C	2100 TG H	C	AM H	C	GO H	C	OS H	C
Sylviidae	*Acrocephalus paludicola*	T			3,4,5	3	12	2	10	8	15	5	7	10	13	3	10	11	15	8	8
Sylviidae	*Acrocephalus schoenobae*				3,4,5,7	4	19	4	17	6	21	5	15	8	22	6	19	10	24	10	17
Sylviidae	*Acrocephalus sorghophilus*	T	H		7	0	0	0	0	0	0	0	0	0	0	0	0	0	0	0	0
Sylviidae	*Acrocephalus bistrigiceps*		H		7	0	3	0	3	0	3	0	3	0	4	0	5	0	5	0	5
Sylviidae	*Acrocephalus agricola*				3,4,7	3	12	3	12	4	14	4	12	5	15	5	17	7	21	10	17
Sylviidae	*Acrocephalus tangorum*	T			14	16	2	15	3	14	2	14	3	17	3	16	3	16	2	16	3
Sylviidae	*Acrocephalus concinens*		H		7	0	0	0	0	0	0	0	0	0	0	0	0	0	0	0	0
Sylviidae	*Acrocephalus scirpaceus*		H		3,4,5,7	0	20	0	16	0	20	0	15	0	23	0	20	0	23	0	17
Sylviidae	*Acrocephalus baeticatus*				4,5	24	3	17	5	24	4	25	3	24	5	19	10	25	6	26	6
Sylviidae	*Acrocephalus dumetorum*		H		3,4,7	0	15	0	15	0	19	0	14	0	18	0	17	0	21	0	17
Sylviidae	*Acrocephalus palustris*		H		3,4,5	0	15	0	13	0	17	0	12	0	18	0	15	0	19	0	13
Sylviidae	*Acrocephalus arundinaceu*				3,4,5,7,8	7	12	6	10	8	14	7	10	9	14	8	13	11	16	11	11
Sylviidae	*Acrocephalus stentoreus*				3,4,5,7,8,10	10	6	11	4	11	5	13	3	12	7	13	7	13	9	18	4
Sylviidae	*Acrocephalus griseldis*	T			3,4,5	10	2	5	2	8	2	8	0	14	2	8	2	16	3	18	2
Sylviidae	*Acrocephalus australis*				8,9	6	5	5	5	24	3	20	3	14	6	12	7	36	5	33	4
Sylviidae	*Acrocephalus familiaris*	T		x	11	100	0x	0	0x	0	0x	0	0x	100	0x	0	0x	100	0x	100	0x
Sylviidae	*Acrocephalus rufescens*				5	31	4	22	7	35	3	40	1	35	5	27	11	40	6	43	2
Sylviidae	*Acrocephalus brevipennis*	T	H	x	13	0	5x	0	5x	0	0x	0	0x	0	5x	0	5x	0	0x	0	0x
Sylviidae	*Acrocephalus gracilirostris*				5	26	2	20	4	27	2	27	2	27	3	22	7	28	3	28	3
Sylviidae	*Acrocephalus newtoni*				6	49	0	28	5	49	0	49	0	49	0	37	5	49	0	49	0
Sylviidae	*Acrocephalus aedon*				7	13	8	13	9	13	10	13	9	14	10	13	10	14	11	15	11
Sylviidae	*Hippolais caligata*		H		3,4,7	0	12	0	12	0	15	0	12	0	15	0	16	0	19	0	16
Sylviidae	*Hippolais rama*				4	0	15	6	15	0	15	0	15	0	15	6	15	0	15	5	15
Sylviidae	*Hippolais pallida*				3,4,5	11	5	11	5	13	6	16	4	17	6	15	6	17	7	19	5
Sylviidae	*Hippolais languida*		H		3,4,5,7	0	3	0	3	0	4	0	3	0	4	0	5	0	6	0	5
Sylviidae	*Hippolais olivetorum*		H		3,4,5	0	20	0	16	0	20	0	13	0	24	0	20	0	23	0	15
Sylviidae	*Hippolais polyglotta*		H		3,4,5	0	16	0	9	0	13	0	7	0	23	0	18	0	21	0	11
Sylviidae	*Hippolais icterina*		H		3,4,5,7	0	24	0	22	0	27	0	19	0	27	0	24	0	30	0	22
Sylviidae	*Chloropeta natalensis*				5	36	2	23	5	34	2	40	1	39	2	27	8	40	2	42	2
Sylviidae	*Chloropeta similis*				5	37	3	26	8	39	2	40	0	38	4	32	9	39	3	40	0
Sylviidae	*Chloropeta gracilirostris*	T			5	27	9	19	5	35	1	36	0	27	9	27	10	35	2	36	0
Sylviidae	*Orthotomus metopias*				14	41	0	36	0	41	0	41	0	41	0	39	0	41	0	41	0
Sylviidae	*Orthotomus cuculatus*				7	19	7	23	6	20	8	23	7	23	9	26	9	27	10	29	10
Sylviidae	*Orthotomus heterolaemus*				7	16	9	24	12	8	13	15	12	16	12	24	12	21	15	22	12
Sylviidae	*Orthotomus sutorius*		H		7	0	4	0	4	0	5	0	4	0	7	0	8	0	10	0	6
Sylviidae	*Orthotomus atrogularis*				7	17	7	21	7	18	8	21	7	21	9	25	10	27	12	29	10
Sylviidae	*Orthotomus castaneiceps*				7	9	8	21	5	15	10	18	7	9	17	36	11	26	22	28	8
Sylviidae	*Orthotomus frontalis*				7	20	15	27	16	12	15	26	16	31	17	27	16	26	21	40	17
Sylviidae	*Orthotomus derbianus*				7	10	11	21	8	16	11	19	8	10	20	32	17	28	25	29	13
Sylviidae	*Orthotomus sericeus*				7	12	4	16	4	15	4	18	4	19	5	22	5	28	5	27	5
Sylviidae	*Orthotomus ruficeps*				7	11	3	15	3	15	3	16	3	18	4	21	5	25	5	25	4
Sylviidae	*Orthotomus sepium*				7	2	0	0	0	0	0	0	0	4	0	1	6	3	0	2	0
Sylviidae	*Orthotomus samarensis*			x	7	27	11x	27	10x	27	3x	50	11x	44	12x	27	10x	36	12x	58	12x
Sylviidae	*Orthotomus nigriceps*				7	17	15	27	17	8	17	19	18	27	18	27	17	25	23	35	18

Family	Species	IUCN	Ag	R	Range Source	Percentage loss in geographic range 2050 TG H	C	AM H	C	GO H	C	OS H	C	2100 TG H	C	AM H	C	GO H	C	OS H	C
Sylviidae	*Orthotomus cinereiceps*				7	18	14	26	18	8	18	17	18	28	17	26	18	24	22	33	18
Sylviidae	*Eremomela icteropygialis*				5	20	4	13	6	21	4	23	3	22	5	15	11	23	7	23	6
Sylviidae	*Eremomela salvadorii*				5	49	7	29	9	50	6	62	4	60	8	38	11	67	7	69	4
Sylviidae	*Eremomela flavicrissalis*				5	38	6	10	31	45	2	46	2	41	6	11	35	45	5	46	3
Sylviidae	*Eremomela pusilla*				5	9	2	5	2	10	2	13	1	12	3	7	4	15	3	15	2
Sylviidae	*Eremomela canescens*				5	13	1	8	4	14	1	15	1	15	1	10	5	16	1	16	1
Sylviidae	*Eremomela scotops*				5	47	3	32	4	45	3	53	1	51	3	36	8	51	3	54	2
Sylviidae	*Eremomela gregalis*				5	2	9	1	11	2	12	1	11	2	12	1	14	2	16	2	15
Sylviidae	*Eremomela badiceps*				5	38	6	26	8	36	7	57	4	52	6	35	10	60	7	65	4
Sylviidae	*Eremomela turneri*	T		x	5	48	0x	54	0x	6	0x	50	0x	49	0x	62	0x	70	0x	59	0x
Sylviidae	*Eremomela atricollis*				5	55	3	39	3	56	2	63	2	61	4	41	6	60	3	64	2
Sylviidae	*Eremomela usticollis*				5	14	2	10	3	13	2	13	1	14	4	10	10	13	4	13	3
Sylviidae	*Randia pseudozosterops*				6	75	0	45	4	78	0	78	0	78	0	71	4	78	0	78	0
Sylviidae	*Newtonia amphichroa*				6	75	0	45	4	78	0	78	0	78	0	71	4	78	0	78	0
Sylviidae	*Newtonia brunneicauda*				6	52	0	31	5	52	0	52	0	52	0	41	5	52	0	52	0
Sylviidae	*Newtonia archboldi*				6	18	0	3	1	18	0	18	0	18	0	10	1	18	0	18	0
Sylviidae	*Newtonia fanovanae*	T		x	6	35	0x	35	0x	35	0x	35	0x	35	0x	35	0x	35	0x	35	0x
Sylviidae	*Sylvietta virens*				5	35	7	23	9	33	8	55	4	49	8	33	12	57	10	62	5
Sylviidae	*Sylvietta denti*				5	32	7	19	8	26	8	53	3	46	8	30	10	51	9	62	4
Sylviidae	*Sylvietta leucophrys*				5	26	5	21	9	32	5	33	0	28	5	23	10	32	7	33	0
Sylviidae	*Sylvietta brachyura*				5	26	4	12	8	29	4	37	3	34	5	17	12	38	7	39	4
Sylviidae	*Sylvietta philippae*				5	18	0	4	14	22	0	22	0	21	0	7	15	22	2	22	0
Sylviidae	*Sylvietta ruficapilla*				5	45	4	31	5	42	4	54	2	52	5	36	10	52	5	56	3
Sylviidae	*Sylvietta whytii*				5	42	1	32	5	43	1	43	1	42	2	36	7	44	1	43	1
Sylviidae	*Sylvietta isabellina*				5	45	7	11	30	55	2	56	1	50	7	15	36	56	3	56	2
Sylviidae	*Sylvietta rufescens*				5	21	3	16	4	20	4	21	3	21	5	16	10	21	6	21	5
Sylviidae	*Hemitesia neumanni*				5	23	3	20	6	25	7	40	0	38	3	24	13	35	8	42	0
Sylviidae	*Macrosphenus kempi*				5	21	8	13	8	25	5	31	5	26	10	18	13	35	7	35	6
Sylviidae	*Macrosphenus flavicans*				5	36	6	21	8	36	7	58	5	53	7	33	10	64	7	66	5
Sylviidae	*Macrosphenus concolor*				5	33	6	21	9	31	8	53	5	49	7	31	11	57	10	61	6
Sylviidae	*Macrosphenus pulitzeri*	T		x	5	17	0x	16	0x	17	0x	17	0x	17	0x	16	0x	17	0x	17	0x
Sylviidae	*Macrosphenus kretschmeri*				5	47	0	37	0	47	0	47	0	47	0	44	0	47	0	47	0
Sylviidae	*Amaurocichla bocagii*	T		x	5	0⊦02x		0⊦02x		100	0x	100	0x	0⊦02x		0⊦02x		100	0x	100	0x
Sylviidae	*Hylia prasina*				5	32	6	20	8	31	7	51	4	46	7	29	10	53	9	57	5
Sylviidae	*Leptopoecile sophiae*				7	32	8	35	9	36	9	37	8	34	17	38	24	46	18	45	16
Sylviidae	*Leptopoecile elegans*				7	36	5	38	4	37	5	38	5	37	8	41	9	41	10	42	9
Sylviidae	*Phylloscopus laetus*				5	31	5	26	7	33	4	36	0	33	5	31	7	36	5	36	0
Sylviidae	*Phylloscopus laurae*				5	54	0	50	0	56	0	60	0	59	1	52	2	60	0	60	0
Sylviidae	*Phylloscopus ruficapillus*				5	28	0	24	0	28	0	28	0	28	1	25	2	28	0	28	0
Sylviidae	*Phylloscopus herberti*				5	33	12	23	7	45	6	51	6	47	18	26	14	57	6	58	6
Sylviidae	*Phylloscopus budongoensi*				5	40	2	26	9	33	8	49	3	51	2	33	9	38	8	52	3
Sylviidae	*Phylloscopus umbrovirens*				4,5	32	2	25	8	34	3	36	0	35	2	28	8	34	5	36	1
Sylviidae	*Phylloscopus trochilus*				3,4,5,7	4	18	3	16	5	19	4	15	6	21	5	20	9	23	9	19
Sylviidae	*Phylloscopus collybita*				3,4,5,7	4	16	4	14	5	17	5	13	7	19	6	18	10	21	10	17

Family	Species	IUCN	Ag	R	Range Source	Percentage loss in geographic range 2050 TG H	TG C	AM H	AM C	GO H	GO C	OS H	OS C	2100 TG H	TG C	AM H	AM C	GO H	GO C	OS H	OS C
Sylviidae	*Phylloscopus sindianus*				4	36	1	40	1	45	0	52	0	57	2	57	2	60	2	61	1
Sylviidae	*Phylloscopus neglectus*				4,7	31	2	31	1	33	1	30	1	31	3	35	1	36	1	37	1
Sylviidae	*Phylloscopus bonelli*				3,4,5	6	20	8	15	12	17	14	12	12	28	12	25	24	27	19	18
Sylviidae	*Phylloscopus orientalis*				3,4	18	12	20	8	14	13	23	8	24	25	26	16	26	22	27	13
Sylviidae	*Phylloscopus sibilatrix*				3,4,5,7	8	29	8	26	11	30	10	25	12	35	12	32	20	36	21	30
Sylviidae	*Phylloscopus fuscatus*				4,7	9	8	8	8	9	9	9	8	9	11	9	15	11	15	11	14
Sylviidae	*Phylloscopus fuligiventer*				7	38	15	47	9	47	9	47	8	42	21	53	26	49	31	51	25
Sylviidae	*Phylloscopus affinis*				7	27	11	29	11	29	11	30	10	29	19	33	23	36	22	36	19
Sylviidae	*Phylloscopus subaffinis*				7	13	1	14	2	14	2	15	1	14	3	14	5	17	3	17	2
Sylviidae	*Phylloscopus griseolus*				4,7	4	16	5	16	5	16	5	15	6	20	6	23	9	25	11	24
Sylviidae	*Phylloscopus armandii*				7	12	0	12	1	12	1	12	0	13	1	13	1	13	1	13	1
Sylviidae	*Phylloscopus schwarzi*				4,7	10	10	8	11	8	12	8	11	10	13	9	13	10	14	11	13
Sylviidae	*Phylloscopus pulcher*				7	29	2	29	1	29	1	30	1	29	3	31	3	31	3	31	2
Sylviidae	*Phylloscopus maculipennis*				7	34	7	37	5	36	7	37	7	36	9	39	7	40	8	41	8
Sylviidae	*Phylloscopus proregulus*				4,7	7	5	7	5	7	5	7	5	8	6	7	7	8	7	8	7
Sylviidae	*Phylloscopus kansuensis*				14	23	9	27	11	28	12	27	11	28	11	30	17	36	14	36	14
Sylviidae	*Phylloscopus chloronotus*				7	45	4	47	3	47	3	48	3	47	7	51	8	52	7	52	6
Sylviidae	*Phylloscopus sichuanensis*				14	7	0	7	0	7	1	7	0	7	0	7	0	7	1	7	0
Sylviidae	*Phylloscopus subviridis*			x	4,7	44	0x	42	0x	45	0x	46	0x	44	0x	45	0x	50	0x	50	0x
Sylviidae	*Phylloscopus inornatus*				4,7	3	12	2	12	3	12	3	12	4	14	3	17	4	18	5	17
Sylviidae	*Phylloscopus humei*				4	52	11	48	11	54	8	57	7	59	15	59	15	68	12	69	11
Sylviidae	*Phylloscopus borealis*				2,3,4,7,8	3	14	3	14	3	14	3	14	4	17	3	19	4	21	5	19
Sylviidae	*Phylloscopus trochiloides*				3,4,7	10	10	10	10	11	11	11	10	12	12	12	13	13	14	14	12
Sylviidae	*Phylloscopus nitidus*				4	12	1	13	0	12	1	16	1	19	1	16	1	20	2	19	3
Sylviidae	*Phylloscopus tenellipes*				7	8	18	6	17	7	20	5	18	9	26	10	31	9	32	19	29
Sylviidae	*Phylloscopus borealoides*				7	17	11	14	13	14	13	13	13	18	16	16	18	18	17	19	18
Sylviidae	*Phylloscopus magnirostris*				7	47	3	49	3	48	3	50	2	49	5	52	5	52	5	53	4
Sylviidae	*Phylloscopus tytleri*				7	57	5	58	0	63	0	56	3	58	5	65	0	66	0	64	3
Sylviidae	*Phylloscopus occipitalis*				4,7	66	5	67	3	67	1	72	1	68	8	73	5	76	3	76	3
Sylviidae	*Phylloscopus coronatus*				7	24	6	23	5	24	6	23	6	24	8	24	9	25	9	25	9
Sylviidae	*Phylloscopus ijimae*	T		x	13	0	0x	0	0x	0	0x	0	0x	0	0x	0	54x	0	54x	0	54x
Sylviidae	*Phylloscopus reguloides*				7	18	5	20	4	18	5	20	5	20	6	22	6	22	7	23	6
Sylviidae	*Phylloscopus hainanus*	T		x	14	0	0x	0	0x	0	0x	0	0x	0	0x	0	0x	0	0x	0	0x
Sylviidae	*Phylloscopus emeiensis*			x	14	10	0x	10	0x	10	0x	10	0x	10	0x	10	0x	10	0x	10	0x
Sylviidae	*Phylloscopus davisoni*				7	13	6	16	5	13	7	14	6	14	8	17	7	17	9	17	8
Sylviidae	*Phylloscopus cantator*				7	43	8	45	7	41	9	41	9	49	9	50	8	46	11	47	10
Sylviidae	*Phylloscopus ricketti*				7	7	0	7	0	7	0	7	0	7	0	7	0	7	0	7	0
Sylviidae	*Phylloscopus olivaceus*				7	21	13	27	16	13	14	27	16	32	16	27	16	27	20	40	16
Sylviidae	*Phylloscopus cebuensis*				7	10	9	22	5	14	10	17	7	10	19	34	13	24	22	28	9
Sylviidae	*Phylloscopus trivirgatus*				7,8	11	4	14	4	10	5	16	4	16	5	17	5	21	7	23	5
Sylviidae	*Phylloscopus sarasinorum*				7	14	14	27	9	18	15	17	11	17	15	31	23	29	33	26	28
Sylviidae	*Phylloscopus presbytes*			x	7	15	18x	18	23x	9	19x	4	19x	15	18x	18	23x	10	19x	16	19x
Sylviidae	*Phylloscopus poliocephalu*				8,9	7	1	6	1	25	1	14	1	13	5	13	7	38	6	32	3
Sylviidae	*Phylloscopus makirensis*			x	9	48	0x	34	0x	40	0x	77	0x	48	0x	34	0x	40	0x	99	0x

Family	Species	IUCN	Ag	R	Range Source	Percentage loss in geographic range															
						2050								2100							
						TG		AM		GO		OS		TG		AM		GO		OS	
						H	C	H	C	H	C	H	C	H	C	H	C	H	C	H	C
Sylviidae	*Phylloscopus amoenus*	T		x	9	14	0x	0	0x	86	0x	100	0x	14	0x	0	0x	86	0x	100	0x
Sylviidae	*Seicercus burkii*				7	19	4	20	3	19	4	20	4	20	5	22	4	22	5	23	5
Sylviidae	*Seicercus xanthoschistos*				7	25	3	28	2	28	2	28	1	27	4	30	2	31	3	32	2
Sylviidae	*Seicercus affinis*				7	29	3	32	2	32	3	31	2	33	5	34	4	35	5	35	4
Sylviidae	*Seicercus poliogenys*				7	39	3	42	3	40	4	43	2	44	5	45	5	44	5	48	4
Sylviidae	*Seicercus castaniceps*				7	16	4	17	4	17	4	19	4	19	5	19	5	21	6	22	5
Sylviidae	*Seicercus montis*				7	14	4	15	5	14	4	20	4	19	4	19	5	29	4	28	4
Sylviidae	*Seicercus grammiceps*				7	21	5	20	6	19	4	22	6	27	6	28	7	40	4	34	6
Sylviidae	*Tickellia hodgsoni*				7	44	5	47	5	46	6	47	4	50	7	50	5	51	7	54	6
Sylviidae	*Abroscopus albogularis*				7	15	2	16	2	15	3	16	3	16	3	17	3	17	4	18	3
Sylviidae	*Abroscopus schisticeps*				7	36	8	39	6	37	7	37	7	40	9	42	9	41	11	42	10
Sylviidae	*Abroscopus superciliaris*				7	19	6	23	5	20	6	22	6	23	8	27	7	27	9	29	8
Sylviidae	*Hyliota flavigaster*				5	41	3	27	5	44	4	52	3	49	4	32	9	52	5	54	4
Sylviidae	*Hyliota australis*				5	56	0	44	1	57	0	58	0	58	2	45	5	58	1	58	1
Sylviidae	*Hyliota violacea*				5	30	4	16	5	24	5	52	3	46	4	25	6	51	6	61	3
Sylviidae	*Megalurus timoriensis*		H		7,8,10	0	12	0	9	0	10	0	7	0	16	0	13	0	15	0	10
Sylviidae	*Megalurus palustris*				7	19	4	21	4	19	4	22	4	21	6	23	6	23	8	28	5
Sylviidae	*Megalurus albolimbatus*	T		x	8	0	13x	0	13x	0	13x	41	13x	32	13x	9	13x	38	13x	41	13x
Sylviidae	*Megalurus gramineus*				8,10	1	10	1	6	1	8	2	3	3	13	1	7	3	12	3	4
Sylviidae	*Megalurus punctatus*				12,13	7	18	7	13	20	14	19	9	9	20	13	16	20	21	24	12
Sylviidae	*Cincloramphus cruralis*		H		10	0	9	0	6	0	8	0	5	0	11	0	9	0	11	0	6
Sylviidae	*Cincloramphus mathewsi*				10	1	13	1	9	2	11	2	7	2	16	1	13	2	16	3	8
Sylviidae	*Eremiornis carteri*				10	0	16	0	16	0	15	0	15	0	17	0	18	0	18	0	18
Sylviidae	*Buettikoferella bivittata*			x	13	0	41x	6	41x	20	26x	0	41x	0	41x	6	41x	20	26x	0	41x
Sylviidae	*Megalurulus mariei*			x	9	20	31x	37	10x	32	22x	33	10x	20	31x	57	12x	39	24x	38	10x
Sylviidae	*Megalurulus grosvenori*			x	8	0	0x	0	0x	66	0x	18	0x	18	0x	16	0x	82	0x	46	0x
Sylviidae	*Megalurulus llaneae*			x	9	12	0x	12	0x	4	0x	6	0x	16	0x	12	0x	38	0x	40	0x
Sylviidae	*Megalurulus whitneyi*			x	9	8	0x	20	0x	18	0x	20	0x	26	0x	20	0x	46	0x	73	0x
Sylviidae	*Megalurulus rubiginosus*				8	7	0	8	0	51	0	46	0	24	0	27	0	72	0x	59	0
Sylviidae	*Trichocichla rufa*				11	26	4	21	4	30	0	43	0	27	4	50	4	86	0x	85	0x
Sylviidae	*Chaetornis striatus*	T	H		7	0	3	0	3	0	3	0	2	0	5	0	7	0	9	0	3
Sylviidae	*Graminicola bengalensis*				7	14	4	18	2	15	2	18	2	17	4	20	3	18	4	19	3
Sylviidae	*Schoenicola brevirostris*				5	41	2	31	4	41	2	45	1	44	2	35	6	45	2	46	1
Sylviidae	*Schoenicola platyura*	T			7	6	0	7	1	10	1	11	1	8	0	8	1	10	1	16	1
Sylviidae	*Garrulax cinereifrons*	T		x	7	45	0x	42	0x	45	0x	45	0x	46	0x	44	0x	46	0x	46	0x
Sylviidae	*Garrulax palliatus*				7	20	3	25	4	22	4	26	5	27	4	32	5	40	4	39	5
Sylviidae	*Garrulax rufifrons*			x	7	0	0x	0	0x	0	0x	0	0x	1	0x	0	0x	0	0x	0	0x
Sylviidae	*Garrulax perspicillatus*		H		7	0	1	0	1	0	1	0	1	0	1	0	1	0	1	0	1
Sylviidae	*Garrulax albogularis*				7	29	2	30	1	29	2	30	1	29	3	31	3	31	3	32	3
Sylviidae	*Garrulax leucolophus*				7	30	8	32	8	30	9	33	9	34	11	36	12	38	14	41	12
Sylviidae	*Garrulax monileger*				7	19	5	21	5	19	6	20	5	21	7	22	8	22	9	24	8
Sylviidae	*Garrulax pectoralis*				7,11	14	3	15	3	14	3	14	3	16	4	16	4	15	4	17	4
Sylviidae	*Garrulax lugubris*				7	22	6	22	6	22	5	28	6	28	6	29	7	43	5	39	6
Sylviidae	*Garrulax calvus*				7	10	0	23	3	18	3	27	0	17	0	29	3	35	3	41	0

Family	Species	IUCN	Ag	R	Range Source	Percentage loss in geographic range															
						2050								2100							
						TG		AM		GO		OS		TG		AM		GO		OS	
						H	C	H	C	H	C	H	C	H	C	H	C	H	C	H	C
Sylviidae	*Garrulax striatus*				7	56	5	58	4	55	5	60	3	62	8	63	7	63	7	66	6
Sylviidae	*Garrulax strepitans*				7	24	10	27	10	22	11	22	12	26	12	29	13	26	14	27	15
Sylviidae	*Garrulax milleti*				7	8	11	10	8	4	11	4	11	8	14	10	28	10	32	11	32
Sylviidae	*Garrulax maesi*				7	14	3	15	2	14	3	15	3	15	4	15	3	16	4	17	4
Sylviidae	*Garrulax ruficollis*				7	49	8	51	8	47	11	52	7	58	11	57	11	54	13	59	9
Sylviidae	*Garrulax nuchalis*				7	43	15	44	10	38	15	33	18	53	21	52	17	50	20	45	25
Sylviidae	*Garrulax chinensis*				7	12	7	16	6	13	7	15	7	14	10	17	9	17	12	19	9
Sylviidae	*Garrulax vassali*				7	7	11	9	10	13	12	14	9	10	17	11	20	20	21	21	16
Sylviidae	*Garrulax galbanus*				7	34	3	32	5	30	7	36	5	37	5	37	7	35	10	41	6
Sylviidae	*Garrulax delesserti*		H		7	0	1	0	2	0	2	0	0	0	2	0	2	0	4	0	0
Sylviidae	*Garrulax gularis*		H		7	0	11	0	10	0	14	0	14	0	15	0	16	0	18	0	17
Sylviidae	*Garrulax davidi*				7	20	5	21	7	22	8	22	6	21	11	23	15	28	11	28	10
Sylviidae	*Garrulax sukatschewi*	T			7	13	0	12	3	14	1	13	0	15	0	12	3	14	1	15	0
Sylviidae	*Garrulax cineraceus*				7	12	2	12	2	11	3	12	2	13	3	13	3	12	3	13	3
Sylviidae	*Garrulax rufogularis*		H		7	0	7	0	6	0	9	0	6	0	10	0	9	0	11	0	8
Sylviidae	*Garrulax lunulatus*				7	16	0	16	0	16	1	16	1	16	1	18	1	16	1	16	2
Sylviidae	*Garrulax bieti*	T			7	27	0	24	0	24	0	24	0	27	0	24	0	27	0	24	0
Sylviidae	*Garrulax maximus*				7	24	1	24	1	24	1	24	1	24	2	26	4	26	3	25	3
Sylviidae	*Garrulax ocellatus*				7	32	2	33	2	32	2	34	2	34	3	36	3	34	3	35	2
Sylviidae	*Garrulax caerulatus*				7,11	49	8	52	7	50	8	51	7	53	11	55	11	54	11	56	10
Sylviidae	*Garrulax poecilorhynchus*				7	11	0	11	0	11	0	10	1	11	0	11	1	11	0	10	1
Sylviidae	*Garrulax mitratus*				7	16	3	19	4	17	3	22	4	22	4	24	5	33	3	33	4
Sylviidae	*Garrulax merulinus*				7	32	11	35	10	30	14	31	13	37	14	38	13	36	17	39	15
Sylviidae	*Garrulax canorus*		H		7,11	0	2	0	2	0	2	0	2	0	3	0	2	0	3	0	3
Sylviidae	*Garrulax sannio*		H		7	0	2	0	2	0	2	0	2	0	3	0	2	0	3	0	3
Sylviidae	*Garrulax cachinnans*	T		x	7	2	0x	50	0x	11	0x	43	0x	13	0x	57	0x	25	0x	80	0x
Sylviidae	*Garrulax jerdoni*				7	33	0	54	0	44	0	60	0	38	1	68	0	56	0	83	0
Sylviidae	*Garrulax lineatus*				7	39	13	42	11	43	10	44	9	42	20	47	21	48	22	52	19
Sylviidae	*Garrulax virgatus*				7	53	5	49	7	39	12	51	5	61	7	58	11	52	18	63	8
Sylviidae	*Garrulax austeni*				7	61	3	60	7	50	10	56	6	68	4	62	8	59	13	66	8
Sylviidae	*Garrulax squamatus*				7	40	9	42	8	38	11	39	11	46	12	45	11	44	13	45	13
Sylviidae	*Garrulax subunicolor*				7	39	5	44	5	43	6	45	3	44	9	46	11	46	13	49	9
Sylviidae	*Garrulax elliotii*				7	16	1	16	1	16	1	16	1	16	1	17	2	17	2	17	2
Sylviidae	*Garrulax variegatus*				7	64	5	64	4	67	1	72	1	66	7	70	5	75	3	74	2
Sylviidae	*Garrulax henrici*				7	64	11	69	8	69	7	68	9	65	14	70	14	70	16	68	15
Sylviidae	*Garrulax affinis*				7	33	5	35	5	35	5	36	4	35	9	38	12	38	12	39	10
Sylviidae	*Garrulax morrisonianus*				7	0	0	0	0	0	0	0	0	0	0	0	0	0	0	0	0
Sylviidae	*Garrulax erythrocephalus*				7	33	9	37	8	34	9	36	9	37	12	40	12	40	14	41	13
Sylviidae	*Garrulax yersini*	T		x	7	14	1x	9	1x	8	1x	8	1x	14	1x	9	12x	18	12x	20	12x
Sylviidae	*Garrulax formosus*				7	16	0	16	0	16	1	16	1	16	1	18	1	16	1	16	2
Sylviidae	*Garrulax milnei*				7	12	4	13	3	12	4	12	4	13	5	14	4	14	5	15	5
Sylviidae	*Liocichla phoenicea*				7	34	7	36	7	33	9	34	8	38	9	39	9	37	11	40	9
Sylviidae	*Liocichla omeiensis*	T			7	8	0	8	0	8	0	8	0	8	0	8	0	8	0	8	0
Sylviidae	*Liocichla steerii*		H		7	0	0	0	0	0	0	0	0	0	0	0	0	0	0	0	0

Family	Species	IUCN	Ag	R	Range Source	Percentage loss in geographic range 2050 TG H	TG C	AM H	AM C	GO H	GO C	OS H	OS C	2100 TG H	TG C	AM H	AM C	GO H	GO C	OS H	OS C
Sylviidae	*Modulatrix stictigula*				14	41	0	36	0	41	0	41	0	41	0	41	0	41	0	41	0
Sylviidae	*Arcanator orostruthus*	T		x	14	31	0x	14	16x	31	0x	31	0x	31	0x	15	16x	31	0x	31	0x
Sylviidae	*Trichastoma rostratum*				7	14	3	16	4	17	3	17	4	21	5	23	5	30	4	27	4
Sylviidae	*Trichastoma celebense*				7	14	13	27	9	19	13	16	12	16	14	30	21	29	29	24	26
Sylviidae	*Trichastoma bicolor*				7	13	3	16	3	15	3	17	3	20	4	22	4	28	4	27	3
Sylviidae	*Leonardina woodi*				7	19	14	26	18	8	18	17	19	29	17	26	18	24	22	34	19
Sylviidae	*Malacocincla abbotti*				7	17	5	20	5	18	6	22	5	22	9	24	9	27	11	29	8
Sylviidae	*Malacocincla sepiarium*				7	13	2	15	3	15	3	18	2	19	3	21	3	28	3	27	2
Sylviidae	*Malacocincla vanderbilti*				7	30	12	31	7	30	4	28	12	38	12	39	10	40	4	39	12
Sylviidae	*Malacocincla perspicillata*	T		x	14	100	0x	0	0x	100	0x	0	0x	100	0x	0	0x	100	0x	100	0x
Sylviidae	*Malacocincla malaccensis*				7	12	3	15	4	14	4	17	3	19	4	21	5	27	5	26	4
Sylviidae	*Malacocincla cinereiceps*			x	7	24	19x	24	23x	25	11x	22	19x	24	19x	24	43x	50	31x	33	49x
Sylviidae	*Pellorneum tickelli*				7	22	8	25	8	22	9	26	9	26	10	29	11	31	13	33	11
Sylviidae	*Pellorneum pyrrogenys*				7	8	0	12	1	11	2	13	1	15	1	17	1	20	2	22	1
Sylviidae	*Pellorneum albiventre*				7	26	9	30	8	26	11	30	10	30	11	33	11	31	14	36	12
Sylviidae	*Pellorneum palustre*	T			7	44	0	51	1	49	2	53	1	51	2	53	1	50	2	54	1
Sylviidae	*Pellorneum ruficeps*				7	18	5	21	5	19	5	23	5	20	8	23	8	23	10	31	7
Sylviidae	*Pellorneum fuscocapillum*				7	48	0x	53	0x	39	3x	51	0x	48	0x	56	0x	48	3x	58	0x
Sylviidae	*Pellorneum capistratum*				7	12	3	15	3	14	3	17	3	19	4	21	5	26	4	26	4
Sylviidae	*Malacopteron magnirostre*				7	13	3	16	4	15	4	18	3	19	4	21	5	27	5	27	4
Sylviidae	*Malacopteron affine*				7	12	2	16	3	15	3	18	2	19	3	22	3	28	3	27	2
Sylviidae	*Malacopteron cinereum*				7	12	5	15	5	14	5	17	5	18	6	20	8	25	9	26	7
Sylviidae	*Malacopteron magnum*				7	13	3	16	4	15	4	18	3	19	4	21	5	27	5	27	4
Sylviidae	*Malacopteron palawanense*			x	7	18	25x	17	30x	23	14x	16	25x	18	25x	17	46x	49	30x	27	50x
Sylviidae	*Malacopteron albogulare*				7	19	3	22	4	18	4	25	3	27	4	30	4	34	4	38	3
Sylviidae	*Illadopsis cleaveri*				5	25	5	14	5	21	4	43	3	42	5	25	6	46	5	53	4
Sylviidae	*Illadopsis albipectus*				5	38	7	27	10	41	7	62	5	57	7	38	10	69	8	70	5
Sylviidae	*Illadopsis rufescens*				5	28	3	17	1	20	4	38	1	32	5	23	5	36	5	39	1
Sylviidae	*Illadopsis puveli*				5	30	5	17	12	30	8	41	5	39	5	21	13	43	10	45	7
Sylviidae	*Illadopsis rufipennis*				5	33	4	22	6	31	4	57	3	52	5	33	7	60	5	66	4
Sylviidae	*Illadopsis fulvescens*				5	36	6	23	9	34	7	57	5	53	7	34	10	62	8	66	5
Sylviidae	*Illadopsis pyrrhoptera*				5	27	3	21	6	27	5	31	0	29	3	24	7	29	6	31	0
Sylviidae	*Pseudoalcippe abyssinica*				5	36	2	27	3	36	2	38	0	38	4	32	6	39	3	39	0
Sylviidae	*Kakamega poliothorax*				5	20	7	15	7	31	6	27	5	27	10	22	10	37	8	30	5
Sylviidae	*Ptyrticus turdinus*				5	43	4	22	14	42	7	62	2	57	4	28	15	58	7	64	2
Sylviidae	*Pomatorhinus hypoleucos*				7	17	8	21	7	18	9	21	8	20	12	23	11	23	15	26	11
Sylviidae	*Pomatorhinus erythrocnemi*				7	18	3	19	3	18	4	19	4	20	5	20	6	21	6	21	5
Sylviidae	*Pomatorhinus erythrogenys*				7	17	10	24	10	15	11	12	11	20	10	27	12	19	14	19	14
Sylviidae	*Pomatorhinus horsfieldii*				7	18	3	21	3	21	2	27	1	21	4	24	4	25	4	41	1
Sylviidae	*Pomatorhinus schisticeps*				7	23	8	27	8	24	9	27	9	26	13	30	13	29	17	32	13
Sylviidae	*Pomatorhinus montanus*				7	13	2	15	2	15	2	16	2	20	3	21	3	27	3	26	2
Sylviidae	*Pomatorhinus ruficollis*				7	18	4	20	3	18	4	20	4	20	5	21	5	21	6	22	5
Sylviidae	*Pomatorhinus ochraceicep*				7	28	11	31	10	27	13	28	12	33	15	34	14	32	17	35	15
Sylviidae	*Pomatorhinus ferruginosus*				7	36	8	40	8	35	10	36	10	41	11	43	11	40	13	43	12

Family	Species	IUCN	Ag	R	Range Source	Percentage loss in geographic range 2050 TG H	2050 TG C	2050 AM H	2050 AM C	2050 GO H	2050 GO C	2050 OS H	2050 OS C	2100 TG H	2100 TG C	2100 AM H	2100 AM C	2100 GO H	2100 GO C	2100 OS H	2100 OS C
Sylviidae	*Xiphirhynchus superciliaris*				7	45	9	50	9	45	12	50	8	52	12	54	12	52	14	57	11
Sylviidae	*Jabouilleia danjoui*				7	5	20	5	18	12	20	14	12	5	20	5	22	21	24	20	15
Sylviidae	*Rimator malacoptilus*				7	42	9	45	8	41	11	46	9	50	11	51	11	53	13	54	11
Sylviidae	*Ptilocichla leucogrammica*	T			7	8	1	13	1	12	2	14	1	16	1	18	1	21	2	23	1
Sylviidae	*Ptilocichla mindanensis*				7	20	14	27	15	13	14	27	16	31	17	27	15	27	20	41	16
Sylviidae	*Ptilocichla falcata*	T		x	7	23	19x	24	22x	25	11x	22	18x	23	19x	24	44x	50	32x	33	50x
Sylviidae	*Kenopia striata*				7	13	2	16	3	16	3	19	3	20	3	22	3	28	3	28	3
Sylviidae	*Napothera macrodactyla*				7	13	2	16	3	15	3	18	2	20	3	22	3	28	3	28	2
Sylviidae	*Napothera rufipectus*				7	25	6	25	6	26	4	27	6	30	6	33	7	47	4	41	6
Sylviidae	*Napothera atrigularis*				7	8	1	13	1	12	2	14	1	16	1	18	1	21	2	23	1
Sylviidae	*Napothera marmorata*				7	22	6	23	6	23	5	29	6	28	7	30	7	44	5	40	6
Sylviidae	*Napothera crispifrons*				7	14	13	19	11	14	15	15	15	16	15	21	15	21	19	22	18
Sylviidae	*Napothera brevicaudata*				7	20	10	22	9	20	11	23	10	24	13	25	14	26	16	29	14
Sylviidae	*Napothera crassa*				7	13	0	24	3	19	3	26	0	21	0	30	3	35	3	42	0
Sylviidae	*Napothera rabori*				7	7	8	21	5	13	9	16	6	7	19	33	12	25	21	27	7
Sylviidae	*Napothera epilepidota*				7	19	6	22	6	19	7	22	6	24	8	25	8	27	9	30	8
Sylviidae	*Pnoepyga albiventer*				7	48	3	50	3	49	3	51	2	52	5	54	6	55	6	55	5
Sylviidae	*Pnoepyga immaculata*				7	53	8x	59	9x	50	4x	67	0x	56	12x	59	13x	61	9x	67	5x
Sylviidae	*Pnoepyga pusilla*				7	17	4	19	4	18	4	19	4	20	5	21	5	22	6	22	5
Sylviidae	*Spelaeornis caudatus*				7	69	4	73	2	71	4	77	2	73	6	80	3	72	5	79	4
Sylviidae	*Spelaeornis badeigularis*	T		x	7	61	0x	86	0x	75	0x	86	0x	86	0x	94	0x	100	0x	92	0x
Sylviidae	*Spelaeornis troglodytoides*				7	33	2	34	2	34	1	35	2	35	3	36	4	36	3	35	3
Sylviidae	*Spelaeornis formosus*				7	47	4	50	5	46	7	50	4	51	6	54	8	51	11	56	7
Sylviidae	*Spelaeornis chocolatinus*				7	31	6	32	7	27	9	27	9	36	9	35	10	32	11	33	11
Sylviidae	*Spelaeornis longicaudatus*	T		x	7	72	0x	86	0x	61	0x	86	0x	72	0x	89	0x	83	0x	89	0x
Sylviidae	*Sphenocichla humei*				7	54	3	59	2	56	4	61	1	63	6	65	5	63	5	69	4
Sylviidae	*Neomixis tenella*				6	52	0	31	5	52	0	52	0	52	0	41	5	52	0	52	0
Sylviidae	*Neomixis viridis*				6	76	0	47	5	79	0	79	0	79	0	72	5	79	0	79	0
Sylviidae	*Neomixis striatigula*				6	46	0	29	3	47	0	47	0	47	0	39	3	47	0	47	0
Sylviidae	*Neomixis flavoviridis*				6	74	0	44	4	78	0	78	0	78	0	71	4	78	0	78	0
Sylviidae	*Stachyris ambigua*				7	37	10	39	9	35	13	37	12	42	12	43	12	42	15	45	14
Sylviidae	*Stachyris rodolphei*			x	7	17	0x	17	0x	20	0x	38	0x	17	0x	17	0x	38	0x	47	0x
Sylviidae	*Stachyris rufifrons*				7	13	4	16	4	15	4	16	4	19	5	21	5	25	5	25	5
Sylviidae	*Stachyris ruficeps*				7	16	4	17	3	16	4	16	4	17	5	18	4	17	5	19	5
Sylviidae	*Stachyris pyrrhops*				7	60	6	63	5	65	3	71	1	64	10	70	8	72	8	74	5
Sylviidae	*Stachyris chrysaea*				7	23	8	27	7	24	9	27	8	27	10	31	10	33	11	34	10
Sylviidae	*Stachyris plateni*				7	20	13	27	14	12	13	26	15	31	16	27	14	27	19	41	15
Sylviidae	*Stachyris dennistouni*				7	6	7	19	4	10	8	15	5	6	18	31	12	20	22	27	6
Sylviidae	*Stachyris nigrocapitata*			x	7	25	12x	28	11x	31	7x	40	12x	36	16x	34	11x	42	14x	46	14x
Sylviidae	*Stachyris capitalis*				7	17	15	27	17	8	17	19	18	27	18	27	17	25	23	35	18
Sylviidae	*Stachyris speciosa*	T		x	7	0	70x	24	46x	0	70x	0	70x	0	70x	24	46x	0	70x	0	70x
Sylviidae	*Stachyris whiteheadi*				7	6	7	19	4	10	8	15	5	6	18	31	11	20	22	27	6
Sylviidae	*Stachyris striata*				7	6	5	14	4	6	6	13	3	6	15	19	14	14	16	21	7
Sylviidae	*Stachyris latistriata*			x	7	46	0x	38	9x	0	9x	9	0x	46	0x	38	9x	0	9x	46	0x

Family	Species	IUCN	Ag	R	Range Source	2050 TG H	2050 TG C	2050 AM H	2050 AM C	2050 GO H	2050 GO C	2050 OS H	2050 OS C	2100 TG H	2100 TG C	2100 AM H	2100 AM C	2100 GO H	2100 GO C	2100 OS H	2100 OS C
Sylviidae	*Stachyris nigrorum*	T		x	7	0	71x	25	46x	0	71x	0	71x	0	71x	25	46x	0	71x	0	71x
Sylviidae	*Stachyris hypogrammica*			x	7	25	19x	25	22x	25	11x	22	18x	25	19x	25	43x	51	32x	34	50x
Sylviidae	*Stachyris grammiceps*				7	6	0	0	0	0	0	0	0	8	0	5	0	6	0	5	1
Sylviidae	*Stachyris herberti*				7	10	4	17	0	15	4	13	4	30	17	27	3	15	17	15	14
Sylviidae	*Stachyris nigriceps*				7	20	6	24	5	22	6	24	6	26	7	28	7	31	8	32	7
Sylviidae	*Stachyris poliocephala*				7	13	2	16	3	15	3	18	2	20	3	22	3	28	3	28	2
Sylviidae	*Stachyris oglei*	T		x	7	55	0x	85	0x	70	0x	85	0x	93	0x	91	0x	99	0x	85	0x
Sylviidae	*Stachyris striolata*				7	16	6	18	5	16	6	17	6	19	6	20	7	23	7	22	7
Sylviidae	*Stachyris leucotis*				7	13	2	16	3	16	3	19	2	20	3	22	3	28	3	28	2
Sylviidae	*Stachyris nigricollis*				7	13	2	16	3	15	3	18	2	20	3	22	3	28	3	28	2
Sylviidae	*Stachyris thoracica*				7	7	0	0	0	0	0	0	0	8	0	5	0	6	0	5	1
Sylviidae	*Stachyris maculata*				7	13	2	16	3	15	3	18	2	20	3	22	3	28	3	28	2
Sylviidae	*Stachyris erythroptera*				7	12	2	15	3	15	3	17	2	19	3	21	3	27	3	27	2
Sylviidae	*Stachyris melanothorax*				7	6	0	0	0	0	0	0	0	7	0	4	0	10	0	4	1
Sylviidae	*Dumetia hyperythra*				7	16	2	17	2	17	2	21	1	17	3	19	4	19	4	30	1
Sylviidae	*Rhopocichla atriceps*				7	29	3	41	2	34	2	44	1	31	5	46	2	39	5	63	1
Sylviidae	*Macronous gularis*				7	18	5	21	5	19	6	22	6	22	8	25	8	27	10	30	8
Sylviidae	*Macronous flavicollis*				7	6	0	0	0	0	0	0	0	8	0	5	0	6	0	5	1
Sylviidae	*Macronous kelleyi*				7	5	18	9	15	12	18	16	13	8	21	10	21	17	25	22	18
Sylviidae	*Macronous striaticeps*				7	21	13	26	16	12	14	26	16	32	16	27	16	27	20	39	16
Sylviidae	*Macronous ptilosus*				7	13	2	16	3	15	3	19	2	20	3	22	3	28	3	28	2
Sylviidae	*Micromacronus leytensis*				7	20	14	27	15	13	13	27	15	31	16	27	15	27	20	42	16
Sylviidae	*Timalia pileata*				7	17	6	20	6	17	7	20	7	19	10	22	10	21	12	24	9
Sylviidae	*Chrysomma sinense*				7	16	4	18	4	17	4	20	3	18	7	20	7	20	9	27	5
Sylviidae	*Chrysomma altirostre*	T			7	16	4	17	4	16	6	19	4	18	7	18	7	18	7	21	6
Sylviidae	*Chrysomma poecilotis*				7	21	1	20	1	20	1	20	1	21	1	22	4	21	2	20	3
Sylviidae	*Turdoides nipalensis*				7	53	7	60	5	59	3	64	0	55	7	63	7	63	4	67	2
Sylviidae	*Turdoides altirostris*		H		3,4	0	0	0	0	0	0	0	0	0	0	0	3	0	2	0	0
Sylviidae	*Turdoides caudatus*				3,4,7	9	3	9	2	9	2	12	2	10	5	11	6	11	8	17	2
Sylviidae	*Turdoides earlei*				7	14	2	14	2	14	3	16	2	15	3	16	5	16	8	19	2
Sylviidae	*Turdoides gularis*				7	11	7	12	8	7	11	13	9	12	11	13	13	11	14	18	10
Sylviidae	*Turdoides longirostris*	T			7	32	1	38	1	40	1	40	1	34	3	39	2	41	3	41	1
Sylviidae	*Turdoides malcolmi*				7	14	4	15	4	14	3	19	2	15	6	16	9	15	10	26	3
Sylviidae	*Turdoides squamiceps*				3,4	0	2	0	1	0	1	0	1	0	2	0	1	0	2	0	2
Sylviidae	*Turdoides fulvus*				3,4,5	6	3	4	3	6	3	7	2	7	3	4	6	8	8	7	7
Sylviidae	*Turdoides aylmeri*				5	32	3	10	18	38	1	39	1	36	4	14	22	39	3	39	2
Sylviidae	*Turdoides rubiginosus*				5	50	7	19	27	59	2	59	2	55	7	25	32	61	2	60	2
Sylviidae	*Turdoides subrufus*				7	29	1	46	3	38	2	50	2	32	3	52	3	43	3	76	2
Sylviidae	*Turdoides striatus*		H		7	0	3	0	3	0	2	0	1	0	4	0	6	0	8	0	2
Sylviidae	*Turdoides rufescens*			x	7	38	0x	34	0x	37	0x	37	0x	39	0x	37	0x	39	0x	38	0x
Sylviidae	*Turdoides affinis*		H		7	0	0	0	1	0	1	0	1	0	2	0	1	0	2	0	1
Sylviidae	*Turdoides reinwardii*				5	24	4	13	5	29	4	40	3	36	4	19	9	42	6	43	5
Sylviidae	*Turdoides tenebrosus*				5	47	9	31	18	55	5	56	5	49	9	37	18	56	5	56	5
Sylviidae	*Turdoides melanops*				5	10	1	6	5	10	1	10	1	10	4	6	11	10	5	10	2

Family	Species	IUCN	Ag	R	Range Source	Percentage loss in geographic range 2050 TG H	TG C	AM H	AM C	GO H	GO C	OS H	OS C	2100 TG H	TG C	AM H	AM C	GO H	GO C	OS H	OS C
Sylviidae	*Turdoides squamulatus*			x	5	13	8x	3	17x	21	0x	21	0x	13	8x	3	17x	21	0x	21	0x
Sylviidae	*Turdoides leucopygius*				5	31	0	19	3	33	0	33	0	33	0	23	5	33	1	33	1
Sylviidae	*Turdoides hartlaubii*				5	19	1	14	2	19	1	20	1	20	2	16	9	20	2	20	2
Sylviidae	*Turdoides bicolor*				5	9	7	3	10	8	8	9	8	9	12	3	20	8	16	9	14
Sylviidae	*Turdoides sharpei*				5	40	5	35	7	43	3	43	3	41	5	37	8	44	4	43	3
Sylviidae	*Turdoides hypoleucus*				5	26	5	22	5	29	2	29	2	26	5	30	5	34	2	33	2
Sylviidae	*Turdoides hindei*	T		x	5	15	0x	15	0x	15	0x	15	0x	15	0x	15	0x	15	0x	15	0x
Sylviidae	*Turdoides plebejus*				5	21	3	10	5	23	4	31	3	28	4	14	9	32	6	33	4
Sylviidae	*Turdoides leucocephalus*				5	24	2	18	5	27	2	27	2	27	2	20	7	27	4	27	4
Sylviidae	*Turdoides jardineii*				5	27	3	21	4	26	3	30	2	29	4	23	8	30	4	30	4
Sylviidae	*Turdoides gymnogenys*				5	20	6	13	8	20	4	18	6	20	12	13	17	20	7	18	9
Sylviidae	*Babax lanceolatus*				7	16	3	17	3	15	4	15	3	18	4	18	4	17	5	17	4
Sylviidae	*Babax waddelli*				7	51	20	58	15	55	18	54	19	54	30	64	26	60	29	58	30
Sylviidae	*Babax koslowi*				7	20	8	20	9	23	8	24	6	20	21	22	39	38	28	39	19
Sylviidae	*Leiothrix argentauris*				7	24	7	26	6	24	7	27	7	27	9	29	9	30	10	32	9
Sylviidae	*Leiothrix lutea*				7,11	19	2	21	2	20	2	21	1	21	4	23	5	23	5	23	4
Sylviidae	*Cutia nipalensis*				7	33	8	35	7	33	8	35	8	36	10	37	9	38	11	40	10
Sylviidae	*Pteruthius rufiventer*				7	44	4	47	4	44	5	46	3	50	7	50	7	50	8	52	6
Sylviidae	*Pteruthius flaviscapis*				7	19	4	22	4	20	5	22	4	23	6	24	6	26	7	27	6
Sylviidae	*Pteruthius xanthochlorus*				7	35	2	36	1	36	2	37	1	36	3	39	3	38	3	39	2
Sylviidae	*Pteruthius melanotis*				7	33	10	36	9	33	11	36	10	38	12	39	12	40	14	43	12
Sylviidae	*Pteruthius aenobarbus*				7	21	10	24	10	20	12	22	11	24	13	26	14	25	16	28	14
Sylviidae	*Gampsorhynchus rufulus*				7	19	9	23	8	20	10	23	9	22	13	26	12	25	16	29	12
Sylviidae	*Actinodura egertoni*				7	44	10	47	10	42	13	45	10	50	12	51	13	48	16	51	12
Sylviidae	*Actinodura ramsayi*				7	15	7	19	6	15	8	16	9	17	9	21	8	19	10	22	10
Sylviidae	*Actinodura nipalensis*			x	7	80	0x	76	0x	80	0x	80	0x	80	0x	80	0x	80	0x	80	0x
Sylviidae	*Actinodura waldeni*				7	40	8	40	8	37	10	41	8	45	11	44	13	43	15	46	11
Sylviidae	*Actinodura souliei*				7	16	1	15	1	14	1	15	0	16	1	16	1	16	1	16	0
Sylviidae	*Actinodura morrisoniana*			x	7	0	0x	0	0x	0	0x	0	0x	0	0x	0	0x	0	0x	0	0x
Sylviidae	*Minla cyanouroptera*				7	20	6	22	6	21	7	22	6	22	8	24	8	25	9	26	8
Sylviidae	*Minla strigula*				7	37	5	38	5	36	6	38	6	40	7	41	7	41	8	42	7
Sylviidae	*Minla ignotincta*				7	24	1	26	1	25	2	26	1	26	2	27	2	27	3	27	2
Sylviidae	*Alcippe chrysotis*				7	19	2	20	2	19	3	19	2	21	3	22	3	21	4	21	3
Sylviidae	*Alcippe variegaticeps*	T		x	7	0	0x	0	0x	0	0x	0	0x	0	0x	0	0x	0	0x	0	0x
Sylviidae	*Alcippe cinerea*				7	44	6	47	7	43	9	49	5	51	8	51	10	49	12	56	7
Sylviidae	*Alcippe castaneceps*				7	28	9	31	8	28	10	31	9	31	11	33	11	33	13	37	12
Sylviidae	*Alcippe vinipectus*				7	38	4	39	4	38	4	40	3	41	8	43	12	44	13	46	9
Sylviidae	*Alcippe striaticollis*				7	32	4	32	5	33	5	34	4	32	11	35	17	41	12	40	9
Sylviidae	*Alcippe ruficapilla*				7	18	0	18	0	17	1	18	1	19	1	18	1	19	1	19	1
Sylviidae	*Alcippe cinereiceps*				7	12	1	12	1	12	1	12	1	13	2	13	2	13	2	14	1
Sylviidae	*Alcippe ludlowi*				7	68	4	72	3	77	1	77	3	75	4	78	6	79	6	78	5
Sylviidae	*Alcippe rufogularis*				7	29	11	33	10	30	12	35	11	33	14	35	14	37	16	41	14
Sylviidae	*Alcippe brunnea*				7	15	3	16	2	15	3	16	3	16	3	17	3	17	4	18	3
Sylviidae	*Alcippe dubia*				7	0	29x	3	29x	0	29x	0	29x	3	29x	3	45x	0	45x	0	45x

Family	Species	IUCN	Ag	R	Range Source	Percentage loss in geographic range: 2050								2100							
						TG		AM		GO		OS		TG		AM		GO		OS	
						H	C	H	C	H	C	H	C	H	C	H	C	H	C	H	C
Sylviidae	*Alcippe brunneicauda*				7	13	2	16	3	16	3	18	2	20	3	22	3	28	3	28	2
Sylviidae	*Alcippe poioicephala*				7	18	6	20	5	19	6	23	5	20	7	22	8	22	10	31	6
Sylviidae	*Alcippe pyrrhoptera*			x	7	0	0x	0	0x	0	0x	0	0x	1	0x	0	0x	0	0x	0	0x
Sylviidae	*Alcippe peracensis*				7	11	15	14	14	16	16	25	14	15	19	16	17	26	19	31	16
Sylviidae	*Alcippe morrisonia*				7	10	3	11	3	10	3	10	3	11	4	11	3	12	4	12	4
Sylviidae	*Alcippe nipalensis*				7	39	6	44	6	40	8	44	6	44	9	48	10	45	11	50	8
Sylviidae	*Lioptilus nigricapillus*				5	3	0	3	0	3	0	3	0	3	0	3	0	3	0	3	0
Sylviidae	*Kupeornis gilberti*	T		x	5	8	12x	8	12x	45	0x	21	0x	31	21x	8	21x	45	0x	30	0x
Sylviidae	*Kupeornis rufocinctus*			x	5	27	0x	8	10x	27	0x	27	0x	27	0x	17	10x	27	0x	27	0x
Sylviidae	*Kupeornis chapini*				5	65	3	54	7	38	10	68	5	66	3	62	7	46	13	68	5
Sylviidae	*Parophasma galinieri*			x	5	63	0x	61	0x	63	0x	63	0x	63	0x	61	3x	63	0x	63	0x
Sylviidae	*Phyllanthus atripennis*				5	37	6	26	10	32	9	55	8	52	8	31	11	51	10	60	9
Sylviidae	*Crocias langbianis*	T		x	7	18	0x	9	0x	8	0x	8	0x	18	0x	9	12x	21	12x	24	12x
Sylviidae	*Crocias albonotatus*			x	7	0	0x	0	0x	0	0x	0	0x	1	0x	0	0x	0	0x	0	0x
Sylviidae	*Heterophasia annectens*				7	33	10	35	9	30	12	32	11	37	12	38	12	36	15	40	14
Sylviidae	*Heterophasia capistrata*				7	75	5	76	3	75	3	82	2	77	8	82	5	82	5	84	4
Sylviidae	*Heterophasia gracilis*				7	38	4	38	5	36	7	38	2	48	5	41	7	44	8	45	4
Sylviidae	*Heterophasia melanoleuca*				7	16	7	19	6	16	8	16	7	18	8	20	8	20	9	21	9
Sylviidae	*Heterophasia auricularis*				7	0	0	0	0	0	0	0	0	0	0	0	0	0	0	0	0
Sylviidae	*Heterophasia pulchella*				7	45	2	47	2	49	2	49	0	52	2	51	2	53	2	54	1
Sylviidae	*Heterophasia picaoides*				7	29	9	32	8	30	10	33	9	34	11	36	11	38	12	41	11
Sylviidae	*Yuhina castaniceps*				7	16	4	17	4	16	5	17	4	18	5	19	5	18	6	20	5
Sylviidae	*Yuhina everetti*				7	9	0	12	1	8	2	14	1	14	0	15	1	18	2	23	1
Sylviidae	*Yuhina bakeri*				7	41	8	43	8	39	11	41	9	47	10	47	11	43	14	47	11
Sylviidae	*Yuhina flavicollis*				7	42	8	44	7	40	9	44	9	47	11	47	11	46	12	49	11
Sylviidae	*Yuhina humilis*				7	16	11	21	12	9	14	9	15	20	11	24	15	12	17	15	18
Sylviidae	*Yuhina gularis*				7	33	7	34	6	31	8	33	7	35	9	37	9	35	11	37	9
Sylviidae	*Yuhina diademata*				7	14	1	15	1	14	1	15	1	14	2	15	2	15	2	16	2
Sylviidae	*Yuhina occipitalis*				7	39	3	43	3	42	2	44	1	43	4	45	4	45	4	47	2
Sylviidae	*Yuhina brunneiceps*				7	0	0	0	0	0	0	0	0	0	0	0	0	0	0	0	0
Sylviidae	*Yuhina nigrimenta*		H		7	0	4	0	3	0	4	0	3	0	4	0	4	0	5	0	4
Sylviidae	*Yuhina zantholeuca*				7	17	4	19	4	17	5	20	4	20	6	21	6	22	7	25	6
Sylviidae	*Myzornis pyrrhoura*				7	47	4	50	4	49	3	53	1	51	6	53	7	53	7	55	5
Sylviidae	*Oxylabes madagascariensi*				6	75	0	45	4	78	0	78	0	78	0	71	4	78	0	78	0
Sylviidae	*Crossleyia xanthophrys*				5,6	24	9	26	6	33	7	47	6	35	13	28	11	43	7	51	6
Sylviidae	*Mystacornis crossleyi*				6	75	0	45	4	78	0	78	0	78	0	71	4	78	0	78	0
Sylviidae	*Panurus biarmicus*				3,4,7	9	11	9	10	10	11	10	10	11	14	10	15	14	16	14	15
Sylviidae	*Conostoma oemodium*				7	25	3	27	2	27	2	28	2	26	5	29	6	29	6	29	5
Sylviidae	*Paradoxornis paradoxus*				7	12	1	12	0	12	1	12	0	12	1	13	0	12	1	12	0
Sylviidae	*Paradoxornis unicolor*				7	37	3	39	3	39	3	40	2	39	5	42	6	41	5	41	5
Sylviidae	*Paradoxornis gularis*				7	16	3	17	3	16	4	17	4	18	4	19	4	18	5	19	5
Sylviidae	*Paradoxornis flavirostris*	T			7	55	4	56	4	52	6	57	4	60	6	62	6	58	9	65	6
Sylviidae	*Paradoxornis guttaticollis*				7	16	4	17	4	15	5	16	5	17	5	18	5	18	6	18	6
Sylviidae	*Paradoxornis conspicillatus*				7	10	1	12	0	12	1	13	0	11	2	13	2	14	1	14	1

Family	Species	IUCN	Ag	R	Range Source	Percentage loss in geographic range 2050 TG H	C	AM H	C	GO H	C	OS H	C	2100 TG H	C	AM H	C	GO H	C	OS H	C
Sylviidae	*Paradoxornis webbianus*				7	9	1	9	1	9	1	8	1	9	1	9	2	9	1	9	1
Sylviidae	*Paradoxornis brunneus*				7	24	14	27	13	22	17	23	16	28	17	29	16	27	19	30	18
Sylviidae	*Paradoxornis alphonsianus*				7	21	0	20	1	20	1	21	0	21	0	20	2	22	1	23	1
Sylviidae	*Paradoxornis zappeyi*	T			7	3	0	3	0	3	0	3	0	3	0	3	0	3	0	3	0
Sylviidae	*Paradoxornis przewalskii*	T			7	24	4	28	0	24	4	28	0	24	4	28	0	24	4	28	0
Sylviidae	*Paradoxornis fulvifrons*		H		7	0	2	0	2	0	2	0	1	0	3	0	4	0	3	0	3
Sylviidae	*Paradoxornis nipalensis*				7	34	6	36	6	31	8	35	7	37	9	40	9	37	11	40	9
Sylviidae	*Paradoxornis verreauxi*				7	11	2	12	1	11	2	12	2	12	2	12	2	12	2	13	3
Sylviidae	*Paradoxornis davidianus*				7	21	10	24	8	20	11	20	11	23	11	26	10	25	13	26	13
Sylviidae	*Paradoxornis atrosupercilia*				7	34	10	37	9	32	12	34	12	39	13	40	12	37	15	42	14
Sylviidae	*Paradoxornis ruficeps*				7	26	10	31	8	25	11	30	10	30	12	34	11	32	13	37	11
Sylviidae	*Paradoxornis heudei*				7	2	1	2	1	2	2	2	2	3	1	2	2	2	2	2	2
Sylviidae	*Rhabdornis mystacalis*				7	13	12	20	12	11	13	16	13	17	17	24	15	22	20	26	15
Sylviidae	*Rhabdornis grandis*				7	5	6	16	4	7	7	13	4	5	17	23	13	16	18	22	6
Sylviidae	*Rhabdornis inornatus*				7	20	15	27	16	13	15	27	16	31	17	27	16	27	21	41	17
Sylviidae	*Chamaea fasciata*				2	0	46	4	32	1	45	4	23	1	48	4	35	4	46	6	26
Sylviidae	*Sylvia buryi*	T			4	0	0	0	0	0	0	0	0	0	0	0	0	0	4	0	4
Sylviidae	*Sylvia lugens*				14	46	1	28	7	49	1	49	2	49	1	36	9	50	1	50	2
Sylviidae	*Sylvia boehmi*				14	34	7	21	11	38	3	38	4	35	7	27	13	42	4	41	5
Sylviidae	*Sylvia layardi*				14	3	6	2	7	3	8	3	7	3	7	2	9	4	10	3	10
Sylviidae	*Sylvia subcaeruleum*				14	7	5	3	7	7	6	7	6	7	8	3	13	7	11	7	9
Sylviidae	*Sylvia atricapilla*				3,4,5,7	6	24	6	20	9	25	8	18	10	29	10	25	16	30	15	22
Sylviidae	*Sylvia borin*				3,4,5,7	6	22	5	19	8	23	7	18	9	26	8	23	14	27	14	21
Sylviidae	*Sylvia communis*		H		3,4,5,7	0	20	0	18	0	22	0	17	0	25	0	22	0	26	0	20
Sylviidae	*Sylvia curruca*				3,4,5,7	4	14	4	13	5	16	5	13	6	17	6	17	9	20	10	17
Sylviidae	*Sylvia minula*				4	10	4	10	3	10	3	15	3	20	6	15	4	22	4	26	4
Sylviidae	*Sylvia althaea*				4	15	3	15	4	19	3	22	2	19	4	22	5	25	4	33	3
Sylviidae	*Sylvia nana*				3,4,5,7	3	8	3	8	3	9	5	8	6	10	5	13	7	16	10	14
Sylviidae	*Sylvia nisoria*		H		3,4,5,7	0	19	0	17	0	22	0	16	0	21	0	19	0	24	0	18
Sylviidae	*Sylvia hortensis*		H		3,4,5	0	11	0	8	0	10	0	6	0	16	0	14	0	17	0	9
Sylviidae	*Sylvia leucomelaena*				3,4,5	0	1	0	1	0	1	0	1	0	1	0	1	0	2	0	2
Sylviidae	*Sylvia rueppelli*				3,4,5	20	5	20	5	21	6	29	4	29	5	26	5	29	6	30	5
Sylviidae	*Sylvia melanocephala*				3,4,5	6	8	7	5	7	7	9	4	9	10	8	8	8	10	10	5
Sylviidae	*Sylvia melanothorax*			x	3,4	26	0x	60	0x	60	0x	36	0x	70	0x	70	0x	70	0x	70	0x
Sylviidae	*Sylvia cantillans*				3,4,5	4	15	4	11	7	14	7	8	8	21	6	18	11	22	9	12
Sylviidae	*Sylvia mystacea*				3,4,5	6	5	6	6	7	7	7	6	7	7	8	7	8	8	14	7
Sylviidae	*Sylvia conspicillata*				3,4,5	12	6	12	4	12	6	13	4	13	7	13	5	13	7	15	4
Sylviidae	*Sylvia deserticola*				3,4,5	8	1	8	1	8	1	8	1	8	1	8	1	8	1	8	1
Sylviidae	*Sylvia undata*				3,4	2	24	4	10	5	19	5	5	3	32	6	21	8	27	7	9
Sylviidae	*Sylvia sarda*			x	3,4,5	1	5x	4	5x	2	5x	8	5x	1	11x	6	11x	4	11x	13	11x
Alaudidae	*Mirafra passerina*				5	13	4	7	6	12	4	12	4	13	7	7	12	12	7	12	7
Alaudidae	*Mirafra cantillans*				4,5,7,8	11	3	8	4	12	3	16	2	13	5	10	8	15	8	18	4
Alaudidae	*Mirafra javanica*				7,8,10	3	12	4	9	3	11	4	8	4	16	5	14	5	18	6	10
Alaudidae	*Mirafra cheniana*				5	8	0	4	4	8	2	8	1	8	3	4	7	8	5	8	4

Family	Species	IUCN	Ag	R	Range Source	Percentage loss in geographic range															
						2050								2100							
						TG		AM		GO		OS		TG		AM		GO		OS	
						H	C	H	C	H	C	H	C	H	C	H	C	H	C	H	C
Alaudidae	*Mirafra albicauda*				5	37	3	26	7	39	1	40	1	38	4	30	8	42	2	41	2
Alaudidae	*Mirafra hova*				6	44	0	25	4	44	0	44	0	44	0	33	4	44	0	44	0
Alaudidae	*Mirafra cordofanica*				3,5	3	3	2	3	3	3	5	2	5	3	2	5	5	5	6	4
Alaudidae	*Mirafra williamsi*				5	53	20x	11	43	72	3x	72	3x	55	20x	11	61x	72	3x	72	3x
Alaudidae	*Mirafra pulpa*			x	5	6	0x	6	0x	6	0x	6	0x	6	0x	6	0x	6	0x	6	0x
Alaudidae	*Mirafra hypermetra*				5	42	5	18	21	48	1	48	1	44	5	21	25	48	1	48	1
Alaudidae	*Mirafra somalica*				5	0	0	0	0	0	0	0	0	0	0	0	0	0	2	0	1
Alaudidae	*Mirafra ashi*	T		x	14	0	0x	0	0x	0	0x	0	0x	0	0x	0	0x	0	0x	0	0x
Alaudidae	*Mirafra africana*				5	20	3	14	4	19	3	21	3	21	5	15	11	20	7	21	5
Alaudidae	*Mirafra angolensis*				5	28	0	26	0	27	0	33	0	28	0	26	4	31	0	33	0
Alaudidae	*Mirafra rufocinnamomea*				5	24	2	15	6	25	2	28	1	27	3	18	9	28	3	28	2
Alaudidae	*Mirafra apiata*				5	6	5	2	6	5	5	6	5	6	7	2	12	5	10	6	8
Alaudidae	*Mirafra collaris*				5	35	3	7	29	39	1	40	1	36	4	9	31	39	5	40	3
Alaudidae	*Mirafra africanoides*				5	17	5	8	10	19	5	19	4	18	7	10	16	19	8	19	7
Alaudidae	*Mirafra erythroptera*		H		7	0	2	0	2	0	2	0	1	0	4	0	8	0	10	0	2
Alaudidae	*Mirafra assamica*		H		7	0	3	0	3	0	3	0	3	0	6	0	7	0	9	0	4
Alaudidae	*Mirafra rufa*				5	2	1	1	3	4	2	4	2	5	2	1	6	5	6	4	6
Alaudidae	*Mirafra gilletti*				5	39	3	11	23	47	1	47	1	44	4	15	28	47	5	47	3
Alaudidae	*Mirafra degodiensis*	T		x	14	94	0x	0	58x	100	0x	100	0x	100	0x	42	58x	100	0x	100	0x
Alaudidae	*Mirafra poecilosterna*				5	45	6	19	24	52	1	52	1	47	6	23	28	52	1	52	1
Alaudidae	*Mirafra sabota*				5	6	6	3	8	6	7	6	7	6	9	3	12	6	10	6	10
Alaudidae	*Pinarocorys erythropygia*				5	14	2	6	3	16	2	21	1	20	2	9	6	22	5	22	3
Alaudidae	*Pinarocorys nigricans*				5	36	2	26	3	35	2	39	2	38	4	27	8	38	3	39	3
Alaudidae	*Heteromirafra archeri*	T		x	5	0	0x	0	0x	0	0x	0	0x	0	0x	0	0x	0	0x	0	0x
Alaudidae	*Heteromirafra sidamoensis*	T		x	14	91	0x	8	38x	91	0x	91	0x	91	0x	52	38x	91	0x	91	0x
Alaudidae	*Heteromirafra ruddi*	T			5	0	0	0	0	0	0	0	0	0	0	0	0	0	0	0	0
Alaudidae	*Certhilauda curvirostris*				5	6	6	4	8	6	8	6	7	6	9	4	13	6	11	6	12
Alaudidae	*Certhilauda chuana*				5	0	0	0	7	0	7	0	6	0	11	0	11	0	10	0	7
Alaudidae	*Certhilauda erythrochlamys*				5	0	45	0	55x	0	55x	0	55x	0	55x	0	66x	0	66x	0	66x
Alaudidae	*Certhilauda albescens*				5	2	6	1	6	2	7	1	6	2	7	1	8	2	10	2	10
Alaudidae	*Certhilauda burra*	T		x	5	5	0x	0	0x	5	0x	5	0x	5	0x	0	2x	5	11x	5	11x
Alaudidae	*Chersomanes albofasciata*				5	7	7	3	9	7	8	7	7	7	10	3	16	7	14	7	13
Alaudidae	*Eremopterix leucotis*				5	18	3	9	8	19	3	21	2	20	4	11	12	21	6	21	5
Alaudidae	*Eremopterix australis*				5	1	6	1	8	1	8	1	7	1	9	1	12	1	13	1	12
Alaudidae	*Eremopterix verticalis*				5	7	7	3	9	6	8	6	7	7	10	3	16	6	14	7	13
Alaudidae	*Eremopterix leucopareia*				5	46	3	33	9	49	2	49	2	47	3	39	10	52	2	51	2
Alaudidae	*Eremopterix signata*				4,5	37	5	9	29	44	1	44	1	40	5	10	33	44	3	44	2
Alaudidae	*Eremopterix nigriceps*				3,4,5,7	5	2	3	2	5	2	7	1	7	3	4	5	8	6	8	3
Alaudidae	*Eremopterix grisea*				7	11	3	12	3	12	3	15	2	12	5	13	7	13	9	20	3
Alaudidae	*Ammomanes cincturus*				3,4,5,7	2	2	2	2	3	2	4	2	4	3	2	3	4	3	5	3
Alaudidae	*Ammomanes phoenicurus*				7	12	4	12	4	12	3	16	2	12	6	13	10	13	12	22	3
Alaudidae	*Ammomanes deserti*				3,4,5,7	4	3	3	2	4	2	6	2	6	3	4	3	6	4	7	3
Alaudidae	*Ammomanes grayi*				5	5	20	0	24	5	26	4	24	5	26	0	42	5	39	4	40
Alaudidae	*Alaemon alaudipes*				3,4,5,7	3	2	3	2	3	2	4	2	4	2	3	3	4	3	5	2

Family	Species	IUCN	Ag	R	Range Source	Percentage loss in geographic range 2050 TG		AM		GO		OS		2100 TG		AM		GO		OS	
						H	C	H	C	H	C	H	C	H	C	H	C	H	C	H	C
Alaudidae	Alaemon hamertoni				5	0	0	0	0	0	0	0	0	0	0	0	0	0	0	0	0
Alaudidae	Ramphocoris clotbey				3,4	8	5	8	5	7	6	8	5	8	5	8	6	7	7	8	6
Alaudidae	Melanocorypha calandra		H		3,4,7	0	6	0	5	0	7	0	5	0	8	0	7	0	9	0	6
Alaudidae	Melanocorypha bimaculata				3,4,5,7	6	5	6	5	7	6	9	5	10	6	9	9	10	12	15	10
Alaudidae	Melanocorypha maxima				7	15	19	16	18	16	18	17	17	16	32	20	40	26	40	25	36
Alaudidae	Melanocorypha mongolica				7	15	5	15	6	15	7	15	6	15	7	15	9	16	9	16	9
Alaudidae	Melanocorypha leucoptera				3,4,7	2	15	1	14	1	20	0	12	3	18	2	14	3	20	7	13
Alaudidae	Melanocorypha yeltoniensi				3,4,7	2	13	1	11	0	16	0	10	3	15	3	12	3	16	6	11
Alaudidae	Calandrella brachydactyla				3,4,5,7	7	10	7	10	8	11	9	9	9	14	9	17	11	19	13	16
Alaudidae	Calandrella cinerea				4,5	22	4	15	5	21	4	25	4	24	6	17	10	24	7	26	6
Alaudidae	Calandrella acutirostris				4,7	14	15	15	15	15	15	16	14	16	24	18	30	22	31	23	28
Alaudidae	Calandrella rufescens		H		3,4,5	0	4	0	3	0	4	0	3	0	5	0	4	0	5	0	4
Alaudidae	Calandrella cheleensis				3,4,7	8	11	9	11	9	11	9	11	10	15	11	20	13	21	14	19
Alaudidae	Calandrella raytal				4,7	8	2	10	1	9	2	10	1	9	3	10	5	10	7	12	2
Alaudidae	Calandrella somalica				5	22	1	10	6	23	0	23	0	22	1	15	6	25	0	24	0
Alaudidae	Spizocorys conirostris				5	8	4	3	6	7	4	7	4	8	6	3	12	7	9	7	7
Alaudidae	Spizocorys sclateri				5	2	7	0	7	2	7	1	7	2	7	0	8	2	8	1	8
Alaudidae	Spizocorys obbiensis				5	0	0	0	0	0	0	0	0	0	0	0	0	0	0	0	0
Alaudidae	Spizocorys personata				5	76	7	8	58	88	0	88	0	81	7	10	66	88	2	88	2
Alaudidae	Spizocorys fringillaris	T			5	0	0	0	0	0	0	0	0	0	0	0	0	0	0	0	0
Alaudidae	Eremalauda starki				5	5	10	1	13	4	13	4	12	5	14	1	21	4	21	4	21
Alaudidae	Eremalauda dunni				3,4,5	2	4	0	4	2	4	3	3	3	4	0	4	3	5	4	5
Alaudidae	Chersophilus duponti				3,4,5	18	7	18	6	17	7	18	6	18	8	18	9	17	12	18	7
Alaudidae	Galerida cristata				3,4,5,7	7	6	5	7	8	7	9	6	9	7	7	10	10	11	12	9
Alaudidae	Galerida theklae				3,4,5	17	5	9	8	18	4	19	3	18	5	11	11	18	6	19	4
Alaudidae	Galerida malabarica				7	26	4	43	3	39	3	47	0	28	5	49	4	43	5	72	0
Alaudidae	Galerida deva		H		7	0	4	0	3	0	5	0	2	0	5	0	10	0	13	0	4
Alaudidae	Galerida modesta				5	12	2	7	3	16	1	19	1	18	2	9	4	22	2	21	2
Alaudidae	Galerida magnirostris		H		5	0	4	0	5	0	6	0	5	0	6	0	6	0	7	0	8
Alaudidae	Pseudalaemon fremantlii				5	17	3	6	11	21	1	21	1	18	4	9	12	21	4	22	3
Alaudidae	Lullula arborea				3,4,5	8	21	8	17	11	21	11	15	14	24	12	21	19	25	17	17
Alaudidae	Alauda arvensis				,3,4,7,10,11,12,1	5	14	5	13	6	15	6	12	8	17	7	17	9	20	9	16
Alaudidae	Alauda japonica		H		7	0	20	0	18	0	20	0	20	0	32	0	38	0	35	0	34
Alaudidae	Alauda gulgula		H		4,7	0	7	0	7	0	7	0	6	0	11	0	13	0	14	0	11
Alaudidae	Alauda razae	T		x	5	35	0x	0	0x	59	0x	59	0x	35	0x	0	0x	59	0x	59	0x
Alaudidae	Eremophila alpestris				2,3,4,7	6	16	7	15	6	16	7	14	7	21	8	24	10	26	11	22
Alaudidae	Eremophila bilopha				3,4	7	5	7	4	7	5	7	4	7	5	7	5	7	6	7	5
Nectariniidae	Promerops gurneyi			x	5	4	0x	4	2x	4	2x	4	2x	4	2x	4	3x	4	2x	4	2x
Nectariniidae	Promerops cafer				5	0	0	0	0	0	1	0	0	0	0	0	2	0	3	0	2
Nectariniidae	Prionochilus olivaceus				7	20	14	28	15	16	14	26	16	30	17	30	15	31	20	38	16
Nectariniidae	Prionochilus maculatus				7	13	2	16	3	15	3	19	2	20	3	22	3	28	3	28	3
Nectariniidae	Prionochilus percussus				7	13	2	16	3	15	3	18	2	19	3	22	3	28	3	27	2
Nectariniidae	Prionochilus plateni		H	x	7	0	32x	0	36x	0	23x	0	24x	0	32x	0	49x	0	36x	0	45x
Nectariniidae	Prionochilus xanthopygius				7	8	0	12	1	11	2	13	1	15	1	17	1	20	2	22	1

Family	Species	IUCN	Ag	R	Range Source	Percentage loss in geographic range 2050 TG H		2050 AM H		2050 GO H		2050 OS H		2100 TG H		2100 AM H		2100 GO H		2100 OS H	
						TG H	TG C	AM H	AM C	GO H	GO C	OS H	OS C	TG H	TG C	AM H	AM C	GO H	GO C	OS H	OS C
Nectariniidae	*Prionochilus thoracicus*				7	13	2	16	3	16	3	19	2	20	3	22	3	29	3	28	2
Nectariniidae	*Dicaeum annae*		H		7	0	7	0	12	0	12	0	7	0	24	0	29	0	16	0	7
Nectariniidae	*Dicaeum agile*		H		7	0	4	0	4	0	4	0	4	0	6	0	7	0	8	0	5
Nectariniidae	*Dicaeum aeruginosum*		H		7	0	13	0	13	0	15	0	14	0	19	0	18	0	22	0	17
Nectariniidae	*Dicaeum everetti*				7	8	0	13	1	11	2	14	1	15	1	18	1	21	2	23	1
Nectariniidae	*Dicaeum proprium*				7	18	14	25	18	8	18	17	18	28	17	25	18	24	22	34	18
Nectariniidae	*Dicaeum chrysorrheum*		H		7	0	5	0	5	0	6	0	5	0	8	0	8	0	10	0	7
Nectariniidae	*Dicaeum melanoxanthum*				7	39	5	41	4	38	5	38	5	42	6	43	5	41	6	42	6
Nectariniidae	*Dicaeum vincens*				7	48	0x	53	0x	40	3x	51	0x	48	0x	56	0x	48	3x	58	0x
Nectariniidae	*Dicaeum aureolimbatum*				7	14	13	25	10	17	14	17	13	16	14	28	20	26	27	23	25
Nectariniidae	*Dicaeum nigrilore*				7	19	14	25	18	8	19	17	19	29	18	25	18	23	23	33	19
Nectariniidae	*Dicaeum anthonyi*				7	12	10	23	10	9	13	16	12	17	17	29	14	22	22	30	12
Nectariniidae	*Dicaeum bicolor*				7	15	11	24	10	13	12	22	11	20	18	30	14	25	21	34	12
Nectariniidae	*Dicaeum quadricolor*	T		x	13	0	16x	14	2x	0	16x	0	16x	0	16x	14	2x	0	16x	0	16x
Nectariniidae	*Dicaeum haematostictum*	T		x	14	31	21x	31	21x	0	28x	7	21x	31	21x	31	21x	0	28x	31	21x
Nectariniidae	*Dicaeum australe*		H		7	0	12	0	12	0	13	0	14	0	18	0	15	0	20	0	16
Nectariniidae	*Dicaeum retrocinctum*	T		x	7	0	7x	3	2x	1	7x	43	5x	0	26x	21	5x	5	26x	43	9x
Nectariniidae	*Dicaeum trigonostigma*				7	14	4	17	5	16	5	18	5	21	6	22	7	27	7	27	6
Nectariniidae	*Dicaeum hypoleucum*				7	13	11	24	11	14	12	21	11	19	16	31	13	28	20	32	12
Nectariniidae	*Dicaeum erythrorhynchos*		H		7	0	2	0	2	0	2	0	1	0	3	0	4	0	4	0	2
Nectariniidae	*Dicaeum concolor*		H		7	0	3	0	3	0	4	0	3	0	5	0	5	0	6	0	4
Nectariniidae	*Dicaeum pygmaeum*				7	15	13	24	12	14	13	22	12	21	18	30	16	27	23	34	15
Nectariniidae	*Dicaeum nehrkorni*				7	14	13	26	9	18	13	16	10	16	14	30	22	28	31	24	27
Nectariniidae	*Dicaeum erythrothorax*				7	18	0	24	0	20	0	20	0	31	0	25	0	37	0	33	0
Nectariniidae	*Dicaeum vulneratum*			x	7	16	0x	19	0x	21	0x	21	0x	34	0x	19	0x	25	0x	44	0x
Nectariniidae	*Dicaeum pectorale*				8	12	1	2	1	5	1	1	2	13	1	9	2	9	2	14	2
Nectariniidae	*Dicaeum geelvinkianum*				8	3	3	6	2	18	1	16	1	9	6	13	6	30	5	27	2
Nectariniidae	*Dicaeum nitidum*			x	8	52	0x	0	0x	33	0x	71	0x	61	0x	58	0x	33	0x	91	0x
Nectariniidae	*Dicaeum eximium*				8	5	0	7	0	50	0	43	0	26	0	23	0	71	0	56	0
Nectariniidae	*Dicaeum aeneum*		H		9	0	6	0	1	0	1	0	1	0	10	0	1	0	2	0	1
Nectariniidae	*Dicaeum tristrami*			x	9	49	0x	34	0x	40	0x	77	0x	49	0x	34	0x	40	0x	99	0x
Nectariniidae	*Dicaeum igniferum*			x	7	13	7x	14	11x	3	11x	5	7x	13	21x	14	25x	14	14x	17	7x
Nectariniidae	*Dicaeum maugei*			x	7	10	19x	2	19x	6	14x	9	16x	10	19x	2	20x	18	15x	9	16x
Nectariniidae	*Dicaeum ignipectus*				7	19	5	20	4	19	5	21	5	21	6	22	6	23	7	25	6
Nectariniidae	*Dicaeum monticolum*				7	9	0	13	1	8	2	12	1	15	0	16	1	17	2	20	1
Nectariniidae	*Dicaeum celebicum*		H		7	0	13	0	9	0	13	0	13	0	14	0	20	0	26	0	24
Nectariniidae	*Dicaeum sanguinolentum*				7	8	4	4	5	3	4	0	5	9	4	7	6	10	4	7	6
Nectariniidae	*Dicaeum hirundinaceum*				7,8,10	1	17	2	11	3	15	3	7	3	20	2	16	4	20	5	9
Nectariniidae	*Dicaeum cruentatum*		H		7	0	5	0	5	0	6	0	6	0	8	0	8	0	10	0	8
Nectariniidae	*Dicaeum trochileum*		H		7	0	0	0	0	0	0	0	0	0	2	0	3	0	1	0	1
Nectariniidae	*Anthreptes fraseri*				5	34	5	23	7	33	6	53	4	49	5	33	8	58	7	61	4
Nectariniidae	*Anthreptes reichenowi*				5	37	0	26	0	37	0	37	0	37	0	29	0	37	0	37	0
Nectariniidae	*Anthreptes anchietae*				5	53	0	41	0	54	0	55	0	56	0	43	3	55	0	55	0
Nectariniidae	*Anthreptes simplex*				7	13	2	16	3	15	3	19	2	20	3	22	3	28	3	28	2

Family	Species	IUCN	Ag	R	Range Source	Percentage loss in geographic range 2050 TG H	TG C	AM H	AM C	GO H	GO C	OS H	OS C	2100 TG H	TG C	AM H	AM C	GO H	GO C	OS H	OS C
Nectariniidae	*Anthreptes malacensis*		H		7	0	6	0	6	0	6	0	6	0	10	0	10	0	12	0	9
Nectariniidae	*Anthreptes rhodolaema*				7	13	2	16	3	16	3	19	3	20	3	22	3	29	3	28	3
Nectariniidae	*Anthreptes singalensis*				7	15	5	18	5	16	6	18	5	19	8	22	8	24	9	26	7
Nectariniidae	*Anthreptes gabonicus*				5	21	5	11	5	19	5	31	3	30	5	18	8	35	7	37	4
Nectariniidae	*Anthreptes longuemarei*				5	38	3	25	5	40	4	49	2	46	5	29	9	49	6	51	4
Nectariniidae	*Anthreptes orientalis*				5	39	4	15	20	44	1	44	1	41	4	18	24	45	2	45	2
Nectariniidae	*Anthreptes neglectus*				5	45	0	31	0	45	0	45	0	45	0	35	0	45	0	45	0
Nectariniidae	*Anthreptes aurantium*				5	35	6	19	9	28	8	54	5	52	6	29	11	54	9	62	5
Nectariniidae	*Anthreptes pallidigaster*	T		x	5	0	0x	0	0x	0	0x	0	0x	0	0x	0	0x	0	0x	0	0x
Nectariniidae	*Anthreptes rectirostris*				5	36	5	24	7	34	5	52	4	50	5	33	8	55	5	59	4
Nectariniidae	*Anthreptes rubritorques*	T		x	5	15	0x	15	0x	15	0x	15	0x	15	0x	15	0x	15	0x	15	0x
Nectariniidae	*Anthreptes collaris*				5	36	3	25	5	34	4	46	2	44	4	30	7	46	5	50	3
Nectariniidae	*Anthreptes platurus*				4,5	10	3	4	4	10	3	15	2	14	3	6	6	16	6	16	5
Nectariniidae	*Anthreptes metallicus*				3,4,5	1	1	0	1	2	0	1	0	1	1	0	1	2	1	1	1
Nectariniidae	*Hypogramma hypogrammic*				7	13	5	17	5	16	6	18	5	18	7	21	7	26	8	27	7
Nectariniidae	*Dreptes thomensis*	T		x	5	0	-102x	0	-102x	100	0x	100	0x	0	-102x	0	-102x	100	0x	100	0x
Nectariniidae	*Nectarinia seimundi*				5	33	6	23	8	32	6	52	5	49	7	33	9	56	7	60	5
Nectariniidae	*Nectarinia batesi*				5	25	4	16	5	29	3	43	3	42	5	25	6	48	4	53	4
Nectariniidae	*Nectarinia olivacea*				5	46	0	39	1	46	0	46	0	46	0	41	2	46	0	46	0
Nectariniidae	*Nectarinia obscura*				14	38	5	26	8	37	6	55	3	51	6	34	10	57	7	62	4
Nectariniidae	*Nectarinia violacea*				5	4	2	4	2	0	5	4	2	4	2	4	4	0	8	4	4
Nectariniidae	*Nectarinia veroxii*				5	20	0	15	0	20	0	20	0	20	1	16	0	22	0	22	0
Nectariniidae	*Nectarinia reichenbachii*				5	35	6	19	11	34	5	37	6	48	8	30	13	53	6	50	8
Nectariniidae	*Nectarinia hartlaubii*			x	5	0	0x	0	0x	0	0x	0	0x	0	0x	0	0x	0	0x	0	0x
Nectariniidae	*Nectarinia newtonii*			x	5	0	-102x	0	-102x	100	0x	100	0x	0	-102x	0	-102x	100	0x	100	0x
Nectariniidae	*Nectarinia oritis*				5	26	7	14	10	18	15	25	14	30	12	27	13	40	15	27	17
Nectariniidae	*Nectarinia alinae*			x	5	27	6x	22	8x	34	5x	30	0x	29	6x	26	9x	35	6x	30	0x
Nectariniidae	*Nectarinia verticalis*				5	32	6	21	8	32	8	47	5	43	7	27	13	47	11	51	7
Nectariniidae	*Nectarinia bannermani*				5	51	0	26	0	53	0	71	0	69	0	31	1	65	0	72	0
Nectariniidae	*Nectarinia cyanolaema*				5	36	6	25	8	35	7	56	4	52	7	35	10	58	7	63	5
Nectariniidae	*Nectarinia balfouri*			x	4,5	0	0x	0	0x	0	0x	0	0x	0	0x	0	0x	0	0x	0	0x
Nectariniidae	*Nectarinia fuliginosa*				5	34	7	24	8	35	5	43	4	42	8	32	9	45	6	48	5
Nectariniidae	*Nectarinia amethystina*				5	39	2	31	2	38	2	43	1	42	3	32	6	41	2	43	2
Nectariniidae	*Nectarinia rubescens*				5	40	8	28	10	40	8	61	4	55	9	37	12	63	9	67	5
Nectariniidae	*Nectarinia senegalensis*				5	24	2	16	5	26	2	28	1	27	3	19	8	28	2	28	2
Nectariniidae	*Nectarinia hunteri*				5	37	3	8	24	42	1	42	1	40	3	12	28	42	2	42	2
Nectariniidae	*Nectarinia adelberti*				5	26	10	14	13	26	9	34	7	32	11	19	16	35	11	36	9
Nectariniidae	*Nectarinia zeylonica*		H		7	0	2	0	2	0	2	0	1	0	3	0	3	0	4	0	1
Nectariniidae	*Nectarinia minima*		H		7	0	4	0	3	0	2	0	1	0	5	0	3	0	6	0	1
Nectariniidae	*Nectarinia sperata*		H		7	0	5	0	6	0	6	0	6	0	7	0	9	0	10	0	8
Nectariniidae	*Nectarinia aspasia*				7,8	6	5	10	4	19	4	17	3	13	6	17	8	29	8	26	7
Nectariniidae	*Nectarinia calcostetha*		H		7	0	4	0	5	0	5	0	5	0	7	0	9	0	8	0	8
Nectariniidae	*Nectarinia jugularis*		H		7,8,9,10	0	7	0	6	0	7	0	6	0	10	0	10	0	12	0	9
Nectariniidae	*Nectarinia buettikoferi*		H	x	7	0	0x	0	0x	0	0x	0	0x	0	25x	0	26x	0	25x	0	0x

Family	Species	IUCN	Ag	R	Range Source	Percentage loss in geographic range 2050 TG H	C	AM H	C	GO H	C	OS H	C	2100 TG H	C	AM H	C	GO H	C	OS H	C
Nectariniidae	*Nectarinia solaris*		H		7	0	13	0	16	0	13	0	13	0	22	0	25	0	15	0	13
Nectariniidae	*Nectarinia sovimanga*				6,13	49	0	27	5	50	0	50	0	50	0	37	5	50	0	50	0
Nectariniidae	*Nectarinia humbloti*			x	13	17	0x	17	0x	17	0x	17	0x	17	0x	17	0x	17	0x	17	0x
Nectariniidae	*Nectarinia comorensis*			x	13	0	0x	0	0x	0	0x	0	0x	0	0x	0	0x	0	0x	0	0x
Nectariniidae	*Nectarinia coquerellii*			x	13	100	0x	0	0x	100	0x	100	0x	100	0x	100	0x	100	0x	100	0x
Nectariniidae	*Nectarinia venusta*				5	39	3	25	9	42	3	46	2	44	4	29	13	46	4	47	3
Nectariniidae	*Nectarinia ursulae*			x	5	5	17x	5	17x	77	0x	57	0x	35	35x	5	35x	77	0x	75	0x
Nectariniidae	*Nectarinia talatala*		H		5	0	1	0	3	0	2	0	2	0	4	0	8	0	3	0	3
Nectariniidae	*Nectarinia oustaleti*				5	46	0	40	0	46	0	46	0	46	0	41	3	46	0	46	0
Nectariniidae	*Nectarinia bouvieri*				5	37	9	33	10	34	13	54	8	47	12	40	13	50	16	55	10
Nectariniidae	*Nectarinia osea*				3,4,5	1	5	2	3	5	2	6	2	5	5	4	3	6	3	7	3
Nectariniidae	*Nectarinia asiatica*		H		4,7	0	4	0	4	0	4	0	3	0	7	0	8	0	10	0	5
Nectariniidae	*Nectarinia habessinica*				4,5	43	6	11	26	51	1	51	1	47	6	16	30	51	2	51	2
Nectariniidae	*Nectarinia lotenia*		H		7	0	2	0	2	0	2	0	1	0	3	0	2	0	3	0	1
Nectariniidae	*Nectarinia manoensis*		H		5	0	1	0	1	0	1	0	1	0	2	0	5	0	1	0	1
Nectariniidae	*Nectarinia chalybea*		H		5	0	5	0	6	0	7	0	6	0	7	0	8	0	10	0	9
Nectariniidae	*Nectarinia afra*				5	1	0	1	0	1	1	1	0	1	1	1	1	1	1	1	1
Nectariniidae	*Nectarinia ludovicensis*				5	41	0	26	0	41	0	41	0	41	0	28	0	41	0	41	0
Nectariniidae	*Nectarinia stuhlmanni*			x	5	20	5x	16	9x	26	5x	22	0x	22	5x	16	9x	27	5x	22	0x
Nectariniidae	*Nectarinia preussi*				5	24	8	21	12	31	10	44	1	34	11	26	13	40	11	45	3
Nectariniidae	*Nectarinia mediocris*				5	36	1	35	1	36	0	36	0	36	1	35	5	37	0	36	0
Nectariniidae	*Nectarinia neergaardi*				5	21	0	21	0	21	0	21	0	21	1	21	1	28	0	28	0
Nectariniidae	*Nectarinia chloropygia*				5	35	6	23	8	34	7	54	4	50	7	33	9	58	7	62	4
Nectariniidae	*Nectarinia minulla*				5	35	5	21	8	29	10	54	6	47	7	35	11	51	11	62	6
Nectariniidae	*Nectarinia regia*				5	24	9	21	11	31	5	33	0	26	9	25	12	33	9	33	0
Nectariniidae	*Nectarinia loveridgei*			x	5	100	0x	100	0x	100	0x	100	0x	100	0x	100	0x	100	0x	100	0x
Nectariniidae	*Nectarinia moreaui*			x	5	25	0x	25	0x	25	0x	25	0x	25	0x	25	0x	25	0x	25	0x
Nectariniidae	*Nectarinia rockefelleri*	T		x	5	29	24x	0	24x	40	24x	29	0x	29	24x	0	53x	40	24x	29	0x
Nectariniidae	*Nectarinia cuprea*				5	29	4	19	7	28	5	38	3	35	5	23	11	37	8	40	4
Nectariniidae	*Nectarinia fusca*				5	4	8	2	9	4	10	3	9	4	11	2	15	4	14	4	14
Nectariniidae	*Nectarinia rufipennis*	T		x	5	0	0x	0	0x	0	0x	0	0x	0	0x	0	0x	0	0x	0	0x
Nectariniidae	*Nectarinia tacazze*				5	35	1	27	2	36	0	36	0	36	1	31	5	37	0	36	0
Nectariniidae	*Nectarinia purpureiventris*				5	24	8	23	12	34	5	33	0	26	8	26	13	34	8	33	0
Nectariniidae	*Nectarinia bocagii*				14	52	5	36	9	45	5	50	9	52	5	39	9	45	5	50	9
Nectariniidae	*Nectarinia kilimensis*				5	31	2	26	4	33	1	34	1	32	3	29	6	34	2	34	1
Nectariniidae	*Nectarinia reichenowi*				5	35	1	32	4	37	1	37	1	37	1	33	4	37	1	37	1
Nectariniidae	*Nectarinia famosa*				5	8	5	7	6	9	6	9	5	8	7	8	8	10	8	10	7
Nectariniidae	*Nectarinia johnstoni*			x	5	28	14x	23	11x	39	3x	39	0x	28	14x	29	11x	39	3x	39	0x
Nectariniidae	*Nectarinia shelleyi*				5	48	0	44	1	48	0	48	0	48	0	44	5	48	0	48	0
Nectariniidae	*Nectarinia erythrocerca*				5	38	3	30	7	42	0	42	0	39	3	35	9	43	1	43	0
Nectariniidae	*Nectarinia congensis*			x	5	0	0x	100	0x	100	0x	100	0x	0	0x	100	0x	100	0x	100	0x
Nectariniidae	*Nectarinia mariquensis*				5	22	6	12	11	23	6	23	5	22	9	14	17	23	9	23	8
Nectariniidae	*Nectarinia bifasciata*				5	36	0	30	1	37	0	39	0	39	1	31	5	39	1	40	1
Nectariniidae	*Nectarinia tsavoensis*				14	24	3	16	12	27	0	27	0	24	3	16	12	28	0	28	0

Family	Species	IUCN	Ag	R	Range Source	2050 TG H	2050 TG C	2050 AM H	2050 AM C	2050 GO H	2050 GO C	2050 OS H	2050 OS C	2100 TG H	2100 TG C	2100 AM H	2100 AM C	2100 GO H	2100 GO C	2100 OS H	2100 OS C
Nectariniidae	*Nectarinia pembae*			x	5	0	0x	0	0x	0	0x	0	0x	0	0x	0	0x	0	0x	0	0x
Nectariniidae	*Nectarinia chalcomelas*				5	1	0	0	1	1	0	1	0	1	0	0	1	1	0	1	0
Nectariniidae	*Nectarinia notata*				6,13	49	0	27	5	50	0	50	0	50	0	37	5	50	0	50	0
Nectariniidae	*Nectarinia coccinigaster*				5	24	14	10	12	24	7	38	6	32	14	14	14	39	11	45	7
Nectariniidae	*Nectarinia johannae*				5	35	5	20	7	30	7	56	4	52	5	32	8	58	8	67	4
Nectariniidae	*Nectarinia superba*				5	37	5	21	8	33	6	52	3	51	6	30	9	56	7	60	4
Nectariniidae	*Nectarinia pulchella*				5	13	3	7	5	14	2	17	2	16	3	9	7	18	5	18	3
Nectariniidae	*Nectarinia nectarinioides*				5	21	0	10	11	21	0	21	0	21	0	10	11	21	0	21	0
Nectariniidae	*Aethopyga primigenius*				7	19	15	27	19	7	19	16	20	30	19	27	19	24	24	34	20x
Nectariniidae	*Aethopyga boltoni*				7	18	14	24	18	7	18	16	18	27	17	24	18	22	21	31	18
Nectariniidae	*Aethopyga flagrans*				7	9	9	23	6	14	10	15	7	9	19	35	12	24	21	26	8
Nectariniidae	*Aethopyga pulcherrima*				7	14	12	24	11	13	12	22	12	20	18	30	14	26	21	34	13
Nectariniidae	*Aethopyga duyvenbodei*	T		x	7	0	0x	0	0x	0	0x	0	0x	0	0x	0	0x	0	0x	0	0x
Nectariniidae	*Aethopyga shelleyi*				7	16	12	23	12	14	13	22	12	21	18	29	17	26	23	34	15
Nectariniidae	*Aethopyga gouldiae*				7	24	7	26	6	25	7	26	7	26	10	29	11	28	12	29	11
Nectariniidae	*Aethopyga nipalensis*		H		7	0	9	0	8	0	10	0	9	0	13	0	13	0	16	0	14
Nectariniidae	*Aethopyga eximia*				7	6	0	0	0	0	0	0	0	10	0	5	6	6	0	5	1
Nectariniidae	*Aethopyga christinae*				7	4	1	5	1	4	1	5	1	4	1	5	1	5	1	6	1
Nectariniidae	*Aethopyga saturata*				7	23	7	26	7	24	8	26	7	26	10	28	11	28	13	31	11
Nectariniidae	*Aethopyga siparaja*		H		7	0	4	0	4	0	5	0	4	0	7	0	7	0	8	0	6
Nectariniidae	*Aethopyga mystacalis*				7	13	2	16	3	15	3	18	2	20	3	22	3	28	3	28	2
Nectariniidae	*Aethopyga ignicauda*				7	47	7	51	6	49	7	52	4	52	10	55	11	54	14	56	9
Nectariniidae	*Arachnothera longirostra*		H		7	0	5	0	5	0	6	0	6	0	8	0	8	0	9	0	7
Nectariniidae	*Arachnothera crassirostris*				7	13	2	16	3	15	3	19	2	20	3	22	3	28	3	28	3
Nectariniidae	*Arachnothera robusta*				7	13	2	16	3	15	3	18	2	20	3	22	3	28	3	28	2
Nectariniidae	*Arachnothera flavigaster*				7	13	2	16	3	15	3	19	2	20	3	22	3	28	3	28	2
Nectariniidae	*Arachnothera chrysogenys*		H		7	0	3	0	3	0	3	0	3	0	4	0	4	0	3	0	3
Nectariniidae	*Arachnothera clarae*				7	13	11	19	12	11	12	15	13	16	17	22	16	22	19	24	15
Nectariniidae	*Arachnothera affinis*		H		7	0	3	0	4	0	4	0	4	0	4	0	5	0	4	0	4
Nectariniidae	*Arachnothera everetti*				7	10	0	10	2	8	2	12	0	15	0	12	2	19	2	19	0
Nectariniidae	*Arachnothera magna*				7	22	8	25	7	23	8	25	8	26	10	27	10	27	12	30	10
Nectariniidae	*Arachnothera juliae*				7	14	0	15	3	16	3	22	0	18	0	19	3	35	3	32	0
Melanocharitidae	*Melanocharis arfakiana*			x	8	7	0x	8	0x	2	0x	4	0x	7	3x	8	3x	3	3x	69	1x
Melanocharitidae	*Melanocharis nigra*				8	5	2	6	2	17	2	15	1	12	4	13	5	29	4	25	2
Melanocharitidae	*Melanocharis longicauda*				8	6	0	4	0	20	0	11	0	10	5	10	7	34	6	29	3
Melanocharitidae	*Melanocharis versteri*				8	6	0	3	0	20	0	12	0	10	6	11	8	34	7	31	3
Melanocharitidae	*Melanocharis striativentris*				8	6	0	3	0	21	0	10	0	10	6	10	7	34	6	28	3
Melanocharitidae	*Melanocharis crassirostris*				8	6	0	3	0	20	0	11	0	10	6	11	8	34	7	29	3
Melanocharitidae	*Toxorhamphus novaeguine*				8	5	2	6	2	15	2	12	1	11	3	14	4	24	3	21	1
Melanocharitidae	*Toxorhamphus poliopterus*				8	5	1	7	1	22	1	15	0	10	8	13	9	36	8	30	3
Melanocharitidae	*Toxorhamphus iliolophus*				8	5	2	6	1	17	1	15	1	11	4	13	5	27	4	25	2
Melanocharitidae	*Oedistoma pygmaeum*				8	4	4	6	3	16	2	15	1	10	5	13	5	26	4	24	2
Paramythiidae	*Oreocharis arfaki*				8	6	0	3	0	20	0	12	0	10	6	11	8	33	7	31	3
Paramythiidae	*Paramythia montium*				8	8	0	3	0	19	0	15	0	12	6	14	8	31	8	32	4

Family	Species	IUCN	Ag	R	Range Source	Percentage loss in geographic range 2050 TG H	2050 TG C	2050 AM H	2050 AM C	2050 GO H	2050 GO C	2050 OS H	2050 OS C	2100 TG H	2100 TG C	2100 AM H	2100 AM C	2100 GO H	2100 GO C	2100 OS H	2100 OS C
Passeridae	*Passer ammodendri*		H		4,7	0	11	0	11	0	11	0	11	0	13	0	20	0	24	0	22
Passeridae	*Passer domesticus*		H		,5,6,7,8,9,10,11,:	0	10	0	9	0	11	0	8	0	12	0	12	0	14	0	10
Passeridae	*Passer hispaniolensis*		H		3,4,5,7	0	8	0	8	0	8	0	7	0	9	0	11	0	13	0	11
Passeridae	*Passer pyrrhonotus*				4,7	4	2	5	2	6	1	5	1	5	5	5	5	6	7	7	1
Passeridae	*Passer castanopterus*				5	34	6	7	25	43	2	44	2	39	6	9	33	43	5	44	4
Passeridae	*Passer rutilans*		H		7	0	4	0	3	0	4	0	4	0	5	0	5	0	5	0	5
Passeridae	*Passer flaveolus*		H		7	0	8	0	8	0	9	0	9	0	14	0	14	0	18	0	14
Passeridae	*Passer moabiticus*			x	3,4	9	0x	33	0x	20	1x	20	0x	45	0x	44	1x	45	1x	45	1x
Passeridae	*Passer iagoensis*		H	x	14	0	7x	0	7x	0	0x	0	0x	0	7x	0	7x	0	0x	0	0x
Passeridae	*Passer rufocinctus*		H		5	0	7	0	12	0	6	0	6	0	8	0	12	0	6	0	6
Passeridae	*Passer shelleyi*				14	59	6	21	24	66	0	66	0	60	6	28	27	67	0	67	0
Passeridae	*Passer cordofanicus*				14	5	0	0	0	10	0	10	0	10	0	3	3	10	3	10	3
Passeridae	*Passer insularis*			x	4,5	0	0x	0	0x	0	0x	0	0x	0	0x	0	0x	0	0x	0	0x
Passeridae	*Passer motitensis*		H		5	0	11	0	14	0	12	0	12	0	16	0	21	0	18	0	18
Passeridae	*Passer melanurus*		H		5	0	5	0	6	0	7	0	6	0	7	0	10	0	11	0	10
Passeridae	*Passer griseus*				5	24	3	15	5	25	4	34	2	32	4	20	8	36	6	37	4
Passeridae	*Passer swainsonii*				5	29	0	17	3	31	0	31	0	31	0	21	7	31	2	31	1
Passeridae	*Passer gongonensis*				5	41	6	15	27	47	1	47	1	42	6	17	29	47	2	47	2
Passeridae	*Passer suahelicus*				5	41	6	36	10	45	5	45	5	43	7	44	10	55	5	51	7
Passeridae	*Passer diffusus*				5	17	3	13	5	16	4	17	3	17	6	14	11	17	6	17	6
Passeridae	*Passer simplex*				3,4,5	0	2	0	2	0	2	1	2	0	2	1	2	1	3	1	2
Passeridae	*Passer montanus*		H		2,3,4,5,7,8,10,11	0	11	0	11	0	12	0	10	0	14	0	15	0	17	0	14
Passeridae	*Passer luteus*				4,5	10	5	4	5	9	5	16	3	14	5	6	10	16	10	17	7
Passeridae	*Passer euchlorus*				4,5	0	0	0	0	0	0	0	0	0	0	0	0	0	0	0	0
Passeridae	*Passer eminibey*				5	41	4	27	13	45	2	45	2	42	5	32	14	49	2	48	2
Passeridae	*Petronia pyrgita*				5	26	4	10	14	28	3	31	2	28	5	12	16	30	6	31	4
Passeridae	*Petronia xanthocollis*				4,7	11	3	12	3	13	2	17	1	15	4	15	6	16	7	25	2
Passeridae	*Petronia superciliaris*				3,5	40	2	29	2	39	2	43	1	43	3	31	7	42	3	44	2
Passeridae	*Petronia dentata*				4,5	19	3	9	4	22	3	28	2	27	3	13	7	29	5	29	3
Passeridae	*Petronia petronia*				3,4,5,7	10	10	11	10	11	11	13	10	13	13	13	15	16	15	18	13
Passeridae	*Carpospiza brachydactyla*				3,4,5	7	3	6	2	7	2	11	2	13	3	9	2	14	3	15	2
Passeridae	*Montifringilla nivalis*				3,4,7	13	16	14	16	14	16	15	15	15	25	17	31	21	32	22	29
Passeridae	*Montifringilla adamsi*				7	17	20	18	19	19	19	19	18	19	32	23	40	28	40	28	36
Passeridae	*Montifringilla taczanowskii*				7	16	14	18	14	18	14	19	13	19	26	23	37	28	37	28	33
Passeridae	*Montifringilla davidiana*				7	11	6	11	7	11	8	11	7	11	8	11	12	12	13	13	12
Passeridae	*Montifringilla ruficollis*				7	17	18	19	17	19	17	19	16	19	29	23	37	28	37	28	33
Passeridae	*Montifringilla blanfordi*				7	15	16	16	16	16	16	17	15	16	27	20	36	25	37	25	33
Passeridae	*Montifringilla theresae*		H		14	0	0	0	0	0	0	0	0	0	0	0	0	0	0	0	0
Passeridae	*Dendronanthus indicus*				4,7	16	3	15	4	15	4	15	4	16	4	16	5	16	5	17	5
Passeridae	*Motacilla alba*		H		2,3,4,5,7	0	17	0	16	0	18	0	15	0	21	0	22	0	25	0	21
Passeridae	*Motacilla lugens*		H		7	0	5	0	5	0	6	0	5	0	9	0	12	0	11	0	10
Passeridae	*Motacilla grandis*		H		7	0	20	0	19	0	20	0	20	0	32	0	38	0	35	0	35
Passeridae	*Motacilla madaraspatensis*				7	11	2	12	2	12	2	15	1	12	4	13	7	13	8	21	2
Passeridae	*Motacilla aguimp*		H		5	0	2	0	5	0	2	0	2	0	3	0	8	0	3	0	3

Family	Species	IUCN	Ag	R	Range Source	Percentage loss in geographic range 2050 TG H	C	AM H	C	GO H	C	OS H	C	2100 TG H	C	AM H	C	GO H	C	OS H	C
Passeridae	*Motacilla capensis*		H		5	0	4	0	6	0	5	0	5	0	6	0	11	0	8	0	7
Passeridae	*Motacilla flaviventris*				6	48	0	30	4	48	0	48	0	48	0	38	4	48	0	48	0
Passeridae	*Motacilla citreola*				3,4,5,7	7	15	7	15	8	16	8	14	9	20	9	22	11	24	12	21
Passeridae	*Motacilla flava*		H		2,3,4,5,7,8,10,11	0	10	0	11	0	11	0	9	0	13	0	16	0	16	0	13
Passeridae	*Motacilla cinerea*				2,3,4,5,7,8	4	9	5	9	5	10	4	10	6	12	6	11	7	13	7	12
Passeridae	*Motacilla clara*				5	42	4	31	5	41	5	52	3	50	6	36	8	51	5	55	3
Passeridae	*Tmetothylacus tenellus*				4,5	27	2	11	15	30	0	30	0	28	2	14	16	30	2	30	1
Passeridae	*Macronyx croceus*				5	22	2	16	3	25	2	28	1	27	3	19	6	30	3	30	2
Passeridae	*Macronyx fuellebornii*				5	50	4	34	5	50	3	57	2	54	5	37	9	55	4	58	2
Passeridae	*Macronyx capensis*				5	7	1	7	1	6	2	7	1	7	2	7	2	7	3	7	2
Passeridae	*Macronyx flavicollis*				5	32	0	20	2	33	0	33	0	33	0	25	3	33	0	33	0
Passeridae	*Macronyx sharpei*	T			5	40	0	33	6	40	0	40	0	40	0	34	6	40	0	40	0
Passeridae	*Macronyx ameliae*				5	24	1	18	3	24	1	25	1	24	1	20	8	26	1	26	1
Passeridae	*Macronyx aurantiigula*				5	30	4	26	6	33	2	32	3	31	5	30	6	35	2	34	3
Passeridae	*Macronyx grimwoodi*				5	28	0	13	0	27	0	28	0	28	0	14	2	28	0	29	0
Passeridae	*Anthus chloris*	T		x	5	18	0x	18	0x	18	0x	18	0x	18	0x	18	0x	18	0x	18	0x
Passeridae	*Anthus lineiventris*				5	36	1	31	2	36	2	36	1	36	3	33	7	36	3	36	3
Passeridae	*Anthus crenatus*				5	6	3	6	3	5	4	6	3	6	3	6	3	6	4	6	4
Passeridae	*Anthus hoeschi*				5	7	0	7	0	7	0	7	0	7	0	7	0	7	0	7	0
Passeridae	*Anthus richardi*				4,5,7,8	12	7	9	8	12	7	12	7	13	8	10	11	13	11	14	9
Passeridae	*Anthus rufulus*				7,8	13	4	15	4	14	4	17	4	15	7	17	8	17	11	22	5
Passeridae	*Anthus novaeseelandiae*				8,10,12,13	0	12	0	9	1	11	1	7	1	13	0	11	1	13	1	8
Passeridae	*Anthus leucophrys*				5	19	2	10	5	21	2	24	1	23	3	13	8	25	3	25	2
Passeridae	*Anthus vaalensis*		H		5	0	3	0	5	0	4	0	4	0	6	0	10	0	6	0	6
Passeridae	*Anthus pallidiventris*		H		5	0	4	0	5	0	6	0	1	0	4	0	5	0	6	0	1
Passeridae	*Anthus melindae*				5	2	0	2	0	2	0	2	0	2	0	2	0	2	0	2	0
Passeridae	*Anthus campestris*		H		3,4,5,7	0	11	0	10	0	13	0	10	0	14	0	16	0	19	0	15
Passeridae	*Anthus berthelotii*		H	x	3	0	10x	0	10x	0	10x	0	10x	0	10x	0	10x	0	15x	0	12x
Passeridae	*Anthus godlewskii*				4,7	2	13	2	13	2	16	2	13	3	16	3	17	4	22	8	18
Passeridae	*Anthus similis*				3,4,5,7	16	4	11	6	17	3	19	3	19	5	13	10	19	7	21	4
Passeridae	*Anthus brachyurus*				5	26	2	18	3	24	3	32	1	30	2	21	5	30	3	32	1
Passeridae	*Anthus caffer*				5	25	7	21	10	27	7	27	7	26	9	23	13	29	9	28	9
Passeridae	*Anthus sokokensis*	T		x	5	0	0x	0	0x	0	0x	0	0x	0	0x	0	0x	0	0x	0	0x
Passeridae	*Anthus trivialis*				3,4,5,7	5	18	4	16	6	19	5	15	7	21	6	20	11	23	11	18
Passeridae	*Anthus hodgsoni*				3,4,7	7	9	7	9	7	9	7	9	8	11	7	13	8	13	9	13
Passeridae	*Anthus gustavi*				3,4,7	0	24	0	24	0	24	0	24	0	29	0	37	0	40	0	37
Passeridae	*Anthus pratensis*				3,4,5	6	26	5	21	10	26	7	20	10	31	9	26	15	30	12	24
Passeridae	*Anthus cervinus*				2,3,4,5,7	0	26	0	26	0	26	0	26	0	34	0	41	0	42	0	41
Passeridae	*Anthus roseatus*				7	14	13	15	13	15	13	15	13	15	21	17	28	22	28	22	25
Passeridae	*Anthus petrosus*				3,4	6	30	10	24	13	26	10	22	9	35	12	33	22	34	15	28
Passeridae	*Anthus spinoletta*				3,4,5,7	7	13	7	14	9	14	9	13	10	18	10	21	16	21	17	19
Passeridae	*Anthus rubescens*				2,7	2	18	2	18	2	18	2	17	2	25	2	33	2	35	2	32
Passeridae	*Anthus sylvanus*				7	18	2	19	2	19	1	20	1	19	3	20	3	21	2	22	2
Passeridae	*Anthus nilghiriensis*			x	7	20	0x	28	0x	21	0x	32	0x	25	1x	31	0x	23	0x	40	0x

Family	Species	IUCN	Ag	R	Range Source	2050 TG H	2050 TG C	2050 AM H	2050 AM C	2050 GO H	2050 GO C	2050 OS H	2050 OS C	2100 TG H	2100 TG C	2100 AM H	2100 AM C	2100 GO H	2100 GO C	2100 OS H	2100 OS C
						Percentage loss in geographic range															
Passeridae	*Anthus correndera*				2	4	6	3	7	4	7	5	6	6	8	6	11	8	12	9	10
Passeridae	*Anthus spragueii*	T			2	2	19	3	14	4	18	3	10	3	21	4	16	5	20	4	12
Passeridae	*Anthus furcatus*				2	4	7	4	7	4	8	5	7	6	9	5	11	7	13	8	10
Passeridae	*Anthus hellmayri*				2	9	6	8	5	11	5	14	4	13	9	13	10	18	9	21	8
Passeridae	*Anthus bogotensis*				2	11	5	12	8	7	7	13	6	12	8	18	12	21	11	18	10
Passeridae	*Anthus lutescens*		H		2	0	12	0	8	0	9	0	6	0	17	0	12	0	13	0	10
Passeridae	*Anthus chacoensis*				2	2	2	1	1	1	0	1	1	2	3	1	1	2	1	2	1
Passeridae	*Anthus nattereri*	T			2	19	6	14	3	26	2	25	4	24	10	27	8	38	3	41	4
Passeridae	*Anthus gutturalis*			x	8	6	0x	4	0x	17	0x	19	0x	15	3x	13	7x	30	7x	30	6x
Passeridae	*Prunella collaris*				3,4,7	12	14	13	14	13	14	13	13	14	21	15	25	18	25	19	23
Passeridae	*Prunella himalayana*				7	8	18	9	18	9	18	9	17	10	23	10	27	12	28	14	25
Passeridae	*Prunella rubeculoides*				7	17	19	19	19	19	19	20	17	20	32	24	41	28	40	29	36
Passeridae	*Prunella strophiata*				7	44	3	46	3	46	3	47	2	46	6	49	6	49	5	50	5
Passeridae	*Prunella montanella*				3,4,7	1	14	0	14	0	14	0	14	2	16	1	19	2	19	2	19
Passeridae	*Prunella ocularis*				3,4	20	5	23	4	30	5	42	4	47	5	34	4	46	5	50	4
Passeridae	*Prunella fagani*				4	0	0	0	0	0	0	0	0	0	0	0	0	0	10	0	10
Passeridae	*Prunella fulvescens*				7	11	15	13	15	13	15	13	15	13	24	15	30	20	29	20	27
Passeridae	*Prunella atrogularis*				3,4,7	9	18	3	22	3	22	2	21	11	19	5	25	13	24	13	24
Passeridae	*Prunella koslowi*				7	0	4	0	5	0	6	0	5	0	6	0	7	0	8	0	8
Passeridae	*Prunella modularis*				3,4,5,12,13	7	27	7	24	10	27	9	22	11	33	10	30	18	33	16	27
Passeridae	*Prunella rubida*				7	8	18	7	17	7	20	6	19	9	26	9	33	10	33	16	30
Passeridae	*Prunella immaculata*				7	46	4	48	3	47	4	46	4	47	8	52	11	52	9	50	9
Passeridae	*Bubalornis albirostris*				5	16	3	8	6	18	3	21	2	21	3	11	9	22	6	22	4
Passeridae	*Bubalornis niger*				5	27	4	12	15	30	3	30	3	28	6	14	20	30	6	30	5
Passeridae	*Dinemellia dinemelli*				5	39	4	15	20	45	1	45	1	42	4	19	24	46	3	45	2
Passeridae	*Sporopipes frontalis*				5	18	3	11	5	20	3	24	2	23	3	13	9	25	6	25	4
Passeridae	*Sporopipes squamifrons*				5	8	5	3	7	7	5	7	5	8	8	3	13	7	11	7	9
Passeridae	*Plocepasser mahali*				5	22	3	13	9	23	3	23	3	23	5	15	14	23	6	23	5
Passeridae	*Plocepasser superciliosus*				5	12	1	7	4	14	1	14	1	14	1	9	5	15	2	14	1
Passeridae	*Plocepasser rufoscapulatu*				5	51	0	41	0	51	0	52	0	52	0	43	5	51	0	52	0
Passeridae	*Plocepasser donaldsoni*				5	66	14	8	64	83	3	83	3	72	14	10	72	83	3	83	3
Passeridae	*Histurgops ruficauda*				5	39	12	37	18	48	10	46	12	43	15	46	18	64	10	56	15
Passeridae	*Pseudonigrita arnaudi*				5	34	4	27	7	37	3	37	3	36	5	34	8	44	3	42	4
Passeridae	*Pseudonigrita cabanisi*				5	53	7	14	37	62	1	62	1	56	7	19	39	64	1	64	1
Passeridae	*Philetairus socius*				5	2	6	1	7	2	6	2	6	2	8	1	9	2	8	2	8
Passeridae	*Ploceus bannermani*	T		x	5	22	2x	2	1x	18	10x	14	9x	38	2x	21	2x	45	10x	14	9x
Passeridae	*Ploceus batesi*	T		x	5	13	17x	0	17x	38	14x	57	14x	22	27x	9	27x	59	14x	72	14x
Passeridae	*Ploceus nigrimentum*				5	28	3x	26	0	31	3x	37	0x	30	3x	33	0x	33	3x	37	0x
Passeridae	*Ploceus baglafecht*				5	34	3	23	6	36	2	39	1	38	3	28	9	41	3	40	2
Passeridae	*Ploceus bertrandi*				5	53	0	44	0	53	0	53	0	53	2	50	5	53	0	53	0
Passeridae	*Ploceus pelzelni*				5	38	3	26	6	37	0	45	0	42	4	31	7	46	1	47	0
Passeridae	*Ploceus subpersonatus*	T		x	5	9	12x	9	12x	19	0x	9	12x	9	12x	9	12x	19	0x	9	12x
Passeridae	*Ploceus luteolus*				5	22	2	12	5	24	2	26	1	26	2	15	7	27	2	27	2
Passeridae	*Ploceus intermedius*				4,5	34	3	23	10	36	2	36	2	35	5	25	14	37	4	37	3

Family	Species	IUCN	Ag	R	Range Source	2050 TG H	2050 TG C	2050 AM H	2050 AM C	2050 GO H	2050 GO C	2050 OS H	2050 OS C	2100 TG H	2100 TG C	2100 AM H	2100 AM C	2100 GO H	2100 GO C	2100 OS H	2100 OS C
Passeridae	*Ploceus ocularis*				5	38	2	30	4	39	2	42	1	41	3	33	8	42	3	43	2
Passeridae	*Ploceus nigricollis*				5	35	4	21	6	33	5	47	3	45	5	27	9	48	6	51	4
Passeridae	*Ploceus melanogaster*				5	30	8	20	14	41	3	39	0	32	11	26	17	44	4	42	0
Passeridae	*Ploceus alienus*				5	31	11	28	16	41	6	40	0	31	11	33	16	41	10	40	0
Passeridae	*Ploceus temporalis*				5	79	6	61	7	90	3	90	4	87	6	61	7	91	3	93	4
Passeridae	*Ploceus capensis*				5	3	3	2	3	2	4	2	3	3	4	2	5	3	6	2	6
Passeridae	*Ploceus subaureus*				5	83	0x	71	0x	83	0x	83	0x	83	1x	77	0x	84	0x	84	0x
Passeridae	*Ploceus xanthops*				5	37	1	31	3	37	1	38	1	37	2	33	6	38	1	38	1
Passeridae	*Ploceus princeps*			x	5	0	0x	0	0x	0	0x	0	0x	0	0x	0	0x	0	0x	0	0x
Passeridae	*Ploceus aurantius*				5	37	8	22	12	32	11	50	4	48	8	31	14	50	12	57	6
Passeridae	*Ploceus bojeri*				5	18	0	5	13	18	0	18	0	18	0	5	13	18	0	18	0
Passeridae	*Ploceus castaneiceps*				5	45	6	35	6	52	0	52	0	45	6	45	6	52	0	52	0
Passeridae	*Ploceus xanthopterus*				5	30	2	25	3	30	1	30	2	30	4	27	12	31	3	31	2
Passeridae	*Ploceus castanops*				5	26	4	20	5	30	0	31	0	26	4	23	8	30	1	31	0
Passeridae	*Ploceus burnieri*	T		x	5	13	0x	13	0x	13	0x	13	0x	13	0x	13	0x	13	0x	13	0x
Passeridae	*Ploceus galbula*				4,5	7	2	4	4	7	2	7	2	7	2	4	5	7	3	7	3
Passeridae	*Ploceus heuglini*				5	16	2	8	4	20	2	23	2	22	2	11	6	26	5	25	3
Passeridae	*Ploceus taeniopterus*				5	12	3	10	8	16	0	16	0	15	3	10	10	19	0	19	0
Passeridae	*Ploceus vitellinus*				5	22	3	11	8	24	2	28	1	26	3	13	10	29	4	29	2
Passeridae	*Ploceus velatus*				5	14	4	10	6	13	5	13	5	14	7	10	12	13	8	14	8
Passeridae	*Ploceus katangae*			x	5	62	0x	61	0x	62	0x	62	0x	62	0x	61	0x	62	0x	62	0x
Passeridae	*Ploceus ruweti*			x	5	75	0x	0	0x	75	0x	75	0x	75	0x	0	0x	75	0x	75	0x
Passeridae	*Ploceus reichardi*			x	5	33	0x	26	0x	33	0x	33	0x	33	0x	30	0x	33	0x	33	0x
Passeridae	*Ploceus cucullatus*				2,5,13	32	3	21	5	33	4	42	2	39	4	26	9	43	5	44	3
Passeridae	*Ploceus grandis*			x	5	0	⊦02x	0	⊦02x	100	0x	100	0x	0	⊦02x	0	⊦02x	100	0x	100	0x
Passeridae	*Ploceus spekei*				5	29	4	21	5	31	3	31	3	30	5	26	6	34	3	33	3
Passeridae	*Ploceus spekeoides*			x	5	49	0x	28	2x	49	0x	49	0x	49	0x	47	2x	49	0x	49	0x
Passeridae	*Ploceus nigerrimus*				5	37	6	22	8	33	7	50	3	46	7	30	11	52	8	55	4
Passeridae	*Ploceus weynsi*				5	44	5	45	12	49	3	48	0	54	5	50	14	55	3	50	0
Passeridae	*Ploceus golandi*	T		x	5	0	0x	0	0x	0	0x	0	0x	0	0x	0	0x	0	0x	0	0x
Passeridae	*Ploceus melanocephalus*				5	21	2	14	4	22	1	26	0	26	2	17	6	30	3	28	2
Passeridae	*Ploceus dicrocephalus*				5	23	0	9	12	27	0	27	0	27	0	12	15	27	0	27	0
Passeridae	*Ploceus jacksoni*				5	45	2	34	8	47	0	47	1	45	3	40	8	49	0	48	1
Passeridae	*Ploceus badius*				5	13	1	6	5	16	0	16	0	16	1	8	8	18	1	18	0
Passeridae	*Ploceus rubiginosus*				5	42	6	18	22	48	3	48	3	46	7	22	27	49	3	49	4
Passeridae	*Ploceus aureonucha*	T		x	5	72	25x	54	25x	42	37x	59	37x	72	25x	72	25x	59	37x	59	37x
Passeridae	*Ploceus tricolor*				5	28	7	20	8	32	6	47	4	43	8	31	9	50	6	56	4
Passeridae	*Ploceus albinucha*				5	30	7	21	9	34	7	47	3	44	8	29	11	46	8	55	3
Passeridae	*Ploceus nelicourvi*				6	76	0	47	5	79	0	79	0	79	0	71	5	79	0	79	0
Passeridae	*Ploceus sakalava*				6	41	0	8	11	41	0	41	0	41	0	16	11	41	0	41	0
Passeridae	*Ploceus benghalensis*		H		7	0	3	0	3	0	3	0	1	0	5	0	7	0	8	0	2
Passeridae	*Ploceus manyar*		H		5,7	0	4	0	4	0	4	0	4	0	7	0	9	0	11	0	5
Passeridae	*Ploceus philippinus*		H		7	0	5	0	5	0	5	0	5	0	8	0	9	0	11	0	6
Passeridae	*Ploceus hypoxanthus*		H		7	0	3	0	4	0	3	0	4	0	9	0	9	0	12	0	8

Family	Species	IUCN	Ag	R	Range Source	2050 TG H	2050 TG C	2050 AM H	2050 AM C	2050 GO H	2050 GO C	2050 OS H	2050 OS C	2100 TG H	2100 TG C	2100 AM H	2100 AM C	2100 GO H	2100 GO C	2100 OS H	2100 OS C
						Percentage loss in geographic range															
Passeridae	*Ploceus megarhynchus*	T			7	11	0	10	0	10	0	10	0	11	0	11	3	11	3	12	0
Passeridae	*Ploceus bicolor*				5	40	3	31	3	38	3	46	2	44	4	35	5	45	3	48	2
Passeridae	*Ploceus preussi*				5	32	6	21	7	36	6	52	4	47	7	36	8	57	7	63	4
Passeridae	*Ploceus dorsomaculatus*				5	31	7	24	14	35	17	51	7	47	7	41	15	52	20	58	8
Passeridae	*Ploceus nicolli*	T			5	17	0x	7	0	17	0x	17	0x	17	0x	17	0x	17	0x	17	0x
Passeridae	*Ploceus olivaceiceps*				5	56	0	45	0	56	0	56	0	56	2	50	1	56	0	56	0
Passeridae	*Ploceus insignis*				5	26	7	21	9	33	3	34	0	28	8	26	10	38	4	35	0
Passeridae	*Ploceus angolensis*				5	57	0	45	0	57	0	58	0	58	0	47	4	57	0	58	0
Passeridae	*Ploceus sanctithomae*			x	5	0⊦02	x	0⊦02	x	100	0x	100	0x	0⊦02	x	0⊦02	x	100	0x	100	0x
Passeridae	*Pachyphantes superciliosu*				5	40	4	26	6	42	3	45	2	44	4	34	10	46	4	47	4
Passeridae	*Malimbus flavipes*	T		x	5	60	0x	60	0x	60	0x	60	0x	60	0x	60	0x	60	0x	60	0x
Passeridae	*Malimbus coronatus*				5	36	3	27	6	39	5	61	6	58	3	46	6	67	5	73	6
Passeridae	*Malimbus cassini*				5	32	4	16	7	26	5	54	3	50	4	28	8	57	6	65	3
Passeridae	*Malimbus ballmanni*	T		x	5	33	5x	35	0x	30	5x	38	0x	33	5x	38	0x	33	5x	38	0x
Passeridae	*Malimbus racheliae*				5	13	14	20	11	17	3	36	3	33	14	28	11	34	3	42	3
Passeridae	*Malimbus scutatus*		H		5	0	5	0	6	0	6	0	3	0	6	0	8	0	7	0	4
Passeridae	*Malimbus ibadanensis*	T	H	x	5	0	0x	0	0x	0	0x	0	0x	0	0x	0	0x	0	0x	0	0x
Passeridae	*Malimbus erythrogaster*				5	35	4	24	7	33	6	61	3	55	4	33	7	62	6	70	3
Passeridae	*Malimbus nitens*				5	31	5	20	7	30	6	49	3	46	6	30	8	53	7	56	4
Passeridae	*Malimbus malimbicus*				5	35	5	21	8	33	6	56	4	52	6	31	9	59	7	63	4
Passeridae	*Malimbus rubricollis*				5	35	5	24	7	36	5	55	3	51	5	35	8	61	6	63	4
Passeridae	*Anaplectes rubriceps*				5	34	1	25	3	35	1	38	1	37	2	28	7	38	2	38	2
Passeridae	*Brachycope anomala*				5	44	12	33	20	39	15	62	7	68	12	47	21	69	16	75	8
Passeridae	*Quelea cardinalis*				5	41	4	30	10	44	2	43	3	42	4	36	11	47	2	46	3
Passeridae	*Quelea erythrops*				5	27	3	18	4	28	3	33	3	32	3	22	6	35	5	34	3
Passeridae	*Quelea quelea*				5	18	3	10	7	19	3	21	2	20	4	12	11	21	5	21	4
Passeridae	*Foudia madagascariensis*				4,6,13	49	0	27	5	50	0	50	0	50	0	37	5	50	0	50	0
Passeridae	*Foudia eminentissima*			x	13	30	0x	11	0x	30	0x	30	0x	30	0x	30	0x	30	0x	30	0x
Passeridae	*Foudia omissa*				6	75	0	45	4	78	0	78	0	78	0	71	4	78	0	78	0
Passeridae	*Foudia rubra*	T		x	13	0	0x	0	0x	0	0x	0	0x	0	0x	0	0x	0	0x	0	0x
Passeridae	*Euplectes afer*				2,5	18	4	12	5	20	4	22	3	22	5	15	8	25	5	24	5
Passeridae	*Euplectes diadematus*		H		5	0	3	0	4	0	0	0	0	0	3	0	4	0	0	0	0
Passeridae	*Euplectes gierowii*				5	53	1	42	1	52	0	54	0	53	1	46	1	56	1	54	0
Passeridae	*Euplectes hordeaceus*				5	35	3	26	4	35	2	38	2	38	4	30	7	40	3	40	2
Passeridae	*Euplectes franciscanus*				2,5	20	3	11	4	23	2	27	2	26	3	15	7	29	4	28	3
Passeridae	*Euplectes orix*				5	17	3	14	5	17	4	17	3	17	5	15	9	18	5	18	5
Passeridae	*Euplectes nigroventris*			x	5	8	0x	8	0x	8	0x	8	0x	8	0x	8	0x	8	0x	8	0x
Passeridae	*Euplectes aureus*				5	36	0	35	0	36	0	36	0	36	0	35	0	36	0	36	0
Passeridae	*Euplectes capensis*				5	32	1	26	3	33	1	34	1	33	1	29	4	34	1	34	1
Passeridae	*Euplectes axillaris*				5	30	2	23	4	32	1	33	1	32	3	26	8	34	2	34	2
Passeridae	*Euplectes macrourus*				5	27	2	19	3	30	2	36	1	33	2	22	6	37	3	38	2
Passeridae	*Euplectes albonotatus*				5	26	2	22	4	26	2	27	2	26	4	24	8	27	3	27	3
Passeridae	*Euplectes ardens*				5	32	2	25	3	34	1	37	1	35	2	29	5	36	2	37	2
Passeridae	*Euplectes hartlaubi*				5	45	1	32	1	43	0	46	0	48	1	37	3	49	0	48	0

Family	Species	IUCN	Ag	R	Range Source	Percentage loss in geographic range 2050 TG H	2050 TG C	2050 AM H	2050 AM C	2050 GO H	2050 GO C	2050 OS H	2050 OS C	2100 TG H	2100 TG C	2100 AM H	2100 AM C	2100 GO H	2100 GO C	2100 OS H	2100 OS C
Passeridae	*Euplectes psammocromius*			x	5	38	0x	38	0x	38	0x	38	0x	38	0x	38	11x	38	0x	38	0x
Passeridae	*Euplectes progne*				5	10	0	8	1	9	1	10	0	10	2	8	3	10	2	10	2
Passeridae	*Euplectes jacksoni*				5	34	2	30	7	35	2	35	2	35	2	30	7	38	2	35	2
Passeridae	*Anomalospiza imberbis*				5	26	1	21	3	27	1	29	1	27	2	23	7	29	2	29	1
Passeridae	*Amblyospiza albifrons*				5	38	2	28	5	37	3	43	2	43	3	33	8	43	4	45	2
Passeridae	*Parmoptila rubrifrons*			x	5	20	0x	16	0x	27	0x	37	0x	30	4x	28	4x	34	0x	37	0x
Passeridae	*Parmoptila jamesoni*				14	47	3	31	7	45	8	54	6	64	3	42	7	62	8	61	6
Passeridae	*Parmoptila woodhousei*				5	32	6	15	6	34	7	59	3	49	6	33	8	59	9	72	3
Passeridae	*Nigrita fusconota*				5	34	6	22	7	32	6	54	4	49	6	33	9	58	7	61	4
Passeridae	*Nigrita bicolor*				5	35	6	22	8	33	7	56	4	52	7	33	10	60	8	64	5
Passeridae	*Nigrita luteifrons*				5	35	5	23	8	33	6	57	4	53	6	34	9	61	7	65	4
Passeridae	*Nigrita canicapilla*				5	35	6	23	8	33	7	52	4	48	6	31	11	54	8	58	5
Passeridae	*Nesocharis shelleyi*				5	18	11	6	12	29	10	26	10	25	15	16	16	39	10	30	10
Passeridae	*Nesocharis ansorgei*				5	22	10	19	12	31	3	35	0	24	10	20	20	31	6	35	0
Passeridae	*Nesocharis capistrata*				5	29	3	20	6	33	4	44	2	43	4	26	12	46	11	46	5
Passeridae	*Pytilia phoenicoptera*				5	12	4	9	4	17	3	23	1	18	4	11	6	23	4	24	2
Passeridae	*Pytilia lineata*				5	43	0	24	2	48	0	48	0	48	0	34	5	48	1	48	1
Passeridae	*Pytilia afra*				5	45	2	34	3	43	3	51	1	50	3	37	7	49	3	52	2
Passeridae	*Pytilia melba*				4,5	18	3	10	7	19	2	20	2	19	4	12	11	21	4	20	3
Passeridae	*Pytilia hypogrammica*				5	23	6	17	5	26	5	41	3	33	6	25	11	43	13	46	8
Passeridae	*Mandingoa nitidula*				5	39	3	29	5	38	4	48	3	47	4	34	6	48	5	51	3
Passeridae	*Cryptospiza reichenovii*				5	39	3	35	4	47	1	44	0	42	6	38	7	47	2	46	0
Passeridae	*Cryptospiza salvadorii*				5	24	5	19	7	24	4	26	0	24	5	21	8	25	5	26	0
Passeridae	*Cryptospiza jacksoni*				5	18	6	14	8	22	5	22	0	18	6	17	8	23	8	22	0
Passeridae	*Cryptospiza shelleyi*	T			5	26	10x	18	14x	33	9x	30	0x	27	10x	23	15x	33	10x	30	0x
Passeridae	*Pyrenestes sanguineus*				5	32	5	15	7	23	7	44	6	38	6	24	12	41	12	44	9
Passeridae	*Pyrenestes ostrinus*				5	37	6	24	7	35	7	55	4	51	6	32	11	56	8	60	5
Passeridae	*Pyrenestes minor*				5	57	0	47	1	57	0	57	0	57	1	50	2	57	0	57	0
Passeridae	*Spermophaga poliogenys*				5	39	5	29	9	38	7	58	7	60	5	34	9	60	7	66	7
Passeridae	*Spermophaga haematina*				5	31	7	19	8	29	7	49	4	47	7	30	11	55	9	58	6
Passeridae	*Spermophaga ruficapilla*				5	41	4	32	8	38	6	57	5	57	4	36	8	53	6	62	5
Passeridae	*Clytospiza monteiri*				5	31	4	19	8	26	7	45	3	42	5	24	9	44	7	49	4
Passeridae	*Hypargos niveoguttatus*				5	46	0	39	0	46	0	49	0	48	1	41	4	49	0	49	0
Passeridae	*Hypargos margaritatus*				5	6	1	6	1	6	1	6	1	6	4	6	6	8	1	8	1
Passeridae	*Euschistospiza dybowskii*				5	30	7	20	10	33	10	54	4	45	9	25	19	52	18	56	9
Passeridae	*Euschistospiza cinereovina*				5	23	4	16	4	27	1	31	0	23	4	22	8	27	5	31	0
Passeridae	*Lagonosticta rufopicta*				5	27	3	15	4	32	3	39	2	36	3	21	8	42	4	41	3
Passeridae	*Lagonosticta nitidula*				14	20	2	14	3	19	2	24	2	22	2	14	10	22	3	24	2
Passeridae	*Lagonosticta senegala*		H		5	0	2	0	4	0	2	0	2	0	3	0	7	0	4	0	3
Passeridae	*Lagonosticta rara*		H		5	0	3	0	6	0	4	0	2	0	3	0	8	0	5	0	3
Passeridae	*Lagonosticta rubricata*				5	36	2	26	4	36	2	40	2	39	3	30	7	40	3	41	2
Passeridae	*Lagonosticta virata*				5	15	4	9	5	11	5	25	3	20	4	13	5	25	5	25	3
Passeridae	*Lagonosticta umbrinodorsa*			x	5	28	0x	0	0x	9	0x	63	0x	78	0x	32	0x	45	0x	63	0x
Passeridae	*Lagonosticta rhodopareia*				5	29	2	21	5	29	2	30	2	29	3	23	10	30	3	30	3

Family	***Species***	***IUCN***	***Ag***	***R***	***Range Source***	***Percentage loss in geographic range***															
						2050								***2100***							
						TG		***AM***		***GO***		***OS***		***TG***		***AM***		***GO***		***OS***	
						H	***C***	***H***	***C***	***H***	***C***	***H***	***C***	***H***	***C***	***H***	***C***	***H***	***C***	***H***	***C***
Passeridae	*Lagonosticta larvata*				5	20	3	11	4	23	2	29	2	28	3	14	6	30	4	30	3
Passeridae	*Lagonosticta sanguinodors*				14	3	0	14	0	19	0	19	0	17	0	17	0	19	0	19	0
Passeridae	*Uraeginthus angolensis*				5	22	3	18	4	22	3	22	2	22	5	19	9	22	4	23	4
Passeridae	*Uraeginthus bengalus*				5,11	24	3	14	5	26	3	33	2	30	4	18	8	33	5	34	3
Passeridae	*Uraeginthus cyanocephalu*				5	40	4	23	18	44	2	44	3	43	4	28	19	48	2	46	3
Passeridae	*Uraeginthus ianthinogaster*				5	42	5	16	22	50	1	50	1	46	5	21	27	50	3	50	2
Passeridae	*Uraeginthus granatina*				5	10	5	4	7	9	5	10	5	10	8	4	15	9	10	10	9
Passeridae	*Estrilda caerulescens*				5,11	7	2	4	2	10	2	15	1	13	2	7	4	15	3	16	2
Passeridae	*Estrilda perreini*				5	51	1	43	0	52	0	59	0	57	1	46	1	57	0	60	0
Passeridae	*Estrilda thomensis*				5	28	0	14	2	28	0	28	0	28	0	21	2	28	0	28	0
Passeridae	*Estrilda quartinia*				5	37	2	29	3	40	1	41	1	39	2	32	6	41	1	41	1
Passeridae	*Estrilda melanotis*				5	9	0	7	1	9	1	9	1	9	2	7	2	9	3	9	3
Passeridae	*Estrilda poliopareia*	T		x	5	0	7x	7	1x	7	0x	1	7x	0	7x	7	1x	7	0x	1	7x
Passeridae	*Estrilda paludicola*				5	39	2	25	3	41	1	44	0	42	2	32	5	43	1	44	0
Passeridae	*Estrilda melpoda*				2,5,11,13	24	4	16	5	27	4	37	3	34	4	21	7	38	6	40	4
Passeridae	*Estrilda rhodopyga*				5	34	4	20	11	39	2	39	2	37	4	23	14	40	3	40	3
Passeridae	*Estrilda rufibarba*				4	0	0	0	0	0	0	0	0	0	0	0	0	0	1	0	1
Passeridae	*Estrilda troglodytes*		H		2,5	0	2	0	2	0	1	0	1	0	2	0	3	0	3	0	1
Passeridae	*Estrilda astrild*				2,3,5,6,9,11,13	22	3	17	5	22	4	24	3	23	5	19	9	24	5	24	4
Passeridae	*Estrilda nigriloris*			x	5	95	0x	95	0x	95	0x	95	0x	95	0x	95	0x	95	0x	95	0x
Passeridae	*Estrilda nonnula*		H		5	0	7	0	11	0	9	0	5	0	8	0	12	0	9	0	5
Passeridae	*Estrilda atricapilla*				5	37	3	22	5	38	4	60	3	55	5	32	8	68	5	70	4
Passeridae	*Estrilda kandti*			x	5	30	9x	25	9x	38	6x	34	0x	31	9x	30	10x	38	6x	34	0x
Passeridae	*Estrilda charmosyna*				5	50	5	17	19	58	1	58	1	55	5	23	27	58	2	58	2
Passeridae	*Estrilda erythronotos*				5	10	4	7	5	10	4	10	4	10	7	8	11	11	6	10	6
Passeridae	*Amandava amandava*		H		2,3,4,5,7,11,13	0	4	0	4	0	4	0	4	0	7	0	8	0	10	0	5
Passeridae	*Amandava formosa*	T			7	13	2	13	2	13	2	17	1	14	4	14	6	14	7	24	1
Passeridae	*Amandava subflava*				4,5	20	2	16	3	22	1	23	1	22	2	19	5	23	2	24	2
Passeridae	*Ortygospiza atricollis*				5	8	2	4	2	11	1	15	1	14	2	7	3	16	3	16	1
Passeridae	*Ortygospiza fuscocrissa*				14	14	2	11	3	14	2	14	2	14	3	12	6	15	3	15	3
Passeridae	*Ortygospiza gabonensis*				5	28	3	22	2	29	0	33	0	30	3	27	5	35	0	34	0
Passeridae	*Ortygospiza locustella*				5	43	1	37	2	43	0	45	0	45	1	40	4	45	0	46	0
Passeridae	*Emblema pictum*				10	0	17	0	17	0	16	0	16	0	17	0	19	0	18	0	18
Passeridae	*Stagonopleura bella*				10	6	32	9	9	11	27	14	7	11	32	13	9	20	27	22	7
Passeridae	*Stagonopleura oculata*				10	0	29	0	14	0	23	0	2	0	37	0	14	0	29	0	2
Passeridae	*Stagonopleura guttata*				10	1	10	1	6	1	9	1	4	5	15	1	7	3	14	2	5
Passeridae	*Oreostruthus fuliginosus*				8	10	0	1	0	13	0	19	0	17	5	14	9	26	9	35	6x
Passeridae	*Neochmia temporalis*				10,11	5	14	7	7	10	11	11	5	10	20	9	11	15	15	17	7
Passeridae	*Neochmia phaeton*				8,10	0	19	0	13	1	17	2	6	0	20	1	17	2	21	2	9
Passeridae	*Neochmia ruficauda*				10	0	12	0	12	0	13	0	10	0	12	0	14	0	14	0	13
Passeridae	*Neochmia modesta*				10	1	24	1	17	2	20	1	14	1	29	1	27	2	31	2	16
Passeridae	*Taeniopygia guttata*				7,10	0	11	0	9	1	10	1	8	1	13	0	12	1	14	1	10
Passeridae	*Taeniopygia bichenovii*		H		10	0	20	0	13	0	17	0	9	0	24	0	20	0	24	0	11
Passeridae	*Poephila personata*				10	0	23	0	14	0	21	0	9	0	24	0	20	0	25	0	11

Family	Species	IUCN	Ag	R	Range Source	Percentage loss in geographic range 2050 TG H	C	AM H	C	GO H	C	OS H	C	2100 TG H	C	AM H	C	GO H	C	OS H	C
Passeridae	*Poephila acuticauda*				10	0	14	0	10	0	13	1	6	0	14	0	14	0	17	1	9
Passeridae	*Poephila cincta*				10	0	33	0	18	0	33	0	14	0	36	0	29	0	38	0	15
Passeridae	*Erythrura hyperythra*				7	11	7	19	6	14	8	19	7	15	10	23	12	25	15	27	11
Passeridae	*Erythrura prasina*				7	12	4	14	4	13	4	16	4	17	7	19	6	23	8	24	6
Passeridae	*Erythrura viridifacies*	T			7	6	9	17	6	10	10	11	8	6	18	24	13	19	18	18	11
Passeridae	*Erythrura tricolor*			x	7	10	24x	9	24x	14	17x	25	22x	10	24x	9	25x	15	18x	25	22x
Passeridae	*Erythrura trichroa*				7,8,9,10,11	10	5	14	3	21	5	16	4	16	8	19	10	33	12	30	10
Passeridae	*Erythrura coloria*			x	7	31	4x	30	6x	16	6x	30	6x	31	4x	30	6x	37	6x	30	6x
Passeridae	*Erythrura papuana*				8	10	2	2	2	15	2	14	0	14	16	6	18	24	18	42	5
Passeridae	*Erythrura psittacea*		H	x	9	0	28x	0	11x	0	16x	0	12x	0	28x	0	12x	0	18x	0	12x
Passeridae	*Erythrura pealii*		H		13	0	6	0	6	0	1	0	1	0	6	0	6	0	1	0	1
Passeridae	*Erythrura cyaneovirens*			x	9,11	15	5x	13	5x	27	1x	33	1x	24	5x	30	5x	65	1x	64	1x
Passeridae	*Erythrura regia*	T		x	9	7	0x	19	0x	40	0x	8	0x	10	0x	32	0x	50	0x	52	0x
Passeridae	*Erythrura kleinschmidti*	T	H		11	0	0	0	0	0	0	0	0	0	0	0	0	0	0	0	0
Passeridae	*Erythrura gouldiae*	T			10	0	14	0	10	0	14	0	4	0	14	0	14	0	17	0	7
Passeridae	*Lemuresthes nana*		H		6	0	0	0	4	0	0	0	0	0	0	0	4	0	0	0	0
Passeridae	*Spermestes caniceps*				14	45	3	32	8	47	2	47	2	46	3	40	9	50	2	49	2
Passeridae	*Spermestes cucullatus*		H		5,13	0	2	0	4	0	3	0	2	0	3	0	7	0	3	0	2
Passeridae	*Spermestes bicolor*				4,5	37	5	26	6	36	5	53	3	50	6	34	8	56	6	59	3
Passeridae	*Spermestes fringilloides*				5	47	4	31	7	44	6	57	3	56	5	37	8	60	6	61	3
Passeridae	*Lonchura cantans*				4,5	11	3	5	6	13	3	15	2	14	3	7	9	16	6	16	4
Passeridae	*Lonchura malabarica*				2,4,7,11	11	3	13	3	13	2	16	1	13	5	15	6	14	8	23	2
Passeridae	*Lonchura striata*		H		7	0	4	0	4	0	4	0	4	0	6	0	6	0	7	0	5
Passeridae	*Lonchura leucogastroides*		H		7	0	0	0	2	0	0	0	2	0	2	0	5	0	2	0	2
Passeridae	*Lonchura fuscans*		H		7	0	1	0	2	0	2	0	0	0	2	0	2	0	2	0	0
Passeridae	*Lonchura molucca*		H		7	0	11	0	11	0	9	0	12	0	15	0	17	0	15	0	14
Passeridae	*Lonchura kelaarti*		H		7	0	1	0	2	0	2	0	0	0	1	0	2	0	3	0	0
Passeridae	*Lonchura punctulata*		H		2,7,10,11,13	0	4	0	4	0	5	0	4	0	7	0	8	0	10	0	5
Passeridae	*Lonchura leucogastra*				7	15	4	19	4	17	4	21	4	21	6	24	5	31	6	30	5
Passeridae	*Lonchura tristissima*				8	5	3	5	2	24	1	24	1	14	7	13	6	39	4	35	2
Passeridae	*Lonchura leucosticta*				8	0	12	4	10	9	4	9	4	3	12	7	12	14	7	13	7
Passeridae	*Lonchura malacca*		H		2,7,11	0	5	0	5	0	6	0	5	0	8	0	7	0	10	0	7
Passeridae	*Lonchura ferruginosa*		H		7	0	0	0	0	0	0	0	0	0	0	0	2	0	0	0	0
Passeridae	*Lonchura quinticolor*		H		7	0	9	0	11	0	7	0	10	0	16	0	18	0	12	0	10
Passeridae	*Lonchura maja*		H		7	0	4	0	4	0	4	0	5	0	6	0	5	0	4	0	5
Passeridae	*Lonchura pallida*		H		7	0	9	0	10	0	8	0	11	0	14	0	18	0	14	0	13
Passeridae	*Lonchura grandis*				8	4	3	4	0	34	0	25	1	18	7	13	5	53	4	37	2
Passeridae	*Lonchura vana*	T		x	8	23	0x	6	0x	8	0x	0	0x	23	0x	6	0x	8	0x	65	0x
Passeridae	*Lonchura caniceps*			x	8	0	2x	3	0x	31	0x	55	0x	3	18x	17	22x	56	23x	64	6x
Passeridae	*Lonchura nevermanni*			x	8	1	21x	5	20x	32	7x	45	7x	1	21x	15	20x	44	7x	63	7x
Passeridae	*Lonchura spectabilis*			x	8	5	0x	0	0x	36	0x	13	0x	19	1x	6	2x	51	1x	35	1x
Passeridae	*Lonchura hunsteini*			x	8,11	0	0x	0	0x	41	0x	53	0x	41	0x	0	0x	88	0x	53	0x
Passeridae	*Lonchura forbesi*			x	8	0	0x	0	0x	24	0x	35	0x	24	0x	0	0x	88	0x	35	0x
Passeridae	*Lonchura nigerrima*			x	8	0	0x	0	0x	0	0x	0	0x	0	0x	0	0x	0	0x	0	0x

Family	Species	IUCN	Ag	R	Range Source	Percentage loss in geographic range 2050 TG		AM		GO		OS		2100 TG		AM		GO		OS	
						H	C	H	C	H	C	H	C	H	C	H	C	H	C	H	C
Passeridae	*Lonchura flaviprymna*				10	0	10	0	9	0	10	0	6	0	10	0	12	0	12	0	8
Passeridae	*Lonchura castaneothorax*				8,9,10,11	2	19	3	12	6	17	6	6	3	25	5	19	7	23	8	7
Passeridae	*Lonchura stygia*			x	8	0	1x	0	0x	0	0x	10	0x	5	1x	14	1x	15	1x	10	1x
Passeridae	*Lonchura teerinki*			x	8	0	0x	45	0x	9	0x	19	0x	6	5x	53	0x	10	0x	19	0x
Passeridae	*Lonchura montana*			x	8	0	0x	2	0x	0	0x	1	0x	7	1x	19	0x	0	0x	1	0x
Passeridae	*Lonchura monticola*			x	8	0	0x	0	0x	1	0x	0	0x	0	21x	0	96x	1	96x	20	80x
Passeridae	*Lonchura melaena*			x	8,9	1	0x	1	0x	59	0x	18	0x	45	0x	47	0x	75	0x	56	0x
Passeridae	*Lonchura oryzivora*	T	H		2,5,7,11,13	0	2	0	2	0	1	0	0	0	2	0	3	0	1	0	0
Passeridae	*Lonchura fuscata*	T	H		7	0	13	0	13	0	6	0	13	0	19	0	19	0	12	0	13
Passeridae	*Heteromunia pectoralis*				10	0	10	0	9	0	8	0	6	0	10	0	11	0	11	0	9
Passeridae	*Amadina fasciata*				5	19	3	11	7	20	3	22	2	21	4	13	10	22	4	23	4
Passeridae	*Amadina erythrocephala*				5	8	5	3	8	7	6	7	6	8	8	3	13	7	10	7	10
Passeridae	*Vidua chalybeata*				5	17	2	12	3	19	2	21	1	20	3	15	6	22	3	22	2
Passeridae	*Vidua camerunensis*				5	19	4	9	5	28	4	33	3	30	4	16	6	39	4	36	3
Passeridae	*Vidua larvaticola*				5	12	2	10	2	18	1	22	2	21	2	14	6	23	4	23	4
Passeridae	*Vidua funerea*				5	27	0	25	0	28	0	29	0	29	1	25	2	29	0	29	0
Passeridae	*Vidua maryae*			x	5	13	0x	33	0x	59	0x	59	0x	46	0x	46	0x	59	0x	59	0x
Passeridae	*Vidua nigeriae*				5	5	0	1	0	9	0	9	0	8	0	6	0	9	0	9	0
Passeridae	*Vidua raricola*				5	9	6	5	7	18	4	24	3	18	6	13	7	24	4	25	3
Passeridae	*Vidua codringtoni*				5	39	0	37	0	39	0	39	0	39	3	39	9	39	0	39	0
Passeridae	*Vidua purpurascens*				5	30	2	26	4	30	2	30	2	30	5	27	11	30	4	30	4
Passeridae	*Vidua wilsoni*				5	28	4	16	6	34	4	45	2	40	5	22	11	48	7	48	5
Passeridae	*Vidua hypocherina*				5	35	3	24	8	38	2	37	2	36	3	28	10	40	2	39	3
Passeridae	*Vidua fischeri*				5	41	3	18	19	48	1	48	1	45	4	23	23	48	3	48	2
Passeridae	*Vidua regia*				5	11	7	6	10	10	8	10	7	11	11	6	15	10	12	10	11
Passeridae	*Vidua macroura*				2,5	18	2	13	4	18	2	21	2	20	3	15	6	21	4	21	3
Passeridae	*Vidua orientalis*				5	8	2	4	3	8	2	12	1	11	2	6	6	12	5	13	3
Passeridae	*Vidua togoensis*				5	26	0	4	0	16	4	25	4	27	0	12	4	26	17x	25	4
Passeridae	*Vidua interjecta*				5	10	2	6	3	14	1	18	1	15	2	9	5	19	3	18	2
Passeridae	*Vidua paradisaea*				5	33	1	26	3	33	1	33	1	33	3	28	7	33	2	33	2
Passeridae	*Vidua obtusa*				5	32	1	27	2	33	1	33	1	33	2	29	7	33	2	33	1
Fringillidae	*Peucedramus taeniatus*				2	11	13	25	6	12	11	27	6	15	16	25	18	15	24	32	16
Fringillidae	*Fringilla coelebs*		H		3,4,5,7,12,13	0	21	0	18	0	22	0	16	0	25	0	22	0	26	0	20
Fringillidae	*Fringilla teydea*				13	0	0	0	0	0	0	9	0	0	0	0	0	0	20	9	11
Fringillidae	*Fringilla montifringilla*				4,5,7	1	12	1	12	1	12	1	12	2	14	1	18	2	19	3	18
Fringillidae	*Serinus pusillus*				3,4,7	16	16	17	16	18	16	20	15	21	21	21	23	26	23	28	21
Fringillidae	*Serinus serinus*		H		3,4,5	0	20	0	14	0	19	0	12	0	25	0	19	0	24	0	15
Fringillidae	*Serinus syriacus*	T		x	3,4	0	0x	22	0x	22	0x	22	0x	22	0x	22	0x	22	0x	22	0x
Fringillidae	*Serinus canaria*		H	x	3,11	0	8x	0	8x	0	8x	0	8x	0	8x	0	8x	0	13x	0	11x
Fringillidae	*Serinus citrinella*				3	13	20	7	20	12	19	19	14	16	30	13	36	24	38	26	25
Fringillidae	*Serinus thibetanus*				7	49	4	50	4	50	4	50	5	50	8	53	12	54	10	53	10
Fringillidae	*Serinus canicollis*		H		5,13	0	4	0	5	0	5	0	5	0	5	0	6	0	6	0	6
Fringillidae	*Serinus flavivertex*				14	25	2	20	2	27	1	27	1	26	2	21	4	27	2	27	1
Fringillidae	*Serinus nigriceps*				5	24	0	24	1	30	0	30	0	30	0	24	6	30	1	30	1

Family	Species	IUCN	Ag	R	Range Source	Percentage loss in geographic range 2050 TG H	TG C	AM H	AM C	GO H	GO C	OS H	OS C	2100 TG H	TG C	AM H	AM C	GO H	GO C	OS H	OS C
Fringillidae	*Serinus frontalis*				5	28	6	21	10	31	4	36	0	31	6	24	12	34	6	36	0
Fringillidae	*Serinus citrinelloides*				5	14	0	13	0	15	0	15	0	15	0	13	1	15	0	15	0
Fringillidae	*Serinus hypostictus*				5	48	1	46	1	48	1	48	1	48	1	48	4	48	1	48	1
Fringillidae	*Serinus capistratus*				5	48	1	36	2	48	1	60	1	57	4	41	6	60	2	63	1
Fringillidae	*Serinus koliensis*				5	35	2	19	7	36	1	36	0	35	2	26	9	36	1	36	0
Fringillidae	*Serinus scotops*				5	3	0	3	0	3	0	3	0	3	0	3	0	3	0	3	0
Fringillidae	*Serinus leucopygius*				5	7	2	4	2	7	2	10	1	9	2	5	6	11	5	11	3
Fringillidae	*Serinus rothschildi*		H		4	0	0	0	0	0	0	0	0	0	0	0	0	0	3	0	3
Fringillidae	*Serinus flavigula*	T		x	5	0	0x	0	0x	0	0x	0	0x	0	0x	0	0x	0	0x	0	0x
Fringillidae	*Serinus xantholaemus*	T		x	5	34	0x	6	0x	34	0x	34	0x	34	0x	6	0x	34	0x	34	0x
Fringillidae	*Serinus xanthopygius*				5	3	0	0	0	3	0	3	0	3	0	0	0	3	0	3	0
Fringillidae	*Serinus atrogularis*				5	16	3	11	5	16	4	17	3	16	5	11	9	16	6	17	5
Fringillidae	*Serinus reichenowi*				5	42	3	31	8	45	2	44	2	43	3	35	10	46	2	45	2
Fringillidae	*Serinus citrinipectus*		H	x	5	0	0x	0	0x	0	0x	0	0x	0	7x	0	2x	0	0x	0	0x
Fringillidae	*Serinus mozambicus*		H		2,5,11,13	0	3	0	4	0	3	0	2	0	4	0	7	0	4	0	3
Fringillidae	*Serinus donaldsoni*				5	40	9	13	16	47	4	47	4	42	9	18	19	47	4	47	4
Fringillidae	*Serinus buchanani*				5	39	4	27	5	43	0	43	0	39	4	40	5	45	0	45	0
Fringillidae	*Serinus dorsostriatus*				5	42	4	24	12	46	2	46	2	44	4	30	14	49	3	48	3
Fringillidae	*Serinus flaviventris*				5	6	5	2	7	5	6	5	5	6	7	2	12	5	10	6	9
Fringillidae	*Serinus sulphuratus*		H		5	0	2	0	2	0	2	0	1	0	3	0	5	0	3	0	2
Fringillidae	*Serinus albogularis*				5	3	6	1	6	3	7	2	6	3	8	1	9	3	9	3	9
Fringillidae	*Serinus reichardi*				5	56	0	48	1	56	0	56	0	57	1	50	4	56	1	56	1
Fringillidae	*Serinus gularis*				5	27	5	22	7	32	3	36	2	33	5	25	9	36	6	36	4
Fringillidae	*Serinus mennelli*				5	48	0	40	0	49	0	49	0	49	1	41	5	49	0	49	0
Fringillidae	*Serinus tristriatus*			x	5	18	0x	16	0x	19	0x	19	0x	19	0x	16	1x	19	0x	19	0x
Fringillidae	*Serinus menachensis*				4	0	0	0	0	0	0	0	0	0	0	0	0	0	10	0	10
Fringillidae	*Serinus ankoberensis*	T	H	x	5	0	0x	0	0x	0	0x	0	0x	0	0x	0	0x	0	0x	0	0x
Fringillidae	*Serinus striolatus*		H		5	0	3	0	5	0	1	0	1	0	3	0	6	0	2	0	1
Fringillidae	*Serinus whytii*				14	24	0	21	0	24	0	24	0	24	1	22	0	24	0	24	0
Fringillidae	*Serinus burtoni*				5	22	3	18	5	30	2	26	0	26	5	22	7	33	4	28	0
Fringillidae	*Serinus rufobrunneus*		H	x	5	0	88x	0	88x	0	0x	0	0x	0	88x	0	88x	0	0x	0	0x
Fringillidae	*Serinus melanochrous*			x	5	0	0x	0	0x	0	0x	0	0x	0	0x	0	0x	0	0x	0	0x
Fringillidae	*Serinus leucopterus*				5	6	2	6	2	0	8	6	2	6	2	6	5	0	11	6	5
Fringillidae	*Serinus totta*				5	0	3	0	3	0	3	0	3	0	3	0	7	0	7	0	7
Fringillidae	*Serinus symonsi*				5	3	0	3	0	3	0	3	0	3	0	3	0	3	0	3	0
Fringillidae	*Serinus alario*				5	2	8	1	9	3	9	1	9	2	10	1	11	4	11	2	12
Fringillidae	*Alario leucolaemus*				14	3	1	1	2	3	2	2	2	3	3	1	5	3	6	2	6
Fringillidae	*Serinus estherae*				7	19	8	22	6	22	4	15	8	23	8	26	13	33	15	25	14
Fringillidae	*Neospiza concolor*	T		x	5	0	102x	0	102x	100	0x	100	0x	0	102x	0	102x	100	0x	100	0x
Fringillidae	*Linurgus olivaceus*				5	23	3	18	4	27	3	28	1	25	4	22	8	31	4	29	1
Fringillidae	*Rhynchostruthus socotranu*			x	4,5	0	0x	0	0x	0	0x	0	0x	0	0x	0	0x	0	11x	0	11x
Fringillidae	*Carduelis chloris*		H		3,4,5,10,12,13	0	21	0	17	0	21	0	16	0	26	0	22	0	26	0	19
Fringillidae	*Carduelis sinica*		H		7	0	21	0	24	0	23	0	22	0	28	0	33	0	33	0	32
Fringillidae	*Carduelis spinoides*		H		7	0	6	0	5	0	6	0	6	0	8	0	9	0	9	0	8

Family	Species	IUCN	Ag	R	Range Source	Percentage loss in geographic range 2050								2100							
						TG		AM		GO		OS		TG		AM		GO		OS	
						H	C	H	C	H	C	H	C	H	C	H	C	H	C	H	C
Fringillidae	*Carduelis ambigua*		H		7	0	5	0	5	0	6	0	6	0	8	0	10	0	11	0	9
Fringillidae	*Carduelis monguilloti*			x	7	22	4x	19	4x	8	4x	8	4x	22	4x	19	11x	14	11x	28	11x
Fringillidae	*Carduelis spinus*				3,4,5,7	7	21	7	19	9	22	8	18	10	25	9	24	13	27	13	22
Fringillidae	*Carduelis pinus*				2	6	20	10	17	8	18	11	16	8	25	12	26	14	28	16	23
Fringillidae	*Carduelis atriceps*			x	2	29	0x	41	0x	29	0x	41	0x	29	0x	41	0x	29	0x	41	0x
Fringillidae	*Carduelis spinescens*		H		2	0	3	0	3	0	3	0	3	0	7	0	7	0	9	0	5
Fringillidae	*Carduelis yarrellii*	T			2	9	25	7	23	9	23	17	16	10	31	10	25	17	25	26	18
Fringillidae	*Carduelis cucullata*	T			2	29	4	24	6	32	7	23	4	36	10	35	11	38	22	32	19
Fringillidae	*Carduelis crassirostris*				2	9	15	7	17	4	17	12	11	12	19	9	20	9	21	19	14
Fringillidae	*Carduelis magellanica*		H		2	0	12	0	9	0	10	0	7	0	17	0	13	0	15	0	11
Fringillidae	*Carduelis siemiradzkii*	T		x	2	0	1x	0	0x	0	1x	0	0x	0	1x	0	0x	0	1x	0	0x
Fringillidae	*Carduelis olivacea*				2	20	6	14	5	15	6	27	6	27	6	23	9	34	9	35	8
Fringillidae	*Carduelis notata*				2	13	8	29	4	19	8	36	4	18	9	31	11	24	13	40	7
Fringillidae	*Carduelis barbata*				2	3	7	2	6	2	7	2	7	4	10	4	13	5	15	5	14
Fringillidae	*Carduelis xanthogastra*				2	21	6	21	5	15	6	31	3	28	10	33	10	30	15	40	5
Fringillidae	*Carduelis atrata*		H		2	0	17	0	18	0	18	0	16	0	21	0	25	0	26	0	21
Fringillidae	*Carduelis uropygialis*				2	5	9	6	4	0	6	4	6	5	10	7	5	8	7	7	9
Fringillidae	*Carduelis tristis*		H		2	0	20	0	13	0	16	0	11	0	23	0	17	0	21	0	14
Fringillidae	*Carduelis psaltria*		H		2	0	16	0	13	0	15	0	12	0	18	0	24	0	27	0	20
Fringillidae	*Carduelis lawrencei*				2	0	41	2	31	2	44	7	23	1	46	2	39	4	49	11	30
Fringillidae	*Carduelis dominicensis*			x	2	3	0x	3	0x	4	0x	4	0x	3	0x	3	0x	4	0x	4	0x
Fringillidae	*Carduelis carduelis*		H		,3,4,5,7,10,12,1	0	18	0	16	0	19	0	15	0	22	0	19	0	23	0	17
Fringillidae	*Carduelis hornemanni*				2,3,7	0	19	0	19	0	18	0	18	0	27	0	32	0	34	0	30
Fringillidae	*Carduelis flammea*				2,3,4,7,12,13	1	17	1	17	1	17	1	17	2	22	2	26	2	28	3	26
Fringillidae	*Carduelis flavirostris*				3,4,7	9	14	9	14	10	15	10	14	10	20	12	24	15	25	15	23
Fringillidae	*Carduelis cannabina*		H		3,4,5,7	0	17	0	14	0	18	0	12	0	19	0	16	0	20	0	14
Fringillidae	*Carduelis yemenensis*				4	0	0	0	0	0	0	0	0	0	0	0	0	0	3	0	3
Fringillidae	*Carduelis johannis*	T		x	5	0	0x	0	0x	0	0x	0	0x	0	0x	0	0x	0	0x	0	0x
Fringillidae	*Leucosticte nemoricola*				7	13	18	15	18	15	18	15	17	15	27	18	32	23	33	23	30
Fringillidae	*Leucosticte brandti*				7	13	19	14	19	15	19	15	18	15	29	17	35	22	35	23	32
Fringillidae	*Leucosticte arctoa*				7	4	13	4	13	4	13	4	13	4	16	4	19	4	20	4	19
Fringillidae	*Leucosticte tephrocotis*				2	0	28	0	28	0	28	0	28	0	35	1	40	1	41	1	39
Fringillidae	*Leucosticte atrata*				2	0	13	1	10	0	10	1	10	0	20	1	20	2	28	5	23
Fringillidae	*Leucosticte australis*			x	2	4	7x	3	4x	24	8x	19	5x	8	8x	36	8x	44	13x	38	8x
Fringillidae	*Callacanthis burtoni*				7	70	4	71	1	71	1	76	1	73	6	78	2	79	2	80	2
Fringillidae	*Rhodopechys sanguinea*				3,4,5,7	11	15	11	15	13	16	16	15	19	19	15	20	20	21	24	20
Fringillidae	*Rhodopechys githaginea*				3,4,5,7	4	3	4	3	4	3	5	3	6	4	5	5	6	5	8	4
Fringillidae	*Rhodopechys mongolica*				4,7	10	12	10	13	11	14	11	13	11	16	12	20	15	21	16	20
Fringillidae	*Rhodopechys obsoleta*		H		3,4,7	0	8	0	9	0	9	0	9	0	11	0	15	0	16	0	15
Fringillidae	*Uragus sibiricus*				7	15	6	15	7	15	7	16	7	16	9	16	12	18	11	19	10
Fringillidae	*Carpodacus rubescens*				7	37	2	39	2	39	2	40	1	39	3	41	5	40	4	41	3
Fringillidae	*Carpodacus nipalensis*				7	45	2	47	2	47	2	49	0	48	2	49	3	48	2	51	1
Fringillidae	*Carpodacus erythrinus*		H		3,4,7	0	14	0	14	0	16	0	13	0	18	0	20	0	21	0	19
Fringillidae	*Carpodacus purpureus*				2	9	26	14	21	14	23	15	19	13	31	19	28	22	30	22	25

Family	Species	IUCN	Ag	R	Range Source	Percentage loss in geographic range 2050 TG H	TG C	AM H	AM C	GO H	GO C	OS H	OS C	2100 TG H	TG C	AM H	AM C	GO H	GO C	OS H	OS C
Fringillidae	*Carpodacus cassinii*				2	3	20	8	16	5	19	9	16	5	28	12	29	12	34	18	24
Fringillidae	*Carpodacus mexicanus*		H		2,11	0	18	0	12	0	15	0	9	0	21	0	19	0	23	0	14
Fringillidae	*Carpodacus pulcherrimus*				7	27	8	29	8	29	9	30	7	29	15	32	21	35	19	35	16
Fringillidae	*Carpodacus eos*				7	29	15	31	15	31	16	32	15	30	28	33	37	40	35	41	30
Fringillidae	*Carpodacus rodochrous*				7	65	7	69	6	68	5	76	3	68	12	77	10	76	12	78	9
Fringillidae	*Carpodacus vinaceus*				7	31	0	33	0	33	0	35	0	33	0	35	1	33	0	35	0
Fringillidae	*Carpodacus edwardsii*				7	45	5	47	3	47	3	47	4	46	7	52	8	49	8	48	8
Fringillidae	*Carpodacus synoicus*				3,4,7	6	14	7	15	8	15	7	14	8	19	8	24	12	25	14	24
Fringillidae	*Carpodacus roseus*				7	0	7	0	7	0	7	0	7	0	9	0	9	0	9	0	9
Fringillidae	*Carpodacus trifasciatus*				7	31	1	31	3	31	2	31	1	32	3	33	7	33	6	33	4
Fringillidae	*Carpodacus rodopeplus*				7	45	3	46	2	46	2	49	1	47	4	49	4	49	4	50	3
Fringillidae	*Carpodacus thura*				7	34	10	37	9	37	10	38	9	36	18	41	24	46	21	46	18
Fringillidae	*Carpodacus rhodochlamys*				7	8	15	9	16	9	15	9	15	10	19	10	20	13	20	15	20
Fringillidae	*Carpodacus rubicilloides*				7	25	14	28	14	28	15	29	13	27	24	32	31	37	30	38	26
Fringillidae	*Carpodacus rubicilla*				3,4,7	12	18	13	18	13	18	13	17	13	27	15	32	20	32	20	30
Fringillidae	*Carpodacus puniceus*				4,7	16	17	18	16	18	16	18	15	18	26	21	31	26	31	27	28
Fringillidae	*Carpodacus roborowskii*				7	9	22	12	24	10	27	13	23	11	35	14	46	29	41	35	31
Fringillidae	*Pinicola enucleator*				2,3,7	1	14	2	14	2	14	2	14	2	18	3	20	4	21	5	19
Fringillidae	*Pinicola subhimachalus*				7	37	5	39	4	39	3	40	3	38	10	42	13	43	13	43	10
Fringillidae	*Haematospiza sipahi*		H		7	0	2	0	2	0	2	0	0	0	3	0	3	0	2	0	1
Fringillidae	*Loxia pytyopsittacus*				3	4	28	4	28	5	28	5	27	5	35	6	35	9	36	10	34
Fringillidae	*Loxia scotica*			x	3	0	19x	15	39x	1	39x	0	26x	15	40x	16	40x	2	40x	0	40x
Fringillidae	*Loxia curvirostra*				2,3,4,5,7	6	18	8	16	7	18	9	16	7	23	10	24	11	26	14	22
Fringillidae	*Loxia leucoptera*				2,7	3	15	5	14	5	15	6	13	5	19	7	19	8	20	9	18
Fringillidae	*Pyrrhula nipalensis*				7	24	3	25	3	25	3	27	2	26	4	27	4	28	4	29	4
Fringillidae	*Pyrrhula leucogenis*				7	14	10	26	11x	6	11	12	11	14	14x	26	15x	19	13x	19	11x
Fringillidae	*Pyrrhula aurantiaca*				7	72	5	70	1	74	1	78	2	74	6	80	1	84	1	83	2
Fringillidae	*Pyrrhula erythrocephala*				7	73	5	74	3	73	3	80	2	75	8	81	5	81	5	83	4
Fringillidae	*Pyrrhula erythaca*				7	23	3	24	3	24	4	24	3	23	7	25	10	28	7	28	6
Fringillidae	*Pyrrhula pyrrhula*		H		3,4,7	0	18	0	16	0	19	0	15	0	21	0	21	0	23	0	20
Fringillidae	*Coccothraustes coccothrau*		H		3,4,5,7	0	19	0	17	0	21	0	15	0	22	0	20	0	24	0	18
Fringillidae	*Eophona migratoria*		H		7	0	2	0	3	0	3	0	3	0	3	0	3	0	3	0	3
Fringillidae	*Eophona personata*		H		7	0	11	0	11	0	12	0	12	0	16	0	19	0	18	0	18
Fringillidae	*Mycerobas icterioides*				7	60	4	58	2	60	0	64	1	63	4	67	2	68	0	71	1
Fringillidae	*Mycerobas affinis*				7	46	4	47	4	46	5	48	4	49	7	51	6	51	6	51	6
Fringillidae	*Mycerobas melanozanthos*				7	48	4	51	3	48	3	51	3	52	5	55	4	53	5	54	4
Fringillidae	*Mycerobas carnipes*				4,7	41	4	43	3	43	4	44	3	44	7	47	7	47	6	47	5
Fringillidae	*Hesperiphona vespertina*				2	10	23	16	18	15	21	18	16	14	28	22	26	24	30	27	23
Fringillidae	*Hesperiphona abeillei*				2	11	10	28	5	14	8	44	5	18	13	31	14	22	19	49	11
Fringillidae	*Pyrrhoplectes epauletta*				7	49	6	53	4	54	3	55	3	53	8	57	7	58	8	57	7
Fringillidae	*Telespiza ultima*	T		x	11	100	0x	0	0x	0	0x	0	0x	100	0x	0	0x	100	0x	100	0x
Fringillidae	*Psittirostra psittacea*	T		x	11	13	1x	19	8x	32	9x	14	8x	35	2x	49	8x	70	9x	62	8x
Fringillidae	*Loxioides bailleui*	T		x	11	0	0x	23	0x	20	0x	22	0x	30	0x	78	0x	57	0x	92	0x
Fringillidae	*Pseudonestor xanthophrys*	T		x	11	0	0x	0	0x	0	0x	0	0x	0	0x	0	0x	100	0x	0	0x

Family	Species	IUCN	Ag	R	Range Source	2050 TG H	2050 TG C	2050 AM H	2050 AM C	2050 GO H	2050 GO C	2050 OS H	2050 OS C	2100 TG H	2100 TG C	2100 AM H	2100 AM C	2100 GO H	2100 GO C	2100 OS H	2100 OS C
						Percentage loss in geographic range															
Fringillidae	*Hemignathus kauaiensis*	T		x	11	5	2x	5	2x	60	7x	35	0x	39	2x	5	2x	60	7x	95	0x
Fringillidae	*Hemignathus virens*				11	4	0	14	0	20	0	26	0	41	3	57	3	55	3	77	3x
Fringillidae	*Hemignathus chloris*	T		x	11	42	19x	19	61x	0	61x	19	61x	42	19x	19	61x	39	61x	19	61x
Fringillidae	*Hemignathus parvus*	T		x	11	5	1x	5	1x	56	5x	39	0x	43	1x	5	1x	56	5x	95	0x
Fringillidae	*Hemignathus lucidus*	T		x	11	9	1x	15	9x	28	10x	11	9x	30	1x	54	9x	70	10x	69	9x
Fringillidae	*Hemignathus munroi*	T		x	11	0	0x	18	0x	18	0x	23	0x	41	0x	77	0x	56	0x	100	0x
Fringillidae	*Oreomystis bairdi*	T		x	11	3	0x	3	0x	56	4x	40	0x	43	0x	3	0x	56	4x	97	0x
Fringillidae	*Oreomystis mana*	T		x	11	0	0x	22	0x	22	0x	11	0x	33	0x	89	0x	60	0x	100	0x
Fringillidae	*Paroreomyza montana*	T		x	11	0	0x	0	0x	45	0x	0	0x	0	1x	45	1x	99	1x	0	1x
Fringillidae	*Paroreomyza maculata*	T		x	11	52	6x	11	57x	0	57x	11	57x	52	6x	11	57x	43	57x	11	57x
Fringillidae	*Loxops caeruleirostris*	T		x	11	3	1x	3	1x	62	4x	34	0x	37	1x	3	1x	62	4x	97	0x
Fringillidae	*Loxops coccineus*	T		x	11	11	1x	15	12x	19	12x	12	12x	34	1x	58	12x	69	12x	63	12x
Fringillidae	*Vestiaria coccinea*				11	8	2	20	7	24	8	23	4	33	3	57	7x	60	8x	74	5x
Fringillidae	*Palmeria dolei*	T		x	11	21	0x	21	0x	42	0x	0	0x	21	2x	42	2x	98	2x	21	2x
Fringillidae	*Himatione sanguinea*				11	7	3	17	6	22	8	26	4	35	6	50	9	53	10	69	7
Fringillidae	*Melamprosops phaeosoma*	T		x	11	0	0x	0	0x	0	0x	0	0x	0	0x	0	0x	100	0x	0	0x
Fringillidae	*Urocynchramus pylzowi*				7	31	7	33	9	34	9	36	7	33	20	36	30	49	18	49	15
Fringillidae	*Melophus lathami*		H		7	0	4	0	3	0	4	0	4	0	5	0	6	0	7	0	5
Fringillidae	*Latoucheornis siemsseni*				7	29	0	29	0	28	0	28	0	29	1	31	4	31	1	28	4
Fringillidae	*Emberiza citrinella*		H		3,4,7,12,13	0	20	0	16	0	21	0	15	0	23	0	19	0	24	0	17
Fringillidae	*Emberiza leucocephalos*				4,7	4	10	4	10	4	11	4	9	5	12	5	12	6	14	7	12
Fringillidae	*Emberiza stewarti*				4,7	13	9	14	10	15	10	16	9	14	11	16	11	17	10	27	9
Fringillidae	*Emberiza cirlus*				3,4,5,12,13	6	17	8	12	9	14	10	9	10	23	11	19	18	21	14	13
Fringillidae	*Emberiza koslowi*				7	15	18	16	19	16	21	18	18	17	35	19	52	35	46	38	36
Fringillidae	*Emberiza cia*				3,4,5,7	8	11	8	10	9	11	11	9	11	14	11	14	14	15	17	11
Fringillidae	*Emberiza godlewskii*				7	12	6	13	7	13	7	14	6	13	10	14	13	17	11	18	10
Fringillidae	*Emberiza cioides*				7	6	7	7	7	7	7	7	7	7	9	7	11	9	12	9	12
Fringillidae	*Emberiza jankowskii*	T			7	9	0	9	0	9	0	9	0	9	0	9	0	9	0	9	0
Fringillidae	*Emberiza buchanani*				3,4,7	6	11	6	11	8	12	10	11	10	13	9	13	13	14	16	13
Fringillidae	*Emberiza cineracea*				3,4	22	0	23	0	22	0	31	0	32	0	26	0	32	0	32	0
Fringillidae	*Emberiza hortulana*		H		3,4,5,7	0	19	0	18	0	22	0	16	0	23	0	21	0	25	0	19
Fringillidae	*Emberiza caesia*				3,4	20	5	19	4	21	6	32	3	34	6	29	5	32	8	35	4
Fringillidae	*Emberiza striolata*				3,4,5,7	5	3	4	3	5	3	7	2	7	3	5	4	7	4	8	3
Fringillidae	*Emberiza impetuani*				5	3	5	2	6	3	6	2	6	3	7	2	9	3	8	2	9
Fringillidae	*Emberiza tahapisi*				4,5	19	3	12	4	20	2	23	2	22	4	15	7	23	4	23	3
Fringillidae	*Emberiza socotrana*	T		x	4,5	0	0x	0	0x	0	0x	0	0x	0	0x	0	0x	0	0x	0	0x
Fringillidae	*Emberiza capensis*				5	6	5	5	6	6	6	6	6	6	7	5	9	7	9	6	9
Fringillidae	*Emberiza vincenti*			x	14	54	0x	52	0x	54	0x	54	0x	54	0x	52	1x	54	0x	54	0x
Fringillidae	*Emberiza tristrami*				7	10	2	10	2	10	2	10	2	11	6	10	8	12	7	13	8
Fringillidae	*Emberiza fucata*		H		7	0	4	0	4	0	4	0	4	0	6	0	7	0	6	0	6
Fringillidae	*Emberiza pusilla*				3,4,7	0	14	0	15	0	15	0	15	1	18	1	22	1	23	1	23
Fringillidae	*Emberiza chrysophrys*				7	0	1	0	1	0	1	0	1	0	2	0	2	0	2	0	2
Fringillidae	*Emberiza rustica*				3,4,7	1	12	1	12	1	13	1	13	2	15	2	18	2	19	3	18
Fringillidae	*Emberiza elegans*				7	10	3	10	3	10	3	9	4	11	4	10	4	11	4	10	4

						Percentage loss in geographic range															
Family	Species	IUCN	Ag	R	Range Source	2050								2100							
						TG		AM		GO		OS		TG		AM		GO		OS	
						H	C	H	C	H	C	H	C	H	C	H	C	H	C	H	C
Fringillidae	Emberiza aureola		H		3,4,7	0	14	0	14	0	16	0	13	0	16	0	18	0	21	0	18
Fringillidae	Emberiza rutila		H		7	0	3	0	3	0	3	0	3	0	4	0	4	0	4	0	4
Fringillidae	Emberiza flaviventris				5	28	3	20	4	28	3	31	2	31	4	21	9	31	5	32	4
Fringillidae	Emberiza poliopleura				5	49	6	13	31	56	2	57	2	52	6	18	36	57	4	57	3
Fringillidae	Emberiza affinis		H		5	0	3	0	6	0	2	0	2	0	3	0	7	0	3	0	2
Fringillidae	Emberiza cabanisi				5	45	3	32	5	44	3	52	2	50	4	36	9	52	4	54	3
Fringillidae	Emberiza melanocephala		H		3,4	0	8	0	8	0	10	0	7	0	11	0	10	0	12	0	9
Fringillidae	Emberiza bruniceps				4,7	3	8	3	9	3	11	4	9	4	11	4	15	6	18	10	16
Fringillidae	Emberiza sulphurata	T			7	5	23	6	18	11	22	5	21	6	33	7	39	12	40	9	40
Fringillidae	Emberiza spodocephala		H		7	0	4	0	4	0	4	0	4	0	5	0	6	0	6	0	6
Fringillidae	Emberiza variabilis				7	5	23	3	22	4	26	3	24	7	31	5	36	7	37	14	34
Fringillidae	Emberiza pallasi				7	3	10	3	10	3	10	3	10	3	12	3	15	3	16	4	16
Fringillidae	Emberiza schoeniclus				3,4,5,7	4	17	4	16	6	19	4	15	7	20	6	19	9	22	9	17
Fringillidae	Emberiza yessoensis				7	7	3	5	4	6	4	6	5	9	4	6	5	8	5	7	6
Fringillidae	Emberiza calandra		H		3,4,5,7	0	12	0	9	0	12	0	8	0	14	0	13	0	16	0	11
Fringillidae	Calcarius mccownii		H		2	0	21	0	17	0	20	0	12	0	21	0	17	0	20	0	12
Fringillidae	Calcarius lapponicus				2,3,4,7	0	21	0	20	0	20	0	20	0	29	0	36	0	38	0	35
Fringillidae	Calcarius pictus		H		2	0	24	0	24	0	23	0	23	0	39	0	48	0	52	0	43
Fringillidae	Calcarius ornatus				2	1	19	0	15	1	18	0	11	1	19	1	15	1	18	1	11
Fringillidae	Plectrophenax nivalis				2,3,4,7	0	17	0	17	0	16	0	16	0	26	0	33	0	35	0	30
Fringillidae	Plectrophenax hyperboreus			x	2	0	0x	0	0x	0	0x	0	0x	0	18x	0	45x	0	45x	0	45x
Fringillidae	Calamospiza melanocorys				2	0	13	0	11	0	13	1	8	1	15	0	12	1	14	1	10
Fringillidae	Passerella iliaca				2	2	20	3	19	2	19	3	18	2	25	3	28	4	29	5	26
Fringillidae	Melospiza melodia				2	5	19	8	14	8	17	9	12	7	22	11	20	13	23	13	17
Fringillidae	Melospiza lincolnii				2	4	20	6	18	6	19	7	18	5	25	8	26	10	28	10	25
Fringillidae	Melospiza georgiana				2	6	17	9	13	10	15	9	11	8	20	13	17	15	19	14	15
Fringillidae	Zonotrichia capensis		H		2	0	13	0	11	0	12	0	9	0	17	0	16	0	18	0	14
Fringillidae	Zonotrichia querula				2	0	15	0	15	0	15	0	15	0	23	0	27	0	29	0	23
Fringillidae	Zonotrichia albicollis				2	6	17	9	15	9	16	9	14	8	21	12	20	13	22	13	19
Fringillidae	Zonotrichia leucophrys				2	1	20	1	19	1	19	2	18	1	26	2	30	3	32	4	28
Fringillidae	Zonotrichia atricapilla				2	0	27	0	27	0	28	0	27	0	36	0	41	1	42	1	40
Fringillidae	Junco vulcani			x	2	44	0x	100	0x	44	0x	44	0x	44	0x	100	0x	100	0x	100	0x
Fringillidae	Junco hyemalis				2	4	20	6	18	6	19	7	17	5	25	9	26	10	28	11	24
Fringillidae	Junco phaeonotus		H		2	0	8	0	5	0	8	0	4	0	8	0	15	0	16	0	8
Fringillidae	Junco bairdi			x	2	0	0x	0	0x	0	0x	38	0x	0	0x	0	0x	0	0x	38	0x
Fringillidae	Passerculus sandwichensis		H		2	0	20	0	17	0	19	0	16	0	25	0	26	0	28	0	23
Fringillidae	Ammodramus maritimus				2	13	43	24	12	13	39	6	9	21	53	38	25	31	48	13	10
Fringillidae	Ammodramus nelsoni				2	3	17	4	12	7	15	3	8	4	19	5	13	8	17	4	10
Fringillidae	Ammodramus caudacutus	T		x	2	6	67x	36	24x	38	45x	28	7x	11	68x	54	24x	50	49x	63	7x
Fringillidae	Ammodramus leconteii				2	3	18	3	13	5	16	3	11	4	20	5	16	6	19	4	14
Fringillidae	Ammodramus henslowii		H		2	0	12	0	4	0	9	0	1	0	13	0	5	0	10	0	1
Fringillidae	Ammodramus bairdii				2	1	18	2	13	2	16	1	9	1	19	2	13	2	17	1	9
Fringillidae	Ammodramus savannarum		H		2	0	13	0	8	0	11	0	6	0	15	0	10	0	14	0	6
Fringillidae	Ammodramus humeralis				2	8	14	6	11	7	12	12	10	11	18	10	15	16	17	19	13

Family	Species	IUCN	Ag	R	Range Source	Percentage loss in geographic range 2050								2100							
						TG		AM		GO		OS		TG		AM		GO		OS	
						H	C	H	C	H	C	H	C	H	C	H	C	H	C	H	C
Fringillidae	*Ammodramus aurifrons*		H		2	0	6	0	6	0	6	0	5	0	7	0	8	0	8	0	6
Fringillidae	*Xenospiza baileyi*	T			2	24	15	49	5	25	10	36	5	25	16	49	11	29	21	41	10
Fringillidae	*Spizella arborea*				2	0	22	0	21	0	21	0	21	0	29	0	34	0	36	0	33
Fringillidae	*Spizella passerina*				2	5	18	8	14	7	17	8	12	8	23	11	21	13	25	12	18
Fringillidae	*Spizella pallida*		H		2	0	21	0	16	0	19	0	12	0	22	0	17	0	21	0	14
Fringillidae	*Spizella breweri*				2	1	19	1	17	1	19	1	15	1	21	1	21	2	24	3	19
Fringillidae	*Spizella pusilla*		H		2	0	17	0	8	0	13	0	5	0	20	0	10	0	15	0	6
Fringillidae	*Spizella wortheni*	T			2	6	22	15	8	10	10	15	12	6	26	15	20	15	16	16	17
Fringillidae	*Spizella atrogularis*				2	4	22	7	18	5	20	8	19	4	26	7	36	7	38	9	34
Fringillidae	*Pooecetes gramineus*		H		2	0	19	0	14	0	17	0	12	0	22	0	19	0	22	0	16
Fringillidae	*Chondestes grammacus*		H		2	0	14	0	10	0	13	0	9	0	17	0	16	0	20	0	14
Fringillidae	*Amphispiza bilineata*				2	2	16	4	14	2	15	5	13	2	19	4	27	3	29	6	23
Fringillidae	*Amphispiza belli*				2	0	11	1	11	0	12	1	10	0	12	1	13	1	15	1	12
Fringillidae	*Aimophila quinquestriata*				2	35	3x	42	2x	29	3x	57	2x	35	3x	42	12x	32	18x	59	7x
Fringillidae	*Aimophila mystacalis*				2	1	6	10	0	1	6	7	0	1	6	10	0	3	6	7	0
Fringillidae	*Aimophila humeralis*			x	2	36	19x	47	19x	44	19x	63	0x	36	19x	47	26x	44	19x	63	0x
Fringillidae	*Aimophila ruficauda*		H		2	0	3	0	2	0	3	0	1	0	3	0	12	0	6	0	1
Fringillidae	*Aimophila sumichrasti*			x	2	40	20x	57	0x	45	0x	45	0x	40	20x	57	20x	57	0x	45	0x
Fringillidae	*Aimophila strigiceps*				2	1	7	1	3	1	5	2	4	1	9	2	9	2	12	2	11
Fringillidae	*Aimophila stolzmanni*				2	3	17	0	21	0	21	0	21	3	19	0	31	0	31	0	31
Fringillidae	*Aimophila aestivalis*		H		2	0	22	0	9	0	15	0	7	0	27	0	15	0	21	0	8
Fringillidae	*Aimophila botterii*				2	7	11	15	7	10	8	16	7	8	13	15	25	13	12	18	10
Fringillidae	*Aimophila cassinii*				2	1	18	3	15	1	16	3	14	1	22	3	28	2	30	3	24
Fringillidae	*Aimophila carpalis*				2	1	18	4	17	1	20	9	16	4	18	4	33	1	33	9	31
Fringillidae	*Aimophila ruficeps*				2	5	13	11	11	5	13	14	10	6	16	11	24	8	28	17	22
Fringillidae	*Aimophila notosticta*			x	2	0	1x	3	0x	0	1x	1	0x	0	1x	3	0x	2	1x	1	0x
Fringillidae	*Aimophila rufescens*				2	15	9	31	4	18	9	35	5	19	10	32	10	23	16	39	9
Fringillidae	*Torreornis inexpectata*	T		x	2	5	27x	0	27x	27	0x	91	0x	5	27x	0	27x	27	0x	91	0x
Fringillidae	*Oriturus superciliosus*				2	21	4	30	3	17	3	32	1	23	4	30	17	24	17	43	7
Fringillidae	*Pipilo chlorurus*				2	0	8	0	8	0	8	0	7	0	9	1	12	1	15	1	12
Fringillidae	*Pipilo ocai*				2	4	1	21	1	13	1	25	0	9	1	21	3	25	1	30	0
Fringillidae	*Pipilo maculatus*				2	4	18	7	14	5	17	8	12	5	21	8	23	8	28	12	20
Fringillidae	*Pipilo erythrophthalmus*				2	16	26	26	13	24	19	26	9	25	29	35	17	42	23	38	10
Fringillidae	*Pipilo albicollis*			x	2	2	0x	7	0x	2	0x	2	0x	2	0x	7	1x	6	0x	2	0x
Fringillidae	*Pipilo fuscus*				2	2	20	6	18	3	19	7	18	3	22	6	33	4	35	8	32
Fringillidae	*Pipilo crissalis*				2	0	29	2	19	1	29	3	12	1	29	2	22	2	29	4	14
Fringillidae	*Pipilo aberti*				2	0	32	0	38	0	39	0	38	0	42	0	59	0	70	0	65
Fringillidae	*Melozone kieneri*				2	14	8	29	4	15	8	35	2	17	11	29	13	21	13	37	4
Fringillidae	*Melozone biarcuatum*		H		2	0	0	0	0	0	0	0	0	0	0	0	1	0	3	0	0
Fringillidae	*Melozone cabanisi*			x	14	0	0x	27	0x	0	0x	60	0x	60	0x	27	60x	27	60x	87	0x
Fringillidae	*Melozone leucotis*			x	2	2	0x	5	0x	2	0x	6	0x	5	0x	5	4x	5	3x	8	0x
Fringillidae	*Arremon aurantiirostris*				2	15	5	21	6	19	6	31	4	24	6	33	9	32	8	45	5
Fringillidae	*Arremon schlegeli*				2	19	6	15	11	13	11	11	7	21	14	18	17	19	27	16	19
Fringillidae	*Arremon taciturnus*				2	6	15	4	15	4	15	9	14	10	17	7	18	16	18	17	17

Family	Species	IUCN	Ag	R	Range Source	2050 TG H	2050 TG C	2050 AM H	2050 AM C	2050 GO H	2050 GO C	2050 OS H	2050 OS C	2100 TG H	2100 TG C	2100 AM H	2100 AM C	2100 GO H	2100 GO C	2100 OS H	2100 OS C
						Percentage loss in geographic range															
Fringillidae	*Arremon franciscanus*				14	10	22	5	1	1	11	16	0	12	40	12	2	17	12	17	1
Fringillidae	*Arremon semitorquatus*				14	22	18	17	27	19	19	18	25	22	40	25	42	50	25	33	40
Fringillidae	*Arremon abeillei*			x	2	0	14x	0	12x	0	14x	1	12x	0	14x	0	19x	1	22x	1	19x
Fringillidae	*Arremon flavirostris*				2	11	13	9	12	9	13	17	11	15	16	17	15	23	16	28	14
Fringillidae	*Arremonops rufivirgatus*				2	3	6	10	4	6	5	16	1	4	10	12	29	9	12	18	3
Fringillidae	*Arremonops tocuyensis*				2	28	9	25	16	19	16	14	11	30	27	28	27	20	48	18	40
Fringillidae	*Arremonops chloronotus*				2	12	5	22	3	14	5	32	3	15	6	29	11	21	12	40	7
Fringillidae	*Arremonops conirostris*				2	15	6	14	7	16	7	22	4	19	7	20	9	28	10	31	6
Fringillidae	*Atlapetes albinucha*				2	7	0	8	0	8	0	15	0	7	0	15	2	8	0	15	0
Fringillidae	*Atlapetes latinuchus*				14	13	6	12	5	8	9	16	6	15	8	23	6	24	12	19	8
Fringillidae	*Atlapetes gutturalis*				2	13	2	14	3	11	5	18	3	15	2	16	4	14	6	20	3
Fringillidae	*Atlapetes pallidinucha*				2	20	3	29	3	15	4	29	4	28	6	39	7	42	12	34	11
Fringillidae	*Atlapetes rufinucha*				2	14	3	15	2	14	2	21	0	14	3	25	2x	25	2x	27	0x
Fringillidae	*Atlapetes melanolaemus*			x	14	0	21x	10	0x	10	4x	0	15x	14	21x	10	22x	20	18x	3	29x
Fringillidae	*Atlapetes leucopis*			x	2	25	0x	31	0x	31	0x	46	0x	39	7x	55	19x	71	2x	46	13x
Fringillidae	*Atlapetes pileatus*				2	8	7	24	2	12	5	32	1	13	8	26	11	18	9	36	3
Fringillidae	*Atlapetes melanocephalus*			x	2	11	0x	76	0x	87	0x	11	0x	87	0x	76	0x	87	0x	87	0x
Fringillidae	*Atlapetes flaviceps*	T		x	2	0	38x	0	38x	0	38x	0	38x	0	38x	0	38x	0	38x	0	38x
Fringillidae	*Atlapetes melanops*			x	14	0	0x	0	0x	0	0x	0	0x	0	0x	0	0x	0	0x	0	0x
Fringillidae	*Atlapetes fuscoolivaceus*			x	2	0	0x	0	0x	0	0x	0	0x	0	0x	0	0x	0	0x	0	0x
Fringillidae	*Atlapetes tricolor*				2	9	3	8	3	11	3	16	4	15	3	9	11	20	3	19	4
Fringillidae	*Atlapetes albofrenatus*			x	2	33	0x	24	6x	5	6x	37	6x	40	9x	32	23x	31	26x	46	8x
Fringillidae	*Atlapetes schistaceus*				2	22	2	21	3	17	3	29	6	27	4	28	7	33	7	30	9
Fringillidae	*Atlapetes canigenis*			x	14	0	30x	0	30x	0	30x	0	30x	0	30x	0	45x	24	30x	24	30x
Fringillidae	*Atlapetes seebohmi*			x	2	1	13x	7	12x	3	16x	6	13x	1	25x	7	25x	8	25x	8	23x
Fringillidae	*Atlapetes nationi*				2	7	52x	5	61x	9	53x	15	44	7	60x	9	68x	20	64x	30	48x
Fringillidae	*Atlapetes leucopterus*				2	11	12	6	8	11	13	21	4	15	12	23	13	29	18	23	9
Fringillidae	*Atlapetes albiceps*				2	0	21x	0	19x	0	21x	0	19x	0	21x	0	30x	0	32x	0	30x
Fringillidae	*Atlapetes pallidiceps*	T		x	2	83	0x	0	0x	14	0x	14	0x	97	0x	89	0x	89	0x	14	0x
Fringillidae	*Atlapetes rufigenis*			x	2	0	0x	30	0x	13	2x	21	0x	0	5x	30	5x	33	5x	33	5x
Fringillidae	*Atlapetes forbesi*			x	14	0	0x	0	0x	0	0x	0	0x	0	0x	0	0x	0	0x	0	0x
Fringillidae	*Atlapetes semirufus*				2	22	4	23	11	10	11	21	7	30	11	31	21	30	35	26	26
Fringillidae	*Atlapetes personatus*				2	10	22	9	27	5	28	2	31	16	29	14	38	10	42	4	50
Fringillidae	*Atlapetes fulviceps*				2	7	0	6	0	1	0	6	0	7	0	7	0	7	0	7	0
Fringillidae	*Atlapetes citrinellus*			x	2	0	0x	0	0x	0	0x	0	0x	0	0x	0	0x	0	0x	0	0x
Fringillidae	*Atlapetes brunneinucha*				2	12	4	14	4	11	5	19	4	17	5	19	8	23	8	24	6
Fringillidae	*Atlapetes virenticeps*				2	17	11	39	2	19	6	39	1	17	17	39	14	23	21	42	9
Fringillidae	*Atlapetes atricapillus*			x	2	24	7x	13	17x	13	17x	18	14x	30	14x	18	22x	14	29x	29	21x
Fringillidae	*Atlapetes torquatus*				2	10	8	11	4	9	7	16	7	14	10	18	11	20	13	20	9
Fringillidae	*Pezopetes capitalis*			x	2	15	24x	36	35x	7	35x	7	35x	15	24x	36	35x	54	35x	42	35x
Fringillidae	*Pselliophorus tibialis*			x	2	13	18x	29	25x	13	25x	16	25x	15	18x	29	28x	51	28x	41	25x
Fringillidae	*Pselliophorus luteoviridis*	T		x	2	45	0x	76	0x	0	0x	76	0x	69	0x	76	0x	0	0x	76	0x
Fringillidae	*Lysurus crassirostris*			x	2	24	1x	36	7x	29	7x	50	3x	29	3x	37	7x	50	9x	60	5x
Fringillidae	*Lysurus castaneiceps*				2	16	13	14	7	13	13	28	13	21	14	27	20	38	13	44	15

Family	Species	IUCN	Ag	R	Range Source	2050 TG H	2050 TG C	2050 AM H	2050 AM C	2050 GO H	2050 GO C	2050 OS H	2050 OS C	2100 TG H	2100 TG C	2100 AM H	2100 AM C	2100 GO H	2100 GO C	2100 OS H	2100 OS C
Fringillidae	*Gubernatrix cristata*	T			2	3	3	2	3	4	4	4	3	5	4	4	5	6	6	6	5
Fringillidae	*Paroaria coronata*				2,11	5	5	2	5	5	5	6	4	6	7	5	9	8	10	9	8
Fringillidae	*Paroaria dominicana*				2	5	15	4	12	7	13	13	7	6	18	7	13	15	15	22	7
Fringillidae	*Paroaria gularis*				2	7	10	5	10	6	9	11	9	12	11	10	12	19	12	21	11
Fringillidae	*Paroaria baeri*				2	7	51	0	31	0	38	0	35	7	70x	0	36	14	46	0	36
Fringillidae	*Paroaria capitata*				2,11	2	8	2	7	2	7	4	6	4	9	4	11	6	11	9	10
Fringillidae	*Vermivora bachmanii*	T			2	11	20	18	6	17	12	13	5	27	27	27	12	40	18	22	5
Fringillidae	*Vermivora pinus*				2	15	22	25	11	23	15	25	9	22	25	34	13	42	17	40	9
Fringillidae	*Vermivora chrysoptera*				2	19	30	32	15	30	22	30	10	26	32	42	17	47	24	47	11
Fringillidae	*Vermivora peregrina*				2	8	21	12	19	12	19	12	18	11	25	15	24	19	25	17	24
Fringillidae	*Vermivora celata*				2	1	19	1	18	1	19	1	18	1	25	2	27	3	29	3	26
Fringillidae	*Vermivora ruficapilla*				2	11	27	19	21	17	24	19	19	16	34	25	31	27	35	28	27
Fringillidae	*Vermivora virginiae*				2	0	8	1	8	0	8	0	7	0	12	2	17	3	20	4	15
Fringillidae	*Vermivora crissalis*				2	3	8	9	7	6	8	10	9	3	11	9	11	11	8	10	10
Fringillidae	*Vermivora luciae*				2	1	22	5	26	1	29	7	26	2	30	5	48	1	55	8	51
Fringillidae	*Parula americana*				2	14	25	22	14	20	19	21	11	22	29	31	19	38	24	30	13
Fringillidae	*Parula pitiayumi*				2	10	14	8	13	10	13	13	11	13	18	13	18	18	18	21	15
Fringillidae	*Parula superciliosa*				2	14	9	26	4	15	8	33	2	17	11	26	14	20	13	35	4
Fringillidae	*Parula gutturalis*			x	2	12	21x	33	31x	10	31x	12	31x	14	21x	33	32x	56	32x	46	31x
Fringillidae	*Dendroica petechia*				2	11	7	17	6	13	7	23	3	16	9	20	13	18	10	28	5
Fringillidae	*Dendroica aestiva*				14	4	18	7	15	6	17	8	14	6	23	10	22	11	24	12	19
Fringillidae	*Dendroica pensylvanica*				2	14	26	22	20	20	22	21	17	20	32	29	27	32	30	32	24
Fringillidae	*Dendroica magnolia*				2	9	21	13	17	13	18	14	15	12	25	18	23	20	24	20	21
Fringillidae	*Dendroica tigrina*				2	7	21	10	18	10	19	10	17	9	25	13	24	16	26	15	23
Fringillidae	*Dendroica caerulescens*				2	21	27	32	20	30	22	34	17	29	31	44	27	49	28	50	23
Fringillidae	*Dendroica coronata*				2	4	19	7	17	6	18	7	15	6	23	9	24	10	26	11	22
Fringillidae	*Dendroica nigrescens*				2	3	16	5	14	5	15	6	12	4	20	7	23	8	27	9	21
Fringillidae	*Dendroica townsendi*				2	5	28	10	25	8	27	12	24	7	37	14	37	15	41	19	33
Fringillidae	*Dendroica occidentalis*				2	7	36	11	25	13	29	14	18	7	41	14	38	19	43	23	25
Fringillidae	*Dendroica virens*				2	11	22	17	16	16	19	17	15	15	26	23	22	25	24	25	20
Fringillidae	*Dendroica chrysoparia*	T			2	0	0	0	0	0	0	0	0	0	0	0	0	0	0	0	0
Fringillidae	*Dendroica fusca*				2	15	27	24	20	22	23	24	17	21	32	33	28	36	30	36	24
Fringillidae	*Dendroica dominica*				2	11	21	19	8	16	14	16	5	20	26	26	13	36	20	24	6
Fringillidae	*Dendroica graciae*				2	9	8	17	6	11	8	22	5	11	10	18	18	15	23	25	17
Fringillidae	*Dendroica adelaidae*			x	2	23	0x	23	0x	23	0x	23	0x	23	0x	23	0x	23	0x	23	0x
Fringillidae	*Dendroica delicata*			x	14	0	46x	37	46x	46	0x	46	0x	0	46x	37	46x	46	0x	83	0x
Fringillidae	*Dendroica subita*			x	14	0	0x	100	0x	0	0x	100	0x	0	0x	100	0x	100	0x	100	0x
Fringillidae	*Dendroica pityophila*			x	2	14	0x	66	1x	26	1x	53	0x	50	0x	81	1x	27	1x	55	0x
Fringillidae	*Dendroica pinus*				2	16	27	27	15	25	20	27	11	25	30	36	19	45	24	40	13
Fringillidae	*Dendroica kirtlandii*	T		x	2	0	102x	5	95x	5	95x	91	9x	0	102x	5	95x	5	95x	91	9x
Fringillidae	*Dendroica discolor*				2	15	29	24	13	21	20	22	10	22	34	34	18	42	25	35	10
Fringillidae	*Dendroica palmarum*				2	6	17	8	15	8	15	8	14	7	21	10	21	13	21	12	20
Fringillidae	*Dendroica castanea*				2	7	16	10	15	9	15	10	15	9	21	13	21	16	22	15	21
Fringillidae	*Dendroica striata*				2	2	20	2	19	3	19	3	19	2	24	3	27	4	27	4	26

Family	Species	IUCN	Ag	R	Range Source	Percentage loss in geographic range 2050 TG H	TG C	AM H	AM C	GO H	GO C	OS H	OS C	2100 TG H	TG C	AM H	AM C	GO H	GO C	OS H	OS C
Fringillidae	*Dendroica cerulea*	T			2	19	23	27	10	25	16	28	7	26	26	39	13	45	19	44	7
Fringillidae	*Dendroica plumbea*			x	2	15	0x	38	0x	15	0x	38	0x	15	0x	38	0x	38	0x	38	0x
Fringillidae	*Dendroica pharetra*			x	2	17	0x	17	5x	11	5x	44	5x	44	0x	17	5x	11	5x	44	5x
Fringillidae	*Dendroica angelae*	T		x	2	23	0x	23	0x	23	0x	23	0x	23	0x	23	0x	23	0x	23	0x
Fringillidae	*Catharopeza bishopi*	T		x	2	0	0x	100	0x	0	0x	0	0x	0	0x	100	0x	0	0x	100	0x
Fringillidae	*Mniotilta varia*				2	10	22	16	16	16	18	17	14	16	26	22	22	27	24	25	19
Fringillidae	*Setophaga ruticilla*				2	10	23	15	18	14	20	16	16	15	29	21	25	26	27	25	22
Fringillidae	*Protonotaria citrea*				2	9	18	16	7	14	12	13	4	17	22	22	10	30	17	20	5
Fringillidae	*Helmitheros vermivorus*				2	18	25	26	12	24	15	27	8	26	28	37	15	46	19	43	9
Fringillidae	*Limnothlypis swainsonii*		H		2	0	22	0	7	0	13	0	4	0	28	0	13	0	20	0	5
Fringillidae	*Seiurus aurocapillus*				2	11	24	17	18	16	20	18	15	15	28	22	23	27	25	26	19
Fringillidae	*Seiurus noveboracensis*				2	5	21	7	19	7	20	7	18	6	27	10	27	11	28	11	25
Fringillidae	*Seiurus motacilla*				2	15	21	24	10	23	13	25	7	26	25	34	13	46	17	40	8
Fringillidae	*Oporornis formosus*				2	14	21	23	8	20	14	23	7	25	26	31	12	45	19	35	7
Fringillidae	*Oporornis agilis*				2	4	27	8	22	8	25	7	20	5	35	11	33	13	36	13	30
Fringillidae	*Oporornis philadelphia*				2	11	23	15	18	16	20	16	16	15	28	20	24	24	26	23	21
Fringillidae	*Oporornis tolmiei*				2	3	21	5	18	4	20	6	16	4	26	7	28	9	32	12	24
Fringillidae	*Geothlypis trichas*		H		2	0	18	0	13	0	16	0	11	0	22	0	18	0	22	0	15
Fringillidae	*Geothlypis beldingi*	T			2	0	1	0	1	0	0	4	0	0	1	0	1	0	0	4	0
Fringillidae	*Geothlypis flavovelata*	T			2	0	2	0	2	0	2	0	2	0	5	0	33	0	2	0	2
Fringillidae	*Geothlypis rostrata*			x	2	28	10x	78	4x	69	10x	53	0x	43	10x	78	7x	85	10x	80	0x
Fringillidae	*Geothlypis semiflava*		H		2	0	4	0	6	0	5	0	1	0	4	0	7	0	6	0	1
Fringillidae	*Geothlypis speciosa*	T		x	2	4	13x	13	4x	4	13x	24	0x	4	13x	13	4x	10	13x	25	0x
Fringillidae	*Geothlypis nelsoni*				2	2	3	8	0	3	0	4	0	2	3	8	9	5	0	4	0
Fringillidae	*Geothlypis aequinoctialis*				2	16	5	11	5	14	4	20	3	20	6	18	7	31	7	32	5
Fringillidae	*Geothlypis chiriquensis*		H	x	2	0	0x	0	83x	0	83x	0	83x	0	0x	0	83x	0	83x	0	83x
Fringillidae	*Geothlypis auricularis*				2	1	5	2	3	9	5	12	2	1	6	9	6	11	11	13	3
Fringillidae	*Geothlypis velata*				2	7	13	5	9	8	11	11	8	11	17	10	13	15	15	17	12
Fringillidae	*Geothlypis poliocephala*		H		2	0	2	0	2	0	3	0	2	0	3	0	15	0	5	0	2
Fringillidae	*Microligea palustris*			x	2	0	4x	3	1x	0	4x	4	0x	0	4x	3	1x	0	4x	4	0x
Fringillidae	*Teretistris fernandinae*			x	2	1	16x	33	15x	26	2x	59	2x	12	16x	40	16x	27	2x	59	2x
Fringillidae	*Teretistris fornsi*			x	2	12	15x	21	18x	4	19x	25	12x	20	15x	23	21x	9	20x	30	12x
Fringillidae	*Leucopeza semperi*	T		x	2	0	46x	37	46x	46	0x	46	0x	0	46x	37	46x	46	0x	83	0x
Fringillidae	*Wilsonia citrina*				2	18	24	26	12	24	16	28	9	28	28	39	15	46	20	44	9
Fringillidae	*Wilsonia pusilla*				2	3	21	4	20	4	20	5	19	4	27	6	30	7	31	8	28
Fringillidae	*Wilsonia canadensis*				2	10	21	15	17	15	19	16	16	14	27	21	24	23	26	23	22
Fringillidae	*Cardellina rubrifrons*				2	8	13	25	9	9	13	35	11	13	17	26	23	14	31	42	25
Fringillidae	*Ergaticus ruber*				2	9	8	26	2	12	6	34	1	11	8	30	7	20	10	37	4
Fringillidae	*Ergaticus versicolor*	T			2	8	0	15	0	10	0	16	0	10	0	16	1	13	1	16	0
Fringillidae	*Myioborus pictus*				2	8	9	18	6	9	9	24	6	10	12	19	19	13	22	26	17
Fringillidae	*Myioborus miniatus*				2	14	8	18	7	13	9	24	7	18	10	22	14	22	14	28	11
Fringillidae	*Myioborus castaneocapillu*				2	6	4	3	9	4	7	3	9	10	9	7	14	10	11	6	18
Fringillidae	*Myioborus pariae*	T		x	2	0	0x	0	0x	0	0x	0	0x	0	0x	0	0x	0	0x	0	0x
Fringillidae	*Myioborus brunniceps*				2	5	2	6	1	1	2	5	1	5	2	6	2	5	3	6	3

Family	Species	IUCN	Ag	R	Range Source	Percentage loss in geographic range 2050 TG		AM		GO		OS		2100 TG		AM		GO		OS	
						H	C	H	C	H	C	H	C	H	C	H	C	H	C	H	C
Fringillidae	*Myioborus albifacies*			x	2	4	2x	0	5x	4	2x	0	5x	4	2x	0	5x	4	2x	0	5x
Fringillidae	*Myioborus cardonai*			x	14	0	85x	0	85x	0	85x	0	85x	0	85x	0	85x	0	85x	15	85x
Fringillidae	*Myioborus torquatus*			x	2	15	16x	37	23x	8	23x	21	23x	18	16x	37	24x	42	24x	47	23x
Fringillidae	*Myioborus melanocephalus*				2	17	6	15	2	13	5	21	7	23	8	27	10	38	10	26	12
Fringillidae	*Myioborus ornatus*				2	16	4	23	6	7	6	25	6	28	4	29	6	24	9	26	9
Fringillidae	*Myioborus albifrons*			x	2	24	0x	19	0x	23	0x	62	0x	24	21x	24	32x	41	41x	75	0x
Fringillidae	*Myioborus flavivertex*			x	2	10	0x	47	0x	57	0x	10	0x	57	0x	47	0x	57	0x	57	0x
Fringillidae	*Euthlypis lachrymosa*				2	11	9	22	5	14	9	30	6	16	11	22	15	20	19	34	11
Fringillidae	*Basileuterus fraseri*				2	4	6	5	4	6	7	8	1	4	6	6	7	7	9	9	3
Fringillidae	*Basileuterus bivittatus*				2	19	9	11	5	11	7	25	5	20	9	19	6	23	8	30	5
Fringillidae	*Basileuterus roraimae*				14	11	14	7	19	4	21	1	23	13	18	12	22	8	26	8	32
Fringillidae	*Basileuterus chrysogaster*				2	24	17	5	9	15	16	36	18	34	17	15	28	37	20	41	22
Fringillidae	*Basileuterus chlorophrys*			x	2	9	6x	10	5x	18	5x	16	0x	16	6x	11	5x	19	5x	24	0x
Fringillidae	*Basileuterus signatus*				2	13	4	15	1	11	2	16	4	14	4	20	3	21	3	19	5
Fringillidae	*Basileuterus luteoviridis*				2	15	5	16	3	11	4	18	6	20	7	22	9	25	9	22	9
Fringillidae	*Basileuterus nigrocristatus*				2	17	7	21	5	16	9	26	8	23	11	31	12	36	14	31	13
Fringillidae	*Basileuterus griseiceps*	T		x	2	0	0x	0	0x	0	0x	0	0x	0	0x	3	0x	3	0x	3	0x
Fringillidae	*Basileuterus basilicus*	T		x	2	15	0x	60	0x	75	0x	15	0x	75	0x	60	0x	75	0x	75	0x
Fringillidae	*Basileuterus cinereicollis*			x	2	17	0x	26	5x	6	5x	33	5x	28	7x	31	15x	43	16x	39	9x
Fringillidae	*Basileuterus conspicillatus*			x	2	25	0x	59	0x	84	0x	25	0x	84	0x	59	0x	84	0x	84	0x
Fringillidae	*Basileuterus coronatus*				2	18	5	16	4	13	6	25	7	24	7	23	10	29	10	29	9
Fringillidae	*Basileuterus culicivorus*				2	10	19	9	19	9	19	14	17	14	22	13	25	17	24	21	22
Fringillidae	*Basileuterus trifasciatus*			x	2	4	19x	1	13x	0	20x	0	14x	4	19x	1	19x	0	26x	0	20x
Fringillidae	*Basileuterus hypoleucus*				2	5	17	3	16	5	21	14	13	12	24	10	24	20	28	26	21
Fringillidae	*Basileuterus rufifrons*				2	13	8	26	3	14	6	31	3	17	9	27	15	19	14	36	7
Fringillidae	*Basileuterus delattrii*				2	12	1	16	2	14	4	22	1	13	1	17	4	17	10	25	2
Fringillidae	*Basileuterus belli*				2	10	7	23	2	13	5	30	2	11	9	25	10	19	10	33	4
Fringillidae	*Basileuterus melanogenys*			x	2	16	17x	40	25x	9	25x	23	25x	22	17x	40	26x	46	26x	50	25x
Fringillidae	*Basileuterus ignotus*	T		x	2	36	0x	8	36x	0	36x	36	0x	36	8x	8	36x	0	44x	92	8x
Fringillidae	*Basileuterus tristriatus*				2	16	5	15	5	15	5	27	5	23	6	23	11	30	9	33	7
Fringillidae	*Basileuterus leucoblepharu*				2	18	6	15	6	23	5	25	5	27	10	27	10	38	7	40	9
Fringillidae	*Basileuterus leucophrys*				2	8	15	4	9	5	15	14	4	11	20	14	12	15	17	21	6
Fringillidae	*Basileuterus flaveolus*				2	9	22	5	20	6	22	12	17	12	28	9	24	14	26	20	20
Fringillidae	*Basileuterus fulvicauda*				2	6	4	6	4	6	4	10	4	11	5	12	5	13	6	19	4
Fringillidae	*Basileuterus rivularis*				2	7	12	5	12	6	13	11	12	13	15	10	15	23	16	22	15
Fringillidae	*Zeledonia coronata*			x	2	16	15x	38	22x	10	22x	23	22x	22	15x	38	25x	45	24x	49	22x
Fringillidae	*Icteria virens*				2	5	18	9	13	7	16	10	11	9	22	12	20	15	25	15	18
Fringillidae	*Granatellus venustus*				2	9	4	21	2	10	4	30	2	10	8	21	18	17	8	31	2
Fringillidae	*Granatellus sallaei*				2	7	4	17	3	11	4	26	3	9	5	22	12	16	10	31	5
Fringillidae	*Granatellus pelzelni*				2	5	16	4	16	4	17	8	16	10	18	7	19	17	20	17	18
Fringillidae	*Xenoligea montana*	T		x	2	2	0x	2	0x	2	0x	2	0x	2	0x	2	0x	2	0x	2	0x
Fringillidae	*Coereba flaveola*				2	9	13	7	11	9	12	14	9	14	16	13	15	20	16	24	12
Fringillidae	*Conirostrum speciosum*				2	7	11	5	10	7	11	11	9	11	14	11	14	17	16	21	13
Fringillidae	*Conirostrum leucogenys*				2	15	5	14	7	12	9	15	4	18	8	18	11	21	18	17	11

Family	Species	IUCN	Ag	R	Range Source	Percentage loss in geographic range 2050 TG H	TG C	AM H	AM C	GO H	GO C	OS H	OS C	2100 TG H	TG C	AM H	AM C	GO H	GO C	OS H	OS C
Fringillidae	*Conirostrum bicolor*				2	7	7	7	5	6	7	10	4	12	8	12	8	16	10	20	6
Fringillidae	*Conirostrum margaritae*				2	12	9	9	3	3	8	13	3	23	9	24	3	20	8	31	3
Fringillidae	*Conirostrum cinereum*				2	6	22	8	21	7	24	9	19	7	30	13	34	20	32	17	30
Fringillidae	*Conirostrum tamarugense*	T	H	x	2	0	0x	0	0x	0	0x	0	0x	0	0x	0	3x	0	66x	0	3x
Fringillidae	*Conirostrum ferrugineiventr*				2	3	11	6	2	6	2	2	6	4	11	7	7	14	3	5	7
Fringillidae	*Conirostrum rufum*			x	2	26	0x	56	0x	32	0x	36	0x	43	0x	61	0x	44	17x	55	6x
Fringillidae	*Conirostrum sitticolor*				2	11	5	14	4	11	6	19	7	16	8	21	10	26	12	23	10
Fringillidae	*Conirostrum albifrons*				2	13	5	15	3	12	5	18	6	19	6	20	9	27	8	23	9
Fringillidae	*Oreomanes fraseri*				2	6	17	10	20	10	16	14	14	7	25	17	27	24	22	20	20
Fringillidae	*Orchesticus abeillei*				2	28	15	19	18	24	9	25	18	30	24x	31	26x	43	13x	33	26x
Fringillidae	*Schistochlamys ruficapillus*				2	7	16	5	7	7	10	11	5	9	23	9	9	13	13	17	7
Fringillidae	*Schistochlamys melanopis*				2	8	17	5	15	7	16	12	13	12	20	10	19	19	20	20	17
Fringillidae	*Neothraupis fasciata*				2	6	17	3	7	4	11	9	4	8	23	7	9	8	14	13	6
Fringillidae	*Cypsnagra hirundinacea*				2	5	18	2	9	3	14	8	7	8	24	5	11	8	16	14	9
Fringillidae	*Conothraupis speculigera*				2	3	21	0	20	0	24	0	20	3	22	0	27	2	31	2	27
Fringillidae	*Conothraupis mesoleuca*	T		x	2	0	0x	0	0x	0	0x	0	0x	0	0x	0	0x	0	0x	0	0x
Fringillidae	*Lamprospiza melanoleuca*				2	6	10	4	10	5	10	10	9	11	11	8	12	19	14	21	11
Fringillidae	*Cissopis leveriana*				2	8	9	6	9	7	8	13	8	13	11	12	11	20	11	23	10
Fringillidae	*Chlorornis riefferii*				2	10	5	11	2	8	3	15	4	15	5	17	8	21	6	18	7
Fringillidae	*Compsothraupis loricata*				2	7	19	5	14	6	15	12	8	8	23	9	15	13	18	20	11
Fringillidae	*Sericossypha albocristata*				2	14	5	16	3	9	5	25	7	25	5	24	10	35	11	32	10
Fringillidae	*Nesospingus speculiferus*			x	2	16	0x	16	0x	16	0x	16	0x	16	0x	16	0x	16	0x	16	0x
Fringillidae	*Chlorospingus ophthalmicu*				2	15	4	14	3	11	4	21	3	19	4	20	7	22	8	25	5
Fringillidae	*Chlorospingus tacarcunae*			x	2	20	13x	2	33x	35	33x	25	13x	20	15x	2	33x	60	35x	25	15x
Fringillidae	*Chlorospingus inornatus*			x	2	0	0x	0	0x	0	0x	0	0x	0	0x	0	0x	0	0x	90	0x
Fringillidae	*Chlorospingus semifuscus*			x	2	4	7x	9	10x	4	7x	17	3x	11	7x	11	10x	5	10x	17	3x
Fringillidae	*Chlorospingus pileatus*			x	2	16	21x	36	30x	7	30x	14	30x	17	21x	36	31x	48	31x	44	30x
Fringillidae	*Chlorospingus parvirostris*				2	21	10	13	6	13	10	27	9	26	11	26	17	37	17	33	15
Fringillidae	*Chlorospingus flavigularis*				2	15	9	13	7	15	9	25	6	21	9	20	12	30	10	33	7
Fringillidae	*Chlorospingus flavovirens*	T		x	14	11	5x	12	5x	21	5x	18	0x	14	5x	12	5x	23	5x	26	0x
Fringillidae	*Chlorospingus canigularis*				2	13	8	12	7	13	9	24	9	24	9	20	14	35	11	35	11
Fringillidae	*Cnemoscopus rubrirostris*				2	19	2	16	3	14	4	22	7	25	4	27	8	30	7	24	9
Fringillidae	*Hemispingus atropileus*				2	19	7	22	7	12	9	27	8	24	9	35	10	38	14	30	13
Fringillidae	*Hemispingus auricularis*				14	5	0	4	0	5	0	8	4	9	0	4	9	14	4	11	4
Fringillidae	*Hemispingus calophrys*				2	8	0	8	0	8	0	18	0	16	0	18	7	18	7	18	7
Fringillidae	*Hemispingus parodii*			x	2	0	0x	0	0x	0	0x	0	0x	0	0x	0	36x	36	0x	36	0x
Fringillidae	*Hemispingus superciliaris*				2	13	6	15	4	12	6	20	8	18	7	20	10	27	11	23	11
Fringillidae	*Hemispingus reyi*			x	2	29	0x	9	3x	10	3x	44	3x	29	10x	9	35x	30	35x	49	4x
Fringillidae	*Hemispingus frontalis*				2	10	4	9	4	9	4	17	5	14	5	17	10	26	9	22	7
Fringillidae	*Hemispingus melanotis*				2	15	3	18	2	13	3	21	5	23	3	24	6	29	7	24	8
Fringillidae	*Hemispingus ochraceus*			x	14	0	70x	30	70x	30	70x	100	0x	0	70x	30	70x	30	70x	100	0x
Fringillidae	*Hemispingus piurae*			x	14	1	1x	0	2x	0	2x	0	2x	1	1x	0	2x	0	2x	0	2x
Fringillidae	*Hemispingus goeringi*	T		x	2	25	0x	29	6x	0	6x	48	6x	25	29x	29	51x	37	43x	73	6x
Fringillidae	*Hemispingus rufosupercilia*	T		x	14	0	0x	0	0x	1	0x	2	0x	1	0x	0	2x	3	2x	3	0x

Family	Species	IUCN	Ag	R	Range Source	2050 TG H	2050 TG C	2050 AM H	2050 AM C	2050 GO H	2050 GO C	2050 OS H	2050 OS C	2100 TG H	2100 TG C	2100 AM H	2100 AM C	2100 GO H	2100 GO C	2100 OS H	2100 OS C
Fringillidae	*Hemispingus verticalis*				2	24	4	33	3	18	7	30	7	31	11	55	9	56	12	34	16
Fringillidae	*Hemispingus xanthophthal*				2	8	4	8	0	10	0	14	4	13	4	13	8	20	5	16	6
Fringillidae	*Hemispingus trifasciatus*				2	4	4	7	0	7	0	10	4	7	4	14	4	16	3	11	7
Fringillidae	*Pyrrhocoma ruficeps*				2	20	8	19	7	27	5	28	6	27	18	31	18	46	11	49	13
Fringillidae	*Thlypopsis fulviceps*				2	19	0	10	5	9	5	27	5	25	7	21	18	26	19	32	7
Fringillidae	*Thlypopsis ornata*				2	10	6	11	5	9	7	14	5	12	10	19	8	23	10	18	8
Fringillidae	*Thlypopsis pectoralis*			x	2	0	5x	0	5x	0	0x	0	0x	0	5x	0	5x	0	0x	0	0x
Fringillidae	*Thlypopsis sordida*				2	7	16	5	13	7	15	12	11	11	20	9	18	15	19	20	15
Fringillidae	*Thlypopsis inornata*			x	2	0	2x	2	0x	0	2x	0	2x	0	2x	2	0x	0	2x	0	2x
Fringillidae	*Thlypopsis ruficeps*				2	4	10	7	5	5	6	8	5	5	10	8	7	10	6	9	5
Fringillidae	*Hemithraupis guira*				2	7	13	5	13	6	13	11	12	12	15	10	16	18	17	22	14
Fringillidae	*Hemithraupis ruficapilla*				2	21	9	14	13	27	8	22	11	29	19	24	23	42	13	36	21
Fringillidae	*Hemithraupis flavicollis*				2	4	11	3	10	3	11	6	11	7	12	7	13	10	14	12	13
Fringillidae	*Chrysothlypis chrysomelas*			x	2	20	4x	34	9x	19	9x	40	4x	29	5x	36	10x	39	11x	57	6x
Fringillidae	*Chrysothlypis salmoni*				2	14	2	14	5	17	0	8	5	27	2	14	5	21	5	23	5
Fringillidae	*Nemosia pileata*				2	8	15	5	14	6	15	12	12	11	18	10	17	17	18	20	15
Fringillidae	*Nemosia rourei*	T		x	2	9	2x	9	2x	2	0x	30	2x	9	2x	9	2x	30	0x	45	2x
Fringillidae	*Phaenicophilus palmarum*				2	15	12	21	10	16	14	32	0	15	15	21	11	16	14	32	0
Fringillidae	*Phaenicophilus poliocephal*				2	24	24x	42	7x	31	18x	48	0x	24	24x	42	7x	31	18x	48	0x
Fringillidae	*Calyptophilus frugivorus*	T		x	2	1	6x	1	0x	1	6x	7	0x	1	6x	1	6x	1	6x	7	0x
Fringillidae	*Calyptophilus tertius*			x	14	3	0x	3	0x	3	0x	3	0x	3	0x	3	0x	3	0x	3	0x
Fringillidae	*Rhodinocichla rosea*				2	20	3	20	6	16	8	15	7	22	7	25	16	17	15	18	17
Fringillidae	*Mitrospingus cassinii*				2	18	5	20	9	17	7	22	5	25	7	22	11	25	12	34	8
Fringillidae	*Mitrospingus oleagineus*				2	9	1	2	10	12	2	2	10	10	12	12	20	19	14	10	31
Fringillidae	*Chlorothraupis carmioli*				2	28	2	46	6	38	7	58	2	38	3	55	9	53	7	63	3
Fringillidae	*Chlorothraupis frenata*				2	29	7	21	5	23	7	41	6	39	7	33	10	42	8	49	7
Fringillidae	*Chlorothraupis olivacea*				2	9	2	11	4	11	0	5	4	19	3	12	4	15	5	20	5
Fringillidae	*Chlorothraupis stolzmanni*				2	17	3	14	7	21	4	18	3	23	3	15	7	22	7	27	3
Fringillidae	*Orthogonys chloricterus*				2	26	15	12	28	27	17	22	24	26	43	24	48	52	25	35	43
Fringillidae	*Eucometis penicillata*				2	8	11	7	11	7	12	13	10	13	14	12	14	20	15	23	12
Fringillidae	*Lanio fulvus*				2	3	9	3	8	3	9	6	8	7	10	7	10	10	12	12	11
Fringillidae	*Lanio versicolor*				2	6	9	5	9	5	10	10	9	11	11	10	11	16	11	20	10
Fringillidae	*Lanio aurantius*				2	11	6	20	4	12	6	27	4	13	7	25	11	20	9	34	6
Fringillidae	*Lanio leucothorax*				2	18	3	35	4	32	4	46	4	28	3	46	8	45	6	54	4
Fringillidae	*Creurgops verticalis*				2	12	5	12	3	12	5	20	8	19	6	20	14	31	8	25	11
Fringillidae	*Creurgops dentata*				2	10	5	7	1	9	1	17	4	16	5	16	9	23	7	19	10
Fringillidae	*Heterospingus rubrifrons*			x	2	20	9x	37	9x	27	9x	49	9x	25	9x	42	9x	54	9x	54	9x
Fringillidae	*Heterospingus xanthopygiu*				2	14	3	14	4	16	2	11	3	23	5	16	4	20	6	25	6
Fringillidae	*Tachyphonus cristatus*				2	5	13	3	13	4	13	8	12	9	15	7	16	15	16	15	15
Fringillidae	*Tachyphonus rufiventer*				2	6	2	7	1	7	2	13	1	13	2	15	2	14	2	24	1
Fringillidae	*Tachyphonus surinamus*				2	5	9	3	8	4	8	7	8	8	9	7	10	13	11	15	10
Fringillidae	*Tachyphonus luctuosus*				2	6	12	5	13	5	13	10	12	11	14	9	15	17	16	19	14
Fringillidae	*Tachyphonus delatrii*				2	22	3	29	6	29	5	35	3	33	4	35	8	37	8	45	5
Fringillidae	*Tachyphonus coronatus*				2	20	8	17	8	25	6	27	7	29	16	28	15	44	10	46	13

Family	Species	IUCN	Ag	R	Range Source	2050 TG H	2050 TG C	2050 AM H	2050 AM C	2050 GO H	2050 GO C	2050 OS H	2050 OS C	2100 TG H	2100 TG C	2100 AM H	2100 AM C	2100 GO H	2100 GO C	2100 OS H	2100 OS C
Fringillidae	*Tachyphonus rufus*				2	9	14	6	11	7	13	15	8	13	17	11	14	19	16	25	11
Fringillidae	*Tachyphonus phoenicius*				2	5	14	4	15	3	15	7	15	9	17	7	18	13	18	13	18
Fringillidae	*Trichothraupis melanops*				2	16	10	14	7	20	7	24	6	23	15	24	13	36	10	38	10
Fringillidae	*Habia rubica*				2	7	8	6	8	6	8	11	8	12	9	11	11	17	10	20	10
Fringillidae	*Habia fuscicauda*				2	12	4	22	4	17	5	30	3	15	5	28	11	23	8	36	5
Fringillidae	*Habia gutturalis*				2	17	2x	14	4x	14	6x	23	0x	20	2x	21	6x	20	6x	23	2x
Fringillidae	*Habia atrimaxillaris*	T		x	2	54	0x	54	0x	54	0x	54	0x	54	0x	54	40x	54	0x	54	0x
Fringillidae	*Habia cristata*			x	2	26	0x	33	2x	8	0x	41	2x	43	0x	33	2x	8	2x	41	2x
Fringillidae	*Piranga bidentata*		H		2	0	9	0	6	0	9	0	5	0	10	0	14	0	14	0	7
Fringillidae	*Piranga flava*		H		2	0	13	0	9	0	11	0	8	0	18	0	14	0	15	0	12
Fringillidae	*Piranga hepatica*				14	8	9	16	8	10	10	21	7	10	12	16	22	12	24	23	18
Fringillidae	*Piranga lutea*				14	11	12	9	13	9	15	13	12	14	16	12	20	16	21	17	18
Fringillidae	*Piranga rubra*				2	8	21	14	12	11	16	14	10	15	25	20	20	27	25	21	15
Fringillidae	*Piranga roseogularis*				2	8	3	19	0	13	3	27	3	12	3	23	11	16	13	37	6
Fringillidae	*Piranga olivacea*		H		2	0	21	0	11	0	15	0	7	0	23	0	13	0	17	0	8
Fringillidae	*Piranga ludoviciana*				2	2	18	4	15	3	17	4	15	3	22	6	24	7	27	9	22
Fringillidae	*Piranga leucoptera*				2	15	3	13	4	12	4	22	4	19	6	19	12	22	10	27	8
Fringillidae	*Piranga erythrocephala*				2	13	11	32	5	17	10	43	5	21	14	32	14	26	20	48	10
Fringillidae	*Piranga rubriceps*				2	10	7	12	5	8	8	18	8	14	8	21	12	25	13	22	11
Fringillidae	*Calochaetes coccineus*				2	14	4	11	3	13	4	22	6	22	4	20	11	33	8	28	9
Fringillidae	*Phlogothraupis sanguinole*				2	20	3	33	4	24	5	42	2	26	3	40	10	36	6	47	3
Fringillidae	*Ramphocelus nigrogularis*				2	5	6	4	6	4	6	8	6	9	7	11	7	13	8	18	6
Fringillidae	*Ramphocelus dimidiatus*				2,11	12	3	13	5	9	5	17	2	14	5	17	8	15	12	19	5
Fringillidae	*Ramphocelus melanogaste*		H		2	0	15	0	15	0	15	0	0	0	15	0	18	0	15	0	0
Fringillidae	*Ramphocelus carbo*				2	7	13	4	11	5	12	10	9	11	15	9	14	15	15	19	12
Fringillidae	*Ramphocelus bresilius*		H		2	0	20	0	21	0	18	0	19	0	26	0	31	0	24	0	29
Fringillidae	*Ramphocelus passerinii*				2	17	5	29	6	22	6	39	3	22	6	37	11	34	8	44	5
Fringillidae	*Ramphocelus costaricensis*			x	14	36	10x	36	23x	24	23x	40	23x	47	10x	36	38x	30	33x	61	23x
Fringillidae	*Ramphocelus flammigerus*				2	1	0x	8	0x	0	0	8	0x	3	0x	9	0x	7	0x	8	0x
Fringillidae	*Ramphocelus icteronotus*				14	14	6	16	7	13	7	17	5	20	8	18	8	20	10	27	7
Fringillidae	*Spindalis zena*				2	13	14	34	14x	23	10	44	6x	24	14	38	16x	28	11	49	6x
Fringillidae	*Spindalis dominicensis*			x	14	9	7x	14	3x	10	7x	19	0x	9	7x	14	5x	10	7x	19	0x
Fringillidae	*Spindalis nigricephala*			x	14	17	0x	17	5x	11	5x	44	5x	44	0x	17	5x	11	5x	44	5x
Fringillidae	*Spindalis portoricensis*			x	14	23	0x	23	0x	23	0x	23	0x	23	0x	23	0x	23	0x	23	0x
Fringillidae	*Thraupis episcopus*				2	7	7	6	7	6	7	12	6	11	8	11	10	17	11	21	9
Fringillidae	*Thraupis glaucocolpa*				2	23	4	20	5	22	5	22	3	28	9	28	7	35	11	32	11
Fringillidae	*Thraupis sayaca*				2	9	14	6	11	8	12	13	9	12	19	11	16	17	17	21	13
Fringillidae	*Thraupis cyanoptera*				2	21	10	9	18	27	10	20	15	23	25	23	31	45	16	32	28
Fringillidae	*Thraupis ornata*				2	12	10	10	12	19	7	15	10	17	16	13	23	31	14	23	18
Fringillidae	*Thraupis abbas*				2	8	3	18	2	14	3	24	2	10	4	21	9	19	7	27	4
Fringillidae	*Thraupis palmarum*				2	8	13	6	11	7	12	12	9	12	16	10	14	17	15	20	12
Fringillidae	*Thraupis cyanocephala*				2	12	6	13	4	11	6	17	6	17	7	19	9	24	9	21	8
Fringillidae	*Thraupis bonariensis*				2	6	7	5	7	6	7	8	6	8	10	8	11	12	11	12	9
Fringillidae	*Cyanicterus cyanicterus*				2	3	22	3	20	4	22	6	20	6	24	7	27	11	29	10	26

Percentage loss in geographic range

Family	Species	IUCN	Ag	R	Range Source	Percentage loss in geographic range 2050 TG H	TG C	AM H	AM C	GO H	GO C	OS H	OS C	2100 TG H	TG C	AM H	AM C	GO H	GO C	OS H	OS C
Fringillidae	*Bangsia arcaei*			x	2	21	2x	38	2x	28	2x	49	2x	30	2x	41	3x	44	2x	56	2x
Fringillidae	*Bangsia melanochlamys*	T		x	14	0	0x	41	0x	0	0x	41	0x	41	0x	41	0x	0	0x	41	0x
Fringillidae	*Bangsia rothschildi*			x	2	11	0x	14	1x	10	0x	14	1x	26	0x	14	1x	10	1x	19	1x
Fringillidae	*Bangsia edwardsi*			x	2	14	0x	9	0x	21	0x	9	0x	17	0x	9	0x	22	0x	21	0x
Fringillidae	*Bangsia aureocincta*	T		x	2	0	0x	59	0x	0	0x	59	0x	38	0x	59	0x	21	0x	59	0x
Fringillidae	*Buthraupis montana*				2	13	5	17	4	11	5	21	6	20	6	24	9	27	9	24	9
Fringillidae	*Buthraupis eximia*			x	2	22	9x	33	7x	21	13x	35	12x	32	18x	49	15x	55	16x	43	21x
Fringillidae	*Buthraupis aureodorsalis*	T		x	2	0	0x	0	0x	0	0x	0	0x	0	0x	0	0x	0	0x	0	0x
Fringillidae	*Buthraupis wetmorei*	T		x	2	24	11x	34	6x	19	16x	29	16x	31	18x	48	17x	52	21x	37	27x
Fringillidae	*Wetmorethraupis sterrhopt*	T		x	2	0	14x	0	14x	29	14x	29	14x	0	14x	25	14x	29	14x	29	14x
Fringillidae	*Anisognathus melanogeny*			x	2	12	0x	55	0x	68	0x	12	0x	68	0x	55	0x	68	0x	68	0x
Fringillidae	*Anisognathus lacrymosus*				2	18	3	14	4	10	5	20	6	24	5	24	12	30	11	23	9
Fringillidae	*Anisognathus igniventris*				2	12	5	16	3	10	4	19	5	18	6	23	7	30	7	21	9
Fringillidae	*Anisognathus somptuosus*				2	13	5	16	5	12	6	21	6	20	6	23	10	28	10	25	9
Fringillidae	*Anisognathus flavinuchus*			x	14	10	3x	9	1x	10	2x	14	1x	17	3x	15	8x	14	9x	14	8x
Fringillidae	*Anisognathus notabilis*			x	2	8	2x	12	7x	7	7x	13	6x	19	2x	12	7x	7	7x	16	6x
Fringillidae	*Stephanophorus diademat*				2	13	5	12	4	19	2	20	3	18	9	21	9	32	5	33	6
Fringillidae	*Iridosornis porphyrocephal*			x	2	20	7x	24	7x	9	7x	31	5x	32	7x	29	7x	15	7x	37	5x
Fringillidae	*Iridosornis analis*				2	9	6	9	3	8	4	15	8	13	6	14	11	24	7	21	9
Fringillidae	*Iridosornis jelskii*				2	7	4	7	0	8	1	11	7	11	4	12	10	21	5	14	11
Fringillidae	*Iridosornis rufivertex*				2	26	10	31	8	20	15	33	14	36	15	44	16x	49	18x	39	23x
Fringillidae	*Iridosornis reinhardti*				2	5	0	4	0	5	0	7	4	9	0	4	7	13	4	9	4
Fringillidae	*Dubusia taeniata*				2	17	3	17	4	11	5	22	6	22	5	26	8	31	10	26	8
Fringillidae	*Delothraupis castaneoventr*				2	7	5	9	0	9	1	10	4	11	5	12	8	17	4	13	8
Fringillidae	*Pipraeidea melanonota*		H		2	0	7	0	6	0	6	0	5	0	12	0	11	0	10	0	9
Fringillidae	*Euphonia jamaica*			x	2	16	0x	16	4x	11	4x	40	4x	40	0x	16	4x	11	4x	40	4x
Fringillidae	*Euphonia plumbea*				2	4	16	3	16	5	16	6	15	8	18	7	21	12	23	11	21
Fringillidae	*Euphonia affinis*				2	11	6	19	4	14	6	29	3	14	8	22	18	19	12	33	7
Fringillidae	*Euphonia luteicapilla*				2	22	5	38	8	31	10	53	7	31	5	44	13	41	12	60	7
Fringillidae	*Euphonia chlorotica*				2	8	14	6	12	8	13	12	10	12	18	11	16	18	17	22	14
Fringillidae	*Euphonia trinitatis*				2	22	4	15	6	19	4	23	3	27	7	23	8	35	11	35	7
Fringillidae	*Euphonia concinna*			x	2	0	6x	0	0x	0	6x	0	0x	0	6x	0	6x	0	6x	0	6x
Fringillidae	*Euphonia saturata*		H		2	0	6	0	8	0	10	0	0	0	6	0	9	0	10	0	0
Fringillidae	*Euphonia finschi*				2	2	19	3	18	5	18	7	18	6	21	6	26	10	26	10	23
Fringillidae	*Euphonia violacea*				2	9	13	7	11	9	11	15	9	14	17	12	15	24	16	25	13
Fringillidae	*Euphonia laniirostris*				2	8	11	5	12	6	12	11	11	11	13	11	15	14	15	19	13
Fringillidae	*Euphonia hirundinacea*			x	2	31	7x	85	0x	33	0x	33	0x	31	7x	85	8x	85	0x	33	0x
Fringillidae	*Euphonia chalybea*				2	21	8	19	8	29	6	30	7	30	18	33	18	51	11	52	15
Fringillidae	*Euphonia elegantissima*				2	14	8	22	5	14	7	30	3	16	10	23	17	19	11	32	4
Fringillidae	*Euphonia musica*				2	11	8	17	6	12	9	23	0	11	9	18	6	13	9	24	0
Fringillidae	*Euphonia cyanocephala*				2	16	7	14	6	17	6	21	6	22	11	24	11	32	11	33	10
Fringillidae	*Euphonia imitans*			x	2	40	9x	30	28x	23	28x	38	28x	54	9x	30	48x	26	40x	59	28x
Fringillidae	*Euphonia fulvicrissa*				2	13	6	17	7	14	5	17	6	20	8	19	7	23	10	27	8
Fringillidae	*Euphonia gouldi*				2	17	4	26	4	20	5	35	2	21	4	34	9	29	6	42	4

Family	Species	IUCN	Ag	R	Range Source	Percentage loss in geographic range 2050 TG		AM		GO		OS		2100 TG		AM		GO		OS	
						H	C	H	C	H	C	H	C	H	C	H	C	H	C	H	C
Fringillidae	*Euphonia chrysopasta*				2	5	9	4	9	4	9	8	9	9	10	8	11	12	11	15	10
Fringillidae	*Euphonia mesochrysa*				2	12	7	10	4	11	6	23	6	18	7	17	12	27	9	30	8
Fringillidae	*Euphonia minuta*				2	6	10	5	10	6	10	9	9	10	11	10	12	14	12	16	11
Fringillidae	*Euphonia anneae*			x	2	17	8x	37	14x	19	14x	40	8x	24	19x	39	14x	37	25x	48	19x
Fringillidae	*Euphonia xanthogaster*				2	5	6	4	6	5	6	8	6	10	7	9	8	12	9	15	8
Fringillidae	*Euphonia rufiventris*				2	5	9	4	9	4	9	8	9	9	10	9	11	12	11	15	10
Fringillidae	*Euphonia cayennensis*				2	3	14	3	13	4	13	8	12	7	15	5	17	17	17	15	15
Fringillidae	*Euphonia pectoralis*				2	18	17	16	15	26	13	25	14	27	22	28	21	42	17	41	20
Fringillidae	*Chlorophonia flavirostris*			x	2	13	1x	9	1x	21	1x	11	0x	17	1x	9	1x	21	1x	22	0x
Fringillidae	*Chlorophonia cyanea*		H		2	0	8	0	8	0	7	0	7	0	13	0	13	0	12	0	12
Fringillidae	*Chlorophonia pyrrhophrys*				2	14	4	14	4	11	5	25	5	21	7	21	15	32	14	34	7
Fringillidae	*Chlorophonia occipitalis*				2	9	1	14	1	14	3	19	1	10	1	15	3	15	3	21	1
Fringillidae	*Chlorophonia callophrys*			x	2	21	11x	31	22x	12	22x	28	22x	29	11x	31	26x	38	26x	47	22x
Fringillidae	*Chlorochrysa phoenicotis*			x	2	5	6x	14	8x	11	8x	22	0x	14	6x	17	8x	12	8x	25	0x
Fringillidae	*Chlorochrysa calliparaea*				14	19	7	12	4	15	6	26	7	24	7	22	10	33	9	33	9
Fringillidae	*Chlorochrysa nitidissima*	T		x	2	0	0x	21	0x	0	0x	21	0x	21	0x	21	0x	0	0x	21	0x
Fringillidae	*Tangara inornata*				2	17	5	20	9	15	8	24	6	24	7	24	11	25	13	33	9
Fringillidae	*Tangara mexicana*		H		2	0	12	0	13	0	13	0	12	0	14	0	15	0	15	0	14
Fringillidae	*Tangara brasiliensis*				2	7	9	9	9	13	5	9	9	8	17	19	29	36	21	19	29
Fringillidae	*Tangara cabanisi*	T		x	2	0	0x	0	0x	0	0x	0	0x	0	0x	0	0x	0	0x	0	0x
Fringillidae	*Tangara palmeri*				2	17	2	10	9	11	3	8	5	20	7	10	9	11	14	19	11
Fringillidae	*Tangara chilensis*				2	5	10	4	10	4	10	7	10	8	11	8	12	11	13	14	12
Fringillidae	*Tangara fastuosa*	T		x	2	1	55x	3	54x	1	53x	1	53x	1	56x	3	67x	4	66x	1	66x
Fringillidae	*Tangara seledon*		H		2	0	11	0	10	0	6	0	8	0	22	0	22	0	13	0	16
Fringillidae	*Tangara cyanocephala*		H		2	0	9	0	10	0	8	0	9	0	22	0	22	0	14	0	18
Fringillidae	*Tangara desmaresti*				2	29	12	21	16	28	9	26	16	34	20	29	23	43	12	32	22
Fringillidae	*Tangara cyanoventris*				2	21	12	18	11	24	7	26	8	26	23	24	21	44	12	36	18
Fringillidae	*Tangara johannae*				2	12	3	13	5	15	0	5	5	25	3	13	5	19	5	21	5
Fringillidae	*Tangara schrankii*				2	5	6	4	6	4	6	7	6	9	6	9	6	11	7	14	7
Fringillidae	*Tangara florida*				2	16	5	18	8	18	8	23	5	18	8	19	9	27	12	35	8
Fringillidae	*Tangara arthus*				2	16	5	14	4	12	5	24	5	23	7	21	11	27	11	30	8
Fringillidae	*Tangara icterocephala*				2	22	5	25	12	15	12	33	8	29	6	27	13	25	15	42	9
Fringillidae	*Tangara xanthocephala*				2	14	5	10	4	10	5	19	6	18	6	18	11	25	9	24	8
Fringillidae	*Tangara chrysotis*				2	11	10	7	5	8	9	17	11	16	10	15	16	29	15	25	14
Fringillidae	*Tangara parzudakii*				2	9	4	9	4	10	5	18	5	17	5	15	11	25	8	24	6
Fringillidae	*Tangara xanthogastra*				2	5	4	4	4	4	4	7	4	9	5	9	6	10	6	14	6
Fringillidae	*Tangara punctata*				2	6	11	4	11	5	11	11	10	11	12	9	13	21	14	20	13
Fringillidae	*Tangara guttata*				2	10	12	10	14	5	15	9	16	14	15	12	19	11	22	14	22
Fringillidae	*Tangara varia*				2	5	13	4	13	3	14	7	13	9	15	7	16	14	18	14	17
Fringillidae	*Tangara rufigula*			x	2	4	3x	7	2x	6	3x	9	0x	9	3x	7	2x	6	3x	12	0x
Fringillidae	*Tangara gyrola*				2	6	10	5	10	6	11	11	10	10	12	10	13	15	13	18	12
Fringillidae	*Tangara lavinia*				2	14	3	19	5	15	2	17	4	21	5	22	5	24	7	27	6
Fringillidae	*Tangara cayana*				2	9	16	7	13	8	14	14	10	12	21	11	17	17	18	22	14
Fringillidae	*Tangara cucullata*			x	2	0	0x	44	0x	0	0x	0	0x	0	0x	44	0x	0	0x	44	0x

Family	Species	IUCN	Ag	R	Range Source	Percentage loss in geographic range 2050 TG H	TG C	AM H	AM C	GO H	GO C	OS H	OS C	2100 TG H	TG C	AM H	AM C	GO H	GO C	OS H	OS C
Fringillidae	*Tangara peruviana*	T			2	29	11	12	25	15	21	14	21	29	36x	21	46x	40	32x	37	41x
Fringillidae	*Tangara preciosa*				2	16	6	14	5	24	4	24	5	23	10	27	9	36	6	39	7
Fringillidae	*Tangara vitriolina*				2	10	3	10	2	7	3	12	1	13	3	14	3	9	4	13	3
Fringillidae	*Tangara meyerdeschauens*	T		x	2	17	1x	0	1x	4	1x	18	1x	35	1x	4	20x	18	20x	18	20x
Fringillidae	*Tangara rufigenis*			x	2	11	0x	3	0x	13	0x	0	0x	16	0x	16	0x	13	3x	0	3x
Fringillidae	*Tangara ruficervix*				2	13	6	14	3	13	5	21	6	18	7	20	10	27	8	25	8
Fringillidae	*Tangara labradorides*				2	8	11	12	9	0	12	7	12	10	11	14	9	8	15	12	14
Fringillidae	*Tangara cyanotis*				2	10	4	8	4	9	4	18	6	15	4	17	11	27	7	25	8
Fringillidae	*Tangara cyanicollis*				2	10	21	7	23	6	26	13	22	15	26	12	27	20	28	20	24
Fringillidae	*Tangara larvata*				2	16	4	21	7	15	7	27	4	20	6	26	11	24	9	33	6
Fringillidae	*Tangara nigrocincta*				2	6	8	5	8	5	9	10	8	11	10	10	10	15	10	18	10
Fringillidae	*Tangara dowii*			x	2	14	13x	31	20x	18	20x	27	20x	18	13x	31	23x	54	23x	50	20x
Fringillidae	*Tangara fucosa*			x	2	15	0x	51	15x	0	15x	15	0x	15	51x	51	15x	0	66x	47	51x
Fringillidae	*Tangara nigroviridis*				2	16	5	14	3	13	4	21	5	22	5	22	9	27	8	26	7
Fringillidae	*Tangara vassorii*				2	15	4	15	3	13	5	22	7	21	7	22	10	27	10	25	9
Fringillidae	*Tangara heinei*				2	13	1	13	2	11	2	19	2	20	4	22	7	24	8	23	3
Fringillidae	*Tangara phillipsi*			x	14	93	0x	69	0x	70	0x	100	0x	93	0x	69	0x	82	0x	100	0x
Fringillidae	*Tangara viridicollis*				2	7	13	7	3	5	11	3	13	10	13	8	12	11	15	6	16
Fringillidae	*Tangara argyrofenges*				2	6	3	8	1	6	2	8	1	6	3	9	1	7	2	8	1
Fringillidae	*Tangara cyanoptera*				2	10	17	10	19	7	21	6	22	15	23	14	26	12	32	10	34
Fringillidae	*Tangara velia*				2	4	9	3	8	3	8	7	8	8	10	7	11	12	11	14	10
Fringillidae	*Tangara callophrys*				2	6	2	4	2	5	2	9	2	11	3	12	4	13	3	20	3
Fringillidae	*Iridophanes pulcherrima*				2	13	7	10	3	11	7	21	9	22	7	18	13	28	9	27	11
Fringillidae	*Pseudodacnis hartlaubi*	T		x	2	0	0x	0	0x	0	0x	0	0x	0	0x	0	0x	0	0x	0	0x
Fringillidae	*Dacnis albiventris*				2	4	2	3	2	2	2	6	2	8	2	10	2	10	3	18	2
Fringillidae	*Dacnis lineata*				2	4	10	3	11	3	11	7	10	7	12	7	13	10	14	12	12
Fringillidae	*Dacnis egregia*				2	12	4	6	4	8	6	14	0	14	4	11	6	14	6	16	2
Fringillidae	*Dacnis flaviventer*				2	5	8	4	8	4	9	8	8	9	10	9	10	13	10	16	9
Fringillidae	*Dacnis nigripes*				2	25	18	11	26	22	17	17	24	26	39	23	42	51	23	30	42
Fringillidae	*Dacnis venusta*				2	18	6	21	10	16	9	23	7	25	8	22	12	24	13	34	9
Fringillidae	*Dacnis cayana*				2	8	12	6	12	7	12	12	10	12	15	10	15	18	15	21	13
Fringillidae	*Dacnis viguieri*				2	15	7	14	12	9	12	19	7	24	15	16	12	19	20	37	15
Fringillidae	*Dacnis berlepschi*	T		x	2	27	4x	15	4x	31	4x	20	0x	27	4x	15	4x	31	4x	36	0x
Fringillidae	*Chlorophanes spiza*				2	7	11	6	12	6	12	11	11	11	13	10	15	17	15	18	13
Fringillidae	*Cyanerpes nitidus*				2	4	7	2	6	2	7	5	6	7	7	5	7	8	8	11	8
Fringillidae	*Cyanerpes lucidus*				2	21	4	35	6	25	7	44	4	27	5	43	9	38	10	51	6
Fringillidae	*Cyanerpes caeruleus*				2	6	10	5	10	6	11	9	10	10	12	9	13	16	14	18	12
Fringillidae	*Cyanerpes cyaneus*				2	6	12	5	12	5	13	9	11	9	14	9	16	15	17	17	14
Fringillidae	*Xenodacnis parina*				2	7	4	7	4	7	5	9	7	11	5	9	7	14	5	11	7
Fringillidae	*Tersina viridis*				2	8	14	6	12	7	13	12	11	13	17	11	16	18	17	20	15
Fringillidae	*Catamblyrhynchus diadem*				2	17	5	14	3	13	6	19	6	22	7	24	10	29	10	24	9
Fringillidae	*Oreothraupis arremonops*	T		x	2	0	33x	14	33x	14	33x	47	0x	0	33x	14	33x	14	33x	47	0x
Fringillidae	*Urothraupis stolzmanni*			x	2	14	6x	18	6x	17	6x	34	6x	22	15x	35	23x	55	8x	35	18x
Fringillidae	*Nephelornis oneilli*			x	2	0	0x	0	0x	0	0x	0	0x	0	0x	0	0x	0	0x	0	0x

Family	Species	IUCN	Ag	R	Range Source	Percentage loss in geographic range 2050 TG H	TG C	AM H	AM C	GO H	GO C	OS H	OS C	2100 TG H	TG C	AM H	AM C	GO H	GO C	OS H	OS C
Fringillidae	*Charitospiza eucosma*				2	6	17	1	9	2	15	9	6	8	24	5	12	7	18	15	8
Fringillidae	*Coryphaspiza melanotis*	T			2	6	15	5	4	7	8	9	3	10	21	9	6	13	10	13	5
Fringillidae	*Saltatricula multicolor*				2	2	10	1	7	2	7	3	7	3	12	2	17	5	20	6	17
Fringillidae	*Coryphospingus pileatus*				2	9	20	7	18	8	19	14	14	11	24	10	21	15	23	22	18
Fringillidae	*Coryphospingus cucullatus*		H		2	0	9	0	7	0	7	0	6	0	13	0	11	0	12	0	10
Fringillidae	*Rhodospingus cruentus*				2	7	6	4	9	7	9	12	0	7	6	5	11	10	11	14	2
Fringillidae	*Phrygilus atriceps*		H		2	0	18	0	21	0	20	0	14	0	24	0	29	0	29	0	18
Fringillidae	*Phrygilus punensis*		H		2	0	14	0	13	0	13	0	12	0	17	0	18	0	17	0	15
Fringillidae	*Phrygilus gayi*				2	2	7	3	7	2	8	3	7	5	11	5	14	6	17	8	16
Fringillidae	*Phrygilus patagonicus*				2	5	13	4	12	4	13	3	13	6	22	6	25	8	25	7	24
Fringillidae	*Phrygilus fruticeti*				2	5	10	5	11	3	11	6	9	6	14	7	18	8	20	12	16
Fringillidae	*Phrygilus unicolor*				2	7	12	8	13	5	13	10	11	9	17	11	20	14	21	17	16
Fringillidae	*Phrygilus dorsalis*				2	1	12	0	13	0	13	3	10	1	12	2	17	2	20	6	14
Fringillidae	*Phrygilus erythronotus*				2	17	21	16	30	9	28	23	20	19	35	27	42	32	40	44	24
Fringillidae	*Phrygilus plebejus*				2	9	13	8	15	6	14	11	12	11	18	13	21	16	22	19	16
Fringillidae	*Phrygilus carbonarius*		H		2	0	11	0	13	0	14	0	12	0	16	0	22	0	27	0	23
Fringillidae	*Phrygilus alaudinus*				2	7	14	10	14	7	14	11	10	10	18	14	19	18	20	19	15
Fringillidae	*Melanodera melanodera*		H		2	0	1	0	1	0	2	0	1	0	2	0	4	0	4	0	4
Fringillidae	*Melanodera xanthogramma*		H		2	0	17	0	17	0	18	0	17	0	28	0	30	0	30	0	30
Fringillidae	*Haplospiza rustica*				2	11	3	16	2	13	4	22	3	16	4	22	6	25	7	26	5
Fringillidae	*Haplospiza unicolor*				2	16	7	15	6	23	4	24	5	23	15	25	14	38	8	39	10
Fringillidae	*Acanthidops bairdii*			x	2	5	26x	32	30x	14	30x	15	30x	7	26x	32	31x	56	31x	46	30x
Fringillidae	*Lophospingus pusillus*				2	4	6	1	5	4	4	4	4	4	8	2	11	6	13	5	11
Fringillidae	*Lophospingus griseocristat*				2	10	4	7	6	7	6	16	0	11	7	8	10	9	12	19	0
Fringillidae	*Donacospiza albifrons*				2	10	4	8	3	13	2	14	3	15	7	14	8	22	5	23	5
Fringillidae	*Diuca speculifera*				2	4	25	8	25	6	24	11	19	6	31	14	31	16	32	23	22
Fringillidae	*Diuca diuca*				2	2	9	2	9	2	10	2	9	3	12	3	16	4	19	5	16
Fringillidae	*Idiopsar brachyurus*				2	7	25	4	25	2	25	19	14	7	31	9	28	8	36	19	14
Fringillidae	*Piezorhina cinerea*				2	0	10	0	13x	0	13x	0	13x	0	13x	0	20x	0	20x	0	20x
Fringillidae	*Xenospingus concolor*		H		2	0	34	0	38	0	45	0	34	0	46	0	69	0	71	0	71
Fringillidae	*Incaspiza pulchra*			x	2	0	27x	24	19x	20	31x	36	6x	0	51x	24	43x	27	46x	46	20x
Fringillidae	*Incaspiza personata*			x	2	0	0x	15	0x	12	0x	16	0x	0	0x	15	1x	16	0x	16	0x
Fringillidae	*Incaspiza ortizi*	T		x	2	0	0x	0	0x	0	0x	0	0x	0	0x	0	0x	0	0x	0	0x
Fringillidae	*Incaspiza laeta*			x	2	0	0x	0	0x	0	0x	0	0x	0	0x	0	0x	0	0x	0	0x
Fringillidae	*Incaspiza watkinsi*			x	2	0	0x	0	0x	0	0x	0	0x	0	0x	0	0x	0	0x	0	0x
Fringillidae	*Poospiza thoracica*				2	27	10	13	17	24	10	27	14	29	19	28	26	45	16	35	20
Fringillidae	*Poospiza boliviana*				2	10	6	8	5	11	5	15	0	10	7	10	7	12	6	17	0
Fringillidae	*Poospiza alticola*	T			2	0	0	17	0	8	0	8	0	0	3	17	1	14	1	14	1
Fringillidae	*Poospiza hypochondria*				2	7	11	8	12	6	12	13	7	11	14	9	19	10	19	17	10
Fringillidae	*Poospiza ornata*		H		2	0	12	0	12	0	13	0	11	0	16	0	20	0	22	0	18
Fringillidae	*Poospiza erythrophrys*				2	4	7	6	4	4	5	6	4	4	7	8	5	7	8	8	5
Fringillidae	*Poospiza whitii*				2	5	7	4	6	5	6	4	5	5	7	4	8	6	9	6	6
Fringillidae	*Poospiza nigrorufa*				2	8	2	6	1	11	1	11	1	11	3	12	2	15	1	16	1
Fringillidae	*Poospiza lateralis*				2	21	4	14	5	27	3	25	4	28	8	28	10	37	6	37	7

Family	Species	IUCN	Ag	R	Range Source	Percentage loss in geographic range 2050								2100							
						TG		AM		GO		OS		TG		AM		GO		OS	
						H	C	H	C	H	C	H	C	H	C	H	C	H	C	H	C
Fringillidae	*Poospiza rubecula*	T			2	0	20	19	14	11	20	29	1	0	33	19	28x	17	33x	37	7
Fringillidae	*Poospiza garleppi*	T	H	x	2	0	36x	0	34x	0	33x	0	1x	0	36x	0	34x	0	38x	0	1x
Fringillidae	*Poospiza baeri*	T	H	x	2	0	1x	0	1x	0	1x	0	0x	0	1x	0	8x	0	12x	0	4x
Fringillidae	*Poospiza caesar*				2	1	3	1	0	0	2	0	2	1	3	1	4	6	2	4	2
Fringillidae	*Poospiza hispaniolensis*		H		2	0	21	0	19	0	25	0	16	0	32	0	41	0	43	0	34
Fringillidae	*Poospiza torquata*				2	3	11	2	11	3	11	3	10	4	13	3	18	5	19	5	16
Fringillidae	*Poospiza melanoleuca*				2	3	5	2	3	3	3	4	3	4	6	4	7	6	8	7	7
Fringillidae	*Poospiza cinerea*	T			2	4	15	1	5	4	13	8	3	7	23	4	9	8	15	12	5
Fringillidae	*Sicalis citrina*				2	7	15	4	8	5	13	10	6	8	23	10	12	10	17	16	9
Fringillidae	*Sicalis lutea*				2	11	20	10	21	6	22	13	18	12	27	16	27	18	30	25	20
Fringillidae	*Sicalis uropygialis*				2	9	17	9	18	7	17	12	14	11	22	13	26	16	26	21	18
Fringillidae	*Sicalis luteocephala*				2	14	15	12	16	12	17	30	2	18	19	15	21	15	25	37	2
Fringillidae	*Sicalis auriventris*				2	0	9	1	8	0	9	3	9	0	11	1	14	3	14	8	16
Fringillidae	*Sicalis olivascens*		H		2	0	13	0	14	0	13	0	11	0	16	0	19	0	19	0	15
Fringillidae	*Sicalis lebruni*				2	0	1	0	1	0	2	0	1	0	2	0	5	0	7	1	6
Fringillidae	*Sicalis columbiana*		H		2	0	11	0	6	0	7	0	3	0	13	0	7	0	10	0	4
Fringillidae	*Sicalis flaveola*		H		2,11	0	11	0	9	0	9	0	7	0	14	0	13	0	14	0	11
Fringillidae	*Sicalis luteola*		H		2	0	4	0	4	0	4	0	4	0	5	0	7	0	8	0	7
Fringillidae	*Sicalis raimondii*				2	0	36	9	35	8	49	8	30	0	64	9	68	23	62	17	64
Fringillidae	*Sicalis taczanowskii*				2	0	12x	0	15x	0	15x	1	15x	0	15x	0	23x	1	24x	1	23x
Fringillidae	*Emberizoides herbicola*		H		2	0	13	0	8	0	10	0	7	0	19	0	12	0	13	0	10
Fringillidae	*Emberizoides duidae*			x	14	0	0x	0	0x	0	0x	0	0x	0	0x	0	0x	0	0x	0	0x
Fringillidae	*Emberizoides ypiranganus*				2	17	6	13	7	22	5	24	6	26	8	25	10	36	6	36	9
Fringillidae	*Embernagra platensis*				2	7	5	5	5	9	4	10	4	10	7	9	8	14	7	14	6
Fringillidae	*Embernagra longicauda*		H		2	0	14	0	5	0	6	0	0	0	18	0	7	0	9	0	2
Fringillidae	*Volatinia jacarina*		H		2	0	11	0	10	0	11	0	9	0	14	0	14	0	15	0	12
Fringillidae	*Sporophila frontalis*	T			2	18	8	19	8	25	6	26	8	25	19	32	19	46	13	51	15
Fringillidae	*Sporophila falcirostris*	T			2	13	11	13	12	24	7	21	10	21	25	20	28	48	17	43	23
Fringillidae	*Sporophila schistacea*		H		2	0	14	0	15	0	14	0	14	0	15	0	17	0	17	0	17
Fringillidae	*Sporophila intermedia*		H		2	0	7	0	9	0	7	0	6	0	10	0	13	0	13	0	11
Fringillidae	*Sporophila plumbea*		H		2	0	18	0	14	0	16	0	13	0	23	0	18	0	19	0	16
Fringillidae	*Sporophila corvina*		H		2	0	5	0	7	0	7	0	4	0	5	0	10	0	9	0	5
Fringillidae	*Sporophila americana*		H		2	0	14	0	12	0	13	0	10	0	14	0	14	0	17	0	12
Fringillidae	*Sporophila murallae*			x	2	10	2x	7	1x	7	1x	25	1x	18	2x	17	2x	35	1x	46	2x
Fringillidae	*Sporophila torqueola*		H		2	0	3	0	2	0	4	0	2	0	4	0	13	0	7	0	3
Fringillidae	*Sporophila collaris*				2	5	10	3	6	4	7	7	6	7	13	6	9	10	11	11	8
Fringillidae	*Sporophila bouvronides*		H		2	0	9	0	10	0	8	0	7	0	9	0	12	0	13	0	7
Fringillidae	*Sporophila lineola*		H		2	0	15	0	12	0	13	0	9	0	19	0	17	0	19	0	14
Fringillidae	*Sporophila luctuosa*		H		2	0	5	0	3	0	5	0	4	0	6	0	8	0	9	0	7
Fringillidae	*Sporophila nigricollis*		H		2	0	13	0	11	0	12	0	8	0	17	0	14	0	16	0	12
Fringillidae	*Sporophila melanops*	T		x	14	0	⊦02x	0	0x	0	0x	0	0x	0	⊦02x	0	0x	0	0x	0	0x
Fringillidae	*Sporophila caerulescens*				2	6	11	4	8	6	9	8	8	8	15	7	13	12	14	12	12
Fringillidae	*Sporophila albogularis*				2	5	16	4	13	8	14	12	9	7	20	6	14	16	15	23	10
Fringillidae	*Sporophila leucoptera*				2	10	17	7	15	8	17	14	13	14	22	12	21	19	24	24	19

Family	Species	IUCN	Ag	R	Range Source	Percentage loss in geographic range															
						2050								2100							
						TG		AM		GO		OS		TG		AM		GO		OS	
						H	C	H	C	H	C	H	C	H	C	H	C	H	C	H	C
Fringillidae	*Sporophila peruviana*		H		2	0	14	0	13	0	17	0	12	0	19	0	30	0	32	0	27
Fringillidae	*Sporophila simplex*		H		2	0	17	0	16	0	19	0	11	0	22	0	20	0	23	0	15
Fringillidae	*Sporophila nigrorufa*	T			2	13	38	6	50	10	48	10	48	16	49	10	60	18	59	21	58
Fringillidae	*Sporophila bouvreuil*				2	8	16	7	9	8	12	13	7	11	22	11	12	15	15	20	9
Fringillidae	*Sporophila minuta*				2	11	6	8	7	10	6	15	6	14	8	14	11	22	10	23	8
Fringillidae	*Sporophila hypoxantha*				2	7	10	6	5	7	7	10	4	11	16	9	8	14	10	15	7
Fringillidae	*Sporophila ruficollis*		H		2	0	4	0	3	0	3	0	3	0	5	0	5	0	6	0	6
Fringillidae	*Sporophila palustris*	T			2	7	1	6	1	10	0	11	1	10	1	10	1	15	0	14	1
Fringillidae	*Sporophila castaneiventris*		H		2	0	7	0	6	0	6	0	4	0	8	0	9	0	8	0	6
Fringillidae	*Sporophila hypochroma*				2	0	2	0	1	0	1	0	1	0	2	0	1	0	1	0	1
Fringillidae	*Sporophila cinnamomea*	T			2	0	0	1	0	1	0	1	0	1	0	2	0	1	0	1	0
Fringillidae	*Sporophila zelichi*	T		x	2	0	0x	0	0x	0	0x	0	0x	0	0x	0	0x	0	0x	0	0x
Fringillidae	*Sporophila melanogaster*				2	26	0	20	0	38	0	22	0	37	0	48	0	50	0	65	0
Fringillidae	*Sporophila telasco*		H		2	0	19	0	19	0	24	0	15	0	27	0	37	0	36	0	31
Fringillidae	*Sporophila insulata*	T		x	14	0	0x	33	0x	33	0x	33	0x	33	0x	33	0x	33	0x	33	0x
Fringillidae	*Oryzoborus nuttingi*		H	x	2	0	0x	0	1x	0	2x	0	0x	0	0x	0	4x	0	2x	0	0x
Fringillidae	*Oryzoborus crassirostris*		H		2	0	7	0	7	0	6	0	6	0	8	0	10	0	9	0	8
Fringillidae	*Oryzoborus atrirostris*				2	11	7	7	7	13	6	25	3	17	7	19	9	28	7	44	4
Fringillidae	*Oryzoborus maximiliani*		H		2	0	15	0	6	0	9	0	4	0	20	0	9	0	13	0	7
Fringillidae	*Oryzoborus angolensis*				2	8	12	6	11	7	12	12	9	12	15	11	14	18	15	21	12
Fringillidae	*Amaurospiza concolor*				2	11	2	18	2	19	4	29	2	15	2	24	5	26	6	31	2
Fringillidae	*Amaurospiza moesta*				2	19	9	19	9	27	5	27	8	26	21	30	20	47	12	48	15
Fringillidae	*Melopyrrha nigra*				2	8	13	27	13	14	9	38	6	17	13	29	15	18	9	41	6
Fringillidae	*Dolospingus fringilloides*			x	2	4	2x	4	1x	0	2x	1	1x	4	3x	4	4x	4	5x	2	4x
Fringillidae	*Catamenia analis*		H		2	0	14	0	16	0	15	0	12	0	18	0	22	0	23	0	18
Fringillidae	*Catamenia inornata*		H		2	0	14	0	15	0	13	0	11	0	17	0	19	0	19	0	14
Fringillidae	*Catamenia homochroa*				2	11	6	12	4	8	4	15	4	14	7	22	9	29	10	19	8
Fringillidae	*Tiaris obscura*		H		2	0	9	0	8	0	9	0	7	0	12	0	16	0	18	0	14
Fringillidae	*Tiaris canora*				2	9	13	25	13	12	9	38	6	18	13	28	16	16	10	40	6
Fringillidae	*Tiaris olivacea*		H		2,11	0	5	0	5	0	6	0	3	0	6	0	11	0	8	0	4
Fringillidae	*Tiaris bicolor*		H		2	0	7	0	8	0	9	0	4	0	13	0	13	0	20	0	13
Fringillidae	*Tiaris fuliginosa*		H		2	0	15	0	14	0	14	0	13	0	25	0	28	0	26	0	25
Fringillidae	*Loxipasser anoxanthus*			x	2	16	0x	16	4x	11	4x	40	4x	40	0x	16	4x	11	4x	40	4x
Fringillidae	*Loxigilla portoricensis*			x	2	18	0x	18	0x	21	0x	21	0x	18	0x	18	0x	21	0x	21	0x
Fringillidae	*Loxigilla violacea*				2	13	7	23	6	19	9	29	1	18	9	23	6	20	9	34	1
Fringillidae	*Loxigilla noctis*			x	2	8	11x	38	6x	22	5x	39	0x	9	11x	48	6x	34	5x	58	0x
Fringillidae	*Diglossa baritula*		H		2	0	3	0	0	0	4	0	0	0	3	0	4	0	4	0	0
Fringillidae	*Diglossa plumbea*		H	x	2	0	14x	0	20x	0	20x	0	20x	0	14x	0	22x	0	21x	0	20x
Fringillidae	*Diglossa sittoides*				2	12	6	12	4	10	5	15	4	15	8	18	8	21	8	18	6
Fringillidae	*Diglossa venezuelensis*	T		x	2	0	0x	0	0x	0	0x	0	0x	0	0x	34	0x	34	0x	34	0x
Fringillidae	*Diglossa albilatera*				2	19	3	21	4	13	5	27	5	24	7	31	9	35	13	32	9
Fringillidae	*Diglossa gloriosissima*	T		x	2	100	0x	48	0x	100	0x	100	0x	100	0x	48	0x	100	0x	100	0x
Fringillidae	*Diglossa lafresnayii*				2	21	6	24	7	16	12	31	11	28	15	36	14	39	20	39	15
Fringillidae	*Diglossa mystacalis*				2	5	3	6	0	6	0	7	2	8	3	10	3	12	3	8	5

Family	Species	IUCN	Ag	R	Range Source	Percentage loss in geographic range 2050 TG H	TG C	AM H	AM C	GO H	GO C	OS H	OS C	2100 TG H	TG C	AM H	AM C	GO H	GO C	OS H	OS C
Fringillidae	*Diglossa gloriosa*			x	2	33	0x	8	3x	16	3x	54	3x	33	8x	8	35x	33	36x	56	3x
Fringillidae	*Diglossa humeralis*				2	17	7	29	5	18	9	28	9	28	11	40	10	47	14	35	16
Fringillidae	*Diglossa brunneiventris*				2	8	9	10	10	9	9	9	11	12	12	11	15	18	13	15	13
Fringillidae	*Diglossa carbonaria*				2	15	16	17	13	16	10	17	10	21	16	19	13	20	12	22	10
Fringillidae	*Diglossa duidae*				2	0	13	0	13	0	13	0	13	0	15	0	21	0	21	0	21
Fringillidae	*Diglossa major*				2	16	2	0	18	18	9	0	18	16	21	12	45	28	32	6	58
Fringillidae	*Diglossopis indigotica*			x	2	1	0x	12	1x	3	0x	13	1x	15	0x	13	1x	3	1x	13	1x
Fringillidae	*Diglossopis glauca*				2	14	6	14	3	13	5	18	9	19	6	19	11	29	9	25	11
Fringillidae	*Diglossopis caerulescens*				2	14	5	16	2	10	3	16	5	18	6	21	8	24	9	19	9
Fringillidae	*Diglossopis cyanea*				2	15	5	14	3	10	5	20	6	20	7	23	9	29	10	24	9
Fringillidae	*Euneornis campestris*			x	2	29	0x	29	3x	25	3x	55	3x	55	0x	29	3x	25	3x	55	3x
Fringillidae	*Melanospiza richardsoni*	T		x	2	0	46x	37	46x	46	0x	46	0x	0	46x	37	46x	46	0x	83	0x
Fringillidae	*Geospiza magnirostris*			x	2	0	11x	0	11x	0	7x	0	11x	0	11x	0	30x	0	41x	0	30x
Fringillidae	*Geospiza fortis*			x	2	0	12x	0	12x	0	7x	0	12x	0	12x	0	30x	0	40x	0	30x
Fringillidae	*Geospiza fuliginosa*			x	2	0	13x	0	13x	0	8x	0	13x	0	13x	0	31x	0	41x	0	31x
Fringillidae	*Geospiza difficilis*			x	2	0	13x	0	13x	0	0x	0	13x	0	13x	0	38x	0	73x	0	38x
Fringillidae	*Geospiza scandens*			x	2	0	8x	0	8x	0	8x	0	8x	0	8x	0	28x	0	44x	0	28x
Fringillidae	*Geospiza conirostris*			x	2	0⊦02x		0⊦02x		0⊦02x		0⊦02x		0⊦02x		0⊦02x		0⊦02x		0⊦02x	
Fringillidae	*Camarhynchus crassirostri*			x	2	0	5x	0	5x	0	0x	0	5x	0	5x	0	25x	0	36x	0	25x
Fringillidae	*Camarhynchus psittacula*			x	2	0	5x	0	5x	0	0x	0	5x	0	5x	0	25x	0	36x	0	25x
Fringillidae	*Camarhynchus pauper*	T		x	2	0	0x	0	0x	0	0x	0	0x	0	0x	0	0x	0⊦02x		0	0x
Fringillidae	*Camarhynchus parvulus*			x	2	0	12x	0	12x	0	7x	0	12x	0	12x	0	31x	0	41x	0	31x
Fringillidae	*Camarhynchus pallidus*			x	2	0	9x	0	9x	0	8x	0	9x	0	9x	0	29x	0	43x	0	29x
Fringillidae	*Camarhynchus heliobates*	T		x	2	0	7x	0	7x	0	0x	0	7x	0	7x	0	22x	0	16x	0	22x
Fringillidae	*Certhidea olivacea*			x	2	0	12x	0	12x	0	8x	0	12x	0	12x	0	31x	0	42x	0	31x
Fringillidae	*Spiza americana*		H		2	0	11	0	7	0	9	0	4	0	13	0	8	0	11	0	5
Fringillidae	*Pheucticus chrysopeplus*				2	14	12	26	8	13	10	35	7	19	14	26	22	19	19	41	11
Fringillidae	*Pheucticus chrysogaster*				2	8	9	7	8	11	9	13	7	10	12	14	13	18	11	18	9
Fringillidae	*Pheucticus tibialis*			x	2	20	9x	34	14x	20	14x	36	14x	26	9x	34	19x	47	17x	52	14x
Fringillidae	*Pheucticus aureoventris*				2	13	6	12	2	11	4	17	5	16	6	17	6	20	8	20	7
Fringillidae	*Pheucticus ludovicianus*				2	13	22	19	14	19	18	20	11	17	24	26	17	31	20	30	14
Fringillidae	*Pheucticus melanocephalu*				2	3	18	7	15	5	17	9	13	4	22	9	24	8	28	13	21
Fringillidae	*Cardinalis cardinalis*		H		2,11,13	0	16	0	9	0	13	0	7	0	20	0	17	0	19	0	11
Fringillidae	*Cardinalis phoeniceus*				2	31	7	18	20	21	12	27	6	32	22	22	27	24	31	33	25
Fringillidae	*Cardinalis sinuatus*		H		2	0	20	0	17	0	18	0	15	0	24	0	35	0	35	0	28
Fringillidae	*Caryothraustes poliogaster*				2	17	4	27	4	20	5	36	3	21	5	34	9	30	7	43	5
Fringillidae	*Caryothraustes canadensis*				2	3	12	3	12	3	12	6	11	6	13	4	15	12	16	11	15
Fringillidae	*Caryothraustes humeralis*				2	5	6	4	5	4	5	9	5	10	6	10	7	15	7	21	6
Fringillidae	*Rhodothraupis celaeno*				2	1	11	5	3	3	3	4	3	1	13	6	48	3	3	4	3
Fringillidae	*Periporphyrus erythromela*				2	4	15	3	15	5	14	7	14	7	16	7	20	15	20	13	18
Fringillidae	*Saltator grossus*				2	5	9	4	9	5	9	9	8	9	10	8	11	15	11	17	10
Fringillidae	*Saltator fuliginosus*				2	19	7	16	7	26	5	27	7	26	15	30	15	45	9	46	12
Fringillidae	*Saltator atriceps*				2	10	4	22	4	16	5	29	3	13	5	27	10	23	8	33	5
Fringillidae	*Saltator maximus*				2	7	12	5	11	6	12	11	10	11	14	9	14	16	15	19	12

Family	Species	IUCN	Ag	R	Range Source	Percentage loss in geographic range 2050 TG H	C	AM H	C	GO H	C	OS H	C	2100 TG H	C	AM H	C	GO H	C	OS H	C
Fringillidae	*Saltator atripennis*		H		2	0	6	0	7	0	9	0	2	0	6	0	8	0	9	0	3
Fringillidae	*Saltator coerulescens*				2	7	12	5	11	6	12	11	10	11	13	10	15	16	16	20	13
Fringillidae	*Saltator similis*		H		2	0	15	0	9	0	12	0	8	0	23	0	14	0	15	0	11
Fringillidae	*Saltator orenocensis*		H		2	0	5	0	6	0	4	0	4	0	9	0	9	0	11	0	9
Fringillidae	*Saltator nigriceps*			x	2	11	8x	5	11x	0	18x	0	16x	11	8x	5	13x	2	20x	2	19x
Fringillidae	*Saltator aurantiirostris*				2	4	10	3	10	4	10	5	8	5	13	4	17	7	17	8	14
Fringillidae	*Saltator maxillosus*				2	22	5	19	6	30	3	33	5	31	14	35	13	54	7	61	10
Fringillidae	*Saltator cinctus*			x	2	18	34x	28	21x	0	48x	1	48x	18	34x	28	21x	10	48x	10	48x
Fringillidae	*Saltator atricollis*				2	7	15	4	7	5	11	10	5	9	21	8	9	9	14	17	7
Fringillidae	*Saltator rufiventris*				2	12	9	14	10	14	8	27	2	20	12	17	16	21	14	33	2
Fringillidae	*Saltator albicollis*		H	x	2	0	18x	0	11x	0	7x	0	0x	0	18x	0	11x	0	7x	0	0x
Fringillidae	*Saltator striatipectus*		H		2	0	7	0	8	0	9	0	6	0	11	0	14	0	18	0	12
Fringillidae	*Cyanoloxia glaucocaerulea*				2	13	1	10	1	16	1	18	1	18	1	19	2	26	1	28	1
Fringillidae	*Cyanocompsa cyanoides*				2	6	10	5	10	5	10	10	9	11	11	10	12	16	13	18	11
Fringillidae	*Cyanocompsa parellina*				2	6	7	12	3	9	6	20	3	6	9	13	28	13	8	20	5
Fringillidae	*Cyanocompsa brissonii*				2	9	14	7	12	10	12	13	9	13	18	12	17	19	18	21	14
Fringillidae	*Guiraca caerulea*		H		2	0	16	0	11	0	14	0	10	0	20	0	21	0	23	0	16
Fringillidae	*Passerina amoena*				2	1	26	3	23	2	26	3	20	1	29	3	26	6	29	6	23
Fringillidae	*Passerina cyanea*		H		2	0	19	0	11	0	15	0	9	0	23	0	16	0	21	0	12
Fringillidae	*Passerina versicolor*				2	3	23	7	18	4	19	9	16	4	25	7	33	6	28	10	22
Fringillidae	*Passerina ciris*				2	5	16	7	11	7	13	9	9	12	20	11	18	17	20	12	13
Fringillidae	*Passerina rositae*			x	2	1	5x	14	0x	14	0x	14	0x	1	5x	14	29x	14	0x	14	0x
Fringillidae	*Passerina leclancherii*		H		2	0	3	0	2	0	2	0	0	0	3	0	20	0	2	0	0
Fringillidae	*Porphyrospiza caerulescen*				2	5	16	3	5	4	11	9	3	7	23	8	7	7	14	15	5
Fringillidae	*Psarocolius oseryi*				2	7	2	5	1	6	1	12	1	12	2	16	1	13	1	30	1
Fringillidae	*Psarocolius decumanus*				2	6	12	4	12	5	12	9	11	10	14	9	15	15	15	18	14
Fringillidae	*Psarocolius viridis*				2	4	8	3	8	3	8	7	8	8	9	6	10	13	10	15	10
Fringillidae	*Psarocolius atrovirens*				2	20	3	8	1	12	3	23	6	25	3	16	10	27	6	27	9
Fringillidae	*Psarocolius angustifrons*				2	7	3	5	3	5	3	9	2	11	3	11	4	13	4	18	3
Fringillidae	*Psarocolius wagleri*				2	16	4	23	7	16	6	29	4	21	5	28	9	25	9	35	5
Fringillidae	*Ocyalus latirostris*				2	1	1	0	0	1	0	2	0	4	1	4	0	3	0	11	0
Fringillidae	*Gymnostinops montezuma*				2	13	4	21	4	16	4	27	3	16	4	26	8	22	6	32	4
Fringillidae	*Gymnostinops cassini*	T		x	2	0	0x	4	0x	0	0x	0	0x	17	4x	4	0x	17	4x	17	4x
Fringillidae	*Gymnostinops bifasciatus*				2	8	15	4	15	14	12	20	12	18	15	6	19	51	18	38	17
Fringillidae	*Psarocolius yuracares*				14	5	9	3	9	3	10	7	9	9	11	7	11	13	11	15	10
Fringillidae	*Gymnostinops guatimozinu*				2	20	6	24	12	12	14	28	5	21	13	29	14	21	21	30	13
Fringillidae	*Cacicus cela*				2	6	12	4	12	5	12	10	11	10	14	9	15	16	15	18	14
Fringillidae	*Cacicus haemorrhous*				2	5	11	4	11	5	11	8	10	9	12	8	13	15	13	16	12
Fringillidae	*Cacicus uropygialis*				2	8	7	13	6	7	7	18	6	13	10	20	12	23	16	24	9
Fringillidae	*Cacicus microrhynchus*				14	19	3	26	6	23	5	31	4	28	4	32	9	32	8	43	6
Fringillidae	*Cacicus koepckeae*	T			2	6	0	5	0	5	0	12	0	6	0	21	0	16	0	24	0
Fringillidae	*Cacicus chrysopterus*				2	12	9	12	8	14	8	17	8	17	11	20	13	24	15	27	13
Fringillidae	*Cacicus chrysonotus*				2	10	5	5	1	8	3	15	5	16	5	15	12	24	8	22	10
Fringillidae	*Cacicus leucoramphus*				14	15	5	19	4	12	6	23	6	23	6	28	13	37	12	28	11

Family	Species	IUCN	Ag	R	Range Source	Percentage loss in geographic range 2050 TG H	TG C	AM H	AM C	GO H	GO C	OS H	OS C	2100 TG H	TG C	AM H	AM C	GO H	GO C	OS H	OS C
Fringillidae	*Cacicus sclateri*				2	9	2	4	1	9	0	15	1	14	2	17	1	20	0	31	1
Fringillidae	*Cacicus solitarius*				2	6	14	4	11	5	13	10	10	10	16	9	14	15	16	19	13
Fringillidae	*Cacicus melanicterus*				2	12	8	21	6	15	7	36	3	15	9	21	26	19	9	38	5
Fringillidae	*Amblycercus holosericeus*				2	13	5	17	5	13	6	23	4	17	6	21	11	22	10	29	6
Fringillidae	*Icterus chrysocephalus*				2	5	7	3	7	4	7	8	6	9	8	8	9	11	10	15	9
Fringillidae	*Icterus cayanensis*				2	7	14	5	12	7	13	11	10	11	17	9	15	16	16	19	13
Fringillidae	*Icterus graduacauda*		H		2	0	4	0	2	0	3	0	1	0	5	0	24	0	3	0	1
Fringillidae	*Icterus chrysater*				2	12	3	19	3	14	5	25	2	16	5	24	8	21	11	30	5
Fringillidae	*Icterus nigrogularis*				2	15	5	11	6	13	5	17	4	17	7	17	9	26	11	25	7
Fringillidae	*Icterus leucopteryx*			x	2	30	0x	30	4x	26	4x	57	4x	57	0x	30	4x	26	4x	57	4x
Fringillidae	*Icterus auratus*				2	3	3	19	0	15	3	27	3	7	5	22	13	17	12	32	6
Fringillidae	*Icterus mesomelas*				2	12	4	17	5	14	6	23	3	16	5	22	9	21	10	29	5
Fringillidae	*Icterus auricapillus*				2	21	5	15	8	14	8	21	5	25	10	21	13	30	18	34	9
Fringillidae	*Icterus graceannae*				2	2	7	0	11	0	11	1	11	2	10	0	17	1	18	1	17
Fringillidae	*Icterus pectoralis*				2	9	1	18	0	18	2	27	0	11	1	18	6	22	6	29	0
Fringillidae	*Icterus gularis*				2	3	5	13	2	10	4	17	2	5	6	14	20	12	8	19	4
Fringillidae	*Icterus pustulatus*				2	10	10	23	5	13	9	32	4	15	11	23	16	19	14	37	7
Fringillidae	*Icterus icterus*				2	30	4	22	4	26	5	31	5	35	8	32	7	43	12	41	12
Fringillidae	*Icterus croconotus*				14	5	10	3	9	4	9	7	8	8	11	9	14	11	14	16	13
Fringillidae	*Icterus jamacaii*				2	8	16	5	14	5	16	14	9	9	20	8	18	13	22	23	15
Fringillidae	*Icterus galbula*		H		2	0	16	0	9	0	12	0	7	0	19	0	11	0	14	0	8
Fringillidae	*Icterus bullockii*		H		2	0	19	0	16	0	19	0	14	0	22	0	25	0	30	0	22
Fringillidae	*Icterus abeillei*				2	15	6	28	2	20	5	32	0	17	6	28	15	25	9	36	2
Fringillidae	*Icterus cucullatus*				2	1	24	4	21	1	24	7	17	2	30	5	39	2	40	8	28
Fringillidae	*Icterus spurius*		H		2	0	15	0	9	0	12	0	6	0	19	0	13	0	16	0	8
Fringillidae	*Icterus wagleri*				2	13	6	29	3	17	6	36	3	16	7	29	13	23	12	39	5
Fringillidae	*Icterus dominicensis*				2	11	11	25	9	16	8	32	4	17	11	26	11	19	8	34	4
Fringillidae	*Icterus prosthemelas*				14	13	4	23	4	18	5	33	3	16	5	30	10	25	9	39	5
Fringillidae	*Icterus oberi*	T		x	2	100	0x	100	0x	100	0x	100	0x	100	0x	100	0x	100	0x	100	0x
Fringillidae	*Icterus bonana*	T		x	2	0	20x	20	0x	0	20x	20	0x	0	20x	90	0x	0	20x	90	0x
Fringillidae	*Icterus laudabilis*			x	2	0	53x	32	53x	53	0x	53	0x	0	53x	32	53x	53	0x	84	0x
Fringillidae	*Icterus maculialatus*				2	7	0	16	0	15	0	20	0	10	0	17	11	18	2	20	0
Fringillidae	*Icterus parisorum*				2	3	17	8	15	4	17	10	15	4	20	8	27	6	30	12	26
Fringillidae	*Nesopsar nigerrimus*	T		x	2	17	0x	17	5x	11	5x	44	5x	44	0x	17	5x	11	5x	44	5x
Fringillidae	*Gymnomystax mexicanus*		H		2	0	3	0	3	0	2	0	2	0	4	0	4	0	4	0	4
Fringillidae	*Xanthocephalus xanthocep*		H		2	0	13	0	10	0	12	0	8	0	15	0	12	0	15	0	10
Fringillidae	*Agelaius flavus*	T			2	7	1	7	1	10	1	8	1	11	2	13	2	16	2	15	3
Fringillidae	*Agelaius xanthophthalmus*			x	14	100	0x	0	0x	100	0x	100	0x	100	0x	100	0x	100	0x	100	0x
Fringillidae	*Agelaius thilius*				2	3	5	3	5	4	5	3	5	4	7	5	8	6	10	6	9
Fringillidae	*Agelaius cyanopus*		H		2	0	13	0	9	0	12	0	9	0	17	0	12	0	15	0	11
Fringillidae	*Agelaius phoeniceus*		H		2	0	17	0	14	0	16	0	12	0	21	0	21	0	24	0	17
Fringillidae	*Agelaius assimilis*			x	2	2	9x	31	9x	36	0x	97	0x	2	9x	31	12x	36	0x	97	0x
Fringillidae	*Agelaius tricolor*		H		2	0	66	0	42	0	65	0	28	0	67	0	49	0	65	0	28
Fringillidae	*Agelaius icterocephalus*				2	16	5	13	5	14	5	18	3	19	7	18	7	26	9	27	6

Family	Species	IUCN	Ag	R	Range Source	Percentage loss in geographic range 2050								2100							
						TG		AM		GO		OS		TG		AM		GO		OS	
						H	C	H	C	H	C	H	C	H	C	H	C	H	C	H	C
Fringillidae	*Agelaius humeralis*		H		2	0	6	0	5	0	5	0	3	0	6	0	7	0	5	0	3
Fringillidae	*Agelaius xanthomus*	T		x	2	15	0x	15	0x	15	0x	15	0x	15	0x	15	0x	15	0x	15	0x
Fringillidae	*Agelaius ruficapillus*				2	8	13	7	8	9	9	12	6	11	18	11	12	17	13	20	9
Fringillidae	*Leistes militaris*		H		14	0	6	0	7	0	6	0	5	0	9	0	10	0	12	0	8
Fringillidae	*Leistes superciliaris*		H		2	0	12	0	11	0	11	0	9	0	15	0	15	0	15	0	13
Fringillidae	*Sturnella bellicosa*		H		2	0	21	0	20	0	25	0	17	0	29	0	38	0	41	0	34
Fringillidae	*Sturnella militaris*	T			2	1	7	0	7	0	8	0	6	1	9	0	13	0	16	0	12
Fringillidae	*Sturnella loyca*		H		2	0	3	0	3	0	3	0	3	0	4	0	5	0	8	0	5
Fringillidae	*Sturnella magna*		H		2	0	12	0	8	0	10	0	6	0	15	0	15	0	18	0	10
Fringillidae	*Sturnella neglecta*		H		2,11	0	16	0	13	0	15	0	11	0	18	0	18	0	22	0	17
Fringillidae	*Pseudoleistes guirahuro*		H		2	0	10	0	4	0	6	0	4	0	16	0	8	0	8	0	6
Fringillidae	*Pseudoleistes virescens*		H		2	0	2	0	1	0	2	0	1	0	2	0	2	0	3	0	2
Fringillidae	*Amblyramphus holosericeu*				2	6	4	5	4	9	4	10	4	9	5	10	5	15	5	14	5
Fringillidae	*Hypopyrrhus pyrohypogast*	T		x	2	23	0x	18	0x	8	0x	28	0x	29	0x	25	0x	8	0x	28	0x
Fringillidae	*Curaeus curaeus*		H		2	0	10	0	10	0	11	0	10	0	18	0	20	0	21	0	20
Fringillidae	*Curaeus forbesi*	T	H		2	0	27	0	19	0	27	0	27	0	27	0	19	0	29	0	29
Fringillidae	*Gnorimopsar chopi*		H		2	0	15	0	10	0	12	0	8	0	20	0	14	0	15	0	11
Fringillidae	*Oreopsar bolivianus*				14	19	9	33	13	19	13	33	4	32	14	38	18	40	17	50	4
Fringillidae	*Lampropsar tanagrinus*				2	5	10	3	10	3	10	6	10	9	11	7	11	9	12	13	11
Fringillidae	*Macroagelaius imthurni*				2	9	12	2	21	7	17	2	21	11	20	9	27	14	22	6	34
Fringillidae	*Macroagelaius subalaris*	T		x	2	30	0x	33	0x	7	0x	32	0x	48	0x	50	0x	42	8x	40	10x
Fringillidae	*Dives atroviolacea*		H		2	0	6	0	5	0	5	0	3	0	6	0	7	0	5	0	3
Fringillidae	*Dives dives*				2	9	3	16	2	13	4	23	2	11	4	18	11	17	7	26	4
Fringillidae	*Dives warszewiczi*				2	3	16	5	15	5	19	9	11	4	25	6	28	9	29	13	21
Fringillidae	*Quiscalus mexicanus*		H		2	0	14	0	12	0	14	0	11	0	17	0	23	0	24	0	19
Fringillidae	*Quiscalus major*		H		2	0	67	0	40	0	57	0	30	0	71	0	46	0	60	0	32
Fringillidae	*Quiscalus nicaraguensis*		H	x	2	0	0x	0	0x	0	0x	0	0x	0	0x	0	4x	0	0x	0	0x
Fringillidae	*Quiscalus quiscula*		H		2	0	19	0	12	0	16	0	10	0	22	0	16	0	20	0	12
Fringillidae	*Quiscalus niger*		H		2	0	7	0	6	0	8	0	1	0	8	0	8	0	8	0	1
Fringillidae	*Quiscalus lugubris*		H		2	0	6	0	6	0	5	0	4	0	7	0	8	0	8	0	6
Fringillidae	*Euphagus carolinus*				2	3	17	5	16	5	17	5	16	5	21	6	23	8	23	7	22
Fringillidae	*Euphagus cyanocephalus*		H		2	0	21	0	16	0	20	0	13	0	24	0	21	0	25	0	17
Fringillidae	*Molothrus badius*		H		2	0	10	0	9	0	10	0	7	0	13	0	13	0	14	0	11
Fringillidae	*Molothrus rufoaxillaris*		H		2	0	6	0	4	0	5	0	4	0	9	0	8	0	8	0	7
Fringillidae	*Molothrus bonariensis*		H		2	0	11	0	10	0	11	0	8	0	15	0	14	0	15	0	12
Fringillidae	*Molothrus aeneus*		H		2	0	11	0	7	0	9	0	6	0	13	0	21	0	18	0	11
Fringillidae	*Molothrus armenti*			x	14	0	0x	0	0x	0	0x	0	0x	0	0x	0	0x	0	0x	0	0x
Fringillidae	*Molothrus ater*				2	6	19	10	14	9	17	10	12	9	23	14	20	16	24	15	16
Fringillidae	*Molothrus oryzivora*		H		2	0	11	0	11	0	11	0	10	0	14	0	14	0	15	0	12
Fringillidae	*Dolichonyx oryzivorus*		H		2	0	14	0	10	0	12	0	6	0	16	0	11	0	14	0	7
