# Supplementary material for: Projected Impacts of Climate and Land-Use Change on the Global Diversity of Birds
Source: PLoS Biol. 2007 Jun 5;5(6):e157. doi: 10.1371/journal.pbio.0050157 (PMC1885834; doi:10.1371/journal.pbio.0050157)
Supplement: Supplementary file 6 [file pbio.0050157.st003.pdf]

Table S3: Species not analyzed (1,125 species).

IUCN: ‘T’ indicates considered ‘threatened’ by IUCN (Red List, 2004).

R: ‘x’ indicates currently less than 20,000km^2 refined extent of occurrence range size.

Supplementary Table 3

Family	*Species*	*IUCN*	*R*
Megapodiidae	*Megapodius laperouse*	T	x
Megapodiidae	*Megapodius pritchardii*	T	x
Anhimidae	*Anhima cornuta*		
Anhimidae	*Chauna chavaria*		
Anhimidae	*Chauna torquata*		
Anseranatidae	*Anseranas semipalmata*		
Dendrocygnidae	*Dendrocygna guttata*		
Dendrocygnidae	*Dendrocygna eytoni*		
Dendrocygnidae	*Dendrocygna bicolor*		
Dendrocygnidae	*Dendrocygna arcuata*		
Dendrocygnidae	*Dendrocygna javanica*		
Dendrocygnidae	*Dendrocygna viduata*		
Dendrocygnidae	*Dendrocygna arborea*	T	
Dendrocygnidae	*Dendrocygna autumnalis*		
Dendrocygnidae	*Thalassornis leuconotus*		
Anatidae	*Nomonyx dominica*		
Anatidae	*Oxyura jamaicensis*		
Anatidae	*Oxyura leucocephala*	T	
Anatidae	*Oxyura maccoa*		
Anatidae	*Oxyura vittata*		
Anatidae	*Oxyura australis*		
Anatidae	*Biziura lobata*		
Anatidae	*Heteronetta atricapilla*		
Anatidae	*Stictonetta naevosa*		
Anatidae	*Cygnus olor*		
Anatidae	*Cygnus atratus*		
Anatidae	*Cygnus melanocorypha*		
Anatidae	*Cygnus cygnus*		
Anatidae	*Cygnus buccinator*		
Anatidae	*Cygnus columbianus*		
Anatidae	*Coscoroba coscoroba*		
Anatidae	*Anser cygnoides*	T	
Anatidae	*Anser brachyrhynchus*		
Anatidae	*Anser fabalis*		
Anatidae	*Anser albifrons*		
Anatidae	*Anser erythropus*	T	
Anatidae	*Anser anser*		
Anatidae	*Anser indicus*		
Anatidae	*Anser caerulescens*		
Anatidae	*Anser rossii*		
Anatidae	*Anser canagica*		
Anatidae	*Branta sandvicensis*	T	x

Family	*Species*	*IUCN*	*R*
Anatidae	*Branta canadensis*		
Anatidae	*Branta leucopsis*		
Anatidae	*Branta bernicla*		
Anatidae	*Branta ruficollis*	T	
Anatidae	*Cereopsis novaehollandiae*		x
Anatidae	*Plectropterus gambensis*		
Anatidae	*Sarkidiornis melanotos*		
Anatidae	*Tadorna ferruginea*		
Anatidae	*Tadorna cana*		
Anatidae	*Tadorna tadornoides*		
Anatidae	*Tadorna variegata*		x
Anatidae	*Tadorna tadorna*		
Anatidae	*Tadorna radjah*		
Anatidae	*Malacorhynchus membranac*		
Anatidae	*Alopochen aegyptiacus*		
Anatidae	*Neochen jubata*		
Anatidae	*Chloephaga melanoptera*		
Anatidae	*Chloephaga picta*		
Anatidae	*Chloephaga hybrida*		
Anatidae	*Chloephaga poliocephala*		
Anatidae	*Chloephaga rubidiceps*		
Anatidae	*Cyanochen cyanopterus*		
Anatidae	*Hymenolaimus malacorhync*	T	x
Anatidae	*Merganetta armata*		
Anatidae	*Tachyeres pteneres*		
Anatidae	*Tachyeres leucocephalus*		x
Anatidae	*Tachyeres brachypterus*		x
Anatidae	*Tachyeres patachonicus*		
Anatidae	*Pteronetta hartlaubii*		
Anatidae	*Cairina moschata*		
Anatidae	*Cairina scutulata*	T	
Anatidae	*Aix sponsa*		
Anatidae	*Aix galericulata*		
Anatidae	*Lophonetta specularioides*		
Anatidae	*Nettapus pulchellus*		
Anatidae	*Nettapus coromandelianus*		
Anatidae	*Nettapus auritus*		
Anatidae	*Callonetta leucophrys*		
Anatidae	*Chenonetta jubata*		
Anatidae	*Amazonetta brasiliensis*		
Anatidae	*Salvadorina waigiuensis*	T	x
Anatidae	*Anas specularis*		
Anatidae	*Anas capensis*		
Anatidae	*Anas strepera*		

Family	Species	IUCN	R
Anatidae	*Anas falcata*		
Anatidae	*Anas penelope*		
Anatidae	*Anas americana*		
Anatidae	*Anas sibilatrix*		
Anatidae	*Anas sparsa*		
Anatidae	*Anas rubripes*		
Anatidae	*Anas platyrhynchos*		
Anatidae	*Anas poecilorhyncha*		
Anatidae	*Anas superciliosa*		
Anatidae	*Anas luzonica*	T	
Anatidae	*Anas undulata*		
Anatidae	*Anas melleri*	T	
Anatidae	*Anas discors*		
Anatidae	*Anas cyanoptera*		
Anatidae	*Anas smithii*		
Anatidae	*Anas platalea*		
Anatidae	*Anas rhynchotis*		
Anatidae	*Anas clypeata*		
Anatidae	*Anas bernieri*	T	
Anatidae	*Anas gibberifrons*		
Anatidae	*Anas castanea*		
Anatidae	*Anas aucklandica*	T	x
Anatidae	*Anas bahamensis*		
Anatidae	*Anas erythrorhyncha*		
Anatidae	*Anas flavirostris*		
Anatidae	*Anas acuta*		
Anatidae	*Anas georgica*		
Anatidae	*Anas querquedula*		
Anatidae	*Anas formosa*	T	
Anatidae	*Anas crecca*		
Anatidae	*Anas versicolor*		
Anatidae	*Anas hottentota*		
Anatidae	*Marmaronetta angustirostris*	T	
Anatidae	*Netta rufina*		
Anatidae	*Netta peposaca*		
Anatidae	*Netta erythrophthalma*		
Anatidae	*Aythya ferina*		
Anatidae	*Aythya valisineria*		
Anatidae	*Aythya americana*		
Anatidae	*Aythya collaris*		
Anatidae	*Aythya nyroca*		
Anatidae	*Aythya innotata*	T	x
Anatidae	*Aythya baeri*	T	
Anatidae	*Aythya australis*		

Family	*Species*	*IUCN*	*R*
Anatidae	*Aythya fuligula*		
Anatidae	*Aythya novaeseelandiae*		x
Anatidae	*Aythya marila*		
Anatidae	*Aythya affinis*		
Anatidae	*Somateria mollissima*		
Anatidae	*Somateria spectabilis*		
Anatidae	*Somateria fischeri*		
Anatidae	*Polysticta stelleri*		
Anatidae	*Histrionicus histrionicus*		
Anatidae	*Clangula hyemalis*		
Anatidae	*Melanitta nigra*		
Anatidae	*Melanitta perspicillata*		
Anatidae	*Melanitta fusca*		
Anatidae	*Bucephala clangula*		
Anatidae	*Bucephala islandica*		
Anatidae	*Bucephala albeola*		
Anatidae	*Mergellus albellus*		
Anatidae	*Lophodytes cucullatus*		
Anatidae	*Mergus octosetaceus*	T	x
Anatidae	*Mergus serrator*		
Anatidae	*Mergus squamatus*	T	
Anatidae	*Mergus merganser*		x
Bucerotidae	*Aceros narcondami*	T	x
Alcedinidae	*Alcedo hercules*		
Alcedinidae	*Alcedo atthis*		
Alcedinidae	*Alcedo semitorquata*		
Alcedinidae	*Alcedo quadribrachys*		
Alcedinidae	*Alcedo meninting*		
Alcedinidae	*Alcedo azurea*		
Alcedinidae	*Alcedo websteri*	T	
Alcedinidae	*Alcedo euryzona*	T	
Alcedinidae	*Alcedo cyanopecta*		
Alcedinidae	*Alcedo argentata*	T	
Alcedinidae	*Alcedo cristata*		
Alcedinidae	*Alcedo vintsioides*		
Alcedinidae	*Alcedo thomensis*		x
Alcedinidae	*Alcedo nais*		x
Alcedinidae	*Alcedo leucogaster*		
Alcedinidae	*Alcedo coerulescens*		
Alcedinidae	*Alcedo pusilla*		
Halcyonidae	*Pelargopsis capensis*		
Halcyonidae	*Pelargopsis melanorhyncha*		
Halcyonidae	*Halcyon coromanda*		
Halcyonidae	*Halcyon pileata*		

Family	Species	IUCN	R
Halcyonidae	*Halcyon senegaloides*		
Halcyonidae	*Todiramphus nigrocyaneus*		
Halcyonidae	*Todiramphus cinnamominus*		x
Halcyonidae	*Todiramphus veneratus*		x
Halcyonidae	*Todiramphus tuta*		x
Halcyonidae	*Todiramphus godeffroyi*	T	x
Halcyonidae	*Todiramphus gambieri*	T	x
Cerylidae	*Megaceryle maxima*		
Cerylidae	*Megaceryle lugubris*		
Cerylidae	*Megaceryle alcyon*		
Cerylidae	*Megaceryle torquata*		
Cerylidae	*Ceryle rudis*		
Cerylidae	*Chloroceryle amazona*		
Cerylidae	*Chloroceryle americana*		
Cerylidae	*Chloroceryle inda*		
Cerylidae	*Chloroceryle aenea*		
Coccyzidae	*Coccyzus ferrugineus*	T	x
Psittacidae	*Trichoglossus rubiginosus*		x
Psittacidae	*Vini kuhlii*	T	x
Psittacidae	*Vini stepheni*	T	x
Psittacidae	*Vini peruviana*	T	x
Psittacidae	*Vini ultramarina*	T	x
Psittacidae	*Cyanoramphus unicolor*	T	x
Apodidae	*Aerodramus elaphrus*	T	x
Apodidae	*Aerodramus pelewensis*		x
Apodidae	*Aerodramus inquieta*		x
Apodidae	*Aerodramus sawtelli*	T	x
Apodidae	*Aerodramus bartschi*	T	x
Apodidae	*Aerodramus leucophaeus*	T	x
Strigidae	*Otus insularis*	T	x
Strigidae	*Otus podarginus*		x
Strigidae	*Ketupa blakistoni*	T	
Strigidae	*Ketupa zeylonensis*		
Strigidae	*Ketupa flavipes*		
Strigidae	*Ketupa ketupu*		
Strigidae	*Scotopelia peli*		
Strigidae	*Scotopelia ussheri*	T	
Strigidae	*Scotopelia bouvieri*		
Strigidae	*Ninox natalis*	T	x
Columbidae	*Gallicolumba kubaryi*	T	x
Columbidae	*Gallicolumba erythroptera*	T	x
Columbidae	*Gallicolumba xanthonura*		x
Columbidae	*Gallicolumba rubescens*	T	x
Columbidae	*Gallicolumba canifrons*		x

Family	*Species*	*IUCN*	*R*
Columbidae	*Ptilinopus roseicapilla*	T	x
Columbidae	*Ptilinopus pelewensis*		x
Columbidae	*Ptilinopus rarotongensis*	T	x
Columbidae	*Ptilinopus huttoni*	T	x
Columbidae	*Ptilinopus purpuratus*		x
Columbidae	*Ptilinopus coralensis*		x
Columbidae	*Ptilinopus chalcurus*	T	x
Columbidae	*Ptilinopus insularis*	T	x
Columbidae	*Ptilinopus mercierii*		x
Columbidae	*Ptilinopus dupetithouarsii*		x
Columbidae	*Alectroenas pulcherrima*		x
Columbidae	*Ducula oceanica*		x
Columbidae	*Ducula aurorae*	T	x
Columbidae	*Ducula galeata*	T	x
Columbidae	*Ducula whartoni*	T	x
Eurypygidae	*Eurypyga helias*		
Gruidae	*Balearica pavonina*		
Gruidae	*Balearica regulorum*		
Gruidae	*Grus leucogeranus*	T	
Gruidae	*Grus antigone*	T	
Gruidae	*Grus rubicunda*		
Gruidae	*Grus vipio*	T	
Gruidae	*Grus canadensis*		
Gruidae	*Grus carunculatus*	T	
Gruidae	*Grus grus*		
Gruidae	*Grus monacha*	T	
Gruidae	*Grus americana*	T	x
Gruidae	*Grus nigricollis*	T	x
Gruidae	*Grus japonensis*	T	
Aramidae	*Aramus guarauna*		
Heliornithidae	*Podica senegalensis*		
Heliornithidae	*Heliopais personata*	T	
Heliornithidae	*Heliornis fulica*		
Rallidae	*Sarothrura rufa*		
Rallidae	*Sarothrura ayresi*	T	
Rallidae	*Sarothrura watersi*	T	
Rallidae	*Coturnicops exquisitus*	T	
Rallidae	*Coturnicops noveboracensis*		
Rallidae	*Rallina canningi*		x
Rallidae	*Rallina fasciata*		
Rallidae	*Laterallus levraudi*	T	
Rallidae	*Laterallus ruber*		
Rallidae	*Laterallus albigularis*		
Rallidae	*Laterallus exilis*		

Family	Species	IUCN	R
Rallidae	*Laterallus jamaicensis*		
Rallidae	*Gallirallus sylvestris*	T	x
Rallidae	*Gallirallus philippensis*		
Rallidae	*Gallirallus owstoni*		x
Rallidae	*Gallirallus striatus*		
Rallidae	*Rallus longirostris*		
Rallidae	*Rallus elegans*		
Rallidae	*Rallus wetmorei*	T	x
Rallidae	*Rallus limicola*		
Rallidae	*Rallus semiplumbeus*	T	
Rallidae	*Rallus antarcticus*	T	
Rallidae	*Rallus aquaticus*		
Rallidae	*Rallus caerulescens*		
Rallidae	*Rallus madagascariensis*	T	
Rallidae	*Lewinia pectoralis*		
Rallidae	*Lewinia muelleri*	T	x
Rallidae	*Dryolimnas cuvieri*		
Rallidae	*Rougetius rougetii*		
Rallidae	*Atlantisia rogersi*	T	x
Rallidae	*Aramides mangle*		
Rallidae	*Aramides cajanea*		
Rallidae	*Aramides calopterus*		
Rallidae	*Amaurolimnas concolor*		
Rallidae	*Amaurornis akool*		
Rallidae	*Amaurornis olivaceus*		
Rallidae	*Amaurornis moluccanus*		
Rallidae	*Amaurornis flavirostra*		
Rallidae	*Amaurornis bicolor*		
Rallidae	*Porzana parva*		
Rallidae	*Porzana pusilla*		
Rallidae	*Porzana porzana*		
Rallidae	*Porzana fluminea*		
Rallidae	*Porzana carolina*		
Rallidae	*Porzana spiloptera*	T	
Rallidae	*Porzana fusca*		
Rallidae	*Porzana atra*	T	x
Rallidae	*Porzana flaviventer*		
Rallidae	*Porzana cinerea*		
Rallidae	*Pardirallus maculatus*		
Rallidae	*Pardirallus nigricans*		
Rallidae	*Pardirallus sanguinolentus*		
Rallidae	*Eulabeornis castaneoventris*		
Rallidae	*Habroptila wallacii*	T	x
Rallidae	*Gallicrex cinerea*		

Family	*Species*	*IUCN*	*R*
Rallidae	*Porphyrio porphyrio*		
Rallidae	*Porphyrio alleni*		
Rallidae	*Porphyrio martinicus*		
Rallidae	*Porphyrio flavirostris*		
Rallidae	*Gallinula nesiotis*	T	x
Rallidae	*Gallinula chloropus*		
Rallidae	*Gallinula tenebrosa*		
Rallidae	*Gallinula angulata*		
Rallidae	*Gallinula melanops*		
Rallidae	*Gallinula ventralis*		
Rallidae	*Gallinula mortierii*		x
Rallidae	*Fulica cristata*		
Rallidae	*Fulica atra*		
Rallidae	*Fulica alai*	T	x
Rallidae	*Fulica americana*		
Rallidae	*Fulica caribaea*		x
Rallidae	*Fulica leucoptera*		
Rallidae	*Fulica ardesiaca*		x
Rallidae	*Fulica armillata*		
Rallidae	*Fulica rufifrons*		
Rallidae	*Fulica gigantea*		
Rallidae	*Fulica cornuta*		x
Scolopacidae	*Gallinago gallinago*		
Scolopacidae	*Gallinago nigripennis*		
Scolopacidae	*Gallinago macrodactyla*		
Scolopacidae	*Lymnocryptes minimus*		
Scolopacidae	*Numenius madagascariensis*		
Scolopacidae	*Tringa erythropus*		
Scolopacidae	*Tringa totanus*		
Scolopacidae	*Tringa stagnatilis*		
Scolopacidae	*Tringa nebularia*		
Scolopacidae	*Tringa guttifer*	T	
Scolopacidae	*Tringa melanoleuca*		
Scolopacidae	*Tringa flavipes*		
Scolopacidae	*Tringa solitaria*		
Scolopacidae	*Tringa ochropus*		
Scolopacidae	*Tringa glareola*		
Scolopacidae	*Tringa cinerea*		
Scolopacidae	*Tringa hypoleucos*		
Scolopacidae	*Tringa macularia*		
Scolopacidae	*Tringa brevipes*		
Scolopacidae	*Tringa incana*		
Scolopacidae	*Catoptrophorus semipalmatu*		
Scolopacidae	*Prosobonia cancellata*	T	x

Family	Species	IUCN	R
Scolopacidae	*Limnodromus griseus*		
Scolopacidae	*Limnodromus scolopaceus*		
Scolopacidae	*Limnodromus semipalmatus*		
Scolopacidae	*Calidris pusilla*		
Scolopacidae	*Calidris subminuta*		
Scolopacidae	*Calidris minutilla*		
Scolopacidae	*Calidris alpina*		
Scolopacidae	*Micropalama himantopus*		
Scolopacidae	*Eurynorhynchus pygmeus*	T	
Scolopacidae	*Philomachus pugnax*		
Scolopacidae	*Phalaropus tricolor*		
Scolopacidae	*Phalaropus lobatus*		
Scolopacidae	*Phalaropus fulicaria*		
Rostratulidae	*Rostratula benghalensis*		
Rostratulidae	*Rostratula semicollaris*		
Jacanidae	*Actophilornis africanus*		
Jacanidae	*Actophilornis albinucha*		
Jacanidae	*Microparra capensis*		
Jacanidae	*Irediparra gallinacea*		
Jacanidae	*Hydrophasianus chirurgus*		
Jacanidae	*Metopidius indicus*		
Jacanidae	*Jacana jacana*		
Chionidae	*Chionis alba*		
Chionidae	*Chionis minor*		x
Pluvianellidae	*Pluvianellus socialis*		x
Burhinidae	*Burhinus senegalensis*		
Burhinidae	*Burhinus vermiculatus*		
Burhinidae	*Burhinus recurvirostris*		
Burhinidae	*Esacus neglectus*		
Charadriidae	*Haematopus ostralegus*		
Charadriidae	*Haematopus moquini*		
Charadriidae	*Haematopus bachmani*		
Charadriidae	*Haematopus palliatus*		
Charadriidae	*Haematopus longirostris*		x
Charadriidae	*Haematopus unicolor*		
Charadriidae	*Haematopus chathamensis*	T	x
Charadriidae	*Haematopus fuliginosus*		
Charadriidae	*Haematopus ater*		
Charadriidae	*Haematopus leucopodus*		
Charadriidae	*Ibidorhyncha struthersii*		
Charadriidae	*Himantopus himantopus*		
Charadriidae	*Himantopus novaezelandiae*	T	x
Charadriidae	*Cladorhynchus leucocephalu*		
Charadriidae	*Recurvirostra avosetta*		

Family	Species	IUCN	R
Charadriidae	*Recurvirostra americana*		
Charadriidae	*Recurvirostra novaehollandia*		
Charadriidae	*Recurvirostra andina*		
Charadriidae	*Charadrius obscurus*	T	x
Charadriidae	*Charadrius placidus*		
Charadriidae	*Charadrius dubius*		
Charadriidae	*Charadrius wilsonia*		
Charadriidae	*Charadrius sanctaehelenae*	T	x
Charadriidae	*Charadrius pecuarius*		
Charadriidae	*Charadrius tricollaris*		
Charadriidae	*Charadrius melodus*	T	
Charadriidae	*Charadrius pallidus*		
Charadriidae	*Charadrius alexandrinus*		
Charadriidae	*Charadrius marginatus*		
Charadriidae	*Charadrius ruficapillus*		
Charadriidae	*Charadrius peronii*		
Charadriidae	*Charadrius javanicus*		x
Charadriidae	*Charadrius collaris*		
Charadriidae	*Charadrius bicinctus*		
Charadriidae	*Charadrius falklandicus*		
Charadriidae	*Charadrius alticola*		
Charadriidae	*Charadrius mongolus*		
Charadriidae	*Thinornis rubricollis*		x
Charadriidae	*Thinornis novaeseelandiae*	T	x
Charadriidae	*Erythrogonys cinctus*		
Charadriidae	*Phegornis mitchellii*		
Charadriidae	*Elseyornis melanops*		
Charadriidae	*Vanellus crassirostris*		
Charadriidae	*Vanellus armatus*		
Charadriidae	*Vanellus spinosus*		
Charadriidae	*Vanellus duvaucelii*		
Charadriidae	*Vanellus albiceps*		
Charadriidae	*Vanellus leucurus*		
Glareolidae	*Pluvianus aegyptius*		
Laridae	*Stercorarius parasiticus*		
Laridae	*Stercorarius pomarinus*		
Laridae	*Stercorarius skua*		
Laridae	*Stercorarius maccormicki*		
Laridae	*Stercorarius chilensis*		
Laridae	*Stercorarius antarctica*		
Laridae	*Rynchops niger*		
Laridae	*Rynchops flavirostris*		
Laridae	*Rynchops albicollis*	T	
Laridae	*Larus scoresbii*		

Family	Species	IUCN	R
Laridae	*Larus pacificus*		
Laridae	*Larus belcheri*		
Laridae	*Larus atlanticus*	T	
Laridae	*Larus crassirostris*		
Laridae	*Larus heermanni*		
Laridae	*Larus leucophthalmus*		
Laridae	*Larus hemprichii*		
Laridae	*Larus canus*		
Laridae	*Larus delawarensis*		
Laridae	*Larus audouinii*		
Laridae	*Larus californicus*		
Laridae	*Larus marinus*		
Laridae	*Larus dominicanus*		
Laridae	*Larus glaucescens*		
Laridae	*Larus occidentalis*		
Laridae	*Larus livens*		
Laridae	*Larus hyperboreus*		
Laridae	*Larus glaucoides*		
Laridae	*Larus thayeri*		
Laridae	*Larus argentatus*		
Laridae	*Larus schistisagus*		
Laridae	*Larus cachinnans*		
Laridae	*Larus armenicus*		x
Laridae	*Larus fuscus*		
Laridae	*Larus ichthyaetus*		
Laridae	*Larus brunnicephalus*		
Laridae	*Larus cirrocephalus*		
Laridae	*Larus hartlaubii*		
Laridae	*Larus novaehollandiae*		
Laridae	*Larus scopulinus*		
Laridae	*Larus bulleri*	T	x
Laridae	*Larus maculipennis*		
Laridae	*Larus ridibundus*		
Laridae	*Larus genei*		
Laridae	*Larus philadelphia*		
Laridae	*Larus saundersi*	T	
Laridae	*Larus serranus*		x
Laridae	*Larus melanocephalus*		
Laridae	*Larus relictus*	T	
Laridae	*Larus fuliginosus*	T	x
Laridae	*Larus atricilla*		
Laridae	*Larus pipixcan*		
Laridae	*Larus minutus*		
Laridae	*Pagophila eburnea*		

Family	*Species*	*IUCN*	*R*
Laridae	*Rhodostethia rosea*		
Laridae	*Xema sabini*		
Laridae	*Creagrus furcatus*		x
Laridae	*Rissa tridactyla*		
Laridae	*Rissa brevirostris*	T	
Laridae	*Sterna nilotica*		
Laridae	*Sterna caspia*		
Laridae	*Sterna maxima*		
Laridae	*Sterna bergii*		
Laridae	*Sterna elegans*		
Laridae	*Sterna bengalensis*		
Laridae	*Sterna bernsteini*	T	
Laridae	*Sterna sandvicensis*		
Laridae	*Sterna aurantia*		
Laridae	*Sterna dougallii*		
Laridae	*Sterna striata*		
Laridae	*Sterna sumatrana*		
Laridae	*Sterna hirundinacea*		
Laridae	*Sterna hirundo*		
Laridae	*Sterna paradisaea*		
Laridae	*Sterna vittata*		
Laridae	*Sterna virgata*		x
Laridae	*Sterna forsteri*		
Laridae	*Sterna trudeaui*		
Laridae	*Sterna albifrons*		
Laridae	*Sterna saundersi*		
Laridae	*Sterna antillarum*		
Laridae	*Sterna superciliaris*		
Laridae	*Sterna lorata*		
Laridae	*Sterna nereis*		
Laridae	*Sterna balaenarum*		
Laridae	*Sterna repressa*		
Laridae	*Sterna acuticauda*		
Laridae	*Sterna aleutica*		
Laridae	*Sterna lunata*		
Laridae	*Sterna anaethetus*		
Laridae	*Sterna fuscata*		
Laridae	*Chlidonias albostriatus*	T	x
Laridae	*Chlidonias hybridus*		
Laridae	*Chlidonias leucopterus*		
Laridae	*Chlidonias niger*		
Laridae	*Phaetusa simplex*		
Laridae	*Anous minutus*		
Laridae	*Anous tenuirostris*		x

Family	Species	IUCN	R
Laridae	*Procelsterna cerulea*		
Laridae	*Procelsterna albivitta*		x
Laridae	*Gygis alba*		
Laridae	*Larosterna inca*		
Laridae	*Alle alle*		
Laridae	*Uria aalge*		
Laridae	*Uria lomvia*		
Laridae	*Alca torda*		
Laridae	*Cepphus grylle*		
Laridae	*Cepphus columba*		
Laridae	*Cepphus carbo*		
Laridae	*Brachyramphus marmoratus*	T	
Laridae	*Brachyramphus brevirostris*	T	
Laridae	*Synthliboramphus hypoleucu*	T	x
Laridae	*Synthliboramphus craveri*	T	x
Laridae	*Synthliboramphus antiquus*		
Laridae	*Synthliboramphus wumizusu*	T	x
Laridae	*Ptychoramphus aleuticus*		
Laridae	*Cyclorrhynchus psittacula*		
Laridae	*Aethia cristatella*		
Laridae	*Aethia pygmaea*		
Laridae	*Aethia pusilla*		
Laridae	*Cerorhinca monocerata*		
Laridae	*Fratercula arctica*		
Laridae	*Fratercula corniculata*		
Laridae	*Fratercula cirrhata*		
Accipitridae	*Pandion haliaetus*		
Accipitridae	*Haliaeetus sanfordi*	T	
Accipitridae	*Haliaeetus vocifer*		
Accipitridae	*Haliaeetus vociferoides*	T	x
Accipitridae	*Haliaeetus leucoryphus*	T	
Accipitridae	*Haliaeetus albicilla*		
Accipitridae	*Haliaeetus leucocephalus*		
Accipitridae	*Haliaeetus pelagicus*	T	
Accipitridae	*Ichthyophaga humilis*		
Accipitridae	*Ichthyophaga ichthyaetus*		
Falconidae	*Falco araea*	T	x
Podicipedidae	*Rollandia rolland*		
Podicipedidae	*Rollandia microptera*	T	x
Podicipedidae	*Tachybaptus ruficollis*		
Podicipedidae	*Tachybaptus novaehollandia*		
Podicipedidae	*Tachybaptus rufolavatus*	T	
Podicipedidae	*Tachybaptus pelzelnii*	T	
Podicipedidae	*Tachybaptus dominicus*		

Family	*Species*	*IUCN*	*R*
Podicipedidae	*Podilymbus podiceps*		
Podicipedidae	*Poliocephalus poliocephalus*		
Podicipedidae	*Poliocephalus rufopectus*	T	
Podicipedidae	*Podiceps major*		
Podicipedidae	*Podiceps grisegena*		
Podicipedidae	*Podiceps cristatus*		
Podicipedidae	*Podiceps auritus*		
Podicipedidae	*Podiceps nigricollis*		
Podicipedidae	*Podiceps occipitalis*		
Podicipedidae	*Podiceps taczanowskii*	T	x
Podicipedidae	*Podiceps gallardoi*		x
Podicipedidae	*Aechmophorus occidentalis*		
Podicipedidae	*Aechmophorus clarkii*		
Phaethontidae	*Phaethon aethereus*		
Phaethontidae	*Phaethon rubricauda*		
Phaethontidae	*Phaethon lepturus*		
Sulidae	*Papasula abbotti*	T	x
Sulidae	*Morus bassanus*		
Sulidae	*Morus capensis*	T	
Sulidae	*Morus serrator*		x
Sulidae	*Sula nebouxii*		x
Sulidae	*Sula variegata*		
Sulidae	*Sula dactylatra*		
Sulidae	*Sula sula*		
Anhingidae	*Anhinga anhinga*		
Anhingidae	*Anhinga melanogaster*		
Phalacrocoracida	*Phalacrocorax africanus*		
Phalacrocoracida	*Phalacrocorax coronatus*		
Phalacrocoracida	*Phalacrocorax pygmeus*		
Phalacrocoracida	*Phalacrocorax niger*		
Phalacrocoracida	*Phalacrocorax melanoleucos*		
Phalacrocoracida	*Phalacrocorax penicillatus*		
Phalacrocoracida	*Phalacrocorax harrisi*	T	x
Phalacrocoracida	*Phalacrocorax neglectus*	T	
Phalacrocoracida	*Phalacrocorax fuscescens*		x
Phalacrocoracida	*Phalacrocorax brasilianus*		
Phalacrocoracida	*Phalacrocorax auritus*		
Phalacrocoracida	*Phalacrocorax fuscicollis*		
Phalacrocoracida	*Phalacrocorax varius*		x
Phalacrocoracida	*Phalacrocorax sulcirostris*		
Phalacrocoracida	*Phalacrocorax carbo*		
Phalacrocoracida	*Phalacrocorax capillatus*		
Phalacrocoracida	*Phalacrocorax nigrogularis*	T	x
Phalacrocoracida	*Phalacrocorax capensis*		

Family	Species	IUCN	R
Phalacrocoracida	*Phalacrocorax bougainvillii*		
Phalacrocoracida	*Phalacrocorax verrucosus*		x
Phalacrocoracida	*Phalacrocorax atriceps*		
Phalacrocoracida	*Phalacrocorax nivalis*		x
Phalacrocoracida	*Phalacrocorax purpurascens*		x
Phalacrocoracida	*Phalacrocorax bransfieldensi*		
Phalacrocoracida	*Phalacrocorax georgianus*		x
Phalacrocoracida	*Phalacrocorax melanogenis*		x
Phalacrocoracida	*Phalacrocorax campbelli*	T	x
Phalacrocoracida	*Phalacrocorax carunculatus*	T	x
Phalacrocoracida	*Phalacrocorax chalconotus*	T	x
Phalacrocoracida	*Phalacrocorax onslowi*	T	x
Phalacrocoracida	*Phalacrocorax colensoi*	T	x
Phalacrocoracida	*Phalacrocorax ranfurlyi*	T	x
Phalacrocoracida	*Phalacrocorax magellanicus*		
Phalacrocoracida	*Phalacrocorax urile*		
Phalacrocoracida	*Phalacrocorax pelagicus*		
Phalacrocoracida	*Phalacrocorax aristotelis*		
Phalacrocoracida	*Phalacrocorax gaimardi*		
Phalacrocoracida	*Phalacrocorax punctatus*		x
Phalacrocoracida	*Phalacrocorax featherstoni*	T	x
Ardeidae	*Tigrisoma mexicanum*		
Ardeidae	*Tigrisoma fasciatum*		
Ardeidae	*Tigrisoma lineatum*		
Ardeidae	*Tigriornis leucolophus*		
Ardeidae	*Cochlearius cochlearia*		
Ardeidae	*Zonerodius heliosylus*		
Ardeidae	*Zebrilus undulatus*		
Ardeidae	*Ixobrychus involucris*		
Ardeidae	*Ixobrychus minutus*		
Ardeidae	*Ixobrychus sinensis*		
Ardeidae	*Ixobrychus exilis*		
Ardeidae	*Ixobrychus eurhythmus*		
Ardeidae	*Ixobrychus cinnamomeus*		
Ardeidae	*Ixobrychus sturmii*		
Ardeidae	*Ixobrychus flavicollis*		
Ardeidae	*Botaurus lentiginosus*		
Ardeidae	*Botaurus pinnatus*		
Ardeidae	*Botaurus stellaris*		
Ardeidae	*Botaurus poiciloptilus*	T	
Ardeidae	*Agamia agami*		
Ardeidae	*Nyctanassa violacea*		
Ardeidae	*Nycticorax nycticorax*		
Ardeidae	*Nycticorax caledonicus*		

Family	*Species*	*IUCN*	*R*
Ardeidae	*Gorsachius leuconotus*		
Ardeidae	*Gorsachius magnificus*	T	x
Ardeidae	*Gorsachius goisagi*	T	x
Ardeidae	*Gorsachius melanolophus*		
Ardeidae	*Syrigma sibilatrix*		
Ardeidae	*Egretta rufescens*		
Ardeidae	*Egretta vinaceigula*	T	
Ardeidae	*Egretta ardesiaca*		
Ardeidae	*Egretta tricolor*		
Ardeidae	*Egretta novaehollandiae*		
Ardeidae	*Egretta caerulea*		
Ardeidae	*Egretta garzetta*		
Ardeidae	*Egretta thula*		
Ardeidae	*Egretta eulophotes*	T	
Ardeidae	*Pilherodius pileatus*		
Ardeidae	*Ardea cinerea*		
Ardeidae	*Ardea herodias*		
Ardeidae	*Ardea cocoi*		
Ardeidae	*Ardea pacifica*		
Ardeidae	*Ardea melanocephala*		
Ardeidae	*Ardea humbloti*	T	
Ardeidae	*Ardea goliath*		
Ardeidae	*Ardea insignis*	T	
Ardeidae	*Ardea sumatrana*		
Ardeidae	*Ardea purpurea*		
Ardeidae	*Ardea picata*		
Ardeidae	*Ardea alba*		
Ardeidae	*Ardea intermedia*		
Ardeidae	*Ardeola ralloides*		
Ardeidae	*Ardeola grayii*		
Ardeidae	*Ardeola bacchus*		
Ardeidae	*Ardeola speciosa*		
Ardeidae	*Ardeola idae*	T	
Ardeidae	*Ardeola rufiventris*		
Ardeidae	*Butorides striatus*		
Scopidae	*Scopus umbretta*		
Phoenicopteridae	*Phoenicopterus ruber*		
Phoenicopteridae	*Phoenicopterus chilensis*		
Phoenicopteridae	*Phoenicopterus minor*		
Phoenicopteridae	*Phoenicopterus andinus*	T	
Phoenicopteridae	*Phoenicopterus jamesi*		
Threskiornithidae	*Eudocimus albus*		
Threskiornithidae	*Eudocimus ruber*		
Threskiornithidae	*Phimosus infuscatus*		

Family	*Species*	*IUCN*	*R*
Threskiornithidae	*Plegadis falcinellus*		
Threskiornithidae	*Plegadis chihi*		
Threskiornithidae	*Plegadis ridgwayi*		
Threskiornithidae	*Cercibis oxycerca*		
Threskiornithidae	*Theristicus caerulescens*		
Threskiornithidae	*Mesembrinibis cayennensis*		
Threskiornithidae	*Bostrychia olivacea*		
Threskiornithidae	*Bostrychia rara*		
Threskiornithidae	*Threskiornis melanocephalus*		
Threskiornithidae	*Threskiornis molucca*		
Threskiornithidae	*Pseudibis papillosa*		
Threskiornithidae	*Pseudibis gigantea*	T	x
Threskiornithidae	*Nipponia nippon*	T	
Threskiornithidae	*Platalea leucorodia*		
Threskiornithidae	*Platalea regia*		
Threskiornithidae	*Platalea alba*		
Threskiornithidae	*Platalea minor*	T	x
Threskiornithidae	*Platalea flavipes*		
Threskiornithidae	*Ajaia ajaja*		
Pelecanidae	*Balaeniceps rex*	T	
Pelecanidae	*Pelecanus onocrotalus*		
Pelecanidae	*Pelecanus rufescens*		
Pelecanidae	*Pelecanus crispus*	T	
Pelecanidae	*Pelecanus philippensis*	T	x
Pelecanidae	*Pelecanus conspicillatus*		
Pelecanidae	*Pelecanus erythrorhynchos*		
Pelecanidae	*Pelecanus occidentalis*		
Ciconiidae	*Mycteria americana*		
Ciconiidae	*Mycteria cinerea*	T	
Ciconiidae	*Mycteria ibis*		
Ciconiidae	*Mycteria leucocephala*		
Ciconiidae	*Anastomus oscitans*		
Ciconiidae	*Anastomus lamelligerus*		
Ciconiidae	*Ciconia nigra*		
Ciconiidae	*Ciconia episcopus*		
Ciconiidae	*Ciconia boyciana*	T	
Ciconiidae	*Ephippiorhynchus asiaticus*		
Ciconiidae	*Ephippiorhynchus senegalen*		
Ciconiidae	*Leptoptilos javanicus*	T	
Ciconiidae	*Leptoptilos dubius*	T	
Fregatidae	*Fregata magnificens*		
Fregatidae	*Fregata aquila*	T	x
Fregatidae	*Fregata minor*		
Fregatidae	*Fregata ariel*		

Family	*Species*	*IUCN*	*R*
Fregatidae	*Fregata andrewsi*	T	x
Spheniscidae	*Aptenodytes patagonicus*		
Spheniscidae	*Aptenodytes forsteri*		
Spheniscidae	*Pygoscelis papua*		
Spheniscidae	*Pygoscelis adeliae*		
Spheniscidae	*Pygoscelis antarctica*		
Spheniscidae	*Eudyptes chrysocome*	T	
Spheniscidae	*Eudyptes pachyrhynchus*	T	x
Spheniscidae	*Eudyptes robustus*	T	x
Spheniscidae	*Eudyptes sclateri*	T	x
Spheniscidae	*Eudyptes chrysolophus*	T	
Spheniscidae	*Eudyptes schlegeli*	T	x
Spheniscidae	*Megadyptes antipodes*	T	x
Spheniscidae	*Eudyptula minor*		
Spheniscidae	*Spheniscus demersus*	T	
Spheniscidae	*Spheniscus humboldti*	T	
Spheniscidae	*Spheniscus magellanicus*		
Spheniscidae	*Spheniscus mendiculus*	T	x
Gaviidae	*Gavia stellata*		
Gaviidae	*Gavia arctica*		
Gaviidae	*Gavia immer*		
Gaviidae	*Gavia adamsii*		
Procellariidae	*Macronectes giganteus*	T	
Procellariidae	*Macronectes halli*		x
Procellariidae	*Fulmarus glacialis*		
Procellariidae	*Fulmarus glacialoides*		x
Procellariidae	*Thalassoica antarctica*		x
Procellariidae	*Daption capense*		
Procellariidae	*Pagodroma nivea*		
Procellariidae	*Lugensa brevirostris*		x
Procellariidae	*Pterodroma aterrima*	T	x
Procellariidae	*Pterodroma rostrata*		x
Procellariidae	*Pterodroma macgillivrayi*	T	x
Procellariidae	*Pterodroma axillaris*	T	x
Procellariidae	*Pterodroma nigripennis*		x
Procellariidae	*Pterodroma cervicalis*	T	x
Procellariidae	*Pterodroma inexpectata*		x
Procellariidae	*Pterodroma hypoleuca*		x
Procellariidae	*Pterodroma leucoptera*	T	
Procellariidae	*Pterodroma cookii*	T	x
Procellariidae	*Pterodroma defilippiana*	T	x
Procellariidae	*Pterodroma pycrofti*	T	
Procellariidae	*Pterodroma alba*	T	x
Procellariidae	*Pterodroma arminjoniana*	T	

Family	*Species*	*IUCN*	*R*
Procellariidae	*Pterodroma phaeopygia*	T	x
Procellariidae	*Pterodroma neglecta*		x
Procellariidae	*Pterodroma externa*	T	x
Procellariidae	*Pterodroma baraui*	T	x
Procellariidae	*Pterodroma ultima*		x
Procellariidae	*Pterodroma solandri*	T	x
Procellariidae	*Pterodroma macroptera*		
Procellariidae	*Pterodroma magentae*	T	x
Procellariidae	*Pterodroma lessonii*		x
Procellariidae	*Pterodroma madeira*	T	x
Procellariidae	*Pterodroma feae*		x
Procellariidae	*Pterodroma mollis*		x
Procellariidae	*Pterodroma incerta*	T	x
Procellariidae	*Pterodroma cahow*	T	x
Procellariidae	*Pterodroma hasitata*	T	
Procellariidae	*Halobaena caerulea*		x
Procellariidae	*Pachyptila vittata*		x
Procellariidae	*Pachyptila salvini*		x
Procellariidae	*Pachyptila desolata*		x
Procellariidae	*Pachyptila belcheri*		x
Procellariidae	*Pachyptila turtur*		
Procellariidae	*Pachyptila crassirostris*		x
Procellariidae	*Bulweria bulwerii*		
Procellariidae	*Bulweria fallax*		x
Procellariidae	*Procellaria aequinoctialis*	T	x
Procellariidae	*Procellaria parkinsoni*	T	x
Procellariidae	*Procellaria westlandica*	T	x
Procellariidae	*Procellaria cinerea*		x
Procellariidae	*Calonectris diomedea*		
Procellariidae	*Calonectris leucomelas*		
Procellariidae	*Puffinus pacificus*		
Procellariidae	*Puffinus bulleri*	T	x
Procellariidae	*Puffinus carneipes*		
Procellariidae	*Puffinus creatopus*	T	x
Procellariidae	*Puffinus gravis*		x
Procellariidae	*Puffinus griseus*		
Procellariidae	*Puffinus tenuirostris*		
Procellariidae	*Puffinus nativitatis*		x
Procellariidae	*Puffinus puffinus*		x
Procellariidae	*Puffinus yelkouan*		
Procellariidae	*Puffinus auricularis*	T	x
Procellariidae	*Puffinus opisthomelas*		x
Procellariidae	*Puffinus gavia*		x
Procellariidae	*Puffinus huttoni*	T	x

Family	Species	IUCN	R
Procellariidae	*Puffinus assimilis*		
Procellariidae	*Puffinus lherminieri*		
Procellariidae	*Puffinus heinrothi*	T	
Procellariidae	*Pelecanoides garnotii*	T	
Procellariidae	*Pelecanoides magellani*		
Procellariidae	*Pelecanoides georgicus*		x
Procellariidae	*Pelecanoides urinatrix*		
Procellariidae	*Diomedea exulans*	T	x
Procellariidae	*Diomedea amsterdamensis*	T	x
Procellariidae	*Diomedea epomophora*	T	x
Procellariidae	*Diomedea irrorata*	T	x
Procellariidae	*Diomedea albatrus*	T	x
Procellariidae	*Diomedea nigripes*	T	x
Procellariidae	*Diomedea immutabilis*	T	x
Procellariidae	*Diomedea melanophris*	T	
Procellariidae	*Diomedea cauta*		
Procellariidae	*Diomedea chrysostoma*	T	x
Procellariidae	*Diomedea chlororhynchos*		x
Procellariidae	*Diomedea bulleri*	T	x
Procellariidae	*Phoebetria fusca*	T	x
Procellariidae	*Phoebetria palpebrata*		
Procellariidae	*Oceanites oceanicus*		
Procellariidae	*Oceanites gracilis*		x
Procellariidae	*Garrodia nereis*		
Procellariidae	*Fregetta tropica*		x
Procellariidae	*Fregetta grallaria*		x
Procellariidae	*Nesofregetta fuliginosa*	T	x
Procellariidae	*Hydrobates pelagicus*		
Procellariidae	*Oceanodroma microsoma*		x
Procellariidae	*Oceanodroma tethys*		x
Procellariidae	*Oceanodroma castro*		
Procellariidae	*Oceanodroma leucorhoa*		
Procellariidae	*Oceanodroma monorhis*		x
Procellariidae	*Oceanodroma tristrami*		x
Procellariidae	*Oceanodroma markhami*		x
Procellariidae	*Oceanodroma matsudairae*		x
Procellariidae	*Oceanodroma melania*		x
Procellariidae	*Oceanodroma homochroa*	T	x
Procellariidae	*Oceanodroma hornbyi*		
Procellariidae	*Oceanodroma furcata*		
Tyrannidae	*Nesotriccus ridgwayi*	T	x
Tyrannidae	*Elaenia ridleyana*	T	x
Furnariidae	*Aphrastura masafuerae*	T	x
Meliphagidae	*Myzomela rubratra*		x

Family	*Species*	*IUCN*	*R*
Meliphagidae	*Myzomela chermesina*	T	x
Meliphagidae	*Apalopteron familiare*		
Pardalotidae	*Gerygone modesta*	T	x
Vireonidae	*Vireo caribaeus*	T	x
Vireonidae	*Vireo gracilirostris*		x
Corvidae	*Colluricincla tenebrosa*		x
Corvidae	*Corvus kubaryi*	T	x
Corvidae	*Rhipidura lepida*		x
Corvidae	*Rhipidura kubaryi*		x
Corvidae	*Dicrurus aldabranus*		x
Corvidae	*Terpsiphone smithii*	T	x
Corvidae	*Terpsiphone corvina*	T	x
Corvidae	*Pomarea dimidiata*	T	x
Corvidae	*Pomarea nigra*	T	x
Corvidae	*Pomarea iphis*	T	x
Corvidae	*Pomarea mendozae*	T	x
Corvidae	*Pomarea whitneyi*	T	x
Corvidae	*Metabolus rugensis*	T	x
Corvidae	*Monarcha godeffroyi*		x
Corvidae	*Monarcha takatsukasae*	T	x
Corvidae	*Myiagra freycineti*		x
Corvidae	*Myiagra erythrops*		x
Corvidae	*Myiagra oceanica*		x
Corvidae	*Myiagra pluto*		x
Cinclidae	*Cinclus cinclus*		
Cinclidae	*Cinclus pallasii*		
Cinclidae	*Cinclus mexicanus*		
Cinclidae	*Cinclus leucocephalus*		x
Cinclidae	*Cinclus schulzi*	T	x
Muscicapidae	*Nesocichla eremita*		x
Muscicapidae	*Copsychus sechellarum*	T	x
Sturnidae	*Aplonis pelzelni*	T	x
Sturnidae	*Aplonis mavornata*		x
Sturnidae	*Aplonis cinerascens*	T	x
Sturnidae	*Aplonis opaca*		x
Sturnidae	*Aplonis feadensis*	T	x
Certhiidae	*Troglodytes tanneri*	T	x
Hirundinidae	*Pseudochelidon sirintarae*	T	
Hirundinidae	*Hirundo perdita*		
Pycnonotidae	*Hypsipetes crassirostris*		x
Zosteropidae	*Zosterops modestus*	T	x
Zosteropidae	*Zosterops conspicillatus*	T	x
Zosteropidae	*Zosterops rotensis*	T	x
Zosteropidae	*Zosterops hypolais*		x

Family	*Species*	*IUCN*	*R*
Zosteropidae	*Zosterops semperi*		x
Zosteropidae	*Zosterops natalis*	T	x
Zosteropidae	*Zosterops tephropleurus*		x
Zosteropidae	*Zosterops tenuirostris*	T	x
Zosteropidae	*Zosterops albogularis*	T	x
Zosteropidae	*Zosterops finschii*		x
Zosteropidae	*Zosterops cinereus*		x
Zosteropidae	*Zosterops oleagineus*		x
Zosteropidae	*Rukia longirostra*		x
Zosteropidae	*Rukia ruki*	T	x
Zosteropidae	*Cleptornis marchei*	T	x
Zosteropidae	*Apalopteron familiare*	T	
Zosteropidae	*Megazosterops palauensis*		x
Sylviidae	*Cettia annae*		x
Sylviidae	*Nesillas aldabrana*		x
Sylviidae	*Acrocephalus luscinia*	T	x
Sylviidae	*Acrocephalus syrinx*		x
Sylviidae	*Acrocephalus rehsei*	T	x
Sylviidae	*Acrocephalus aequinoctialis*		x
Sylviidae	*Acrocephalus caffer*	T	x
Sylviidae	*Acrocephalus mendanae*		x
Sylviidae	*Acrocephalus atyphus*		x
Sylviidae	*Acrocephalus kerearako*		x
Sylviidae	*Acrocephalus rimatarae*	T	x
Sylviidae	*Acrocephalus vaughani*	T	x
Sylviidae	*Acrocephalus taiti*	T	x
Sylviidae	*Acrocephalus rodericanus*	T	x
Sylviidae	*Acrocephalus sechellensis*	T	x
Nectariniidae	*Nectarinia dussumieri*		x
Passeridae	*Anthus longicaudatus*		
Passeridae	*Anthus antarcticus*		x
Passeridae	*Foudia sechellarum*	T	x
Passeridae	*Foudia flavicans*	T	x
Fringillidae	*Pyrrhula murina*	T	
Fringillidae	*Telespiza cantans*	T	x
Fringillidae	*Junco insularis*	T	x
Fringillidae	*Dendroica vitellina*		x
Fringillidae	*Rowettia goughensis*	T	x
Fringillidae	*Nesospiza acunhae*	T	x
Fringillidae	*Nesospiza wilkinsi*	T	x
Fringillidae	*Pinaroloxias inornata*	T	x
