# Supplementary material for: Projected Impacts of Climate and Land-Use Change on the Global Diversity of Birds
Source: PLoS Biol. 2007 Jun 5;5(6):e157. doi: 10.1371/journal.pbio.0050157 (PMC1885834; doi:10.1371/journal.pbio.0050157)
Supplement: Supplementary file 7 [file pbio.0050157.st001.doc]

**Table S1: Projected environmental change and its impact on birds – based on unrefined maps.**

|  |  |  |  |  | ≥ 50% Range Lost | | | |  | Threatened | | | |  | Extinct | | | | | | |
| --- | --- | --- | --- | --- | --- | --- | --- | --- | --- | --- | --- | --- | --- | --- | --- | --- | --- | --- | --- | --- | --- |
|  |  |  |  |  |  |  |  |  |  |  |  |  |  |  |  |  |  |  |  |  |  |
|  |  |  |  |  |  |  |  |  |  |  |  |  |  |  |  |  |  |  |  |  |  |
|  |  |  |  |  | Species | |  | Range lost to Agr. |  | Species *(IUCN)* | |  | Range lost to Agr. |  | Species *(IUCN)* | |  | Range lost to Agr. |  | Species lost to | |
|  | IPCC |  | Year |  |  |  |  |  |  |  |  |  |  |  |  |  |  |  |  | Agr. | Clim. |
|  |  |  |  |  |  |  |  |  |  |  |  |  |  |  |  |  |  |  |  |  |  |
|  |  |  |  |  |  |  |  |  |  |  |  |  |  |  |  |  |  |  |  |  |  |
| **TechnoGarden** | **B1** |  | ***2050*** |  | 448 |  |  | 43% |  | 170 | *(92)* |  | 54% |  | 48 | *(29)* | | 43% |  | 20 | 27 |
|  |  |  |  |  |  |  |  |  |  |  |  |  |  |  |  |  |  |  |  |  |  |
|  |  |  | ***2100*** |  | 988 |  |  | 45% |  | 253 | *(112)* |  | 63% |  | 51 | *(31)* | | 45% |  | 22 | 27 |
|  |  |  |  |  |  |  |  |  |  |  |  |  |  |  |  |  |  |  |  |  |  |
| **Adapting Mosaic** | **B2** |  | ***2050*** |  | 398 |  |  | 39% |  | 216 | *(91)* |  | 57% |  | 48 | *(24)* | | 39% |  | 18 | 27 |
|  |  |  |  |  |  |  |  |  |  |  |  |  |  |  |  |  |  |  |  |  |  |
|  |  |  | ***2100*** |  | 952 |  |  | 48% |  | 323 | *(118)* |  | 58% |  | 61 | *(32)* | | 48% |  | 26 | 27 |
|  |  |  |  |  |  |  |  |  |  |  |  |  |  |  |  |  |  |  |  |  |  |
| **Global Orchestration** | **A1** |  | ***2050*** |  | 540 |  |  | 70% |  | 214 | *(97)* |  | 72% |  | 52 | *(25)* | | 70% |  | 34 | 12 |
|  |  |  |  |  |  |  |  |  |  |  |  |  |  |  |  |  |  |  |  |  |  |
|  |  |  | ***2100*** |  | 1,767 |  |  | 72% |  | 380 | *(151)* |  | 71% |  | 73 | *(40)* | | 72% |  | 46 | 14 |
|  |  |  |  |  |  |  |  |  |  |  |  |  |  |  |  |  |  |  |  |  |  |
| **Order from Strength** | **A2** |  | ***2050*** |  | 906 |  |  | 79% |  | 261 | *(96)* |  | 80% |  | 51 | *(26)* | | 79% |  | 37 | 9 |
|  |  |  |  |  |  |  |  |  |  |  |  |  |  |  |  |  |  |  |  |  |  |
|  |  |  | ***2100*** |  | 1,804 |  |  | 84% |  | 456 | *(161)* |  | 81% |  | 80 | *(41)* | | 84% |  | 60 | 9 |
|  |  |  |  |  |  |  |  |  |  |  |  |  |  |  |  |  |  |  |  |  |  |
|  |  |  |  |  |  |  |  |  |  |  |  |  |  |  |  |  |  |  |  |  |  |

Summary of projected impact of environmental change on birds under the four socioeconomic scenarios of the Millennium Ecosystem Assessment – *based on unrefined extent of occurrence maps*. Counts of species are given for three threat categories, those that lose at least 50% of their current range, those that will most likely be considered threatened under the current IUCN criteria (≥ 50% range loss and under 20,000km^2^ predicted range size), and those that are predicted to go extinct (100% range loss). In the ‘≥ 50% Range Lost’ category additionally counts of species with ≥ 50% range lost exclusively (*excl.*) due to each threat type (climate change, *Clim.,* and human land-use change, *Hab.*) are given. Numbers in brackets indicate the count of species in a category that are currently listed as “Threatened” by IUCN. The percentage of range size lost to agriculture (*Hab.*) as opposed to climate change (100% minus this value) is given for each category. All our calculations assume the ranges of the species remain stationary and have insufficient time to adapt to predicted climate or land use changes.
